# Initial quantitative development of the Norse Feedback system: a novel clinical feedback system for routine mental healthcare

**DOI:** 10.1007/s11136-021-02825-1

**Published:** 2021-04-13

**Authors:** Andrew A. McAleavey, Samuel S. Nordberg, Christian Moltu

**Affiliations:** 1 Center for Health Research, Helse Førde, Førde, 6807 Norway; 2 Harvard School of Population Medicine, District General Hospital of Førde, Cambridge, MA UK; 3 District General Hospital of Førde, Førde, Norway; 4 Department of Health and Caring Sciences, Western Norway University of Applied Science, Førde, Norway; 5 Weill Cornell Medical College, New York, NY USA; 6 Helse Førde, Førde, Norway

**Keywords:** Routine outcome monitoring, Clinical feedback systems, Item response theory, Measure development, Psychometric scale analysis

## Abstract

**Purpose:**

As routine outcome monitoring has become prevalent in psychological practice, there is need for measurement tools covering diverse symptoms, treatment processes, patient strengths, and risks. Here we describe the development and initial tests of the psychometric properties of a multi-scale system for use in mental healthcare, Norse Feedback.

**Methods:**

In Study 1, we present the item-generation process and structure of the Norse Feedback, a 17-scale digital-first measurement tool for psychopathology and treatment-relevant variables. In Study 2, we present analyses of this initial measure in a nonclinical sample of 794 healthy controls and a sample of 222 mental health patients. In Study 3, we present the analysis of a revised 20-scale system in two separate samples of patients. In each analysis, we investigate item and test information in particular, including analysis of differential item functioning on gender, age, site, and sample differences where applicable.

**Results:**

Scales performed variably. Changes to items and scales are described. Several scales appeared to reliably discriminate individuals entering mental health treatment on severity, and others are less reliable. Marked improvements in scale internal consistency and measurement precision were observed between the first and second implemented versions.

**Conclusion:**

This system includes some scales with reasonable structural validity, though several areas for future development are identified. The system was developed to be iteratively re-evaluated, to strengthen the validity of its scales over time. There are currently a number of limitations on inferences from these scores, which future developments should address.

**Supplementary Information:**

The online version contains supplementary material available at 10.1007/s11136-021-02825-1.

Practice in mental health has come to rely on measurement of patient symptoms at regular intervals, also known as routine outcome monitoring (ROM) [1, 2]. Several commonly used measurement instruments also provide clinical feedback systems (CFS; e.g., [3–5]), which may help clinicians adjust treatment and prevent deterioration during psychotherapy. Standardized self-report measurements are now considered best practice in many psychotherapy settings [6], and randomized trials have found encouraging, but inconsistent, treatment effects of using ROM/CFS [7–9].

There are a number of constraints on the measure development of a ROM/CFS system. Such instruments must be appropriate for heterogeneous patients, necessitating great breadth [10]. They also need to be appropriate for use in clinical settings, so are often brief [11, 12]. Clinicians report that some instruments fail to assesses their treatment targets [13], and many patients report that their goals for change are not captured by common measures [14]. Thus, there are patients and therapists who do not find brief, broad measures useful [15].

In this manuscript we present the Norse Feedback (NF), a new ROM/CFS designed to address these needs of patients and clinicians. A key tenet of its development has been iterative measure development based both on psychometric and clinical data to maximize clinical utility. We report the first quantitative studies on the development and performance of the first two NF versions. The outcome of this manuscript is not a final measure, but rather, a depiction of the NF at present, which is intended to be revised and iteratively improved in the future.

## Study 1

In this study we describe the initial development and initial implementation of the NF. Analysis of the perceived needs of a new ROM system began with focused qualitative analysis of interviews with mental health patients and clinicians, described elsewhere [16]. This led to several specific goals for a new ROM.

The most significant deviation from many existing ROM tools that provider and patient interviews [16] revealed was a preference for several measurement targets, including specific symptoms and other relatively narrow constructs, mirroring clinical assessment and case conceptualization. Many ROM/CFS measures are broad general distress measures [4, 5, 17], rather than measures of narrow constructs defined by practitioners. Moreover, research suggests that global distress measures omit significant issues from the vast majority (95%) of patients who would choose to track something not included in one of these standard instruments [14]. Patients and providers also reported wanting ROM/CFS to measure trans-diagnostic constructs, not diagnostic severity. In addition, patients and providers requested measures of trust, openness, life goals, and functioning.

Therapists, while invested in monitoring symptoms and risk, also wanted ROM/CFS to focus on functional and phenomenological aspects of recovery. Patients and providers both requested that ROM/CFS facilitate difficult conversations between patient and therapist: about the alliance, miscommunications, and treatment style. Lastly, both patients and providers wanted strengths-based information [16]. These findings are consistent with a meta synthesis of patient experiences with ROM tools, which emphasized the need for such instruments to capture complexity and support collaborative practice [18].

To address these needs, we sought to develop a measurement tool that was both broad and specific. Early in planning, we decided that the system would require multiple scales with different narrow constructs. As a guiding example, rather than a scale for Major Depressive Disorder, we created separate scales for several related trans-diagnostic features like negative affect, rumination, and demoralization. As targets for assessment, we included many common mental health symptoms/problems as well as markers of functioning and wellbeing. We also planned to adopt continuous quality improvement to respond to newly identified challenges [19]. This required a concomitant implementation and development process, in which we iteratively developed the measure, made it available for use, and evaluated its performance.

### Initial item development

On the basis of the reported needs from patients and clinicians, initial items were conceived and written in a three-day event convened for the purpose of translating qualitative findings into a psychometric instrument. Two clinical psychologists who had been involved in the qualitative study (SSN and CM) followed a process that cycled through three stages: identifying targets for assessment through targeted discussions with clinician and patient stakeholders followed by and qualitative theme-building based heavily on the themes identified by patients and therapists in [16]; independently developing individual items that were thought to indicate those targets; and then building an initial item set through consensus. In some cases, patients with prominent specific symptoms provided informal suggestions for items relevant to their treatment (e.g., patients with eating disorders provided suggestions for relevant items). One of the outcomes of this meeting was the decision that further development should include a wider variety of stakeholders, especially patients and clinicians, in item development. The 17 targets for assessment identified by this process are described in Table 1.

**Table 1:** Scales from Norse Feedback 1.0

Scale name	Brief description	# Items
Attachment	Orientation to others in close relationships	4
Avoidance	Fear-based avoidance of various stimuli	6
Connectedness	Feeling of closeness to other people, social relatedness	7
Demoralization	A sense of loss of certainty that improvement will occur	5
Eating problems	Maladaptive thoughts and behaviors related to food	6
Emotional Distancing	Internal avoidance of negative feeling states	2
Hurtful rumination	Repetitive negative thought; worry and depressive rumination	6
Hypervigilance	Over-awareness of potential physical threats, especially in public	4
Perfectionism	A need for control that interferes; unacceptance of compromise	6
Pressure from Negative Affect	General negative affect	9
Psychosis	Frank psychosis/hallucinations, paranoia	3
Relational distress	Problems in close relationships	7
Resilience	Strength factors, self-efficacy for recovery	12
Social Role Functioning	Overall self-description of performance at work, home, and socially	4
Somatic Anxiety	Symptoms of physical anxiety	6
Substance Use	Problematic alcohol and drug use	4
Suicide Risk	Conscious suicidal ideation and impulsivity	4

English labels used here, scale developed in English and Norwegian simultaneously

This process resulted in 90 items consensually believed to relate to these scale targets, with some items scored on multiple scales. Items were to be rated on a seven-point Likert scale, with a stem focused on the patient’s sense of themselves in the past week, anchored at “This is not at all true for me” and “This is completely true for me.”

Additionally, five items were developed to assess the therapeutic alliance, primarily targeting elements of Bordin’s tripartite model [20], and four items to collect feedback from patients on the therapy process because these were of strong interest to patient and provider stakeholders in the earlier qualitative study. These items were determined to require a separate revision process because they related to therapy process rather than patient variables and are not described in this manuscript. The system was intended to be used exclusively through digital technology, and particularly mobile devices. The NF is intended primarily to be completed by patients and reviewed by clinicians before clinical encounters. In this way, it would not occupy in-person time, would not require additional technology at the clinical environment, and would allow patients to create a private environment for themselves to complete the questionnaire.

After an initial version of the instrument was completed, we deployed it briefly at one hospital, both for a non-patient population and a specialist mental health care patient population. This pilot found that the system required roughly 15 min on average per administration. Given clinical experience and recommendations from other sources [12, 17], we aimed to reduce this substantially, especially for repeated use in clinical settings. This led to the development of a semi-independent scale system, wherein individual scales are modularly assigned to patients after an initial assessment in which all scales are completed. Scale assignment is presently based on severity, and only pertains to post-initial administrations of the NF [19]. Given this, the NF can be thought of as similar to a battery of separable tests, rather than a single instrument. In principle, each scale is designed to be administered independent of the others. While this does not address the length of the initial administration, it should greatly reduce the time burden at later administrations while retaining consistent items and scale content across repeated assessments.

### Discussion

In this study we have described initial development of the items and structure of the Norse Feedback, a novel multi-scale system for routine outcome monitoring in mental healthcare. This tool was implemented by a technological partner and made available through data-secure internet protocols. In subsequent studies, we describe the evaluation and revision of this tool. These studies cover the initial assessment only, not questions related to change during treatment, which is beyond the scope of this manuscript.

## Study 2

The goal of this study was to test the performance of this instrument in clinical and nonclinical samples. We were primarily interested in the reliability and validity of individual scale scores, as opposed to the performance of the NF tool as a whole, because the NF scales are designed to be algorithmically selected, independently of one another at post-initial administrations.

### Methods

#### Participants

The nonclinical sample included 794 respondents, comprised of 637 hospital employees (from 2000 invited), 109 college employees (from 400 invited), and 48 students (from 700 invited) who responded to electronic request for study participation. Most (616, 78%) were female, and 36 self-identified as current mental health patients. This sample was highly educated, with 222 reporting completing 4 years of college/university, 170 completing a Master’s degree, and 30 completing a doctoral degree; an additional 142 had completed 3 years of college and 74 completed a vocational certificate, with only 149 either not graduating high school or only graduating high school. The majority, 764 (98%), identified as heterosexual.

The clinical sample was comprised of 222 unique patients in inpatient (41) and outpatient (171) mental healthcare in the same locale, who completed the NF as part of routine care (demographic data only available for 212 patients). Only the intake administrations, in which all NF scales were administered to all participants, were used in this study. The majority, 142 (67%), were female. In this sample, high school graduate was the most common educational status (85), with 48 participants completing 3 or 4 years of college, 39 completing a vocational certificate, 28 not completing high school, and 6 with a Master’s. The majority, 190 (96%), identified as heterosexual.

#### Measures

The Norse Feedback instrument as described in Study 1.

#### Procedure

Prior to scheduled appointments at the mental health service, clinical participants were provided a 48-h period during which they could access the NF measure through secure URL. Nonclinical participants were recruited via email with credentials to a secure website. All data were anonymized prior to analysis. The project was determined exempt by the REC (2018/993/Regional Committees for Medical and Health Research Ethics, North) from the Act on medical and health research and conducted in accordance with local institutional Data Protection Officer.

#### Data analysis

Our primary interest was the performance of individual scales, because each scale may be administered in isolation. As such, our primary analyses treated scales as separate unidimensional scales, rather than one multidimensional scale. While multidimensional analyses would likely provide benefits to precision and accuracy of estimates, this was determined to be less than optimal for two reasons. First, interpretability of multidimensional models was thought to be less clear than a simple one scale per item rule, particularly for clinicians and patients. Second, the longer-term vision for the CFS was to allow for algorithmically based scale selection, especially after an initial administration of all scales. This would lead to potentially independent scale administration, rather than consideration of all items on the NF at once.

We conducted testing for unidimensionality in multiple ways. First, eigenvalues were extracted and plotted in a scree plot with visual inspection. A very large first eigenvalue and lower remaining eigenvalues (especially below 1.0) was considered to indicate unidimensionality especially when the first eigenvalue accounted for greater than 20% of the total variance [21]. Further, 20 simulated data sets of equal size and character were generated, and their eigenvalues were compared, with unidimensionality supported if only one eigenvalue from the true data set exceeds the simulated random data [22]. The ratio of first to second eigenvalues was calculated, with values greater than 5 indicating strong evidence of unidimensionality [23]. We also conducted single latent variable confirmatory factor analyses on each scale, assessing item loading values and model fit. Several indicators from classical test theory were computed as well, including Cronbach’s alpha, item-total correlations, and these values for the scale with each item removed.

IRT consisted of the graded response model [24], a parametric IRT model common when using ordered polytomous data. We examined item characteristic curves, test information functions, and item information functions generated in the R program ltm [25]. Test information was interpreted with particular focus, where higher values of test information were generally more desirable. Test information is the inverse of the scale’s error variance at the level of the latent “ability” (in this case symptom or problem severity). Information in this sense provides a robust single marker for test precision, and its graphical representation shows what range of the latent trait is well-measured. More specifically, scales with peak test information below 5 (analogous to Cronbach’s alpha = 0.8) were considered to require substantial revision, and scales with peak test information over 10 (analogous to alpha = 0.9) were considered to be performing generally well, therefore requiring less revision for psychometric reasons at present. Wider ranges of high test information across the latent trait were desirable, though high peak information over narrow ranges was sometimes quite important for clinical reasons.

When differential item functioning (DIF) testing was possible given minimum 4 items per scale, we used iterative ordinal logistic regression [26] with the lordif R package [27], which performs automatic search for DIF using the mirt R package [28] and quantification of the impact of DIF on scale scores via iterative purification. We used a conservative alpha level of 0.01 to detect DIF as recommended [27], and compare magnitude of effect sizes using McFadden’s pseudo *R*^2^. In this study, we investigated DIF as a function of sample (clinical vs. nonclinical) and self-reported gender across both samples. As an initial validity test, we also conducted receiver-operator characteristic (ROC) curve analyses for each scale score, to test ability of the scales to discriminate between psychiatric patients (the clinical sample) from nonclinical sample.

Finally, we conducted exploratory factor analyses (EFA) and principle components analysis (PCA) of all items to inform subsequent revision, though these were secondary analyses due to the NF scale selection rules. The intention in conducting these analyses was mainly to identify groups of highly intercorrelated items, which might represent clinically important constructs for development in future versions of the NF. Our primary implementation of EFA was Geomin rotation and WLSMV estimation in Mplus. As sensitivity analyses, we also conducted several similar analyses to assess robustness of this factor solution. For these analyses, we used the psych package (v. 1.8.12) in R [29] and Mplus [30], omitted correlated error variances among the items, tested a variety of estimation and rotation methods (ML, minres, WLS, PLS, GLS, WLSMV), and compared different methods of determining the number of extracted factors (eigenvalues greater than 1 and parallel analysis).

### Results

**Fig. 1:**
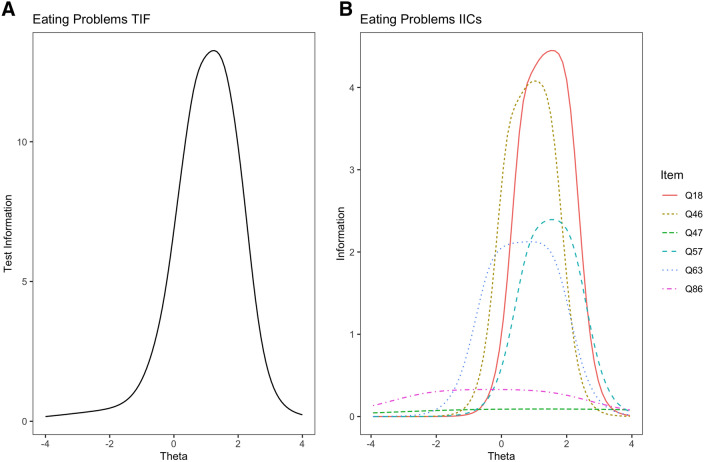
Test and item information for the Eating Problems scale of the NF in Study 2

The IRT analyses of the 17 scales of the NF had varying outcomes. All showed acceptable to strong evidence of unidimensionality. Complete analysis results per scale are available in the supplementary materials^1^ and we present only an illustrative example here. The Eating Problems scale of the NF displayed an overall good test information function in the clinical sample (Fig. 1a), with a relatively high peak test information over 13. The ability levels at which acceptable information was found were relatively narrow on this scale, extending from the range [− 0.2, 2.5] SD of the latent trait. A slightly positively shifted range of test information can be expected on this latent variable, as the observed scores are decidedly positively skewed with many mental health patients having no eating problems and only a relatively severe minority requiring highly informative assessment. However, examination of the item information curves (Fig. 1b) showed that at least two items are not performing well in this scale. These two items contribute almost no information at any level of the latent trait, while the remaining five items constituted the entire range of information here. Moreover, a single item contributes about 40% of the total marginal information, suggesting that this highly discriminating item is essential to the scale. Summaries of the other scales’ performance on these tests are in Table 2.

**Table 2:** Scales from Norse Feedback 1.0: Performance summary in Study 2

Scale name	Peak Test Information	Location of peak test information (*θ*)	Range of test information > 5	Cronbach's Alpha	Eigenvalue ratio	Proportion of variance explained	TLI	RMSEA	AUC
Attachment	2.03	-0.42	–	0.61	2.09	0.28	0.8	0.12	0.52
Avoidance	3.59	0.18	–	0.66	2.15	0.28	0.86	0.09	0.86
Connectedness	3.71	0.30	–	0.71	2.51	0.28	0.76	0.11	0.72
Demoralization	6.07	0.30	[− 1.03, 1.39]	0.80	3.74	0.46	0.99	0.02	0.85
Eating problems	13.26	1.25	[− 0.20, 2.46]	0.75	3.19	0.42	0.98	0.05	0.68
Hurtful rumination	4.42	−0.06	–	0.76	2.98	0.39	0.85	0.13	0.86
Hypervigilance	2.37	−0.42	–	0.50	1.64	0.26	0.85	0.09	0.81
Perfectionism	3.59	0.42	–	0.73	2.70	0.28	0.82	0.10	0.78
Pressure from Negative Affect	6.65	−0.06	[− 1.76, 1.39]	0.76	3.08	0.31	0.91	0.07	0.87
Psychosis	1.86	2.00	–	0.41	1.63	0.22	NA	NA	0.72
Relational distress	4.36	0.67	–	0.75	2.81	0.31	0.86	0.09	0.77
Resilience	4.52	0.18	–	0.77	2.51	0.23	0.61	0.11	0.77
Social Role Functioning	8.31	0.30	[− 1.76, 1.15]	0.66	2.18	0.52	NA	NA	0.54
Somatic Anxiety	5.31	−0.06	[− 0.79, 0.91]	0.76	2.92	0.37	0.89	0.10	0.86
Substance Use	15.25	1.27	[0.18, 2.36]	0.92	9.68	0.75	0.95	0.15	0.61
Suicide Risk^*^	10.10	1.27	[0.06, 2.36]	0.68	2.41	0.36	0.91	0.09	0.75

Emotional Distancing scale only had 2 items in this version, scale analysis not conducted. *: One item was removed from the Suicide Risk scale prior to analysis, due to linear separation issues causing nonconvergence. Eigenvalue ratio is the ratio of first to second eigenvalues. Proportion of variance explained indicates the proportion of explained variance in a one-factor solution

*TLI* Tucker-Lewis Index, *AUC *Area under the receiver-operator characteristic curve separating clinical and nonclinical samples

In DIF analyses, the Eating Problems scale did show statistically significant, but negligible to small (McFadden’s *R*^2^ < 0.02, [31]) uniform DIF across samples on all 6 items, and similar non-uniform DIF on two items. There was also statistically significant evidence of uniform DIF and non-uniform DIF related to gender in two items, both with negligible effect sizes (McFadden’s *R*^2^ < 0.005). Given these very small effect sizes for DIF, interpretations of scale score comparisons across samples for the Eating Problems scale should not be detrimentally affected. However, the Eating Problems scale did not discriminate between clinical and nonclinical samples particularly well. The total area under the curve (AUC) for this scale was 0.68. This relatively poor performance is likely related to the low base rate of eating-related pathology in the clinical sample, compared to other more common concerns (mood, anxiety, and interpersonal distress). Note in Supplementary Table S1 that Connectedness, Demoralization, Pressure from Negative Affect, Relational Distress, and Somatic Anxiety did show substantial DIF between the clinical and nonclinical samples. Scores on these scales should not be interpreted directly across these samples. Results of DIF analyses across genders are in Supplementary Table S2. No items on any other scale showed DIF with greater than small effect size (McFadden’s *R*^2^ < 0.02) across genders (see Supplement).

The factor analyses using different methods showed a range of potential number of factors to extract, as expected. While there were 20 eigenvalues greater than 1, parallel analysis using factor analysis supported up to 14 factors, and parallel analysis using principle components suggested up to 9 components were present. Our primary Mplus implementation of EFA with WLSMV estimation suggested that a 12-factor solution was optimal. As opposed to the 13- and 14-factor solutions, all factors in this solution had at least 2 items with a standardized factor loading over 0.4, and all factors had relatively clear interpretations. Factor loadings for this solution are presented in Supplementary Table S3.

### Discussion

This study was the first substantial quantitative analysis of the reliability and validity of the NF scales. While unidimensionality was supported across all scales, there were clear areas for improvement as well. For instance, nine scales did not achieve test information over 5 at any theta value, our a priori minimally acceptable level. These were prioritized for further development for psychometric reasons. The process for revising the instrument included this psychometric input alongside direct feedback from users on the experience of using the tool. Briefly, the psychometric results were synthesized into clinician-focused summaries for each scale and item, along with a reorganization for the scales of the NF. This quantitative information was presented to a group of researcher-clinicians at a 2-day event convened specifically for this purpose. This group examined the factor analysis, IRT results, and discussed their experience of the scales’ fit to their patients’ reported experience. The factors identified in the EFA were considered alongside the IRT analysis, especially when substantial restructuring was required. That is, items that contributed to poorly performing scales in the IRT model were examined for loading patterns with other items in this EFA, which helped generate ideas for new potential targets of assessment in subsequent revision and identify reasons for misfit. The suggestions of clinicians were then taken to a group of patients for review and further suggestions.

This process, which is described further in [19], led to a new 102-item Norse Feedback measure with 20 patient subscales following a four domain structure (symptom expression, problem maintaining processes, resources, and personal consequences), three treatment process scales (alliance, needs in treatment, and medication), and five single-item assessments that do not load on any scales. Many of the worst-performing scales on psychometric analyses were substantially revised or reorganized. The scales are described in Table 3. This new version was implemented in clinical practice, and its performance is reported in the Study 3.

**Table 3:** Scales from Norse Feedback 2.0: Organization, item retention, construct coverage

Domain	Scale	Description	Number of Items	Number of Items from Previous Norse
Symptom Expression	Eating Problems	Disordered eating and body image	5	4
	Sad Affect	Negative, especially sad, feeling state	4	1
	Somatic Anxiety	Physical markers of anxiety/fear response	5	4
	Substance Use	Problematic use of drugs and/or alcohol	4	3
	Suicide	Thoughts, impulses, and plans related to suicide	4	2
	Trauma Reactions	Re-experiencing and intrusive memories, hypervigilance to threat	4	0
Resources	Readiness for Recovery	Stage of change in behavior	3	0
	Recovery Environment	External supports for behavior changes	5	1
	Social Safety	Sense of comfort and emotional support in close relationships	6	4
Problem-maintaining processes	Need for Control	Problematic need for control, perfectionism	4	3
	Hopelessness	Expectation of effort futility	5	4
	Internal Avoidance	Attempts to avoid feelings and thoughts	5	3
	Irritability	Frequent interpersonal conflict, feelings of anger	3	2
	Self-Criticism	Conscious negative self-statements	7	2
	Situational Avoidance	Avoidance of external stimuli due to fear	3	1
	Social Avoidance	Avoidance of social situations due to fear	3	1
	Worry	Conscious repetitive anxious apprehension	3	1
Personal Consequences	Cognitive Problems	Decrements in concentration and cognition	6	0
	General Functioning	Sense of functioning in work, family, social domains	3	1
	Substance Recovery	Sense of progress in managing substance use problems (only open when Substance Use scale is open)	4	0

## Study 3

The second version of the Norse Feedback system was implemented after study 2. In evaluating performance of the second-generation scales, we emphasized similar IRT-based analyses that were presented in Study 2.

### Methods

#### Participants

Data for this study derived from routine use of the NF at two large specialist mental health services in Western Norway, which were the two earliest adopters of the NF in practice and collected large volume of data prior to analysis. One is located in one of the larger cities in this region, and the other in a smaller city. Both are providers in Norway’s national health system. At Site 1, data were collected from October 2017 to January 2019, during which time 617 individuals completed an intake with this version of the NF and are included in this study. At Site 2596 individuals completed an intake from May, 2018 to January 2019. Data were anonymized prior to analysis and research ethics compliance is identical to Study 2.

#### Measures

NF, as described above.

#### Procedures

Participants were electronically notified that they had been assigned an administration of the NF to complete within 48 h, prior to scheduled appointments, via secured internet connection. Identical classical test theory and IRT analyses were conducted using the same procedures described in Study 1.

#### Data analysis

One of the key stakeholder-driven study targets in this study was examination of site-based DIF, which was considered plausible based on the different catchment populations of these two services. Specifically, the two sites differ by city size and diversity, with one located in one of the largest cities in Norway and the other in a relatively small town (pop. approx. 10,000). Both clinical sites also have slightly different integration of clinical services and therefore specialize in slightly different treatments. Accordingly, clinicians reported concern that the patient populations may not be equivalent across sites, which posed challenges for future development processes involving patients and providers from several sites. Therefore, we wished to share site DIF analyses with clinical stakeholders in future development. We used methods described earlier to evaluate item performance within each subscale, as detailed in Study 2. We first tested DIF across sites, and across available demographic variables of gender and age (median split at 29.15 years). We additionally examined item floor/ceiling effects, response frequencies, scale score distributions, and inter-scale score correlations.

### Results

**Fig. 2:**
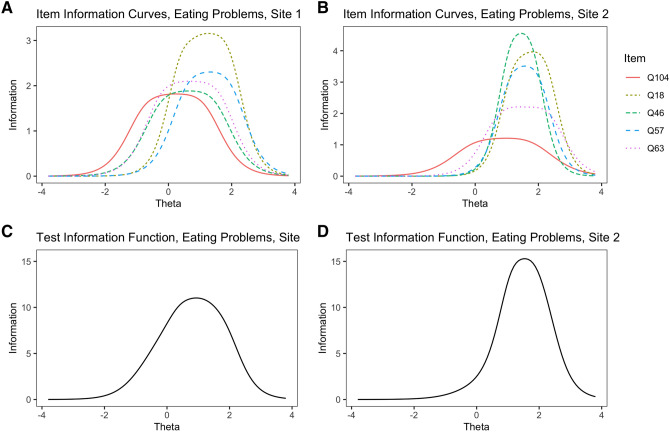
Item and test information functions for the Eating Problems scale of the NF in Study 3

Similar to Study 2, summaries and an illustrative example are presented here, with the complete results in supplementary materials. Table 4 contains summaries of each scale total information peaks and range of the latent trait on which the scale’s information was greater than 5. Item information functions of the Eating Problems scale are presented in Fig. 2a and b, and the test information functions are in 2C and 2D. The Eating Problems scale was revised after Study 2 to remove two items and include one new item suggested by clinicians. The overall test information functions suggest that it strongly discriminates among patients in both samples, especially at the higher end of the latent trait. This is expected and appropriate for a latent trait that is most clinically relevant at higher levels.

**Table 4:** Scales of Norse Feedback 2.0: Summary results of Study 3

Domain	Scale	Peak Test Information		Location of peak test information (θ)		Range of test information above 5		Cronbach's Alpha		Eigenvalue ratio	Proportion of variance explained			TLI		RMSEA	
*Site 1*																	
Symptom Expression	Eating Problems	11.03	0.93		[− 0.53 2.22]		0.86		5.76		0.56		0.95				0.11
	Sad Affect	8.82	0.12		[− 2.06, 1.33]		0.84		4.67		0.66		NA				NA
	Somatic Anxiety	5.44	− 0.04		[− 1.09, 0.93]		0.80		4.00		0.46		0.93				0.10
	Substance Use	14.18	2.14		[0.53, 3.27]		0.85		6.06		0.59		1.00				0.01
	Suicide						dnc										
	Trauma Reactions	9.32	0.61		[− 0.36, 1.49]		0.74		3.00		0.47		1.00				0.03
Resources	Readiness for Recovery	0.92	− 0.53		–		0.41		1.55		0.20		NA				NA
	Recovery Environment	2.40	0.85		–		0.64		2.58		0.27		0.98				0.03
	Social Safety	4.27	− 0.20		–		0.75		2.79		0.35		0.87				0.11
Problem-maintaining processes	Need for Control	2.48	− 0.04		–		0.62		1.95		0.30		0.57				0.19
	Hopelessness	6.63	− 0.20		[− 1.58, 1.49]		0.81		3.04		0.48		0.87				0.15
	Internal Avoidance	5.46	− 0.44		[− 1.41, 0.85]		0.73		2.68		0.40		0.98				0.04
	Irritability	2.26	0.93		–		0.45		1.64		0.28		NA				NA
	Self-Criticism	12.10	0.20		[− 1.66, 2.06]		0.89		5.59		0.50		0.94				0.09
	Situational Avoidance	3.70	0.53		–		0.65		2.33		0.41		NA				NA
	Social Avoidance	5.56	0.04		[− 1.01, 1.25]		0.75		3.42		0.51		NA				NA
	Worry	6.25	− 1.01		[− 1.98, 0.44]		0.79		4.25		0.57		NA				NA
Personal Consequences	Cognitive Problems	11.34	− 0.04		[− 1.74, 1.9]		0.89		4.88		0.58		0.75				0.25
	General Functioning	1.98	− 0.69		–		0.56		2.08		0.33		NA				NA
	Substance Recovery	5.22	1.58		[1.25, 1.82]		0.50		1.75		0.26		0.94				0.06
*Site 2*																	
Symptom Expression	Eating Problems	15.28	1.58		[0.44, 2.71]		0.84		5.19		0.53				0.96	0.09	
	Sad Affect	9.58	0.44		[− 1.66, 1.74]		0.84		4.80		0.66				NA	NA	
	Somatic Anxiety	5.75	0.28		[− 1.01, 1.25]		0.81		3.89		0.46				0.91	0.12	
	Substance Use	13.46	1.66		[0.53, 2.95]						0.76				0.96	0.15	
	Suicide	11.11	2.06		[1.01, 3.03]		0.82		6.17		0.59				1.00	0.00	
	Trauma Reactions	6.39	0.77		[− 0.44, 1.74]		0.77		3.39		0.48				0.99	0.05	
Resources	Readiness for Recovery	1.23	− 0.12		–		0.44		1.57		0.26				NA	NA	
	Recovery Environment	2.61	1.01		–		0.63		2.20		0.27				0.90	0.08	
	Social Safety	4.18	− 0.12		–		0.74		2.75		0.34				0.87	0.10	
Problem-maintaining processes	Need for Control	3.05	0.36		–		0.67		2.51		0.35				0.87	0.11	
	Hopelessness	7.27	0.44		[− 1.33, 1.66]		0.81		3.26		0.48				0.88	0.14	
	Internal Avoidance	6.54	− 0.12		[− 1.41, 1.33]		0.76		2.93		0.43				1.00	0.01	
	Irritability	4.65	1.74		–		0.45		1.56		0.40				NA	NA	
	Self-Criticism	15.96	0.77		[− 1.25, 2.55]		0.90		6.46		0.54				0.93	0.11	
	Situational Avoidance	3.41	0.61		–		0.63		2.22		0.41				NA	NA	
	Social Avoidance	6.62	0.44		[− 1.09, 1.66]		0.79		3.89		0.57				NA	NA	
	Worry	6.32	− 0.69		[− 1.9, 0.85]		0.81		4.60		0.59				NA	NA	
Personal Consequences	Cognitive Problems	13.45	0.36		[− 1.49, 2.06]		0.90		5.77		0.60				0.81	0.22	
	General Functioning	2.20	− 0.61		–		0.60		2.23		0.36				NA	NA	
	Substance Recovery						dnc										

Proportion of variance explained indicates the proportion of explained variance in a one-factor solution. *TLI* Tucker-Lewis Index, *AUC* Area under the receiver-operator characteristic curve separating clinical and nonclinical samples. *dnc* These analyses did not converge, likely because of a linear combination of answer responses or excess zeros in the sample. In other respects (i.e., sample descriptives, floor/ceiling effects), these data sets are highly comparable across sites. Eigenvalue ratio is the ratio of first to second eigenvalues. Some theta values and ranges have had their signs reversed from the analytic output, due to automatic item recoding algorithms

**Fig. 3:**
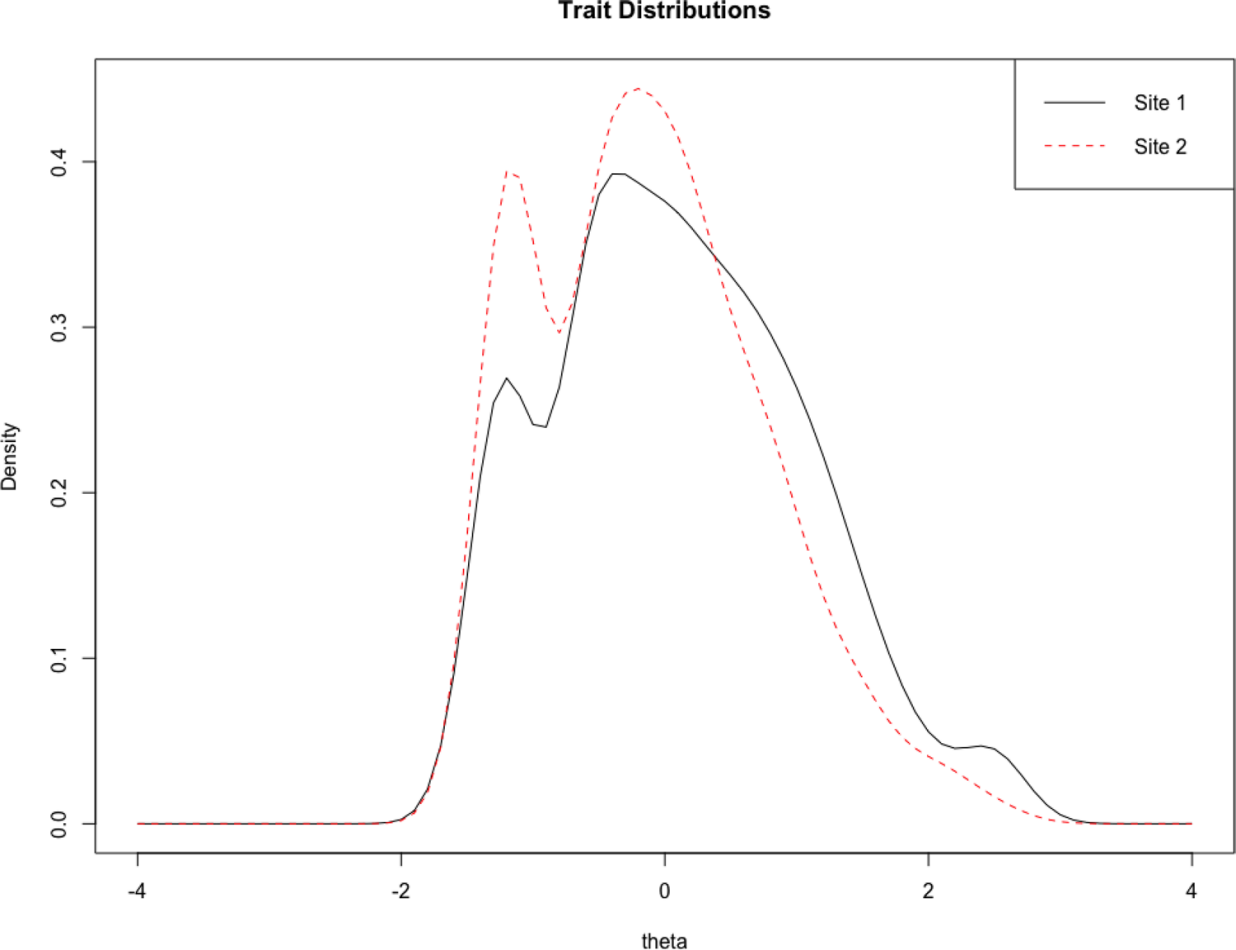
Eating Problems scale DIF: Trait distribution across the two sites in Study 3. Site 1 appears to include more patients who are higher on this latent trait, while Site 2 appears to have a slightly higher concentration of individuals at the lower levels of the trait. The bimodal appearance in both samples is driven by excess zeros in the responses, from participants with no self-reported eating concerns

**Fig. 4:**
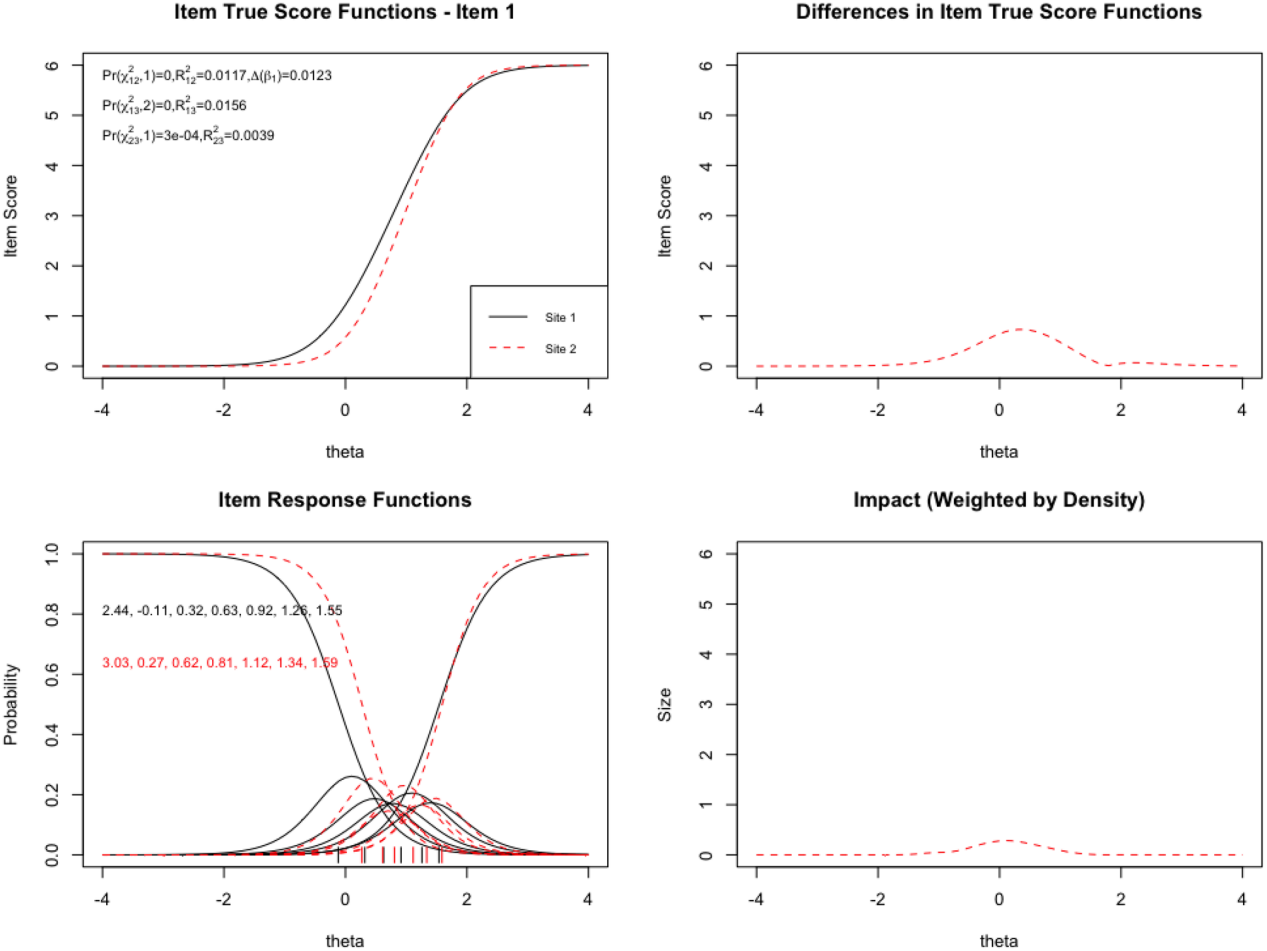
Eating Problems scale DIF: One item displays DIF across two sites in Study 3. One item, Q46, was identified as having DIF using logistic ordinal regression with iterative purification, while the others were nonsignificant. The item appears to have slightly higher difficulty in Site 2 than Site 1 (upper left). This is confirmed by comparison of the item response’s characteristic curves (lower left), which show a high degree of clustering of middle responses, possibly justifying a reduction in response options. However, both the unweighted (upper right) and weighted (lower right) plots of impact suggest that this item’s DIF will have minimal impact on observed scores across the samples, particularly after accounting for distribution

**Fig. 5:**
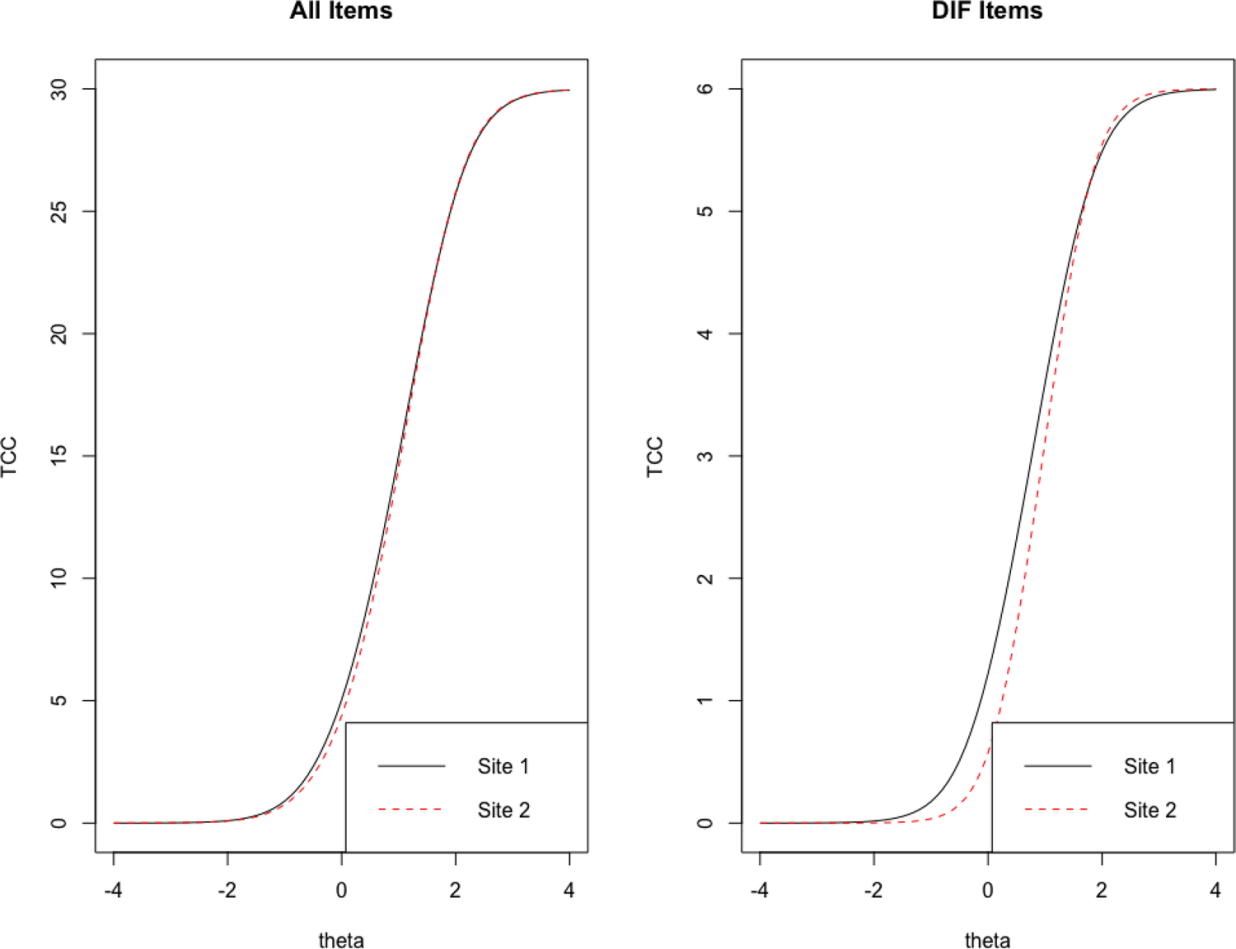
Eating Problems scale DIF: Test characteristic curves (TCCs) in Study 3. Though the one item with DIF does have the previously observed different pattern across sites (right), the test including that item (left) is nearly identical in functioning across the sites, confirming the minimal impact of this DIF

**Fig. 6:**
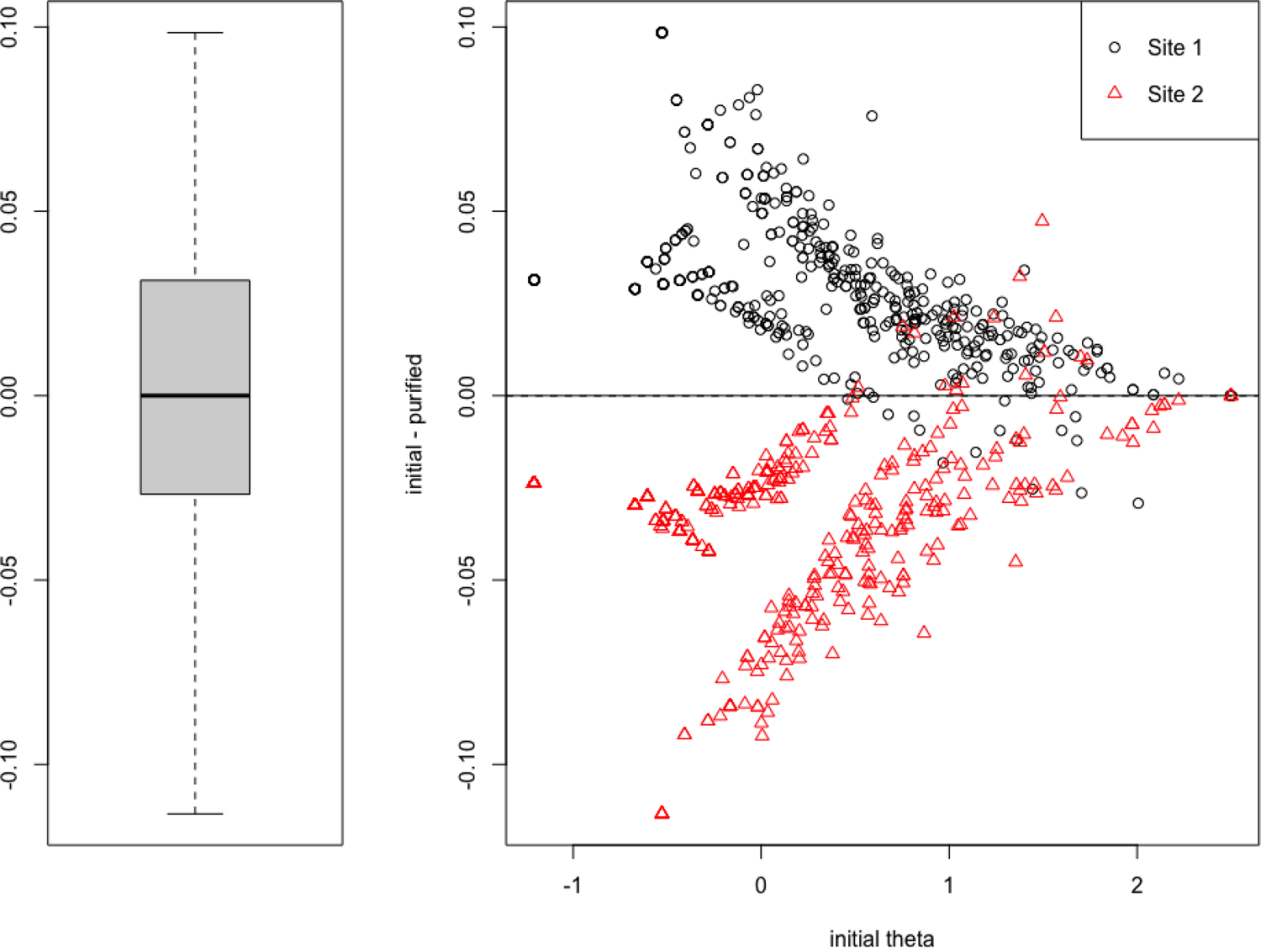
Eating Problems scale DIF: Impact of DIF across sites in Study 3. The DIF did affect scoring across the sites, though this was small in absolute magnitude (less than 0.1 SD), and entirely located in the less-severe range of the latent trait (right). This suggests that low scores at Site 2 should be very slightly lower, and low scores at Site 1 should be very slightly higher, than a DIF-naïve model. However, the objective magnitude of this change is small, and the location of concern (lower severity) is less clinically relevant than the higher severity range

Analyses site-based DIF on Eating Problems are presented in Figs. 3, 4, 5, 6. Across sites, there was a moderate difference in latent trait distributions, with Site 2 having slightly less high-pathology patients than Site 1. Only Q46 was found to have statistically significant DIF using logistic ordinal regression methods. In Site 2, this item appeared to have slightly higher difficulty and discrimination than in Site 1, with tests of uniform and non-uniform DIF both showing statistical significance (*p* < 0.001). However, the DIF effect size was small: McFadden’s R^2^ change for uniform DIF was 0.01, and for non-uniform DIF this value was 0.003.^2^ On the scale scores, these differences amounted to less than one scale point at most, and when weighted by density of responses, result in negligible changes to scores. The test characteristic curve using all items appears to be nearly unaffected by this difference. What differences are present appear to be exclusively at the low end of the latent trait. See Supplementary Table S3 for summaries of DIF by site on all scales. Analyses also revealed minimal DIF by age and gender across all scales of the NF (Supplementary Tables S4 and S5). On both gender and age, 18 items showed statistically significant DIF, but the magnitude of these effects was small to negligible: the largest effect size across all items was *R*^2^ = 0.02 (on Eating Problems), and most significant effects were below 0.005.

Further analyses for the other scales are included in the supplementary materials.

Table 5 shows correlations between the 20 scale scores. The correlations are quite consistent across sites. Overall, these scales show moderate to strong intercorrelations, with some exceptions. Only 3% of the correlations were greater than *r* = 0.6; 36% were in the range *r* = [0.4, 0.6]; 39% were within [0.2, 0.4]; and 22% were *r* < 0.2.

**Table 5:** Scale score intercorrelations at each site in Study 3

		1	2	3	4	5	6	7	8	9	10	11	12	13	14	15	16	17	18	19	20
1	Cognitive Problems	1.00	0.52	0.28	0.54	0.59	0.54	0.48	0.25	0.32	0.61	0.57	0.44	0.49	0.31	0.54	0.18	0.09	0.44	0.44	0.51
2	Need for Control	0.47	1.00	0.42	0.45	0.44	0.53	0.53	0.22	0.29	0.46	0.57	0.46	0.55	0.33	0.50	0.03	0.14	0.31	0.42	0.49
3	Eating Problems	0.26	0.37	1.00	0.26	0.32	0.34	0.32	0.17	0.22	0.30	0.45	0.27	0.28	0.20	0.27	− 0.15	− 0.03	0.27	0.23	0.24
4	General Functioning	0.51	0.42	0.21	1.00	0.62	0.50	0.35	0.42	0.43	0.58	0.60	0.41	0.47	0.41	0.44	0.18	0.00	0.39	0.27	0.44
5	Hopelessness	0.50	0.44	0.28	0.59	1.00	0.59	0.42	0.44	0.45	0.77	0.65	0.50	0.58	0.39	0.53	0.26	0.03	0.60	0.39	0.58
6	Internal Avoidance	0.45	0.45	0.30	0.42	0.57	1.00	0.42	0.26	0.31	0.59	0.60	0.53	0.58	0.57	0.52	0.28	0.14	0.40	0.48	0.59
7	Irritability	0.31	0.46	0.22	0.23	0.38	0.34	1.00	0.14	0.31	0.40	0.47	0.38	0.42	0.30	0.31	− 0.08	0.07	0.28	0.36	0.40
8	Readiness for Recovery	0.19	0.08	0.05	0.31	0.40	0.24	0.11	1.00	0.33	0.33	0.39	0.22	0.29	0.41	0.21	0.35	− 0.13	0.24	0.10	0.18
9	Recovery Environment	0.18	0.21	0.12	0.36	0.40	0.22	0.33	0.34	1.00	0.41	0.44	0.27	0.36	0.46	0.21	0.23	0.12	0.36	0.26	0.21
10	Sad Affect	0.47	0.37	0.26	0.57	0.73	0.55	0.34	0.34	0.37	1.00	0.64	0.47	0.60	0.38	0.60	0.16	0.09	0.60	0.43	0.61
11	Self-Criticism	0.45	0.56	0.38	0.57	0.65	0.57	0.34	0.23	0.34	0.61	1.00	0.49	0.62	0.51	0.45	0.15	0.08	0.54	0.40	0.49
12	Situational Avoidance	0.31	0.38	0.29	0.34	0.40	0.39	0.29	0.12	0.27	0.37	0.37	1.00	0.59	0.29	0.58	0.09	0.08	0.33	0.51	0.52
13	Social Avoidance	0.37	0.42	0.26	0.45	0.50	0.46	0.31	0.17	0.27	0.53	0.47	0.58	1.00	0.45	0.55	0.12	0.12	0.42	0.43	0.55
14	Social Safety	0.18	0.27	0.17	0.38	0.34	0.54	0.21	0.31	0.36	0.41	0.45	0.26	0.44	1.00	0.27	0.33	0.08	0.29	0.20	0.27
15	Somatic Anxiety	0.48	0.46	0.25	0.38	0.52	0.54	0.37	0.15	0.23	0.48	0.45	0.50	0.47	0.24	1.00	0.10	0.06	0.34	0.54	0.66
16	Substance Recovery	0.04	0.00	0.09	-0.03	0.04	0.09	0.10	0.20	0.15	0.00	0.06	0.03	0.07	0.27	0.09	1.00	0.36	0.28	0.06	0.04
17	Substance Use	0.18	0.14	0.09	0.13	0.15	0.13	0.14	0.01	0.12	0.18	0.20	0.09	0.02	0.04	0.11	0.34	1.00	0.12	0.12	0.03
18	Suicide Risk	0.29	0.29	0.20	0.37	0.54	0.31	0.22	0.24	0.26	0.53	0.49	0.23	0.29	0.21	0.30	0.09	0.19	1.00	0.37	0.34
19	Trauma Reactions	0.35	0.37	0.22	0.23	0.33	0.37	0.45	0.03	0.23	0.28	0.29	0.46	0.34	0.10	0.57	0.04	0.15	0.20	1.00	0.47
20	Worry	0.48	0.51	0.33	0.46	0.58	0.57	0.41	0.14	0.19	0.57	0.54	0.46	0.44	0.25	0.66	− 0.06	0.14	0.32	0.51	1.00

Results from Site 1 are in the lower diagonal, results from Site 2 are in the upper diagonal

### Discussion

In this study, we tested the second version of the Norse Feedback instrument in two clinical settings. Results demonstrate some areas of strength, including scales covering Substance Use, Sad Affect, Trauma Reactions, and Cognitive Problems, all of which demonstrate good to excellent total information over a wide range of the latent traits, while maintaining a small number of items. Other scales clearly require improvement in subsequent revisions of the instrument. These include Social Safety, Situational Avoidance, and Recovery Environment. Very limited evidence of DIF across clinical sites, gender, and age was observed on all scales. This contrasts with DIF analyses from Study 2, in which the previous NF version demonstrated some meaningful DIF, especially between clinical and nonclinical samples. It remains an open question whether DIF for this version of the NF between a clinical and nonclinical sample would be substantial or negligible.

While we did not conduct inferential statistics or dimension reduction on the large correlation matrices between scale scores, some patterns are worth noting. First, most correlations are positive, medium effects (note that higher scores on resource scales indicate less resource). This accords with overall positive associations between psychosocial problems of different types. Few correlations are above *r* = 0.6, which indicates that these scale scores are not redundant. Nevertheless, an optimal measurement system would take advantage of these correlations, which may reduce the test length.

## General discussion

This manuscript reports the initial development, implementation, and initial reliability and validity of Norse Feedback. The NF is a novel clinical feedback system developed to incorporate patient and clinician stakeholder feedback. The initial structural validity findings presented here do not represent a final instrument, because the NF is intended to be revised iteratively and indefinitely. Rather, the goal is to generate a tool that has clinical use now, and improve the psychometric functioning through revision. The data presented here suggest that many of the scale scores of the NF have structural validity as demonstrated by unidimensionality and acceptable scale information, while other scales provide little value as sum scores. Those scales may be best interpreted as a group of individual items until new items are generated to improve their validity.

Throughout this process, we have worked to improve the experience of clinicians and patients using the NF. A variety of scales demonstrate acceptable information at common ranges, the most recent version of the NF demonstrates small to negligible DIF across sites, gender, and age groups, and initial validity analyses in Study 2 largely conform to expectations regarding discrimination between clinical and nonclinical populations. We believe that with greater evidence of criterion validity and appropriate temporal features, the NF could be a promising measure for incorporation into routine mental healthcare settings. Development of the NF on the basis of these findings is ongoing. The development process has brought both psychometric and clinical user experiences together, so that future versions of the NF will address concerns raised in this manuscript and in interviews with clinicians and patients using the system.

## Limitations and future directions

These studies have several limitations. The most obvious relate to the limited criterion validity presented here. While we have elected to focus efforts of structure and internal consistency at present, establishing validity of the NF scales through correlations with other outcomes is an important next step. Currently, the NF scores rely on face-valid interpretation by clinicians and patients, which is not ideal. Another major area for future research is the validity of these scales for the assessment of individual change over time. Further analyses, possibly including measurement invariance testing over time and analysis of sensitivity to change, should be conducted prior to concluding that change scores from these measures are valid for use as outcomes in applied settings. In the meantime, scale scores are best interpreted as indicators of between-person severity differences. When interpreting changes over time, clinicians should evaluate whether any changes on scale scores reflect clinically meaningful improvement or deterioration, and consider other clinical information prior to concluding that score changes (or lack thereof) represent meaningful differences (or lack thereof). Finally, a significant limitation of these studies is that we have not examined the validity of the measure’s personalized features, especially the adaptive scale selection process that occurs at post-initial administrations, because we have only presented intake clinical and single-administration nonclinical data. The functioning of these adaptation rules is addressed elsewhere [19], and should continue to be the focus of investigation. Again, clinicians should be mindful of their patients’ item responses and carefully explore whether the NF’s adaptation is clinically appropriate using clinical judgment.

Additionally, while multidimensional IRT models have been intentionally left out of the development process to date in order to maintain each scale’s independence, solutions that make use of more of this information may be more efficient. Future research should investigate methods for incorporating multidimensional models while maintaining scale independence and interpretability. Further, most scales of the NF are relatively short and imprecise when compared to longer assessment tools validated in diagnosis. There are limits to short instruments that these scales do not overcome. Nevertheless, by providing an array of several narrow scales, the NF provides an alternative broad assessment, on constructs of interest to patients and clinicians.

Overall, these results comprise the first psychometric evaluation of the Norse Feedback scales. At present, a 20-scale structure has been designed and implemented. This process has been conducted in practice settings and relied on quantitative analyses presented here and qualitative feedback from patients and clinicians. Because the instrument is intended to change over time, the present manuscript is not definitive evidence of validity of the scale scores for particular uses. Future work on the Norse Feedback will address psychometric and clinical issues in these scales through new versions, with as much score interpretability as possible maintained across scale iterations. The measure development process used here, which entail clinical research collaboration, iterative re-evaluation, and quantitative and qualitative analyses, are an example of patient-oriented research to improve clinical efficiency.

## Supplementary Information

Below is the link to the electronic supplementary material.

Supplementary file1 (pdf 13559 kb)

## Footnotes

1 Due to the length of this document, we have also provided more navigable materials at https://osf.io/6xvmf/?view_only=fcbfbb26e65c4c7bbb6e8cede3e975bc

2 Slight numerical differences between figures and tables are due to different implementations between R packages ltm and mirt and do not change interpretation.
