# Supplementary material for: Initial quantitative development of the Norse Feedback system: a novel clinical feedback system for routine mental healthcare
Source: Qual Life Res. 2021 Apr 13;30(11):3097–115. doi: 10.1007/s11136-021-02825-1 (PMC8528796; doi:10.1007/s11136-021-02825-1)
Supplement: Supplementary file 1 — Supplementary file1 (pdf 13559 kb) [file 11136_2021_2825_MOESM1_ESM.pdf]

Supplement to: "Initial quantitative development of the Norse Feedback system: A novel adaptive multidimensional tool for use in routine mental healthcare."

Quality of Life Research

Table of Contents

Supplementary Table S1
DIF by sample (clinical v. nonclinical) in Study 2 2
Supplementary Table S2
DIF by gender in Study 2 6
Supplementary Table S3
Standardized geomin rotated factor loadings from 12-factor EFA, Study 2 10
Supplementary Table S4
DIF by sites in Study 3 13
Supplementary Table S5
DIF by age in Study 3 16
Supplementary Table S6
DIF by gender in Study 3 18
Analysis supplement
Please use HTML version

Supplementary Table S1. DIF by sample (clinical v. nonclinical) in Study 2

			Uniform DIF: χ2 Model 1 v. 2		Non-Uniform DIF: χ2 Model 2 v. 3		Total DIF: χ^2 Model 1 v. 3	
Scale	Item	Brief content	*p*	McFadden's R^2	*p*	McFadden's R^2	*p*	McFadden's R^2
Attachment	41	Form strong connections	<.0001	0.0105	0.0117	0.0013	<.0001	0.0118
Attachment	1	Care for others	0.0179	0.0014	0.0241	0.0013	0.0048	0.0028
Attachment	50	Trusting	<.0001	0.0214	0.0633	0.0007	<.0001	0.0221
Attachment	89	Comfortable w/ emotions	<.0001	0.0049	0.0627	0.0007	<.0001	0.0055
Avoidance	78	Avoid thoughts	<.0001	0.0436	0.0001	0.0034	<.0001	0.0470
Avoidance	8	Avoid places	<.0001	0.0067	0.8427	<.0001	<.0001	0.0068
Avoidance	10	Emotions help me	<.0001	0.0140	0.8806	<.0001	<.0001	0.0140
Avoidance	17	Afraid of things	0.0214	0.0017	0.0004	0.0041	0.0001	0.0058
Avoidance	34	Avoid emotions	<.0001	0.0104	0.0001	0.0031	<.0001	0.0135
Avoidance	64	Avoid people	<.0001	0.0320	0.8568	<.0001	<.0001	0.0320
Connectedness	43	Comfortable w/ friends	<.0001	0.0056	0.0006	0.0025	<.0001	0.0081
Connectedness	45	Sat. w/ sex life	<.0001	0.0106	0.9808	<.0001	<.0001	0.0106
Connectedness	50	Trusting	<.0001	0.0414	0.0019	0.0021	<.0001	0.0435
Connectedness	54	No control	<.0001	0.1151	0.0275	0.0013	<.0001	0.1164
Connectedness	62	Have friends	<.0001	0.0186	0.0012	0.0025	<.0001	0.0211
Connectedness	69	Feel alone	<.0001	0.0723	0.6404	0.0001	<.0001	0.0724
Connectedness	80	Have support	<.0001	0.0210	<.0001	0.0073	<.0001	0.0283
Demoralization	88	Feel trapped	<.0001	0.1453	<.0001	0.0044	<.0001	0.1497
Demoralization	24	Can't handle things	<.0001	0.0626	0.0996	0.0006	<.0001	0.0632
Demoralization	36	Others don't understand	<.0001	0.0335	<.0001	0.0076	<.0001	0.0411
Demoralization	42	Feel depressed	<.0001	0.0984	0.0001	0.0034	<.0001	0.1018
Demoralization	61	No hope	<.0001	0.0239	0.9601	<.0001	<.0001	0.0239
Eating problems	63	Control food	<.0001	0.0187	0.0278	0.0012	<.0001	0.0199
Eating problems	18	Food planning	0.7208	0.0001	<.0001	0.0159	<.0001	0.0160
Eating problems	46	Afraid lose control of eating	<.0001	0.0066	0.0189	0.0017	<.0001	0.0083
Eating problems	47	Digestive problems	<.0001	0.0099	0.0035	0.0019	<.0001	0.0119
Eating problems	57	Eating prevents socializing	0.0001	0.0086	0.7931	<.0001	0.0003	0.0087

Eating problems	86	Body image discomfort	<.0001	0.0179	0.0070	0.0014	<.0001	0.0193
Hypervigilance	2	Spend energy ensuring safety	0.0002	0.0027	0.3568	0.0002	0.0007	0.0029
Hypervigilance	7	Prepared for worst	0.0011	0.0021	0.2490	0.0003	0.0025	0.0023
Hypervigilance	9	Feel safe at home	<.0001	0.0145	0.0001	0.0050	<.0001	0.0195
Hypervigilance	50	Trusting	<.0001	0.0051	0.4669	0.0001	<.0001	0.0052
Pressure from Negative Affect	28	Might cry uncontrollably	<.0001	0.0331	0.0141	0.0015	<.0001	0.0346
Pressure from Negative Affect	21	Anger	<.0001	0.0057	0.8857	<.0001	<.0001	0.0057
Pressure from Negative Affect	30	Health worry	<.0001	0.0045	0.1536	0.0005	<.0001	0.0050
Pressure from Negative Affect	32	Self-harm	<.0001	0.0243	0.0949	0.0026	<.0001	0.0269
Pressure from Negative Affect	33	Sleep well	<.0001	0.0047	0.6517	<.0001	<.0001	0.0048
Pressure from Negative Affect	42	Feel depressed	<.0001	0.1272	0.1544	0.0004	<.0001	0.1277
Pressure from Negative Affect	51	Restlessness	<.0001	0.1173	0.0503	0.0008	<.0001	0.1181
Pressure from Negative Affect	60	Feel down	<.0001	0.0715	0.0151	0.0012	<.0001	0.0727
Pressure from Negative Affect	88	Feel trapped	<.0001	0.1314	0.0069	0.0019	<.0001	0.1333
Perfectionism-Control	20	Worry about carelessness	0.2801	0.0002	0.0180	0.0012	0.0341	0.0014
Perfectionism-Control	26	Doing things right interferes	0.9059	<.0001	0.8102	<.0001	0.9648	<.0001
Perfectionism-Control	28	Might cry uncontrollably	<.0001	0.0193	<.0001	0.0045	<.0001	0.0238
Perfectionism-Control	38	Self-berate	<.0001	0.0100	<.0001	0.0058	<.0001	0.0158
Perfectionism-Control	48	Order compulsively	0.0179	0.0022	0.9653	<.0001	0.0606	0.0022
Perfectionism-Control	52	Need control	0.0406	0.0008	0.0954	0.0006	0.0306	0.0014
Perfectionism-Control	68	Don't let others control	0.0436	0.0008	0.5156	0.0001	0.1056	0.0009
Relational distress	81	Relationships cause stress	<.0001	0.1177	0.0014	0.0022	<.0001	0.1199
Relational distress	25	Affected by others' opinions	<.0001	0.0183	0.0714	0.0006	<.0001	0.0190
Relational distress	36	Others don't understand	<.0001	0.0643	0.3496	0.0003	<.0001	0.0646
Relational distress	37	Annoyed by others	<.0001	0.0089	0.0203	0.0011	<.0001	0.0101

Relational distress	65	Many conflicts	<.0001	0.0237	0.0070	0.0024	<.0001	0.0262
Relational distress	66	Others cause conflicts	<.0001	0.0209	<.0001	0.0106	<.0001	0.0315
Relational distress	69	Have friends	<.0001	0.0558	0.6452	<.0001	<.0001	0.0558
Resilience and personal coping	27	Feel productive	<.0001	0.0074	0.7361	<.0001	<.0001	0.0075
Resilience and personal coping	6	Enjoy job/school	<.0001	0.0128	0.1685	0.0006	<.0001	0.0134
Resilience and personal coping	15	Hope	<.0001	0.0124	<.0001	0.0127	<.0001	0.0251
Resilience and personal coping	22	Can relax	<.0001	0.0197	0.6677	<.0001	<.0001	0.0197
Resilience and personal coping	33	Sleep well	0.0274	0.0012	0.7617	<.0001	0.0838	0.0012
Resilience and personal coping	40	Assertive	0.9819	<.0001	0.0198	0.0014	0.0661	0.0014
Resilience and personal coping	55	Do things for pleasure	0.0031	0.0021	<.0001	0.0041	<.0001	0.0063
Resilience and personal coping	56	Exercise is good	0.0179	0.0015	0.9940	<.0001	0.0607	0.0015
Resilience and personal coping	67	Like self	<.0001	0.0070	0.5486	0.0001	<.0001	0.0071
Resilience and personal coping	70	Not comfortable with support	<.0001	0.0140	0.0735	0.0008	<.0001	0.0147
Resilience and personal coping	84	Active for health	0.0310	0.0012	0.5760	0.0001	0.0835	0.0013
Resilience and personal coping	89	Comfortable w/ emotions	0.0652	0.0009	0.6070	0.0001	0.1601	0.0009
Hurtful Rumination	39	Uncontrollable worries	0.4392	0.0001	0.7104	<.0001	0.6920	0.0001
Hurtful Rumination	3	Physical tension	0.4665	0.0001	0.0877	0.0006	0.1786	0.0007
Hurtful Rumination	38	Self-berate	<.0001	0.0103	0.6195	0.0001	<.0001	0.0103
Hurtful Rumination	76	Worthlessness	0.3878	0.0002	0.6394	0.0001	0.6171	0.0003
Hurtful Rumination	85	Need to ruminate less	0.3260	0.0002	0.0631	0.0007	0.1098	0.0009
Social Role Functioning	87	Important to community	<.0001	0.0059	0.5205	0.0001	<.0001	0.0060
Social Role Functioning	5	Value in community	<.0001	0.0046	0.3929	0.0002	0.0001	0.0048
Social Role Functioning	22	Can relax	<.0001	0.0072	0.2994	0.0002	<.0001	0.0074
Social Role Functioning	27	Feel productive	<.0001	0.0039	0.7270	<.0001	0.0002	0.0039
Somatic Anxiety	3	Physical tension	<.0001	0.0688	0.5280	0.0001	<.0001	0.0689
Somatic Anxiety	30	Health worry	<.0001	0.0201	0.4738	0.0001	<.0001	0.0202

Somatic Anxiety	47	Digestive problems	<.0001	0.0103	0.6598	<.0001	<.0001	0.0103
Somatic Anxiety	51	Restlessness	<.0001	0.2311	0.0021	0.0019	<.0001	0.2330
Somatic Anxiety	53	Physical arousal	<.0001	0.1363	<.0001	0.0047	<.0001	0.1410
Somatic Anxiety	75	Fear without reason	<.0001	0.2190	0.6639	<.0001	<.0001	0.2190
Substance Use	35	Concerned dependent	0.0002	0.0089	0.0079	0.0044	<.0001	0.0133
Substance Use	4	Use interferes	0.0024	0.0066	0.0106	0.0047	0.0004	0.0113
Substance Use	16	Others mentioned use	0.0043	0.0062	0.5304	0.0003	0.0140	0.0065
Substance Use	59	Think should reduce use	0.0045	0.0041	0.2518	0.0007	0.0092	0.0047
Suicide risk	44	Thoughts of suicide	0.1851	0.0006	0.1666	0.0006	0.1597	0.0012
Suicide risk	19	Better if dead	0.0384	0.0016	0.1734	0.0007	0.0464	0.0023
Suicide risk	77	Scared of impulsive suicide	0.4118	0.0003	0.6103	0.0001	0.6271	0.0005
Suicide risk	79	Plans for suicide	0.0529	0.0025	0.3444	0.0006	0.0982	0.0030

Note. Rows where McFadden's $R^2 < .02$ are presented in gray text for clarity.

Supplementary Table S2. DIF by gender in Study 2

		Uniform DIF: χ^2 Model 1 v. 2		Total DIF: χ^2 Model 1 v. 3		Non-Uniform DIF: χ^2 Model 2 v. 3	
Scale	Item	p	McFadden's R^2	p	McFadden's R^2	p	McFadden's R^2
Attachment	41	0.041	0.0008	0.114	0.0009	0.695	<.0001
Attachment	1	<.001	0.0178	<.001	0.0178	0.884	<.0001
Attachment	50	0.070	0.0006	<.001	0.0081	<.001	0.0075
Attachment	89	0.590	0.0001	0.736	0.0001	0.569	0.0001
Avoidance	78	0.535	0.0001	0.821	0.0001	0.920	<.0001
Avoidance	8	0.234	0.0003	0.439	0.0004	0.628	0.0001
Avoidance	10	0.072	0.0007	0.132	0.0009	0.366	0.0002
Avoidance	17	0.004	0.0027	0.016	0.0027	0.939	<.0001
Avoidance	34	0.031	0.0010	<.001	0.0032	0.001	0.0022
Avoidance	64	0.000	0.0034	0.000	0.0043	0.064	0.0008
Connectedness	43	0.048	0.0008	0.064	0.0012	0.204	0.0003
Connectedness	45	0.000	0.0030	<.001	0.0053	0.001	0.0023
Connectedness	50	0.085	0.0006	0.077	0.0011	0.143	0.0005
Connectedness	54	0.240	0.0004	0.296	0.0007	0.304	0.0003
Connectedness	62	<.001	0.0072	<.001	0.0082	0.045	0.0010
Connectedness	69	0.621	0.0001	0.871	0.0001	0.858	<.0001
Connectedness	80	<.001	0.0052	<.001	0.0052	0.763	<.0001
Demoralization	88	0.857	<.0001	0.963	<.0001	0.834	<.0001
Demoralization	24	<.001	0.0077	<.001	0.0077	0.758	<.0001
Demoralization	36	0.888	<.0001	0.792	0.0001	0.504	0.0001
Demoralization	42	0.345	0.0002	0.585	0.0002	0.670	<.0001
Demoralization	61	0.056	0.0010	0.101	0.0013	0.332	0.0003
Eating problems	63	0.135	0.0005	0.265	0.0007	0.516	0.0001
Eating problems	18	0.018	0.0024	0.041	0.0027	0.370	0.0003

Eating problems	46	0.983	<.0001	0.940	<.0001	0.724	<.0001
Eating problems	47	0.733	<.0001	0.757	0.0001	0.508	0.0001
Eating problems	57	0.002	0.0054	<.001	0.0094	0.009	0.0040
Eating problems	86	0.001	0.0022	<.001	0.0039	0.003	0.0017
Hypervigilance	2	0.244	0.0003	0.085	0.0010	0.059	0.0007
Hypervigilance	7	0.635	<.0001	0.548	0.0002	0.322	0.0002
Hypervigilance	9	0.018	0.0018	0.053	0.0019	0.573	0.0001
Hypervigilance	50	0.613	<.0001	0.038	0.0013	0.012	0.0012
Pressure from Negative Affect	28	<.001	0.0151	<.001	0.0152	0.493	0.0001
Pressure from Negative Affect	21	0.805	<.0001	0.792	0.0001	0.524	0.0001
Pressure from Negative Affect	30	0.608	0.0001	0.845	0.0001	0.784	<.0001
Pressure from Negative Affect	32	0.714	0.0001	0.610	0.0009	0.356	0.0008
Pressure from Negative Affect	33	0.163	0.0004	0.194	0.0006	0.248	0.0003
Pressure from Negative Affect	42	0.740	<.0001	0.859	0.0001	0.659	<.0001
Pressure from Negative Affect	51	0.682	<.0001	0.148	0.0008	0.056	0.0008
Pressure from Negative Affect	60	0.771	<.0001	0.414	0.0004	0.195	0.0003
Pressure from Negative Affect	88	0.280	0.0003	0.358	0.0005	0.347	0.0002
Perfectionism-Control	20	0.177	0.0004	0.395	0.0004	0.849	<.0001
Perfectionism-Control	26	<.001	0.0040	<.001	0.0041	0.640	<.0001
Perfectionism-Control	28	<.001	0.0094	<.001	0.0098	0.208	0.0004
Perfectionism-Control	38	0.588	0.0001	0.282	0.0006	0.134	0.0005
Perfectionism-Control	48	0.032	0.0018	0.079	0.0020	0.479	0.0002

Perfectionism-Control	52	0.150	0.0004	0.199	0.0006	0.281	0.0002
Perfectionism-Control	68	0.031	0.0009	0.097	0.0009	0.946	<.0001
Relational distress	81	0.371	0.0002	0.665	0.0002	0.898	<.0001
Relational distress	25	<.001	0.0035	<.001	0.0039	0.150	0.0004
Relational distress	36	0.950	<.0001	0.997	<.0001	0.962	<.0001
Relational distress	37	0.284	0.0002	0.558	0.0002	0.889	<.0001
Relational distress	65	0.257	0.0004	0.494	0.0004	0.727	<.0001
Relational distress	66	<.001	0.0085	<.001	0.0086	0.564	0.0001
Relational distress	69	0.805	<.0001	0.086	0.0011	0.028	0.0010
Resilience and personal coping	27	0.444	0.0001	0.675	0.0002	0.654	0.0001
Resilience and personal coping	6	<.001	0.0050	<.001	0.0052	0.456	0.0002
Resilience and personal coping	15	0.014	0.0015	0.036	0.0017	0.444	0.0001
Resilience and personal coping	22	0.132	0.0006	0.113	0.0011	0.147	0.0005
Resilience and personal coping	33	0.351	0.0002	0.455	0.0004	0.401	0.0002
Resilience and personal coping	40	0.325	0.0002	0.575	0.0003	0.713	<.0001
Resilience and personal coping	55	0.057	0.0009	0.001	0.0036	0.001	0.0027
Resilience and personal coping	56	0.834	<.0001	0.006	0.0027	0.001	0.0027
Resilience and personal coping	67	0.570	0.0001	0.568	0.0003	0.369	0.0002
Resilience and personal coping	70	0.071	0.0008	0.190	0.0008	0.809	<.0001
Resilience and personal coping	84	0.026	0.0013	0.030	0.0018	0.153	0.0005

Resilience and personal coping	89	0.944	<.0001	0.988	<.0001	0.887	<.0001
Hurtful Rumination	39	0.652	<.0001	0.600	0.0002	0.366	0.0002
Hurtful Rumination	3	<.001	0.0050	<.001	0.0050	0.739	<.0001
Hurtful Rumination	38	0.297	0.0002	0.195	0.0007	0.139	0.0005
Hurtful Rumination	76	0.612	0.0001	0.447	0.0005	0.244	0.0004
Hurtful Rumination	85	0.000	0.0025	0.001	0.0027	0.323	0.0002
Social Role Functioning	87	0.176	0.0005	0.391	0.0005	0.841	<.0001
Social Role Functioning	5	0.993	<.0001	0.912	<.0001	0.668	<.0001
Social Role Functioning	22	0.023	0.0012	0.025	0.0017	0.136	0.0005
Social Role Functioning	27	0.455	0.0001	0.708	0.0002	0.717	<.0001
Somatic Anxiety	3	<.001	0.0057	<.001	0.0059	0.285	0.0002
Somatic Anxiety	30	0.292	0.0002	0.544	0.0003	0.740	<.0001
Somatic Anxiety	47	0.036	0.0010	0.054	0.0013	0.232	0.0003
Somatic Anxiety	51	<.001	0.0039	<.001	0.0051	0.018	0.0011
Somatic Anxiety	53	0.885	<.0001	0.557	0.0003	0.284	0.0003
Somatic Anxiety	75	0.685	<.0001	0.713	0.0001	0.474	0.0001
Substance Use	35	0.066	0.0018	0.184	0.0018	0.958	<.0001
Substance Use	4	0.481	0.0004	0.753	0.0004	0.790	0.0001
Substance Use	16	0.044	0.0028	0.087	0.0034	0.363	0.0006
Substance Use	59	0.474	0.0003	0.682	0.0004	0.616	0.0001
Suicide risk	44	0.122	0.0008	0.079	0.0016	0.102	0.0009
Suicide risk	19	0.042	0.0015	0.087	0.0017	0.389	0.0003
Suicide risk	77	0.769	<.0001	0.894	0.0001	0.711	0.0001
Suicide risk	79	0.391	0.0004	0.689	0.0004	0.927	<.0001

Note. Rows where McFadden's $R^2 < .02$ are presented in gray text for clarity. That is all rows in this analysis.

Supplemental Table S3. Standardized geomin rotated factor loadings from 12-factor EFA, Study 2.

Item	Assigned Scale	1	2	3	4	5	6	7	8	9	10	11	12
20	Perfectionism-Control	**0.77**	0.13	-0.02	0.10	-0.03	0.05	-0.08	-0.04	0.09	-0.08	0.04	-0.01
23	Emotional Distancing	**0.77**	0.11	0.04	0.00	0.11	-0.06	-0.16	-0.02	0.08	-0.04	-0.01	0.02
39	Hurtful Rumination	**0.74**	-0.23	0.10	-0.05	0.02	-0.10	0.23	0.01	-0.05	0.02	-0.05	-0.11
3	Hurtful Rumination	**0.69**	0.11	-0.08	-0.06	-0.04	0.08	-0.03	-0.02	0.00	-0.03	0.03	-0.39
2	Hypervigilance	**0.66**	0.18	-0.05	0.00	-0.06	0.03	-0.01	0.21	0.27	-0.03	0.06	0.07
51	Pressure from Negative Affect	**0.65**	-0.30	0.01	0.00	0.07	-0.03	0.27	0.00	-0.03	0.03	-0.06	-0.17
78	Avoidance	**0.61**	-0.12	0.01	0.07	0.01	0.09	-0.02	-0.03	-0.01	-0.01	-0.05	0.10
31	Emotional Distancing	**0.60**	-0.09	0.06	0.04	0.04	-0.04	-0.31	0.21	-0.01	-0.19	0.12	0.12
7	Hypervigilance	**0.56**	0.09	-0.08	-0.02	0.01	0.21	-0.07	0.01	0.14	0.08	0.15	-0.02
81	Relational distress	**0.51**	-0.06	-0.07	-0.10	0.02	-0.25	0.03	0.02	0.27	0.31	-0.02	0.09
8	Avoidance	**0.47**	-0.03	0.02	-0.01	-0.04	-0.07	-0.02	0.15	0.25	0.08	-0.19	0.22
34	Avoidance	**0.45**	-0.01	0.03	0.01	0.00	0.21	0.02	-0.04	0.31	0.21	0.02	-0.03
58	Psychosis	**0.44**	0.24	0.09	0.15	0.05	0.01	-0.31	0.16	-0.10	0.01	-0.14	-0.01
75	Somatic Anxiety	**0.42**	-0.27	0.08	0.01	-0.06	0.10	0.17	0.21	0.14	-0.01	-0.09	-0.10
85	Hurtful Rumination	**0.40**	0.04	-0.18	0.08	-0.06	-0.01	0.22	0.09	0.11	-0.01	-0.23	-0.21
88	Demoralization	0.39	-0.18	-0.01	0.06	0.25	-0.15	0.25	0.05	0.02	0.09	-0.04	-0.27
64	Avoidance	0.37	-0.25	0.03	-0.07	0.07	-0.12	-0.02	0.05	0.31	0.27	0.03	0.22
25	Relational distress	0.37	-0.04	-0.01	0.21	0.03	0.22	0.12	0.05	-0.10	0.12	-0.28	0.18
53	Somatic Anxiety	0.37	-0.01	0.03	-0.10	0.09	-0.05	0.05	0.32	0.35	-0.02	0.03	-0.34
38	Hurtful Rumination	0.35	-0.20	-0.03	0.25	0.07	0.05	-0.03	0.02	-0.03	0.25	-0.07	0.07
54	Connectedness	0.31	-0.21	0.04	0.04	0.17	0.14	-0.03	0.26	0.19	0.00	0.08	-0.12
6	Resilience and personal coping	-0.12	**0.79**	0.16	0.10	0.17	0.11	0.22	-0.04	-0.03	0.03	0.01	-0.03
27	Resilience and personal coping	0.01	**0.70**	0.13	-0.01	0.01	-0.06	0.30	-0.11	-0.07	0.06	-0.10	-0.01
15	Resilience and personal coping	0.14	**0.53**	0.16	-0.09	-0.19	0.16	0.07	0.05	0.01	-0.04	0.10	0.13
42	Demoralization	0.36	**-0.52**	-0.02	-0.08	0.27	0.04	0.21	-0.01	-0.12	0.08	0.01	-0.08
60	Pressure from Negative Affect	-0.25	**0.49**	-0.04	0.09	-0.20	-0.08	0.02	0.15	0.00	0.00	0.17	0.12
69	Connectedness	0.33	-0.35	0.06	0.07	0.10	0.06	-0.01	-0.08	0.14	0.19	-0.06	-0.01
62	Connectedness	0.07	0.33	-0.01	-0.03	0.00	0.10	-0.02	0.03	-0.12	-0.24	0.23	-0.06
59	Substance Use	-0.03	0.00	**0.96**	0.01	-0.09	-0.03	-0.01	-0.07	0.23	0.02	-0.03	0.01
35	Substance Use	0.16	0.04	**0.95**	-0.01	0.00	-0.08	0.01	0.06	-0.01	-0.02	-0.12	-0.06
4	Substance Use	0.02	0.00	**0.95**	0.06	0.11	0.03	0.02	0.02	-0.05	-0.03	-0.01	0.00
16	Substance Use	0.02	-0.01	**0.91**	-0.03	0.02	0.02	-0.08	0.01	0.21	0.02	0.07	0.03
18	Eating problems	0.03	0.08	0.07	**0.97**	-0.02	-0.03	-0.07	-0.04	0.03	0.02	0.02	-0.06
46	Eating problems	0.04	0.00	0.00	**0.86**	-0.10	0.15	-0.15	0.01	-0.03	0.05	-0.04	-0.07
57	Eating problems	-0.18	-0.06	0.05	**0.85**	0.22	-0.11	-0.02	0.09	0.03	-0.09	-0.05	0.00
63	Eating problems	0.00	0.04	-0.01	**0.82**	0.05	0.02	0.03	0.00	-0.01	0.15	0.24	-0.01
86	Eating problems	0.04	0.04	-0.18	0.37	0.21	0.30	0.03	0.06	0.19	-0.06	-0.25	0.00
26	Perfectionism-Control	0.29	-0.22	-0.06	0.32	0.00	0.00	0.07	0.16	-0.04	-0.01	0.06	0.08
44	Suicide risk	0.01	0.02	0.07	-0.07	**0.90**	0.03	0.13	-0.05	0.07	-0.02	0.04	0.01
79	Suicide risk	-0.01	0.03	-0.11	-0.10	**0.84**	-0.11	-0.32	0.10	-0.02	-0.02	0.01	-0.04

19	Suicide risk	0.08	-0.05	0.00	0.02	**0.84**	0.02	-0.05	-0.17	-0.05	0.12	-0.01	0.01
77	Suicide risk	-0.05	-0.03	0.07	-0.10	**0.78**	0.02	-0.14	0.01	0.02	0.10	-0.04	0.00
76	Hurtful Rumination	0.19	-0.28	-0.07	0.13	**0.60**	0.04	-0.03	-0.01	0.01	-0.01	-0.05	0.11
61	Demoralization	0.09	**-0.45**	0.07	0.08	**0.52**	-0.08	-0.01	0.00	0.03	-0.01	0.09	-0.02
32	Pressure from Negative Affect	-0.03	-0.02	-0.03	0.20	**0.49**	0.02	-0.20	0.22	0.00	0.07	-0.08	-0.02
24	Demoralization	0.17	-0.29	0.01	-0.02	**0.40**	0.14	0.09	0.07	-0.01	0.09	-0.03	-0.27
49	Psychosis	0.13	0.04	0.04	0.08	0.32	-0.27	-0.15	-0.01	0.02	0.26	0.14	0.03
1	Attachment	0.02	0.02	-0.04	-0.08	-0.02	**0.57**	0.31	0.01	0.09	-0.14	0.21	0.06
28	Perfectionism-Control	0.37	-0.01	-0.06	0.11	0.10	0.37	0.02	-0.21	-0.05	0.19	-0.12	-0.03
87	Social Role Functioning	-0.05	0.07	0.16	-0.02	-0.31	0.02	**0.46**	0.07	-0.05	-0.12	0.15	-0.01
5	Social Role Functioning	-0.06	0.10	0.25	-0.11	-0.33	0.07	**0.46**	0.07	-0.02	-0.25	0.12	0.01
56	Resilience and personal coping	0.03	-0.05	-0.02	0.38	-0.05	-0.34	**0.42**	-0.05	0.04	0.02	**0.40**	0.01
10	Avoidance	-0.02	0.16	0.01	-0.15	0.05	0.02	0.38	0.07	-0.06	-0.12	0.26	0.12
9	Hypervigilance	-0.01	-0.07	0.00	0.05	-0.16	0.14	0.28	-0.15	0.03	-0.23	0.26	0.07
17	Avoidance	0.10	-0.18	0.07	-0.06	-0.04	0.17	-0.03	**0.59**	0.07	0.08	-0.12	0.14
29	Psychosis	0.03	0.08	0.14	0.01	0.23	0.00	**-0.48**	**0.50**	-0.06	0.07	-0.01	0.04
30	Pressure from Negative Affect	0.09	-0.13	0.00	0.10	-0.09	0.38	-0.02	**0.44**	-0.07	0.03	0.02	-0.20
48	Perfectionism-Control	0.31	-0.01	-0.05	0.17	0.08	-0.12	0.02	**0.41**	-0.10	-0.01	-0.02	0.01
52	Perfectionism-Control	0.12	0.13	-0.17	0.22	0.19	-0.03	0.27	0.38	0.21	-0.05	-0.01	0.04
83	Independent	-0.01	-0.08	-0.01	0.01	-0.15	-0.14	0.29	0.32	-0.07	0.23	-0.05	-0.03
43	Connectedness	0.02	0.16	-0.01	-0.13	0.03	0.02	0.15	0.05	**-0.54**	-0.06	0.28	0.00
50	Attachment	0.04	0.06	0.19	0.06	-0.07	-0.01	0.21	0.05	**-0.47**	-0.29	0.05	0.12
68	Perfectionism-Control	0.02	0.10	-0.14	0.18	-0.09	0.07	0.18	0.20	**0.47**	0.21	-0.02	-0.03
41	Attachment	0.00	0.03	-0.01	0.01	-0.02	**0.41**	0.33	-0.09	**-0.46**	0.06	0.05	0.04
70	Resilience and personal coping	0.14	-0.19	0.00	0.08	0.03	0.18	0.13	-0.10	**0.42**	0.27	0.02	-0.01
65	Relational distress	-0.06	0.00	0.01	0.07	0.00	-0.05	0.01	0.11	-0.03	**0.75**	0.14	-0.08
37	Relational distress	0.07	0.00	-0.02	-0.02	-0.06	0.00	-0.05	0.15	-0.06	**0.63**	-0.18	-0.03
36	Demoralization	0.27	-0.04	0.16	0.16	-0.03	0.12	-0.27	-0.01	0.06	**0.51**	0.04	0.01
66	Relational distress	0.07	0.17	0.02	-0.20	0.11	-0.24	0.03	0.01	0.02	**0.49**	0.03	0.09
21	Pressure from Negative Affect	0.01	0.08	-0.15	-0.01	0.09	0.07	-0.01	0.32	0.04	**0.48**	0.02	-0.14
80	Connectedness	0.21	0.23	-0.03	0.00	-0.12	0.01	0.05	-0.03	-0.01	-0.29	0.29	-0.08
45	Connectedness	0.00	-0.03	0.06	-0.08	-0.13	0.02	-0.19	-0.04	-0.01	0.01	**0.57**	-0.11
40	Resilience and personal coping	-0.04	0.14	-0.09	0.04	-0.01	0.02	-0.04	0.24	-0.26	0.10	**0.53**	0.18
89	Attachment	-0.10	-0.03	-0.02	-0.10	-0.07	0.01	0.08	0.21	-0.23	-0.01	**0.48**	-0.05
55	Resilience and personal coping	-0.04	-0.14	0.13	0.07	0.07	-0.04	-0.04	0.17	0.07	0.00	**0.46**	0.04
84	Resilience and personal coping	0.10	0.17	-0.10	0.23	-0.16	-0.34	0.28	-0.12	-0.03	0.01	**0.41**	0.01

67	Resilience and personal coping	-0.01	0.16	0.10	-0.15	-0.18	-0.02	0.22	0.02	-0.15	0.03	**0.41**	-0.10
33	Pressure from Negative Affect	0.05	0.29	-0.07	-0.07	-0.03	-0.02	0.05	0.02	-0.13	0.02	0.01	**0.63**
22	Resilience and personal coping	-0.35	0.08	0.09	-0.04	0.15	0.01	0.09	0.04	0.00	-0.05	0.39	**0.42**
47	Eating problems	-0.11	-0.02	0.02	0.21	0.06	0.06	0.11	0.18	0.00	0.19	0.04	-0.38
82	Independent	-0.05	0.14	0.13	0.02	0.00	0.04	0.30	0.00	0.15	-0.04	0.00	0.35

Loadings with absolute values between -0.2 and 0.2 are in gray text to increase interpretability. All loadings greater or equal to 0.4 in absolute value are bolded.

Supplementary Table S4. DIF by sites in Study 3.

			Uniform DIF: χ2 Model 1 v. 2		Non-Uniform DIF: χ2 Model 2 v. 3		Total DIF: χ^2 Model 1 v. 3	
Scale	Item	Brief description	*p*	McFadden's R^2	*p*	McFadden's R^2	*p*	McFadden's R^2
Cognitive Problems	142	Difficulty focusing	<.0001	0.0044	0.6215	0.0001	<.0001	0.0045
Cognitive Problems	143	Memory problems	0.3222	0.0002	0.6667	<.0001	0.5584	0.0003
Cognitive Problems	144	Mentally slow	0.0723	0.0007	0.1343	0.0005	0.0648	0.0012
Cognitive Problems	145	Forgetful	0.3204	0.0002	0.0055	0.0017	0.0130	0.0020
Cognitive Problems	146	Worried brain not working	<.0001	0.0043	0.7618	<.0001	0.0001	0.0043
Cognitive Problems	147	Not thinking clearly	<.0001	0.0075	0.3953	0.0002	<.0001	0.0077
Need for Control	26	Doing things right interferes	0.2984	0.0003	0.6219	0.0001	0.5156	0.0003
Need for Control	20	Worry about carelessness	0.3509	0.0002	0.3580	0.0002	0.4242	0.0004
Need for Control	68	Don't let others control	0.9284	<.0001	0.8092	<.0001	0.9673	<.0001
Need for Control	130	Difficult to live with	0.7021	<.0001	0.0736	0.0008	0.1876	0.0008
Eating Problems	46	Afraid lose control of eating	<.0001	0.0109	0.0009	0.0031	<.0001	0.0141
Eating Problems	104	Worry about weight	0.5078	0.0001	0.1627	0.0005	0.3030	0.0006
Eating Problems	57	Eating prevents socializing	0.1095	0.0010	0.1937	0.0007	0.1194	0.0017
Eating Problems	18	Food planning	0.0716	0.0013	0.5407	0.0001	0.1637	0.0014
Eating Problems	63	Control food	0.7031	<.0001	0.6079	0.0001	0.8153	0.0001
Hopelessness	15	Hope	0.7698	<.0001	0.1946	0.0004	0.4132	0.0004
Hopelessness	61	No hope	0.2203	0.0004	0.1592	0.0005	0.1752	0.0008
Hopelessness	115	Things don't get better	0.0004	0.0028	0.6868	<.0001	0.0019	0.0028
Hopelessness	24	Can't handle things	0.8816	<.0001	0.3298	0.0002	0.6152	0.0002
Hopelessness	88	Feel trapped	0.4459	0.0001	0.3642	0.0002	0.4955	0.0003
Internal Avoidance	10	Emotions help me	0.0213	0.0013	0.4836	0.0001	0.0552	0.0014
Internal Avoidance	34	Avoid emotions	0.1876	0.0004	0.5363	0.0001	0.3466	0.0005

Internal Avoidance	78	Avoid thoughts	0.6329	0.0001	0.3066	0.0003	0.5290	0.0003
Internal Avoidance	122	Shut down feelings	0.0579	0.0009	0.6662	<.0001	0.1508	0.0009
Internal Avoidance	123	If start, feelings would overwhelm	0.3790	0.0002	0.8502	<.0001	0.6671	0.0002
Recovery Environment	80	Have support	0.4151	0.0002	0.5842	0.0001	0.6177	0.0003
Recovery Environment	136	Supportive environment	0.0120	0.0016	0.2135	0.0004	0.0197	0.0020
Recovery Environment	138	Satisfied with life	0.6253	0.0001	0.5246	0.0001	0.7250	0.0002
Recovery Environment	139	Have opportunity for pleasure	0.2301	0.0004	0.9922	<.0001	0.4866	0.0004
Recovery Environment	137	Not enough money	0.0329	0.0013	0.1253	0.0007	0.0318	0.0020
Self-Criticism	67	Like self	0.0002	0.0033	0.8643	<.0001	0.0012	0.0033
Self-Criticism	38	Self-berate	0.1691	0.0004	0.4128	0.0002	0.2779	0.0006
Self-Criticism	126	Ashamed of self	0.8852	<.0001	0.6461	0.0001	0.8906	0.0001
Self-Criticism	124	Make stupid mistakes	0.1335	0.0006	0.0721	0.0008	0.0644	0.0013
Self-Criticism	129	I am a bad person deep down	0.6862	<.0001	0.3880	0.0002	0.6350	0.0002
Self-Criticism	127	Unlovable	0.9407	<.0001	0.5265	0.0001	0.8160	0.0001
Self-Criticism	128	Self-disgust	0.4151	0.0002	0.9541	<.0001	0.7162	0.0002
Self-Criticism	101	Totally worthless	0.3064	0.0003	0.1932	0.0004	0.2542	0.0007
Social Safety	43	Comfortable w/ friends	0.1621	0.0005	0.6229	0.0001	0.3334	0.0005
Social Safety	131	Comfortable sharing emotions	0.0769	0.0007	0.7187	<.0001	0.1960	0.0008
Social Safety	40	Assertive	0.4163	0.0002	0.8944	<.0001	0.7124	0.0002
Social Safety	132	Set limits	0.0383	0.0010	0.1844	0.0004	0.0484	0.0014
Social Safety	62	Have friends	0.1211	0.0006	0.3009	0.0002	0.1761	0.0008
Social Safety	50	Trusting	0.0302	0.0011	0.1698	0.0005	0.0372	0.0016
Somatic Anxiety	51	Restlessness	0.0005	0.0028	0.0444	0.0009	0.0003	0.0037
Somatic Anxiety	103	Tense all day	0.0082	0.0016	0.9689	<.0001	0.0302	0.0016
Somatic Anxiety	75	Fear without reason	0.0625	0.0007	0.7404	<.0001	0.1671	0.0008

Somatic Anxiety	3	Physical tension	0.4325	0.0001	0.1429	0.0005	0.2513	0.0006
Somatic Anxiety	53	Physical arousal	0.1934	0.0004	0.4131	0.0001	0.3071	0.0005
Substance Recovery	109	Can handle problems w/o substance	0.0620	0.0055	0.8217	0.0001	0.1709	0.0056
Substance Recovery	154	Can manage a day w/o substance	0.5039	0.0013	0.9732	<.0001	0.7993	0.0013
Substance Recovery	108	Urges manageable	0.1890	0.0033	0.9633	<.0001	0.4216	0.0033
Substance Recovery	155	Substance problems are improving	0.6279	0.0004	0.3318	0.0016	0.5552	0.0020
Substance Use	59	Think should reduce use	0.6697	0.0001	0.4792	0.0002	0.7109	0.0003
Substance Use	4	Use interferes	0.7540	0.0001	0.6263	0.0002	0.8457	0.0002
Substance Use	107	Others worry about Use	0.0006	0.0072	0.4790	0.0003	0.0023	0.0075
Substance Use	35	Concerned dependent	0.6110	0.0002	0.8563	<.0001	0.8643	0.0002
Suicide	19	Better if dead	0.5702	0.0001	0.0188	0.0015	0.0538	0.0016
Suicide	105	Made suicide plans	0.1000	0.0010	0.7703	<.0001	0.2476	0.0010
Suicide	77	Scared of impulsive suicide	0.3612	0.0003	0.8768	<.0001	0.6513	0.0003
Suicide	106	Decided to kill self	0.6705	0.0001	0.2564	0.0008	0.4797	0.0009
Trauma Reaction	111	Overwhelming memories	0.0792	0.0009	0.7529	<.0001	0.2039	0.0009
Trauma Reaction	110	Nightmares	0.5611	0.0001	0.5161	0.0001	0.6840	0.0002
Trauma Reaction	113	Alert to dangers	0.0175	0.0015	0.2923	0.0003	0.0341	0.0018
Trauma Reaction	112	Try to control memories	0.0194	0.0017	0.5577	0.0001	0.0547	0.0018

Note. Rows where McFadden's $R^2 < .02$ are presented in gray text for clarity. That is all rows in this analysis.

Supplementary Table S5. DIF by age in Study 3

		Uniform DIF: $\chi 2$ Model 1 v. 2		Total DIF: $\chi 2$ Model 1 v. 3		Non-Uniform DIF: $\chi 2$ Model 2 v. 3	
Scale	Item	*p*	McFadden's R^2	*p*	McFadden's R^2	*p*	McFadden's R^2
Cognitive Problems	142	<.001	0.006	<.001	0.006	0.727	<.001
Cognitive Problems	143	0.001	0.003	0.002	0.003	0.183	0.000
Cognitive Problems	144	0.272	0.000	0.328	0.001	0.312	0.000
Cognitive Problems	145	0.001	0.002	0.005	0.002	0.748	<.001
Cognitive Problems	146	0.654	<.001	0.128	0.001	0.048	0.001
Cognitive Problems	147	<.001	0.005	<.001	0.006	0.162	0.000
Need for Control	26	0.085	0.001	0.110	0.001	0.230	0.000
Need for Control	20	<.001	0.005	<.001	0.007	0.012	0.002
Need for Control	68	0.691	<.001	0.806	0.000	0.600	0.000
Need for Control	130	0.726	<.001	0.940	<.001	0.969	<.001
Eating Problems	46	0.028	0.001	0.072	0.002	0.500	0.000
Eating Problems	104	0.848	<.001	0.422	0.000	0.194	0.000
Eating Problems	57	0.076	0.001	0.163	0.002	0.487	0.000
Eating Problems	18	0.825	<.001	0.937	0.000	0.776	<.001
Eating Problems	63	0.227	0.000	0.475	0.000	0.867	<.001
Hopelessness	15	0.628	0.000	0.857	0.000	0.785	<.001
Hopelessness	61	0.126	0.001	0.217	0.001	0.399	0.000
Hopelessness	115	0.279	0.000	0.434	0.000	0.479	0.000
Hopelessness	24	0.516	0.000	0.257	0.001	0.130	0.001
Hopelessness	88	0.338	0.000	0.442	0.000	0.397	0.000
Internal Avoidance	10	0.000	0.004	0.000	0.004	0.448	0.000
Internal Avoidance	34	0.379	0.000	0.640	0.000	0.733	<.001
Internal Avoidance	78	0.347	0.000	0.605	0.000	0.731	<.001
Internal Avoidance	122	0.891	<.001	0.887	0.000	0.638	0.000
Internal Avoidance	123	0.077	0.001	0.198	0.001	0.738	<.001
Recovery Environment	80	0.004	0.002	0.010	0.003	0.437	0.000
Recovery Environment	136	0.427	0.000	0.710	0.000	0.820	<.001
Recovery Environment	138	0.000	0.003	<.001	0.006	0.002	0.003
Recovery Environment	139	0.492	0.000	0.731	0.000	0.694	<.001
Recovery Environment	137	0.974	<.001	0.421	0.001	0.189	0.001
Self-Criticism	67	0.989	<.001	0.272	0.001	0.106	0.001
Self-Criticism	38	0.002	0.002	0.002	0.003	0.087	0.001
Self-Criticism	126	0.206	0.000	0.449	0.000	0.981	<.001
Self-Criticism	124	0.300	0.000	0.001	0.003	0.001	0.003

Self-Criticism	129	0.064	0.001	0.145	0.001	0.514	0.000
Self-Criticism	127	0.142	0.001	0.219	0.001	0.347	0.000
Self-Criticism	128	0.020	0.001	0.063	0.001	0.742	<.001
Self-Criticism	101	0.001	0.003	0.004	0.003	0.641	0.000
Social Safety	43	0.821	<.001	0.871	0.000	0.636	0.000
Social Safety	131	0.011	0.002	0.032	0.002	0.501	0.000
Social Safety	40	0.003	0.002	0.010	0.002	0.647	0.000
Social Safety	132	0.309	0.000	0.390	0.000	0.357	0.000
Social Safety	62	0.187	0.000	0.310	0.001	0.437	0.000
Social Safety	50	0.000	0.003	0.001	0.004	0.116	0.001
Somatic Anxiety	51	0.007	0.002	0.000	0.005	0.001	0.003
Somatic Anxiety	103	0.552	0.000	0.790	0.000	0.732	<.001
Somatic Anxiety	75	<.001	0.004	<.001	0.004	0.071	0.001
Somatic Anxiety	3	0.000	0.003	0.000	0.004	0.030	0.001
Somatic Anxiety	53	0.006	0.002	0.018	0.002	0.535	0.000
Substance Recovery	109	0.207	0.003	0.407	0.003	0.648	0.000
Substance Recovery	154	0.482	0.002	0.778	0.002	0.930	<.001
Substance Recovery	108	0.219	0.003	0.454	0.003	0.795	0.000
Substance Recovery	155	0.986	<.001	0.607	0.002	0.318	0.002
Substance Use	59	0.445	0.000	0.712	0.000	0.757	<.001
Substance Use	4	0.504	0.000	0.278	0.002	0.146	0.001
Substance Use	107	0.567	0.000	0.483	0.001	0.289	0.001
Substance Use	35	0.000	0.009	0.001	0.010	0.133	0.002
Suicide	19	0.774	<.001	0.498	0.000	0.252	0.000
Suicide	105	0.181	0.001	0.247	0.001	0.315	0.000
Suicide	77	0.742	<.001	0.916	0.000	0.795	<.001
Suicide	106	0.000	0.010	0.000	0.012	0.071	0.002
Trauma Reaction	111	0.442	0.000	0.408	0.001	0.273	0.000
Trauma Reaction	110	0.059	0.001	0.168	0.001	0.912	<.001
Trauma Reaction	113	0.289	0.000	0.522	0.000	0.674	<.001
Trauma Reaction	112	0.108	0.001	0.262	0.001	0.762	<.001

Note. Rows where McFadden's $R^2 < .02$ are presented in gray text for clarity. That is all rows in this analysis.

Supplementary Table S6. DIF by gender in Study 3.

		Uniform DIF: χ2 Model 1 v. 2		Total DIF: χ2 Model 1 v. 3		Non-Uniform DIF: χ2 Model 2 v. 3	
Scale	Item	*p*	McFadden's R^2	*p*	McFadden's R^2	*p*	McFadden's R^2
Cognitive Problems	142	0.375	0.000	0.150	0.001	0.083	0.001
Cognitive Problems	143	0.205	0.000	0.212	0.001	0.222	0.000
Cognitive Problems	144	0.234	0.000	0.315	0.001	0.346	0.000
Cognitive Problems	145	0.202	0.000	0.207	0.001	0.217	0.000
Cognitive Problems	146	0.010	0.002	0.034	0.002	0.752	<.001
Cognitive Problems	147	0.005	0.002	0.016	0.002	0.466	0.000
Need for Control	26	0.527	0.000	0.591	0.000	0.419	0.000
Need for Control	20	0.342	0.000	0.231	0.001	0.154	0.001
Need for Control	68	<.001	0.009	<.001	0.010	0.070	0.001
Need for Control	130	0.000	0.004	0.001	0.004	0.920	<.001
Eating Problems	46	<.001	0.014	<.001	0.015	0.122	0.001
Eating Problems	104	<.001	0.021	<.001	0.021	0.371	0.000
Eating Problems	57	0.338	0.000	0.319	0.001	0.242	0.001
Eating Problems	18	<.001	0.013	<.001	0.013	0.285	0.001
Eating Problems	63	0.210	0.000	0.449	0.000	0.865	<.001
Hopelessness	15	0.109	0.001	0.179	0.001	0.350	0.000
Hopelessness	61	<.001	0.005	<.001	0.005	0.470	0.000
Hopelessness	115	0.428	0.000	0.373	0.000	0.246	0.000
Hopelessness	24	0.104	0.001	0.263	0.001	0.887	<.001
Hopelessness	88	0.615	0.000	0.833	0.000	0.738	<.001
Internal Avoidance	10	0.733	<.001	0.854	0.000	0.656	<.001
Internal Avoidance	34	0.940	<.001	0.990	<.001	0.904	<.001
Internal Avoidance	78	0.427	0.000	0.652	0.000	0.636	0.000
Internal Avoidance	122	<.001	0.007	<.001	0.007	0.968	<.001
Internal Avoidance	123	0.000	0.003	0.001	0.003	0.961	<.001
Recovery Environment	80	0.735	<.001	0.720	0.000	0.461	0.000
Recovery Environment	136	0.771	<.001	0.253	0.001	0.103	0.001
Recovery Environment	138	<.001	0.005	<.001	0.008	0.002	0.002
Recovery Environment	139	0.000	0.004	0.001	0.004	0.777	<.001
Recovery Environment	137	0.703	<.001	0.927	<.001	0.945	<.001
Self-Criticism	67	0.296	0.000	0.287	0.001	0.236	0.000
Self-Criticism	38	0.290	0.000	0.572	0.000	0.977	<.001
Self-Criticism	126	0.013	0.002	0.044	0.002	0.830	<.001
Self-Criticism	124	0.449	0.000	0.650	0.000	0.591	0.000
Self-Criticism	129	0.723	<.001	0.346	0.001	0.158	0.001

Self-Criticism	127	0.241	0.000	0.445	0.000	0.622	0.000
Self-Criticism	128	0.010	0.002	0.037	0.002	0.842	<.001
Self-Criticism	101	0.015	0.002	0.017	0.002	0.126	0.001
Social Safety	43	0.659	<.001	0.841	0.000	0.699	<.001
Social Safety	131	<.001	0.005	<.001	0.005	0.918	<.001
Social Safety	40	0.120	0.001	0.279	0.001	0.719	<.001
Social Safety	132	0.004	0.002	0.014	0.002	0.741	<.001
Social Safety	62	0.001	0.003	<.001	0.005	0.003	0.002
Social Safety	50	<.001	0.008	<.001	0.008	0.981	<.001
Somatic Anxiety	51	0.873	<.001	0.980	<.001	0.899	<.001
Somatic Anxiety	103	<.001	0.008	<.001	0.009	0.045	0.001
Somatic Anxiety	75	0.112	0.001	0.041	0.001	0.050	0.001
Somatic Anxiety	3	<.001	0.010	<.001	0.014	<.001	0.004
Somatic Anxiety	53	0.006	0.002	<.001	0.006	<.001	0.004
Substance Recovery	109	0.760	0.000	0.949	0.000	0.912	<.001
Substance Recovery	154	0.429	0.002	0.602	0.003	0.532	0.001
Substance Recovery	108	0.912	<.001	0.732	0.001	0.434	0.001
Substance Recovery	155	0.610	0.000	0.872	0.000	0.911	<.001
Substance Use	59	0.026	0.002	0.082	0.002	0.926	<.001
Substance Use	4	0.984	<.001	0.692	0.001	0.391	0.001
Substance Use	107	0.167	0.001	0.135	0.003	0.148	0.001
Substance Use	35	0.839	<.001	0.979	<.001	0.986	<.001
Suicide	19	0.586	0.000	0.437	0.000	0.244	0.000
Suicide	105	0.005	0.003	<.001	0.009	0.000	0.006
Suicide	77	0.874	<.001	0.552	0.000	0.281	0.000
Suicide	106	0.450	0.000	0.150	0.002	0.072	0.002
Trauma Reaction	111	0.340	0.000	0.617	0.000	0.816	<.001
Trauma Reaction	110	0.199	0.001	0.294	0.001	0.371	0.000
Trauma Reaction	113	0.156	0.001	0.366	0.001	0.984	<.001
Trauma Reaction	112	0.001	0.004	0.001	0.004	0.207	0.001

Note. Rows where McFadden's $R^2 < .02$ are presented in gray text for clarity.

Analysis results supplement to 'Initial quantitative development of the Norse Feedback system A novel clinical feedback system for routine mental healthcare'

Andrew A. McAleavey, Samuel S. Nordberg, & Christian Moltu

November 19, 2020

Contents

Summary **5**

Preliminary **6**

Functions . . . 6
Reading data . . . 6

Study 2: Scale performance **7**

Attachment . . . 7
Reliability: Attachment . . . 7
Unidimensionality: Attachment . . . 7
Graded-Response Model: Attachment . . . 8
Gender-based DIF: Attachment . . . 9
Sample-based DIF: Attachment . . . 12
Avoidance . . . 13
Reliability: Avoidance . . . 13
Unidimensionality: Avoidance . . . 14
Graded-Response Model: Avoidance . . . 15
Gender-based DIF: Avoidance . . . 16
Sample-based DIF: Avoidance . . . 19
Connectedness . . . 20
Reliability: Connectedness . . . 20
Unidimensionality: Connectedness . . . 21
Graded-Response Model: Connectedness . . . 22
Gender-based DIF: Connectedness . . . 23
Sample-based DIF: Connectedness . . . 26
Demoralization . . . 27
Reliability: Demoralization . . . 27
Unidimensionality: Demoralization . . . 28
Graded-Response Model: Demoralization . . . 29
Gender-based DIF: Demoralization . . . 30
Sample-based DIF: Demoralization . . . 32
Eating Problems . . . 33
Reliability: Eating Problems . . . 33
Unidimensionality: Eating Problems . . . 34
Graded-Response Model: Eating Problems . . . 35
Gender-based DIF: Eating Problems . . . 36
Sample-based DIF: Eating Problems . . . 39

Hypervigilance . . . 40
Reliability: Hypervigilance . . . 40
Unidimensionality: Hypervigilance . . . 40
Graded-Response Model: Hypervigilance . . . 41
Gender-based DIF: Hypervigilance . . . 42
Sample-based DIF: Hypervigilance . . . 43
Pressure from Negative Affect . . . 43
Reliability: Pressure from Negative Affect . . . 43
Unidimensionality: Pressure from Negative Affect . . . 44
Graded-Response Model: Pressure from Negative Affect . . . 45
Gender-based DIF: Pressure from Negative Affect . . . 46
Sample-based DIF: Pressure from Negative Affect . . . 49
Perfectionism . . . 50
Reliability: Perfectionism . . . 50
Unidimensionality: Perfectionism . . . 50
Graded-Response Model: Perfectionism . . . 51
Gender-based DIF: Perfectionism . . . 52
Sample-based DIF: Perfectionism . . . 55
Psychosis . . . 58
Reliability: Psychosis . . . 58
Unidimensionality: Psychosis . . . 59
Graded-Response Model: Psychosis . . . 60
Gender-based DIF: Psychosis . . . 61
Sample-based DIF: Psychosis . . . 61
Relational Distress . . . 61
Reliability: Relational Distress . . . 61
Unidimensionality: Relational Distress . . . 62
Graded-Response Model: Relational Distress . . . 63
Gender-based DIF: Relational Distress . . . 64
Sample-based DIF: Relational Distress . . . 67
Resilience . . . 68
Reliability: Resilience . . . 68
Unidimensionality: Resilience . . . 68
Graded-Response Model: Resilience . . . 69
Gender-based DIF: Resilience . . . 71
Sample-based DIF: Resilience . . . 74
Hurtful Rumination . . . 79
Reliability: Hurtful Rumination . . . 79
Unidimensionality: Hurtful Rumination . . . 80
Graded-Response Model: Hurtful Rumination . . . 81
Gender-based DIF: Hurtful Rumination . . . 82
Sample-based DIF: Hurtful Rumination . . . 85
Social Role Functioning . . . 87
Reliability: Social Role Functioning . . . 87
Unidimensionality: Social Role Functioning . . . 88
Graded-Response Model: Social Role Functioning . . . 89
Gender-based DIF: Social Role Functioning . . . 90
Sample-based DIF: Social Role Functioning . . . 90
Somatic Anxiety . . . 91
Reliability: Somatic Anxiety . . . 91
Unidimensionality: Somatic Anxiety . . . 92
Graded-Response Model: Somatic Anxiety . . . 93
Gender-based DIF: Somatic Anxiety . . . 94
Sample-based DIF: Somatic Anxiety . . . 97

Substance Use . . . 98
Reliability: Substance Use . . . 98
Unidimensionality: Substance Use . . . 98
Graded-Response Model: Substance Use . . . 99
Gender-based DIF: Substance Use . . . 100
Sample-based DIF: Substance Use . . . 101
Suicide Risk . . . 101
Reliability: Suicide Risk . . . 101
Unidimensionality: Suicide Risk . . . 102
Graded-Response Model: Suicide Risk . . . 103
Gender-based DIF: Suicide Risk . . . 104
Sample-based DIF: Suicide Risk . . . 104

Study 2: ROC curves 105
Attachment . . . 105
Avoidance . . . 106
Connectedness . . . 107
Demoralization . . . 108
Eating problems . . . 109
Hurtful rumination . . . 110
Hypervigilance . . . 111
Perfectionism . . . 112
Pressure from Negative Affect . . . 113
Psychosis . . . 114
Relational distress . . . 115
Resilience . . . 116
Social Role Functioning . . . 117
Somatic Anxiety . . . 118
Substance Use . . . 119
Suicide Risk . . . 120

Study 3: Scale performance 120
Eating Problems . . . 120
Site 1 . . . 120
Site 2 . . . 123
Site DIF . . . 126
Gender-based DIF: Eating Problems . . . 128
Age-based DIF: Eating Problems . . . 132
Sad Affect . . . 132
Site 1 . . . 132
Site 2 . . . 135
Gender-based DIF: Sad Affect . . . 138
Age-based DIF: Sad Affect . . . 138
Somatic Anxiety . . . 138
Site 1 . . . 138
Site 2 . . . 141
Site DIF . . . 144
Gender-based DIF: Somatic Anxiety . . . 146
Age-based DIF: Somatic Anxiety . . . 150
Substance Use . . . 154
Site 1 . . . 154
Site 2 . . . 157
Site DIF . . . 160
Gender-based DIF: Substance Use . . . 162

Age-based DIF: Substance Use . . . 163
Suicide . . . 165
Site 1 . . . 165
Site 2 . . . 166
Gender-based DIF: Suicide . . . 168
Age-based DIF: Suicide . . . 170
Trauma Reaction . . . 173
Site 1 . . . 173
Site 2 . . . 176
Site DIF . . . 179
Gender-based DIF: Trauma Reaction . . . 181
Age-based DIF: Trauma Reaction . . . 184
Readiness for Recovery . . . 184
Site 1 . . . 184
Site 2 . . . 187
Gender-based DIF: Readiness for Recovery . . . 190
Age-based DIF: Readiness for Recovery . . . 190
Recovery Environment . . . 190
Site 1 . . . 190
Site 2 . . . 193
Site DIF . . . 196
Gender-based DIF: Recovery Environment . . . 196
Age-based DIF: Recovery Environment . . . 199
Social Safety . . . 202
Site 1 . . . 202
Site 2 . . . 205
Site DIF . . . 208
Gender-based DIF: Social Safety . . . 209
Age-based DIF: Social Safety . . . 213
Need for Control . . . 216
Site 1 . . . 216
Site 2 . . . 219
Site DIF . . . 222
Gender-based DIF: Need for Control . . . 222
Age-based DIF: Need for Control . . . 225
Hopelessness . . . 228
Site 1 . . . 228
Site 2 . . . 231
Site DIF . . . 234
Gender-based DIF: Hopelessness . . . 236
Age-based DIF: Hopelessness . . . 239
Internal Avoidance . . . 239
Site 1 . . . 239
Site 2 . . . 242
Site DIF . . . 245
Gender-based DIF: Internal Avoidance . . . 247
Age-based DIF: Internal Avoidance . . . 250
Irritability . . . 253
Site 1 . . . 253
Site 2 . . . 256
Gender-based DIF: Irritability . . . 259
Age-based DIF: Irritability . . . 259
Self-Criticism . . . 259
Site 1 . . . 259

Site 2 . . . 262
Site DIF . . . 265
Gender-based DIF: Self-Criticism . . . 267
Age-based DIF: Self-Criticism . . . 268
Situational Avoidance . . . 271
Site 1 . . . 271
Site 2 . . . 274
Gender-based DIF: Situational Avoidance . . . 277
Age-based DIF: Situational Avoidance . . . 277
Social Avoidance . . . 277
Site 1 . . . 277
Site 2 . . . 280
Gender-based DIF: Social Avoidance . . . 283
Age-based DIF: Social Avoidance . . . 283
Worry . . . 283
Site 1 . . . 283
Site 2 . . . 286
Gender-based DIF: Worry . . . 289
Age-based DIF: Worry . . . 289
Cognitive Problems . . . 289
Site 1 . . . 289
Site 2 . . . 292
Site DIF . . . 295
Gender-based DIF: Cognitive Problems . . . 299
Age-based DIF: Cognitive Problems . . . 302
General Functioning . . . 306
Site 1 . . . 306
Site 2 . . . 309
Gender-based DIF: General Functioning . . . 312
Age-based DIF: General Functioning . . . 312
Substance Recovery . . . 312
Site 1 . . . 312
Site 2 . . . 315
Gender-based DIF: Substance Recovery . . . 315
Age-based DIF: Substance Recovery . . . 315

Session Information **316**
Functions . . . 316
Session Info . . . 322

This is the analysis output companion supplement to "Initial quantitative development of the Norse Feedback system: A novel adaptive multidimensional tool for use in routine mental healthcare." **If you are NOT reading this in the HTML version, we strongly advise that you do so, as this document is too long to navigate as a PDF**. Proceed to this webpage (https://osf.io/6xvmf/?view_only=fcbfbb26e65c4c7bbb6e8cede3e975bc), download the HTML version, and open that file using your web browser. The contents are identical to this document.

Summary

This supplement is ordered roughly by the order of the manuscript.

The *Preliminary* section covers some essential but not substantively important work. Specifically it includes the R language code for the custom functions used in these analyses, to ease replication.

In *Study 2: Scale performance*, we present information on each scale's reliability (Cronbach's alpha, mean item-total correlation, and alpha if removed statistics), unidimensionality (scree plot, eigenvalue ratio, actual eigenvalues, and factor analysis output including goodness of fit), a graded response model for that scale, along with DIF analyses for gender and sample (clinical vs. nonclinical). The outputs are a combination of standard software-generated outputs and sythesized results.

These analytic outputs are already described in the text, but as we could not feasibly include all meaningful information for each scale in a single table, interested readers are directed to these individual scale analyses. Relevant DIF output includes the number of items flagged for DIF, along with the magnitude of effect size.

In *Study 2: ROC curves*, we present visual representations of the scales' discrimination abilities between the clinical and nonclinical samples in this study.

In *Study 3: Scale performance*, we present very similar analyses to those presented in *Study 2: Scale performance*. The primary differences are in the group comparisons for DIF analysis. Outputs are similar or exactly the same.

The *Session Information* section contains the custom functions and loaded packages required to run these analyses.

Preliminary

Loaded libraries are found at the end of this document, along with version numbers of packages and R. We omit the call here for legibility.

Functions

Several convenience and analysis functions are defined at this point in the report with suppressed output, and are repeated in the *Session Information* section at the end of the document.

These functions rely on objects of type `grm` from the package `{ltm}` and type `roc` from the package `{pROC}`, among others. Again, they are actually defined at this point in the analysis code, but are not presented until the final section of this document for reference.

All installed packages are included in the Session Information section below. The subsequent code loops through different repeatable reports, each with several functions for each scale, and knits a single report from them.

Reading data

This section imports data from external repositories, the location of which is obscured for security reasons. Some (but not all) analysis objects are read into the global environment from previously-saved locations. This reduces compilation time of this supplement. Reproduction of analyses is still demonstrated within the code below. Interested readers may examine all code upon request (to AAM), though most analysis code is not included for here for clarity.

Study 2: Scale performance

Attachment

Reliability: Attachment

```
## Cronbach's alpha is 0.607.
## Mean item-total correlation is 0.278.
## If each item were dropped:
##      raw_alpha std.alpha G6(smc) average_r  S/N alpha se   var.r med.r
## Q41       0.49      0.49    0.40      0.24 0.96    0.035 0.00670  0.21
## Q1        0.57      0.57    0.47      0.30 1.31    0.030 0.00089  0.30
## Q50-      0.54      0.54    0.45      0.29 1.20    0.032 0.00584  0.28
## Q89       0.54      0.54    0.45      0.28 1.17    0.032 0.00885  0.30
```

Unidimensionality: Attachment

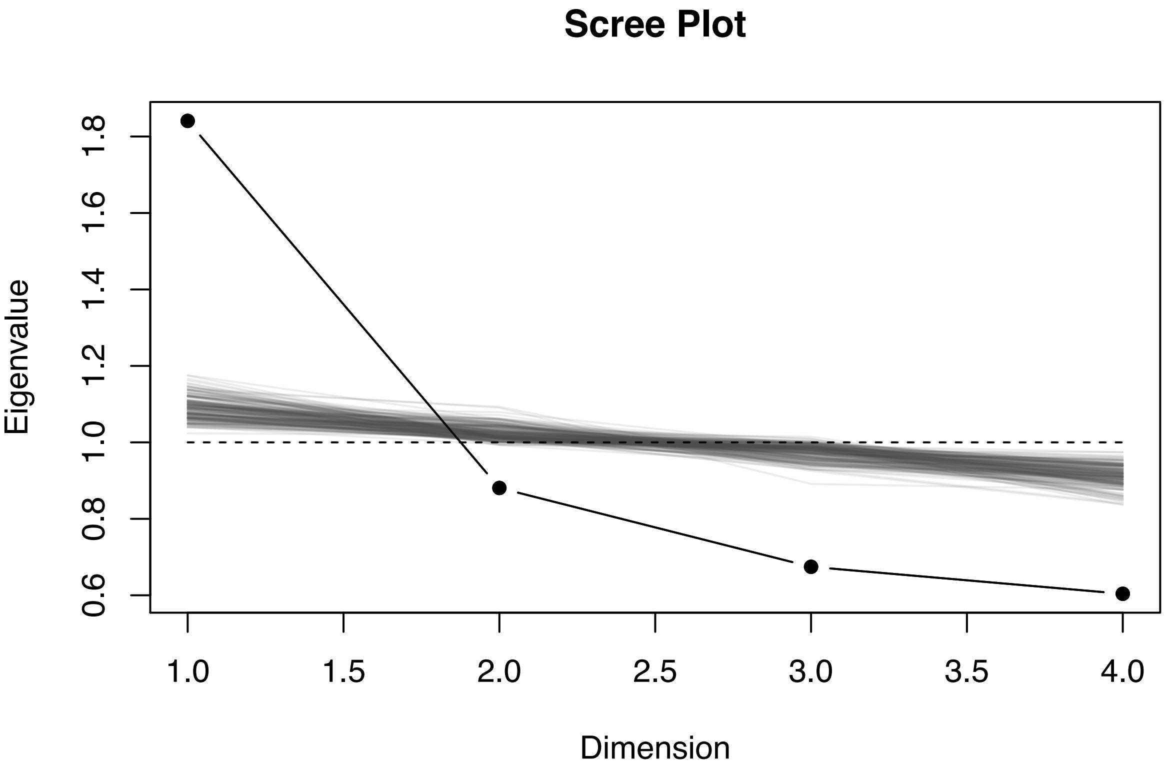


```
## [1] "Ratio of first to second eigenvalues: 2.09"

## [1] 1.8408832 0.8807387 0.6744616 0.6039165

## Factor Analysis using method =  minres
## Call: fa(r = grm_obj$X)
## Standardized loadings (pattern matrix) based upon correlation matrix
##       MR1   h2   u2 com
## Q41  0.63 0.40 0.60   1
## Q1   0.47 0.22 0.78   1
## Q50 -0.50 0.25 0.75   1
## Q89  0.51 0.26 0.74   1
```

```
##
##                 MR1
## SS loadings    1.14
## Proportion Var 0.28
##
## Mean item complexity =  1
## Test of the hypothesis that 1 factor is sufficient.
##
## The degrees of freedom for the null model are  6  and the objective function was  0.41 with Chi Squa
## The degrees of freedom for the model are 2  and the objective function was  0.03
##
## The root mean square of the residuals (RMSR) is  0.05
## The df corrected root mean square of the residuals is  0.09
##
## The harmonic number of observations is  600 with the empirical chi square  19.9  with prob <  4.8e-0
## The total number of observations was  619  with Likelihood Chi Square =  18.96  with prob <  7.6e-05
##
## Tucker Lewis Index of factoring reliability =  0.796
## RMSEA index =  0.117  and the 90 % confidence intervals are  0.073 0.168
## BIC =  6.1
## Fit based upon off diagonal values = 0.97
## Measures of factor score adequacy
##                                                    MR1
## Correlation of (regression) scores with factors   0.79
## Multiple R square of scores with factors          0.62
## Minimum correlation of possible factor scores     0.24
```

Graded-Response Model: Attachment

```
##     Extrmt1 Extrmt2 Extrmt3 Extrmt4 Extrmt5 Extrmt6 Dscrmn
## Q41  -2.521  -1.489  -0.855  -0.270   0.270   0.920  1.659
## Q1   -3.596  -2.512  -1.895  -1.186  -0.477   0.547  1.128
## Q50   3.150   1.824   0.999   0.230  -0.497  -1.300 -1.030
## Q89  -2.350  -1.344  -0.483   0.459   1.214   2.079  1.106
```

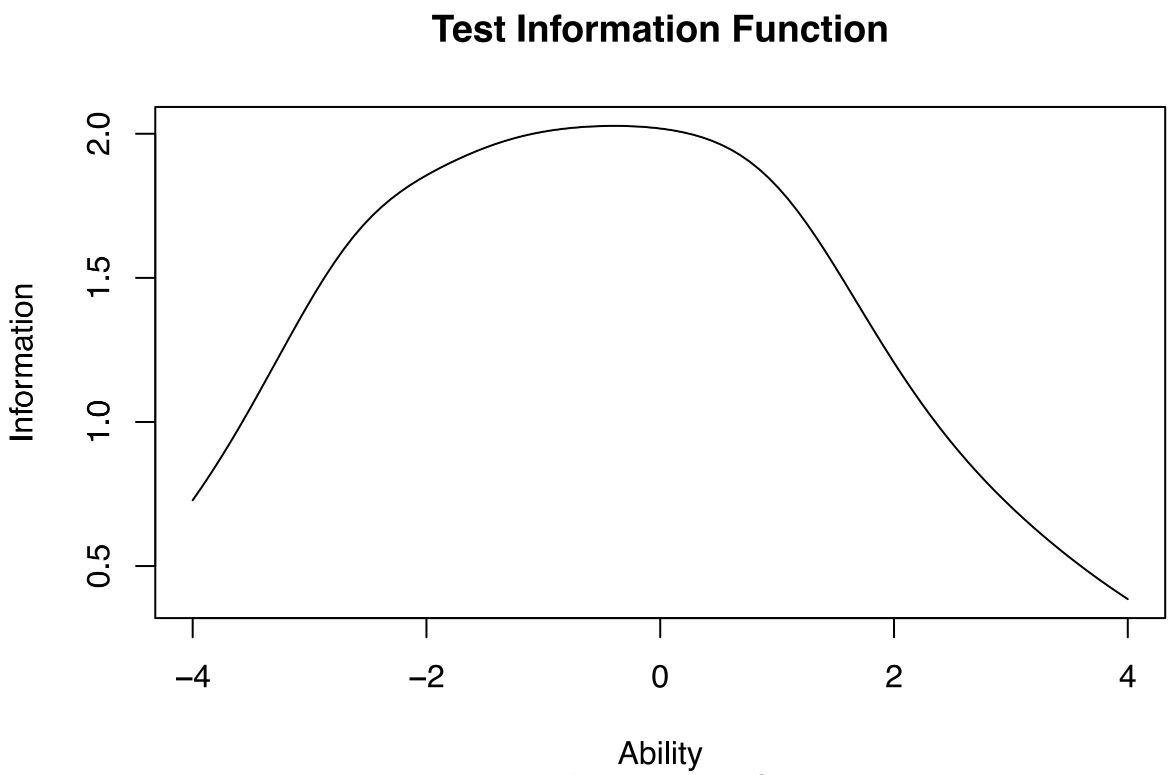

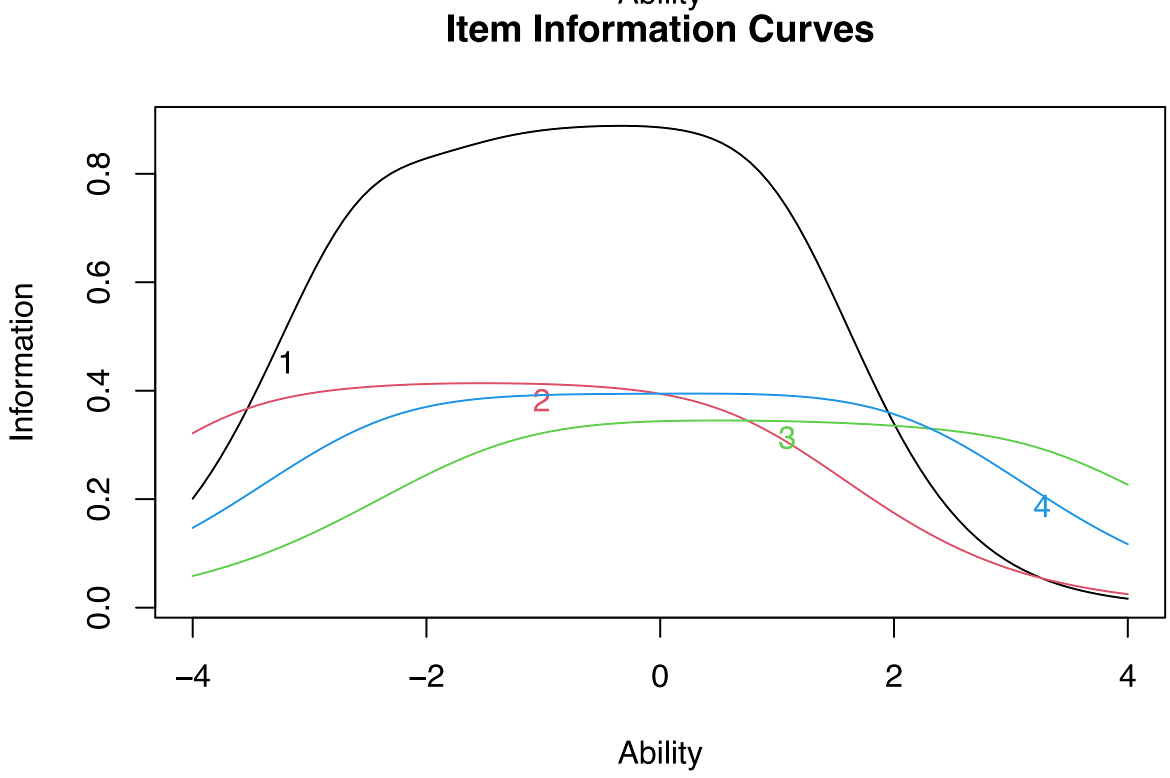

Gender-based DIF: Attachment

```
## Call:
## lordif::lordif(resp.data = as.data.frame(sex.data), group = sex)
```

```
##
##   Number of DIF groups: 2
##
##   Number of items flagged for DIF: 2 of 4
##
##   Items flagged: 2, 3
##
##   Number of iterations for purification: 3 of 10
##
##   Detection criterion: Chisqr
##
##   Threshold: alpha = 0.01
##
##   item ncat  chi12  chi13  chi23
## 1    1    7 0.0406 0.1139 0.6945
## 2    2    7 0.0000 0.0000 0.8835
## 3    3    7 0.0697 0.0000 0.0000
## 4    4    7 0.5904 0.7356 0.5690
```

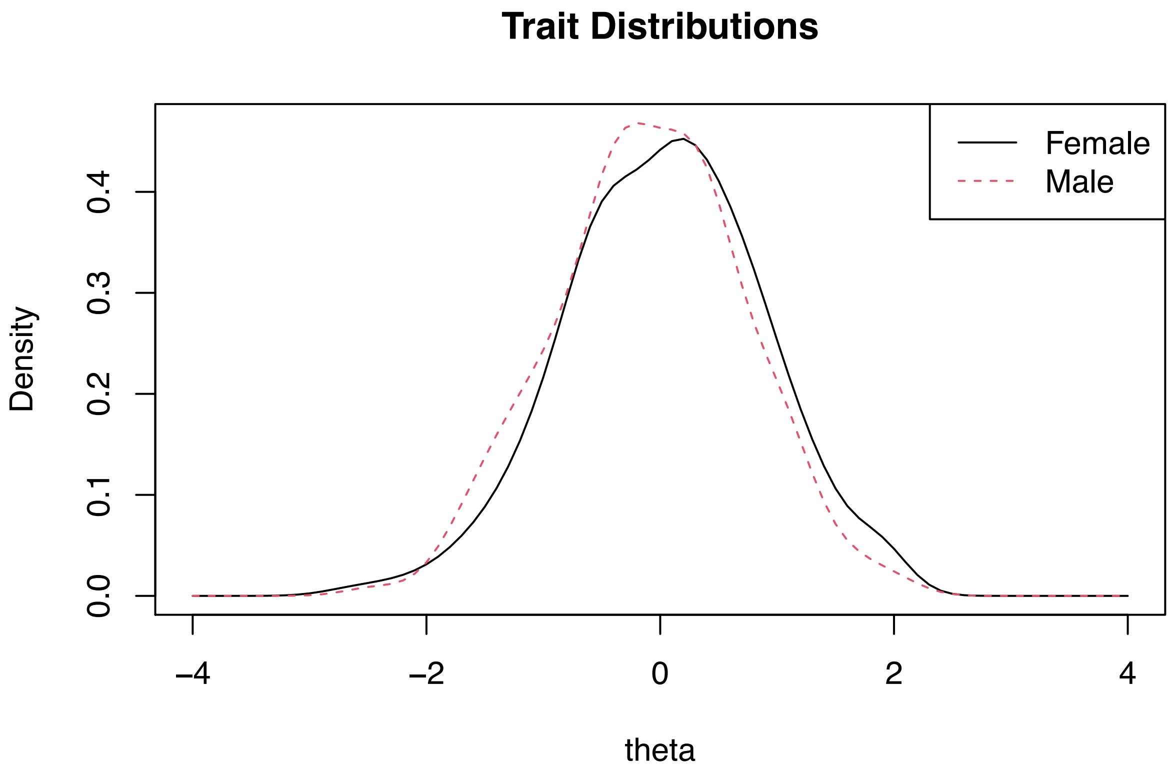

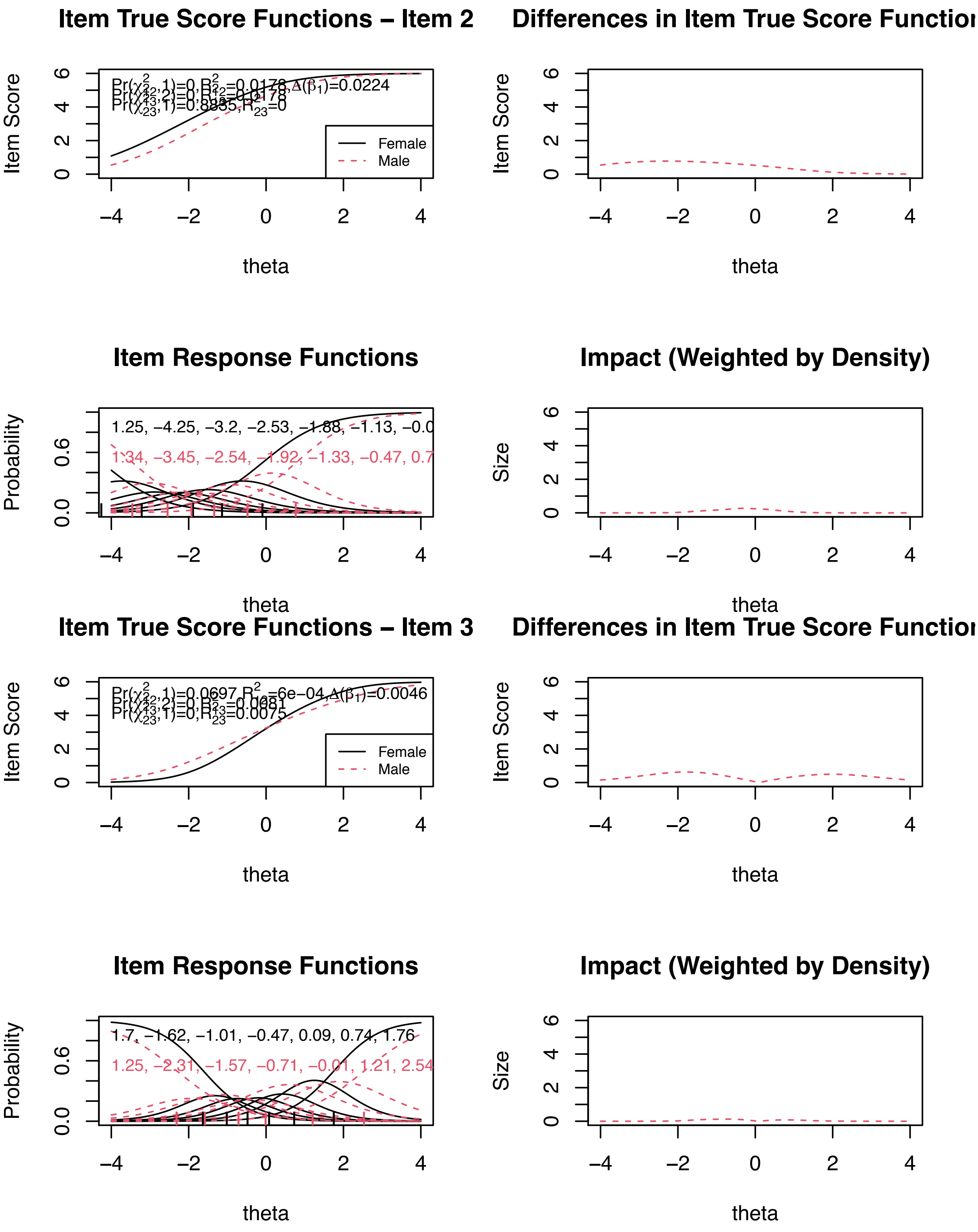
Item True Score Functions – Item 2
Differences in Item True Score Functio
Item Score
theta
Female
Male
Item Response Functions
Impact (Weighted by Density)
Probability
Size
1.25, −4.25, −3.2, −2.53, −1.88, −1.13, −0.
1.34, −3.45, −2.54, −1.92, −1.33, −0.47, 0.7
Item True Score Functions – Item 3
1.7, −1.62, −1.01, −0.47, 0.09, 0.74, 1.76
1.25, −2.31, −1.57, −0.71, −0.01, 1.21, 2.54

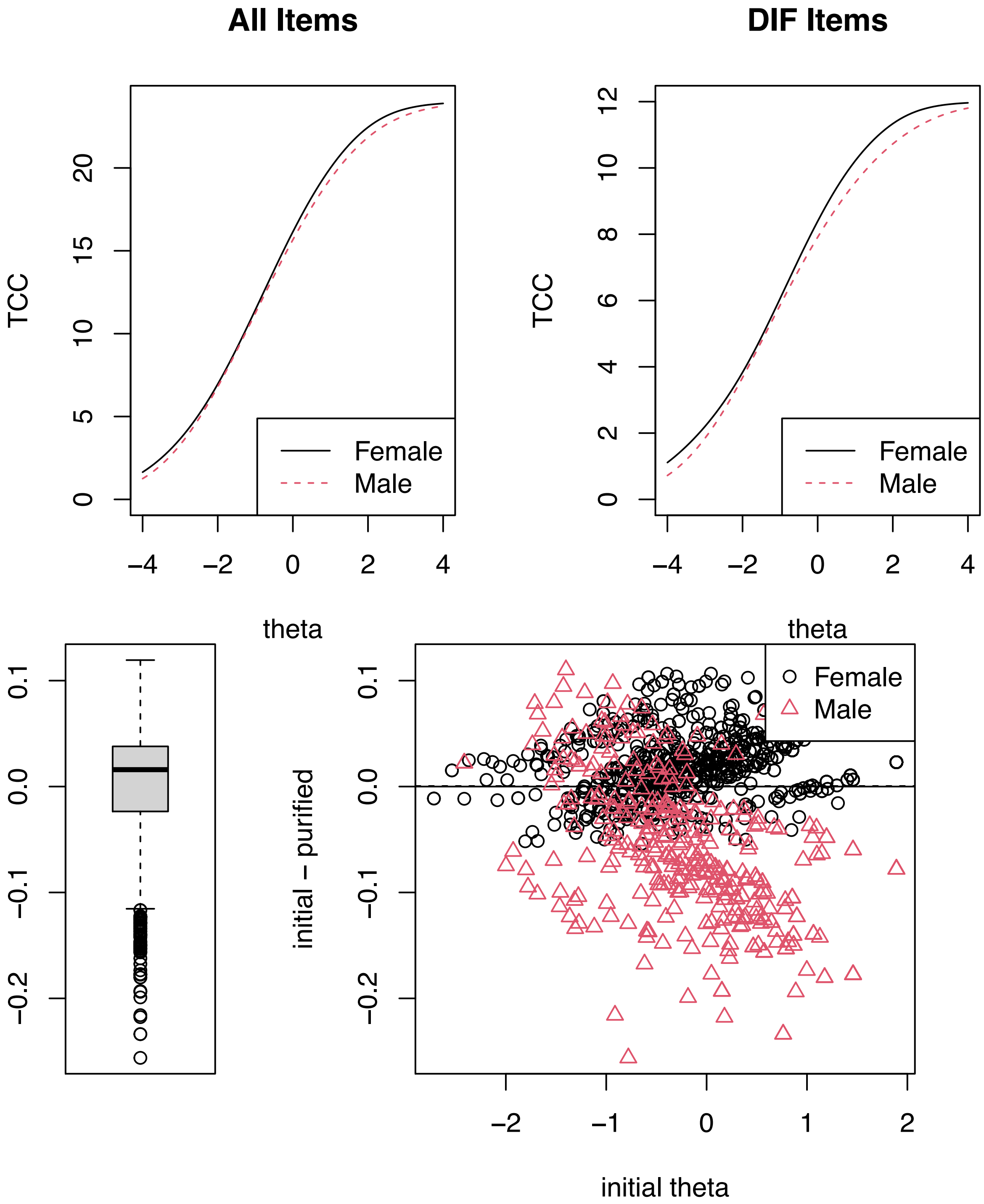

Sample-based DIF: Attachment

```
## Call:
## lordif::lordif(resp.data = as.data.frame(sample.data), group = clinYN)
##
##   Number of DIF groups: 2
##
```

```
##   Number of items flagged for DIF: 4 of 4
##
##   Items flagged: 1, 2, 3, 4
##
##   Number of iterations for purification: 2 of 10
##
##   Detection criterion: Chisqr
##
##   Threshold: alpha = 0.01
##
##   item ncat  chi12  chi13  chi23
## 1    1    7 0.0000 0.0000 0.0117
## 2    2    6 0.0179 0.0048 0.0241
## 3    3    7 0.0000 0.0000 0.0633
## 4    4    7 0.0000 0.0000 0.0627
```

Back to top

Avoidance

Reliability: Avoidance

```
## Cronbach's alpha is 0.663.
## Mean item-total correlation is 0.242.
## If each item were dropped:
##     raw_alpha std.alpha G6(smc) average_r S/N alpha se var.r med.r
## Q78      0.61      0.60    0.59      0.23 1.5    0.024 0.026  0.21
## Q8       0.58      0.58    0.54      0.21 1.4    0.026 0.015  0.19
## Q10      0.69      0.69    0.66      0.31 2.2    0.019 0.012  0.31
## Q17      0.67      0.66    0.64      0.28 1.9    0.021 0.020  0.30
## Q34      0.58      0.57    0.55      0.21 1.3    0.027 0.021  0.19
## Q64      0.58      0.57    0.55      0.21 1.3    0.026 0.018  0.20
```

Unidimensionality: Avoidance

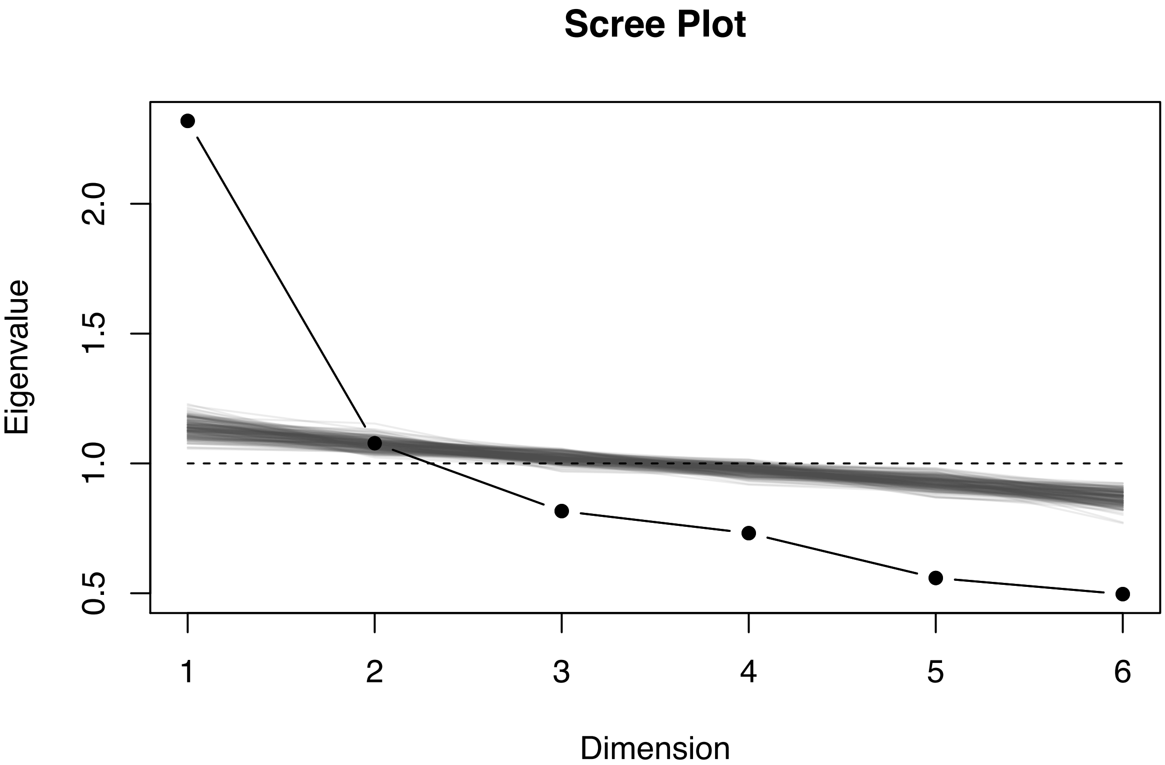


```
## [1] "Ratio of first to second eigenvalues: 2.152"

## [1] 2.3191260 1.0777077 0.8164151 0.7313954 0.5587366 0.4966192

## Factor Analysis using method =  minres
## Call: fa(r = grm_obj$X)
## Standardized loadings (pattern matrix) based upon correlation matrix
##      MR1    h2   u2 com
## Q78 0.51 0.265 0.74   1
## Q8  0.65 0.422 0.58   1
## Q10 0.20 0.041 0.96   1
## Q17 0.33 0.108 0.89   1
## Q34 0.64 0.404 0.60   1
## Q64 0.66 0.438 0.56   1
##
##                 MR1
## SS loadings    1.68
## Proportion Var 0.28
##
## Mean item complexity =  1
## Test of the hypothesis that 1 factor is sufficient.
##
## The degrees of freedom for the null model are  15  and the objective function was  0.88 with Chi Squa
## The degrees of freedom for the model are 9  and the objective function was  0.09
##
## The root mean square of the residuals (RMSR) is  0.06
## The df corrected root mean square of the residuals is  0.07
##
```

```
## The harmonic number of observations is  588 with the empirical chi square  56.01  with prob <  7.8e-[illegible]
## The total number of observations was  619  with Likelihood Chi Square =  52.48  with prob <  3.7e-08
##
## Tucker Lewis Index of factoring reliability =  0.862
## RMSEA index =  0.088  and the 90 % confidence intervals are  0.066 0.112
## BIC =  -5.37
## Fit based upon off diagonal values = 0.96
## Measures of factor score adequacy
##                                                    MR1
## Correlation of (regression) scores with factors   0.85
## Multiple R square of scores with factors          0.73
## Minimum correlation of possible factor scores     0.46
```

Graded-Response Model: Avoidance

```
##     Extrmt1 Extrmt2 Extrmt3 Extrmt4 Extrmt5 Extrmt6 Dscrmn
## Q78  -1.691  -1.103  -0.620   0.011   0.690   1.603  1.204
## Q8   -0.959  -0.378   0.007   0.503   1.016   1.702  1.758
## Q10  -4.307  -2.102  -0.561   1.571   3.157   5.490  0.426
## Q17   0.239   1.113   1.539   2.111   2.758   3.607  0.793
## Q34  -1.558  -0.925  -0.518  -0.044   0.536   1.156  1.684
## Q64  -1.162  -0.468   0.014   0.598   1.076   1.768  1.722
```

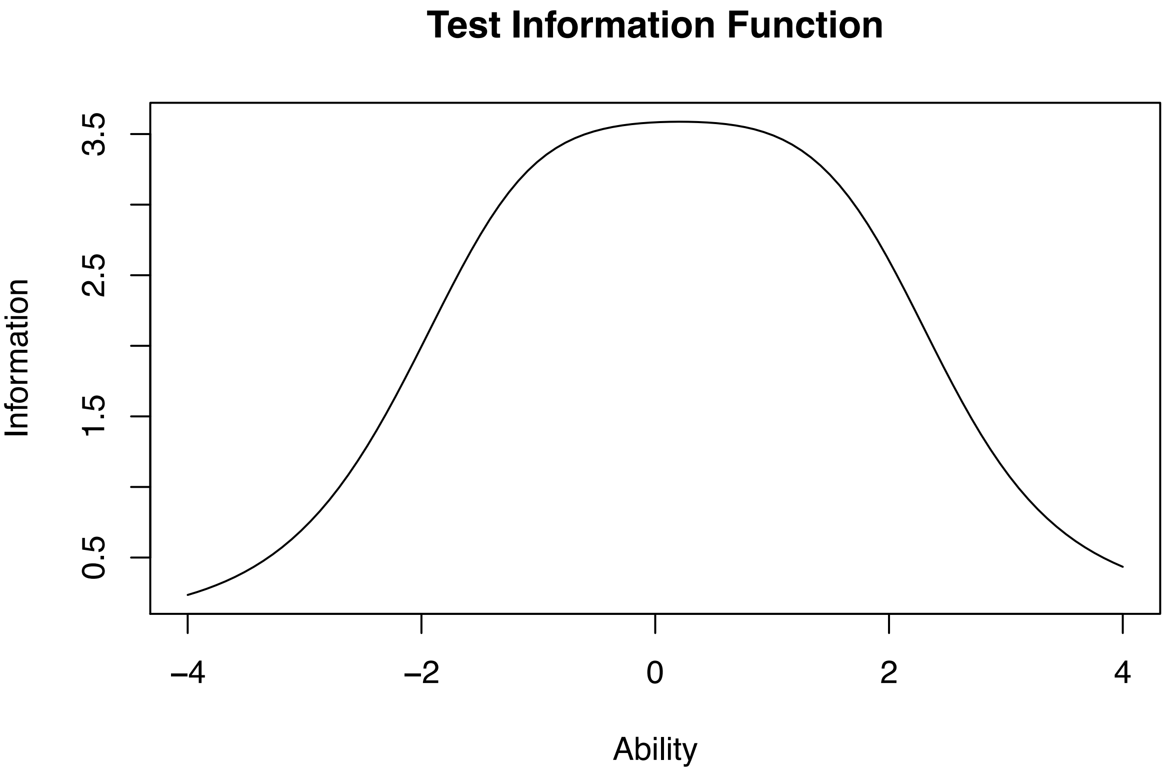

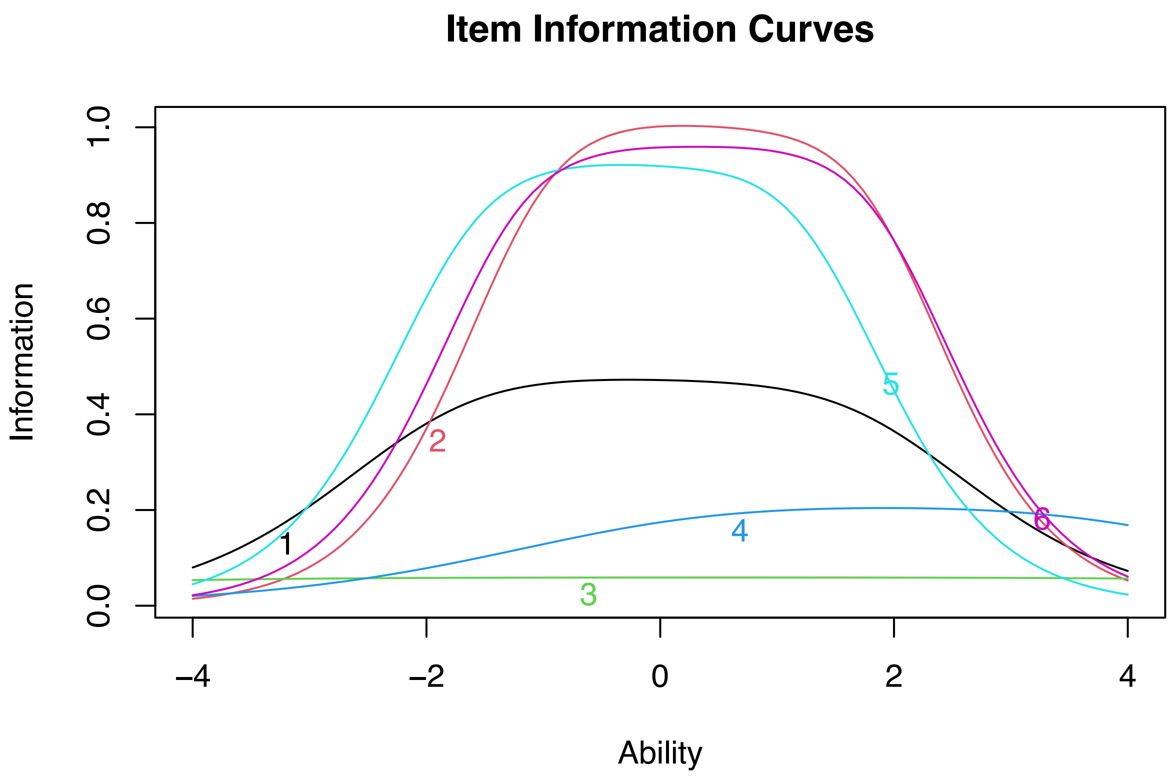

Gender-based DIF: Avoidance

```
## Call:
## lordif::lordif(resp.data = as.data.frame(sex.data), group = sex)
##
##   Number of DIF groups: 2
##
##   Number of items flagged for DIF: 3 of 6
##
##   Items flagged: 4, 5, 6
##
##   Number of iterations for purification: 2 of 10
##
##   Detection criterion: Chisqr
##
##   Threshold: alpha = 0.01
##
##   item ncat  chi12  chi13  chi23
## 1    1    7 0.5349 0.8207 0.9195
## 2    2    7 0.2344 0.4385 0.6278
## 3    3    7 0.0718 0.1315 0.3664
## 4    4    7 0.0039 0.0157 0.9387
## 5    5    7 0.0306 0.0005 0.0012
## 6    6    7 0.0002 0.0001 0.0636
```

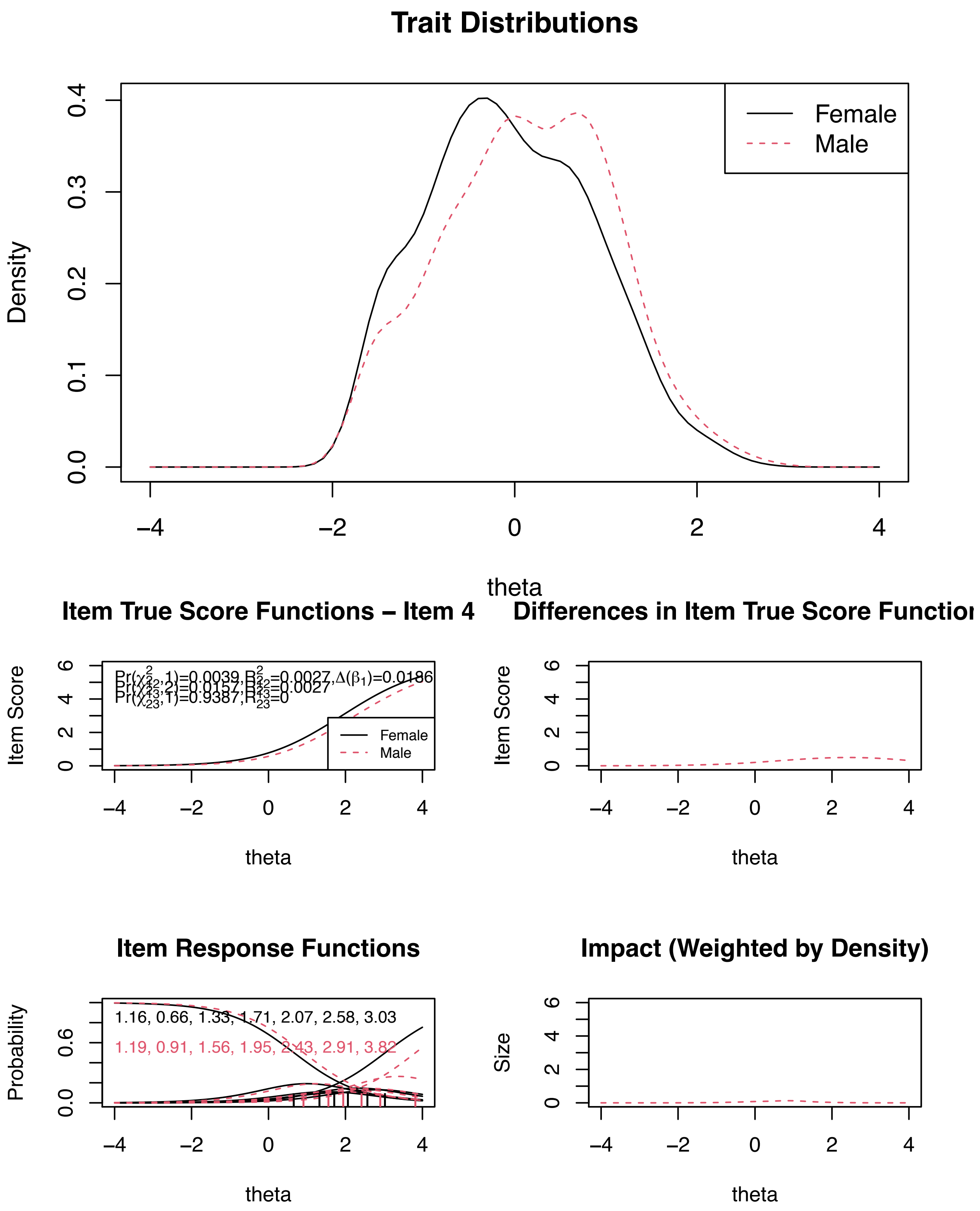
Trait Distributions
Density
theta
Female
Male
Item True Score Functions – Item 4
Differences in Item True Score Functio
Item Score
Female
Male
Item Response Functions
Probability
1.16, 0.66, 1.33, 1.71, 2.07, 2.58, 3.03
1.19, 0.91, 1.56, 1.95, 2.43, 2.91, 3.82
Impact (Weighted by Density)
Size

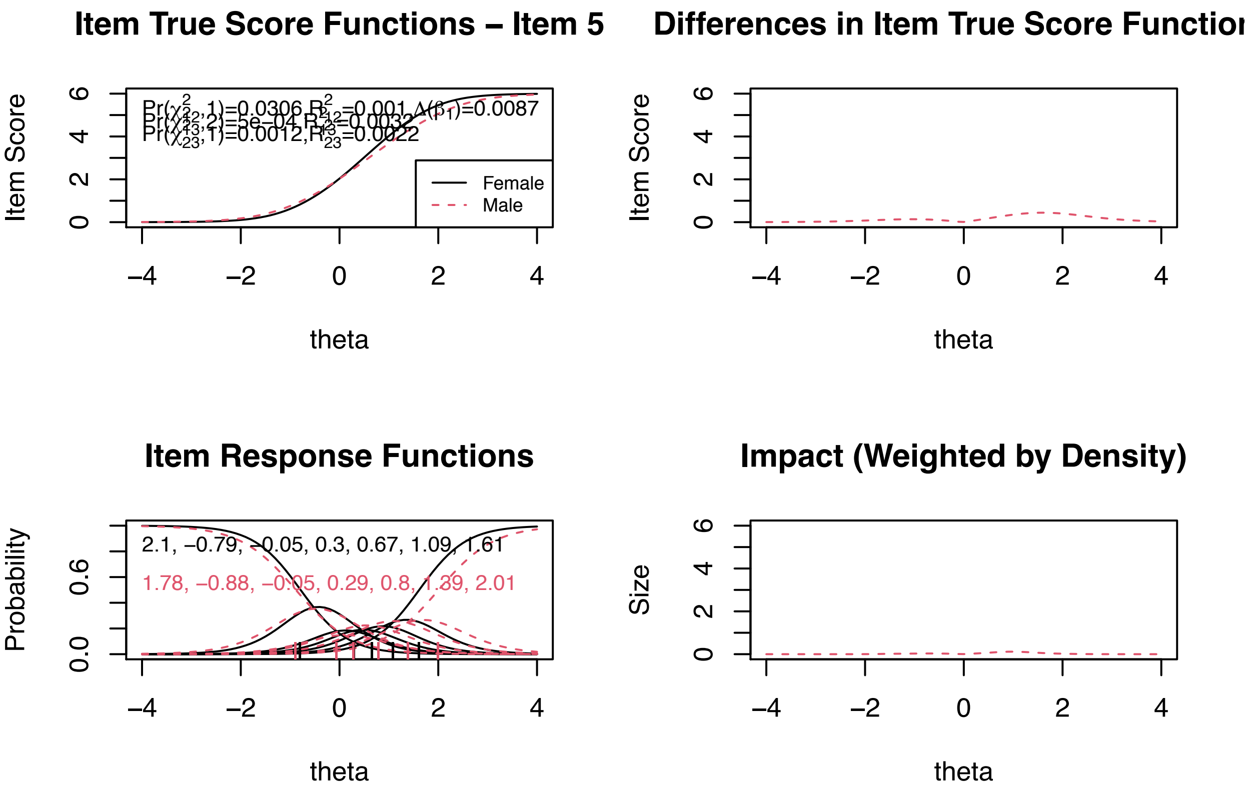
Item True Score Functions – Item 5
Pr(χ212,1)=0.0306,R212=0.001,Δ(β1)=0.0087
Pr(χ213,2)=5e−04,R213=0.0032
Pr(χ223,1)=0.0012,R223=0.0022
Female
Male
Item Score
theta
Differences in Item True Score Functio
Item Score
theta
Item Response Functions
2.1, −0.79, −0.05, 0.3, 0.67, 1.09, 1.61
1.78, −0.88, −0.05, 0.29, 0.8, 1.39, 2.01
Probability
theta
Impact (Weighted by Density)
Size
theta

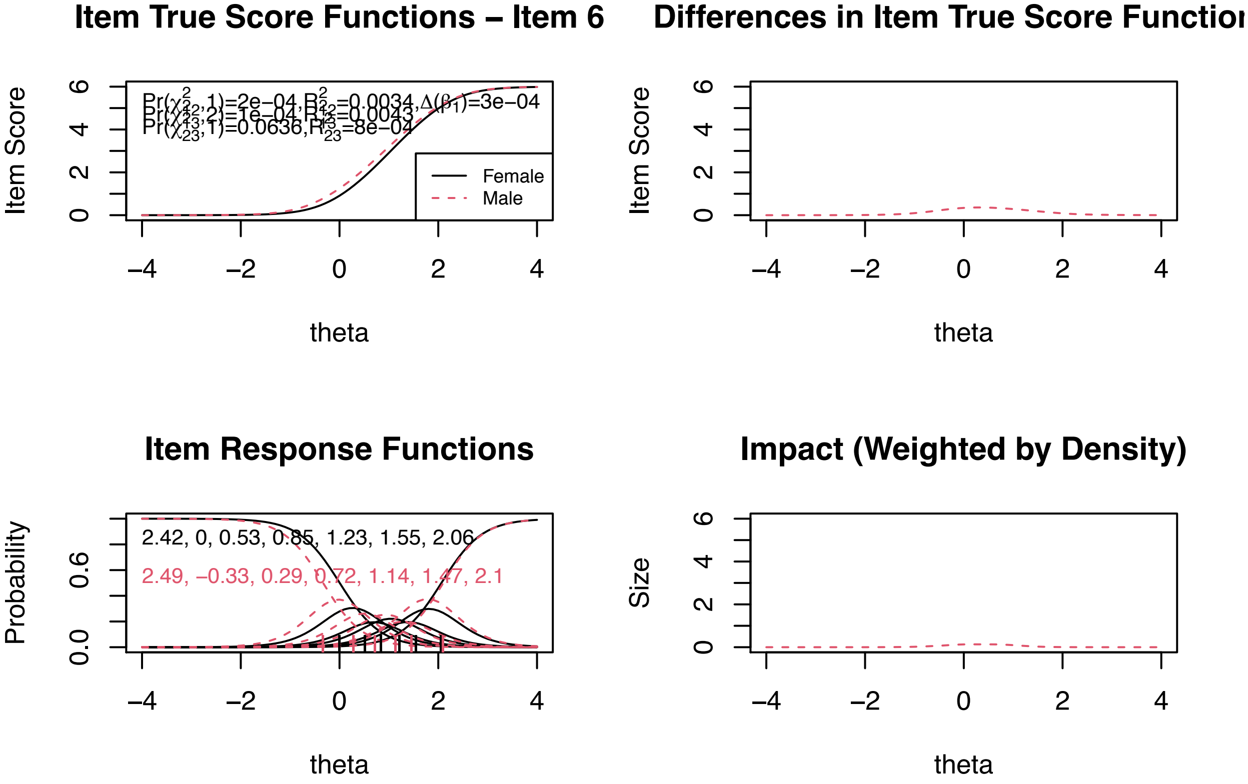
Item True Score Functions – Item 6
Pr(χ212,1)=2e−04,R212=0.0034,Δ(β1)=3e−04
Pr(χ213,2)=1e−04,R213=0.0043
Pr(χ223,1)=0.0636,R223=8e−04
Female
Male
Item Score
theta
Differences in Item True Score Functio
Item Score
theta
Item Response Functions
2.42, 0, 0.53, 0.85, 1.23, 1.55, 2.06
2.49, −0.33, 0.29, 0.72, 1.14, 1.47, 2.1
Probability
theta
Impact (Weighted by Density)
Size
theta

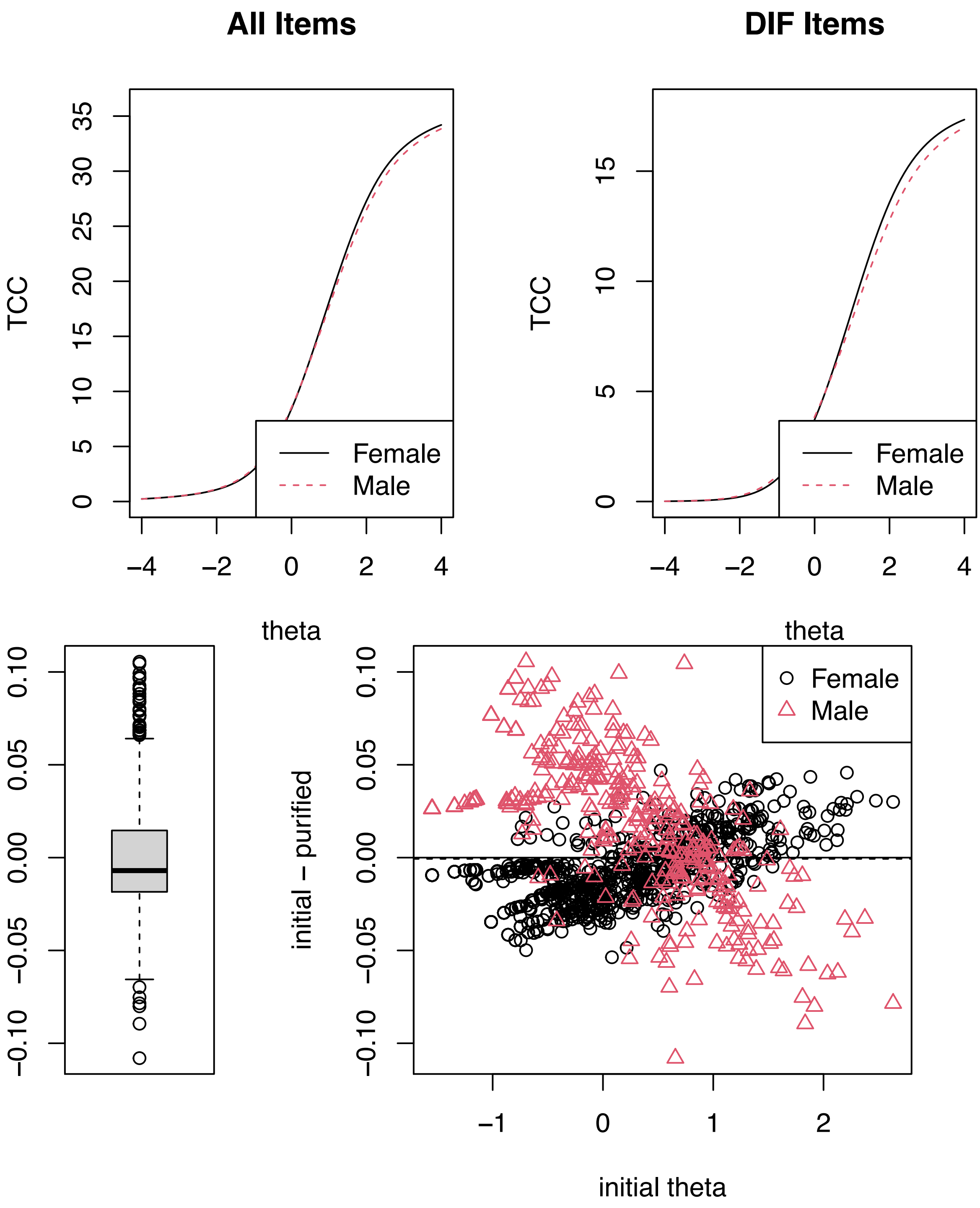

Sample-based DIF: Avoidance

```
## Call:
## lordif::lordif(resp.data = as.data.frame(sample.data), group = clinYN)
##
##   Number of DIF groups: 2
##
```

```
##   Number of items flagged for DIF: 6 of 6
##
##   Items flagged: 1, 2, 3, 4, 5, 6
##
##   Number of iterations for purification: 2 of 10
##
##   Detection criterion: Chisqr
##
##   Threshold: alpha = 0.01
##
##   item ncat  chi12 chi13  chi23
## 1    1    7 0.0000 0e+00 0.0001
## 2    2    7 0.0000 0e+00 0.8427
## 3    3    7 0.0000 0e+00 0.8806
## 4    4    7 0.0214 1e-04 0.0004
## 5    5    7 0.0000 0e+00 0.0001
## 6    6    6 0.0000 0e+00 0.8568
```

Back to top

Connectedness

Reliability: Connectedness

```
## Cronbach's alpha is 0.71.
## Mean item-total correlation is 0.263.
## If each item were dropped:
##      raw_alpha std.alpha G6(smc) average_r S/N alpha se  var.r med.r
## Q43-      0.66      0.67    0.65      0.25 2.0    0.021 0.0095  0.23
## Q45       0.72      0.72    0.71      0.30 2.6    0.017 0.0104  0.29
## Q50-      0.68      0.68    0.67      0.26 2.1    0.020 0.0135  0.23
## Q54       0.70      0.70    0.68      0.28 2.4    0.019 0.0112  0.28
## Q62       0.65      0.66    0.64      0.24 1.9    0.022 0.0097  0.23
## Q69       0.66      0.67    0.65      0.25 2.0    0.021 0.0140  0.21
## Q80       0.67      0.68    0.66      0.26 2.1    0.020 0.0138  0.21
```

Unidimensionality: Connectedness

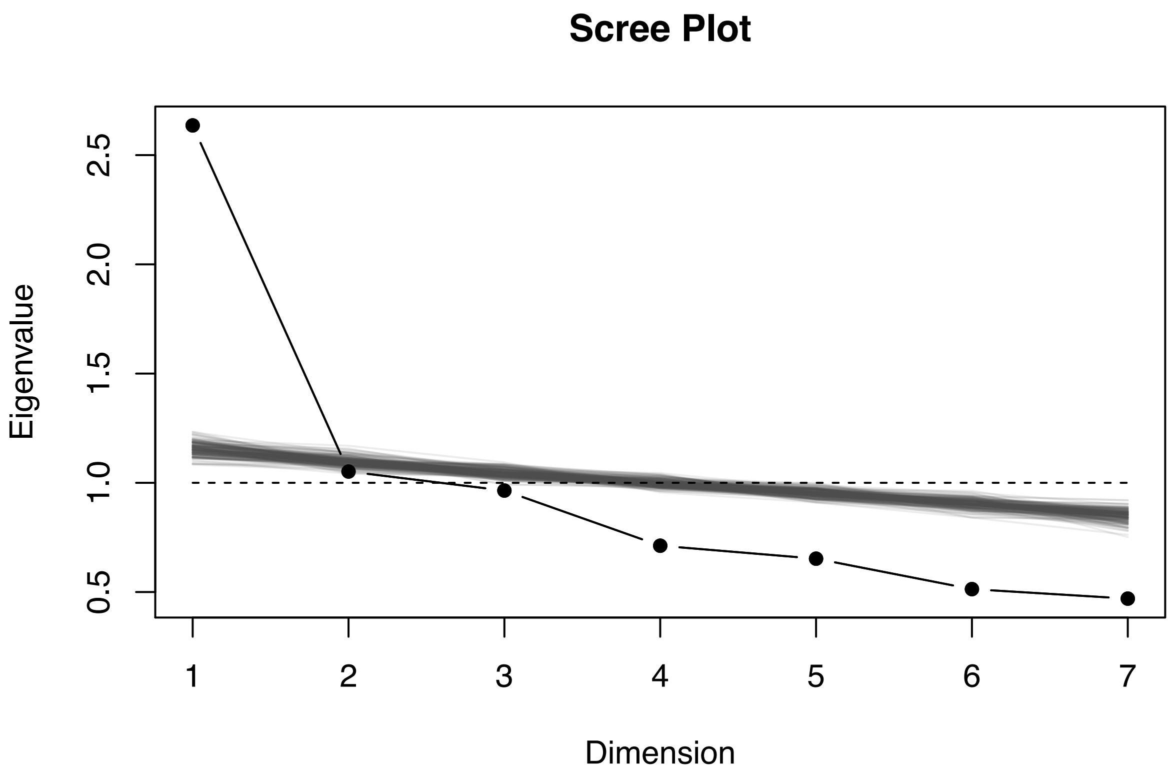


```
## [1] "Ratio of first to second eigenvalues: 2.506"

## [1] 2.6359076 1.0516989 0.9643826 0.7120680 0.6526773 0.5133448 0.4699208

## Factor Analysis using method =  minres
## Call: fa(r = grm_obj$X)
## Standardized loadings (pattern matrix) based upon correlation matrix
##       MR1    h2   u2 com
## Q43 -0.62 0.385 0.61   1
## Q45  0.30 0.092 0.91   1
## Q50 -0.53 0.284 0.72   1
## Q54  0.39 0.151 0.85   1
## Q62  0.66 0.437 0.56   1
## Q69  0.56 0.314 0.69   1
## Q80  0.54 0.292 0.71   1
##
##                 MR1
## SS loadings    1.96
## Proportion Var 0.28
##
## Mean item complexity =  1
## Test of the hypothesis that 1 factor is sufficient.
##
## The degrees of freedom for the null model are  21  and the objective function was  1.2 with Chi Squa
## The degrees of freedom for the model are 14  and the objective function was  0.2
##
## The root mean square of the residuals (RMSR) is  0.07
## The df corrected root mean square of the residuals is  0.09
```

```
##
## The harmonic number of observations is  570 with the empirical chi square  122.58  with prob <  2e-1
## The total number of observations was  619  with Likelihood Chi Square =  121.65  with prob <  3e-19
##
## Tucker Lewis Index of factoring reliability =  0.775
## RMSEA index =  0.111  and the 90 % confidence intervals are  0.094 0.13
## BIC =  31.66
## Fit based upon off diagonal values = 0.94
## Measures of factor score adequacy
##                                                   MR1
## Correlation of (regression) scores with factors   0.86
## Multiple R square of scores with factors          0.75
## Minimum correlation of possible factor scores     0.49
```

Graded-Response Model: Connectedness

```
##     Extrmt1 Extrmt2 Extrmt3 Extrmt4 Extrmt5 Extrmt6 Dscrmn
## Q43  -1.674  -1.011  -0.456   0.110   0.606   1.500  1.618
## Q45   2.043   0.908   0.269  -0.914  -1.641  -2.948 -0.593
## Q50  -2.858  -1.662  -0.909  -0.208   0.457   1.184  1.189
## Q54   3.218   1.807   0.873  -0.063  -0.955  -1.899 -0.750
## Q62   1.825   1.107   0.628   0.222  -0.209  -0.857 -1.851
## Q69   1.771   1.017   0.422  -0.344  -0.850  -1.550 -1.191
## Q80   3.328   2.116   1.632   0.945   0.281  -0.498 -1.309
```

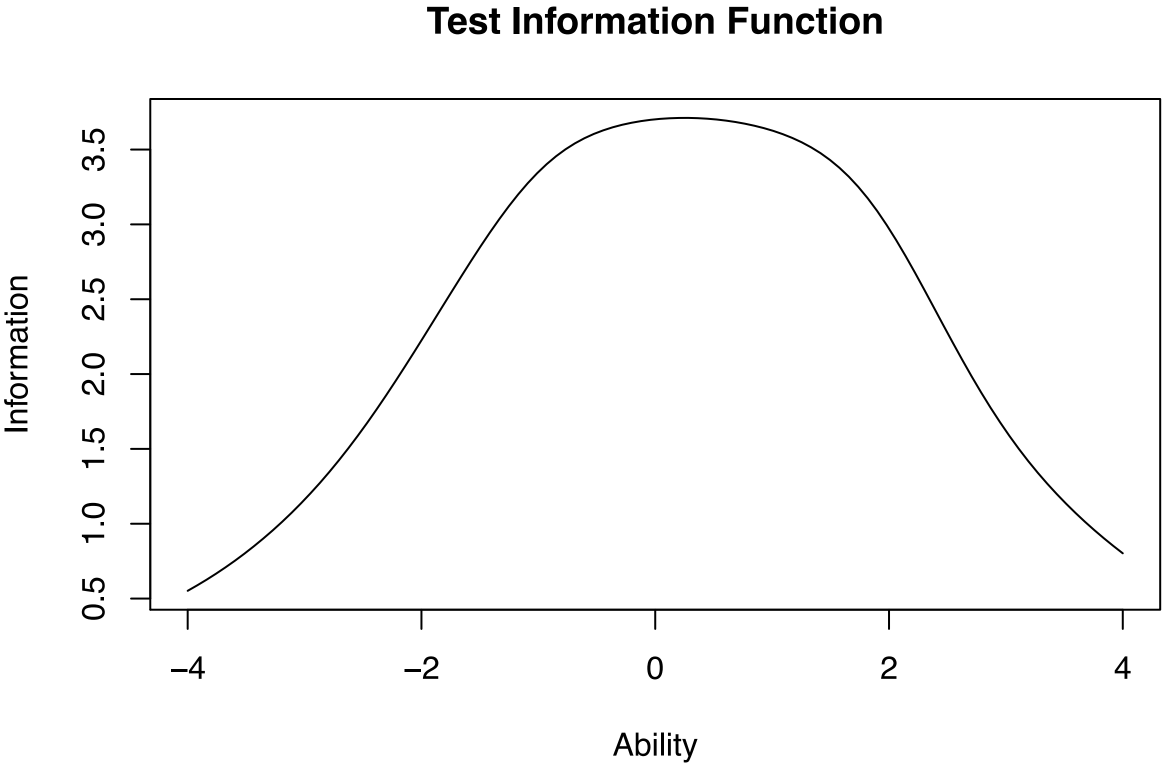

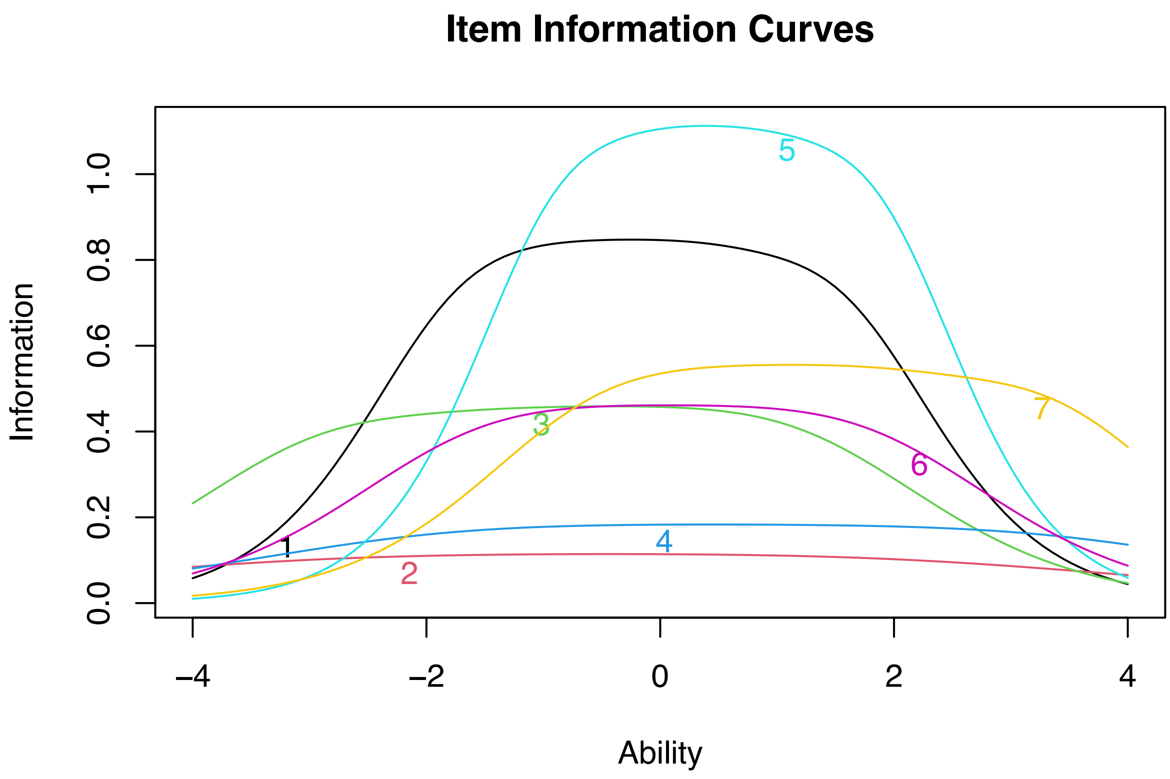

Gender-based DIF: Connectedness

```
## Call:
## lordif::lordif(resp.data = as.data.frame(sex.data), group = sex)
##
##   Number of DIF groups: 2
##
##   Number of items flagged for DIF: 3 of 7
##
##   Items flagged: 2, 5, 7
##
##   Number of iterations for purification: 4 of 10
##
##   Detection criterion: Chisqr
##
##   Threshold: alpha = 0.01
##
##   item ncat  chi12  chi13  chi23
## 1    1    7 0.0484 0.0636 0.2042
## 2    2    7 0.0002 0.0000 0.0012
## 3    3    7 0.0845 0.0774 0.1434
## 4    4    7 0.2402 0.2957 0.3039
## 5    5    7 0.0000 0.0000 0.0449
## 6    6    7 0.6212 0.8710 0.8580
## 7    7    7 0.0000 0.0001 0.7625
```

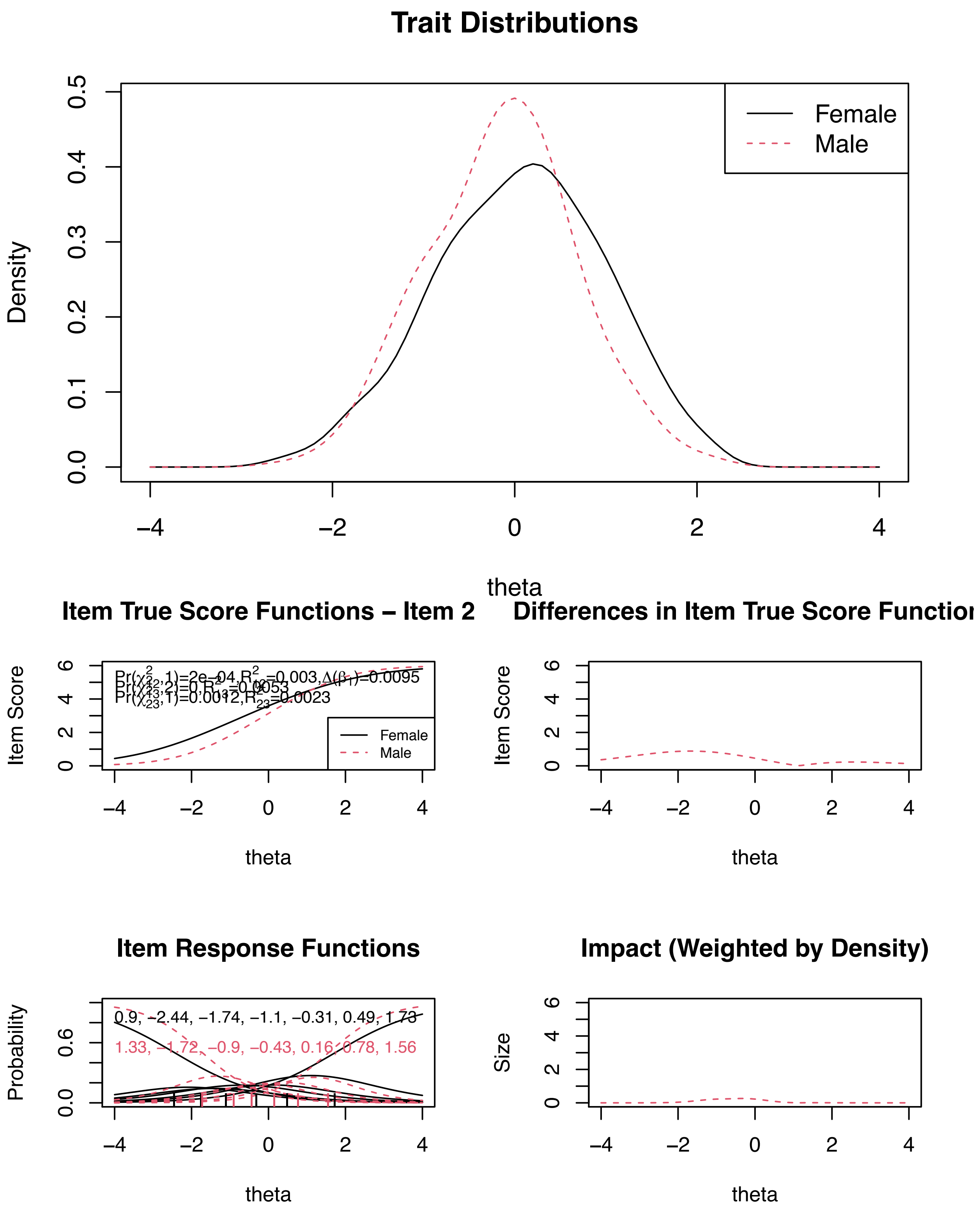
Trait Distributions
Density
theta
Female
Male
Item True Score Functions – Item 2
Item Score
Female
Male
Differences in Item True Score Functio
Item Response Functions
Probability
0.9, −2.44, −1.74, −1.1, −0.31, 0.49, 1.73
1.33, −1.72, −0.9, −0.43, 0.16, 0.78, 1.56
Impact (Weighted by Density)
Size

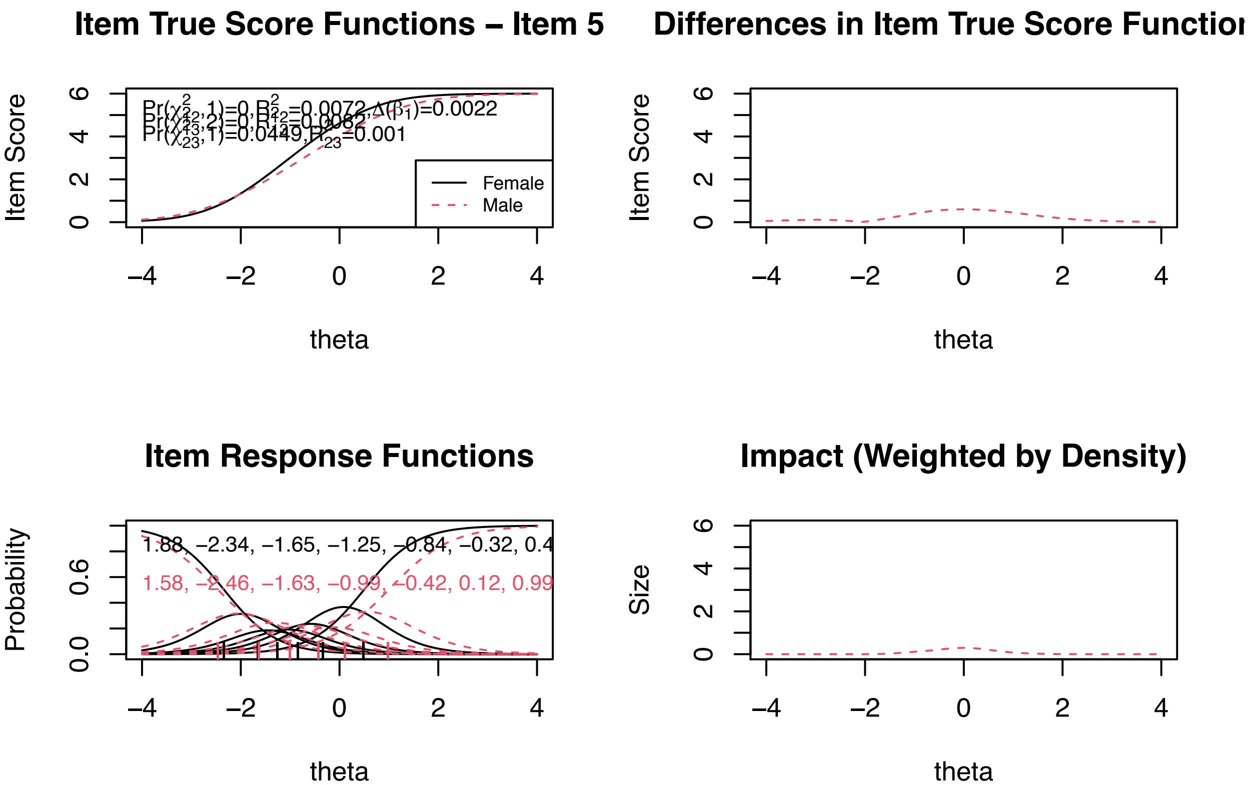
Item True Score Functions – Item 5
Differences in Item True Score Functio
Item Score
theta
Female
Male
Item Response Functions
Probability
1.88, –2.34, –1.65, –1.25, –0.84, –0.32, 0.4
1.58, –2.46, –1.63, –0.99, –0.42, 0.12, 0.99
Impact (Weighted by Density)
Size

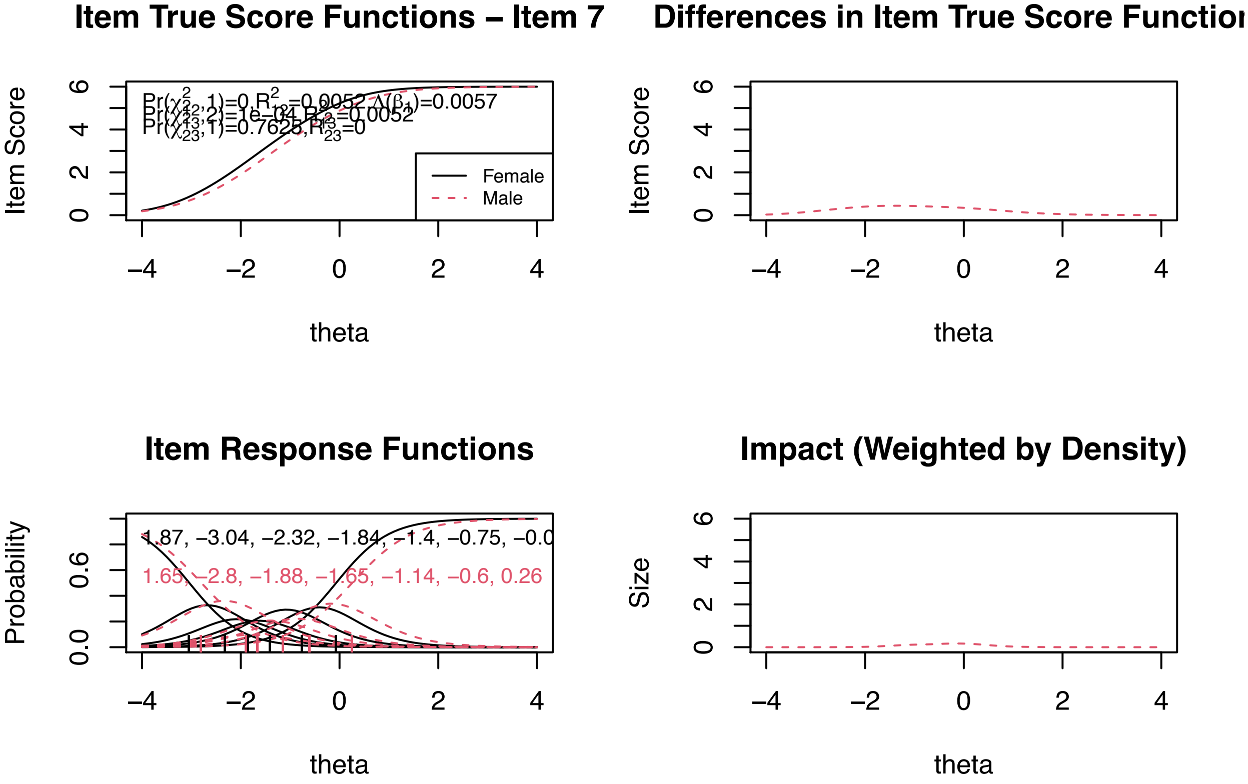
Item True Score Functions – Item 7
Differences in Item True Score Functio
Item Score
theta
Female
Male
Item Response Functions
Probability
1.65, –2.8, –1.88, –1.65, –1.14, –0.6, 0.26
Impact (Weighted by Density)
Size

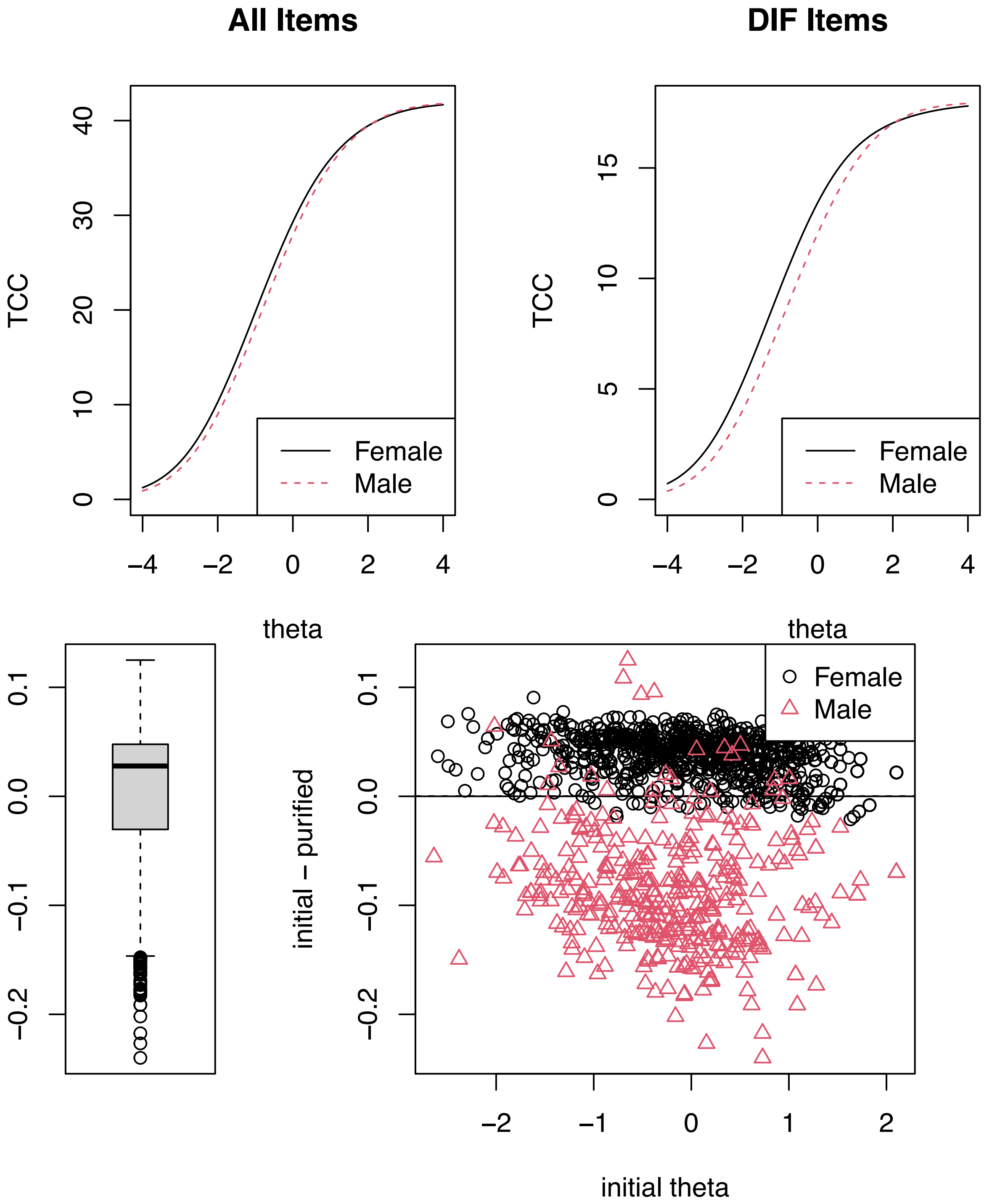

Sample-based DIF: Connectedness

```
## Call:
## lordif::lordif(resp.data = as.data.frame(sample.data), group = clinYN)
##
##   Number of DIF groups: 2
##
```

```
##   Number of items flagged for DIF: 7 of 7
##
##   Items flagged: 1, 2, 3, 4, 5, 6, 7
##
##   Number of iterations for purification: 2 of 10
##
##   Detection criterion: Chisqr
##
##   Threshold: alpha = 0.01
##
##   item ncat chi12 chi13  chi23
## 1    1    7     0     0 0.0006
## 2    2    7     0     0 0.9808
## 3    3    7     0     0 0.0019
## 4    4    7     0     0 0.0275
## 5    5    7     0     0 0.0012
## 6    6    7     0     0 0.6404
## 7    7    7     0     0 0.0000
```

Back to top

Demoralization

Reliability: Demoralization

```
## Cronbach's alpha is 0.799.
## Mean item-total correlation is 0.441.
## If each item were dropped:
##     raw_alpha std.alpha G6(smc) average_r S/N alpha se  var.r med.r
## Q88      0.75      0.75    0.70      0.42 2.9    0.016 0.0108  0.41
## Q24      0.73      0.73    0.68      0.41 2.7    0.017 0.0071  0.39
## Q36      0.80      0.81    0.76      0.51 4.1    0.013 0.0026  0.51
## Q42      0.75      0.74    0.70      0.42 2.9    0.016 0.0081  0.39
## Q61      0.76      0.76    0.72      0.44 3.2    0.015 0.0137  0.44
```

Unidimensionality: Demoralization

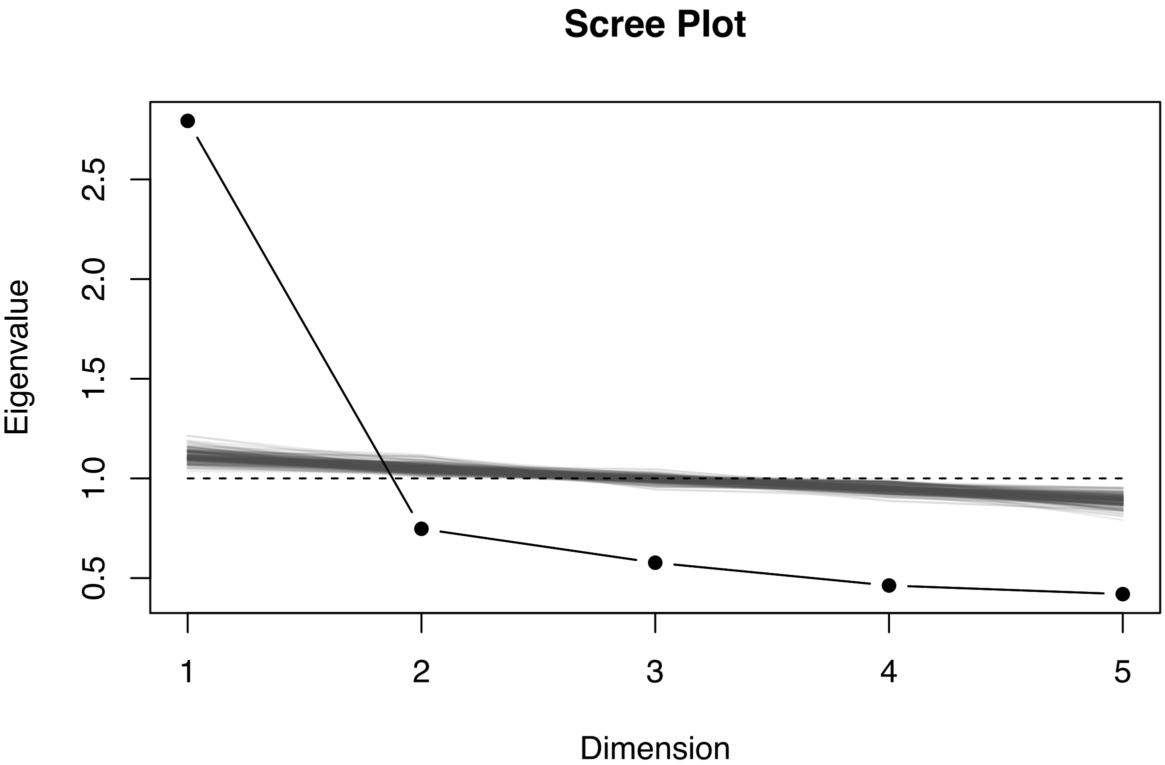


```
## [1] "Ratio of first to second eigenvalues: 3.739"

## [1] 2.7934518 0.7472057 0.5770869 0.4626998 0.4195558

## Factor Analysis using method =  minres
## Call: fa(r = grm_obj$X)
## Standardized loadings (pattern matrix) based upon correlation matrix
##      MR1   h2   u2 com
## Q88 0.71 0.51 0.49   1
## Q24 0.78 0.60 0.40   1
## Q36 0.48 0.23 0.77   1
## Q42 0.72 0.52 0.48   1
## Q61 0.65 0.42 0.58   1
##
##                 MR1
## SS loadings    2.28
## Proportion Var 0.46
##
## Mean item complexity =  1
## Test of the hypothesis that 1 factor is sufficient.
##
## The degrees of freedom for the null model are  10  and the objective function was  1.45 with Chi Squa
## The degrees of freedom for the model are 5  and the objective function was  0.01
##
## The root mean square of the residuals (RMSR) is  0.02
## The df corrected root mean square of the residuals is  0.03
##
## The harmonic number of observations is  598 with the empirical chi square  4.45  with prob <  0.49
```

```
## The total number of observations was  619  with Likelihood Chi Square =  6.66  with prob <  0.25
##
## Tucker Lewis Index of factoring reliability =  0.996
## RMSEA index =  0.023  and the 90 % confidence intervals are  0 0.064
## BIC =  -25.48
## Fit based upon off diagonal values = 1
## Measures of factor score adequacy
##                                                    MR1
## Correlation of (regression) scores with factors   0.91
## Multiple R square of scores with factors          0.82
## Minimum correlation of possible factor scores     0.65
```

Graded-Response Model: Demoralization

```
##     Extrmt1 Extrmt2 Extrmt3 Extrmt4 Extrmt5 Extrmt6 Dscrmn
## Q88  -1.045  -0.594  -0.253   0.150   0.592   1.028  1.991
## Q24  -1.091  -0.472  -0.030   0.406   0.874   1.476  2.439
## Q36  -0.399   0.406   0.937   1.660   2.416   3.251  1.116
## Q42  -1.555  -0.860  -0.439  -0.039   0.495   1.099  2.046
## Q61  -0.376   0.164   0.640   1.168   1.754   2.268  1.890
```

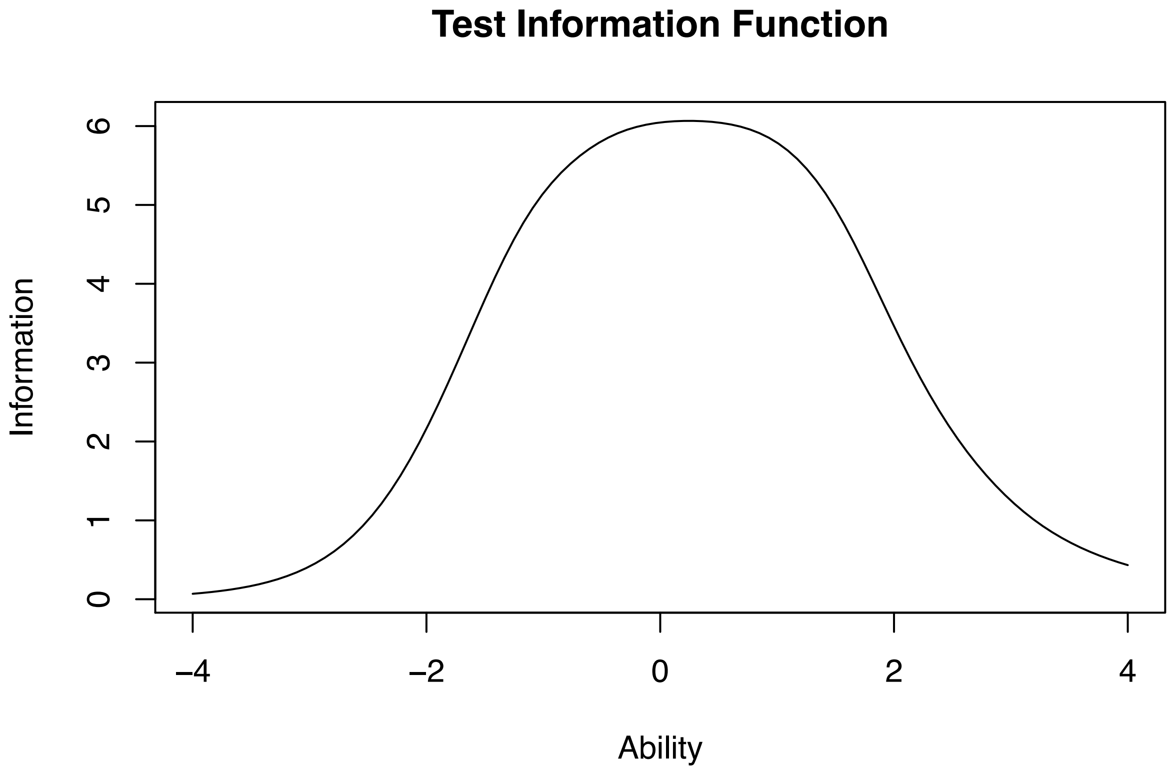

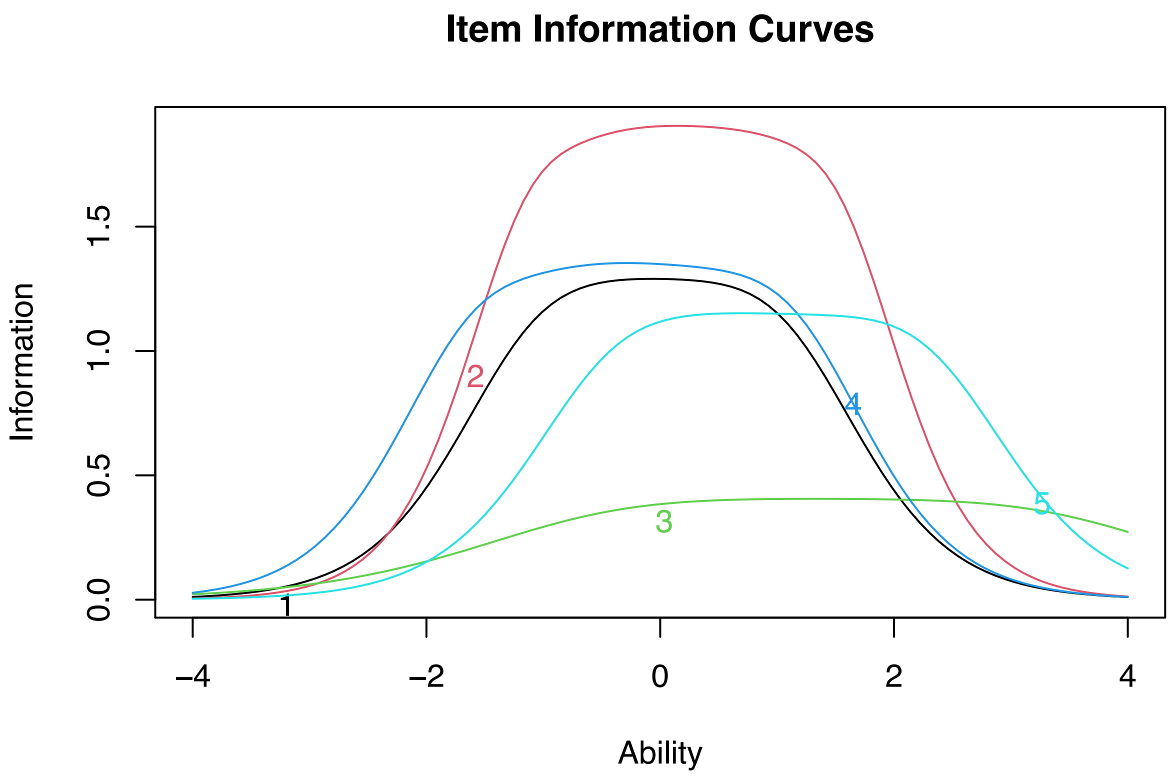

Gender-based DIF: Demoralization

```
## Call:
## lordif::lordif(resp.data = as.data.frame(sex.data), group = sex)
##
##   Number of DIF groups: 2
##
##   Number of items flagged for DIF: 1 of 5
##
##   Items flagged: 2
##
##   Number of iterations for purification: 3 of 10
##
##   Detection criterion: Chisqr
##
##   Threshold: alpha = 0.01
##
##   item ncat  chi12  chi13  chi23
## 1    1    7 0.8571 0.9625 0.8336
## 2    2    7 0.0000 0.0000 0.7581
## 3    3    7 0.8880 0.7918 0.5038
## 4    4    7 0.3452 0.5849 0.6699
## 5    5    7 0.0560 0.1005 0.3319
```

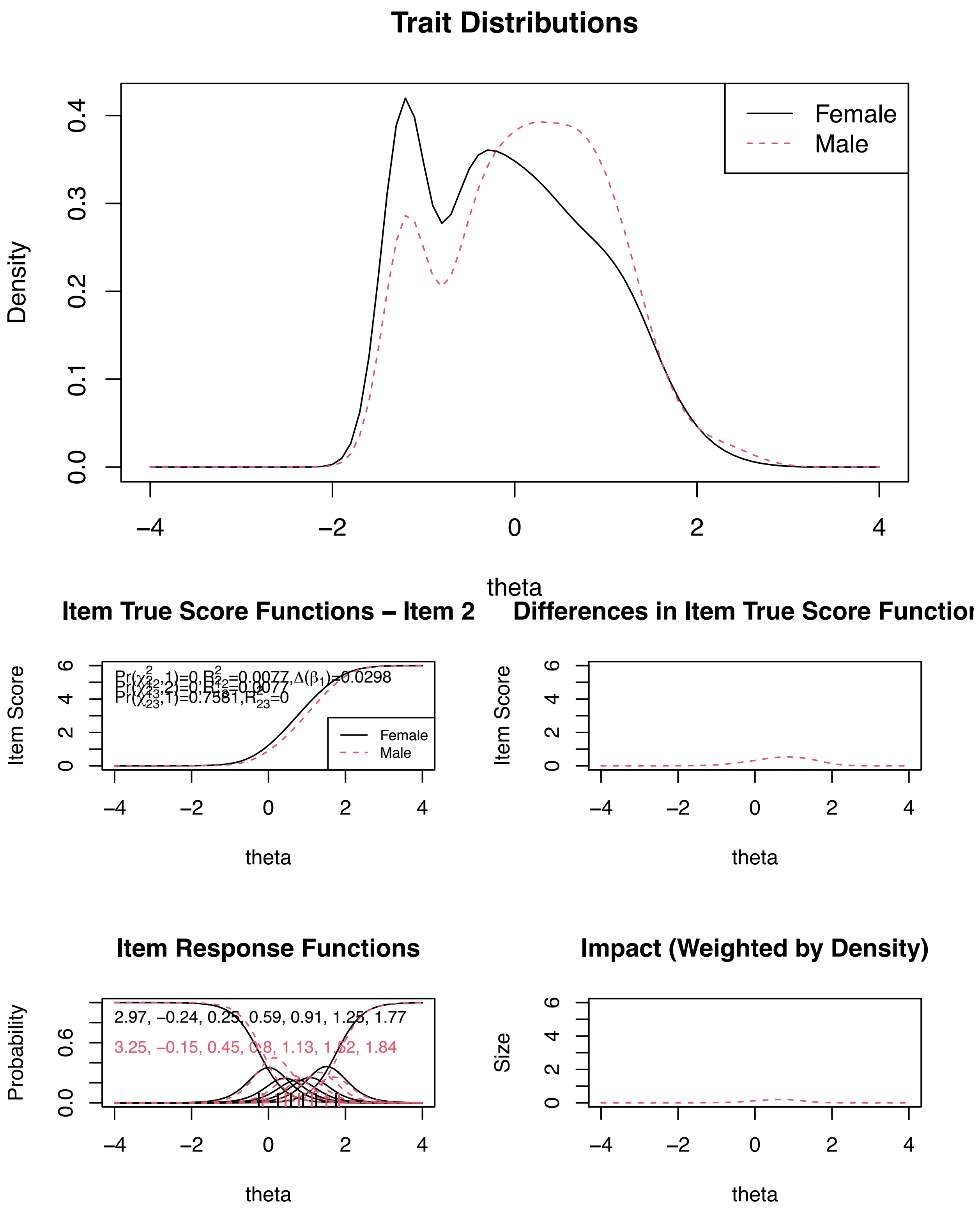
Trait Distributions
Density
theta
Female
Male
Item True Score Functions – Item 2
Differences in Item True Score Functio
Item Score
Item Response Functions
2.97, −0.24, 0.25, 0.59, 0.91, 1.25, 1.77
3.25, −0.15, 0.45, 0.8, 1.13, 1.52, 1.84
Probability
Impact (Weighted by Density)
Size

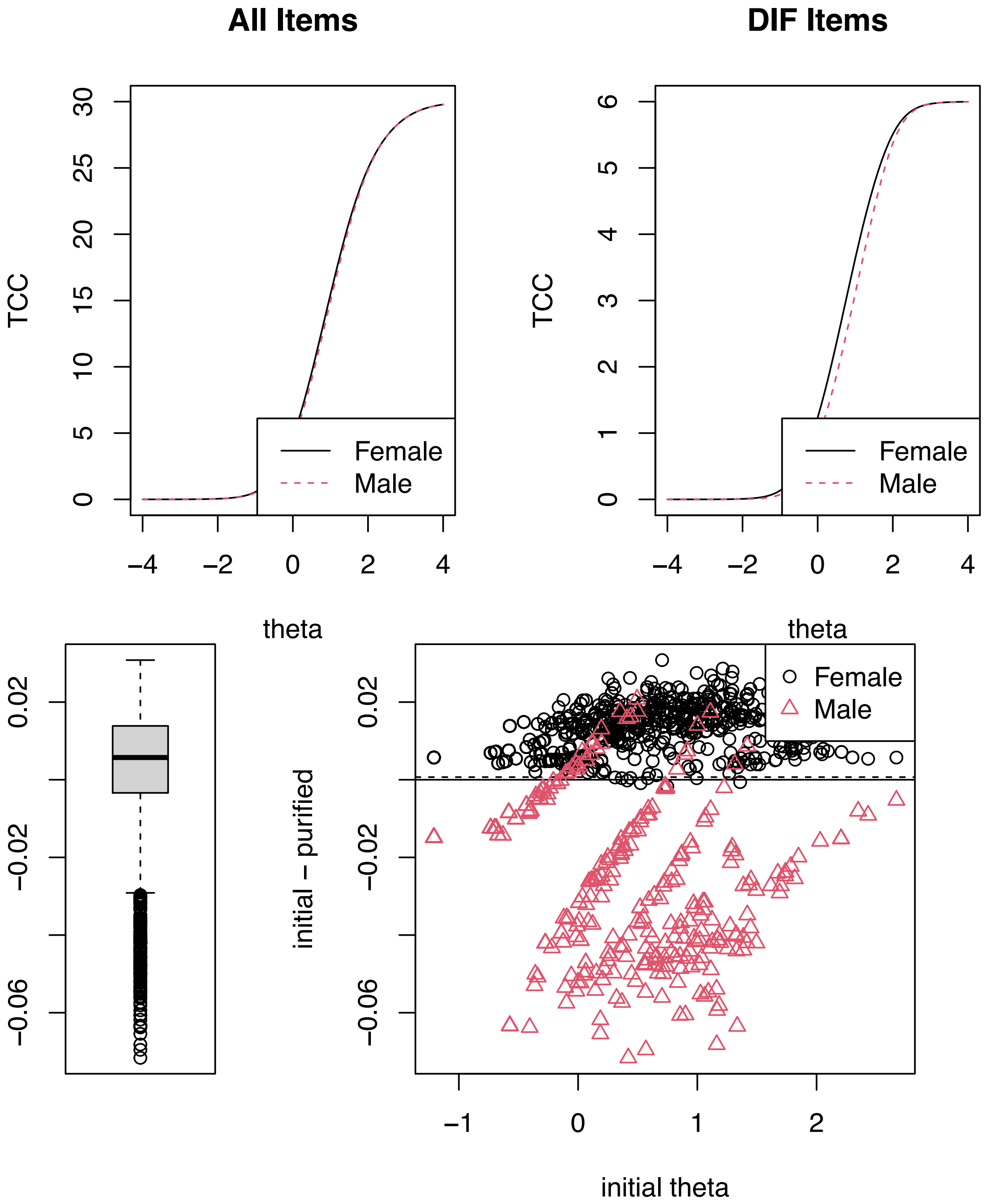

Sample-based DIF: Demoralization

```
## Call:
## lordif::lordif(resp.data = as.data.frame(sample.data), group = clinYN)
##
##   Number of DIF groups: 2
##
```

```
##   Number of items flagged for DIF: 5 of 5
##
##   Items flagged: 1, 2, 3, 4, 5
##
##   Number of iterations for purification: 4 of 10
##
##   Detection criterion: Chisqr
##
##   Threshold: alpha = 0.01
##
##   item ncat chi12 chi13  chi23
## 1    1    7     0     0 0.0000
## 2    2    7     0     0 0.0996
## 3    3    6     0     0 0.0000
## 4    4    7     0     0 0.0001
## 5    5    7     0     0 0.9601
```

Back to top

Eating Problems

Reliability: Eating Problems

```
## Cronbach's alpha is 0.751.
## Mean item-total correlation is 0.365.
## If each item were dropped:
##     raw_alpha std.alpha G6(smc) average_r S/N alpha se var.r med.r
## Q63      0.69      0.72    0.71      0.34 2.6    0.020 0.037  0.27
## Q18      0.68      0.70    0.68      0.32 2.3    0.020 0.027  0.26
## Q46      0.66      0.69    0.68      0.31 2.2    0.022 0.030  0.26
## Q47      0.80      0.81    0.79      0.46 4.3    0.013 0.024  0.49
## Q57      0.71      0.73    0.72      0.35 2.7    0.019 0.040  0.28
## Q86      0.75      0.78    0.77      0.41 3.5    0.016 0.044  0.49
```

Unidimensionality: Eating Problems

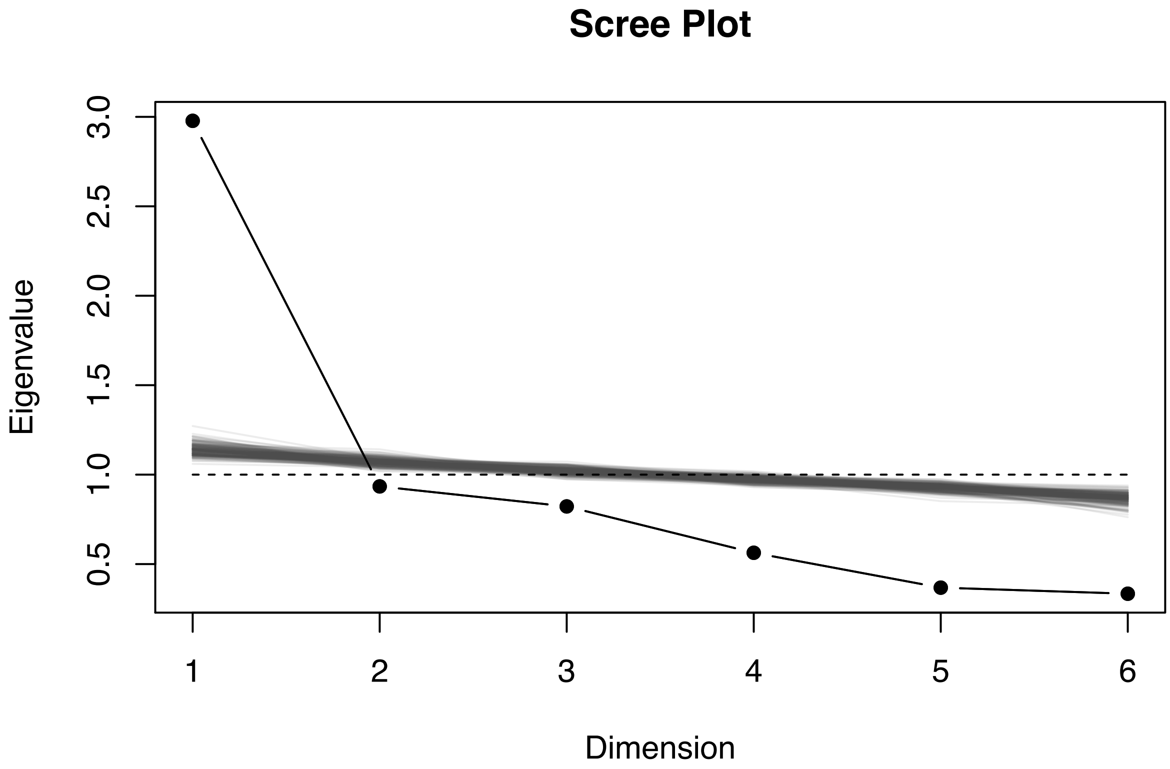


```
## [1] "Ratio of first to second eigenvalues: 3.187"

## [1] 2.9776746 0.9342068 0.8223128 0.5632239 0.3682961 0.3342858

## Factor Analysis using method =  minres
## Call: fa(r = grm_obj$X)
## Standardized loadings (pattern matrix) based upon correlation matrix
##      MR1   h2   u2 com
## Q63 0.71 0.51 0.49   1
## Q18 0.82 0.67 0.33   1
## Q46 0.83 0.69 0.31   1
## Q47 0.24 0.06 0.94   1
## Q57 0.66 0.44 0.56   1
## Q86 0.39 0.16 0.84   1
##
##                 MR1
## SS loadings    2.52
## Proportion Var 0.42
##
## Mean item complexity =  1
## Test of the hypothesis that 1 factor is sufficient.
##
## The degrees of freedom for the null model are  15  and the objective function was  1.84 with Chi Squa
## The degrees of freedom for the model are 9  and the objective function was  0.04
##
## The root mean square of the residuals (RMSR) is  0.03
## The df corrected root mean square of the residuals is  0.04
##
```

```
## The harmonic number of observations is  595 with the empirical chi square  13.27  with prob <  0.15
## The total number of observations was  619  with Likelihood Chi Square =  25.12  with prob <  0.0028
##
## Tucker Lewis Index of factoring reliability =  0.976
## RMSEA index =  0.054  and the 90 % confidence intervals are  0.029 0.079
## BIC =  -32.73
## Fit based upon off diagonal values = 1
## Measures of factor score adequacy
##                                                    MR1
## Correlation of (regression) scores with factors   0.93
## Multiple R square of scores with factors          0.86
## Minimum correlation of possible factor scores     0.73
```

Graded-Response Model: Eating Problems

```
##     Extrmt1 Extrmt2 Extrmt3 Extrmt4 Extrmt5 Extrmt6 Dscrmn
## Q63  -0.323   0.167   0.556   0.954   1.261   1.655  2.565
## Q18   0.666   1.116   1.380   1.634   1.782   2.056  3.708
## Q46   0.216   0.682   0.874   1.091   1.303   1.532  3.543
## Q47  -1.224   0.057   0.737   1.700   2.578   3.842  0.531
## Q57   0.860   1.254   1.486   1.685   1.869   2.169  2.710
## Q86  -2.243  -1.367  -0.724  -0.006   0.728   1.573  1.003
```

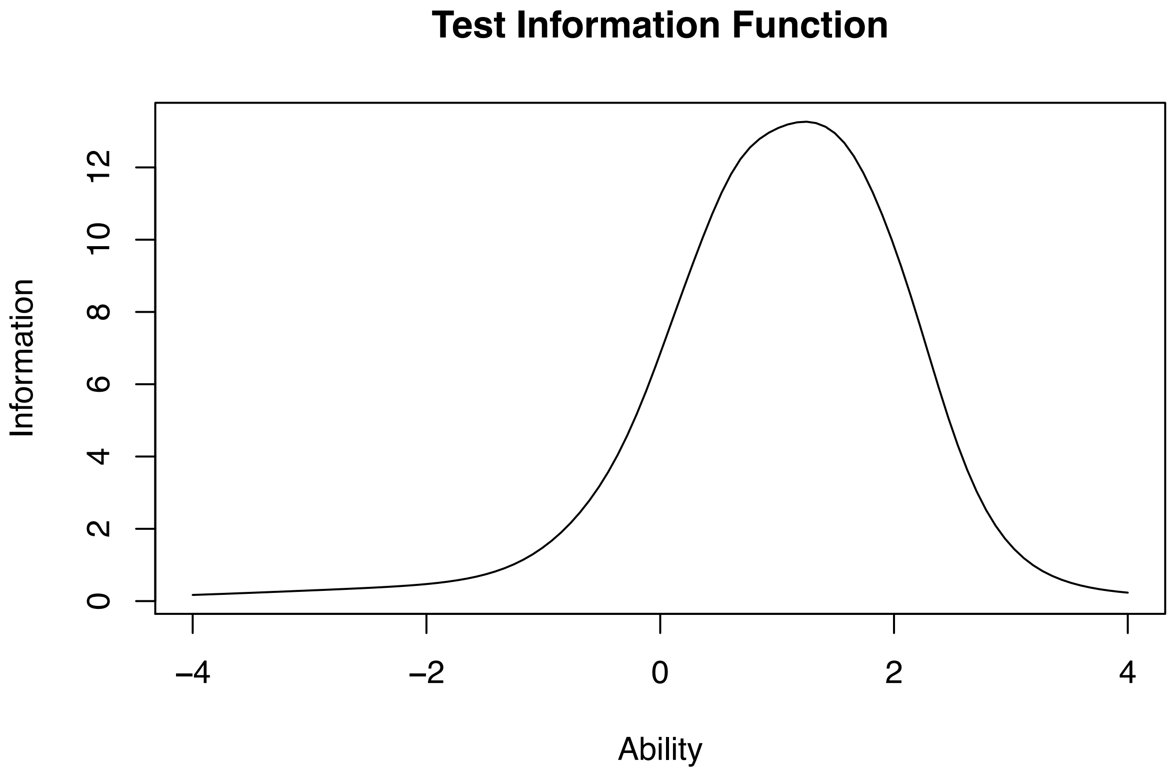

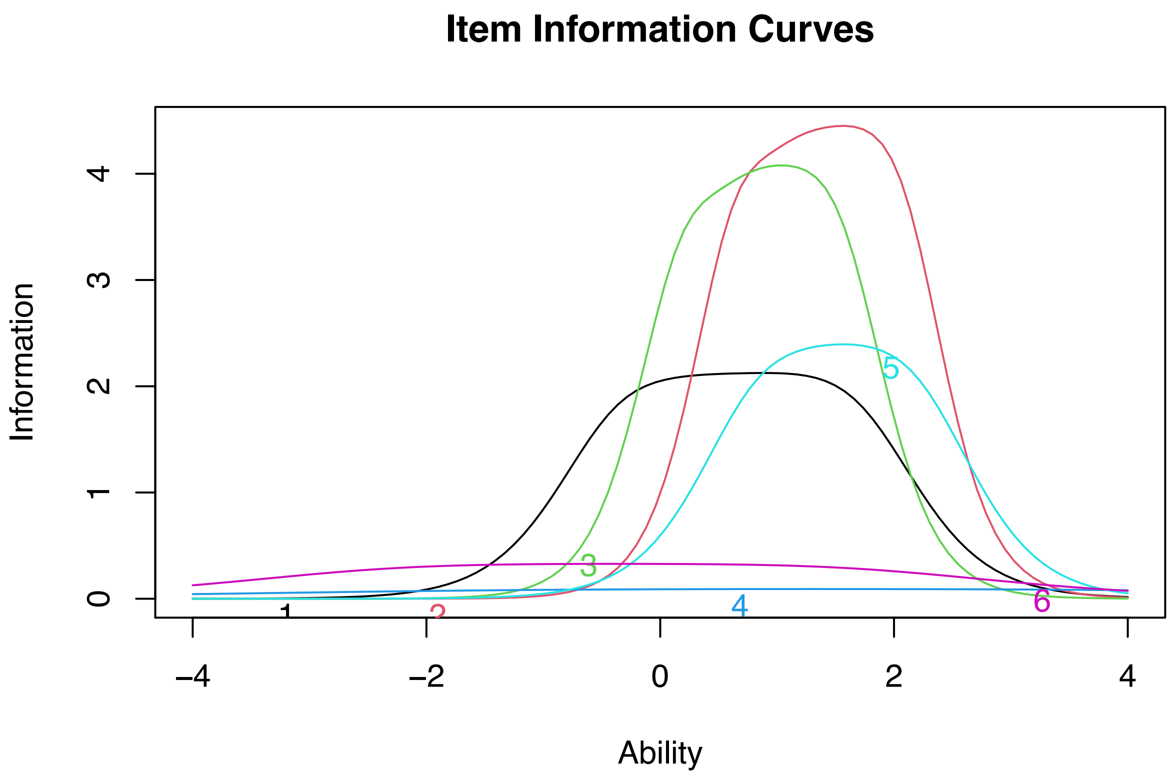

Gender-based DIF: Eating Problems

```
## Call:
## lordif::lordif(resp.data = as.data.frame(sex.data), group = sex)
##
##   Number of DIF groups: 2
##
##   Number of items flagged for DIF: 2 of 6
##
##   Items flagged: 5, 6
##
##   Number of iterations for purification: 2 of 10
##
##   Detection criterion: Chisqr
##
##   Threshold: alpha = 0.01
##
##   item ncat  chi12  chi13  chi23
## 1    1    7 0.1350 0.2649 0.5156
## 2    2    5 0.0183 0.0413 0.3703
## 3    3    7 0.9834 0.9395 0.7244
## 4    4    7 0.7325 0.7573 0.5076
## 5    5    5 0.0022 0.0003 0.0085
## 6    6    7 0.0008 0.0000 0.0029
```

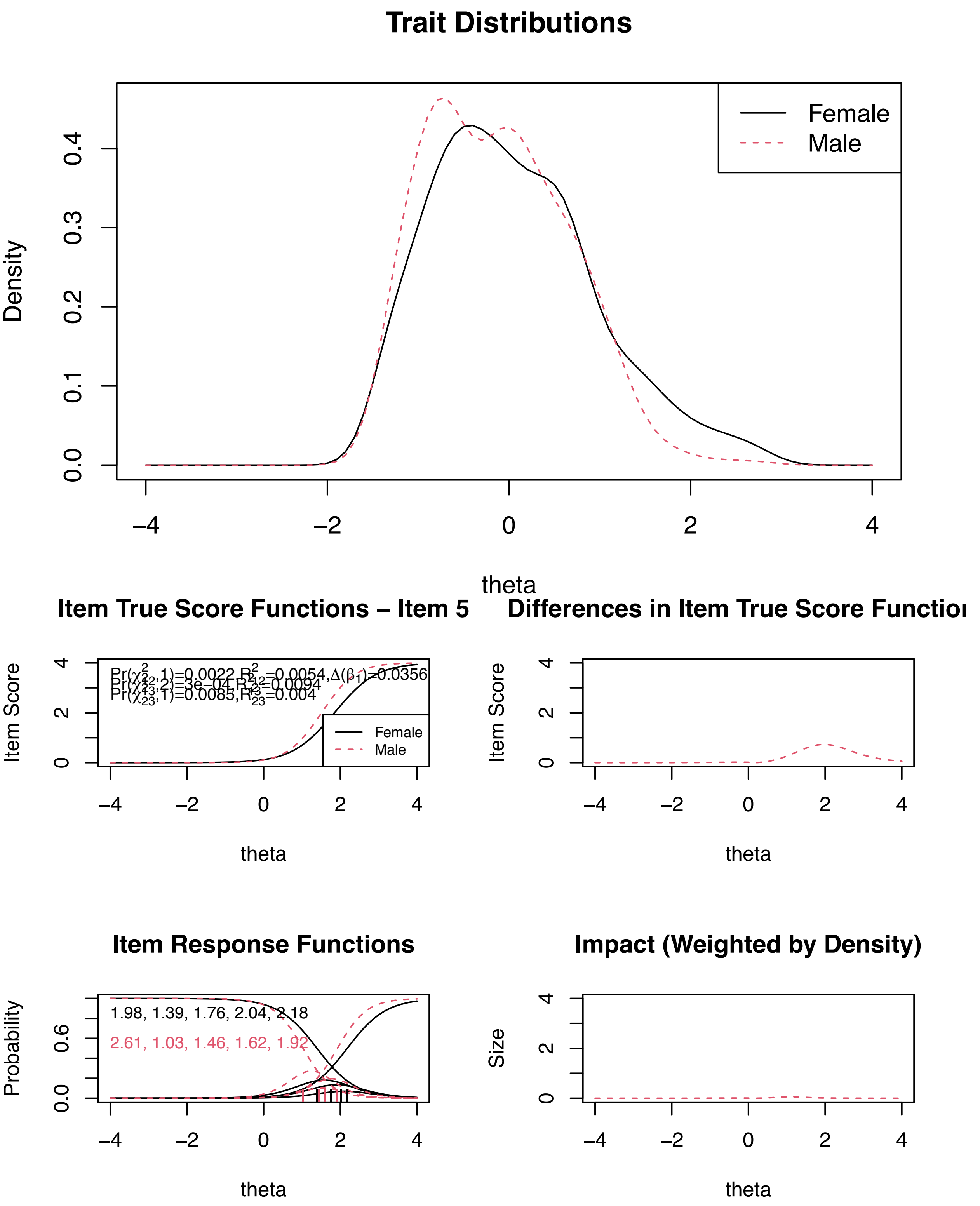
Trait Distributions
Density
theta
Female
Male
Item True Score Functions – Item 5
Item Score
theta
Female
Male
Differences in Item True Score Functio
Item Score
theta
Item Response Functions
Probability
1.98, 1.39, 1.76, 2.04, 2.18
2.61, 1.03, 1.46, 1.62, 1.92
theta
Impact (Weighted by Density)
Size
theta

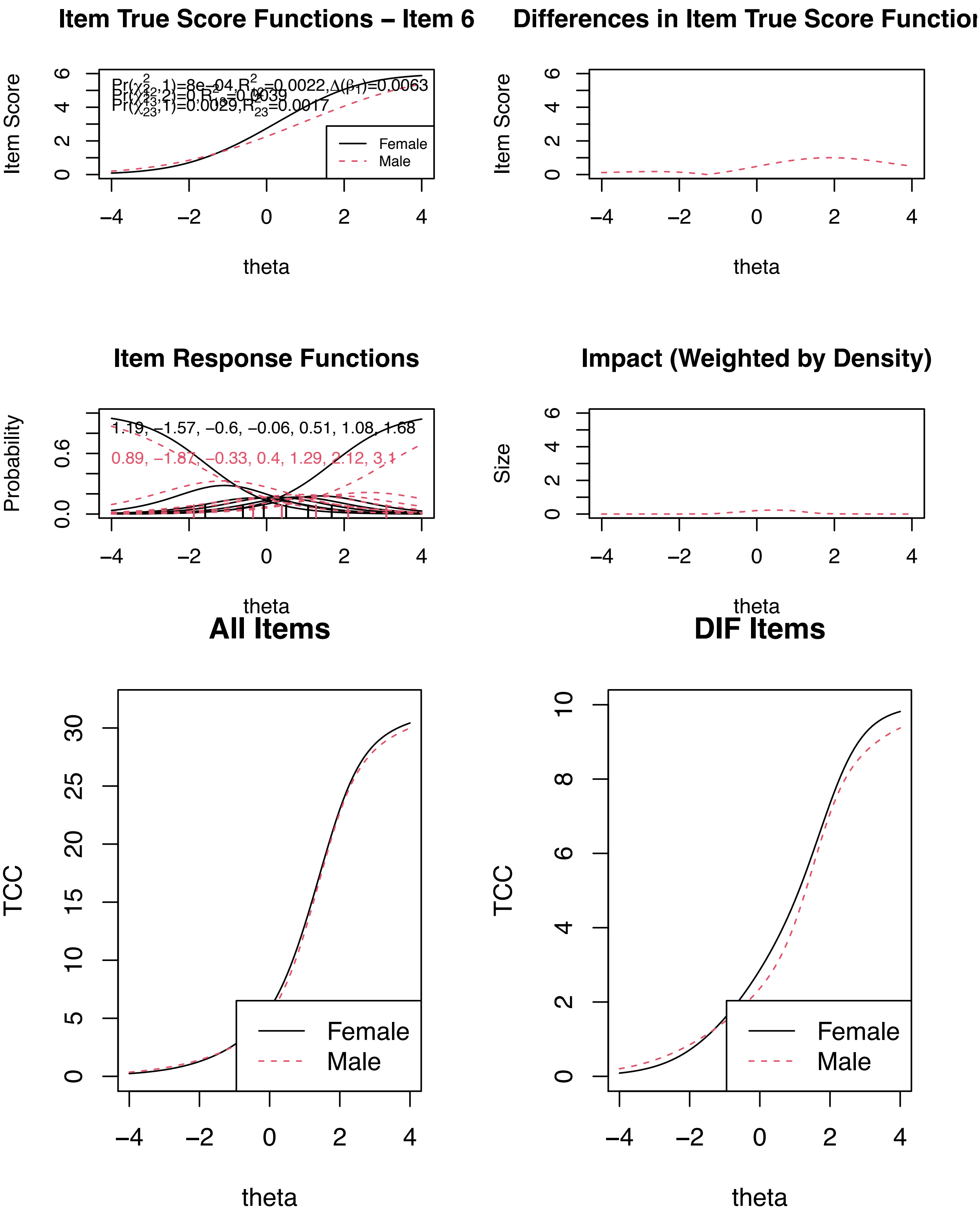
Item True Score Functions – Item 6
Item Score
theta
Female
Male
Differences in Item True Score Functio
Item Score
theta
Item Response Functions
Probability
theta
0.89, −1.87, −0.33, 0.4, 1.29, 2.12, 3.1
Impact (Weighted by Density)
Size
theta
All Items
TCC
theta
Female
Male
DIF Items
TCC
theta
Female
Male

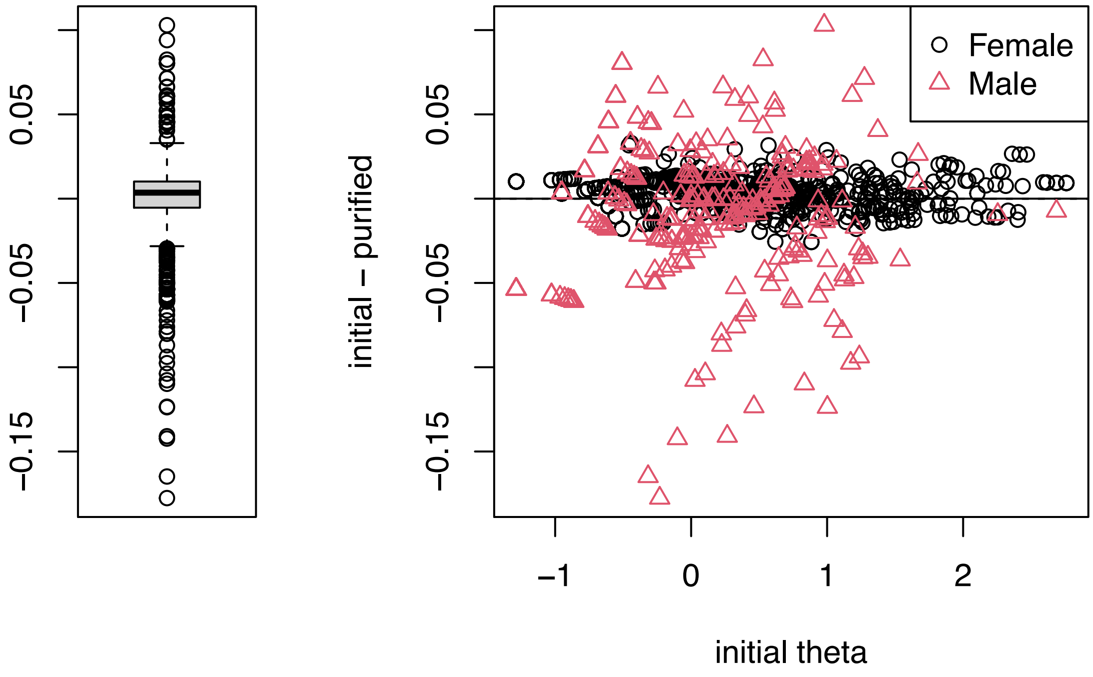

Sample-based DIF: Eating Problems

```
## Call:
## lordif::lordif(resp.data = as.data.frame(sample.data), group = clinYN)
##
##   Number of DIF groups: 2
##
##   Number of items flagged for DIF: 6 of 6
##
##   Items flagged: 1, 2, 3, 4, 5, 6
##
##   Number of iterations for purification: 2 of 10
##
##   Detection criterion: Chisqr
##
##   Threshold: alpha = 0.01
##
##   item ncat  chi12 chi13  chi23
## 1    1    7 0.0000 0e+00 0.0278
## 2    2    6 0.7208 0e+00 0.0000
## 3    3    7 0.0000 0e+00 0.0189
## 4    4    7 0.0000 0e+00 0.0035
## 5    5    6 0.0001 3e-04 0.7931
## 6    6    7 0.0000 0e+00 0.0070
```

Back to top

Hypervigilance

Reliability: Hypervigilance

```
## Cronbach's alpha is 0.502.
## Mean item-total correlation is 0.199.
## If each item were dropped:
##      raw_alpha std.alpha G6(smc) average_r  S/N alpha se  var.r med.r
## Q2        0.40      0.40    0.32      0.18 0.68    0.036 0.0024  0.17
## Q7        0.30      0.30    0.23      0.13 0.43    0.043 0.0015  0.12
## Q9-       0.51      0.51    0.44      0.25 1.03    0.029 0.0290  0.24
## Q50-      0.48      0.48    0.41      0.23 0.91    0.032 0.0302  0.15
```

Unidimensionality: Hypervigilance

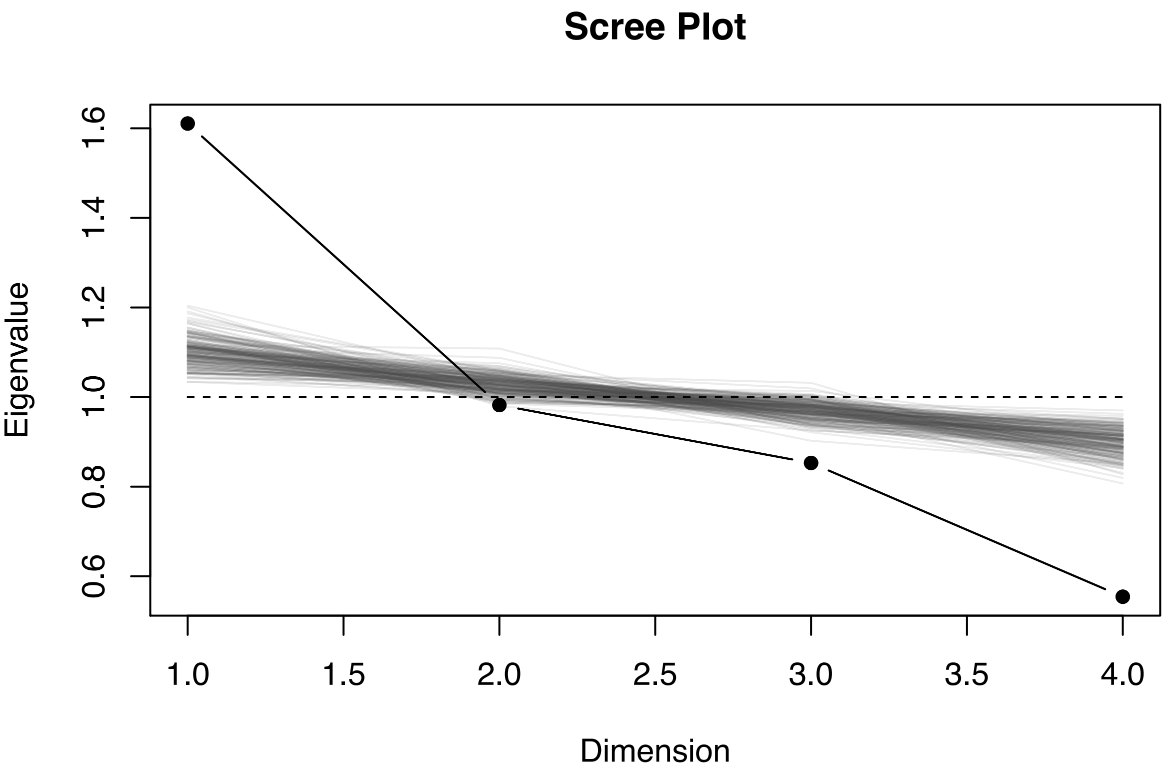


```
## [1] "Ratio of first to second eigenvalues: 1.64"

## [1] 1.6106444 0.9821730 0.8529011 0.5542815

## Factor Analysis using method =  minres
## Call: fa(r = grm_obj$X)
## Standardized loadings (pattern matrix) based upon correlation matrix
##       MR1    h2   u2 com
## Q2   0.50 0.254 0.75   1
## Q7   0.81 0.661 0.34   1
## Q9  -0.22 0.050 0.95   1
## Q50 -0.27 0.074 0.93   1
##
##                 MR1
## SS loadings    1.04
```

```
## Proportion Var 0.26
##
## Mean item complexity =  1
## Test of the hypothesis that 1 factor is sufficient.
##
## The degrees of freedom for the null model are  6  and the objective function was  0.29 with Chi Squa
## The degrees of freedom for the model are 2  and the objective function was  0.02
##
## The root mean square of the residuals (RMSR) is  0.04
## The df corrected root mean square of the residuals is  0.08
##
## The harmonic number of observations is  526 with the empirical chi square  12.71  with prob <  0.001
## The total number of observations was  814  with Likelihood Chi Square =  13.6  with prob <  0.0011
##
## Tucker Lewis Index of factoring reliability =  0.848
## RMSEA index =  0.084  and the 90 % confidence intervals are  0.046 0.129
## BIC =  0.19
## Fit based upon off diagonal values = 0.96
## Measures of factor score adequacy
##                                                   MR1
## Correlation of (regression) scores with factors   0.84
## Multiple R square of scores with factors          0.71
## Minimum correlation of possible factor scores     0.42
```

Graded-Response Model: Hypervigilance

```
##     Extrmt1 Extrmt2 Extrmt3 Extrmt4 Extrmt5 Extrmt6 Dscrmn
## Q2   -1.170  -0.297   0.325   0.912   1.596   2.307  1.271
## Q7   -1.662  -0.946  -0.551  -0.045   0.600   1.299  2.311
## Q9    7.802   5.107   3.237   2.268   0.712  -1.297 -0.443
## Q50   2.410   0.586  -0.980  -2.933  -4.620  -7.070 -0.542
```

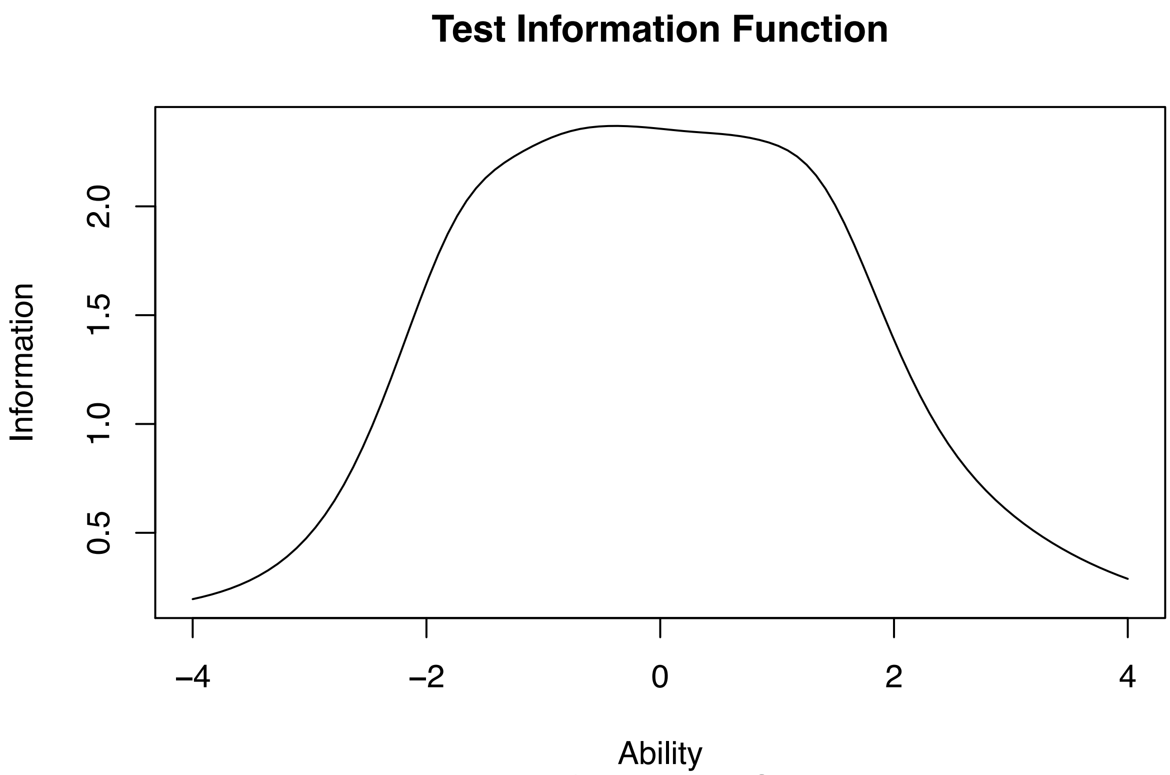

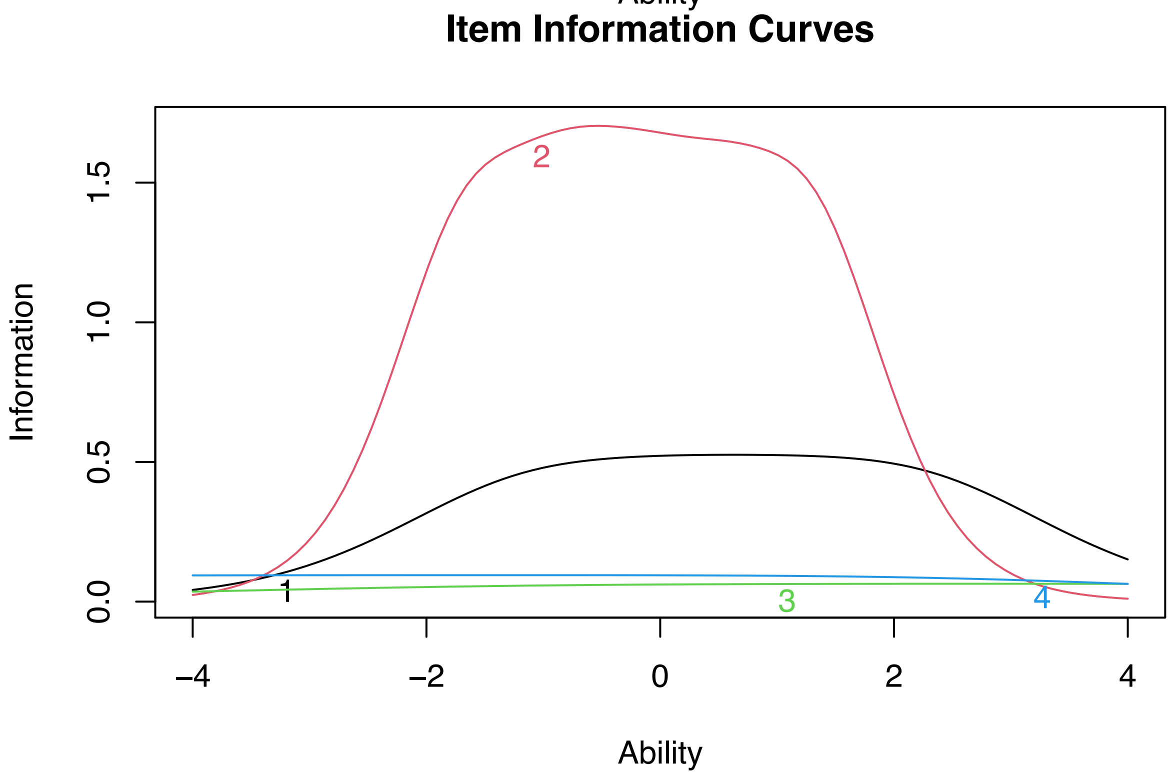

Gender-based DIF: Hypervigilance

```
## Call:
## lordif::lordif(resp.data = as.data.frame(sex.data), group = sex)
```

```
##
##   Number of DIF groups: 2
##
##   Number of items flagged for DIF: 0 of 4
##
##   Items flagged:
##
##   Number of iterations for purification: 1 of 10
##
##   Detection criterion: Chisqr
##
##   Threshold: alpha = 0.01
```

Sample-based DIF: Hypervigilance

```
## Call:
## lordif::lordif(resp.data = as.data.frame(sample.data), group = clinYN)
##
##   Number of DIF groups: 2
##
##   Number of items flagged for DIF: 4 of 4
##
##   Items flagged: 1, 2, 3, 4
##
##   Number of iterations for purification: 1 of 10
##
##   Detection criterion: Chisqr
##
##   Threshold: alpha = 0.01
##
##   item ncat  chi12  chi13  chi23
## 1    1    7 0.0002 0.0007 0.3568
## 2    2    7 0.0011 0.0025 0.2490
## 3    3    6 0.0000 0.0000 0.0001
## 4    4    7 0.0000 0.0000 0.4669
```

Back to top

Pressure from Negative Affect

Reliability: Pressure from Negative Affect

```
## Cronbach's alpha is 0.762.
## Mean item-total correlation is 0.266.
## If each item were dropped:
##     raw_alpha std.alpha G6(smc) average_r S/N alpha se var.r med.r
## Q28      0.74      0.75    0.75      0.27 2.9    0.015 0.024  0.21
## Q21      0.76      0.76    0.76      0.29 3.2    0.014 0.023  0.23
## Q30      0.76      0.77    0.77      0.29 3.3    0.014 0.022  0.24
## Q32      0.76      0.77    0.77      0.29 3.3    0.014 0.022  0.24
## Q33      0.76      0.76    0.76      0.29 3.2    0.014 0.022  0.22
## Q42      0.71      0.71    0.71      0.24 2.5    0.017 0.013  0.21
## Q51      0.72      0.72    0.72      0.24 2.6    0.017 0.017  0.21
```

```
## Q60      0.72      0.72    0.71      0.24 2.6    0.016 0.014  0.21
## Q88      0.71      0.72    0.72      0.24 2.5    0.017 0.018  0.21
```

Unidimensionality: Pressure from Negative Affect

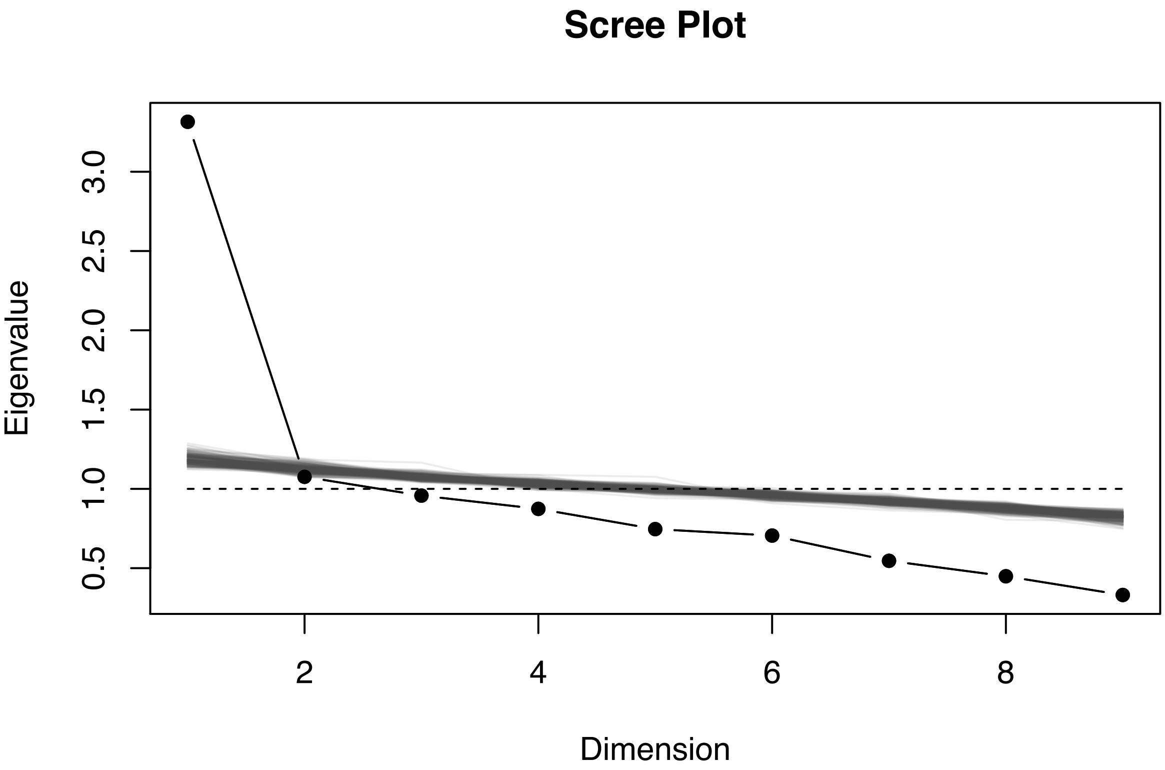


```
## [1] "Ratio of first to second eigenvalues: 3.081"

## [1] 3.3149321 1.0759714 0.9575043 0.8739044 0.7461750 0.7054858 0.5463449
## [8] 0.4489102 0.3307720

## Factor Analysis using method =  minres
## Call: fa(r = grm_obj$X)
## Standardized loadings (pattern matrix) based upon correlation matrix
##      MR1    h2   u2 com
## Q28 0.47 0.217 0.78   1
## Q21 0.33 0.107 0.89   1
## Q30 0.30 0.091 0.91   1
## Q32 0.30 0.091 0.91   1
## Q33 0.36 0.127 0.87   1
## Q42 0.78 0.612 0.39   1
## Q51 0.70 0.488 0.51   1
## Q60 0.72 0.515 0.49   1
## Q88 0.71 0.498 0.50   1
##
##                 MR1
## SS loadings    2.75
## Proportion Var 0.31
##
## Mean item complexity =  1
## Test of the hypothesis that 1 factor is sufficient.
```

```
##
## The degrees of freedom for the null model are  36  and the objective function was  2.06 with Chi Squa
## The degrees of freedom for the model are 27  and the objective function was  0.18
##
## The root mean square of the residuals (RMSR) is  0.05
## The df corrected root mean square of the residuals is  0.06
##
## The harmonic number of observations is  596 with the empirical chi square  100.86  with prob <  1.9e
## The total number of observations was  619  with Likelihood Chi Square =  107.41  with prob <  1.5e-1
##
## Tucker Lewis Index of factoring reliability =  0.913
## RMSEA index =  0.069  and the 90 % confidence intervals are  0.056 0.083
## BIC =  -66.15
## Fit based upon off diagonal values = 0.97
## Measures of factor score adequacy
##                                                    MR1
## Correlation of (regression) scores with factors   0.92
## Multiple R square of scores with factors          0.84
## Minimum correlation of possible factor scores     0.68
```

Graded-Response Model: Pressure from Negative Affect

```
##     Extrmt1 Extrmt2 Extrmt3 Extrmt4 Extrmt5 Extrmt6 Dscrmn
## Q28  -0.888  -0.079   0.405   0.964   1.460   2.286  1.012
## Q21  -0.821   0.517   1.144   1.904   2.699   3.756  0.597
## Q30  -1.704  -0.432   0.642   1.685   2.946   4.047  0.565
## Q32   1.857   2.400   2.642   2.878   3.022   3.325  0.861
## Q33  -3.680  -2.399  -1.641  -0.695   0.496   1.656  0.728
## Q42  -1.452  -0.815  -0.423  -0.043   0.460   1.014  2.611
## Q51  -1.853  -1.332  -0.854  -0.345   0.261   0.865  1.934
## Q60  -2.524  -1.833  -1.017  -0.143   0.675   1.441  2.178
## Q88  -1.098  -0.625  -0.268   0.156   0.618   1.080  1.788
```

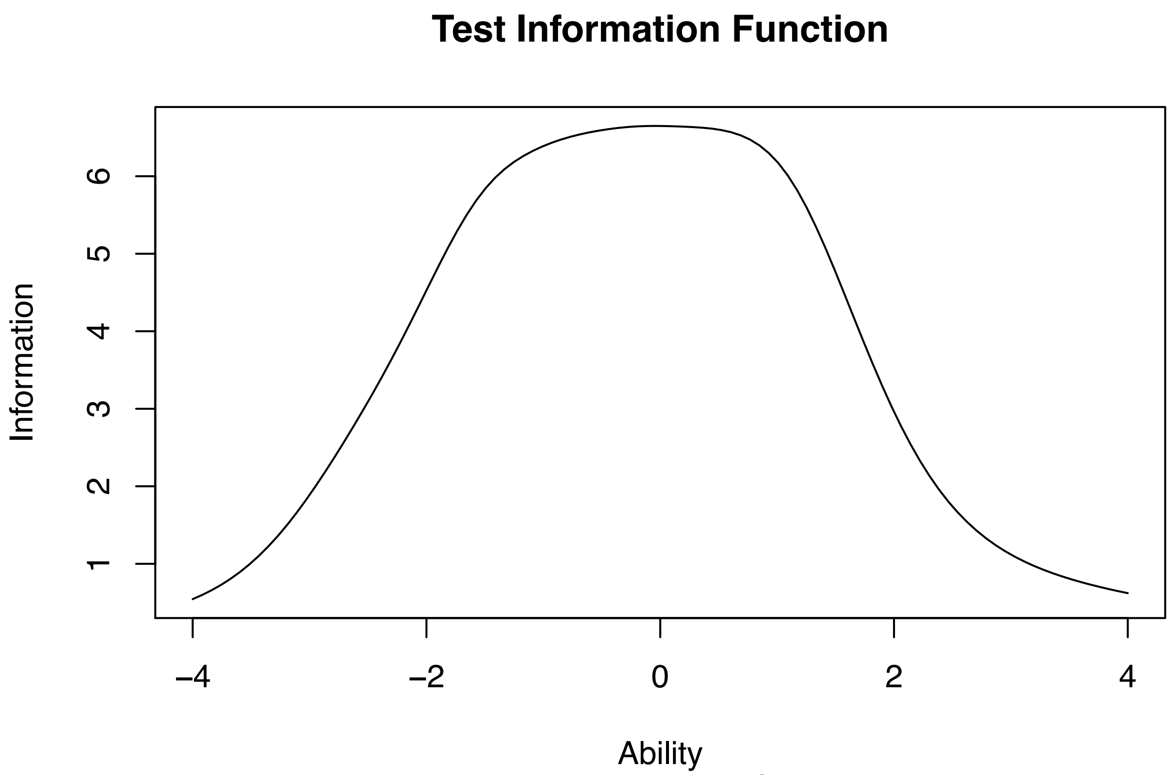

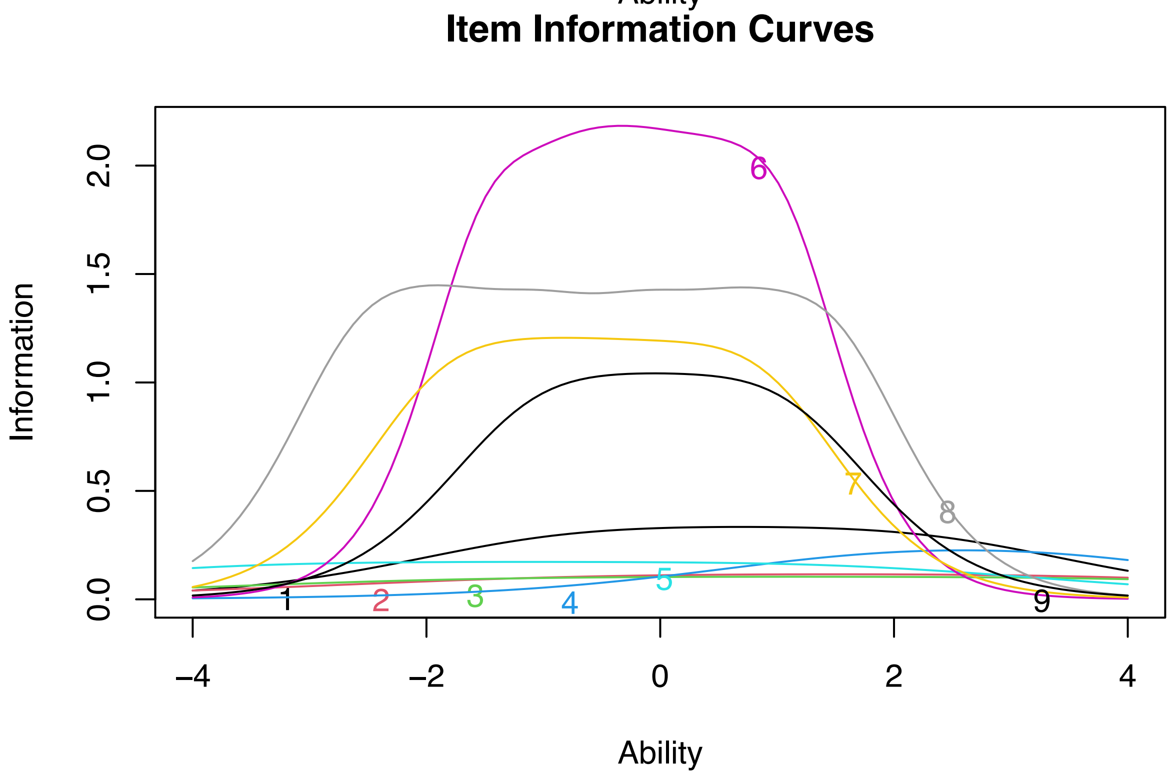

Gender-based DIF: Pressure from Negative Affect

```
## Call:
## lordif::lordif(resp.data = as.data.frame(sex.data), group = sex)
```

```
##
##   Number of DIF groups: 2
##
##   Number of items flagged for DIF: 1 of 9
##
##   Items flagged: 1
##
##   Number of iterations for purification: 2 of 10
##
##   Detection criterion: Chisqr
##
##   Threshold: alpha = 0.01
##
##   item ncat  chi12  chi13  chi23
## 1    1    7 0.0000 0.0000 0.4929
## 2    2    7 0.8050 0.7915 0.5236
## 3    3    7 0.6081 0.8446 0.7844
## 4    4    3 0.7143 0.6104 0.3556
## 5    5    7 0.1627 0.1935 0.2478
## 6    6    7 0.7395 0.8586 0.6593
## 7    7    7 0.6815 0.1484 0.0562
## 8    8    7 0.7710 0.4137 0.1949
## 9    9    7 0.2798 0.3582 0.3467
```

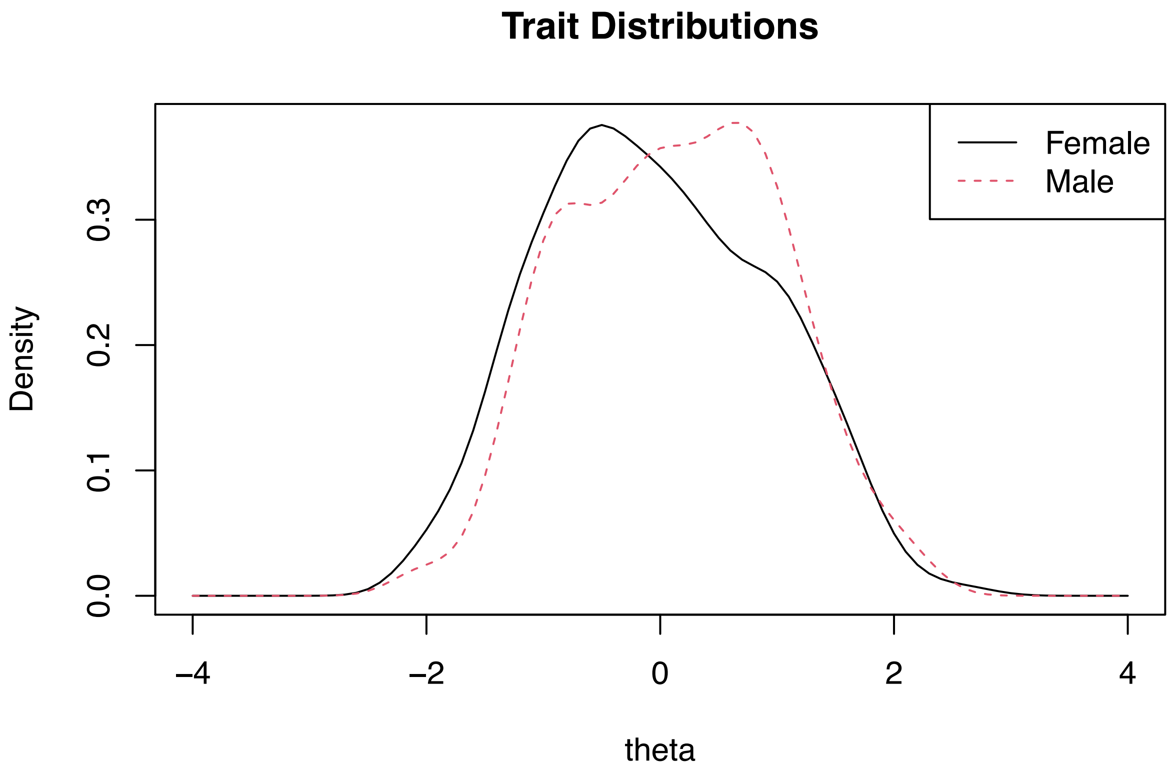

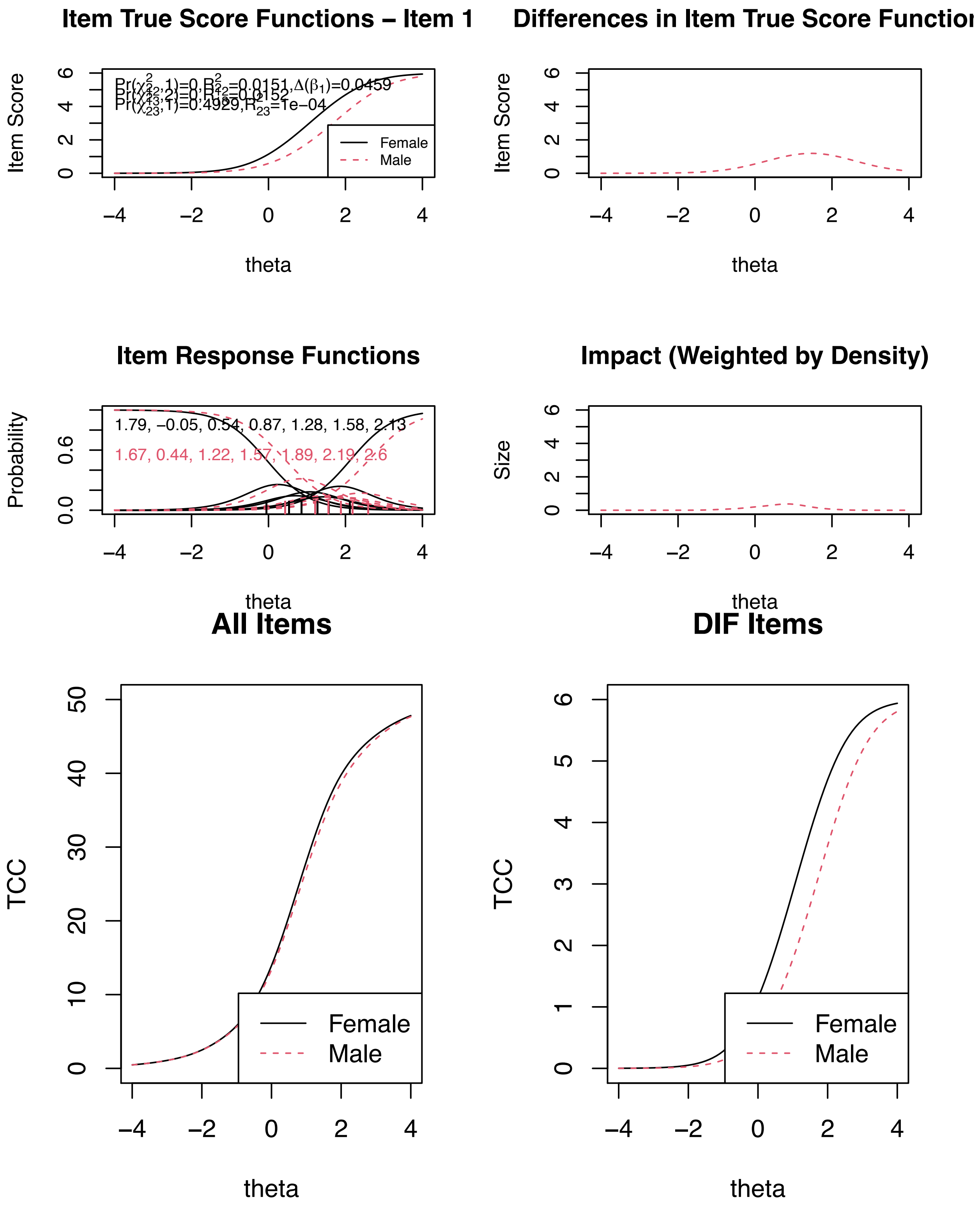

Item True Score Functions – Item 1
Differences in Item True Score Functions
Item Response Functions
Impact (Weighted by Density)
All Items
DIF Items
Item Score
Probability
Size
TCC
theta
Female
Male
1.79, –0.05, 0.54, 0.87, 1.28, 1.58, 2.13
1.67, 0.44, 1.22, 1.57, 1.89, 2.19, 2.6

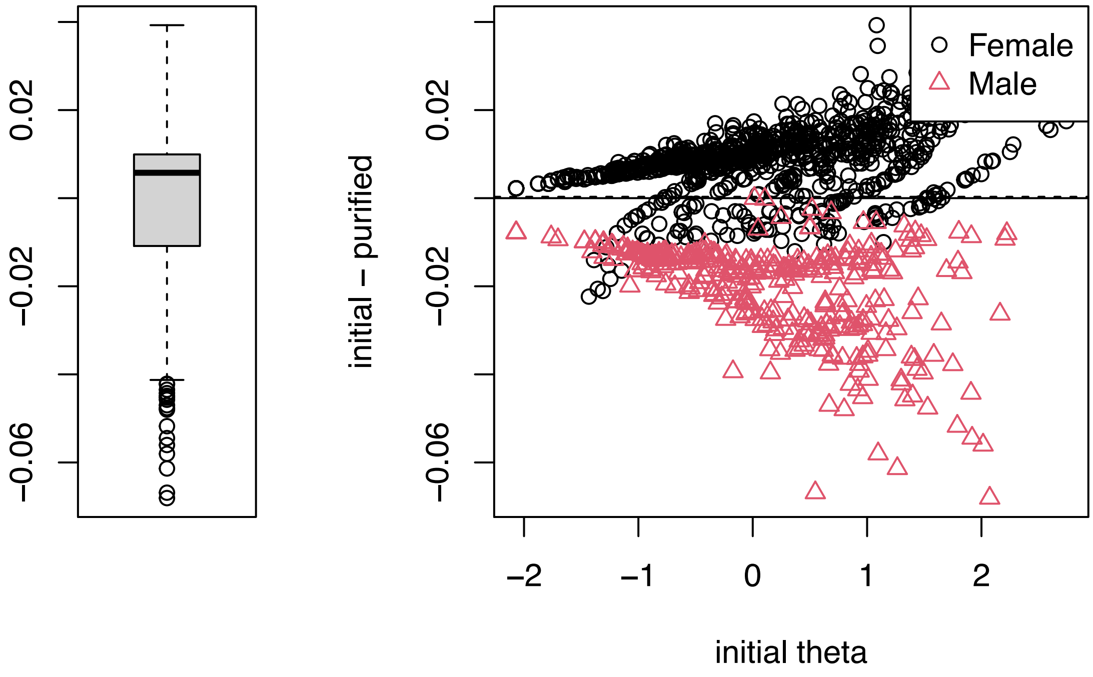

Sample-based DIF: Pressure from Negative Affect

```
## Call:
## lordif::lordif(resp.data = as.data.frame(sample.data), group = clinYN)
##
##   Number of DIF groups: 2
##
##   Number of items flagged for DIF: 9 of 9
##
##   Items flagged: 1, 2, 3, 4, 5, 6, 7, 8, 9
##
##   Number of iterations for purification: 4 of 10
##
##   Detection criterion: Chisqr
##
##   Threshold: alpha = 0.01
##
##   item ncat chi12 chi13  chi23
## 1    1    7     0     0 0.0141
## 2    2    7     0     0 0.8857
## 3    3    7     0     0 0.1536
## 4    4    3     0     0 0.0949
## 5    5    7     0     0 0.6517
## 6    6    7     0     0 0.1544
## 7    7    7     0     0 0.0503
## 8    8    7     0     0 0.0151
## 9    9    7     0     0 0.0069
```

Back to top

Perfectionism

Reliability: Perfectionism

```
## Cronbach's alpha is 0.732.
## Mean item-total correlation is 0.282.
## If each item were dropped:
##     raw_alpha std.alpha G6(smc) average_r S/N alpha se  var.r med.r
## Q20      0.70      0.70    0.68      0.28 2.3    0.016 0.0063  0.26
## Q26      0.68      0.69    0.66      0.27 2.2    0.017 0.0055  0.26
## Q28      0.71      0.71    0.68      0.29 2.5    0.016 0.0056  0.27
## Q38      0.69      0.69    0.67      0.28 2.3    0.017 0.0065  0.26
## Q48      0.71      0.72    0.70      0.30 2.5    0.016 0.0061  0.29
## Q52      0.70      0.69    0.66      0.27 2.3    0.016 0.0033  0.26
## Q68      0.71      0.71    0.68      0.29 2.4    0.016 0.0038  0.29
```

Unidimensionality: Perfectionism

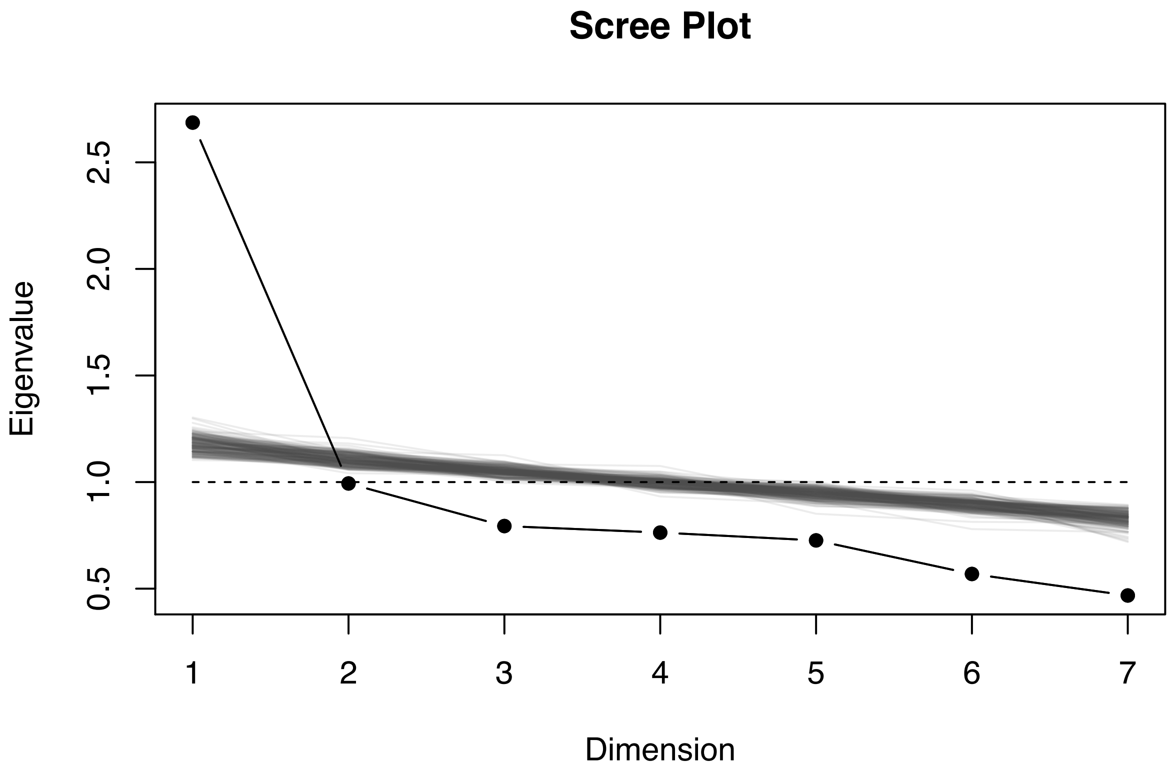


```
## [1] "Ratio of first to second eigenvalues: 2.704"

## [1] 2.6864042 0.9934927 0.7937032 0.7631167 0.7263723 0.5689176 0.4679932

## Factor Analysis using method =  minres
## Call: fa(r = grm_obj$X)
## Standardized loadings (pattern matrix) based upon correlation matrix
##      MR1   h2   u2 com
## Q20 0.53 0.28 0.72   1
## Q26 0.62 0.39 0.61   1
## Q28 0.46 0.21 0.79   1
## Q38 0.57 0.32 0.68   1
```

```
## Q48 0.45 0.21 0.79   1
## Q52 0.57 0.33 0.67   1
## Q68 0.49 0.24 0.76   1
##
##                  MR1
## SS loadings    1.98
## Proportion Var 0.28
##
## Mean item complexity =  1
## Test of the hypothesis that 1 factor is sufficient.
##
## The degrees of freedom for the null model are  21  and the objective function was  1.16 with Chi Squa
## The degrees of freedom for the model are 14  and the objective function was  0.15
##
## The root mean square of the residuals (RMSR) is  0.06
## The df corrected root mean square of the residuals is  0.07
##
## The harmonic number of observations is  458 with the empirical chi square  68.2  with prob <  4.1e-09
## The total number of observations was  814  with Likelihood Chi Square =  122.68  with prob <  1.9e-19
##
## Tucker Lewis Index of factoring reliability =  0.823
## RMSEA index =  0.098  and the 90 % confidence intervals are  0.082 0.114
## BIC =  28.85
## Fit based upon off diagonal values = 0.96
## Measures of factor score adequacy
##                                                    MR1
## Correlation of (regression) scores with factors   0.86
## Multiple R square of scores with factors          0.74
## Minimum correlation of possible factor scores     0.48
```

Graded-Response Model: Perfectionism

```
##     Extrmt1 Extrmt2 Extrmt3 Extrmt4 Extrmt5 Extrmt6 Dscrmn
## Q20  -1.465  -0.536   0.132   0.828   1.586   2.587  1.254
## Q26  -1.282  -0.488  -0.036   0.519   1.228   2.075  1.514
## Q28  -0.760  -0.097   0.467   0.981   1.560   2.469  1.122
## Q38  -1.649  -0.849  -0.264   0.274   0.919   1.781  1.263
## Q48   0.546   1.285   1.688   2.150   2.744   3.236  1.037
## Q52  -2.610  -1.935  -1.416  -0.584   0.209   1.215  1.460
## Q68  -2.779  -1.732  -1.067  -0.487   0.331   1.174  1.184
```

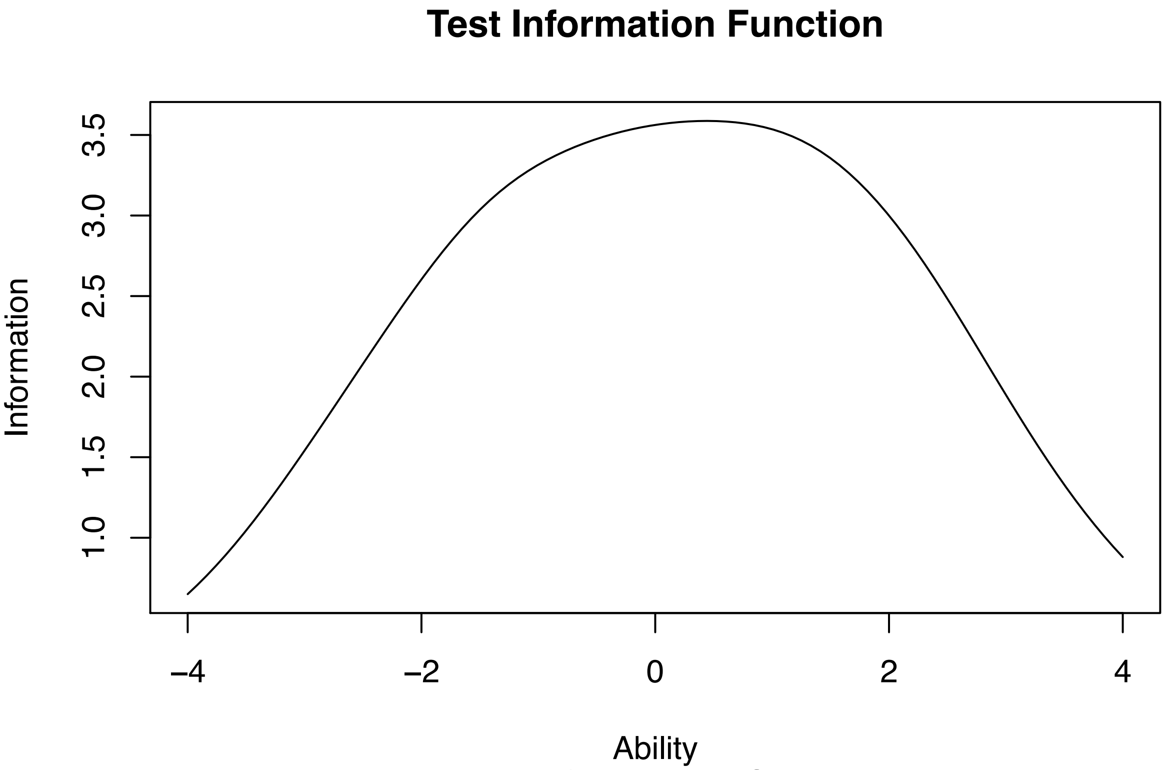

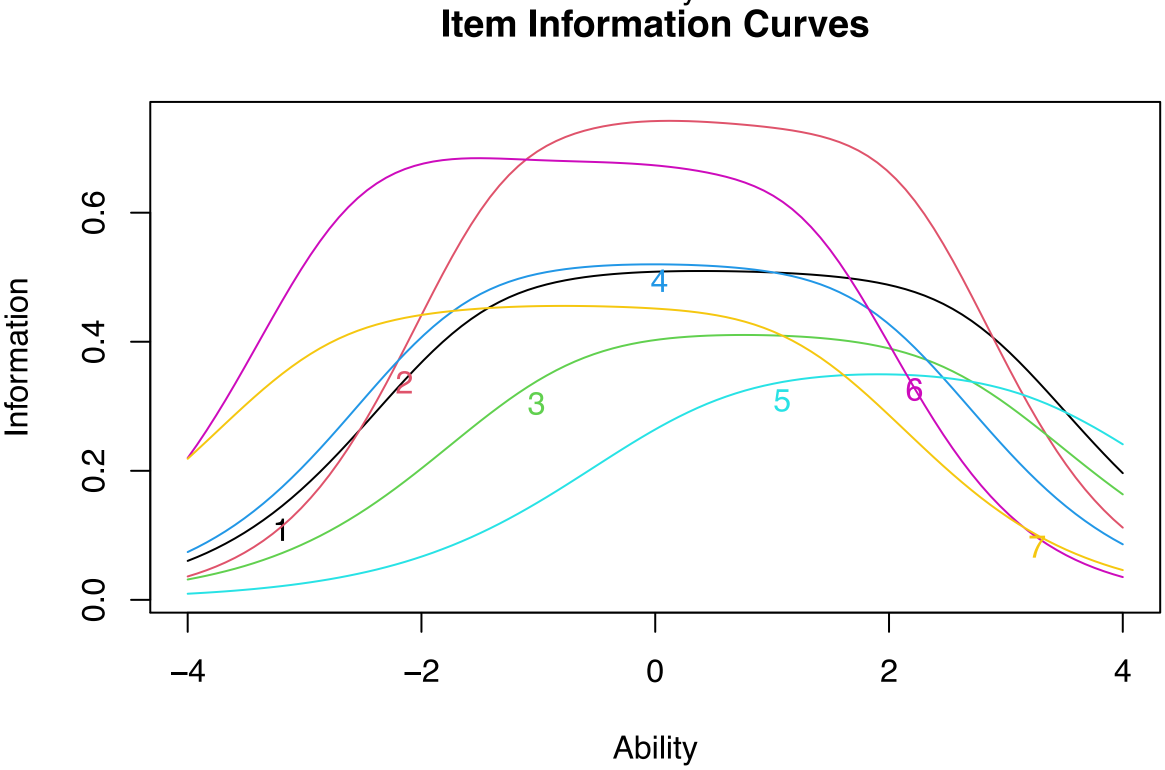

Gender-based DIF: Perfectionism

```
## Call:
## lordif::lordif(resp.data = as.data.frame(sex.data), group = sex)
```

```
##
##   Number of DIF groups: 2
##
##   Number of items flagged for DIF: 2 of 7
##
##   Items flagged: 2, 3
##
##   Number of iterations for purification: 2 of 10
##
##   Detection criterion: Chisqr
##
##   Threshold: alpha = 0.01
##
##   item ncat  chi12  chi13  chi23
## 1    1    7 0.1771 0.3950 0.8489
## 2    2    7 0.0000 0.0001 0.6397
## 3    3    7 0.0000 0.0000 0.2082
## 4    4    7 0.5884 0.2817 0.1344
## 5    5    7 0.0324 0.0789 0.4790
## 6    6    7 0.1503 0.1989 0.2813
## 7    7    7 0.0309 0.0972 0.9456
```

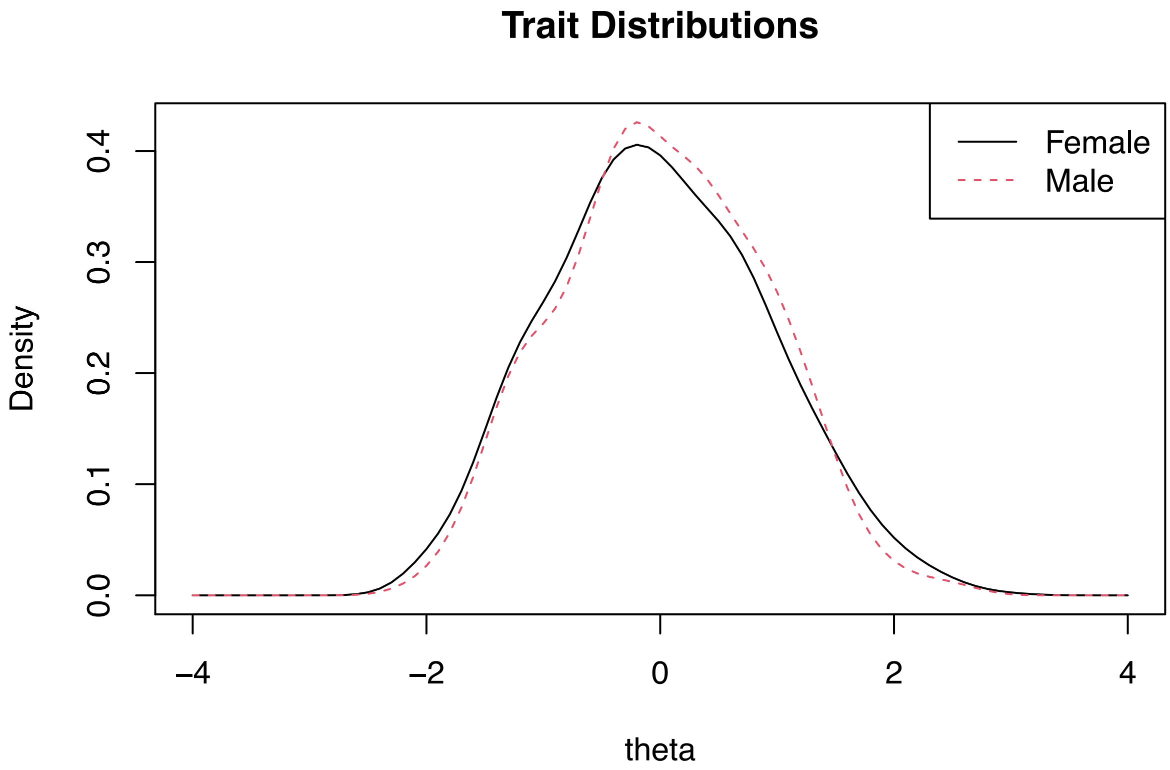

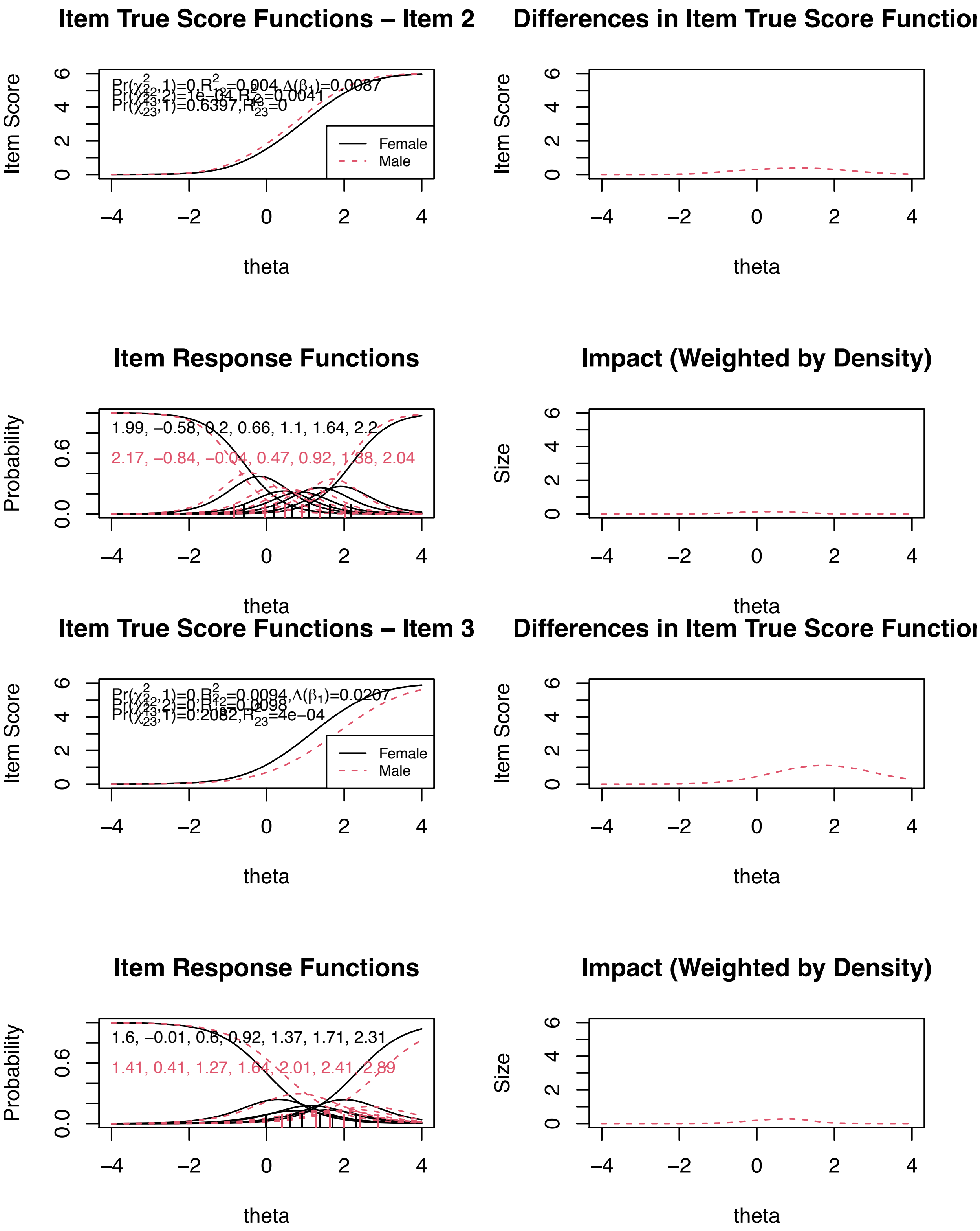
Item True Score Functions – Item 2
Differences in Item True Score Functioı
Item Score
theta
Female
Male
Item Response Functions
Impact (Weighted by Density)
Probability
Size
1.99, –0.58, 0.2, 0.66, 1.1, 1.64, 2.2
2.17, –0.84, –0.04, 0.47, 0.92, 1.38, 2.04
Item True Score Functions – Item 3
1.6, –0.01, 0.6, 0.92, 1.37, 1.71, 2.31
1.41, 0.41, 1.27, 1.64, 2.01, 2.41, 2.89

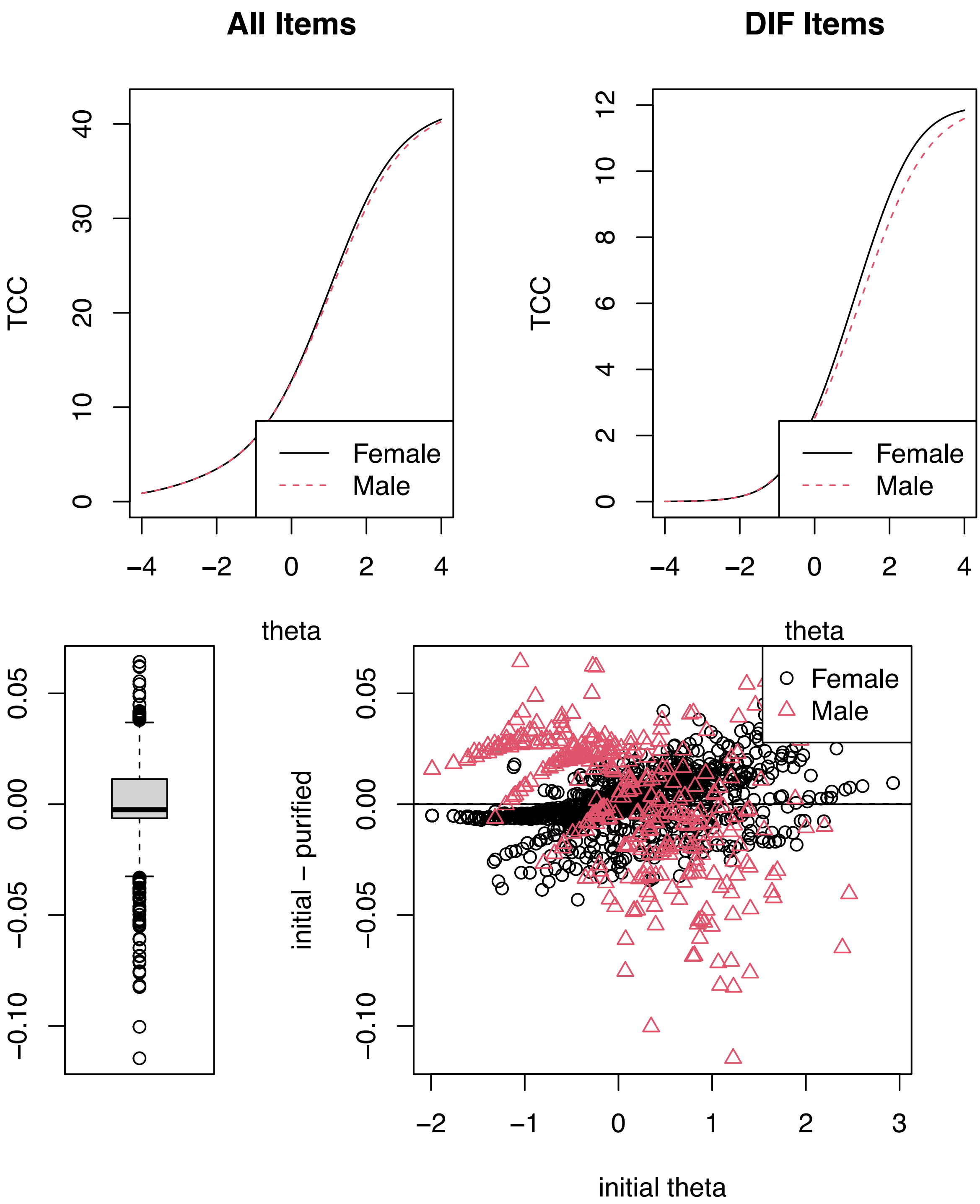

Sample-based DIF: Perfectionism

```
## Call:
## lordif::lordif(resp.data = as.data.frame(sample.data), group = clinYN)
##
##   Number of DIF groups: 2
##
```

```
##    Number of items flagged for DIF: 2 of 7
##
##    Items flagged: 3, 4
##
##    Number of iterations for purification: 3 of 10
##
##    Detection criterion: Chisqr
##
##    Threshold: alpha = 0.01
##
##    item ncat  chi12  chi13  chi23
## 1    1    7 0.2801 0.0341 0.0180
## 2    2    7 0.9059 0.9648 0.8102
## 3    3    7 0.0000 0.0000 0.0000
## 4    4    7 0.0000 0.0000 0.0000
## 5    5    7 0.0179 0.0606 0.9653
## 6    6    7 0.0406 0.0306 0.0954
## 7    7    7 0.0436 0.1056 0.5156
```

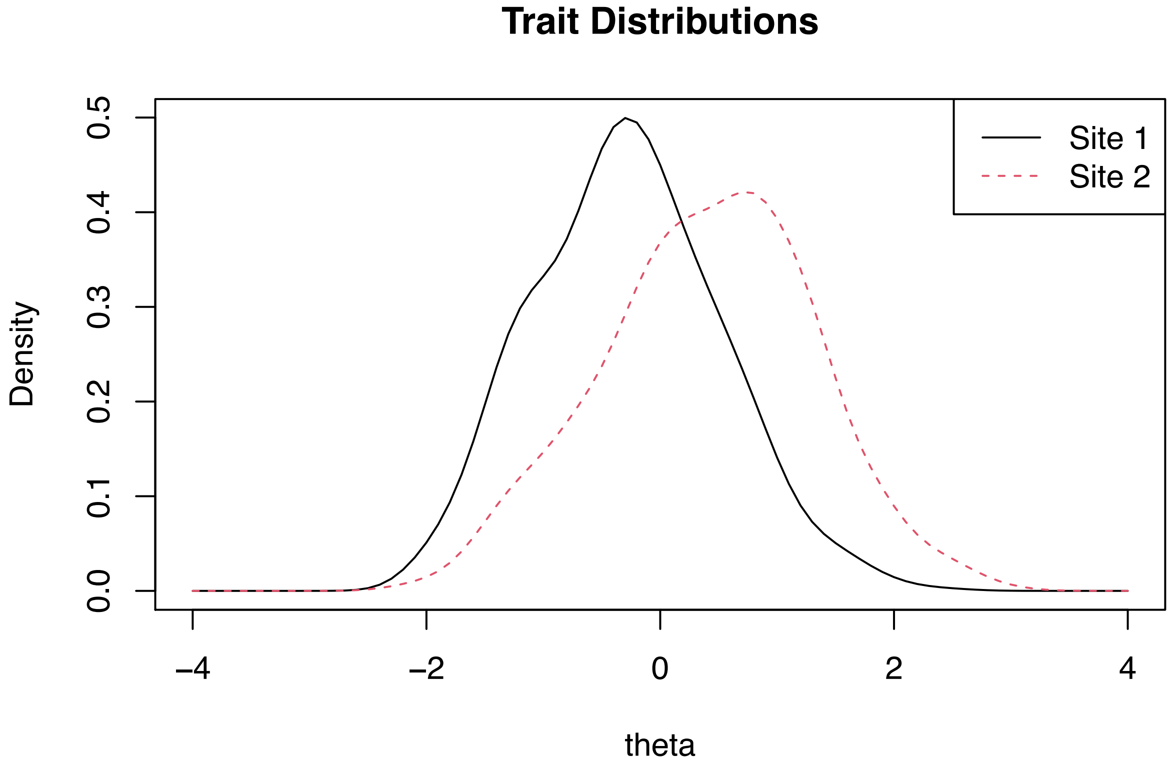

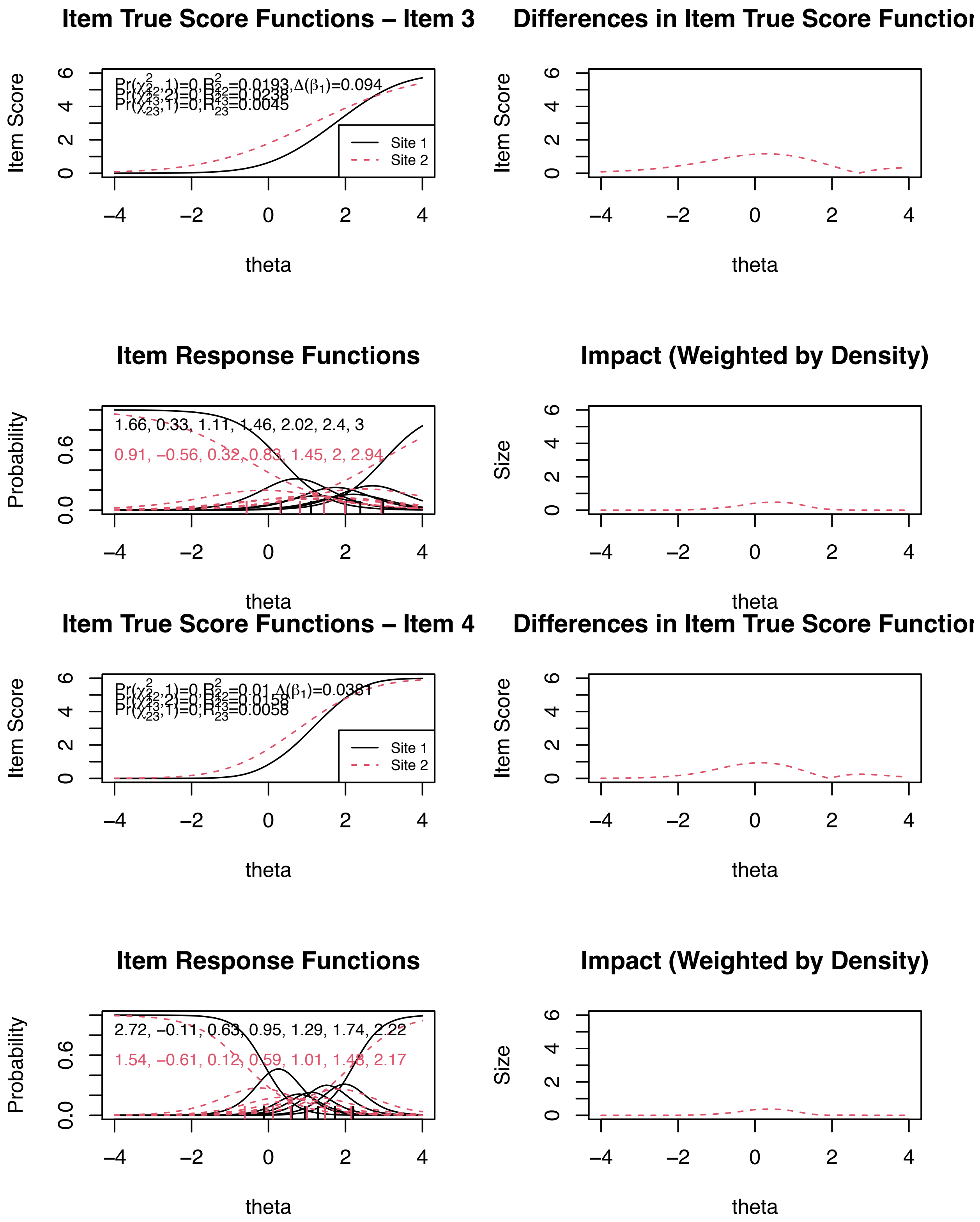
Item True Score Functions – Item 3
Differences in Item True Score Functioı
Item Score
theta
Site 1
Site 2
Item Response Functions
Impact (Weighted by Density)
Probability
Size
1.66, 0.33, 1.11, 1.46, 2.02, 2.4, 3
0.91, −0.56, 0.32, 0.83, 1.45, 2, 2.94
Item True Score Functions – Item 4
2.72, −0.11, 0.63, 0.95, 1.29, 1.74, 2.22
1.54, −0.61, 0.12, 0.59, 1.01, 1.48, 2.17

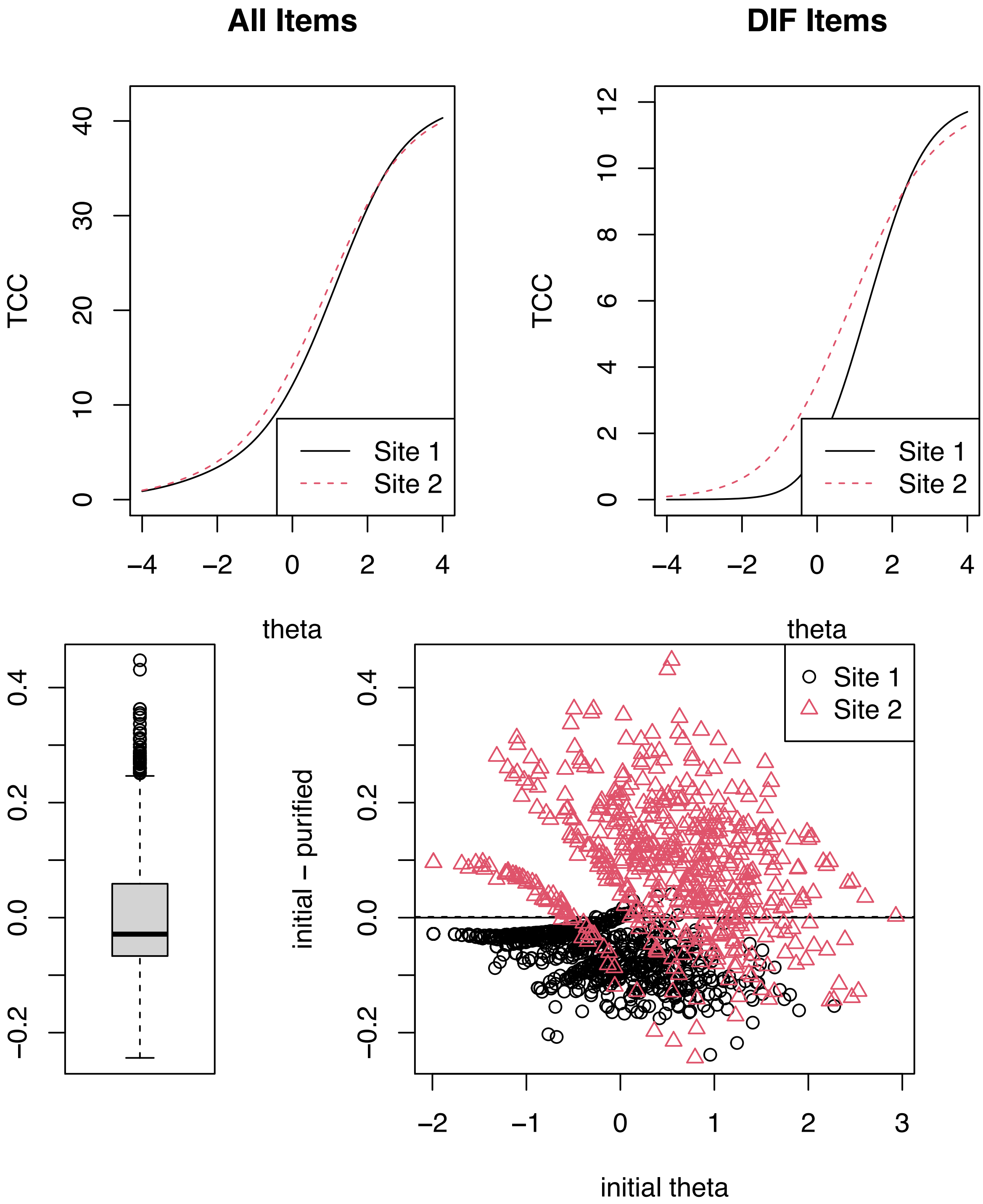

Back to top

Psychosis

Reliability: Psychosis

```
## Cronbach's alpha is 0.416.
## Mean item-total correlation is 0.202.
```

```
## If each item were dropped:
##     raw_alpha std.alpha G6(smc) average_r  S/N alpha se var.r med.r
## Q58      0.27      0.28    0.16      0.16 0.38    0.057    NA  0.16
## Q29      0.28      0.29    0.17      0.17 0.40    0.057    NA  0.17
## Q49      0.40      0.43    0.28      0.28 0.76    0.044    NA  0.28
```

Unidimensionality: Psychosis

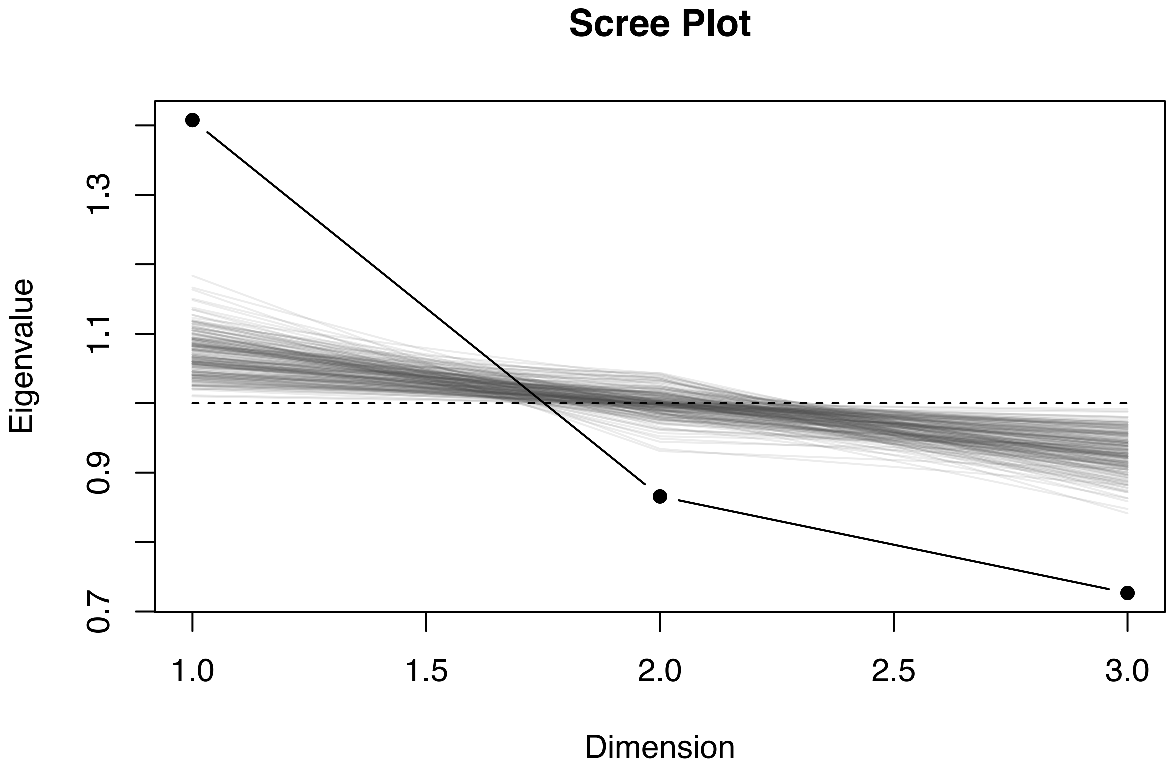


```
## [1] "Ratio of first to second eigenvalues: 1.626"

## [1] 1.4076462 0.8657056 0.7266482

## Factor Analysis using method =  minres
## Call: fa(r = grm_obj$X)
## Standardized loadings (pattern matrix) based upon correlation matrix
##      MR1   h2   u2 com
## Q58 0.54 0.29 0.71   1
## Q29 0.51 0.26 0.74   1
## Q49 0.32 0.10 0.90   1
##
##                 MR1
## SS loadings    0.65
## Proportion Var 0.22
##
## Mean item complexity =  1
## Test of the hypothesis that 1 factor is sufficient.
##
## The degrees of freedom for the null model are  3  and the objective function was  0.12 with Chi Squa
## The degrees of freedom for the model are 0  and the objective function was  0
##
```

```
## The root mean square of the residuals (RMSR) is  0
## The df corrected root mean square of the residuals is  NA
##
## The harmonic number of observations is  595 with the empirical chi square  0  with prob <  NA
## The total number of observations was  619  with Likelihood Chi Square =  0  with prob <  NA
##
## Tucker Lewis Index of factoring reliability =  -Inf
## Fit based upon off diagonal values = 1
## Measures of factor score adequacy
##                                                     MR1
## Correlation of (regression) scores with factors    0.68
## Multiple R square of scores with factors           0.46
## Minimum correlation of possible factor scores     -0.07
```

Graded-Response Model: Psychosis

```
##     Extrmt1 Extrmt2 Extrmt3 Extrmt4 Extrmt5 Extrmt6 Dscrmn
## Q58  -0.406  -0.982  -1.329  -1.636  -2.119  -2.544 -1.464
## Q29  -1.460  -1.887  -2.119  -2.495  -2.699  -2.981 -1.767
## Q49  -1.286  -2.119  -2.741  -3.447  -4.359  -5.238 -0.729
```

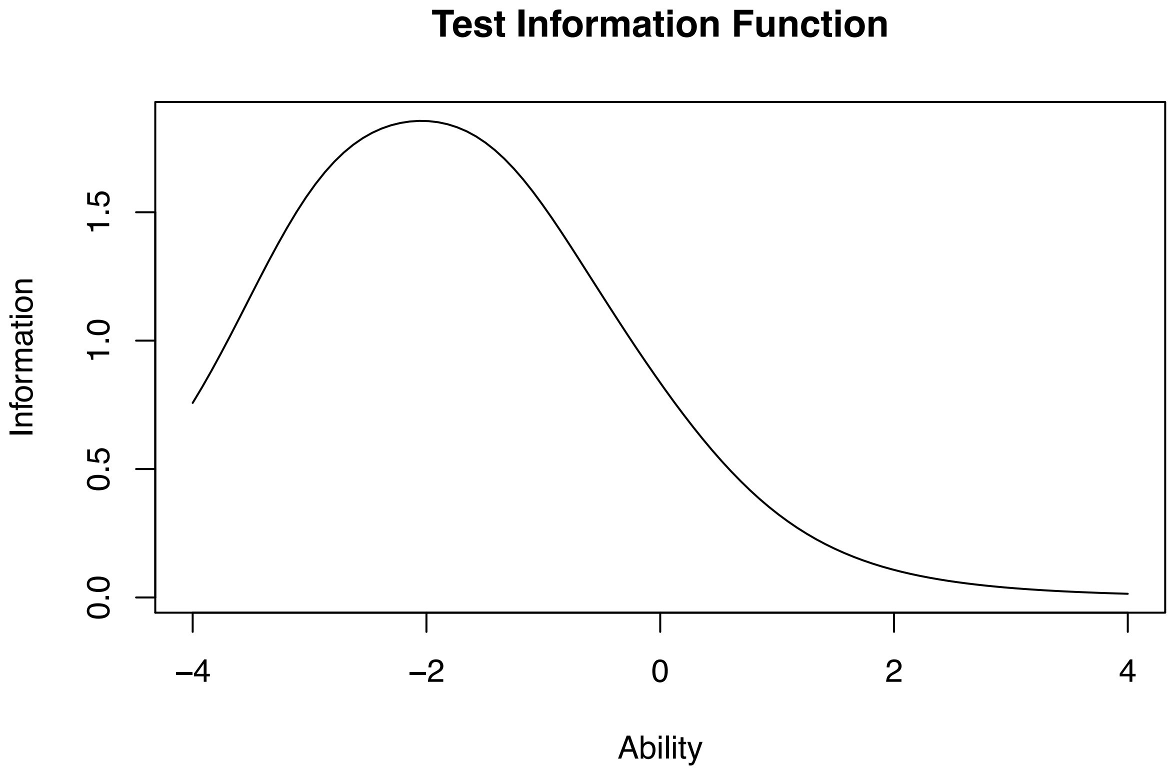

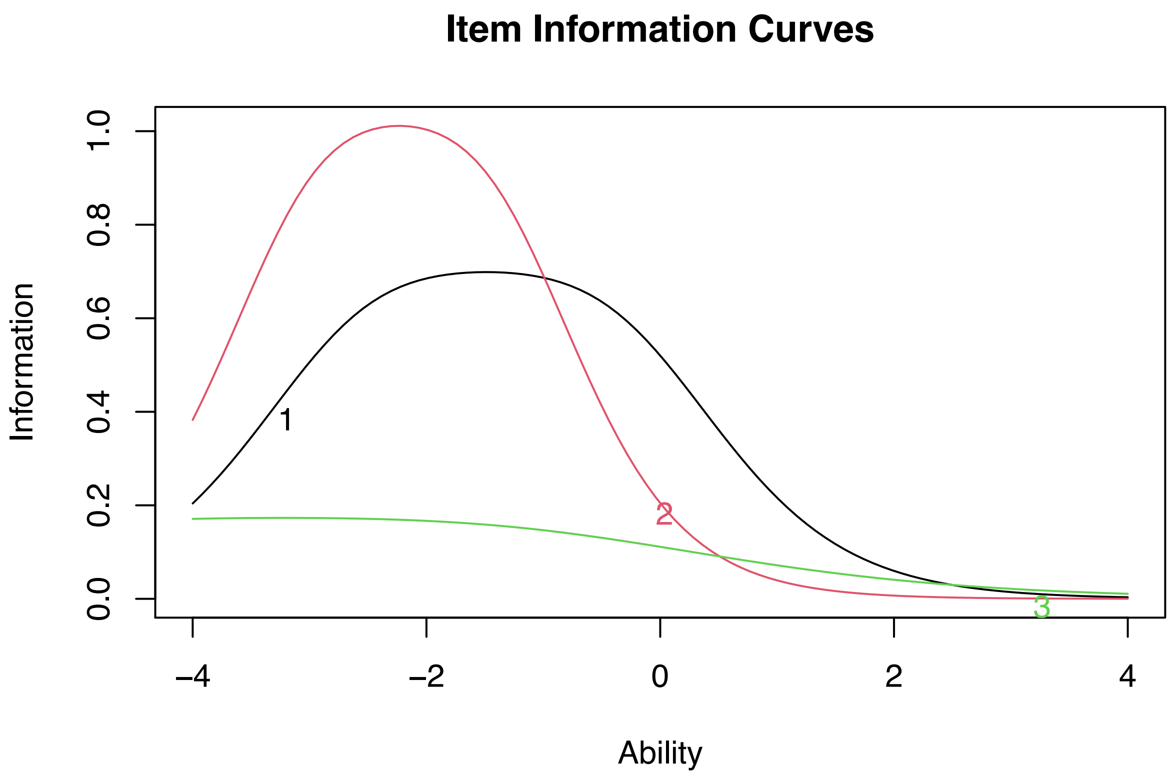

Gender-based DIF: Psychosis

```
## No Gender-based DIF detected
```

Sample-based DIF: Psychosis

```
## No Sample-based DIF detected
```

Back to top

Relational Distress

Reliability: Relational Distress

```
## Cronbach's alpha is 0.746.
## Mean item-total correlation is 0.296.
## If each item were dropped:
##      raw_alpha std.alpha G6(smc) average_r S/N alpha se  var.r med.r
## Q81       0.69      0.69    0.67      0.27 2.3    0.019 0.0086  0.26
## Q25       0.73      0.73    0.71      0.31 2.7    0.017 0.0062  0.31
## Q36       0.70      0.70    0.68      0.28 2.4    0.018 0.0092  0.29
## Q37       0.71      0.70    0.68      0.28 2.4    0.018 0.0087  0.29
## Q65       0.72      0.72    0.70      0.30 2.6    0.017 0.0078  0.31
## Q66       0.74      0.74    0.72      0.33 2.9    0.016 0.0063  0.35
## Q69-      0.71      0.71    0.69      0.29 2.4    0.018 0.0078  0.31
```

Unidimensionality: Relational Distress

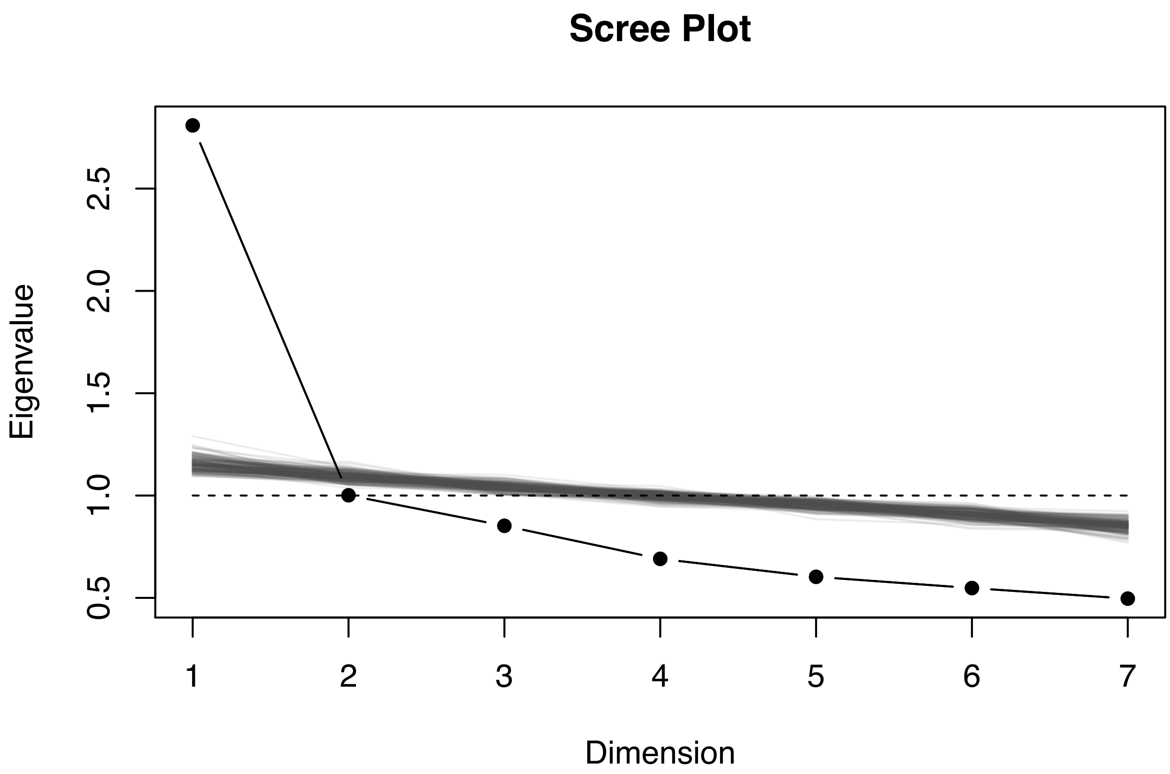


```
## [1] "Ratio of first to second eigenvalues: 2.805"

## [1] 2.8086127 1.0013286 0.8522043 0.6906615 0.6028426 0.5479389 0.4964113

## Factor Analysis using method =  minres
## Call: fa(r = grm_obj$X)
## Standardized loadings (pattern matrix) based upon correlation matrix
##       MR1   h2   u2 com
## Q81  0.65 0.43 0.57   1
## Q25  0.46 0.21 0.79   1
## Q36  0.62 0.38 0.62   1
## Q37  0.60 0.36 0.64   1
## Q65  0.51 0.26 0.74   1
## Q66  0.40 0.16 0.84   1
## Q69 -0.58 0.34 0.66   1
##
##                 MR1
## SS loadings    2.14
## Proportion Var 0.31
##
## Mean item complexity =  1
## Test of the hypothesis that 1 factor is sufficient.
##
## The degrees of freedom for the null model are  21  and the objective function was  1.3 with Chi Squa
## The degrees of freedom for the model are 14  and the objective function was  0.14
##
## The root mean square of the residuals (RMSR) is  0.06
## The df corrected root mean square of the residuals is  0.07
```

```
##
## The harmonic number of observations is  594 with the empirical chi square  86.05  with prob <  2.1e-
## The total number of observations was  619  with Likelihood Chi Square =  87.49  with prob <  1.1e-12
##
## Tucker Lewis Index of factoring reliability =  0.859
## RMSEA index =  0.092  and the 90 % confidence intervals are  0.074 0.111
## BIC =  -2.51
## Fit based upon off diagonal values = 0.96
## Measures of factor score adequacy
##                                                    MR1
## Correlation of (regression) scores with factors   0.87
## Multiple R square of scores with factors          0.77
## Minimum correlation of possible factor scores     0.53
```

Graded-Response Model: Relational Distress

```
##     Extrmt1 Extrmt2 Extrmt3 Extrmt4 Extrmt5 Extrmt6 Dscrmn
## Q81  -1.595  -0.899  -0.348   0.204   0.742   1.405  1.690
## Q25  -2.363  -1.454  -0.766  -0.212   0.453   1.275  1.051
## Q36  -0.286   0.325   0.725   1.267   1.832   2.431  1.751
## Q37  -1.469  -0.390   0.249   0.870   1.409   2.158  1.439
## Q65  -0.140   0.967   1.539   2.143   2.848   3.685  1.237
## Q66  -0.140   0.740   1.328   2.107   2.956   3.634  0.980
## Q69   1.602   0.937   0.404  -0.283  -0.741  -1.381 -1.416
```

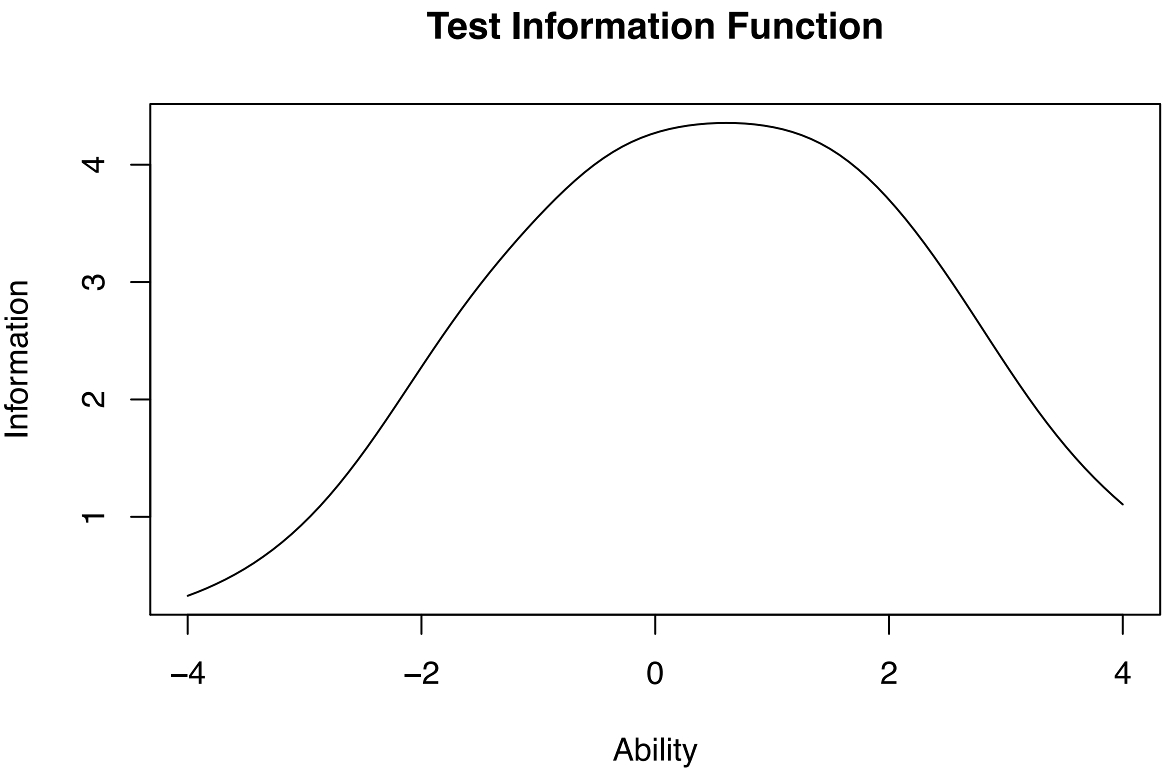

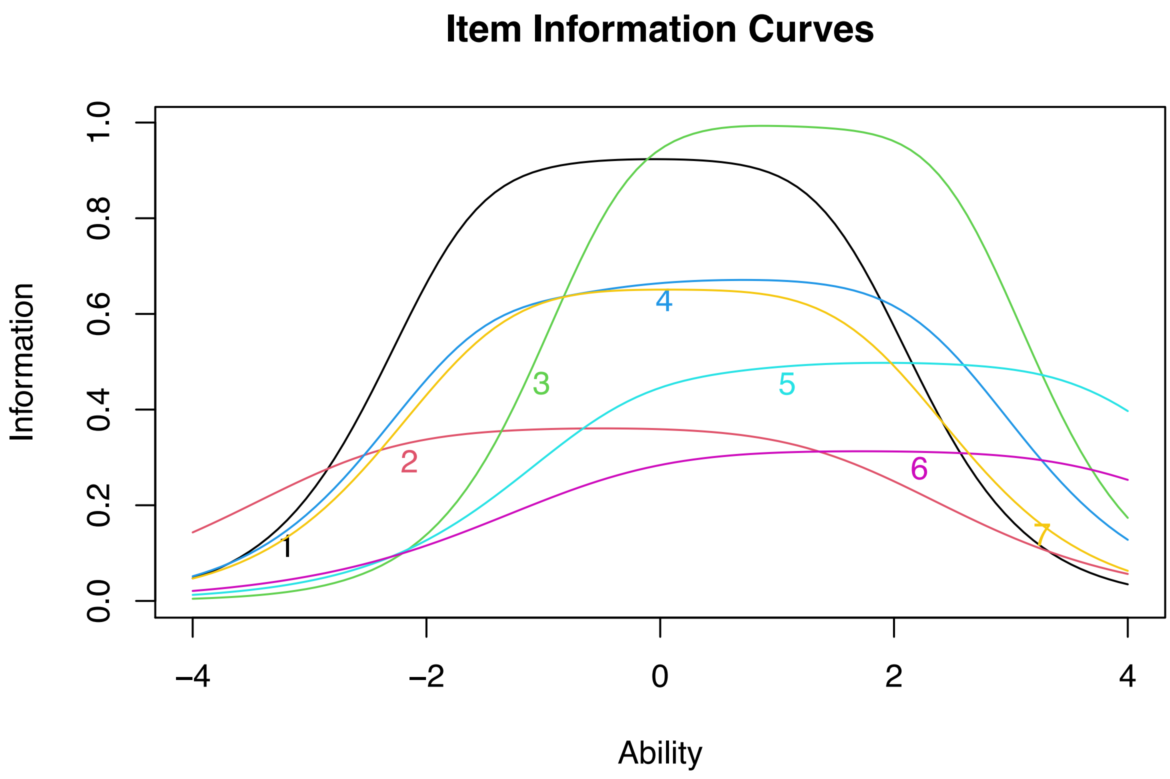

Gender-based DIF: Relational Distress

```
## Call:
## lordif::lordif(resp.data = as.data.frame(sex.data), group = sex)
##
##   Number of DIF groups: 2
##
##   Number of items flagged for DIF: 2 of 7
##
##   Items flagged: 2, 6
##
##   Number of iterations for purification: 2 of 10
##
##   Detection criterion: Chisqr
##
##   Threshold: alpha = 0.01
##
##   item ncat  chi12  chi13  chi23
## 1    1    7 0.3710 0.6647 0.8975
## 2    2    7 0.0000 0.0000 0.1495
## 3    3    6 0.9503 0.9970 0.9624
## 4    4    7 0.2840 0.5578 0.8886
## 5    5    7 0.2566 0.4943 0.7266
## 6    6    7 0.0000 0.0000 0.5641
## 7    7    7 0.8048 0.0858 0.0277
```

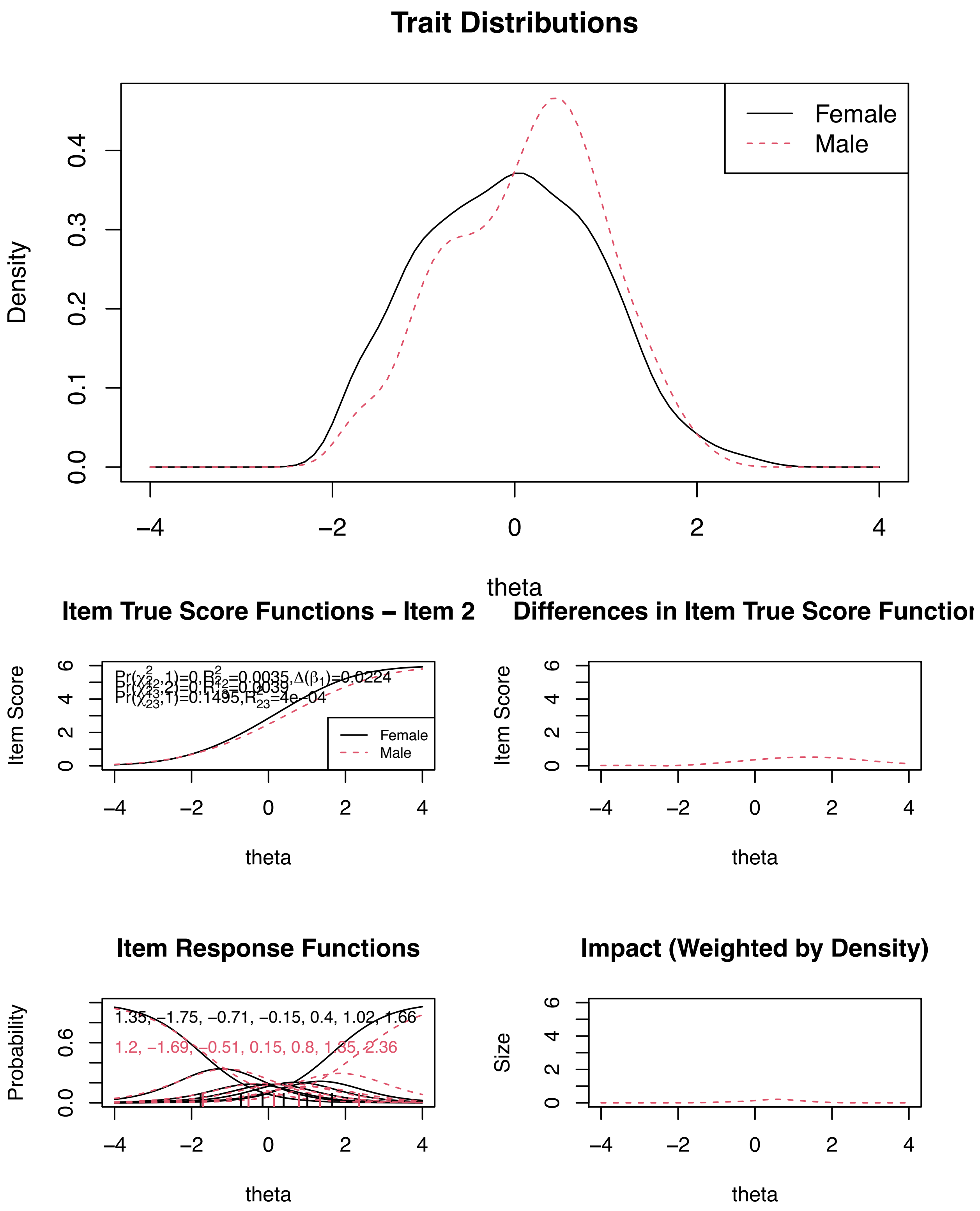
Trait Distributions
Density
theta
Female
Male
Item True Score Functions – Item 2
Item Score
Differences in Item True Score Functio
Item Response Functions
Probability
1.35, –1.75, –0.71, –0.15, 0.4, 1.02, 1.66
1.2, –1.69, –0.51, 0.15, 0.8, 1.35, 2.36
Impact (Weighted by Density)
Size

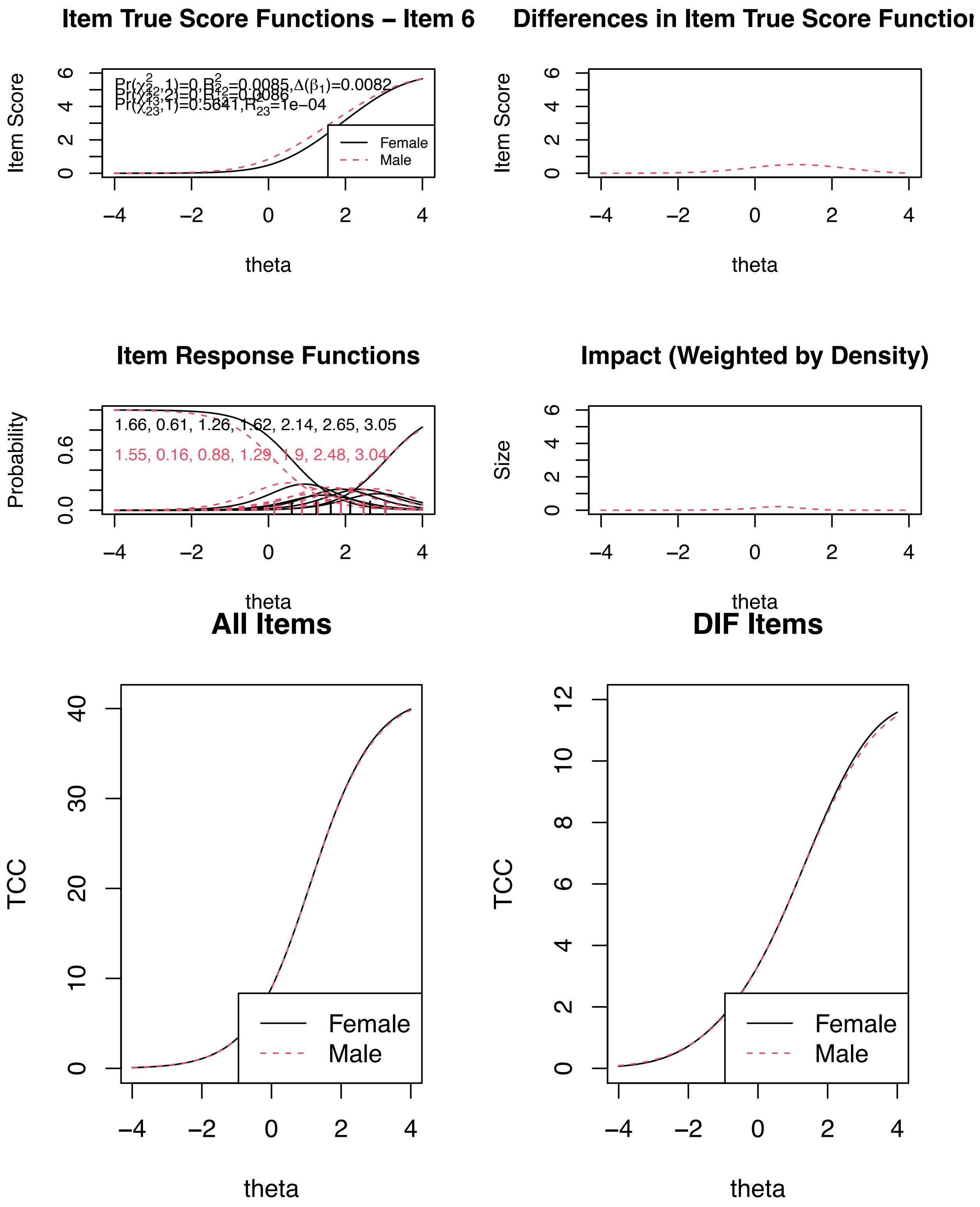
Item True Score Functions – Item 6
Item Score
theta
Female
Male
Differences in Item True Score Functio
Item Score
theta
Item Response Functions
Probability
1.66, 0.61, 1.26, 1.62, 2.14, 2.65, 3.05
1.55, 0.16, 0.88, 1.29, 1.9, 2.48, 3.04
theta
Impact (Weighted by Density)
Size
theta
All Items
TCC
Female
Male
theta
DIF Items
TCC
Female
Male
theta

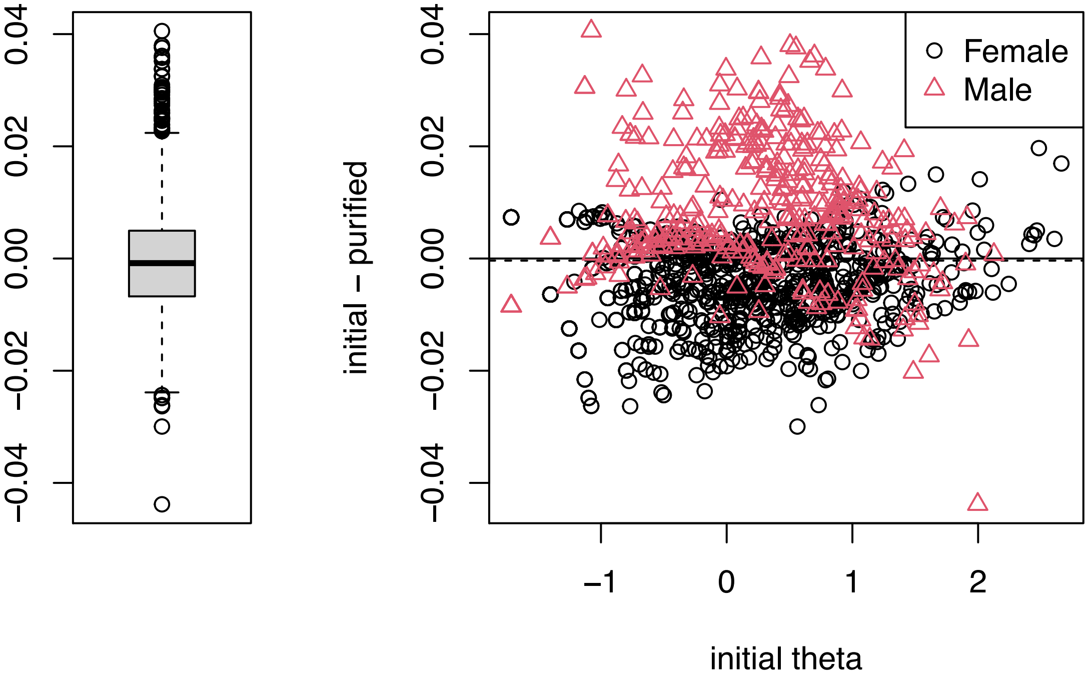

Sample-based DIF: Relational Distress

```
## Call:
## lordif::lordif(resp.data = as.data.frame(sample.data), group = clinYN)
##
##   Number of DIF groups: 2
##
##   Number of items flagged for DIF: 7 of 7
##
##   Items flagged: 1, 2, 3, 4, 5, 6, 7
##
##   Number of iterations for purification: 4 of 10
##
##   Detection criterion: Chisqr
##
##   Threshold: alpha = 0.01
##
##   item ncat chi12 chi13  chi23
## 1    1    7     0     0 0.0014
## 2    2    7     0     0 0.0714
## 3    3    6     0     0 0.3496
## 4    4    7     0     0 0.0203
## 5    5    5     0     0 0.0070
## 6    6    7     0     0 0.0000
## 7    7    7     0     0 0.6452
```

Back to top

Resilience

Reliability: Resilience

```
## Cronbach's alpha is 0.768.
## Mean item-total correlation is 0.218.
## If each item were dropped:
##      raw_alpha std.alpha G6(smc) average_r S/N alpha se var.r med.r
## Q27-      0.75      0.75    0.76      0.22 3.0    0.015 0.011  0.21
## Q6        0.75      0.75    0.76      0.21 3.0    0.015 0.011  0.21
## Q15       0.74      0.75    0.77      0.21 3.0    0.015 0.014  0.20
## Q22       0.74      0.74    0.76      0.21 2.9    0.015 0.014  0.20
## Q33-      0.76      0.76    0.78      0.22 3.2    0.014 0.014  0.21
## Q40       0.75      0.75    0.77      0.21 3.0    0.015 0.014  0.21
## Q55       0.78      0.78    0.80      0.24 3.5    0.013 0.011  0.23
## Q56       0.76      0.76    0.77      0.23 3.2    0.014 0.012  0.22
## Q67       0.74      0.74    0.77      0.21 2.9    0.015 0.014  0.20
## Q70       0.76      0.76    0.78      0.22 3.2    0.014 0.014  0.21
## Q84       0.75      0.75    0.76      0.22 3.0    0.015 0.013  0.21
## Q89       0.75      0.75    0.77      0.21 2.9    0.015 0.013  0.21
```

Unidimensionality: Resilience

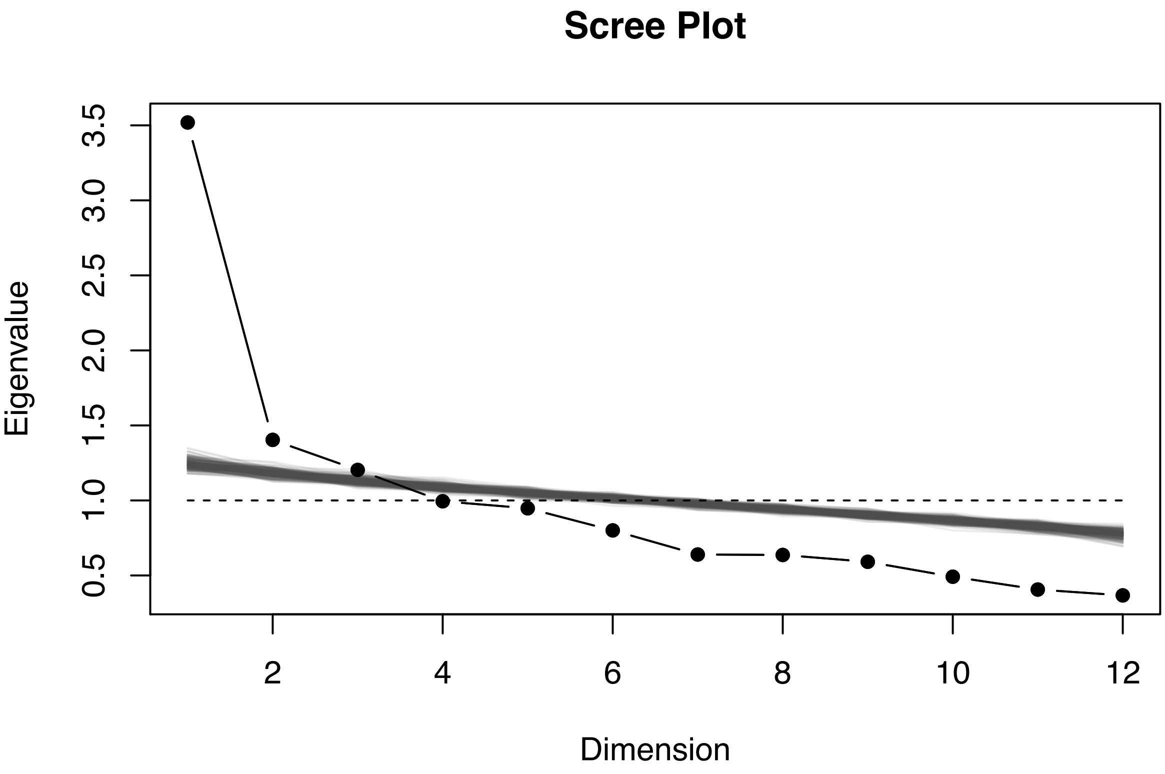


```
## [1] "Ratio of first to second eigenvalues: 2.507"

##  [1] 3.5189340 1.4035499 1.2032386 0.9944428 0.9475198 0.8002591 0.6395633
##  [8] 0.6366297 0.5910327 0.4915612 0.4061040 0.3671649

## Factor Analysis using method =  minres
## Call: fa(r = grm_obj$X)
```

```
## Standardized loadings (pattern matrix) based upon correlation matrix
##       MR1    h2   u2 com
## Q27 -0.52 0.275 0.73   1
## Q6   0.54 0.290 0.71   1
## Q15  0.55 0.305 0.69   1
## Q22  0.56 0.312 0.69   1
## Q33 -0.41 0.166 0.83   1
## Q40  0.52 0.267 0.73   1
## Q55  0.18 0.033 0.97   1
## Q56  0.36 0.127 0.87   1
## Q67  0.57 0.325 0.67   1
## Q70  0.42 0.173 0.83   1
## Q84  0.46 0.210 0.79   1
## Q89  0.55 0.299 0.70   1
##
##                 MR1
## SS loadings    2.78
## Proportion Var 0.23
##
## Mean item complexity =  1
## Test of the hypothesis that 1 factor is sufficient.
##
## The degrees of freedom for the null model are  66  and the objective function was  2.54 with Chi Squa
## The degrees of freedom for the model are 54  and the objective function was  0.87
##
## The root mean square of the residuals (RMSR) is  0.09
## The df corrected root mean square of the residuals is  0.1
##
## The harmonic number of observations is  553 with the empirical chi square  559.56  with prob <  3.5e-
## The total number of observations was  619  with Likelihood Chi Square =  533.07  with prob <  5.7e-80
##
## Tucker Lewis Index of factoring reliability =  0.607
## RMSEA index =  0.12  and the 90 % confidence intervals are  0.111 0.129
## BIC =  185.96
## Fit based upon off diagonal values = 0.87
## Measures of factor score adequacy
##                                                   MR1
## Correlation of (regression) scores with factors   0.89
## Multiple R square of scores with factors          0.79
## Minimum correlation of possible factor scores     0.58
```

Graded-Response Model: Resilience

```
##     Extrmt1 Extrmt2 Extrmt3 Extrmt4 Extrmt5 Extrmt6 Dscrmn
## Q27   2.149   1.182   0.488  -0.160  -0.697  -1.553 -1.111
## Q6   -1.952  -1.231  -0.707  -0.117   0.466   1.401  1.168
## Q15  -2.479  -1.537  -0.933  -0.283   0.564   1.490  1.264
## Q22  -1.038  -0.183   0.532   1.178   1.647   2.257  1.341
## Q33   3.300   2.142   1.455   0.596  -0.479  -1.517 -0.823
## Q40  -1.664  -0.385   0.500   1.217   2.092   3.015  1.153
## Q55  -3.975  -1.878  -0.214   2.049   3.716   5.843  0.405
## Q56  -2.755  -1.713  -0.925   0.032   0.782   1.855  0.723
## Q67  -1.938  -1.317  -0.624   0.157   0.968   1.914  1.445
```

```
## Q70  -1.174  -0.105   0.675   1.497   2.148   3.132  0.886
## Q84  -2.372  -1.388  -0.711   0.165   1.028   1.821  0.939
## Q89  -2.135  -1.216  -0.431   0.431   1.122   1.914  1.262
```

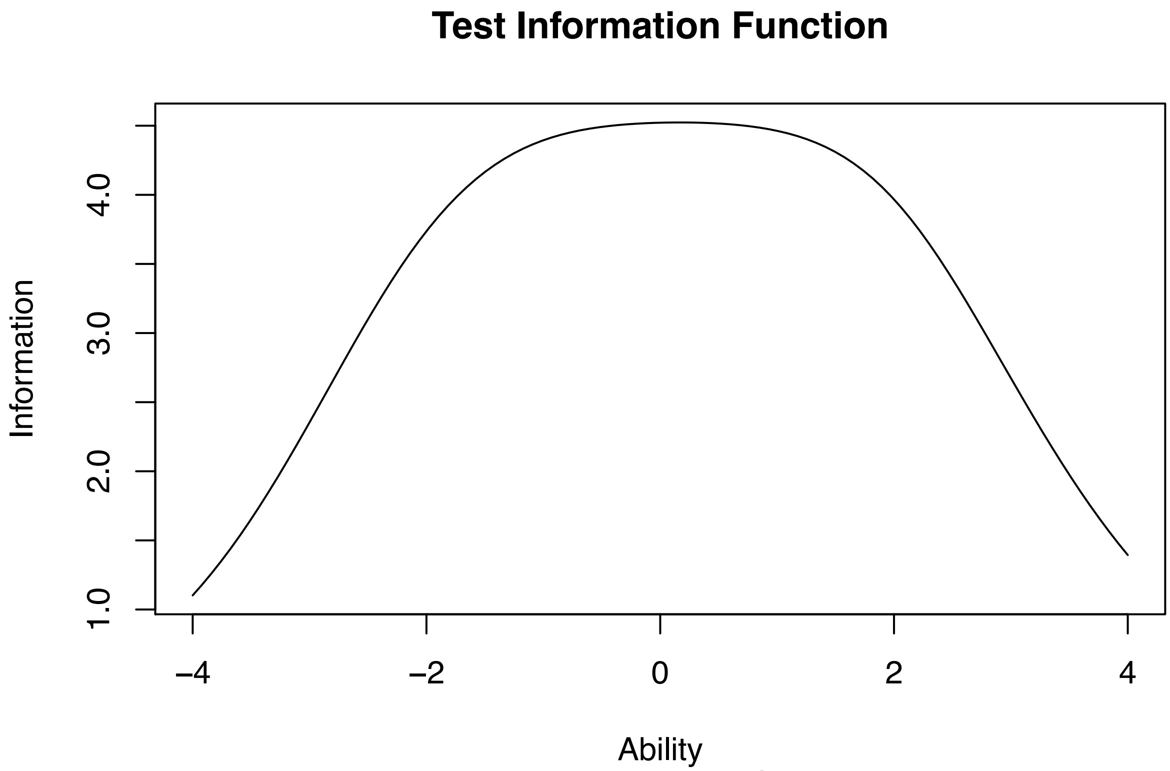

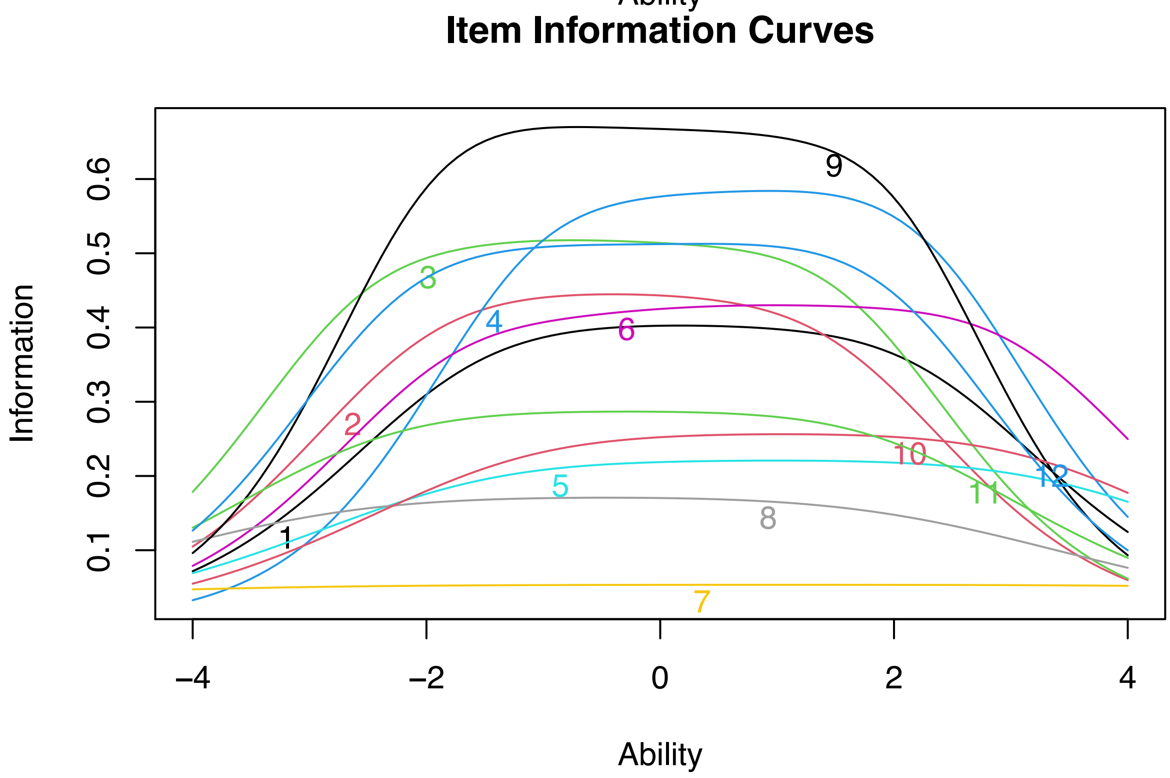

Gender-based DIF: Resilience

```
## Call:
## lordif::lordif(resp.data = as.data.frame(sex.data), group = sex)
##
##   Number of DIF groups: 2
##
##   Number of items flagged for DIF: 3 of 12
##
##   Items flagged: 2, 7, 8
##
##   Number of iterations for purification: 2 of 10
##
##   Detection criterion: Chisqr
##
##   Threshold: alpha = 0.01
##
##    item ncat  chi12  chi13  chi23
## 1     1    7 0.4444 0.6752 0.6543
## 2     2    7 0.0000 0.0001 0.4558
## 3     3    7 0.0136 0.0355 0.4438
## 4     4    7 0.1324 0.1125 0.1468
## 5     5    7 0.3505 0.4547 0.4013
## 6     6    7 0.3245 0.5750 0.7125
## 7     7    7 0.0573 0.0007 0.0009
## 8     8    7 0.8336 0.0059 0.0014
## 9     9    7 0.5698 0.5684 0.3691
## 10   10    7 0.0709 0.1900 0.8094
## 11   11    7 0.0257 0.0299 0.1528
## 12   12    7 0.9439 0.9876 0.8874
```

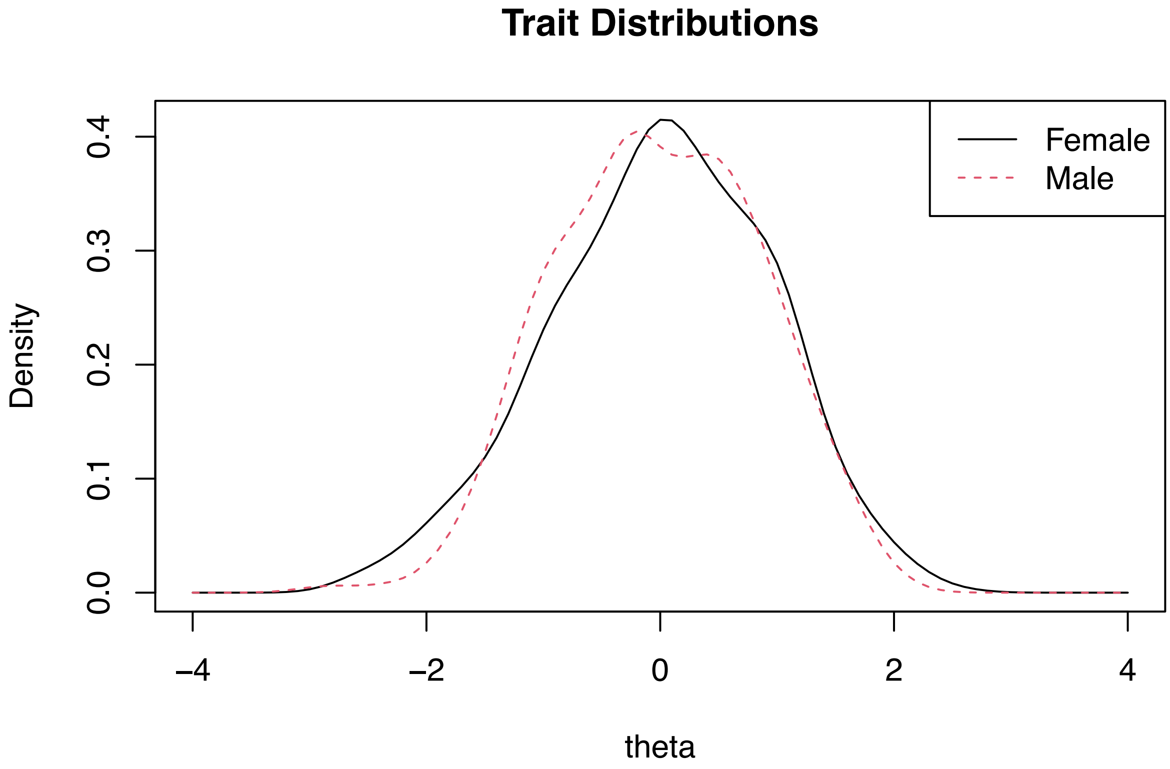

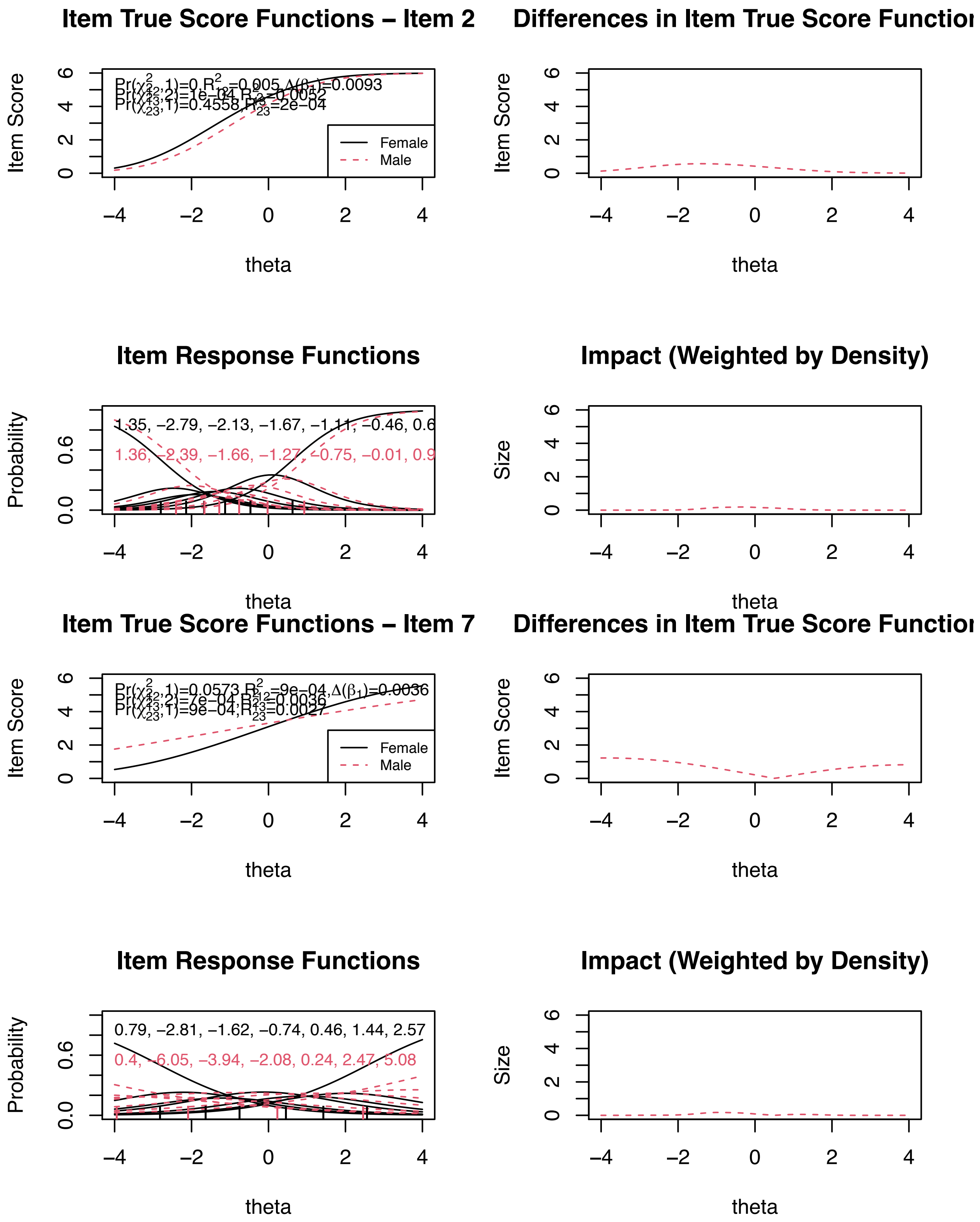
Item True Score Functions – Item 2
Differences in Item True Score Functio
Item Score
theta
Female
Male
Item Response Functions
Impact (Weighted by Density)
Probability
Size
Item True Score Functions – Item 7
Differences in Item True Score Functio
Item Score
theta
Female
Male
Item Response Functions
Impact (Weighted by Density)
0.79, −2.81, −1.62, −0.74, 0.46, 1.44, 2.57
Probability
Size
theta

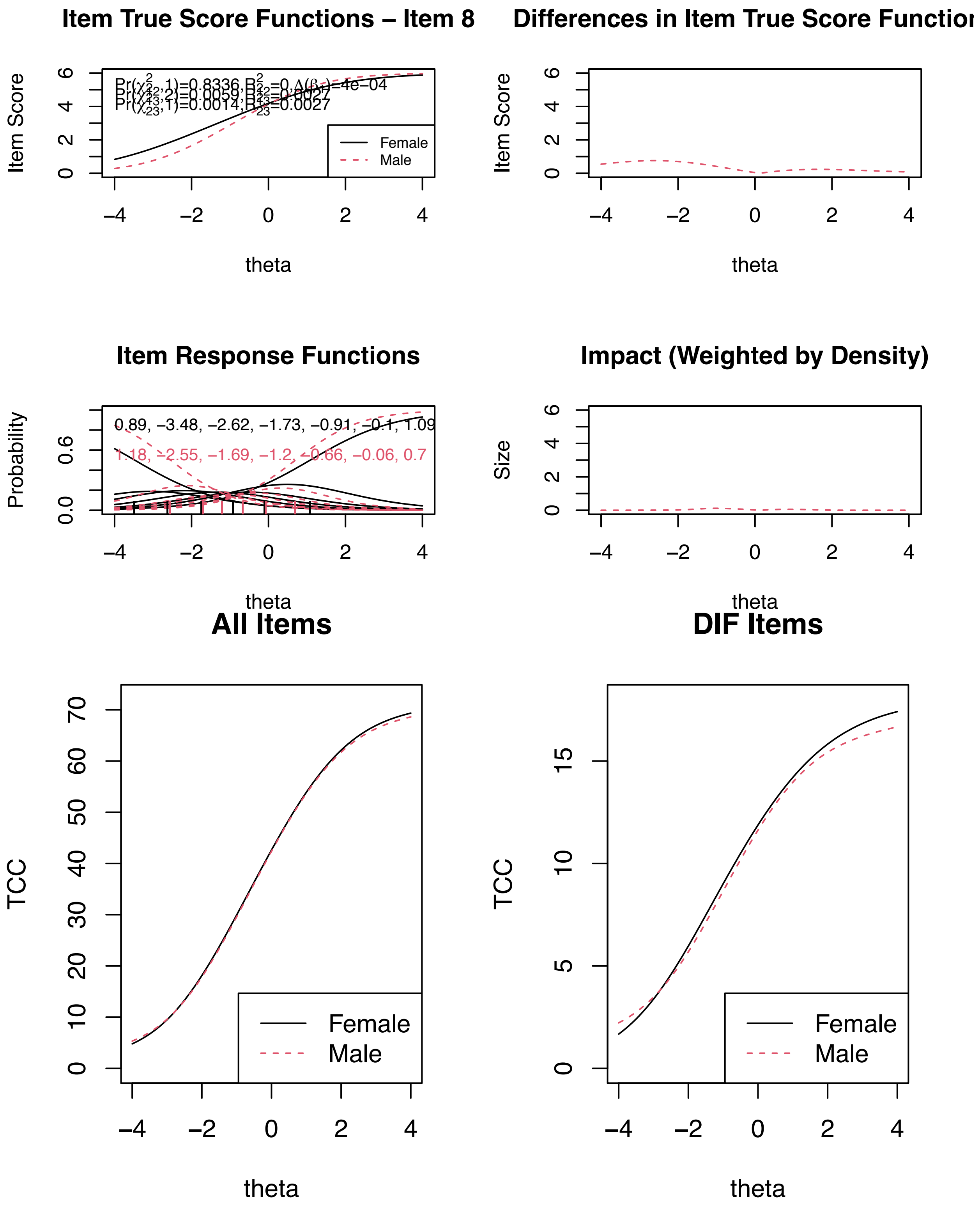
Item True Score Functions – Item 8
Differences in Item True Score Functio
Item Score
theta
Female
Male
Item Response Functions
Probability
0.89, −3.48, −2.62, −1.73, −0.91, −0.1, 1.09
1.18, −2.55, −1.69, −1.2, −0.66, −0.06, 0.7
Impact (Weighted by Density)
Size
All Items
DIF Items
TCC

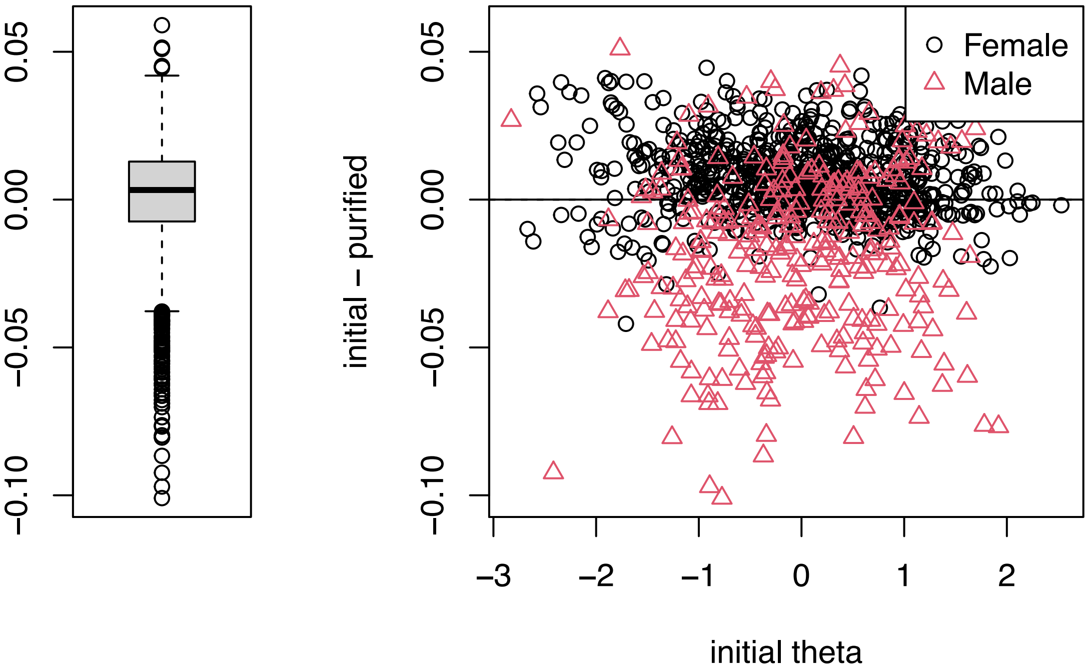

Sample-based DIF: Resilience

```
## Call:
## lordif::lordif(resp.data = as.data.frame(sample.data), group = clinYN)
##
##   Number of DIF groups: 2
##
##   Number of items flagged for DIF: 7 of 12
##
##   Items flagged: 1, 2, 3, 4, 7, 9, 10
##
##   Number of iterations for purification: 5 of 10
##
##   Detection criterion: Chisqr
##
##   Threshold: alpha = 0.01
##
##    item ncat  chi12  chi13  chi23
## 1     1    7 0.0000 0.0000 0.7361
## 2     2    6 0.0000 0.0000 0.1685
## 3     3    7 0.0000 0.0000 0.0000
## 4     4    7 0.0000 0.0000 0.6677
## 5     5    7 0.0274 0.0838 0.7617
## 6     6    7 0.9819 0.0661 0.0198
## 7     7    7 0.0031 0.0000 0.0000
## 8     8    7 0.0179 0.0607 0.9940
## 9     9    7 0.0000 0.0000 0.5486
## 10   10    7 0.0000 0.0000 0.0735
## 11   11    7 0.0310 0.0835 0.5760
## 12   12    7 0.0652 0.1601 0.6070
```

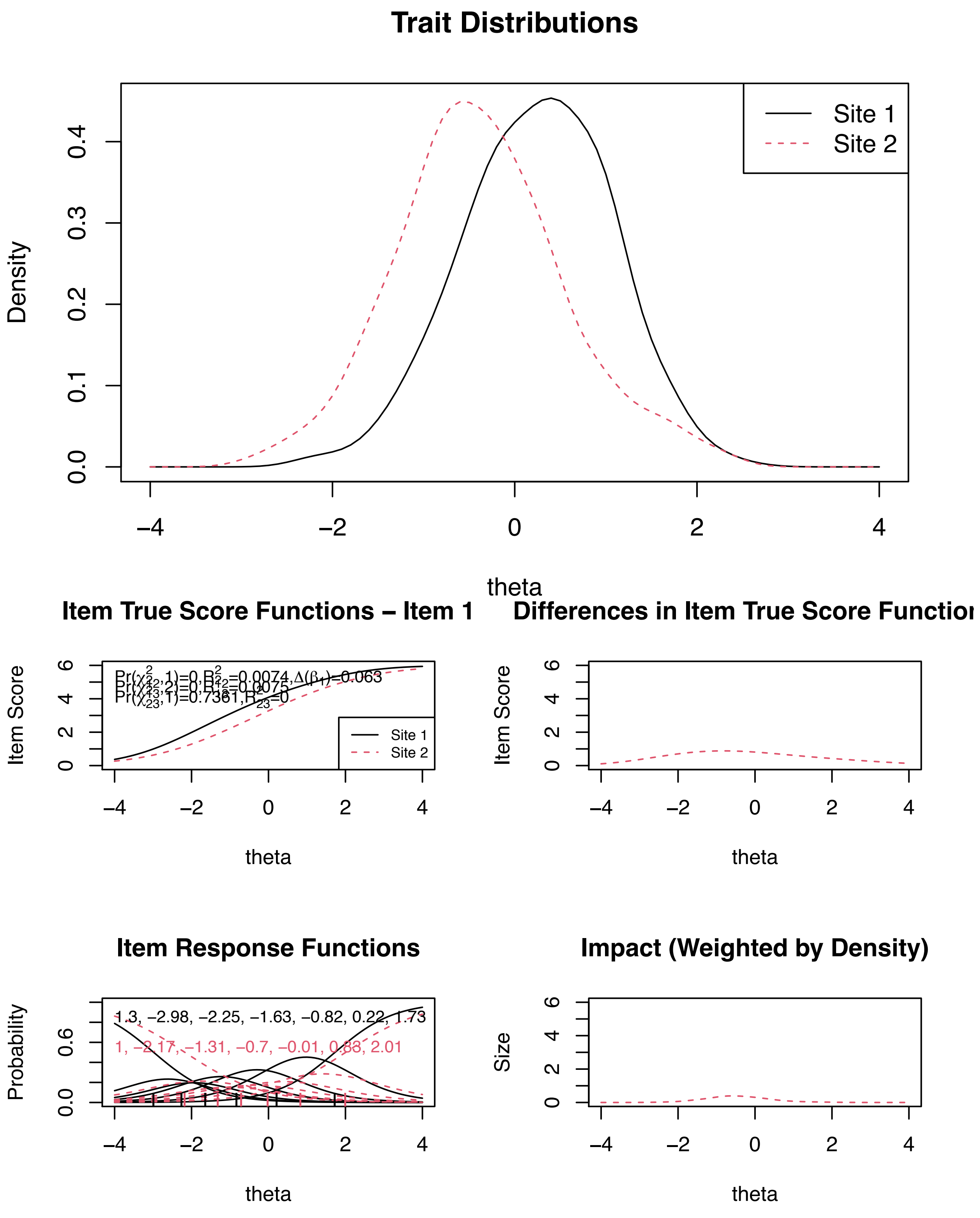
Trait Distributions
Density
theta
Site 1
Site 2
Item True Score Functions – Item 1
Item Score
theta
Site 1
Site 2
Differences in Item True Score Functio
Item Score
theta
Item Response Functions
Probability
theta
1.3, −2.98, −2.25, −1.63, −0.82, 0.22, 1.73
1, −2.17, −1.31, −0.7, −0.01, 0.88, 2.01
Impact (Weighted by Density)
Size
theta

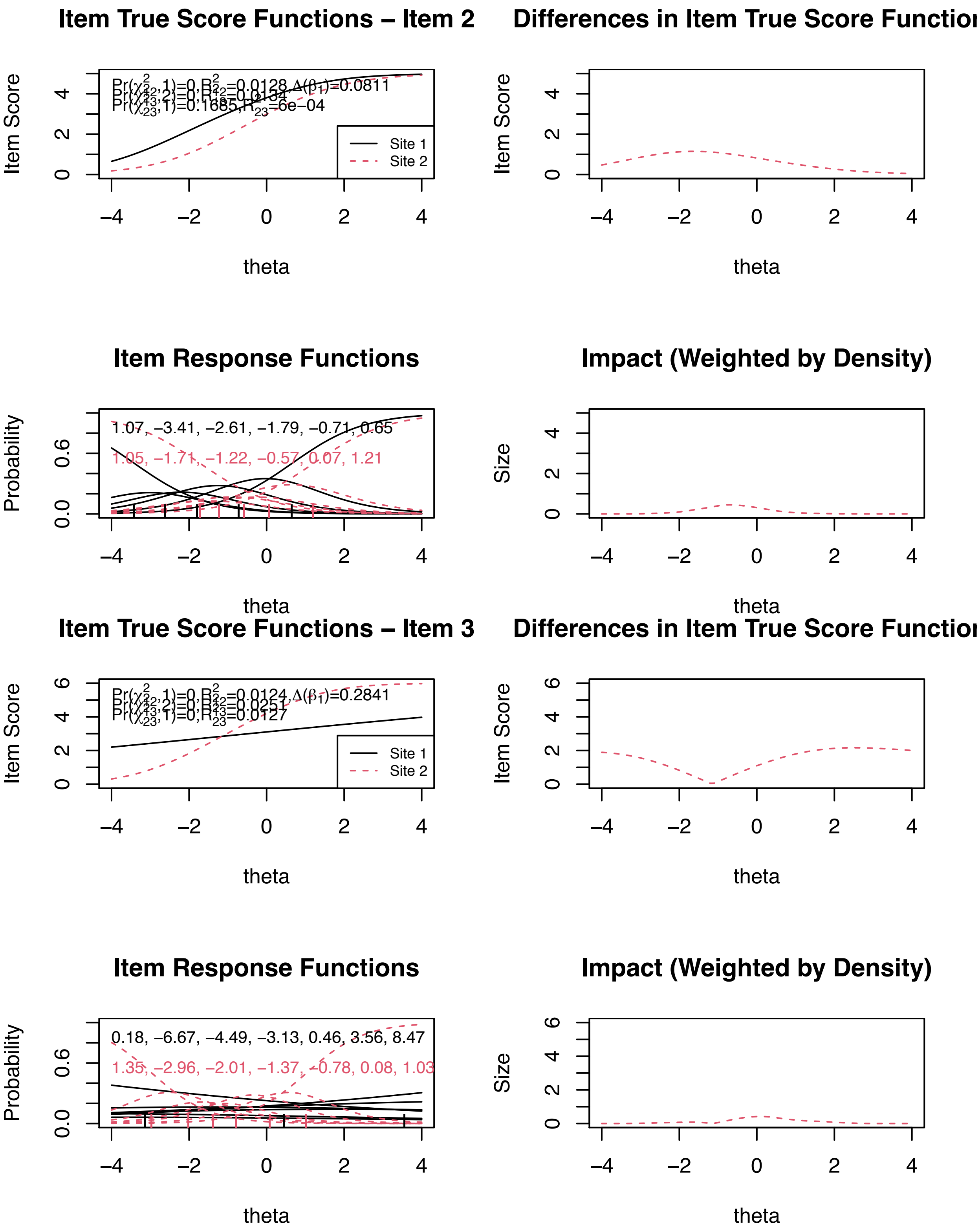
Item True Score Functions – Item 2
Differences in Item True Score Functio
Item Score
theta
Site 1
Site 2
Item Response Functions
Impact (Weighted by Density)
Probability
Size
1.07, –3.41, –2.61, –1.79, –0.71, 0.65
1.05, –1.71, –1.22, –0.57, 0.07, 1.21
Item True Score Functions – Item 3
0.18, –6.67, –4.49, –3.13, 0.46, 3.56, 8.47
1.35, –2.96, –2.01, –1.37, –0.78, 0.08, 1.03

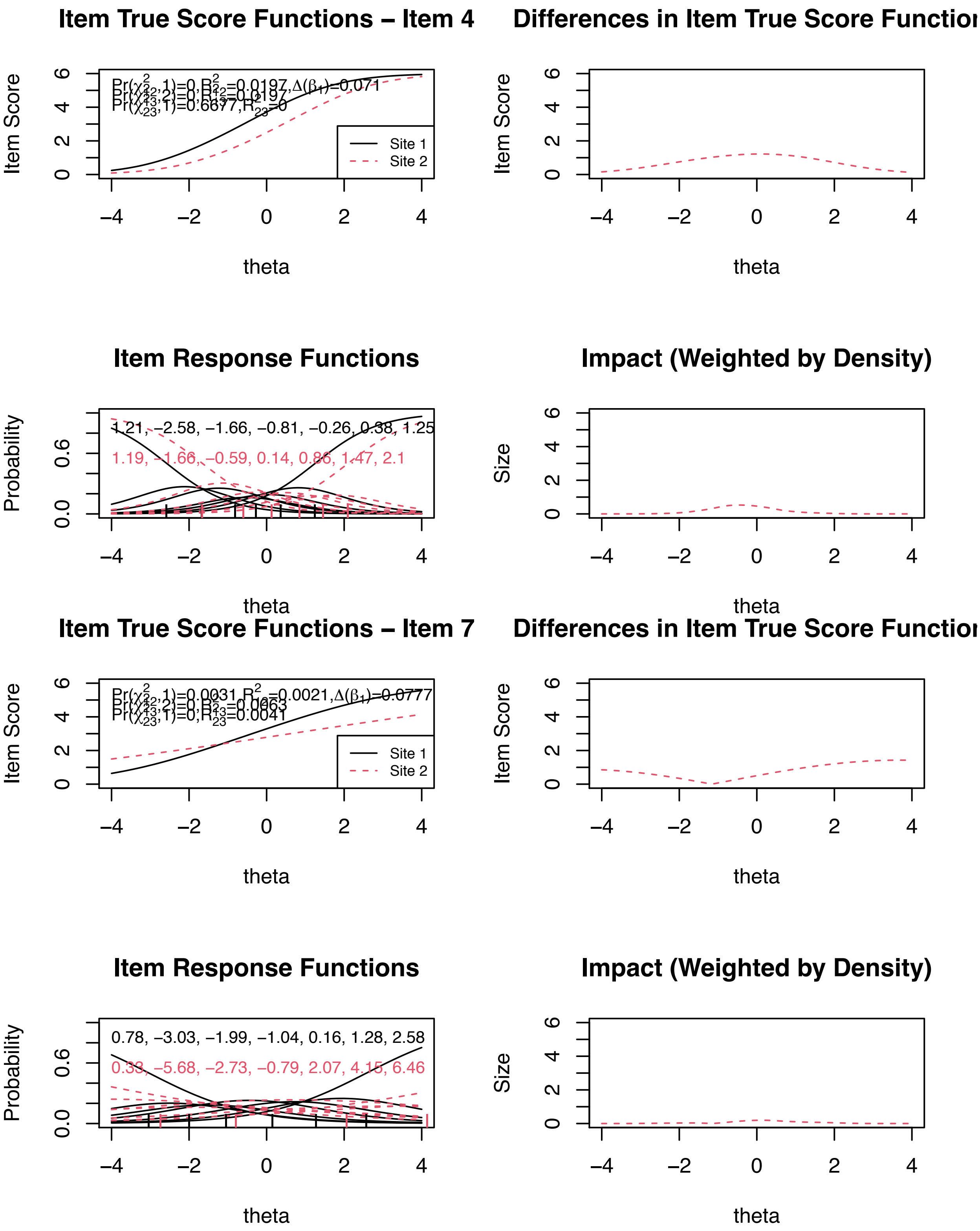
Item True Score Functions – Item 4
Differences in Item True Score Functions
Item Score
theta
Site 1
Site 2
Item Response Functions
Impact (Weighted by Density)
Probability
Size
1.21, –2.58, –1.66, –0.81, –0.26, 0.38, 1.25
1.19, –1.66, –0.59, 0.14, 0.86, 1.47, 2.1
Item True Score Functions – Item 7
0.78, –3.03, –1.99, –1.04, 0.16, 1.28, 2.58
0.38, –5.68, –2.73, –0.79, 2.07, 4.15, 6.46

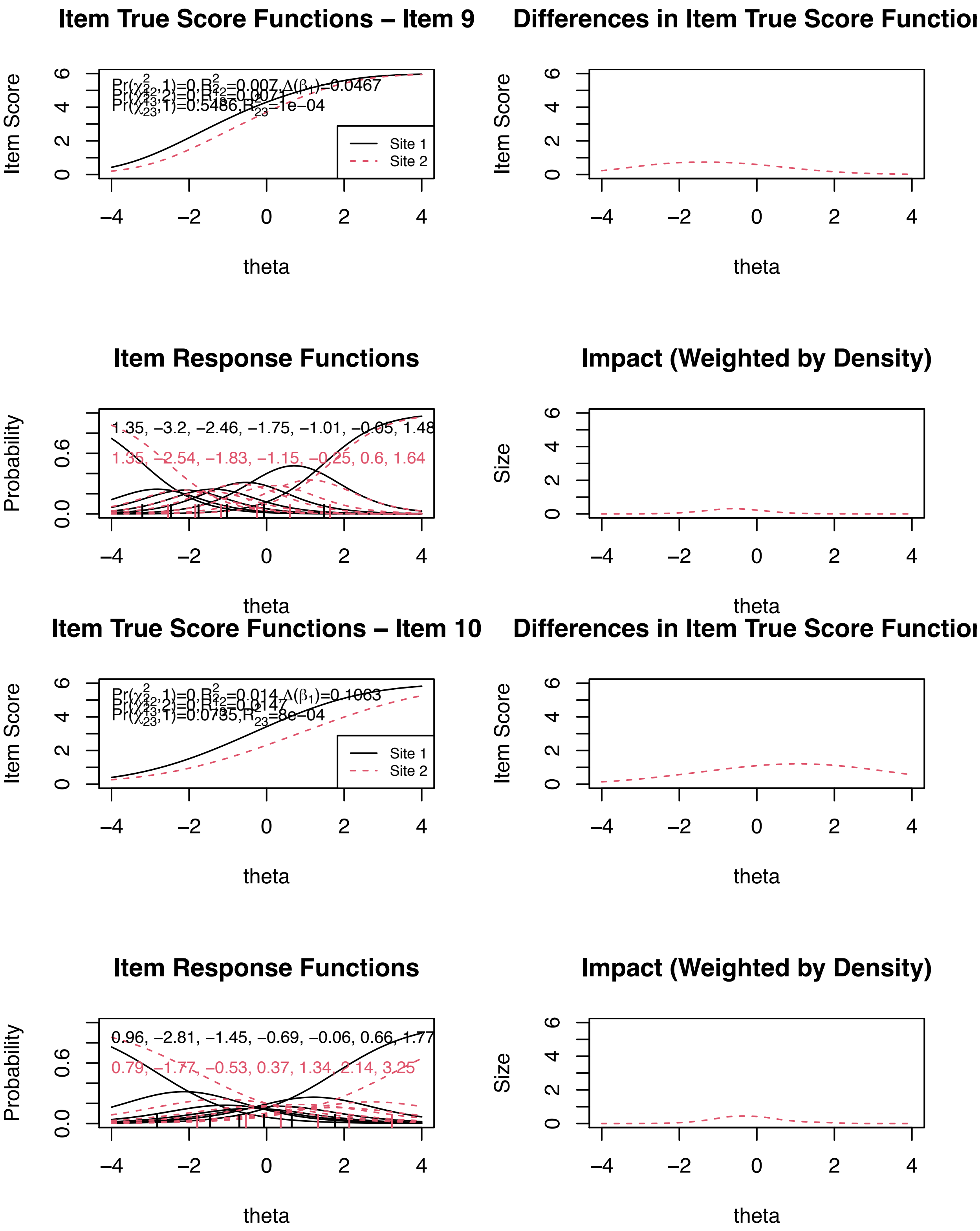
Item True Score Functions – Item 9
Differences in Item True Score Functio
Item Score
theta
Site 1
Site 2
Item Response Functions
Impact (Weighted by Density)
Probability
Size
1.35, −3.2, −2.46, −1.75, −1.01, −0.05, 1.48
1.35, −2.54, −1.83, −1.15, −0.25, 0.6, 1.64
Item True Score Functions – Item 10
Differences in Item True Score Functio
Item Response Functions
Impact (Weighted by Density)
0.96, −2.81, −1.45, −0.69, −0.06, 0.66, 1.77
0.79, −1.77, −0.53, 0.37, 1.34, 2.14, 3.25

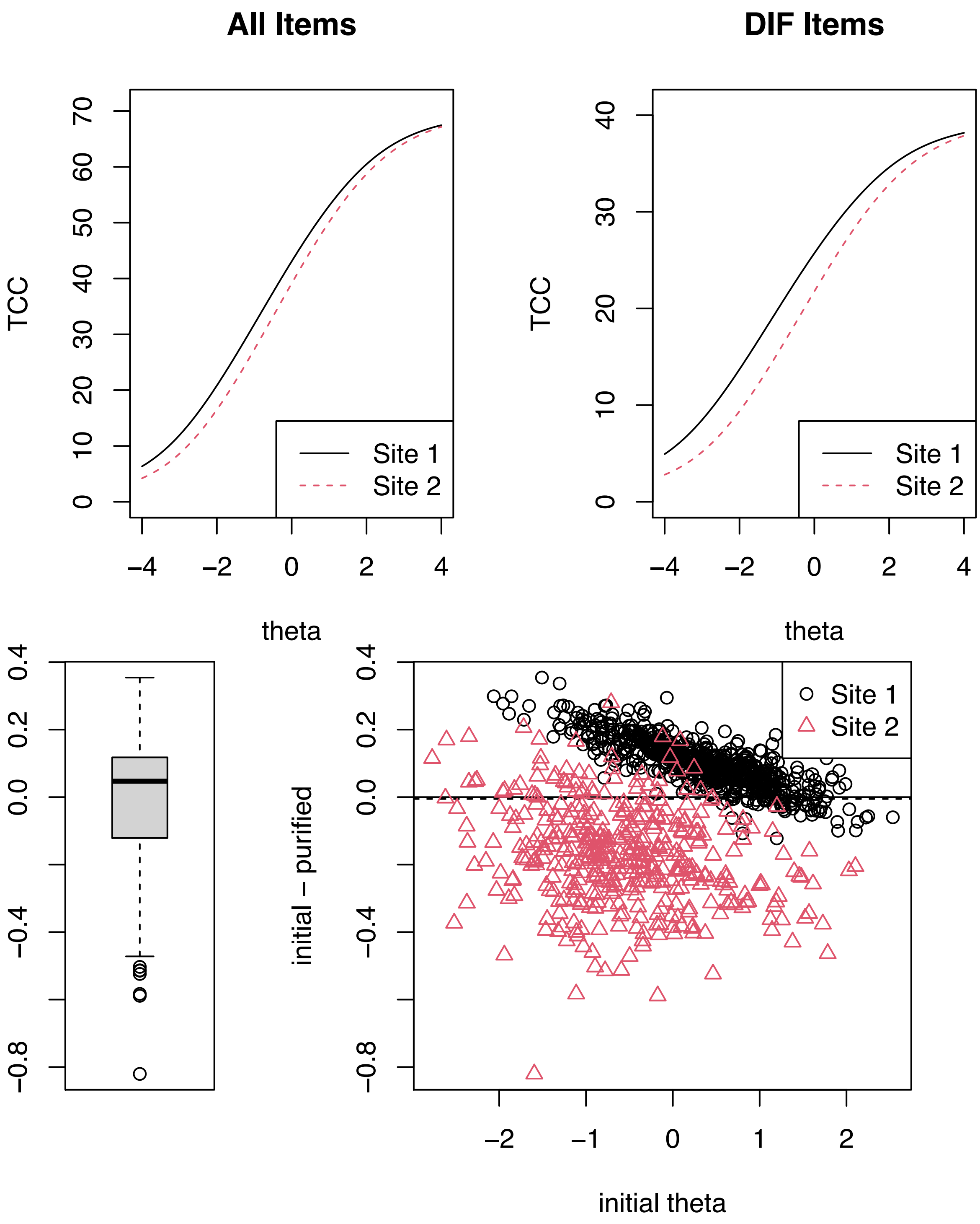

Back to top

Hurtful Rumination

Reliability: Hurtful Rumination

```
## Cronbach's alpha is 0.757.
```

```
## Mean item-total correlation is 0.385.
## If each item were dropped:
##     raw_alpha std.alpha G6(smc) average_r S/N alpha se  var.r med.r
## Q39      0.68      0.68    0.64      0.35 2.1    0.021 0.0095  0.33
## Q3       0.74      0.74    0.69      0.41 2.8    0.017 0.0081  0.43
## Q38      0.70      0.70    0.64      0.37 2.3    0.020 0.0064  0.38
## Q76      0.72      0.72    0.67      0.40 2.6    0.018 0.0042  0.40
## Q85      0.73      0.73    0.68      0.40 2.7    0.018 0.0096  0.42
```

Unidimensionality: Hurtful Rumination

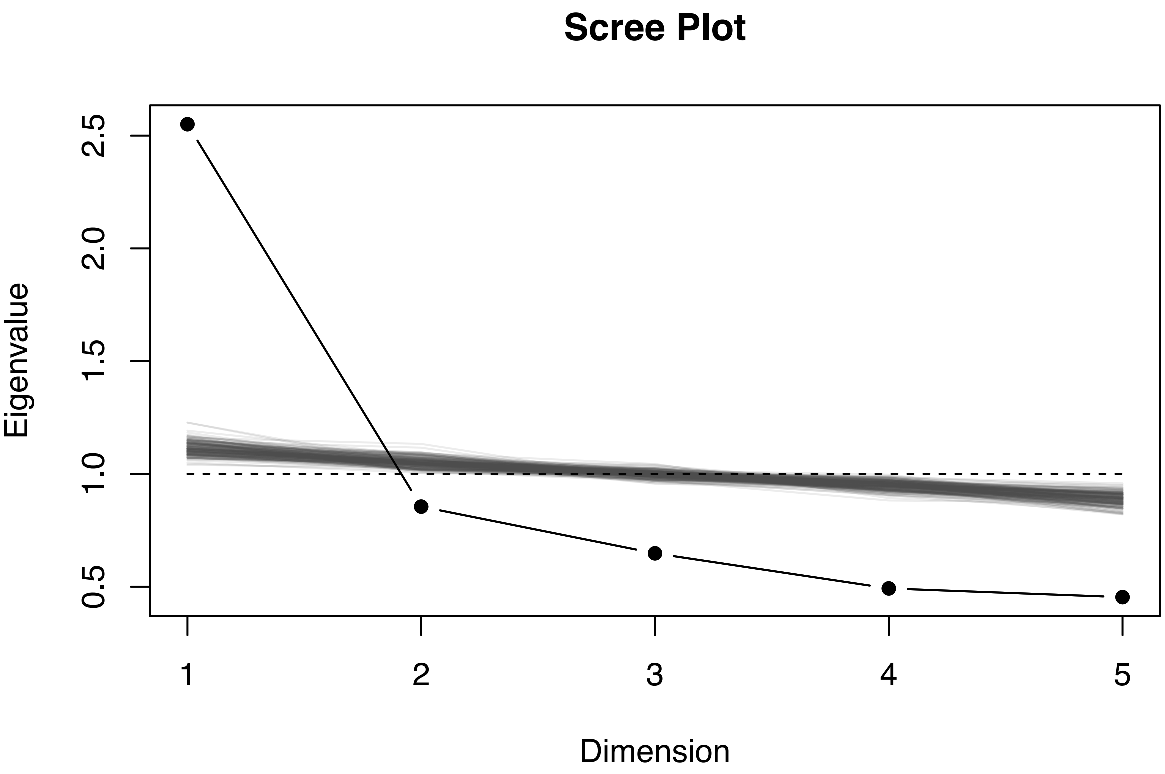


```
## [1] "Ratio of first to second eigenvalues: 2.982"

## [1] 2.5504445 0.8552244 0.6480348 0.4924785 0.4538178

## Factor Analysis using method =  minres
## Call: fa(r = grm_obj$X)
## Standardized loadings (pattern matrix) based upon correlation matrix
##      MR1   h2   u2 com
## Q39 0.73 0.54 0.46   1
## Q3  0.53 0.28 0.72   1
## Q38 0.68 0.46 0.54   1
## Q76 0.59 0.35 0.65   1
## Q85 0.57 0.33 0.67   1
##
##                 MR1
## SS loadings    1.96
## Proportion Var 0.39
##
## Mean item complexity =  1
```

```
## Test of the hypothesis that 1 factor is sufficient.
##
## The degrees of freedom for the null model are  10  and the objective function was  1.15 with Chi Squa
## The degrees of freedom for the model are 5  and the objective function was  0.09
##
## The root mean square of the residuals (RMSR) is  0.06
## The df corrected root mean square of the residuals is  0.09
##
## The harmonic number of observations is  598 with the empirical chi square  43.99  with prob <  2.3e-0
## The total number of observations was  619  with Likelihood Chi Square =  57.54  with prob <  3.9e-11
##
## Tucker Lewis Index of factoring reliability =  0.85
## RMSEA index =  0.13  and the 90 % confidence intervals are  0.101 0.162
## BIC =  25.4
## Fit based upon off diagonal values = 0.98
## Measures of factor score adequacy
##                                                     MR1
## Correlation of (regression) scores with factors   0.88
## Multiple R square of scores with factors          0.78
## Minimum correlation of possible factor scores     0.55
```

Graded-Response Model: Hurtful Rumination

```
##     Extrmt1 Extrmt2 Extrmt3 Extrmt4 Extrmt5 Extrmt6 Dscrmn
## Q39  -1.885  -1.123  -0.740  -0.302   0.158   0.686  2.004
## Q3   -1.547  -0.766  -0.218   0.303   1.015   1.745  1.149
## Q38  -0.918  -0.286   0.106   0.481   0.947   1.562  1.921
## Q76  -0.445   0.029   0.446   0.994   1.543   2.160  1.661
## Q85  -2.602  -2.139  -1.578  -1.182  -0.670   0.050  1.474
```

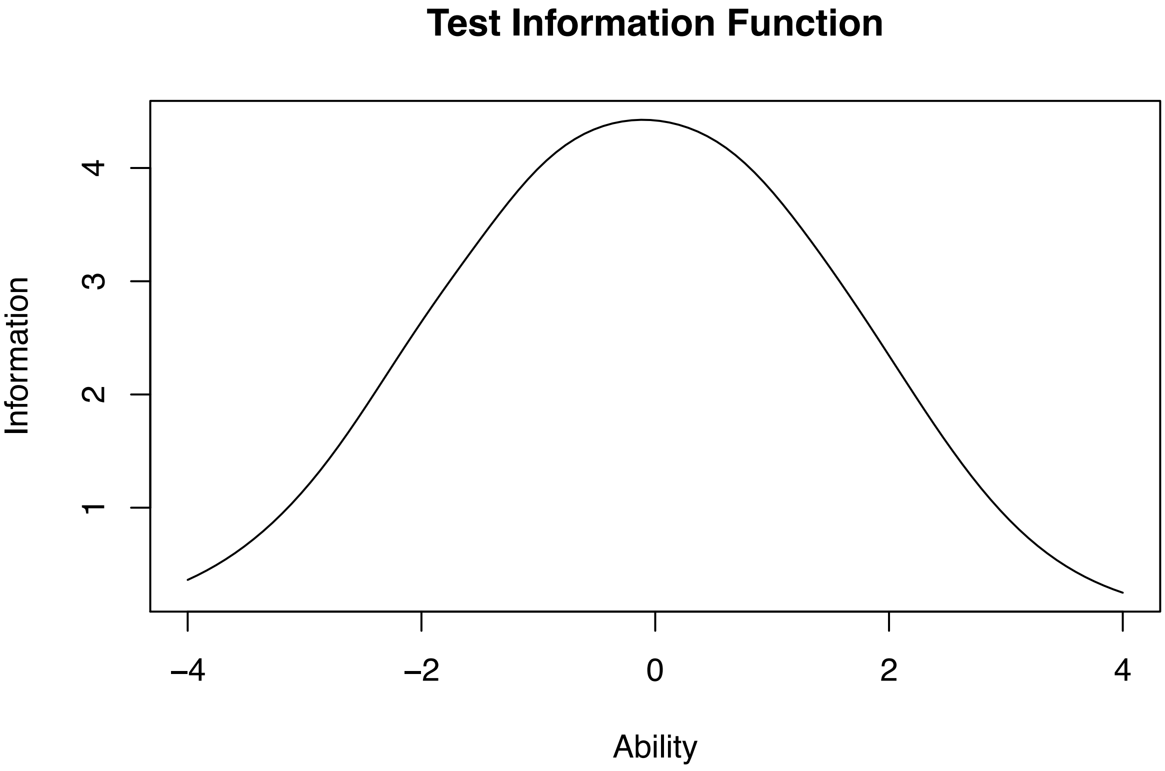

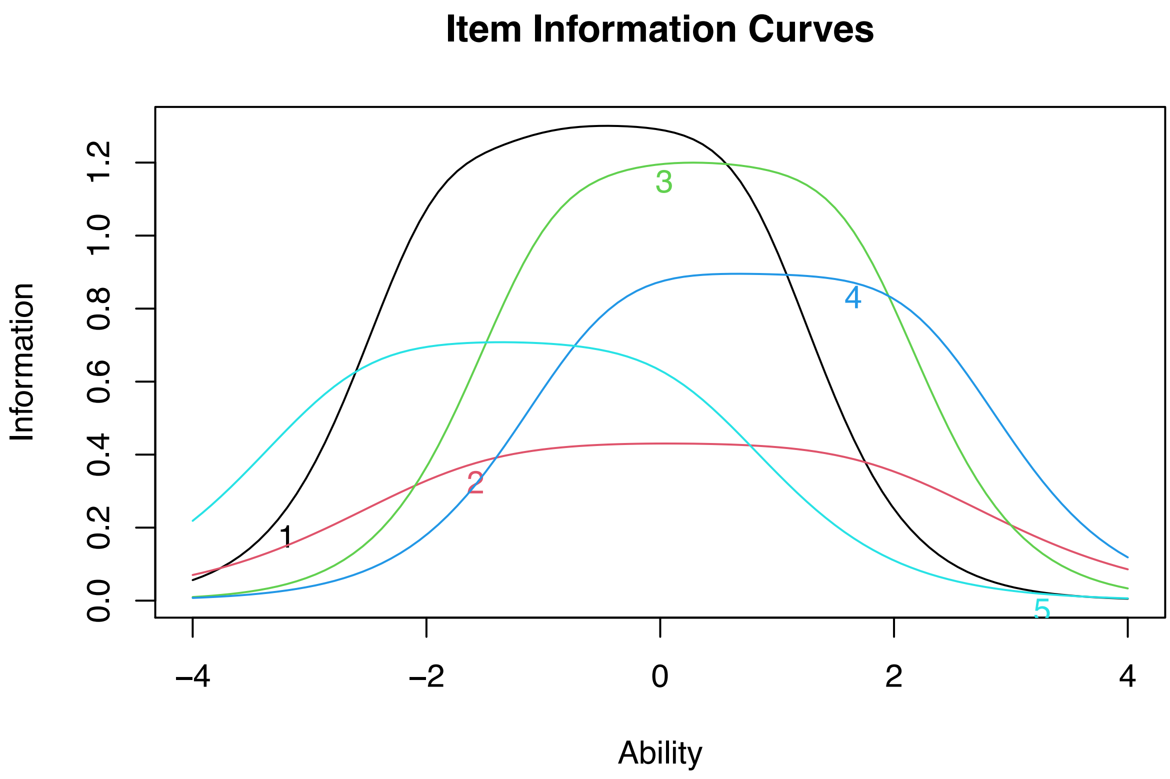

Gender-based DIF: Hurtful Rumination

```
## Call:
## lordif::lordif(resp.data = as.data.frame(sex.data), group = sex)
##
##   Number of DIF groups: 2
##
##   Number of items flagged for DIF: 2 of 5
##
##   Items flagged: 2, 5
##
##   Number of iterations for purification: 3 of 10
##
##   Detection criterion: Chisqr
##
##   Threshold: alpha = 0.01
##
##   item ncat  chi12  chi13  chi23
## 1    1    7 0.6519 0.6004 0.3662
## 2    2    7 0.0000 0.0000 0.7387
## 3    3    7 0.2967 0.1945 0.1393
## 4    4    7 0.6118 0.4465 0.2444
## 5    5    7 0.0003 0.0010 0.3226
```

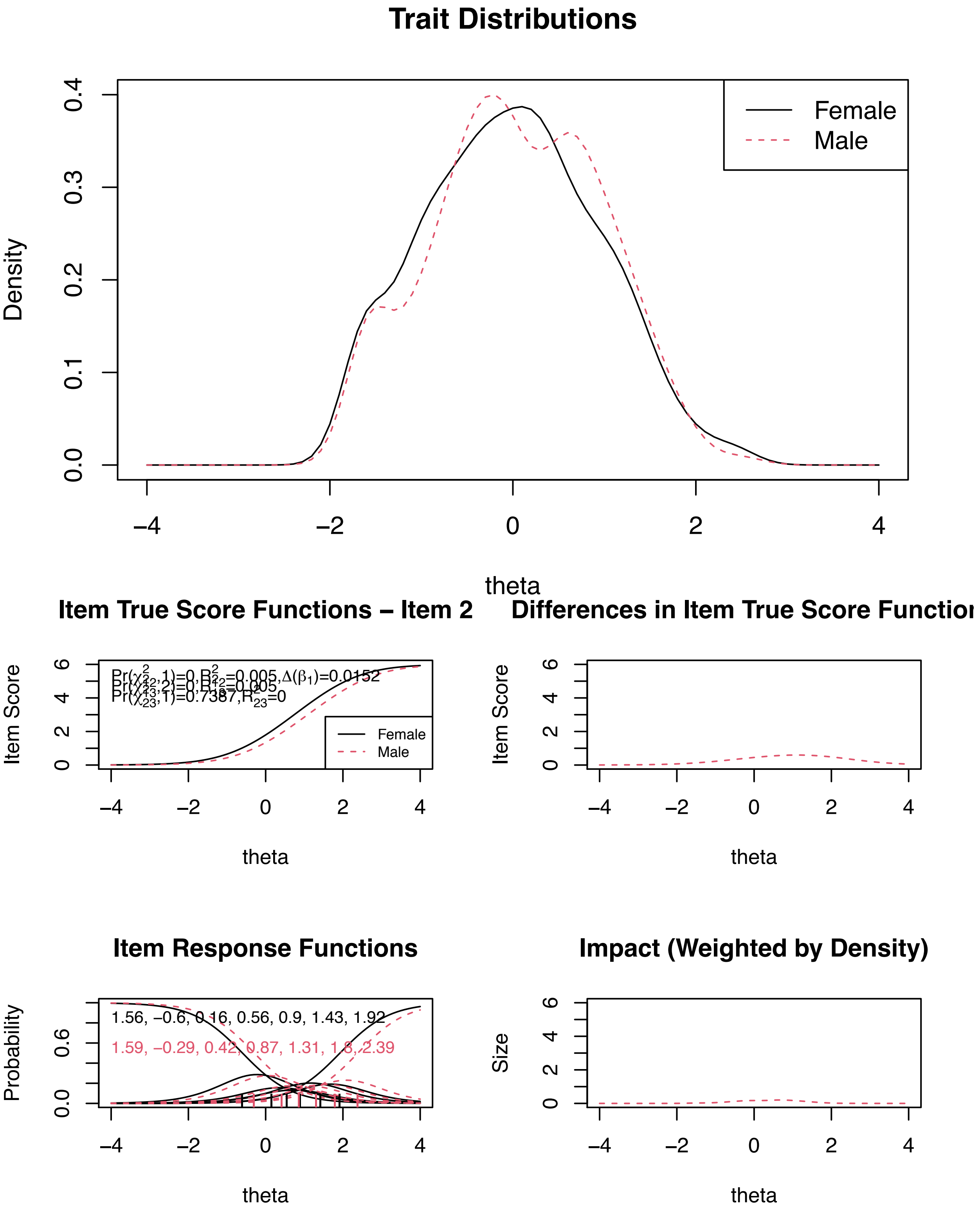
Trait Distributions
Density
theta
Female
Male
Item True Score Functions – Item 2
Differences in Item True Score Functio
Item Score
1.56, −0.6, 0.16, 0.56, 0.9, 1.43, 1.92
1.59, −0.29, 0.42, 0.87, 1.31, 1.8, 2.39
Item Response Functions
Impact (Weighted by Density)
Probability
Size

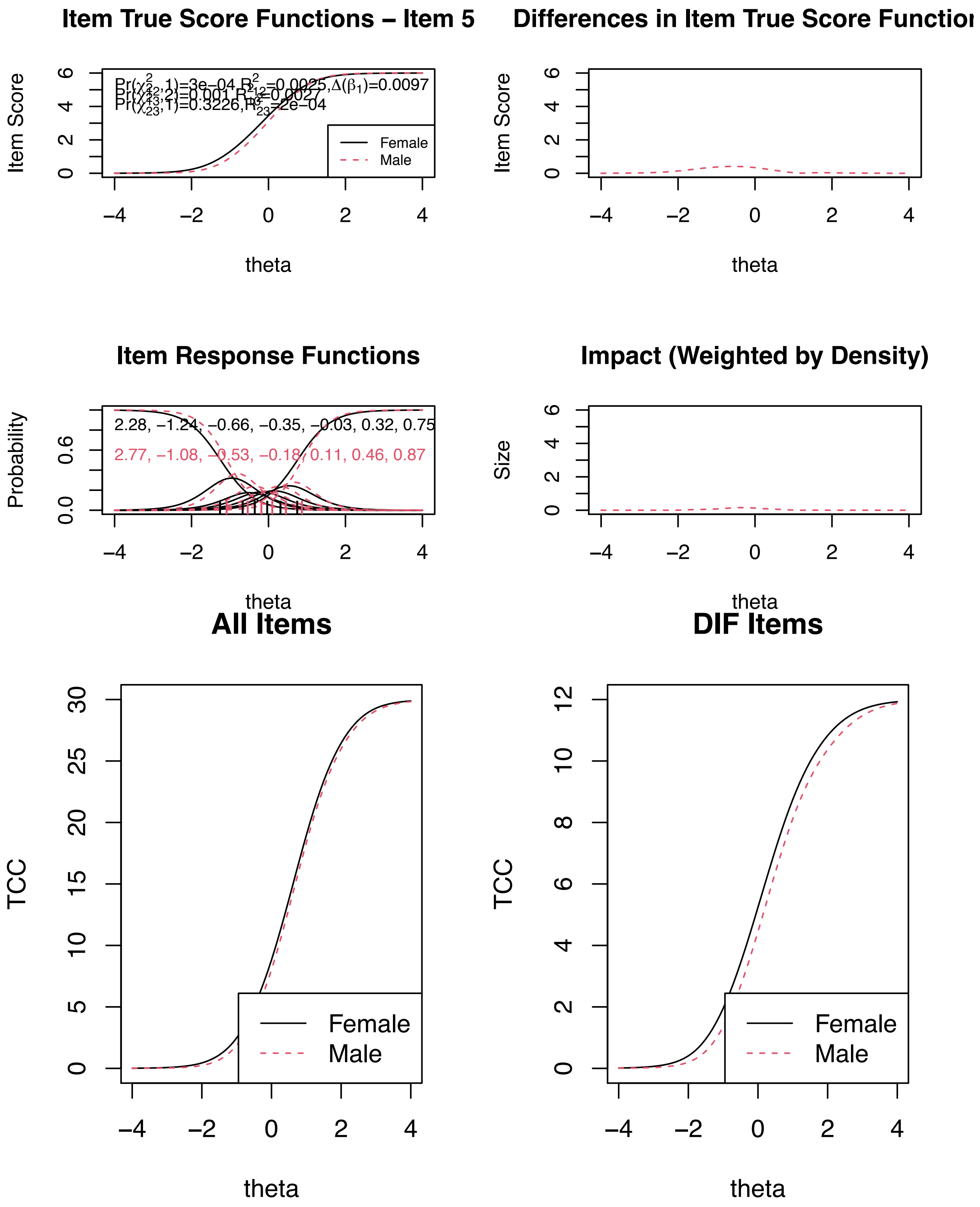
Item True Score Functions – Item 5
Differences in Item True Score Functio
Item Response Functions
Impact (Weighted by Density)
All Items
DIF Items
Item Score
Probability
Size
TCC
theta
Female
Male
2.28, −1.24, −0.66, −0.35, −0.03, 0.32, 0.75
2.77, −1.08, −0.53, −0.18, 0.11, 0.46, 0.87

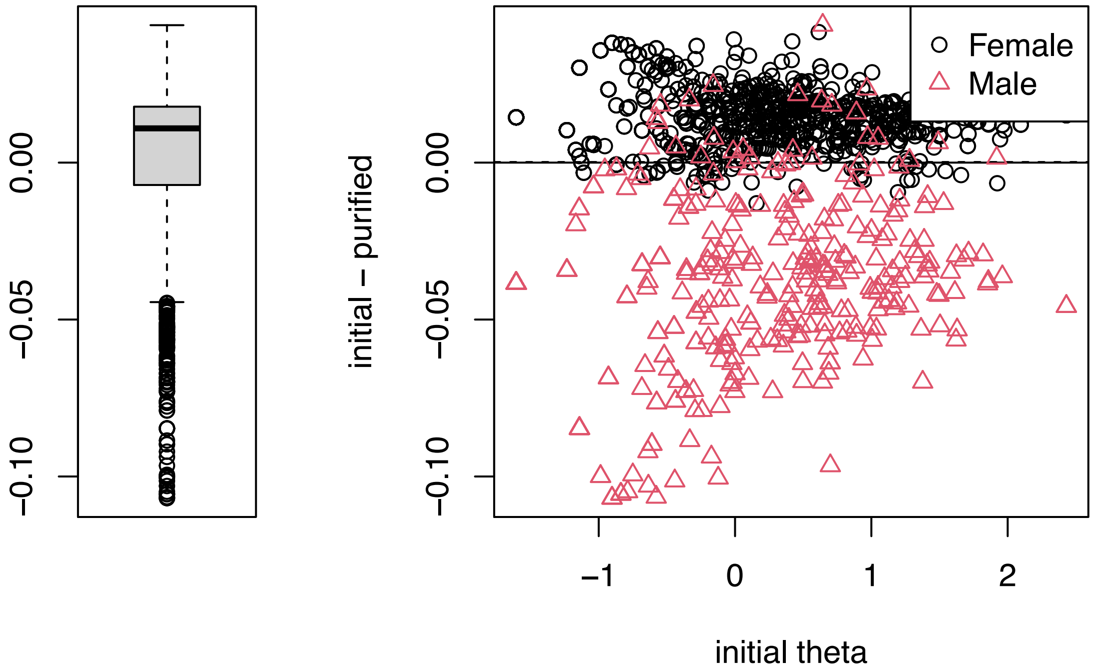

Sample-based DIF: Hurtful Rumination

```
## Call:
## lordif::lordif(resp.data = as.data.frame(sample.data), group = clinYN)
##
##   Number of DIF groups: 2
##
##   Number of items flagged for DIF: 1 of 5
##
##   Items flagged: 3
##
##   Number of iterations for purification: 2 of 10
##
##   Detection criterion: Chisqr
##
##   Threshold: alpha = 0.01
##
##   item ncat  chi12  chi13  chi23
## 1    1    7 0.4392 0.6920 0.7104
## 2    2    7 0.4665 0.1786 0.0877
## 3    3    7 0.0000 0.0000 0.6195
## 4    4    7 0.3878 0.6171 0.6394
## 5    5    7 0.3260 0.1098 0.0631
```

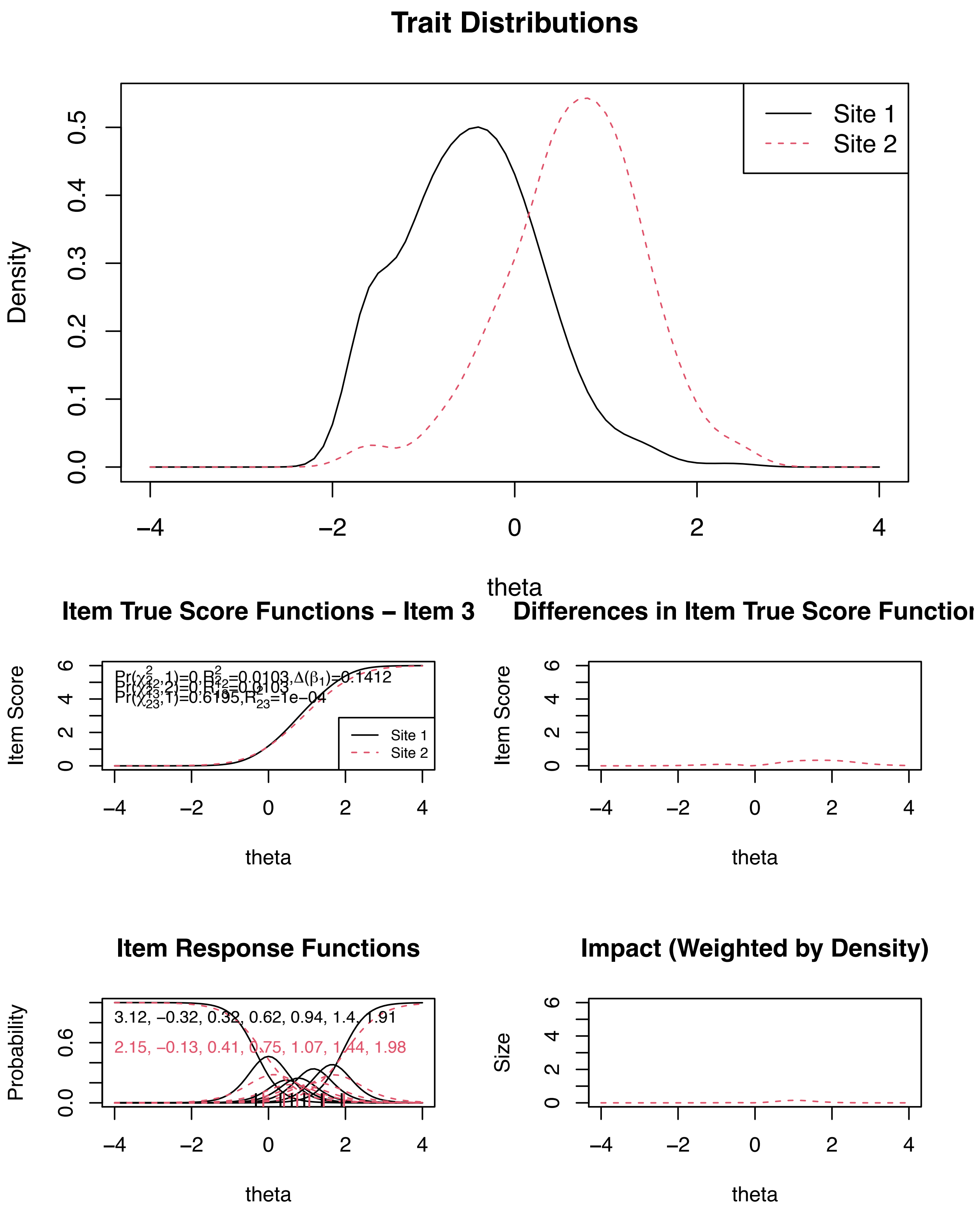
Trait Distributions
Density
theta
Site 1
Site 2
Item True Score Functions – Item 3
Item Score
Differences in Item True Score Functio
Item Response Functions
Probability
3.12, −0.32, 0.32, 0.62, 0.94, 1.4, 1.91
2.15, −0.13, 0.41, 0.75, 1.07, 1.44, 1.98
Impact (Weighted by Density)
Size

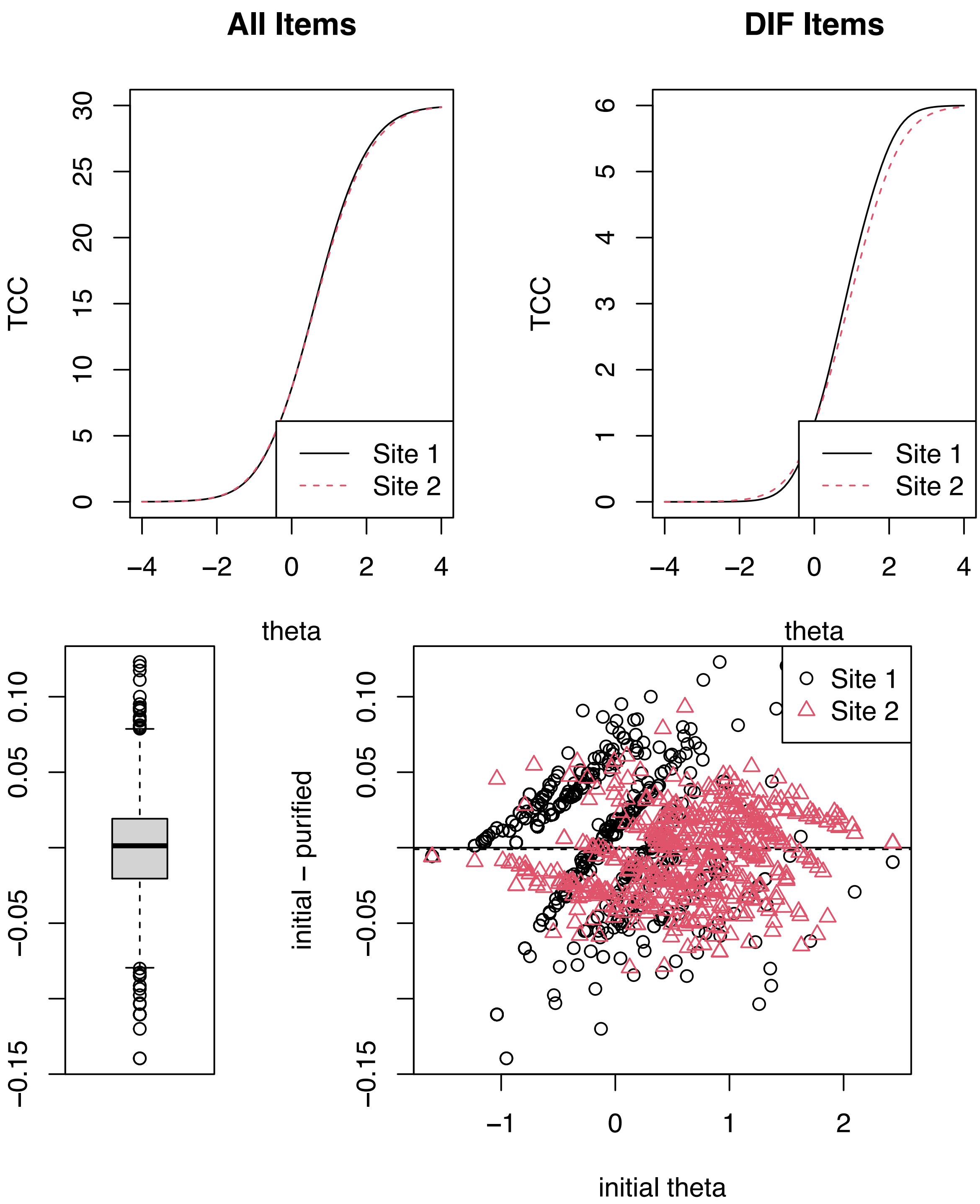

Back to top

Social Role Functioning

Reliability: Social Role Functioning

```
## Cronbach's alpha is 0.676.
## Mean item-total correlation is 0.346.
```

```
## If each item were dropped:
##      raw_alpha std.alpha G6(smc) average_r S/N alpha se   var.r med.r
## Q87-      0.55      0.55    0.45      0.29 1.2    0.032 0.00078  0.27
## Q5        0.51      0.51    0.41      0.26 1.0    0.034 0.00066  0.27
## Q22       0.66      0.67    0.65      0.40 2.0    0.024 0.07214  0.27
## Q27-      0.70      0.70    0.67      0.43 2.3    0.021 0.05867  0.32
```

Unidimensionality: Social Role Functioning

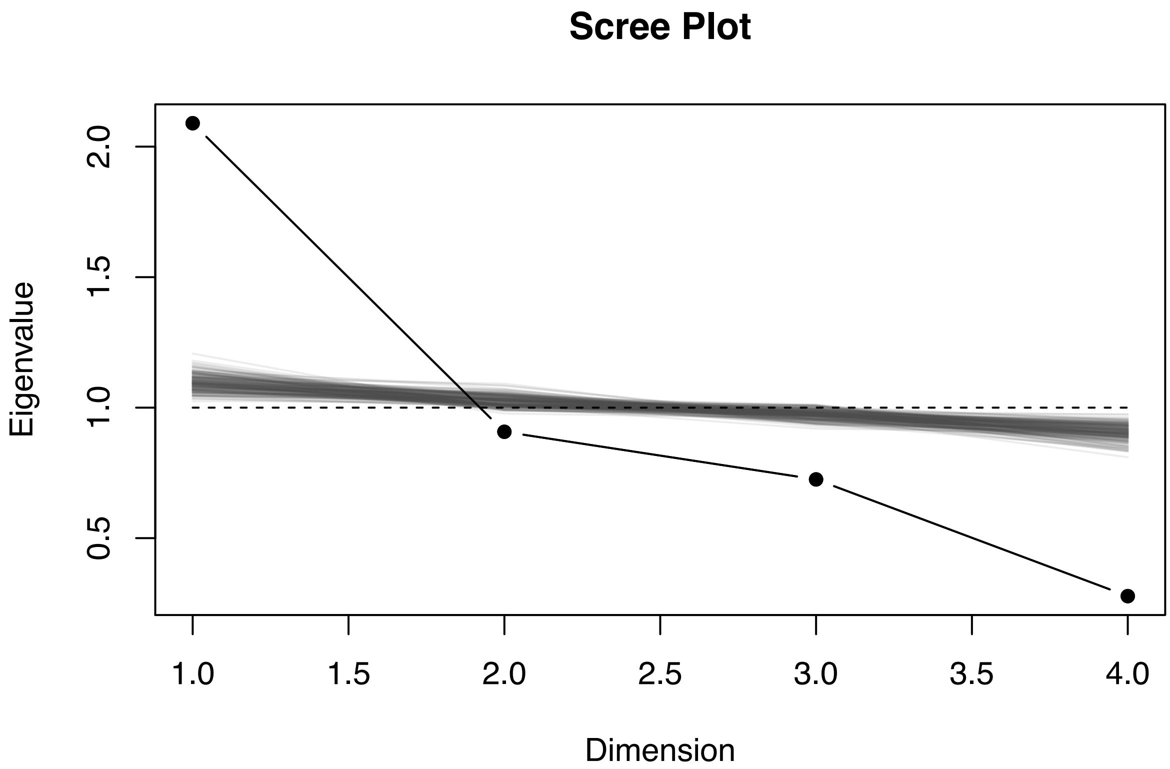


```
## [1] "Ratio of first to second eigenvalues: 2.303"

## [1] 2.0896950 0.9074757 0.7249887 0.2778405

## Factor Analysis using method =  minres
## Call: fa(r = grm_obj$X)
## Standardized loadings (pattern matrix) based upon correlation matrix
##       MR1   h2   u2 com
## Q87 -0.77 0.59 0.41   1
## Q5   0.90 0.81 0.19   1
## Q22  0.39 0.15 0.85   1
## Q27 -0.34 0.12 0.88   1
##
##                 MR1
## SS loadings    1.67
## Proportion Var 0.42
##
## Mean item complexity =  1
## Test of the hypothesis that 1 factor is sufficient.
##
## The degrees of freedom for the null model are  6  and the objective function was  0.96 with Chi Squa
```

```
## The degrees of freedom for the model are 2  and the objective function was  0.04
##
## The root mean square of the residuals (RMSR) is  0.06
## The df corrected root mean square of the residuals is  0.11
##
## The harmonic number of observations is  540 with the empirical chi square  23.88  with prob <  6.5e-[illegible]
## The total number of observations was  619  with Likelihood Chi Square =  23.98  with prob <  6.2e-06
##
## Tucker Lewis Index of factoring reliability =  0.887
## RMSEA index =  0.133  and the 90 % confidence intervals are  0.089 0.184
## BIC =  11.12
## Fit based upon off diagonal values = 0.98
## Measures of factor score adequacy
##                                                    MR1
## Correlation of (regression) scores with factors   0.92
## Multiple R square of scores with factors          0.85
## Minimum correlation of possible factor scores     0.71
```

Graded-Response Model: Social Role Functioning

```
##     Extrmt1 Extrmt2 Extrmt3 Extrmt4 Extrmt5 Extrmt6 Dscrmn
## Q87   1.033   0.562   0.147  -0.403  -0.823  -1.403 -2.866
## Q5   -1.565  -1.001  -0.605  -0.038   0.382   0.821  4.237
## Q22  -1.504  -0.242   0.788   1.745   2.438   3.341  0.787
## Q27   3.330   1.845   0.790  -0.212  -1.047  -2.401 -0.629
```

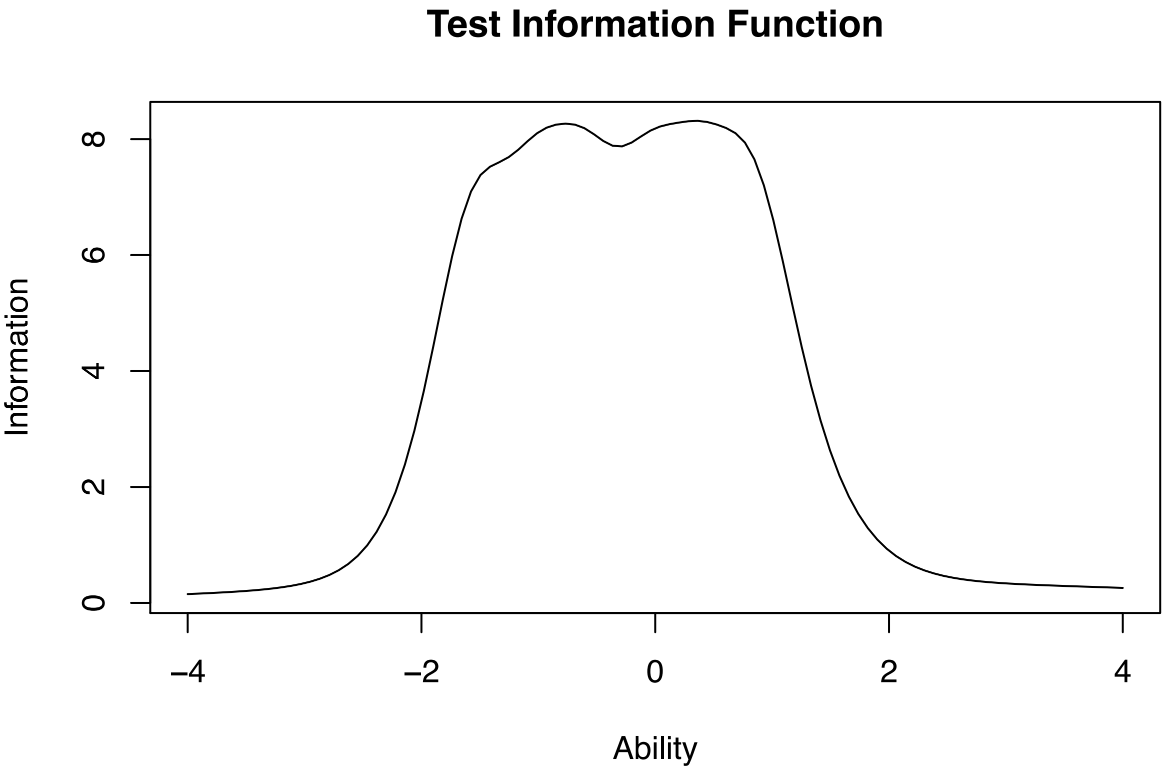

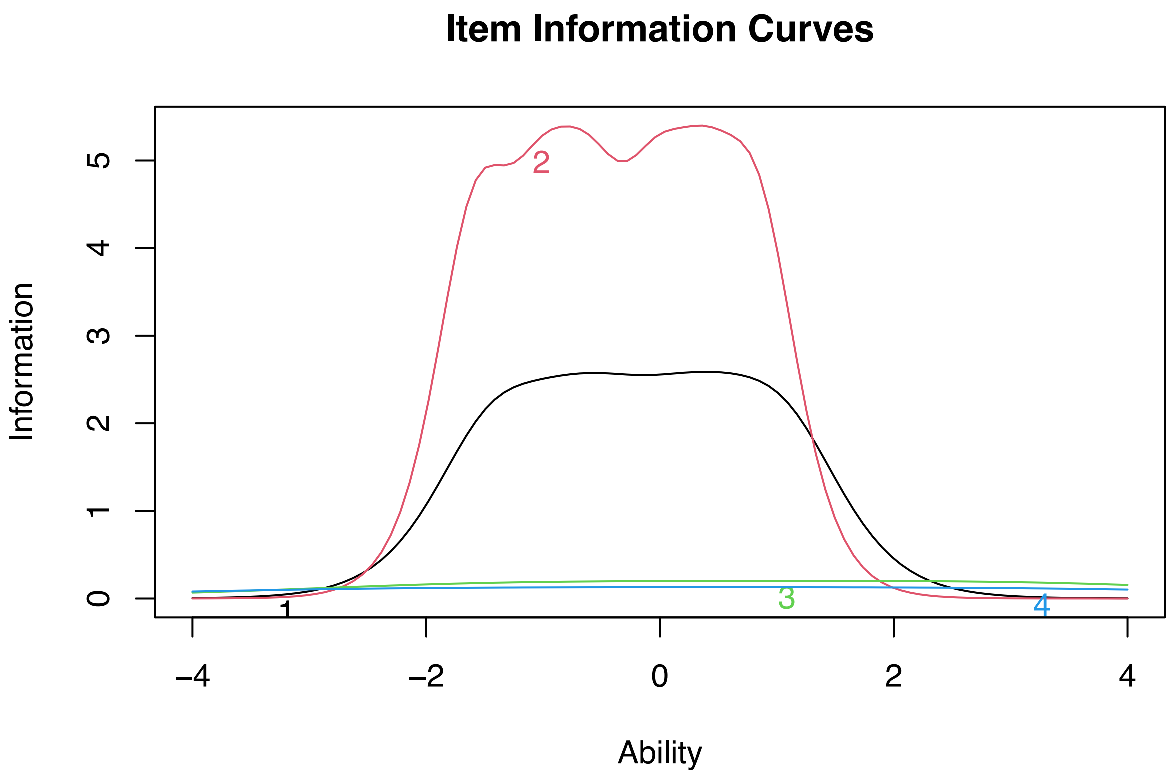

Gender-based DIF: Social Role Functioning

```
## Call:
## lordif::lordif(resp.data = as.data.frame(sex.data), group = sex)
##
##   Number of DIF groups: 2
##
##   Number of items flagged for DIF: 0 of 4
##
##   Items flagged:
##
##   Number of iterations for purification: 1 of 10
##
##   Detection criterion: Chisqr
##
##   Threshold: alpha = 0.01
```

Sample-based DIF: Social Role Functioning

```
## Call:
## lordif::lordif(resp.data = as.data.frame(sample.data), group = clinYN)
##
##   Number of DIF groups: 2
##
##   Number of items flagged for DIF: 4 of 4
##
##   Items flagged: 1, 2, 3, 4
##
```

```
##   Number of iterations for purification: 1 of 10
##
##   Detection criterion: Chisqr
##
##   Threshold: alpha = 0.01
##
##   item ncat chi12 chi13  chi23
## 1    1    7     0 0e+00 0.5205
## 2    2    7     0 1e-04 0.3929
## 3    3    7     0 0e+00 0.2994
## 4    4    7     0 2e-04 0.7270
```

Back to top

Somatic Anxiety

Reliability: Somatic Anxiety

```
## Cronbach's alpha is 0.76.
## Mean item-total correlation is 0.348.
## If each item were dropped:
##     raw_alpha std.alpha G6(smc) average_r S/N alpha se  var.r med.r
## Q3       0.72      0.72    0.70      0.34 2.6    0.018 0.0186  0.30
## Q30      0.75      0.75    0.73      0.37 3.0    0.016 0.0179  0.40
## Q47      0.77      0.77    0.74      0.40 3.3    0.015 0.0121  0.40
## Q51      0.71      0.72    0.68      0.33 2.5    0.018 0.0080  0.30
## Q53      0.70      0.70    0.68      0.32 2.4    0.019 0.0146  0.29
## Q75      0.70      0.70    0.66      0.32 2.3    0.019 0.0076  0.29
```

Unidimensionality: Somatic Anxiety

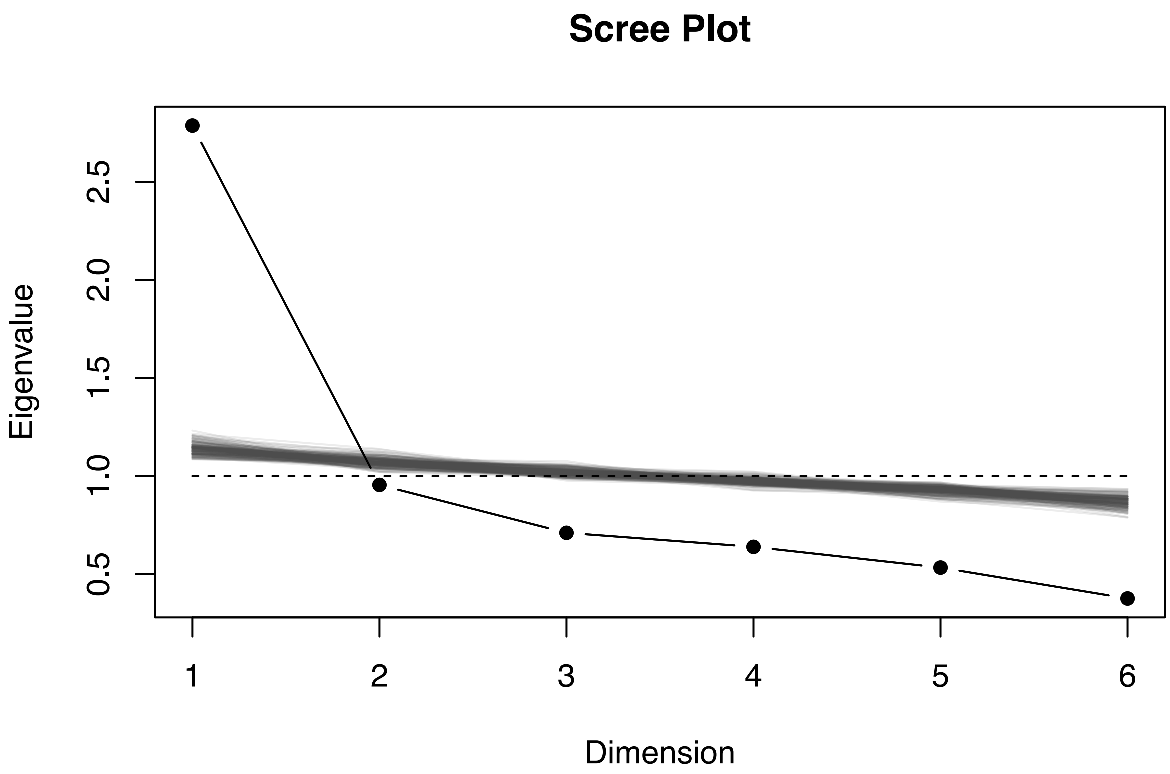


```
## [1] "Ratio of first to second eigenvalues: 2.918"

## [1] 2.7863327 0.9548338 0.7105823 0.6390692 0.5333155 0.3758666

## Factor Analysis using method =  minres
## Call: fa(r = grm_obj$X)
## Standardized loadings (pattern matrix) based upon correlation matrix
##      MR1   h2   u2 com
## Q3  0.60 0.36 0.64   1
## Q30 0.46 0.21 0.79   1
## Q47 0.38 0.14 0.86   1
## Q51 0.67 0.45 0.55   1
## Q53 0.70 0.49 0.51   1
## Q75 0.74 0.55 0.45   1
##
##                 MR1
## SS loadings    2.21
## Proportion Var 0.37
##
## Mean item complexity =  1
## Test of the hypothesis that 1 factor is sufficient.
##
## The degrees of freedom for the null model are  15  and the objective function was  1.42 with Chi Squa
## The degrees of freedom for the model are 9  and the objective function was  0.11
##
## The root mean square of the residuals (RMSR) is  0.06
## The df corrected root mean square of the residuals is  0.07
##
```

```
## The harmonic number of observations is  602 with the empirical chi square  55.62  with prob <  9.3e-[illegible]
## The total number of observations was  619  with Likelihood Chi Square =  67.87  with prob <  4e-11
##
## Tucker Lewis Index of factoring reliability =  0.885
## RMSEA index =  0.103  and the 90 % confidence intervals are  0.081 0.126
## BIC =  10.01
## Fit based upon off diagonal values = 0.98
## Measures of factor score adequacy
##                                                    MR1
## Correlation of (regression) scores with factors   0.89
## Multiple R square of scores with factors          0.80
## Minimum correlation of possible factor scores     0.60
```

Graded-Response Model: Somatic Anxiety

```
##     Extrmt1 Extrmt2 Extrmt3 Extrmt4 Extrmt5 Extrmt6 Dscrmn
## Q3   -1.333  -0.639  -0.158   0.296   0.909   1.541  1.406
## Q30  -1.129  -0.305   0.396   1.088   1.923   2.635  0.944
## Q47  -0.803   0.125   0.582   1.286   1.921   2.831  0.708
## Q51  -1.813  -1.288  -0.816  -0.330   0.250   0.852  1.950
## Q53  -0.769  -0.260   0.125   0.487   0.916   1.489  1.912
## Q75  -1.144  -0.680  -0.313   0.089   0.576   1.035  2.380
```

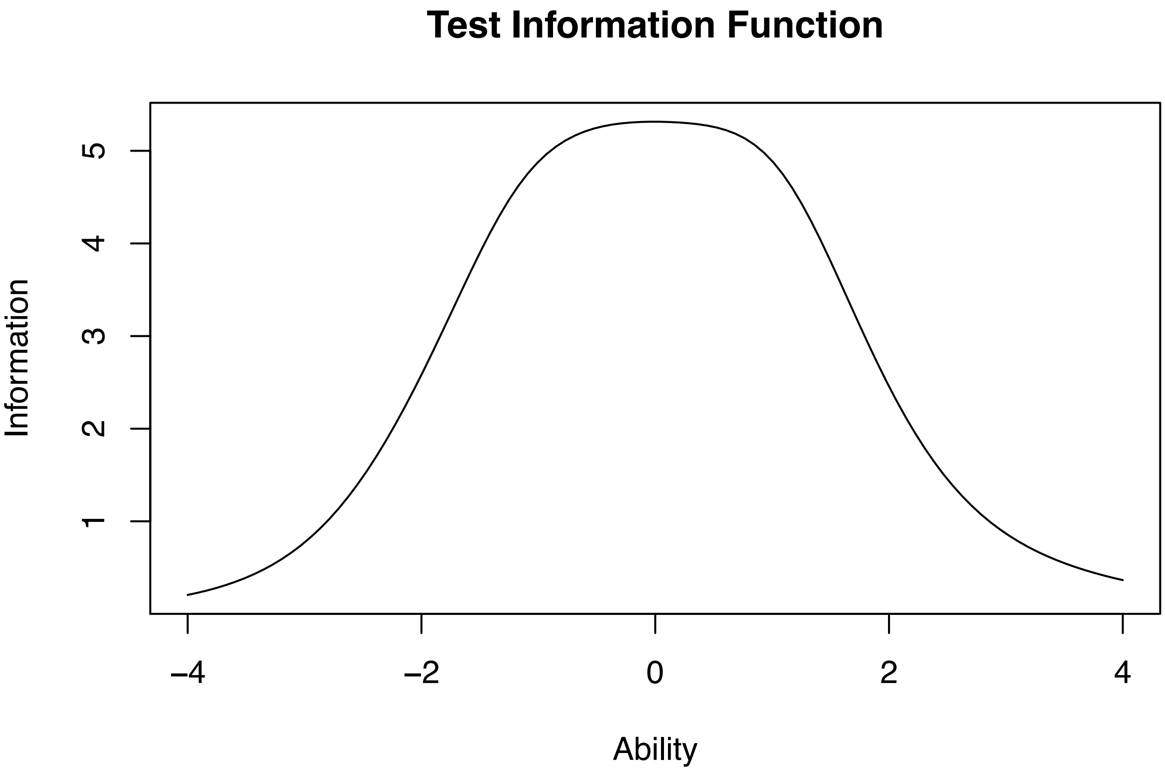

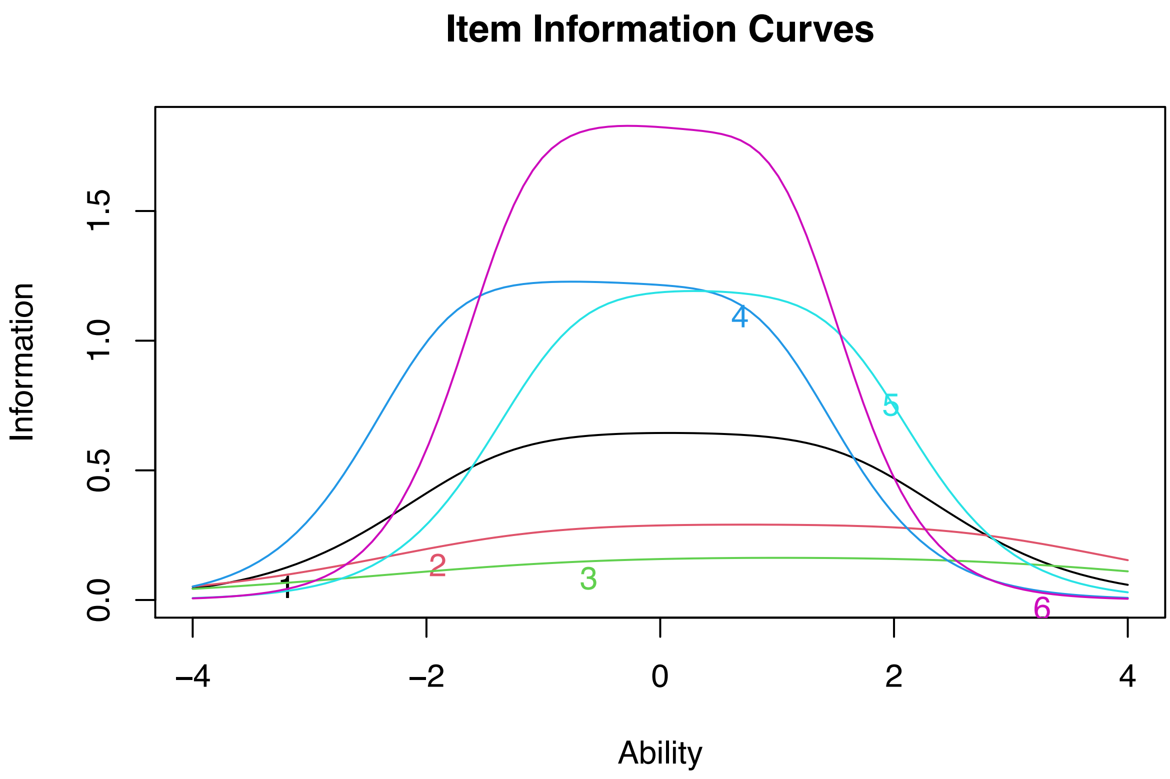

Gender-based DIF: Somatic Anxiety

```
## Call:
## lordif::lordif(resp.data = as.data.frame(sex.data), group = sex)
##
##   Number of DIF groups: 2
##
##   Number of items flagged for DIF: 2 of 6
##
##   Items flagged: 1, 4
##
##   Number of iterations for purification: 2 of 10
##
##   Detection criterion: Chisqr
##
##   Threshold: alpha = 0.01
##
##   item ncat  chi12  chi13  chi23
## 1    1    7 0.0000 0.0000 0.2851
## 2    2    7 0.2923 0.5435 0.7397
## 3    3    7 0.0357 0.0539 0.2319
## 4    4    7 0.0000 0.0000 0.0183
## 5    5    7 0.8853 0.5568 0.2835
## 6    6    7 0.6848 0.7125 0.4738
```

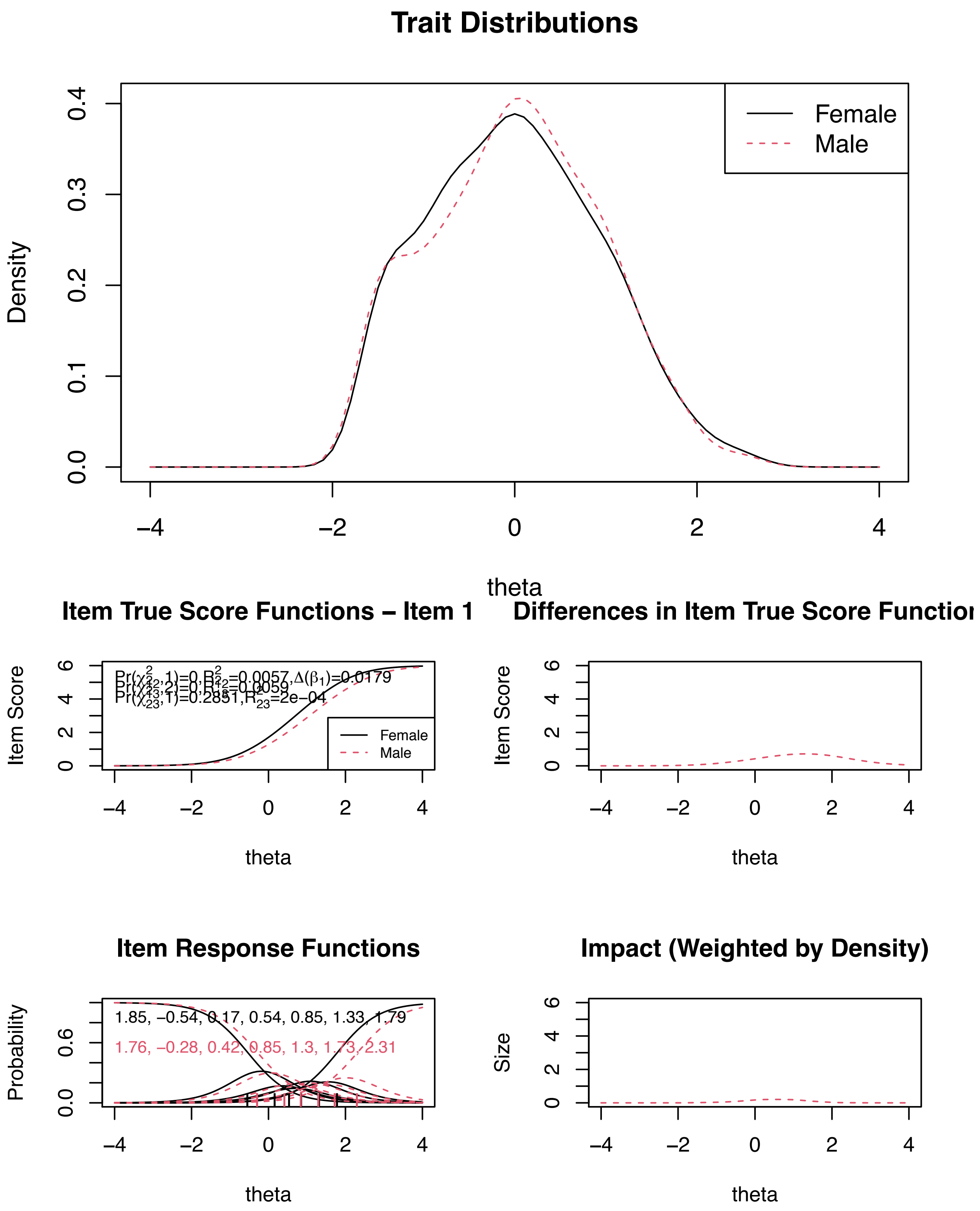

Trait Distributions
Density
Female
Male
theta
Item True Score Functions – Item 1
Item Score
Female
Male
Differences in Item True Score Functio
Item Response Functions
Probability
1.85, −0.54, 0.17, 0.54, 0.85, 1.33, 1.79
1.76, −0.28, 0.42, 0.85, 1.3, 1.73, 2.31
Impact (Weighted by Density)
Size

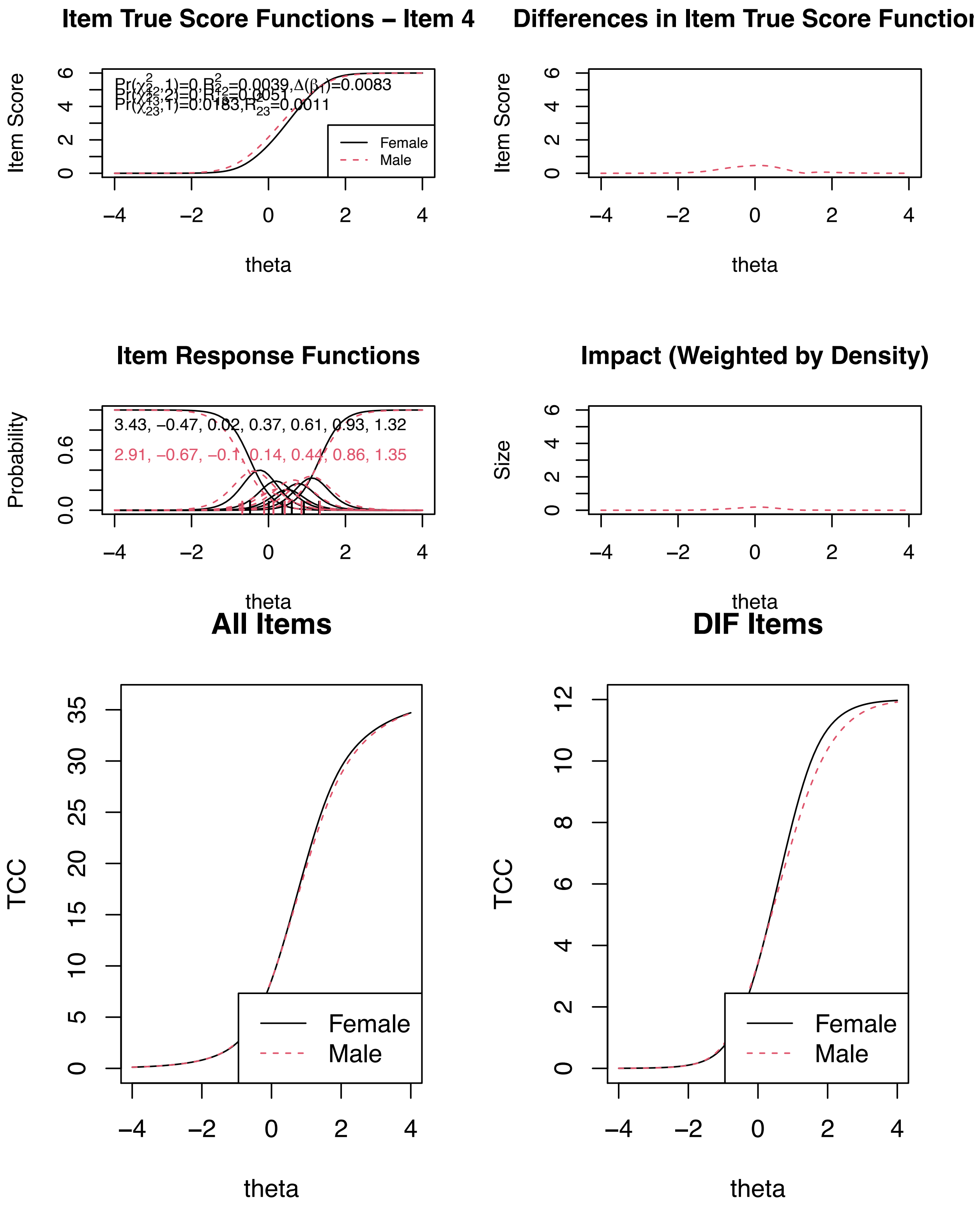
Item True Score Functions – Item 4
Differences in Item True Score Functio
Item Score
theta
Female
Male
Item Response Functions
Impact (Weighted by Density)
Probability
Size
3.43, −0.47, 0.02, 0.37, 0.61, 0.93, 1.32
2.91, −0.67, −0.1, 0.14, 0.44, 0.86, 1.35
All Items
DIF Items
TCC

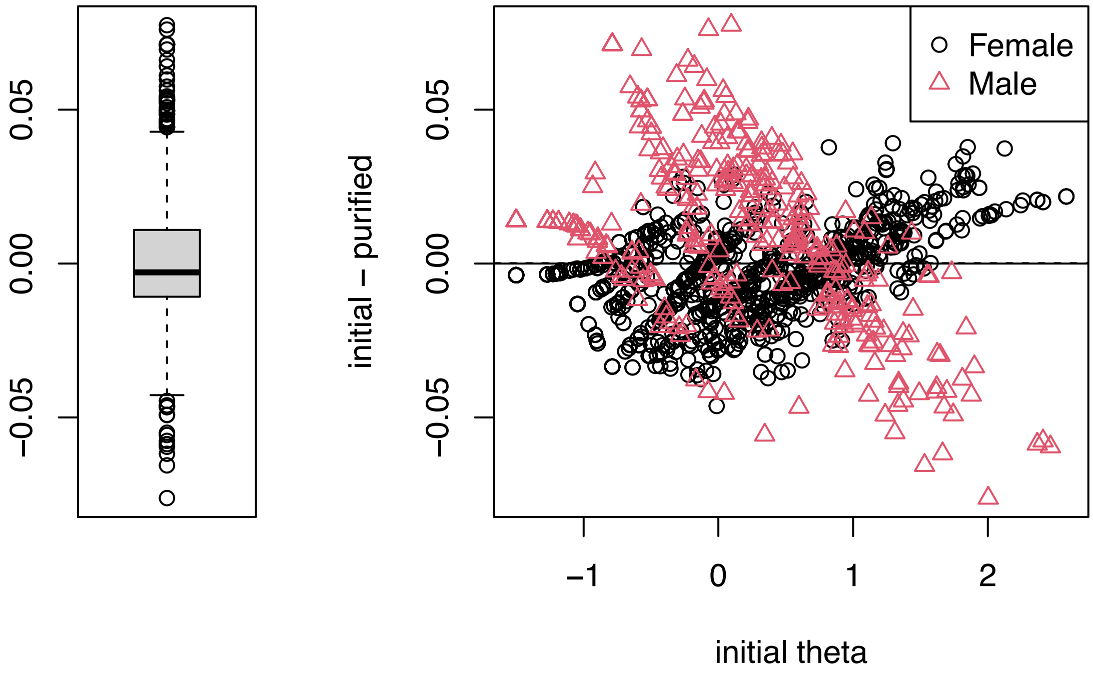

Sample-based DIF: Somatic Anxiety

```
## Call:
## lordif::lordif(resp.data = as.data.frame(sample.data), group = clinYN)
##
##   Number of DIF groups: 2
##
##   Number of items flagged for DIF: 6 of 6
##
##   Items flagged: 1, 2, 3, 4, 5, 6
##
##   Number of iterations for purification: 4 of 10
##
##   Detection criterion: Chisqr
##
##   Threshold: alpha = 0.01
##
##   item ncat chi12 chi13  chi23
## 1    1    7     0     0 0.5280
## 2    2    7     0     0 0.4738
## 3    3    7     0     0 0.6598
## 4    4    7     0     0 0.0021
## 5    5    7     0     0 0.0000
## 6    6    7     0     0 0.6639
```

Back to top

Substance Use

Reliability: Substance Use

```
## Cronbach's alpha is 0.918.
## Mean item-total correlation is 0.742.
## If each item were dropped:
##     raw_alpha std.alpha G6(smc) average_r S/N alpha se   var.r med.r
## Q35      0.89      0.89    0.85      0.74 8.4   0.0073 2.7e-03  0.76
## Q4       0.90      0.90    0.86      0.75 9.1   0.0069 6.7e-04  0.76
## Q16      0.90      0.91    0.86      0.76 9.5   0.0067 2.8e-06  0.76
## Q59      0.88      0.89    0.84      0.72 7.7   0.0081 1.8e-03  0.72
```

Unidimensionality: Substance Use

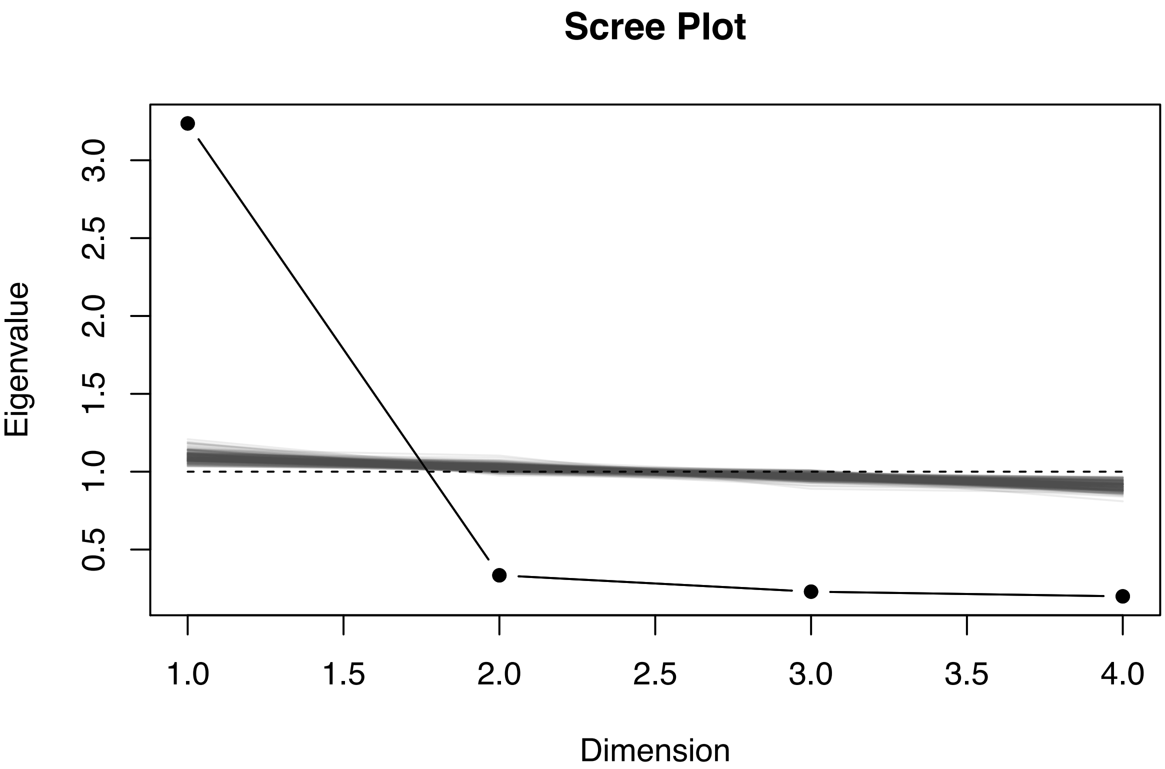


```
## [1] "Ratio of first to second eigenvalues: 9.675"

## [1] 3.2363277 0.3345069 0.2296355 0.1995300

## Factor Analysis using method =  minres
## Call: fa(r = grm_obj$X)
## Standardized loadings (pattern matrix) based upon correlation matrix
##      MR1   h2   u2 com
## Q35 0.88 0.77 0.23   1
## Q4  0.85 0.72 0.28   1
## Q16 0.83 0.69 0.31   1
## Q59 0.90 0.80 0.20   1
##
##                 MR1
## SS loadings    2.98
```

```
## Proportion Var 0.75
##
## Mean item complexity =  1
## Test of the hypothesis that 1 factor is sufficient.
##
## The degrees of freedom for the null model are  6  and the objective function was  3 with Chi Square o
## The degrees of freedom for the model are 2  and the objective function was  0.05
##
## The root mean square of the residuals (RMSR) is  0.02
## The df corrected root mean square of the residuals is  0.04
##
## The harmonic number of observations is  561 with the empirical chi square  3.54  with prob <  0.17
## The total number of observations was  619  with Likelihood Chi Square =  30.49  with prob <  2.4e-07
##
## Tucker Lewis Index of factoring reliability =  0.954
## RMSEA index =  0.152  and the 90 % confidence intervals are  0.107 0.202
## BIC =  17.63
## Fit based upon off diagonal values = 1
## Measures of factor score adequacy
##                                                    MR1
## Correlation of (regression) scores with factors   0.96
## Multiple R square of scores with factors          0.92
## Minimum correlation of possible factor scores     0.85
```

Graded-Response Model: Substance Use

```
##     Extrmt1 Extrmt2 Extrmt3 Extrmt4 Extrmt5 Extrmt6 Dscrmn
## Q35  -0.614  -0.949  -1.146  -1.468  -1.650  -1.915 -3.567
## Q4   -0.968  -1.313  -1.521  -1.707  -1.919  -2.193 -3.321
## Q16  -0.795  -1.092  -1.254  -1.434  -1.590  -1.793 -2.971
## Q59  -0.612  -0.878  -1.022  -1.146  -1.423  -1.605 -3.849
```

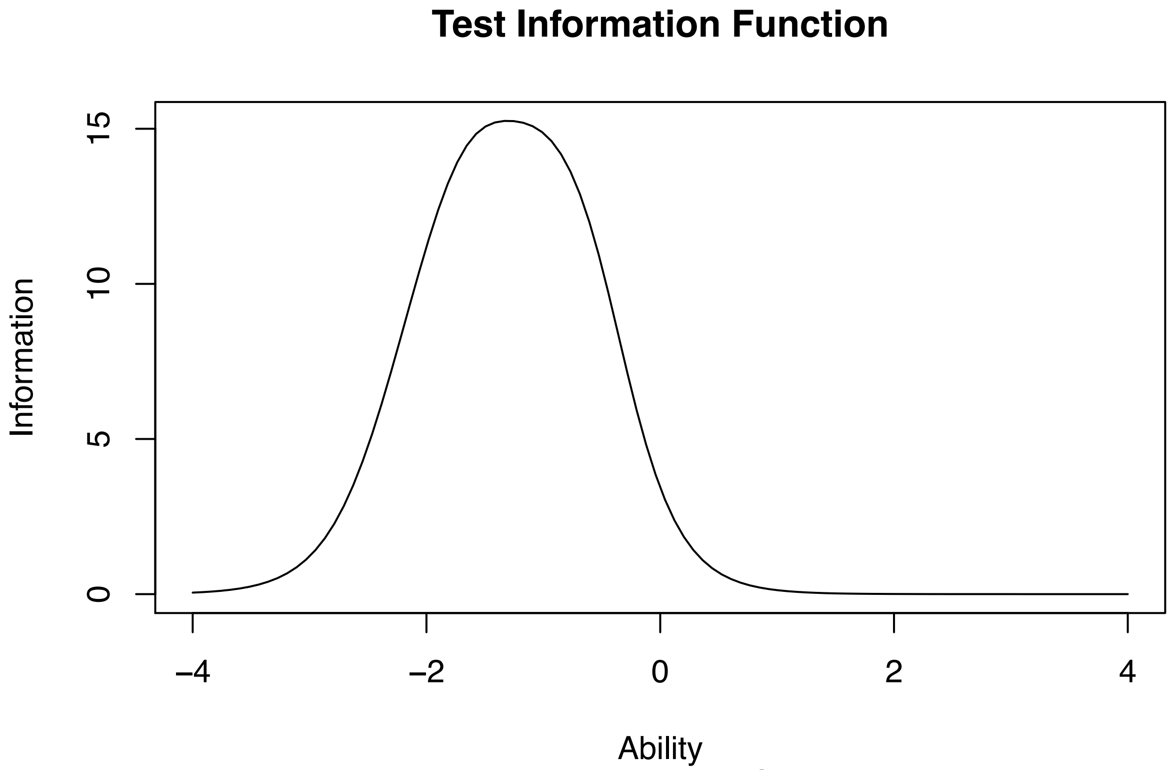

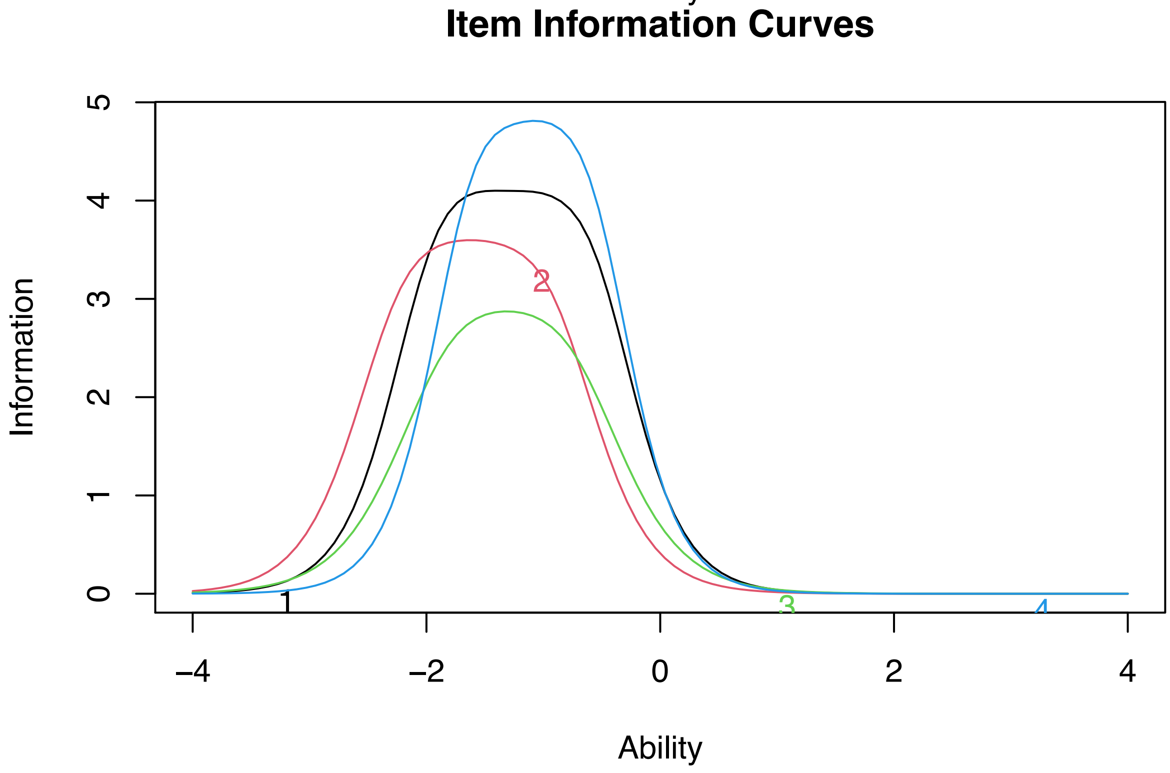

Gender-based DIF: Substance Use

```
## Call:
## lordif::lordif(resp.data = as.data.frame(sex.data), group = sex)
```

```
##
##   Number of DIF groups: 2
##
##   Number of items flagged for DIF: 0 of 4
##
##   Items flagged:
##
##   Number of iterations for purification: 1 of 10
##
##   Detection criterion: Chisqr
##
##   Threshold: alpha = 0.01
```

Sample-based DIF: Substance Use

```
## Call:
## lordif::lordif(resp.data = as.data.frame(sample.data), group = clinYN)
##
##   Number of DIF groups: 2
##
##   Number of items flagged for DIF: 4 of 4
##
##   Items flagged: 1, 2, 3, 4
##
##   Number of iterations for purification: 1 of 10
##
##   Detection criterion: Chisqr
##
##   Threshold: alpha = 0.01
##
##   item ncat  chi12  chi13  chi23
## 1    1    4 0.0002 0.0000 0.0079
## 2    2    5 0.0024 0.0004 0.0106
## 3    3    4 0.0043 0.0140 0.5304
## 4    4    7 0.0045 0.0092 0.2518
```

Back to top

Suicide Risk

Reliability: Suicide Risk

```
## Cronbach's alpha is 0.678.
## Mean item-total correlation is 0.343.
## If each item were dropped:
##     raw_alpha std.alpha G6(smc) average_r S/N alpha se   var.r med.r
## Q44      0.68      0.68    0.59      0.42 2.1    0.034 0.00058  0.42
## Q19      0.63      0.63    0.54      0.36 1.7    0.040 0.00507  0.35
## Q77      0.58      0.57    0.50      0.31 1.3    0.046 0.01859  0.35
## Q79      0.54      0.54    0.46      0.28 1.2    0.049 0.01391  0.30
```

Unidimensionality: Suicide Risk

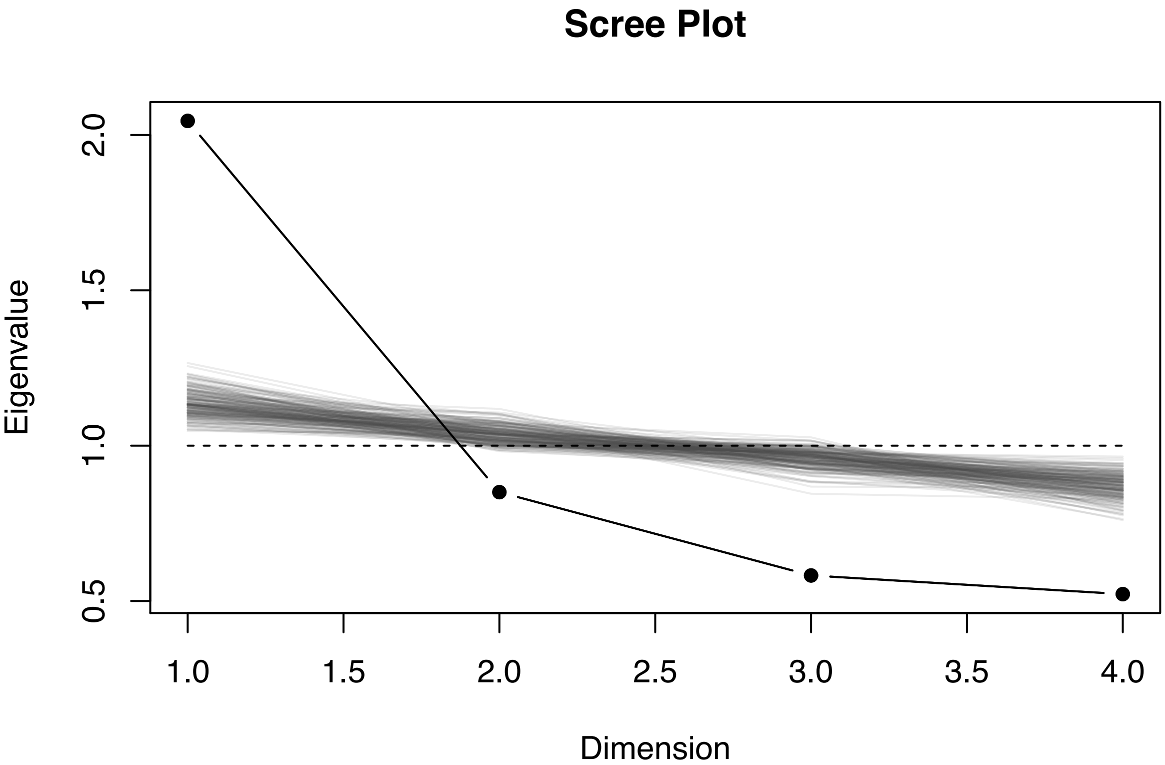


```
## [1] "Ratio of first to second eigenvalues: 2.405"

## [1] 2.0452860 0.8503631 0.5821785 0.5221724

## Factor Analysis using method =  minres
## Call: fa(r = grm_obj$X)
## Standardized loadings (pattern matrix) based upon correlation matrix
##      MR1   h2   u2 com
## Q44 0.43 0.18 0.82   1
## Q19 0.55 0.30 0.70   1
## Q77 0.65 0.42 0.58   1
## Q79 0.74 0.54 0.46   1
##
##                 MR1
## SS loadings    1.45
## Proportion Var 0.36
##
## Mean item complexity =  1
## Test of the hypothesis that 1 factor is sufficient.
##
## The degrees of freedom for the null model are  6  and the objective function was  0.64 with Chi Squa
## The degrees of freedom for the model are 2  and the objective function was  0.03
##
## The root mean square of the residuals (RMSR) is  0.04
## The df corrected root mean square of the residuals is  0.07
##
## The harmonic number of observations is  257 with the empirical chi square  5.64  with prob <  0.06
## The total number of observations was  257  with Likelihood Chi Square =  6.47  with prob <  0.039
```

```
##
## Tucker Lewis Index of factoring reliability =  0.914
## RMSEA index =  0.093  and the 90 % confidence intervals are  0.018 0.178
## BIC =  -4.63
## Fit based upon off diagonal values = 0.99
## Measures of factor score adequacy
##                                                    MR1
## Correlation of (regression) scores with factors   0.85
## Multiple R square of scores with factors          0.72
## Minimum correlation of possible factor scores     0.44
```

Graded-Response Model: Suicide Risk

```
##     Extrmt1 Extrmt2 Extrmt3 Extrmt4 Extrmt5 Extrmt6 Dscrmn
## Q44  -4.210  -2.338  -1.451  -0.879  -0.197   0.899  0.819
## Q19  -1.722  -0.994  -0.298   0.401   1.216   1.914  1.399
## Q77  -0.943  -0.080   0.352   0.828   1.304   1.857  1.729
## Q79  -0.313   0.264   0.720   1.025   1.459   1.933  1.798
```

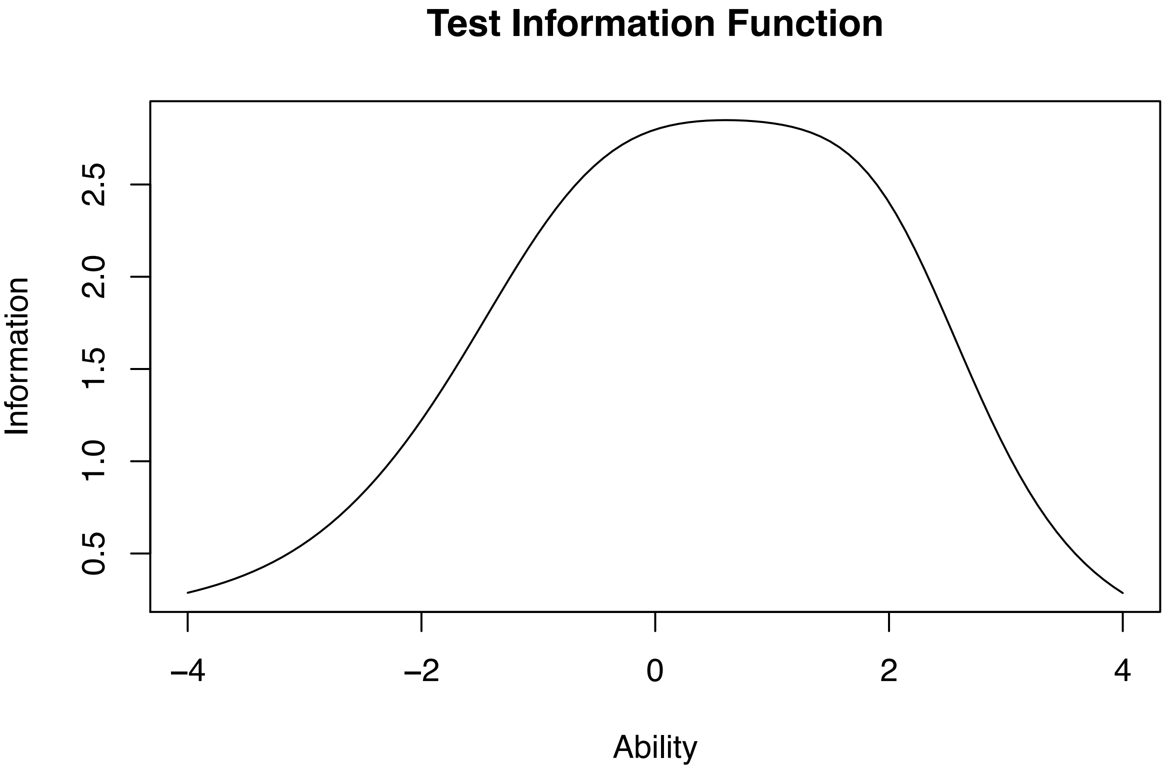

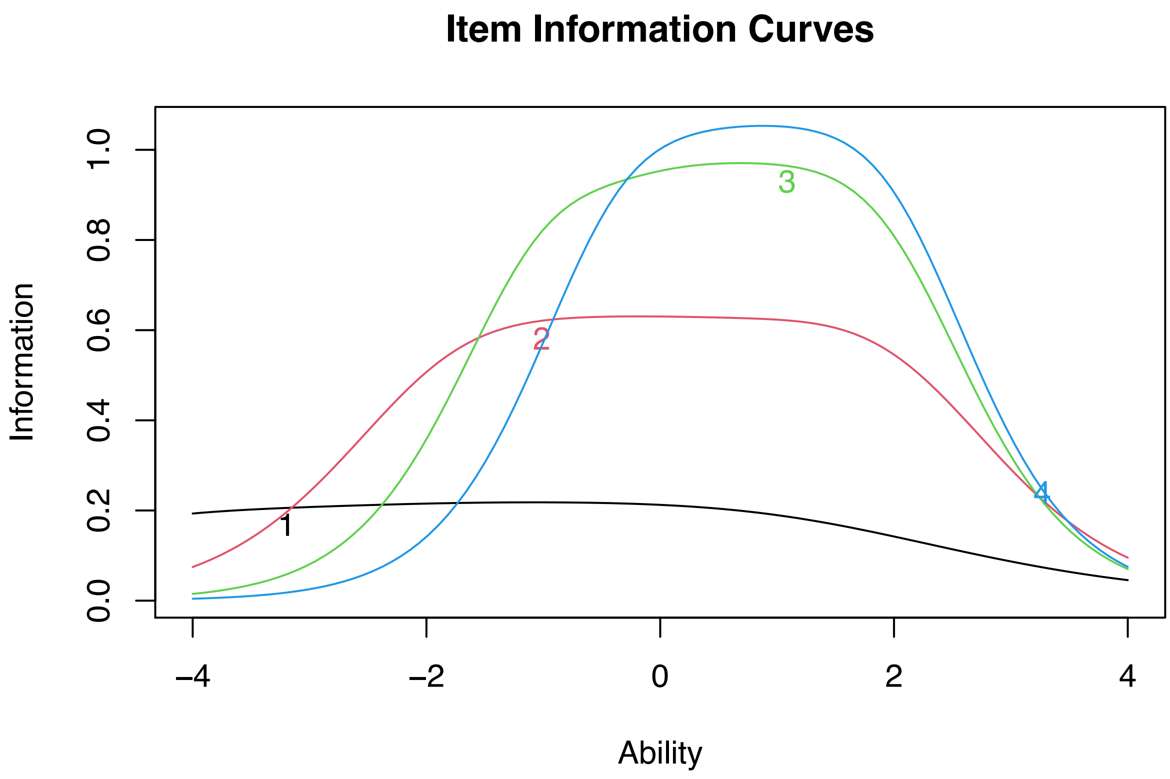

Gender-based DIF: Suicide Risk

```
## Call:
## lordif::lordif(resp.data = as.data.frame(sex.data), group = sex)
##
##   Number of DIF groups: 2
##
##   Number of items flagged for DIF: 0 of 4
##
##   Items flagged:
##
##   Number of iterations for purification: 1 of 10
##
##   Detection criterion: Chisqr
##
##   Threshold: alpha = 0.01
```

Sample-based DIF: Suicide Risk

```
## Call:
## lordif::lordif(resp.data = as.data.frame(sample.data), group = clinYN)
##
##   Number of DIF groups: 2
##
##   Number of items flagged for DIF: 0 of 4
##
##   Items flagged:
##
```

```
##   Number of iterations for purification: 1 of 10
##
##   Detection criterion: Chisqr
##
##   Threshold: alpha = 0.01
```

Back to top

Study 2: ROC curves

For each scale, unadjusted scale scores are compared between the clinical and nonclinical samples in Study 2. For those scales that demonstrated substantial sample-based DIF (Connectedness, Demoralization, Pressure from Negative Affect, Relational Distress, and Somatic Anxiety), these comparisons should not be interpreted.

In all cases, the ROC curve and the area under the curve are presented below. The AUC appears in the manuscript as well.

Attachment

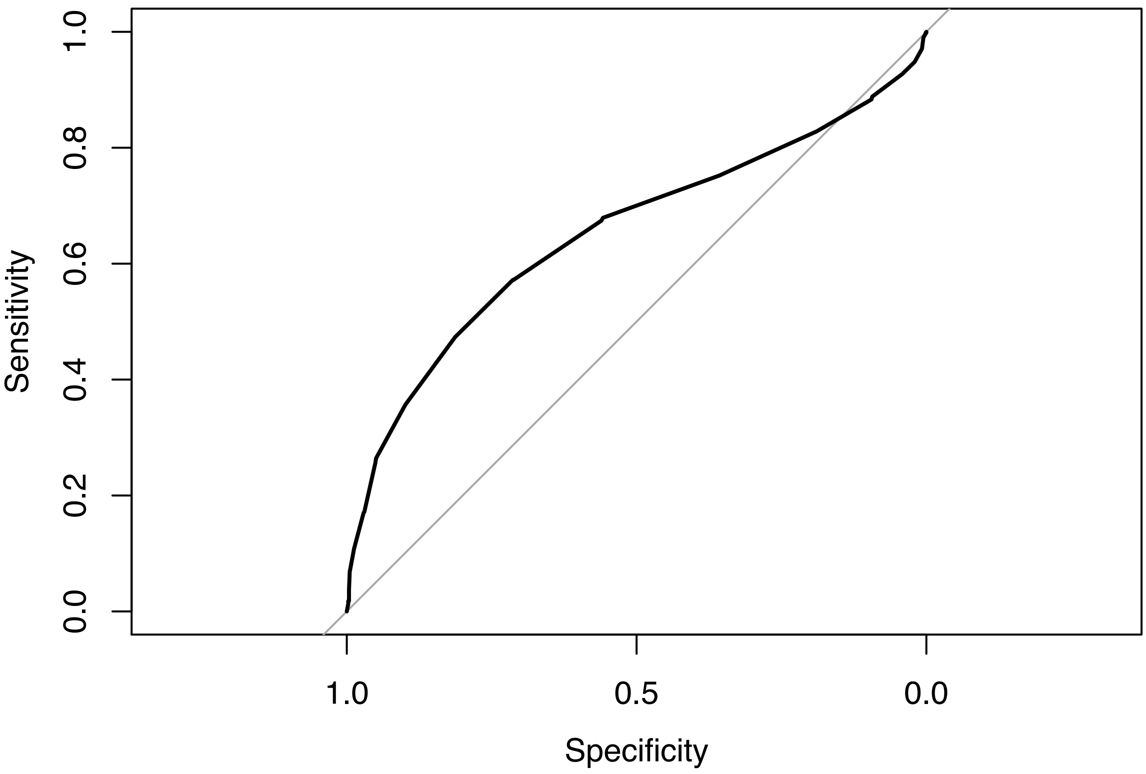


```
## Area under the curve: 0.6532
```

Avoidance

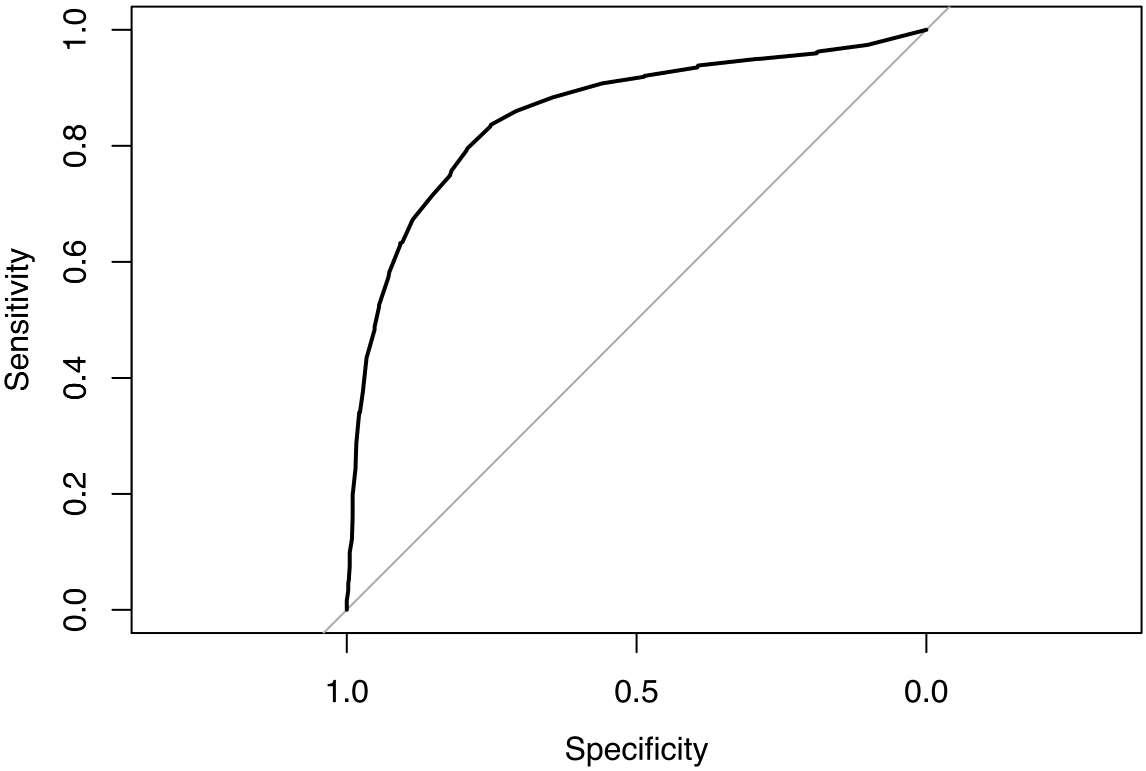


```
## Area under the curve: 0.8564
```

Connectedness

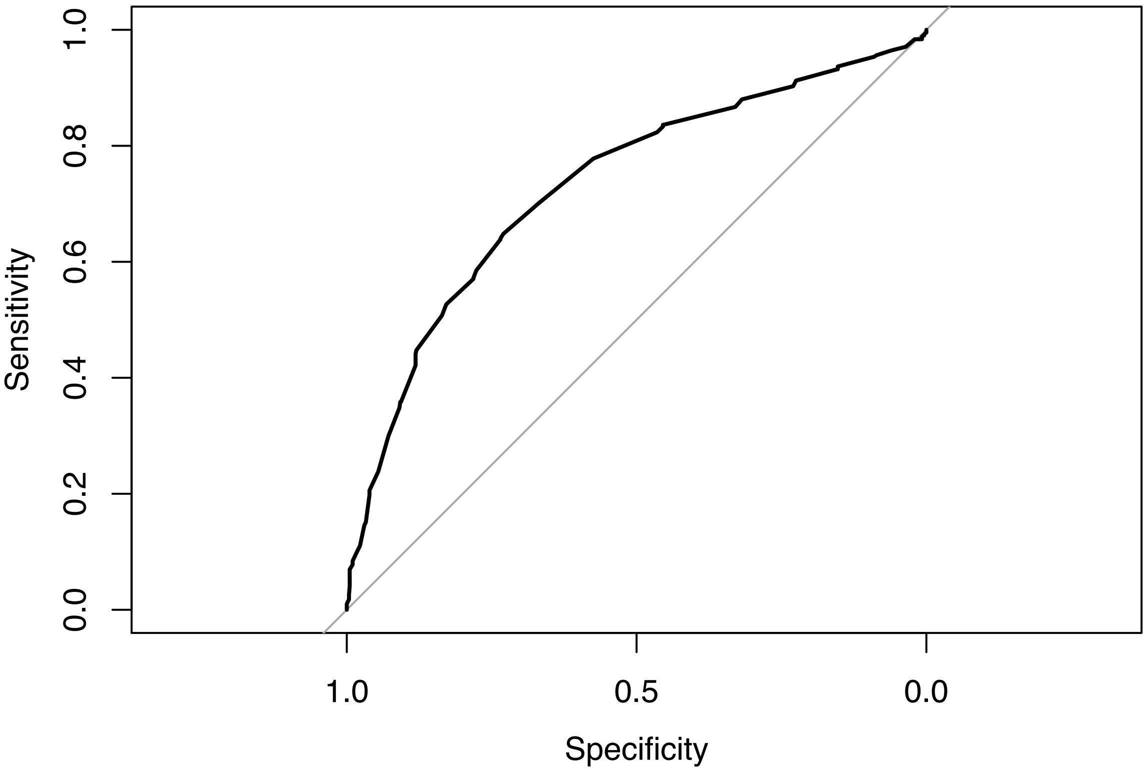


```
## Area under the curve: 0.7321
```

Demoralization

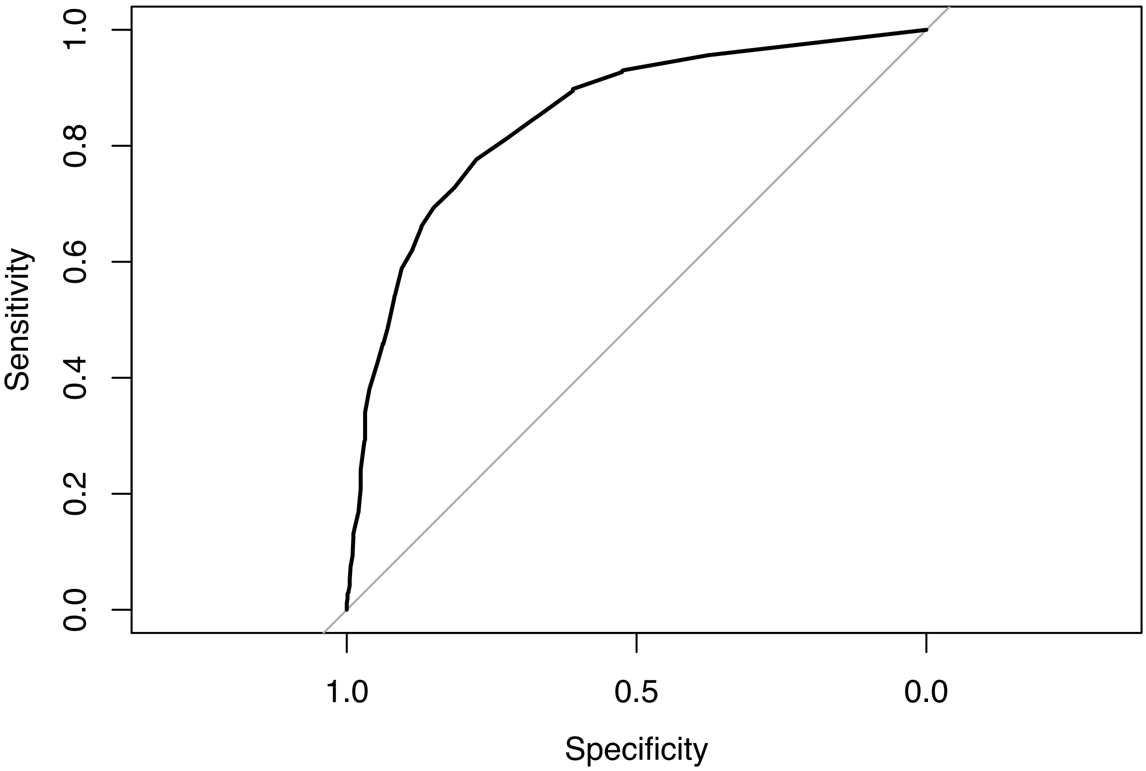


```
## Area under the curve: 0.8483
```

Eating problems

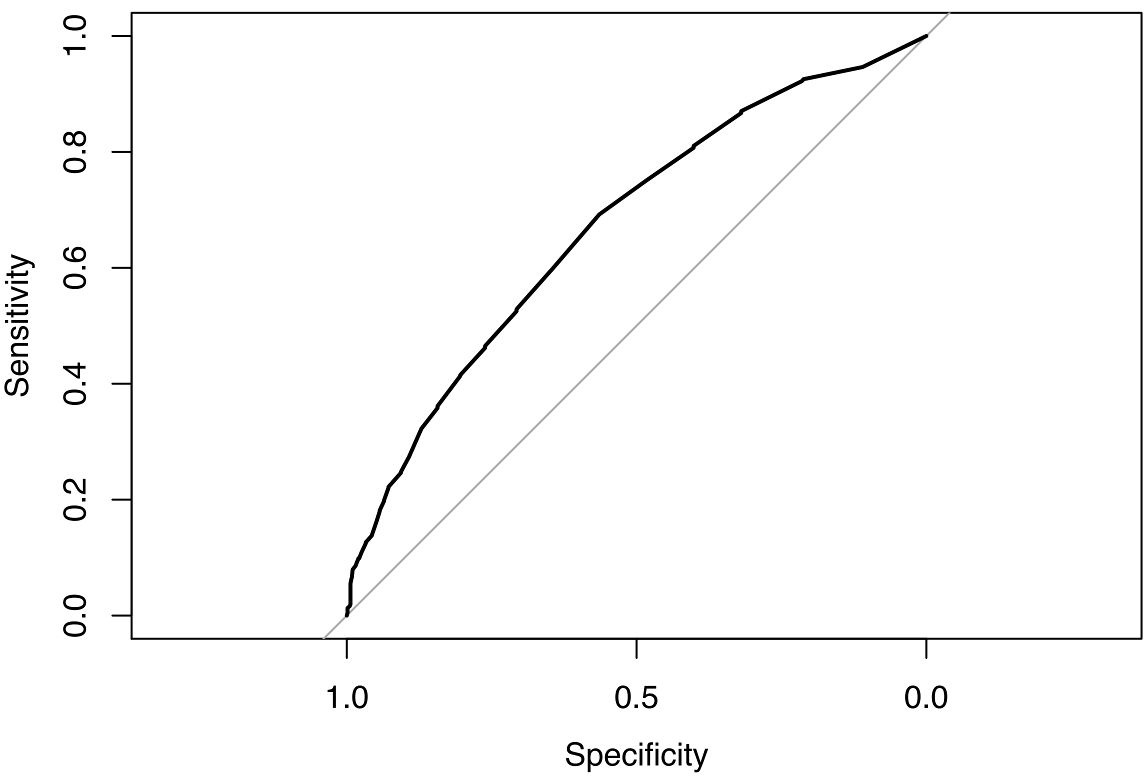


```
## Area under the curve: 0.6715
```

Hurtful rumination

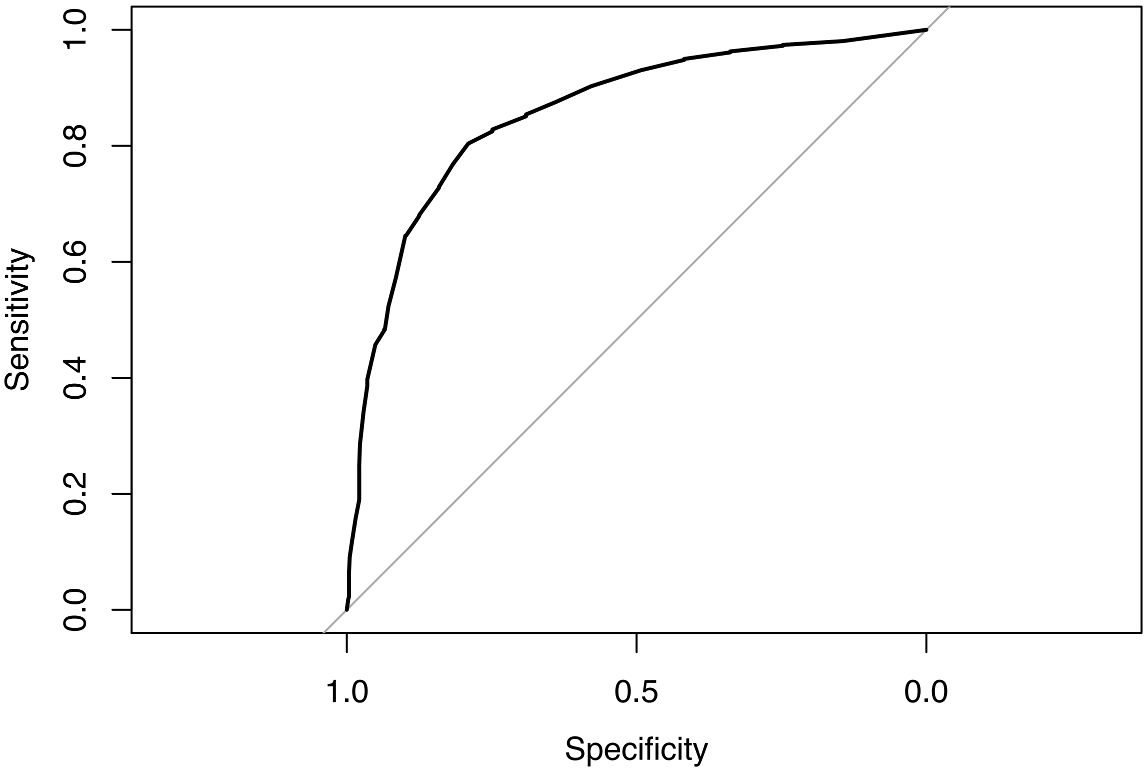


```
## Area under the curve: 0.8572
```

Hypervigilance

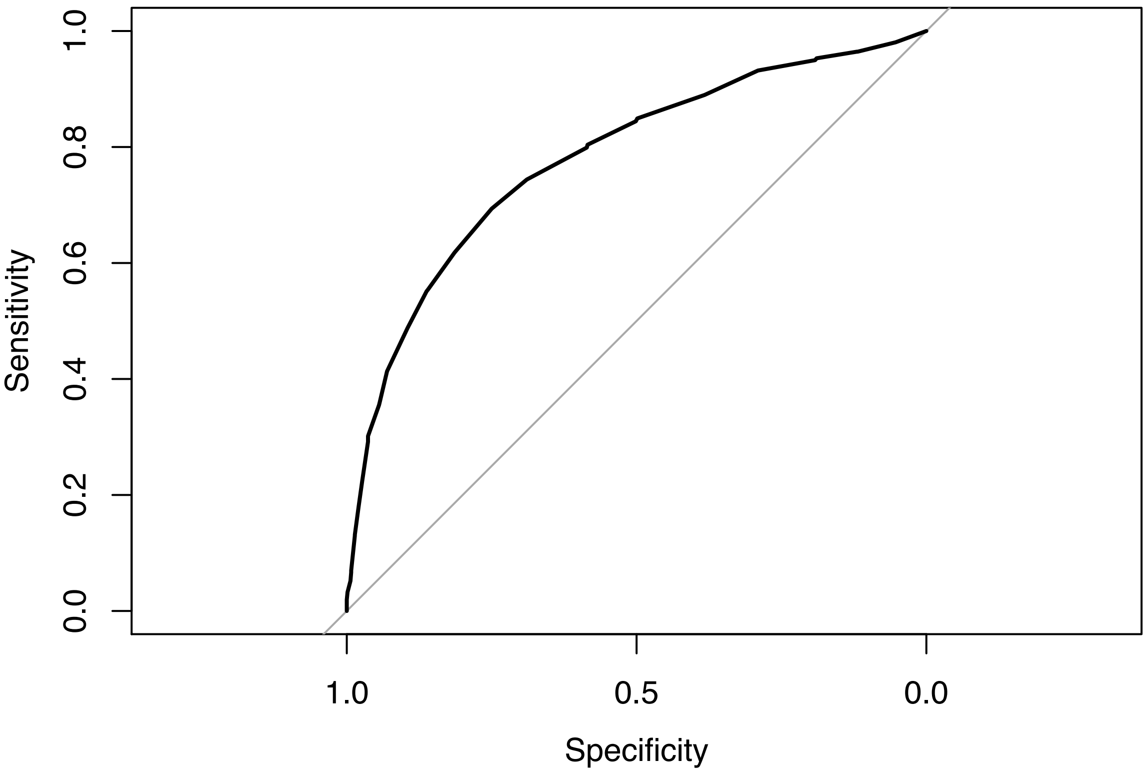


```
## Area under the curve: 0.78
```

Perfectionism

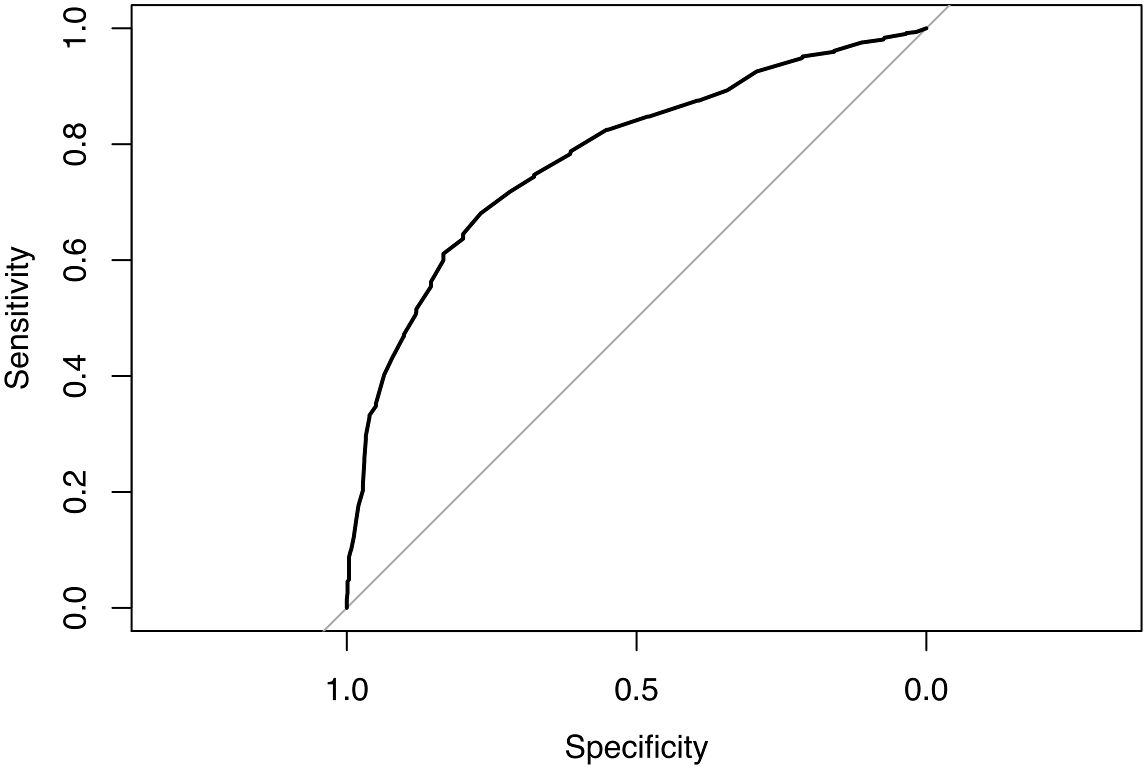


```
## Area under the curve: 0.7799
```

Pressure from Negative Affect

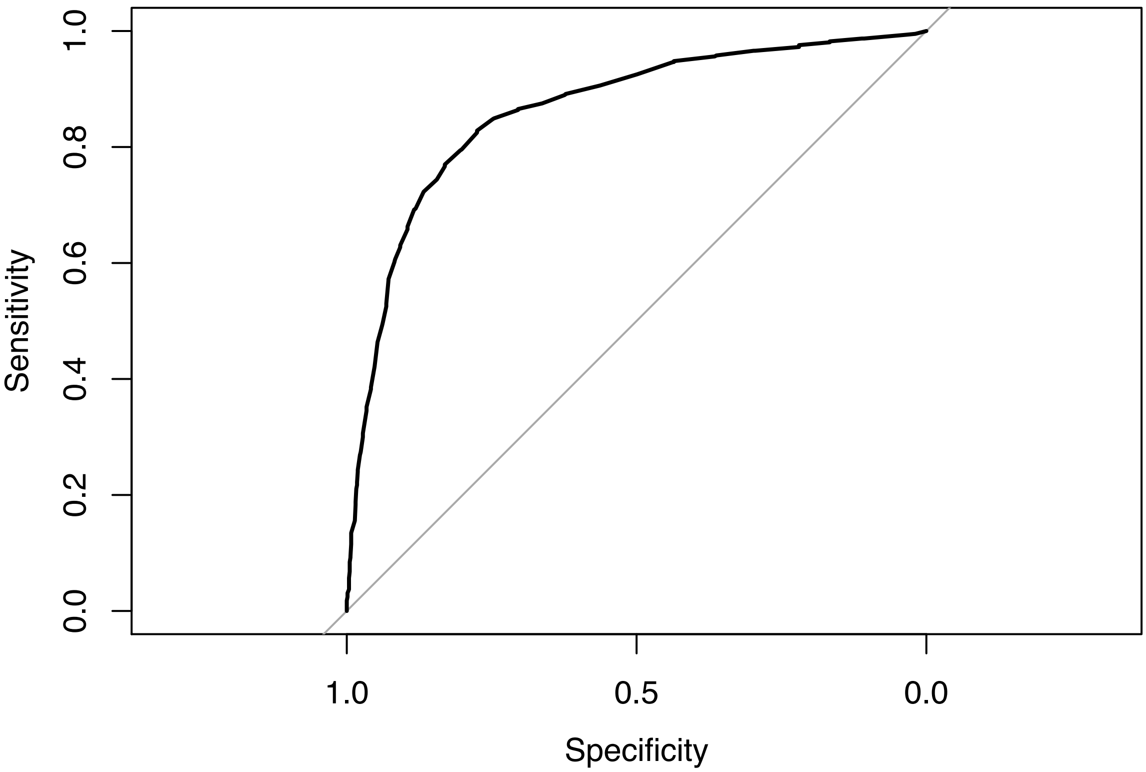


```
## Area under the curve: 0.8626
```

Psychosis

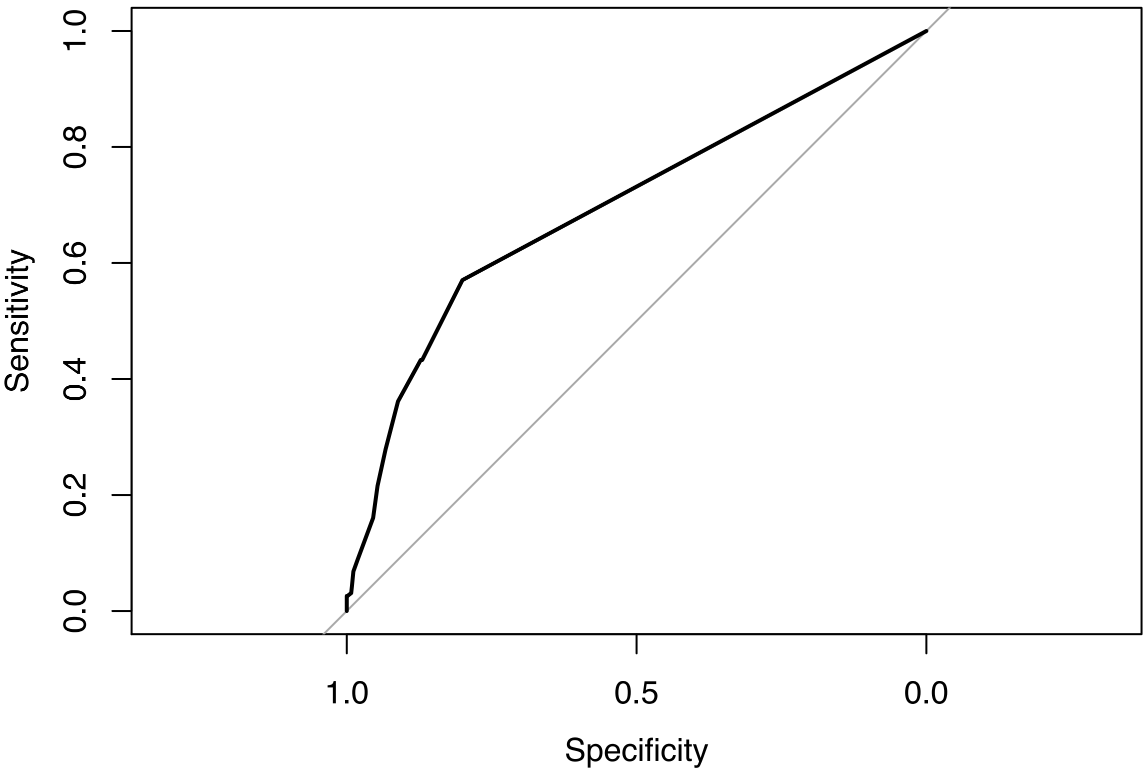


```
## Area under the curve: 0.6961
```

Relational distress

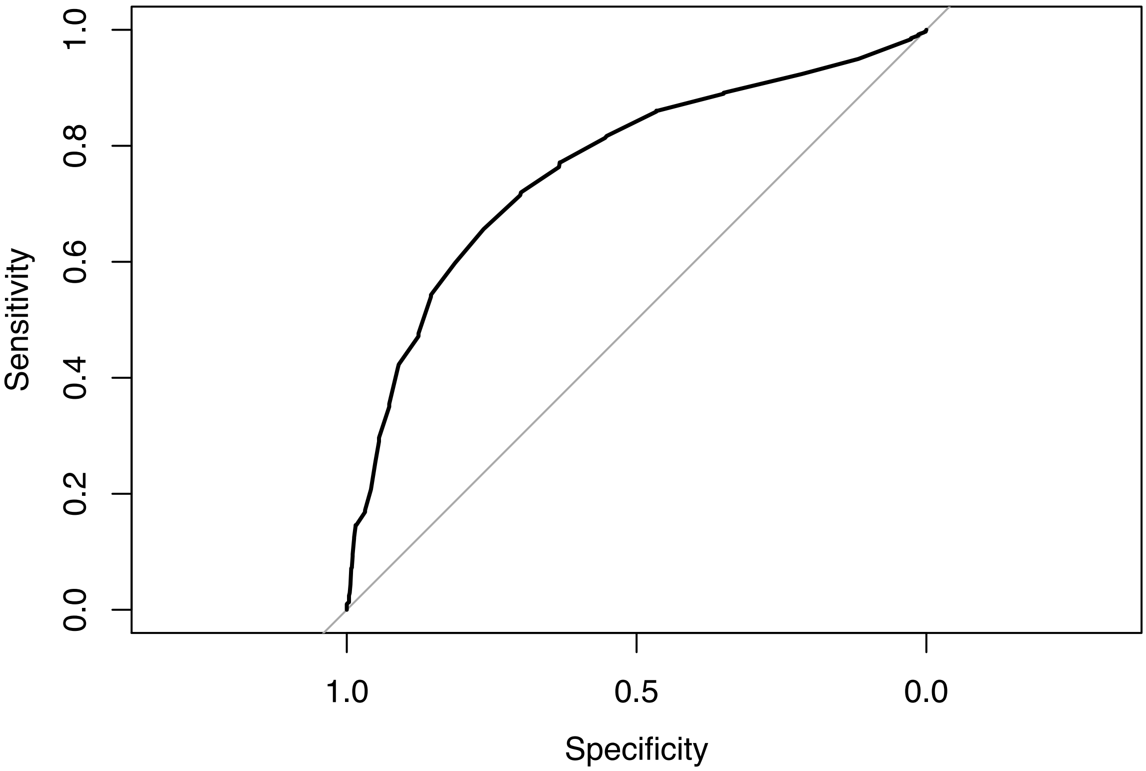


```
## Area under the curve: 0.7612
```

Resilience

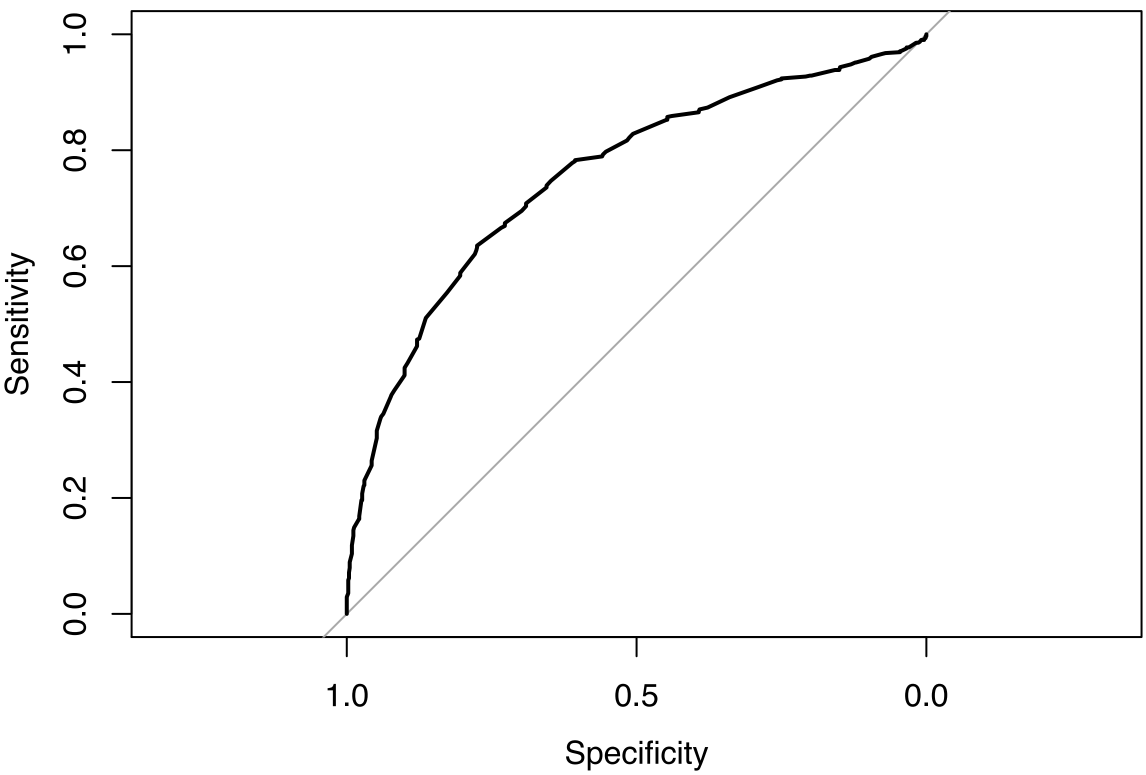


```
## Area under the curve: 0.7562
```

Social Role Functioning

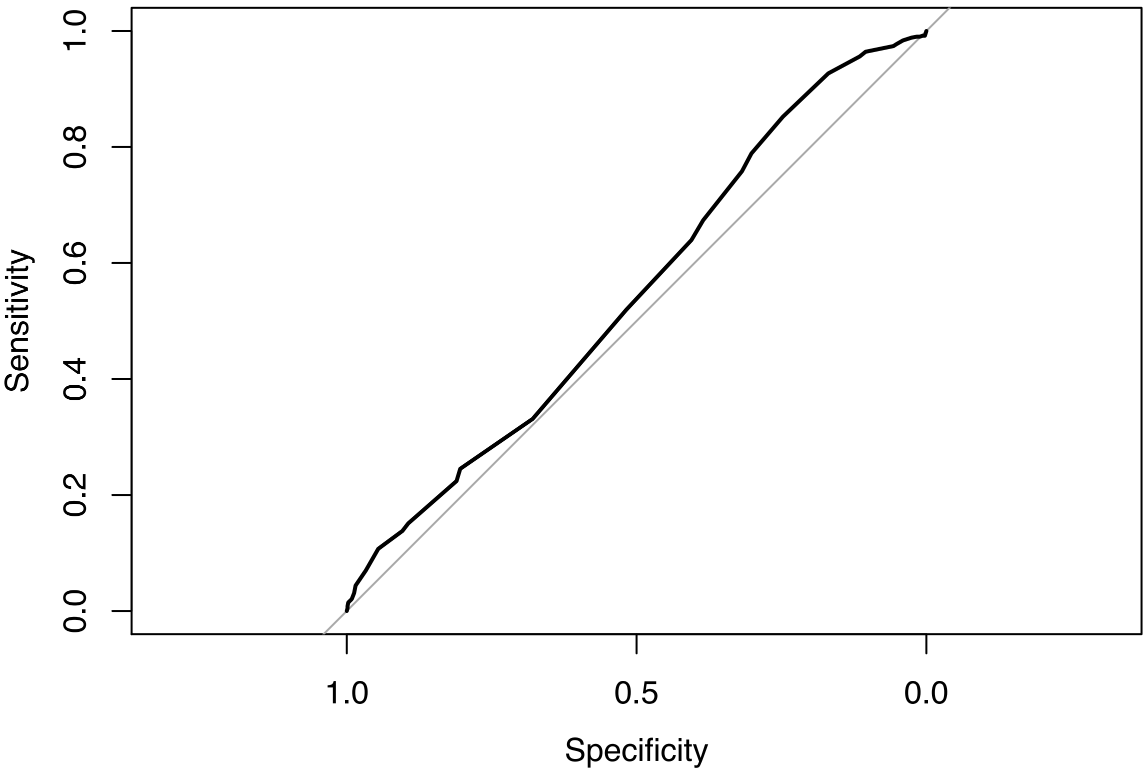


```
## Area under the curve: 0.5482
```

Somatic Anxiety

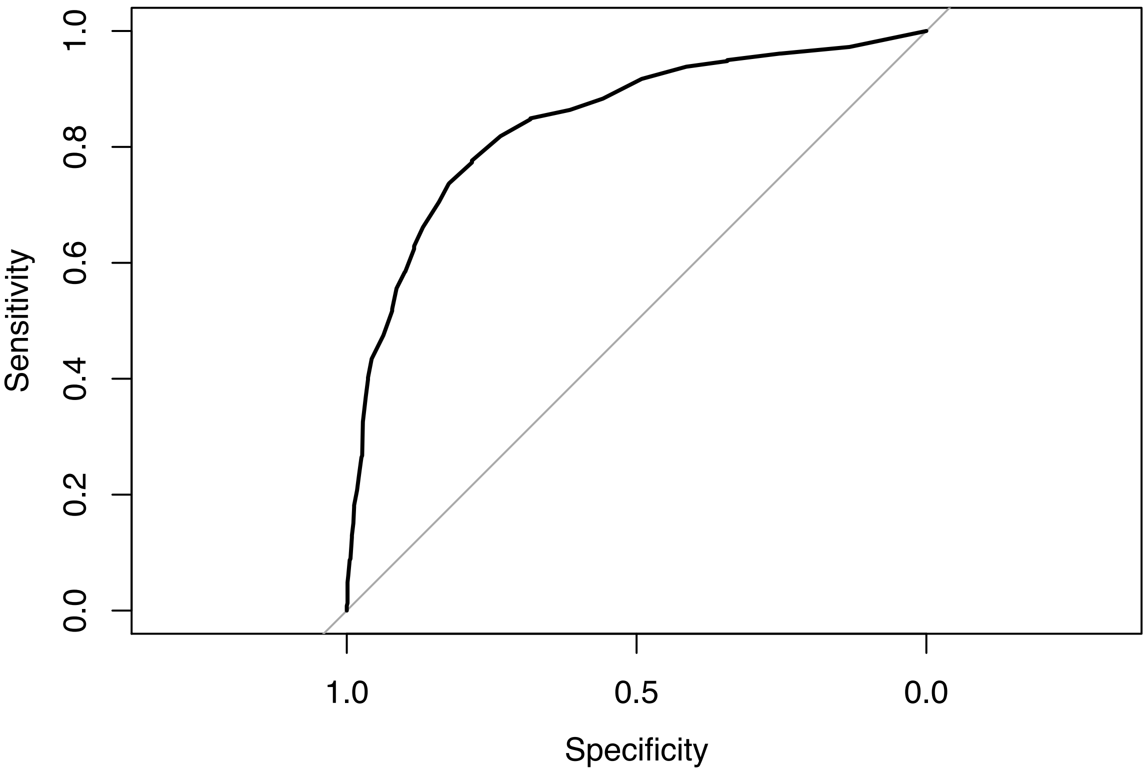


```
## Area under the curve: 0.8426
```

Substance Use

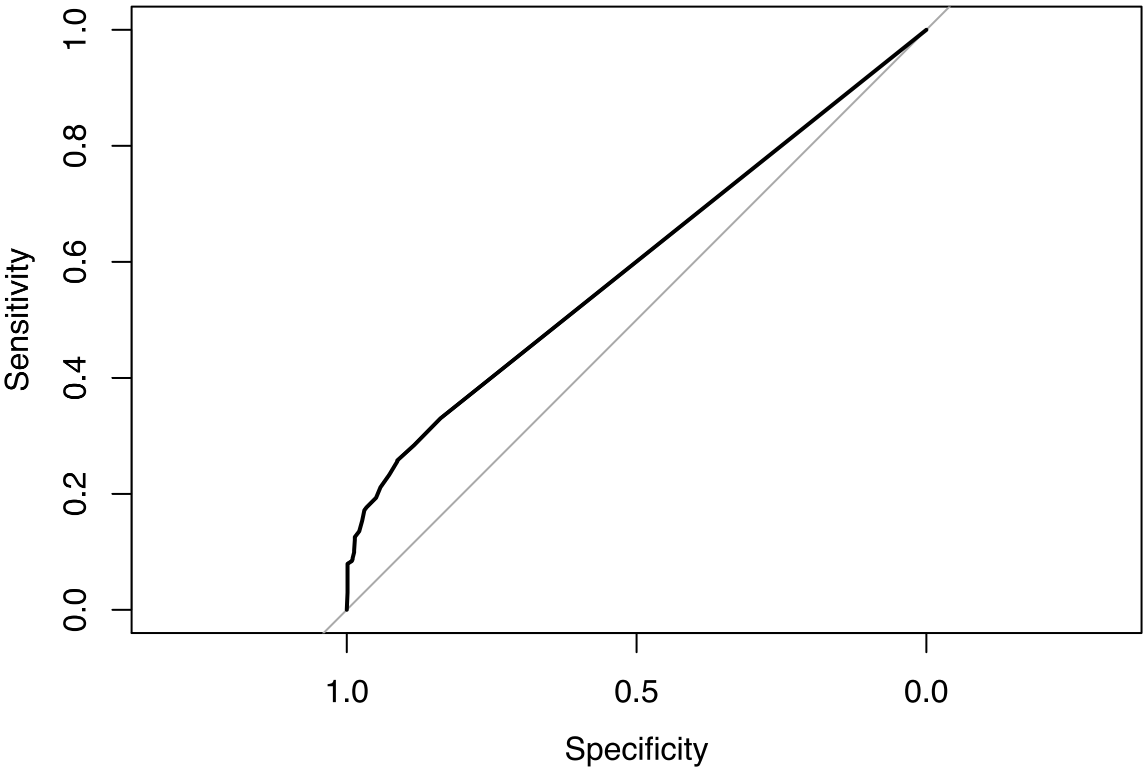


```
## Area under the curve: 0.5948
```

Suicide Risk

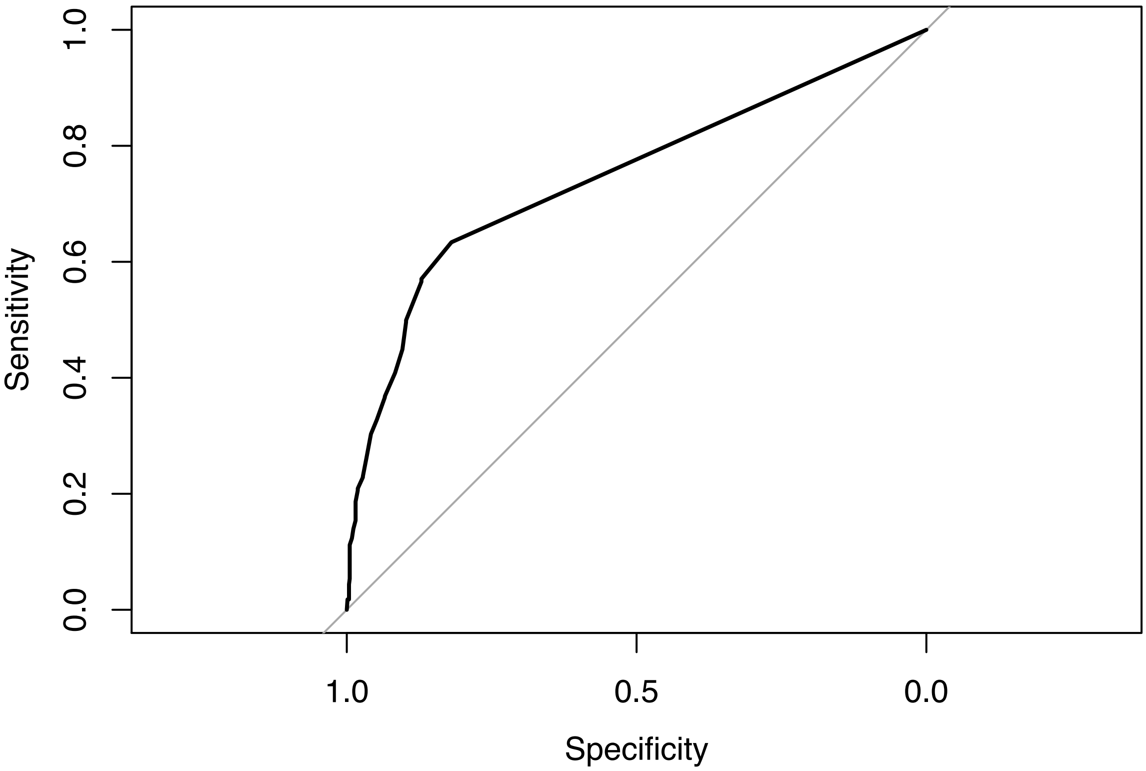


```
## Area under the curve: 0.7457
```

Study 3: Scale performance

Eating Problems

Site 1

Reliability: Eating Problems

```
## Cronbach's alpha is 0.86.
## Mean item-total correlation is 0.561.
## If each item were dropped:
##      raw_alpha std.alpha G6(smc) average_r S/N alpha se  var.r med.r
## Q46       0.82      0.83    0.79      0.55 4.9    0.012 0.0044  0.56
## Q104      0.84      0.85    0.81      0.58 5.5    0.010 0.0013  0.57
## Q57       0.84      0.85    0.81      0.58 5.5    0.010 0.0022  0.57
## Q18       0.83      0.83    0.79      0.55 4.9    0.011 0.0027  0.56
## Q63       0.82      0.83    0.79      0.55 4.8    0.011 0.0031  0.57
```

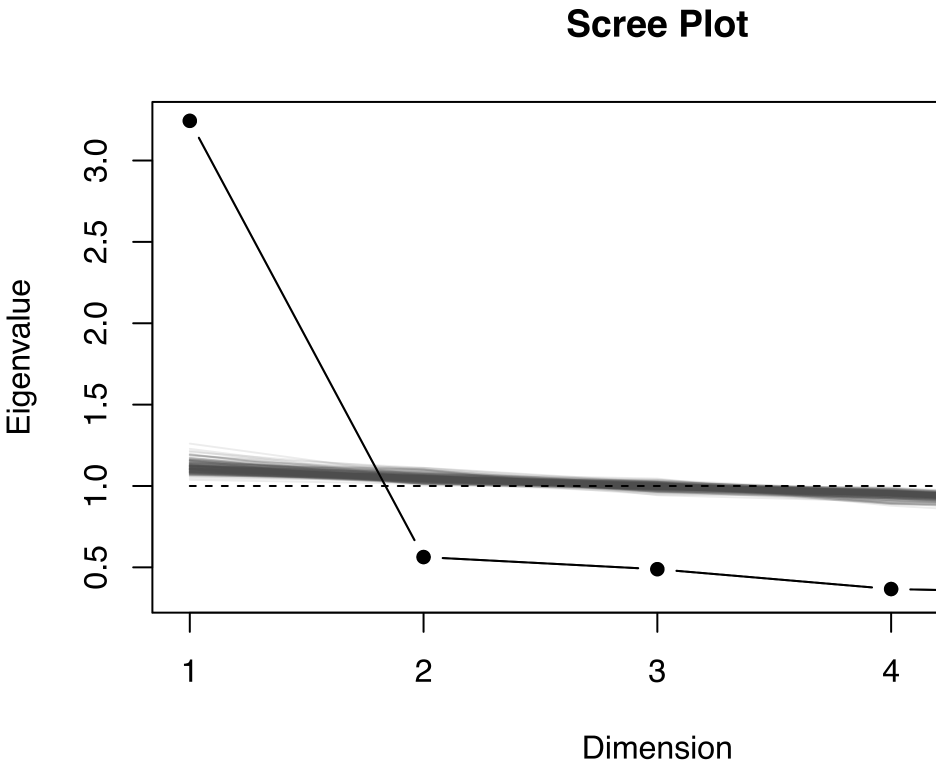

Unidimensionality: Eating Problems

```
## [1] "Ratio of first to second eigenvalues: 5.76"

## [1] 3.2438805 0.5631446 0.4885950 0.3666453 0.3377346

## Factor Analysis using method =  minres
## Call: fa(r = grm_obj$X)
## Standardized loadings (pattern matrix) based upon correlation matrix
##       MR1   h2   u2 com
## Q46  0.78 0.60 0.40   1
## Q104 0.70 0.49 0.51   1
## Q57  0.71 0.50 0.50   1
## Q18  0.77 0.59 0.41   1
## Q63  0.79 0.62 0.38   1
##
##                 MR1
## SS loadings    2.81
## Proportion Var 0.56
##
## Mean item complexity =  1
## Test of the hypothesis that 1 factor is sufficient.
##
## The degrees of freedom for the null model are  10  and the objective function was  2.2 with Chi Squa
## The degrees of freedom for the model are 5  and the objective function was  0.07
##
## The root mean square of the residuals (RMSR) is  0.03
## The df corrected root mean square of the residuals is  0.05
##
## The harmonic number of observations is  592 with the empirical chi square  14.43  with prob <  0.013
## The total number of observations was  617  with Likelihood Chi Square =  40.05  with prob <  1.5e-07
##
## Tucker Lewis Index of factoring reliability =  0.948
```

```
## RMSEA index =  0.107  and the 90 % confidence intervals are  0.077 0.138
## BIC =  7.92
## Fit based upon off diagonal values = 1
## Measures of factor score adequacy
##                                                    MR1
## Correlation of (regression) scores with factors   0.93
## Multiple R square of scores with factors          0.87
## Minimum correlation of possible factor scores     0.74
```

Graded-Response Model: Eating Problems

```
##      Extrmt1 Extrmt2 Extrmt3 Extrmt4 Extrmt5 Extrmt6 Dscrmn
## Q46   -0.219   0.219   0.513   0.797   1.144   1.446  2.405
## Q104  -0.722  -0.216   0.084   0.367   0.753   1.108  2.363
## Q57    0.595   0.964   1.246   1.464   1.697   1.960  2.657
## Q18    0.415   0.889   1.108   1.334   1.613   1.917  3.116
## Q63   -0.216   0.242   0.565   0.832   1.254   1.581  2.544
```

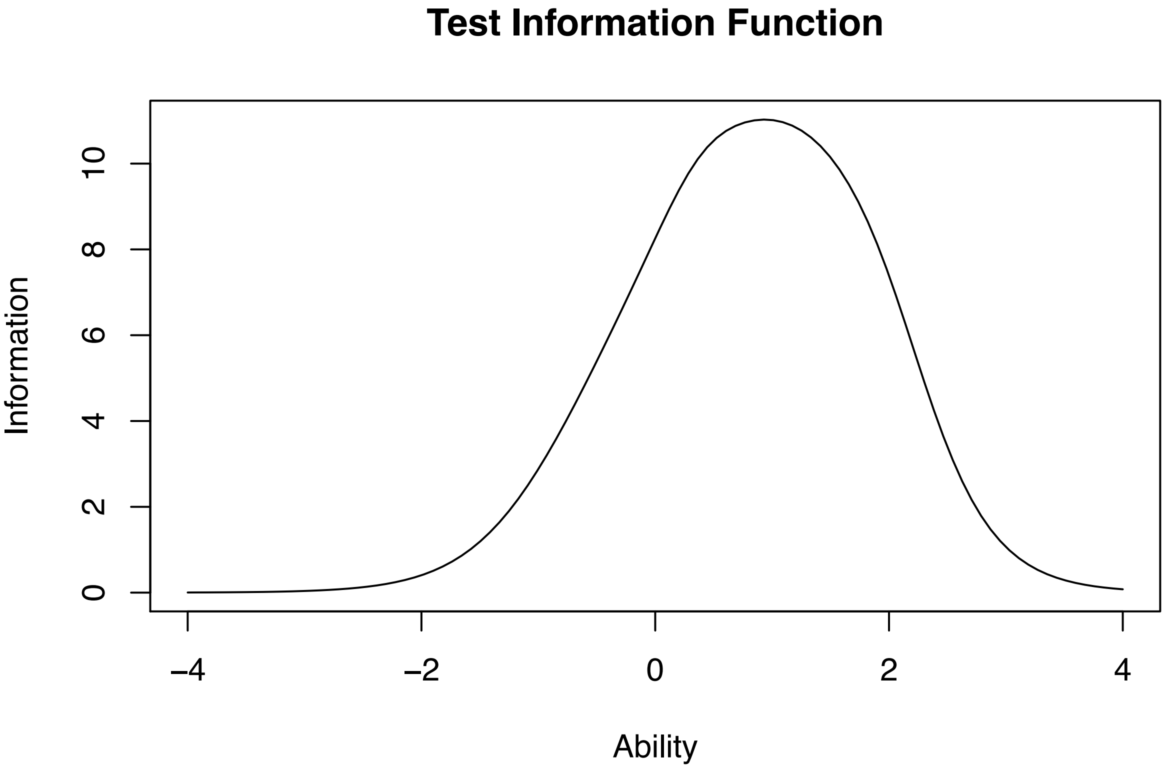

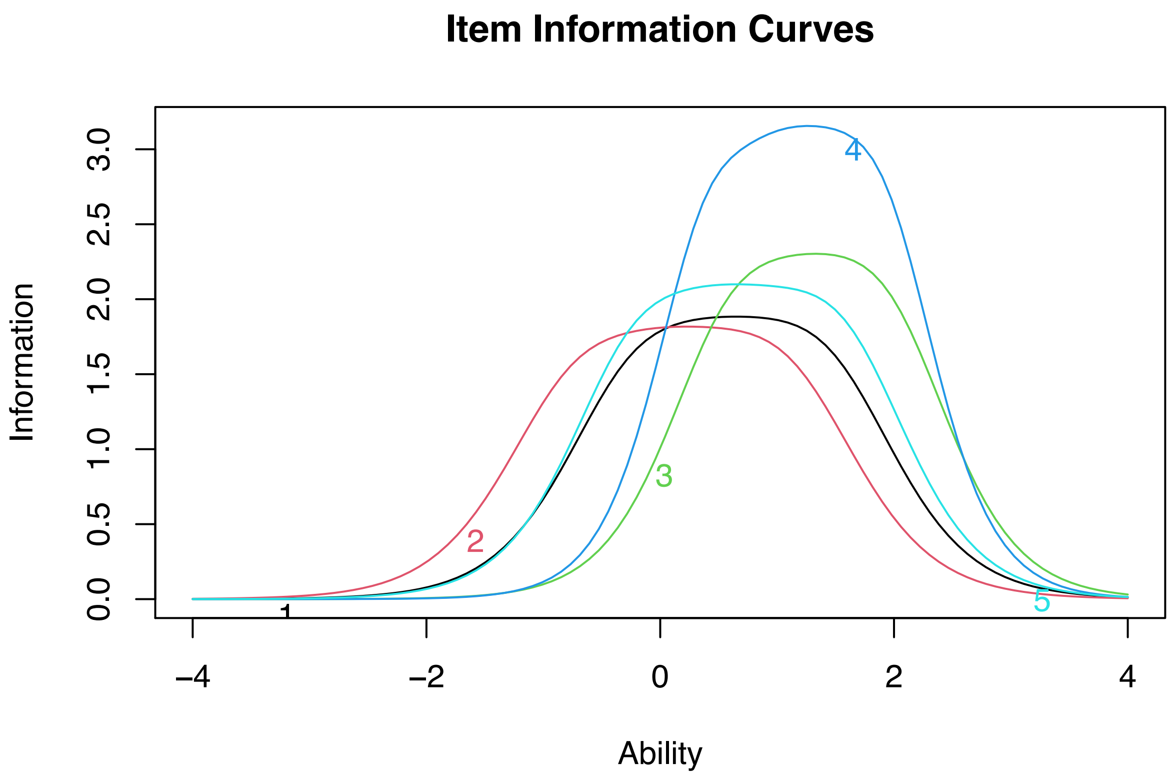

Back to top

Site 2

Reliability: Eating Problems

```
## Cronbach's alpha is 0.836.
## Mean item-total correlation is 0.525.
## If each item were dropped:
##      raw_alpha std.alpha G6(smc) average_r S/N alpha se  var.r med.r
## Q46       0.78      0.80    0.76      0.50 4.0    0.014 0.0034  0.50
## Q104      0.81      0.82    0.78      0.53 4.6    0.012 0.0036  0.54
## Q57       0.82      0.83    0.79      0.55 5.0    0.012 0.0022  0.55
## Q18       0.80      0.80    0.76      0.50 4.1    0.013 0.0041  0.51
## Q63       0.81      0.82    0.78      0.53 4.6    0.012 0.0042  0.53
```

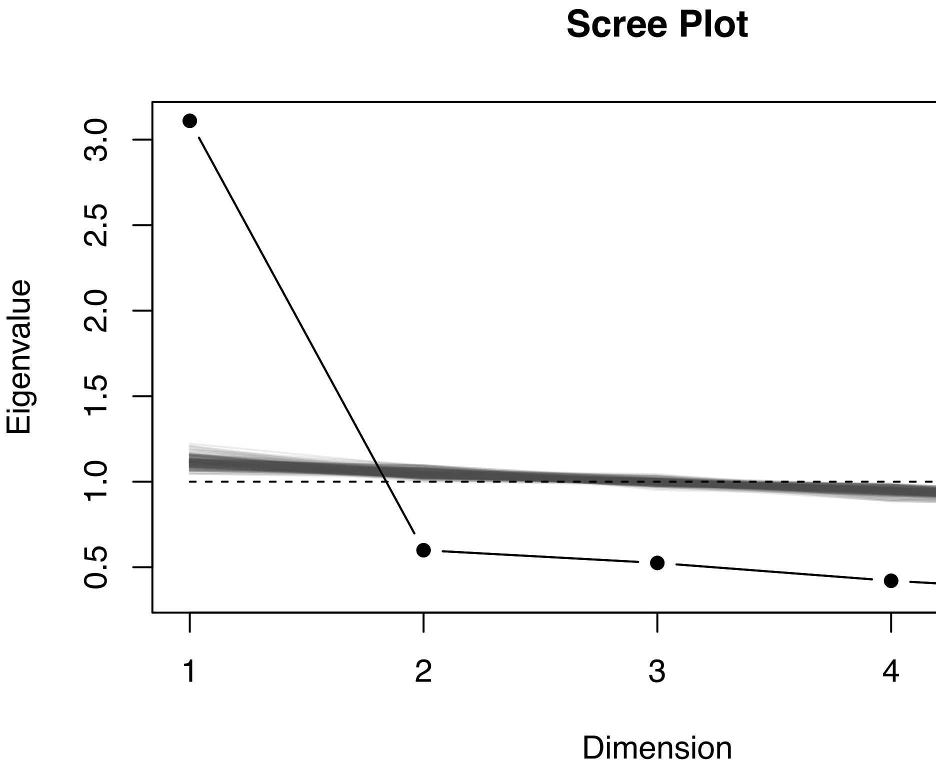

Unidimensionality: Eating Problems

```
## [1] "Ratio of first to second eigenvalues: 5.188"

## [1] 3.1099043 0.5994110 0.5250118 0.4206820 0.3449910

## Factor Analysis using method =  minres
## Call: fa(r = grm_obj$X)
## Standardized loadings (pattern matrix) based upon correlation matrix
##       MR1   h2   u2 com
## Q46  0.79 0.63 0.37   1
## Q104 0.70 0.49 0.51   1
## Q57  0.65 0.42 0.58   1
## Q18  0.78 0.61 0.39   1
## Q63  0.70 0.50 0.50   1
##
##                 MR1
## SS loadings    2.65
## Proportion Var 0.53
##
## Mean item complexity =  1
## Test of the hypothesis that 1 factor is sufficient.
##
## The degrees of freedom for the null model are  10  and the objective function was  1.95 with Chi Squa
## The degrees of freedom for the model are 5  and the objective function was  0.05
##
## The root mean square of the residuals (RMSR) is  0.03
## The df corrected root mean square of the residuals is  0.04
##
## The harmonic number of observations is  572 with the empirical chi square  11.25  with prob <  0.047
## The total number of observations was  596  with Likelihood Chi Square =  28.49  with prob <  2.9e-05
##
## Tucker Lewis Index of factoring reliability =  0.959
```

```
## RMSEA index =  0.089  and the 90 % confidence intervals are  0.059 0.122
## BIC =  -3.46
## Fit based upon off diagonal values = 1
## Measures of factor score adequacy
##                                                    MR1
## Correlation of (regression) scores with factors   0.93
## Multiple R square of scores with factors          0.86
## Minimum correlation of possible factor scores     0.71
```

Graded-Response Model: Eating Problems

```
##      Extrmt1 Extrmt2 Extrmt3 Extrmt4 Extrmt5 Extrmt6 Dscrmn
## Q46   -1.087  -1.272  -1.337  -1.577  -1.692  -1.817 -3.757
## Q104   0.022  -0.590  -0.940  -1.299  -1.680  -1.780 -1.931
## Q57   -1.098  -1.372  -1.452  -1.707  -1.906  -2.019 -3.293
## Q18   -1.207  -1.595  -1.757  -2.001  -2.165  -2.255 -3.501
## Q63   -0.745  -1.171  -1.416  -1.915  -2.193  -2.336 -2.628
```

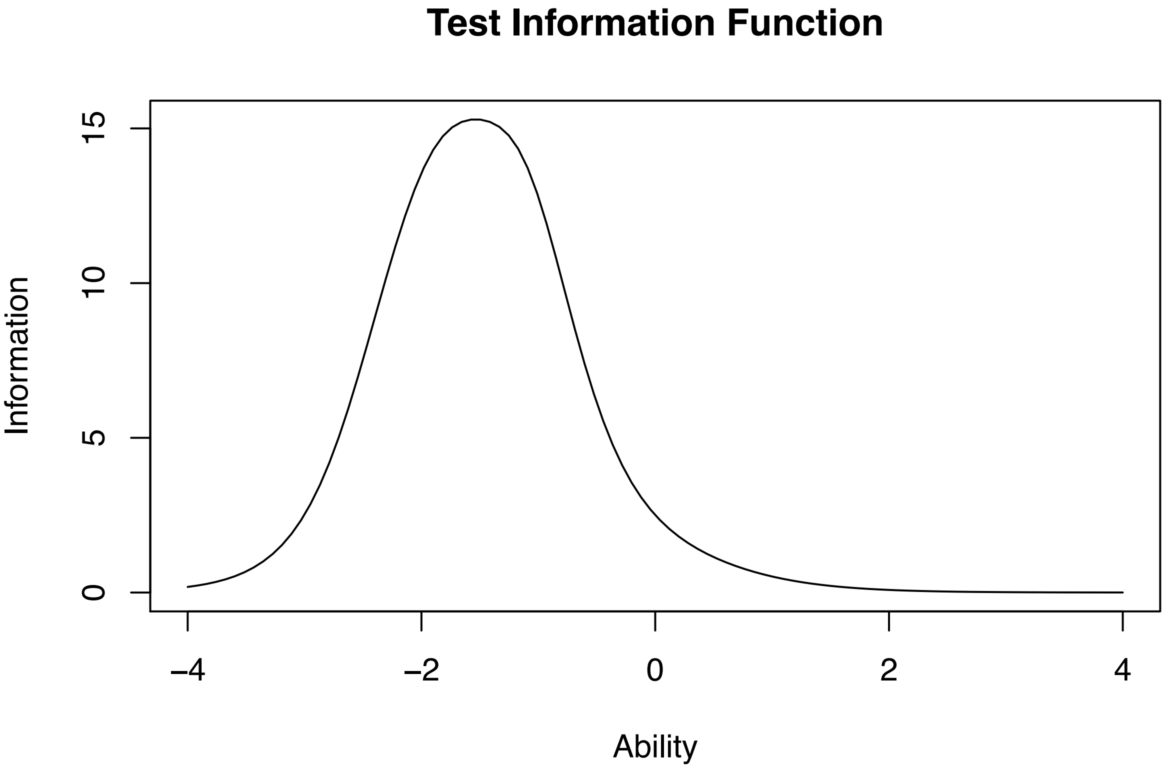

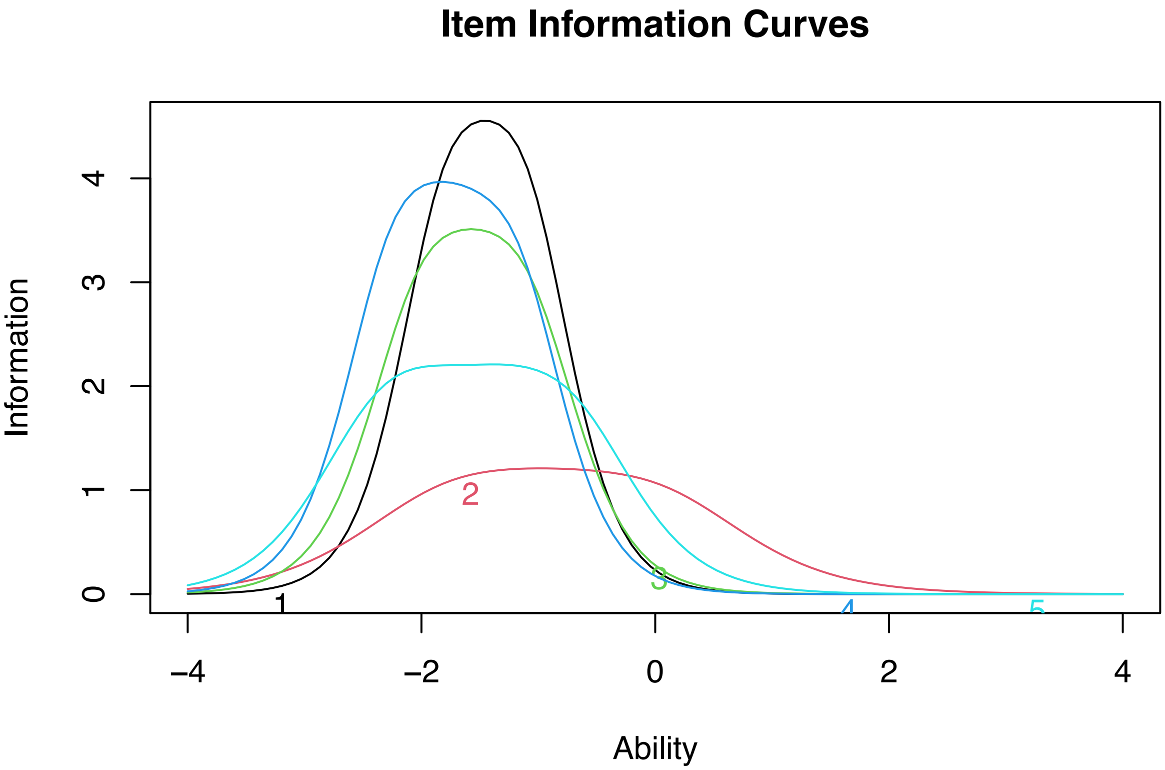

Back to top

Site DIF

```
## Call:
## lordif::lordif(resp.data = as.data.frame(merged_data), group = site)
##
##   Number of DIF groups: 2
##
##   Number of items flagged for DIF: 1 of 5
##
##   Items flagged: 1
##
##   Number of iterations for purification: 3 of 10
##
##   Detection criterion: Chisqr
##
##   Threshold: alpha = 0.01
##
##   item ncat  chi12  chi13  chi23
## 1    1    7 0.0000 0.0000 0.0003
## 2    2    7 0.2634 0.1704 0.1303
## 3    3    7 0.2341 0.2054 0.1859
## 4    4    7 0.0414 0.1130 0.6533
## 5    5    7 0.7899 0.9417 0.8246
```

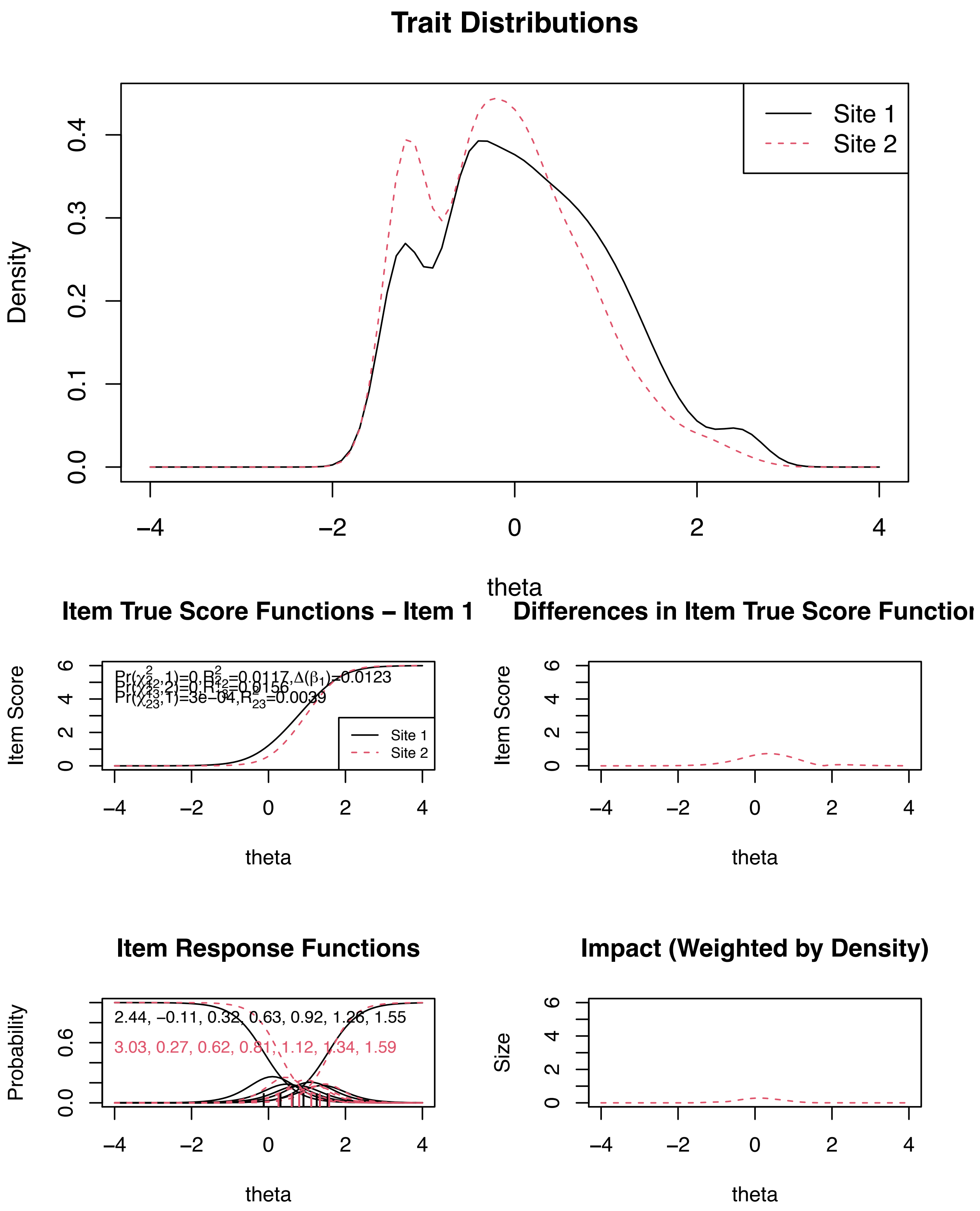
Trait Distributions
Density
theta
Site 1
Site 2
Item True Score Functions – Item 1
Differences in Item True Score Functioı
Item Score
Site 1
Site 2
Item Response Functions
Impact (Weighted by Density)
Probability
Size
2.44, −0.11, 0.32, 0.63, 0.92, 1.26, 1.55
3.03, 0.27, 0.62, 0.81, 1.12, 1.34, 1.59

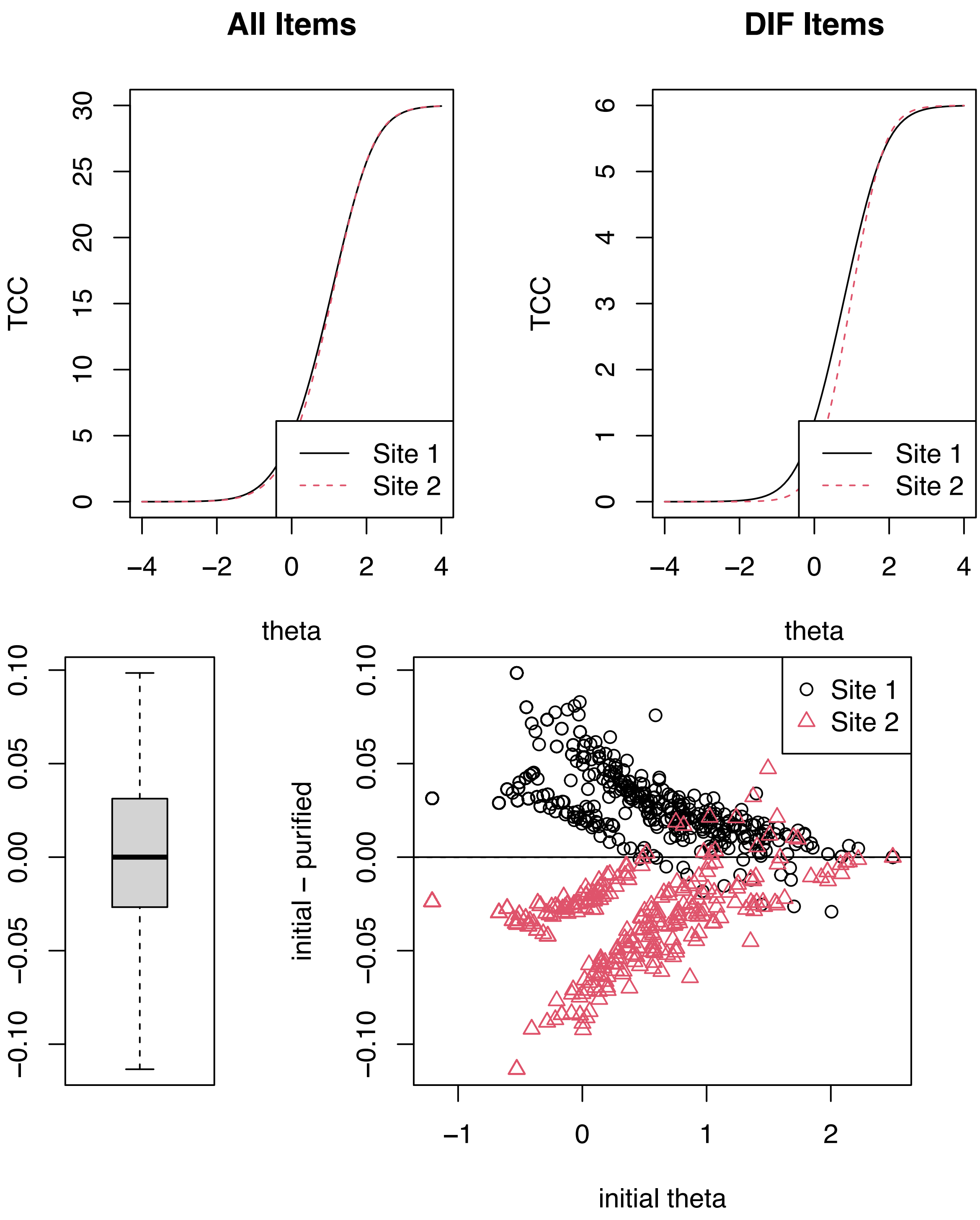

Back to top

Gender-based DIF: Eating Problems

```
## Call:
## lordif::lordif(resp.data = as.data.frame(sex.data), group = sex)
##
```

```
##   Number of DIF groups: 2
##
##   Number of items flagged for DIF: 3 of 5
##
##   Items flagged: 1, 2, 4
##
##   Number of iterations for purification: 5 of 10
##
##   Detection criterion: Chisqr
##
##   Threshold: alpha = 0.01
##
##   item ncat  chi12  chi13  chi23
## 1    1    7 0.0000 0.0000 0.1223
## 2    2    7 0.0000 0.0000 0.3713
## 3    3    6 0.3378 0.3189 0.2423
## 4    4    4 0.0000 0.0000 0.2853
## 5    5    7 0.2097 0.4488 0.8653
```

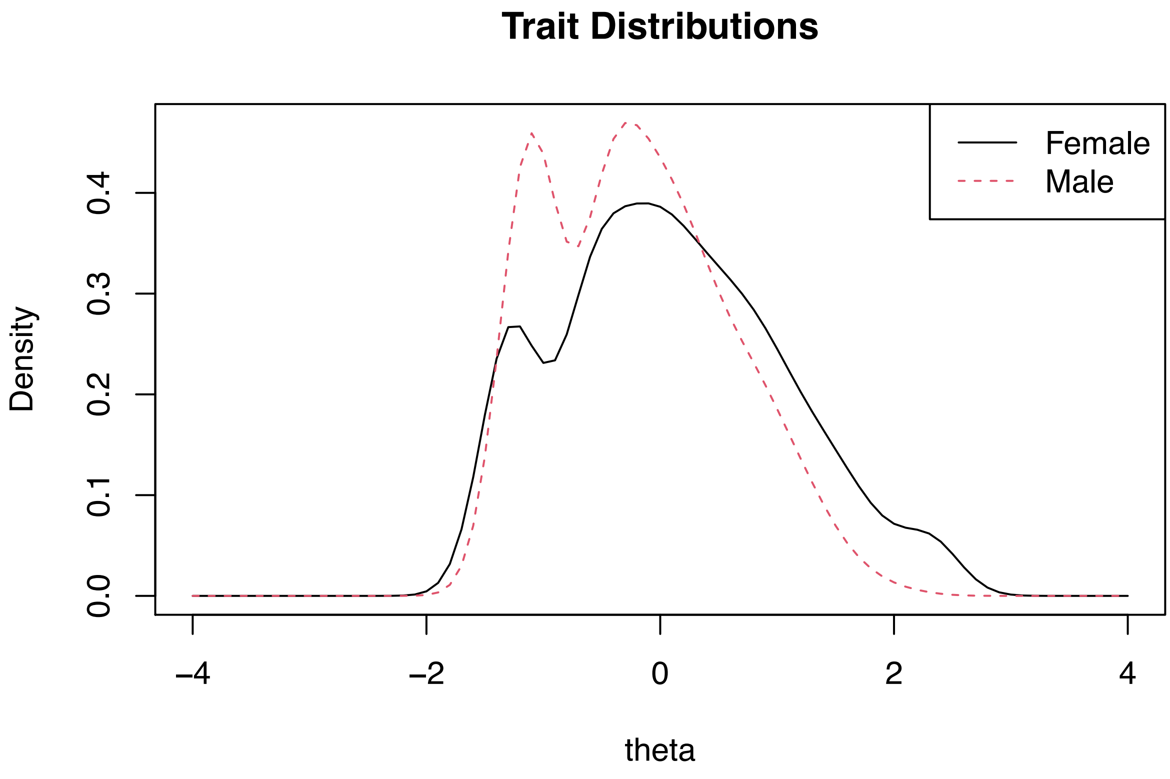

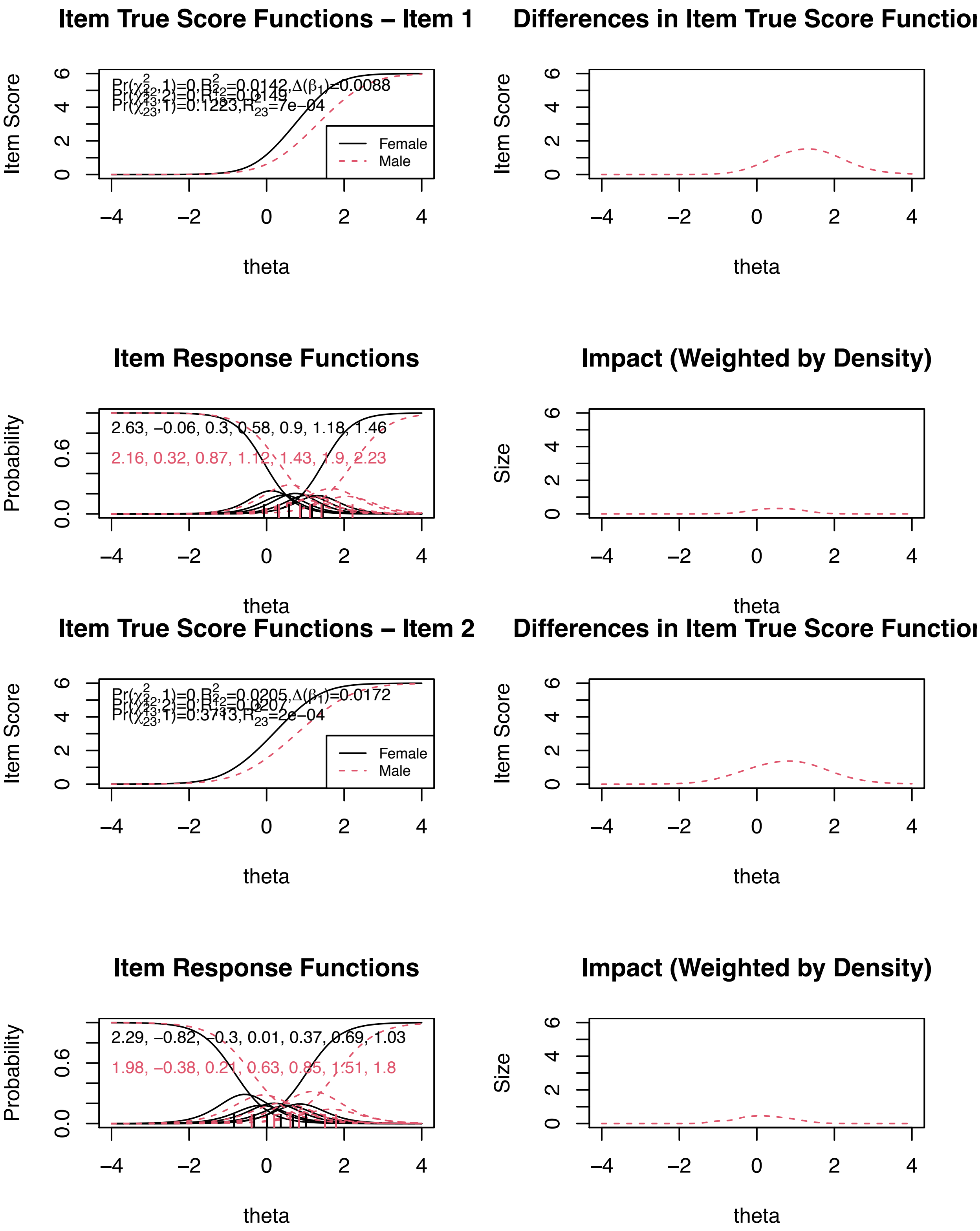
Item True Score Functions – Item 1
Differences in Item True Score Functio
Item Score
theta
Female
Male
Item Response Functions
Impact (Weighted by Density)
Probability
Size
2.63, –0.06, 0.3, 0.58, 0.9, 1.18, 1.46
2.16, 0.32, 0.87, 1.12, 1.43, 1.9, 2.23
Item True Score Functions – Item 2
2.29, –0.82, –0.3, 0.01, 0.37, 0.69, 1.03
1.98, –0.38, 0.21, 0.63, 0.85, 1.51, 1.8

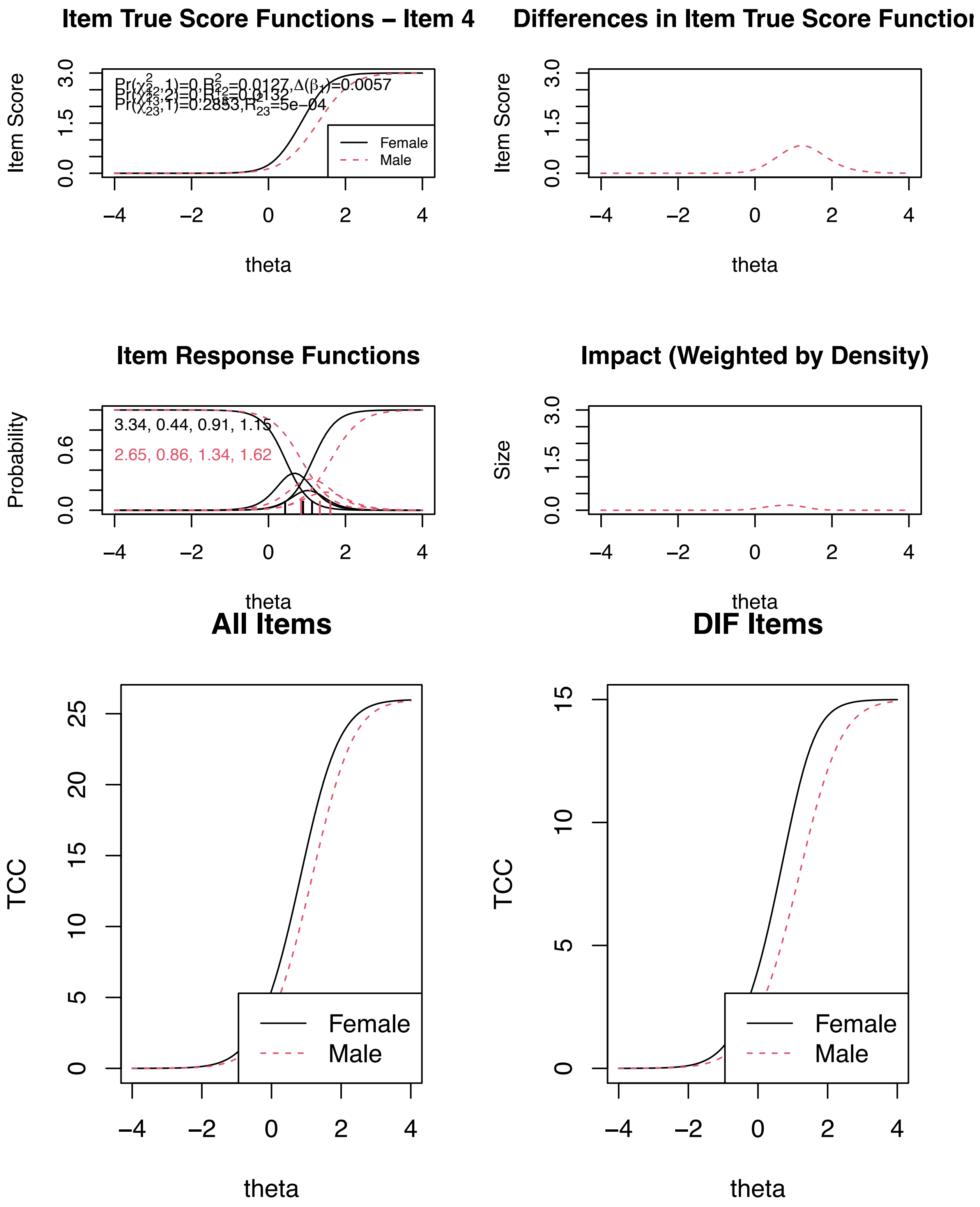
Item True Score Functions – Item 4
Differences in Item True Score Functio
Item Response Functions
Impact (Weighted by Density)
All Items
DIF Items
Item Score
Probability
Size
TCC
theta
Female
Male
3.34, 0.44, 0.91, 1.15
2.65, 0.86, 1.34, 1.62

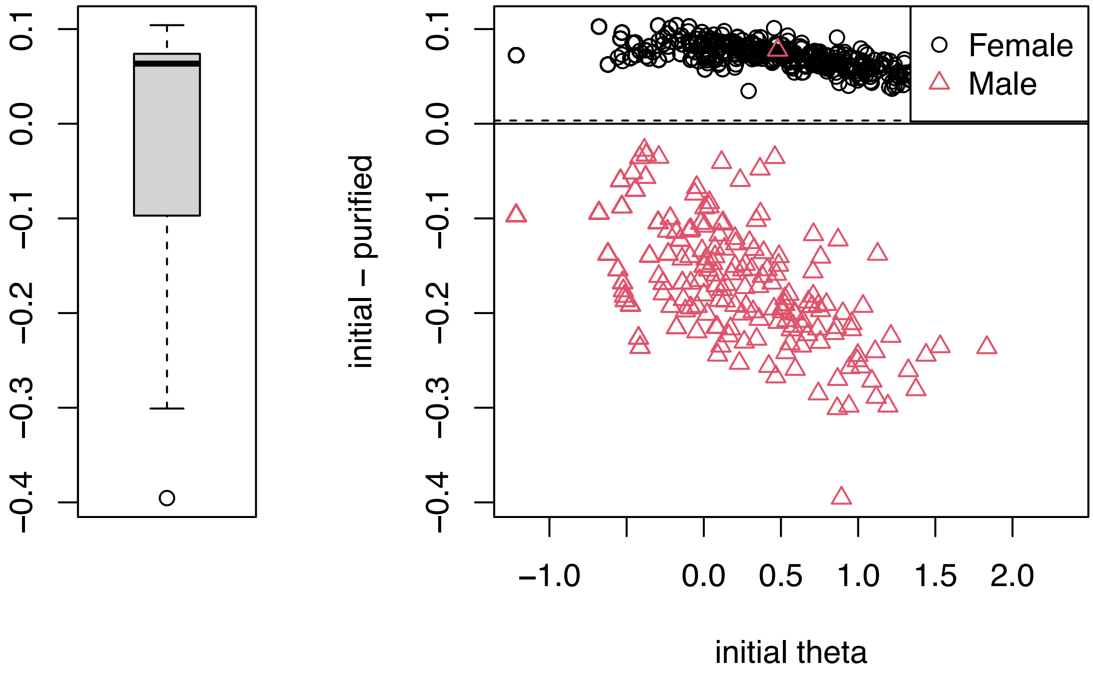

Age-based DIF: Eating Problems

```
## Call:
## lordif::lordif(resp.data = as.data.frame(age.data), group = age)
##
##   Number of DIF groups: 2
##
##   Number of items flagged for DIF: 0 of 5
##
##   Items flagged:
##
##   Number of iterations for purification: 1 of 10
##
##   Detection criterion: Chisqr
##
##   Threshold: alpha = 0.01
```

Back to top

Sad Affect

Site 1

Reliability: Sad Affect

```
## Cronbach's alpha is 0.837.
## Mean item-total correlation is 0.639.
## If each item were dropped:
##      raw_alpha std.alpha G6(smc) average_r S/N alpha se var.r med.r
## Q42       0.75      0.76    0.61      0.61 3.1    0.020    NA  0.61
## Q102      0.87      0.87    0.76      0.76 6.4    0.011    NA  0.76
```

```
## Q100      0.70      0.71    0.55      0.55 2.4    0.024    NA  0.55
```

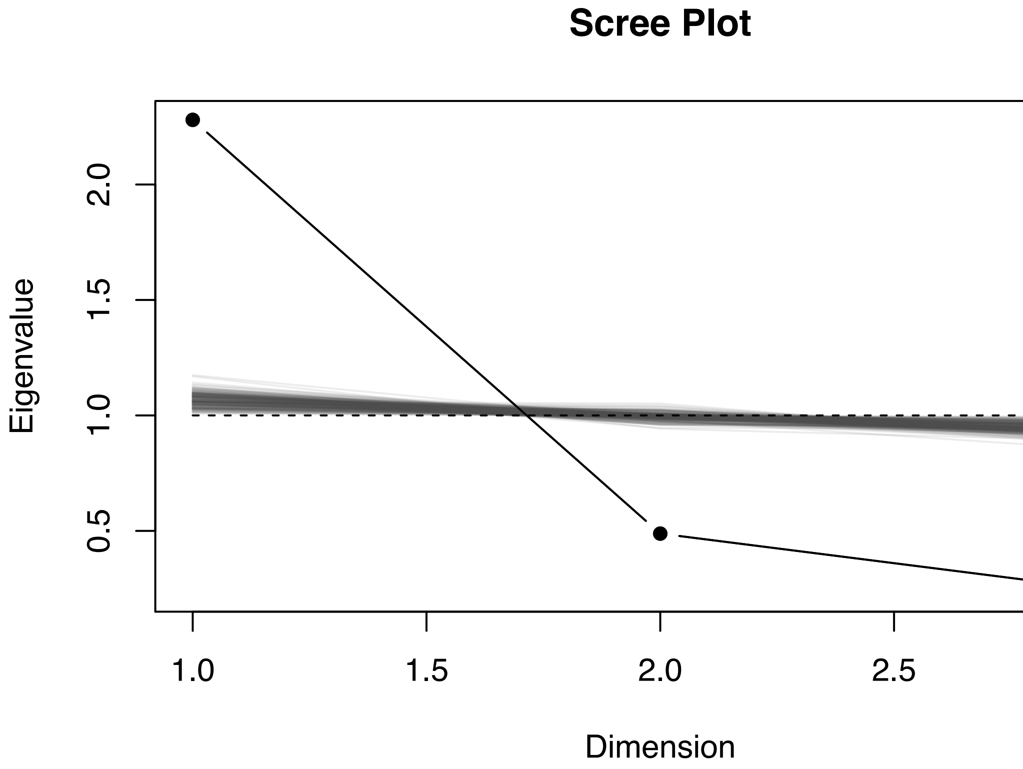

Unidimensionality: Sad Affect

```
## [1] "Ratio of first to second eigenvalues: 4.67"
```

```
## [1] 2.2798353 0.4881968 0.2319679
```

```
## Factor Analysis using method =  minres
## Call: fa(r = grm_obj$X)
## Standardized loadings (pattern matrix) based upon correlation matrix
##       MR1   h2   u2 com
## Q42  0.83 0.68 0.32   1
## Q102 0.66 0.43 0.57   1
## Q100 0.92 0.85 0.15   1
##
##                 MR1
## SS loadings    1.97
## Proportion Var 0.66
##
## Mean item complexity =  1
## Test of the hypothesis that 1 factor is sufficient.
##
## The degrees of freedom for the null model are  3  and the objective function was  1.35 with Chi Squa
## The degrees of freedom for the model are 0  and the objective function was  0
##
## The root mean square of the residuals (RMSR) is  0
## The df corrected root mean square of the residuals is  NA
##
## The harmonic number of observations is  598 with the empirical chi square  0  with prob <  NA
## The total number of observations was  617  with Likelihood Chi Square =  0  with prob <  NA
##
```

```
## Tucker Lewis Index of factoring reliability =  -Inf
## Fit based upon off diagonal values = 1
## Measures of factor score adequacy
##                                                   MR1
## Correlation of (regression) scores with factors   0.95
## Multiple R square of scores with factors          0.90
## Minimum correlation of possible factor scores     0.79
```

Graded-Response Model: Sad Affect

```
##      Extrmt1 Extrmt2 Extrmt3 Extrmt4 Extrmt5 Extrmt6 Dscrmn
## Q42   -1.823  -1.087  -0.646  -0.112   0.390   0.896  3.013
## Q102  -1.007  -0.388  -0.009   0.465   1.077   1.782  1.722
## Q100  -1.707  -1.041  -0.557  -0.013   0.462   1.011  4.146
```

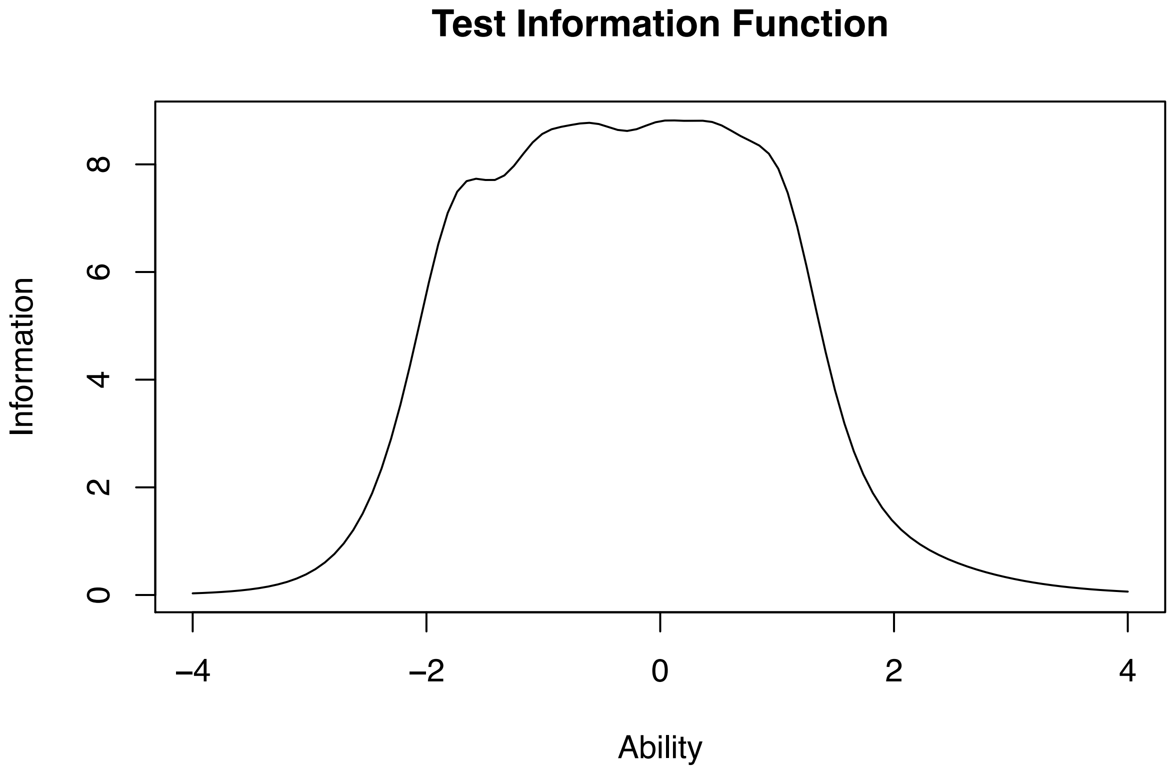

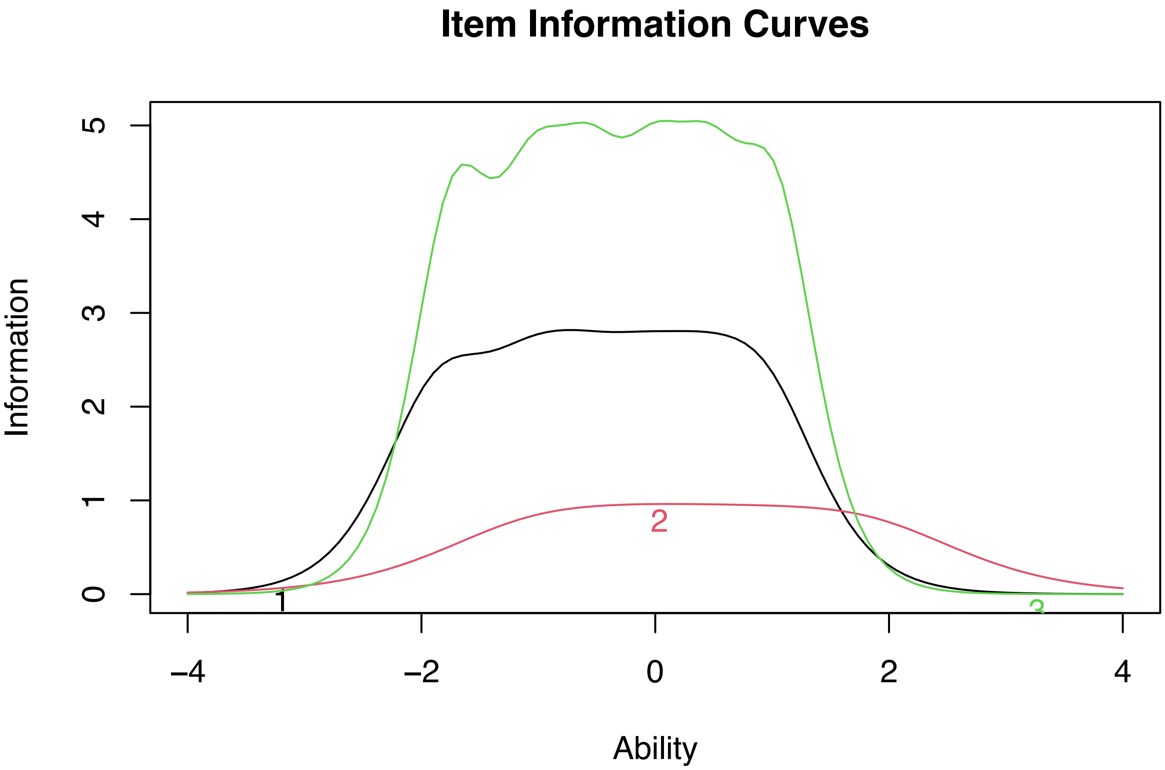

Back to top

Site 2

Reliability: Sad Affect

```
## Cronbach's alpha is 0.838.
## Mean item-total correlation is 0.638.
## If each item were dropped:
##      raw_alpha std.alpha G6(smc) average_r S/N alpha se var.r med.r
## Q42       0.74      0.75    0.59      0.59 2.9    0.021    NA  0.59
## Q102      0.86      0.86    0.76      0.76 6.3    0.011    NA  0.76
## Q100      0.72      0.72    0.56      0.56 2.5    0.023    NA  0.56
```

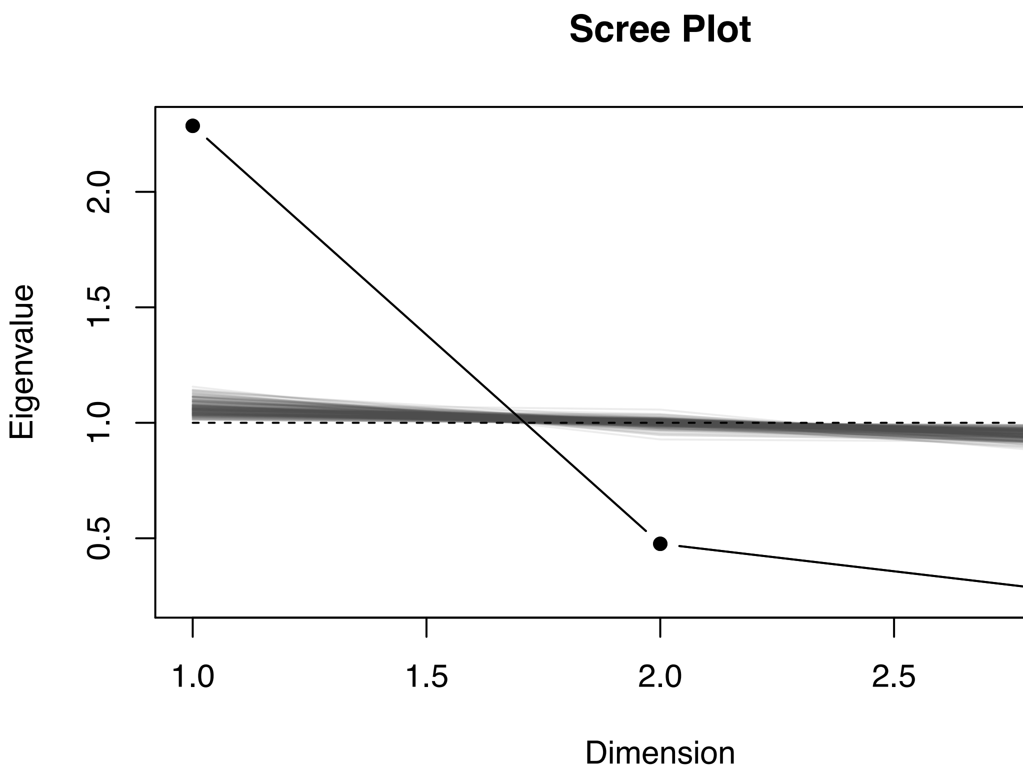

Unidimensionality: Sad Affect

```
## [1] "Ratio of first to second eigenvalues: 4.801"

## [1] 2.2857213 0.4760639 0.2382148

## Factor Analysis using method =  minres
## Call: fa(r = grm_obj$X)
## Standardized loadings (pattern matrix) based upon correlation matrix
##       MR1   h2   u2 com
## Q42  0.85 0.72 0.28   1
## Q102 0.67 0.44 0.56   1
## Q100 0.90 0.80 0.20   1
##
##                 MR1
## SS loadings    1.97
## Proportion Var 0.66
##
## Mean item complexity =  1
## Test of the hypothesis that 1 factor is sufficient.
##
## The degrees of freedom for the null model are  3  and the objective function was  1.35 with Chi Squa
## The degrees of freedom for the model are 0  and the objective function was  0
##
## The root mean square of the residuals (RMSR) is  0
## The df corrected root mean square of the residuals is  NA
##
## The harmonic number of observations is  575 with the empirical chi square  0  with prob <  NA
## The total number of observations was  596  with Likelihood Chi Square =  0  with prob <  NA
##
## Tucker Lewis Index of factoring reliability =  -Inf
## Fit based upon off diagonal values = 1
## Measures of factor score adequacy
```

```
##                                                    MR1
## Correlation of (regression) scores with factors   0.94
## Multiple R square of scores with factors          0.88
## Minimum correlation of possible factor scores     0.76
```

Graded-Response Model: Sad Affect

```
##      Extrmt1 Extrmt2 Extrmt3 Extrmt4 Extrmt5 Extrmt6 Dscrmn
## Q42   -1.344  -0.626  -0.233   0.234   0.665   1.073  3.479
## Q102  -0.447   0.055   0.492   0.914   1.416   1.951  1.923
## Q100  -1.301  -0.657  -0.104   0.390   0.885   1.447  3.952
```

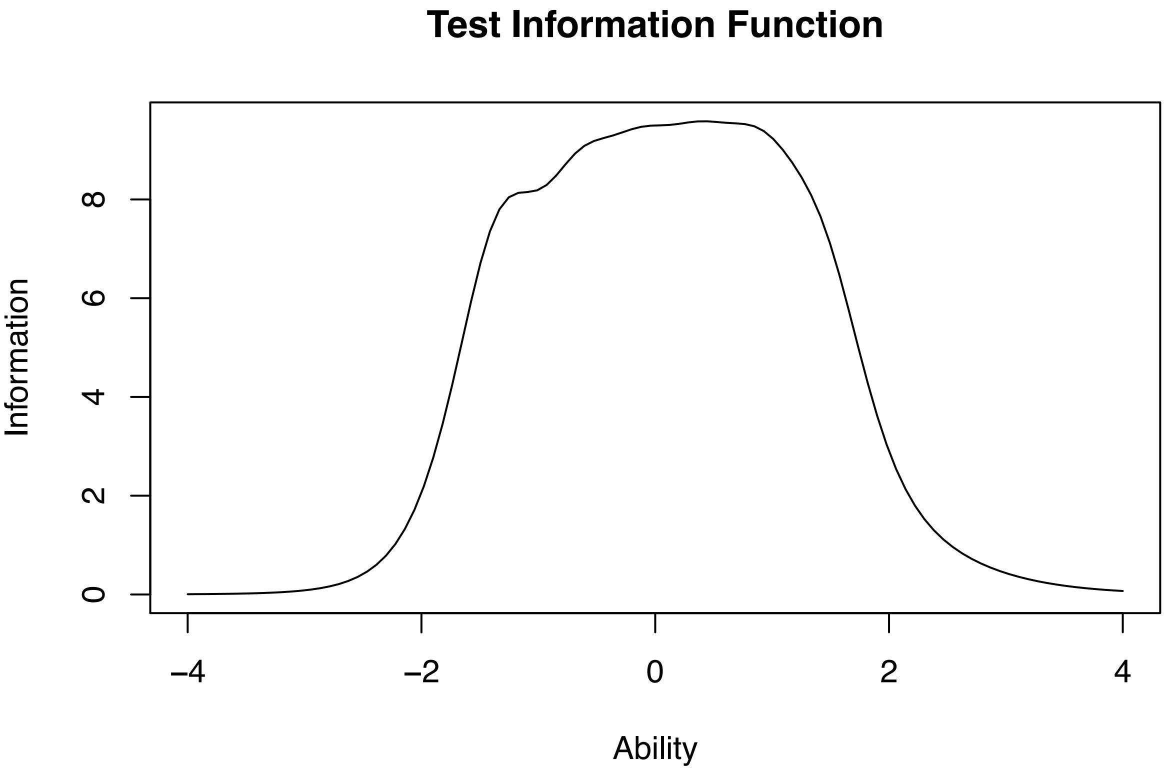

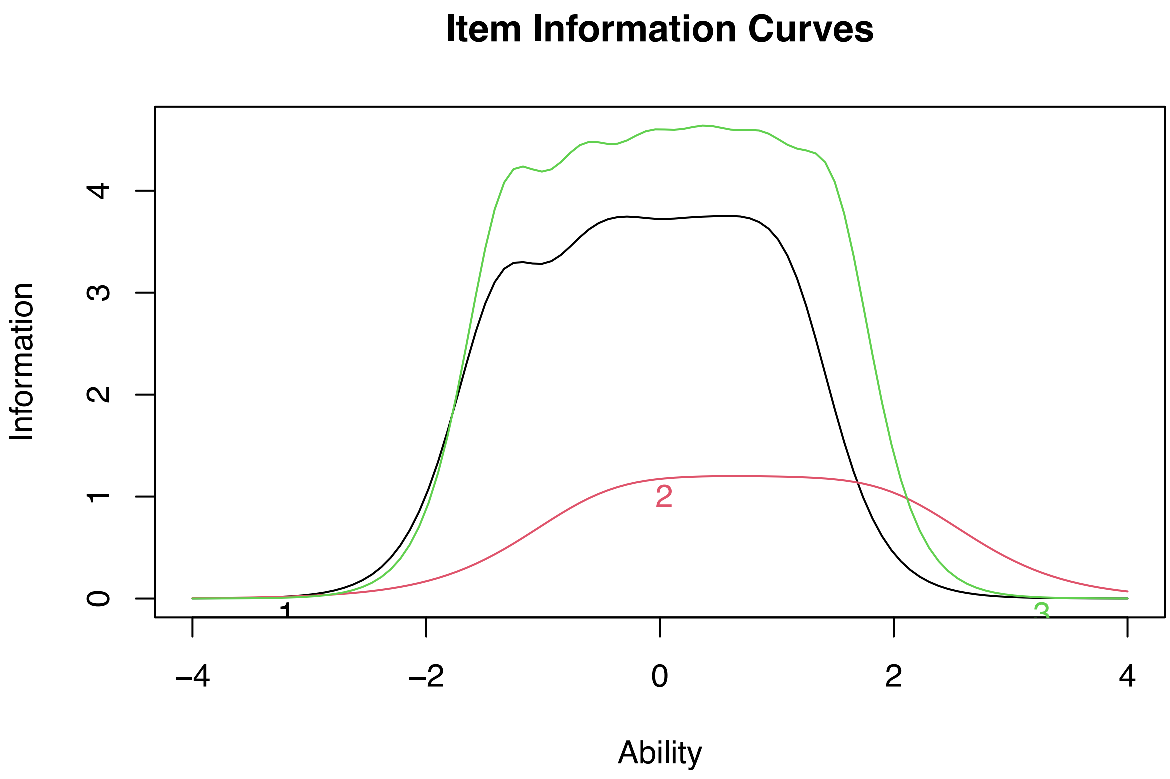

Back to top

Gender-based DIF: Sad Affect

```
## No Gender-based DIF detected
```

Age-based DIF: Sad Affect

```
## No age-based DIF detected
```

Back to top

Somatic Anxiety

Site 1

Reliability: Somatic Anxiety

```
## Cronbach's alpha is 0.803.
## Mean item-total correlation is 0.456.
## If each item were dropped:
##      raw_alpha std.alpha G6(smc) average_r S/N alpha se  var.r med.r
## Q51       0.76      0.76    0.71      0.44 3.2    0.016 0.0045  0.45
## Q103      0.78      0.78    0.73      0.47 3.6    0.015 0.0026  0.47
## Q75       0.76      0.76    0.71      0.44 3.2    0.016 0.0036  0.44
## Q3        0.78      0.78    0.73      0.47 3.6    0.015 0.0017  0.48
## Q53       0.76      0.76    0.71      0.45 3.2    0.016 0.0041  0.46
```

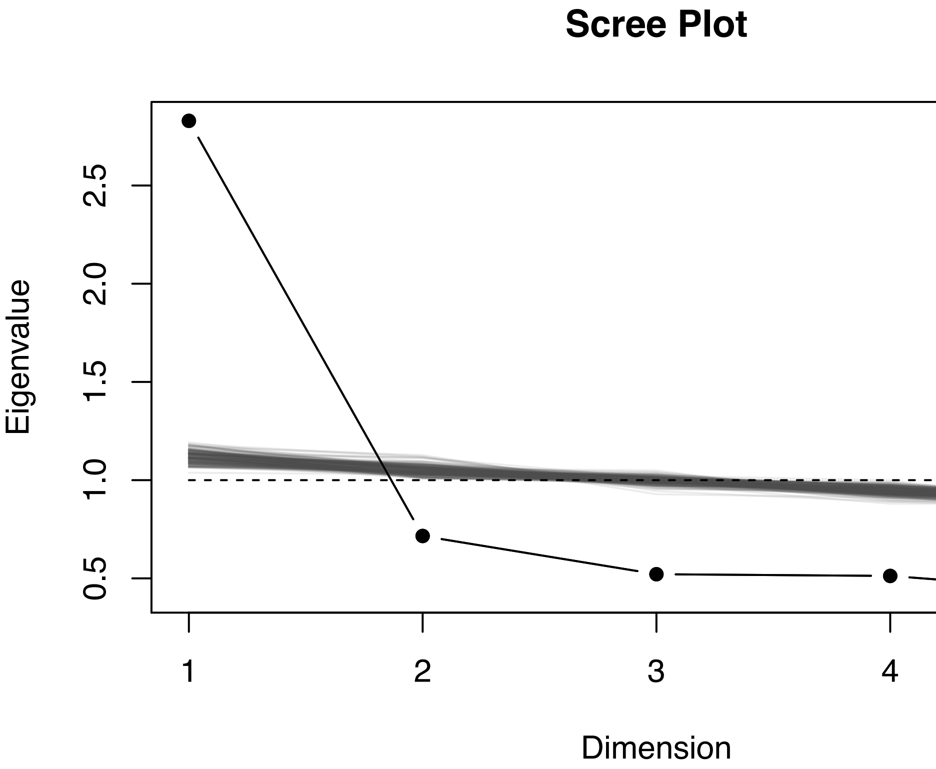

Unidimensionality: Somatic Anxiety

```
## [1] "Ratio of first to second eigenvalues: 3.953"

## [1] 2.8291224 0.7157431 0.5210768 0.5123736 0.4216841

## Factor Analysis using method =  minres
## Call: fa(r = grm_obj$X)
## Standardized loadings (pattern matrix) based upon correlation matrix
##       MR1   h2   u2 com
## Q51  0.71 0.50 0.50   1
## Q103 0.64 0.40 0.60   1
## Q75  0.72 0.51 0.49   1
## Q3   0.63 0.39 0.61   1
## Q53  0.69 0.48 0.52   1
##
##                 MR1
## SS loadings    2.29
## Proportion Var 0.46
##
## Mean item complexity =  1
## Test of the hypothesis that 1 factor is sufficient.
##
## The degrees of freedom for the null model are  10  and the objective function was  1.48 with Chi Squa
## The degrees of freedom for the model are 5  and the objective function was  0.06
##
## The root mean square of the residuals (RMSR) is  0.04
## The df corrected root mean square of the residuals is  0.06
##
## The harmonic number of observations is  595 with the empirical chi square  22.37  with prob <  0.0004
## The total number of observations was  617  with Likelihood Chi Square =  37.38  with prob <  5e-07
##
## Tucker Lewis Index of factoring reliability =  0.928
```

```
## RMSEA index =  0.102  and the 90 % confidence intervals are  0.073 0.134
## BIC =  5.25
## Fit based upon off diagonal values = 0.99
## Measures of factor score adequacy
##                                                    MR1
## Correlation of (regression) scores with factors   0.90
## Multiple R square of scores with factors          0.81
## Minimum correlation of possible factor scores     0.62
```

Graded-Response Model: Somatic Anxiety

```
##      Extrmt1 Extrmt2 Extrmt3 Extrmt4 Extrmt5 Extrmt6 Dscrmn
## Q51   -2.462  -1.833  -1.241  -0.739  -0.029   0.714  2.072
## Q103  -0.751  -0.048   0.507   1.045   1.609   2.268  1.753
## Q75   -1.581  -1.031  -0.618  -0.198   0.385   1.008  2.069
## Q3    -1.160  -0.682  -0.248   0.226   0.774   1.345  1.495
## Q53   -1.063  -0.470  -0.104   0.316   0.828   1.448  1.785
```

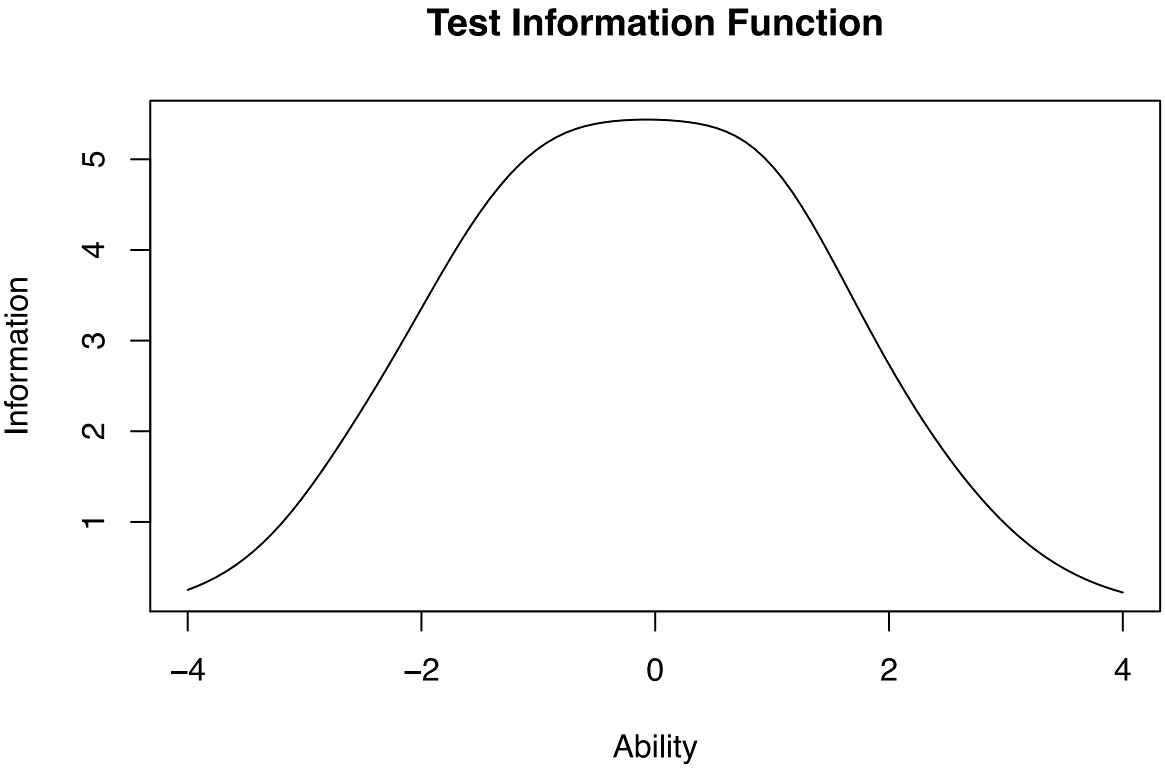

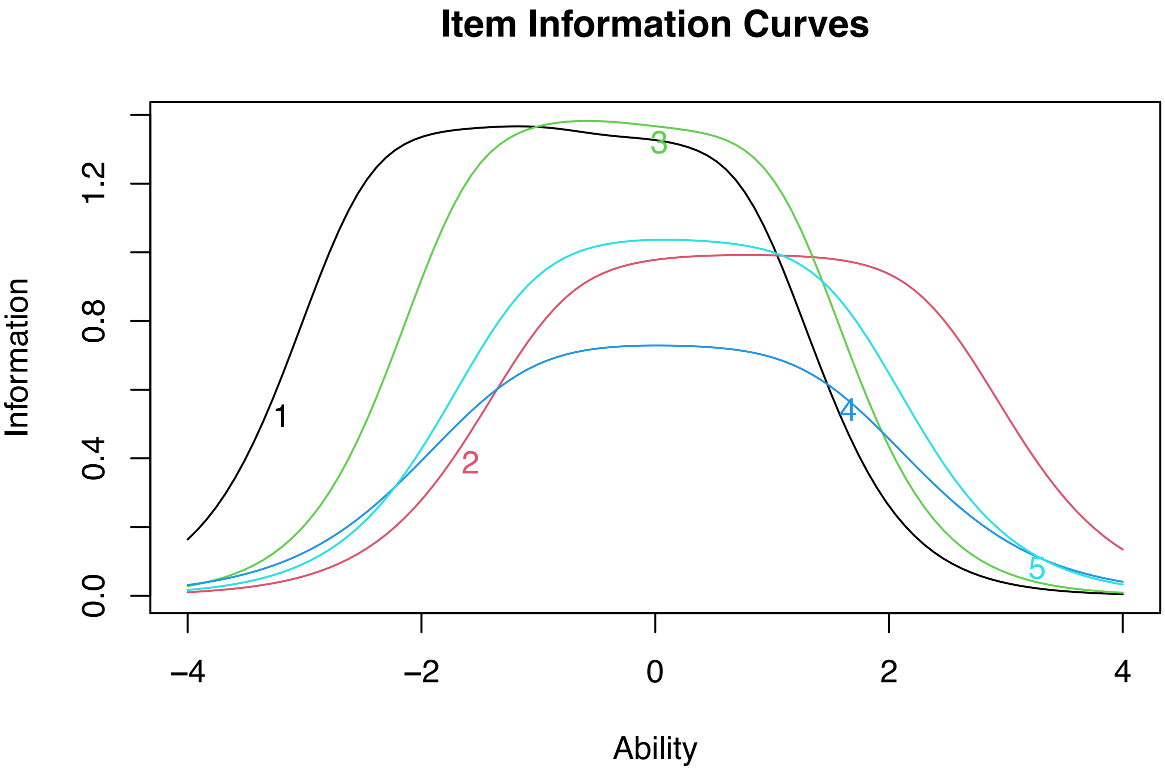

Back to top

Site 2

Reliability: Somatic Anxiety

```
## Cronbach's alpha is 0.808.
## Mean item-total correlation is 0.459.
## If each item were dropped:
##      raw_alpha std.alpha G6(smc) average_r S/N alpha se  var.r med.r
## Q51       0.77      0.77    0.73      0.45 3.3    0.015 0.0068  0.47
## Q103      0.79      0.79    0.75      0.48 3.7    0.014 0.0060  0.49
## Q75       0.75      0.76    0.71      0.44 3.1    0.016 0.0055  0.45
## Q3        0.79      0.79    0.75      0.49 3.9    0.014 0.0023  0.48
## Q53       0.75      0.75    0.71      0.44 3.1    0.017 0.0078  0.42
```

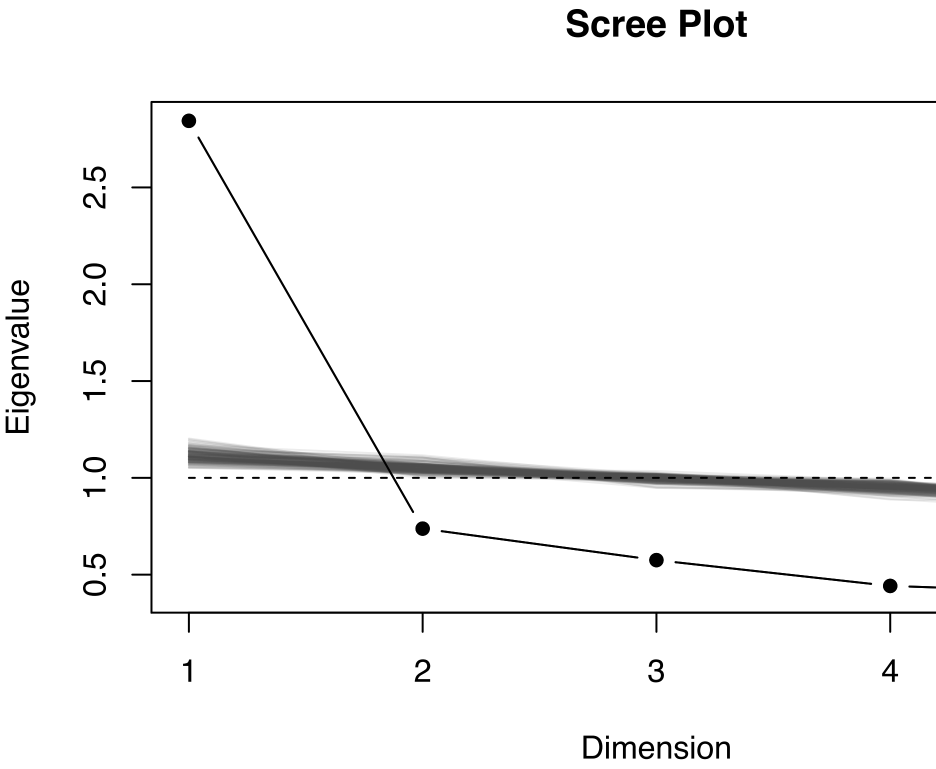

Unidimensionality: Somatic Anxiety

```
## [1] "Ratio of first to second eigenvalues: 3.855"

## [1] 2.8439146 0.7377162 0.5750519 0.4418454 0.4014719

## Factor Analysis using method =  minres
## Call: fa(r = grm_obj$X)
## Standardized loadings (pattern matrix) based upon correlation matrix
##       MR1   h2   u2 com
## Q51  0.69 0.48 0.52   1
## Q103 0.62 0.38 0.62   1
## Q75  0.75 0.56 0.44   1
## Q3   0.59 0.35 0.65   1
## Q53  0.74 0.55 0.45   1
##
##                 MR1
## SS loadings    2.32
## Proportion Var 0.46
##
## Mean item complexity =  1
## Test of the hypothesis that 1 factor is sufficient.
##
## The degrees of freedom for the null model are  10  and the objective function was  1.54 with Chi Squa
## The degrees of freedom for the model are 5  and the objective function was  0.08
##
## The root mean square of the residuals (RMSR) is  0.05
## The df corrected root mean square of the residuals is  0.07
##
## The harmonic number of observations is  578 with the empirical chi square  26.46  with prob <  7.3e-0
## The total number of observations was  596  with Likelihood Chi Square =  46.14  with prob <  8.5e-09
##
## Tucker Lewis Index of factoring reliability =  0.909
```

```
## RMSEA index =  0.117  and the 90 % confidence intervals are  0.088 0.15
## BIC =  14.18
## Fit based upon off diagonal values = 0.99
## Measures of factor score adequacy
##                                                    MR1
## Correlation of (regression) scores with factors   0.91
## Multiple R square of scores with factors          0.82
## Minimum correlation of possible factor scores     0.64
```

Graded-Response Model: Somatic Anxiety

```
##      Extrmt1 Extrmt2 Extrmt3 Extrmt4 Extrmt5 Extrmt6 Dscrmn
## Q51   -2.291  -1.404  -0.796  -0.187   0.415   0.961  1.924
## Q103  -0.551   0.138   0.644   1.191   1.592   2.323  1.696
## Q75   -1.313  -0.704  -0.307   0.164   0.642   1.157  2.341
## Q3    -1.028  -0.394   0.199   0.615   1.274   1.862  1.364
## Q53   -0.869  -0.338   0.119   0.506   0.922   1.453  1.998
```

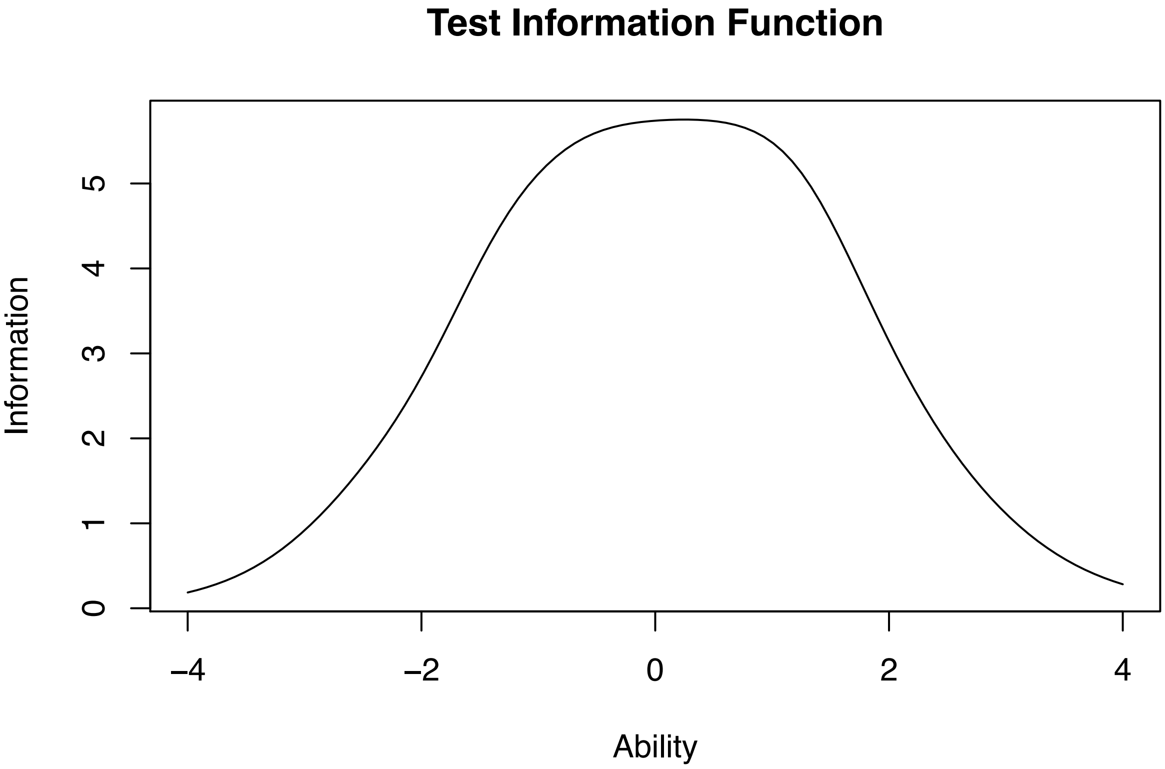

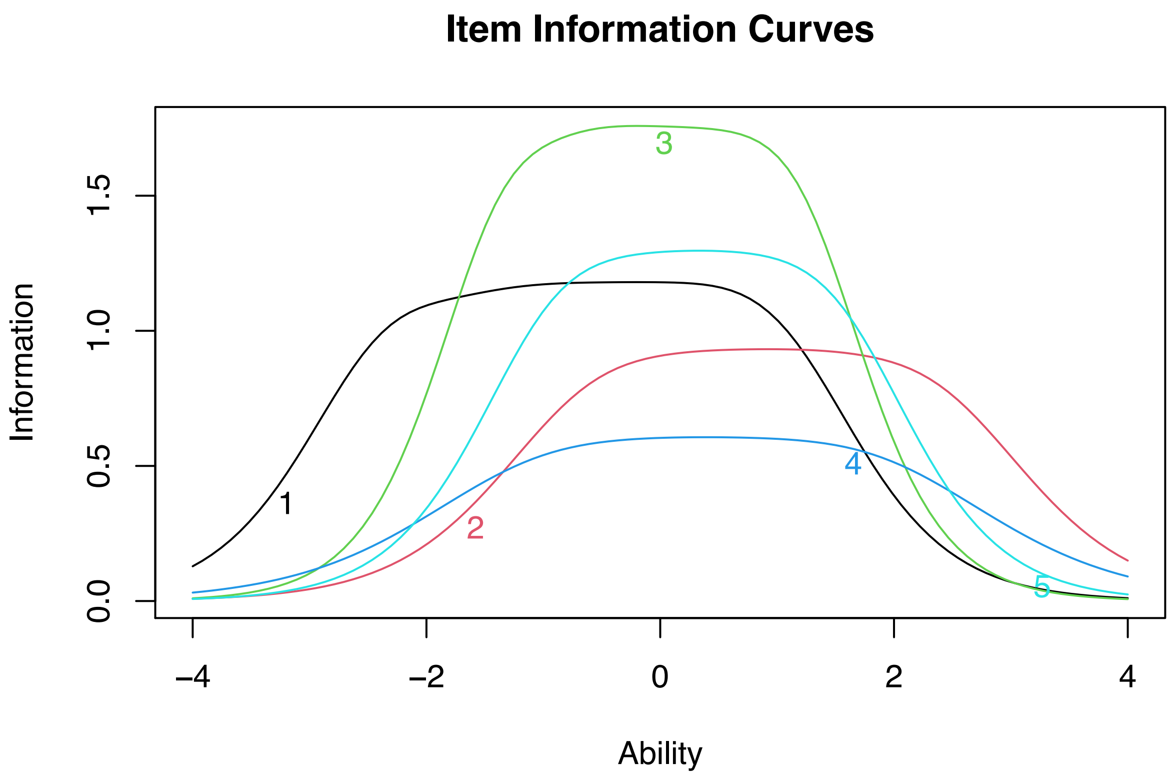

Back to top

Site DIF

```
## Call:
## lordif::lordif(resp.data = as.data.frame(merged_data), group = site)
##
##   Number of DIF groups: 2
##
##   Number of items flagged for DIF: 1 of 5
##
##   Items flagged: 1
##
##   Number of iterations for purification: 2 of 10
##
##   Detection criterion: Chisqr
##
##   Threshold: alpha = 0.01
##
##   item ncat  chi12  chi13  chi23
## 1    1    7 0.0001 0.0002 0.2917
## 2    2    7 0.1997 0.3781 0.5837
## 3    3    7 0.1224 0.2884 0.7514
## 4    4    7 0.0725 0.0523 0.1019
## 5    5    7 0.1865 0.3079 0.4345
```

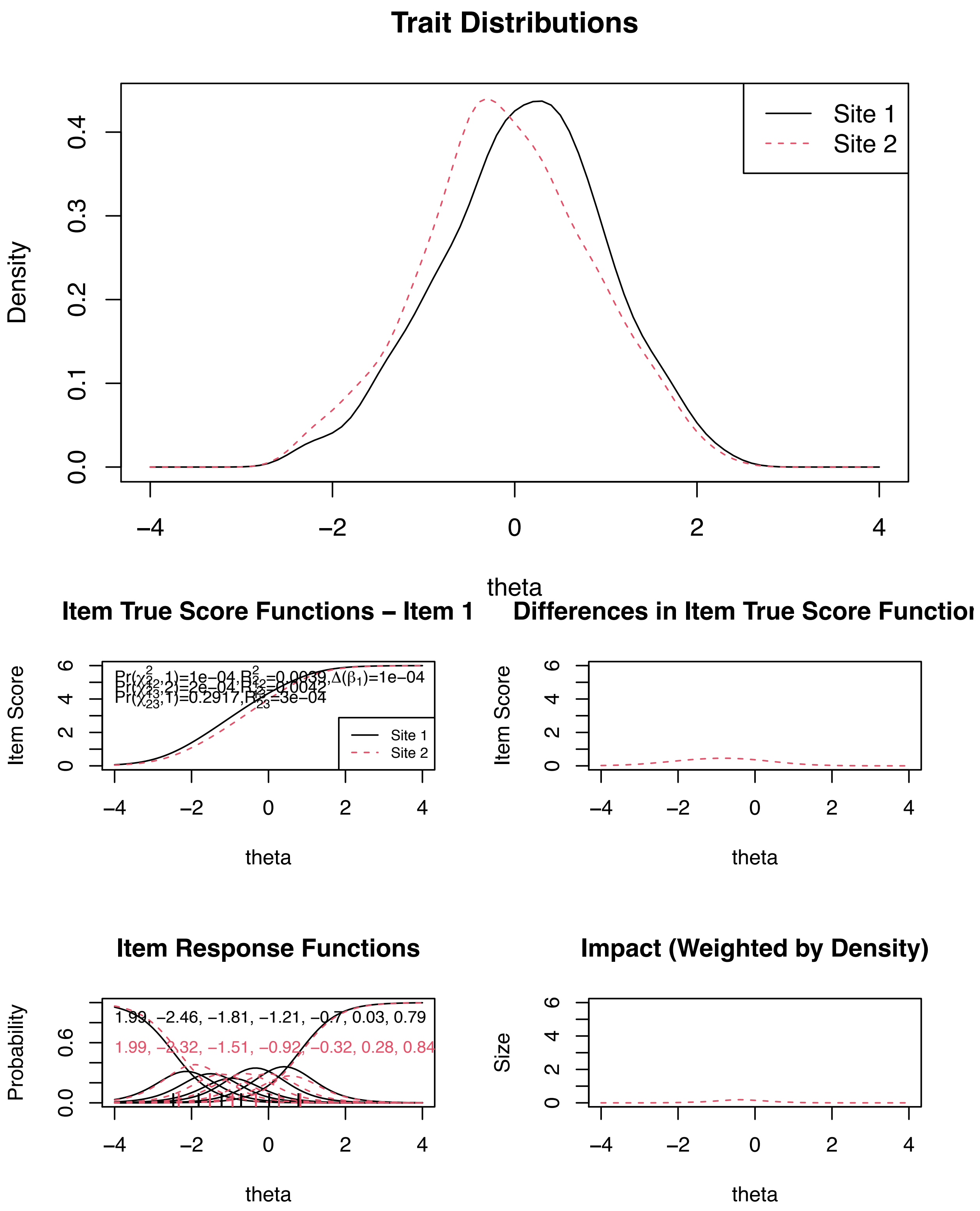
Trait Distributions
Density
theta
Site 1
Site 2
Item True Score Functions – Item 1
Differences in Item True Score Functio
Item Score
Item Response Functions
Probability
1.99, −2.46, −1.81, −1.21, −0.7, 0.03, 0.79
1.99, −2.32, −1.51, −0.92, −0.32, 0.28, 0.84
Impact (Weighted by Density)
Size

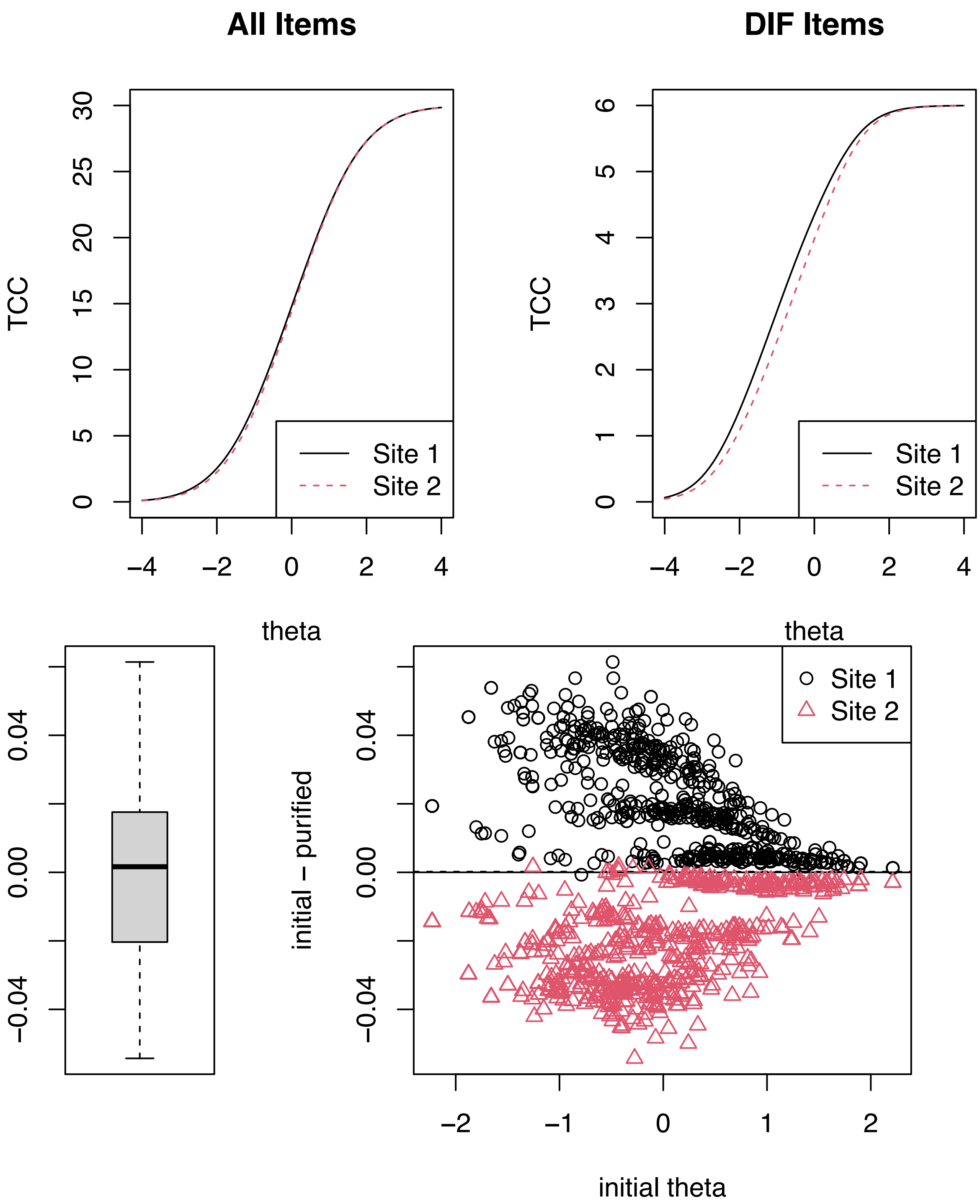

Back to top

Gender-based DIF: Somatic Anxiety

```
## Call:
## lordif::lordif(resp.data = as.data.frame(sex.data), group = sex)
##
##   Number of DIF groups: 2
```

```
##
##   Number of items flagged for DIF: 3 of 5
##
##   Items flagged: 2, 4, 5
##
##   Number of iterations for purification: 3 of 10
##
##   Detection criterion: Chisqr
##
##   Threshold: alpha = 0.01
##
##   item ncat  chi12  chi13  chi23
## 1    1    7 0.8733 0.9795 0.8991
## 2    2    7 0.0000 0.0000 0.0454
## 3    3    7 0.1115 0.0413 0.0500
## 4    4    7 0.0000 0.0000 0.0000
## 5    5    7 0.0058 0.0000 0.0000
```

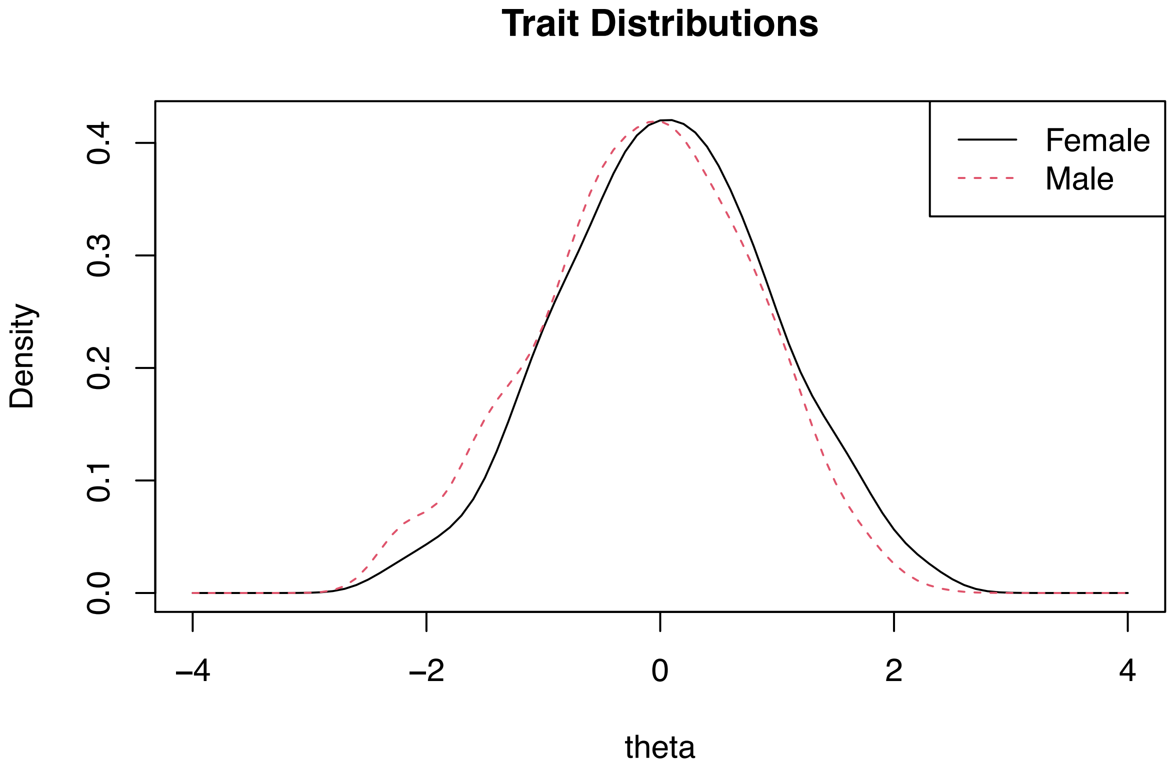

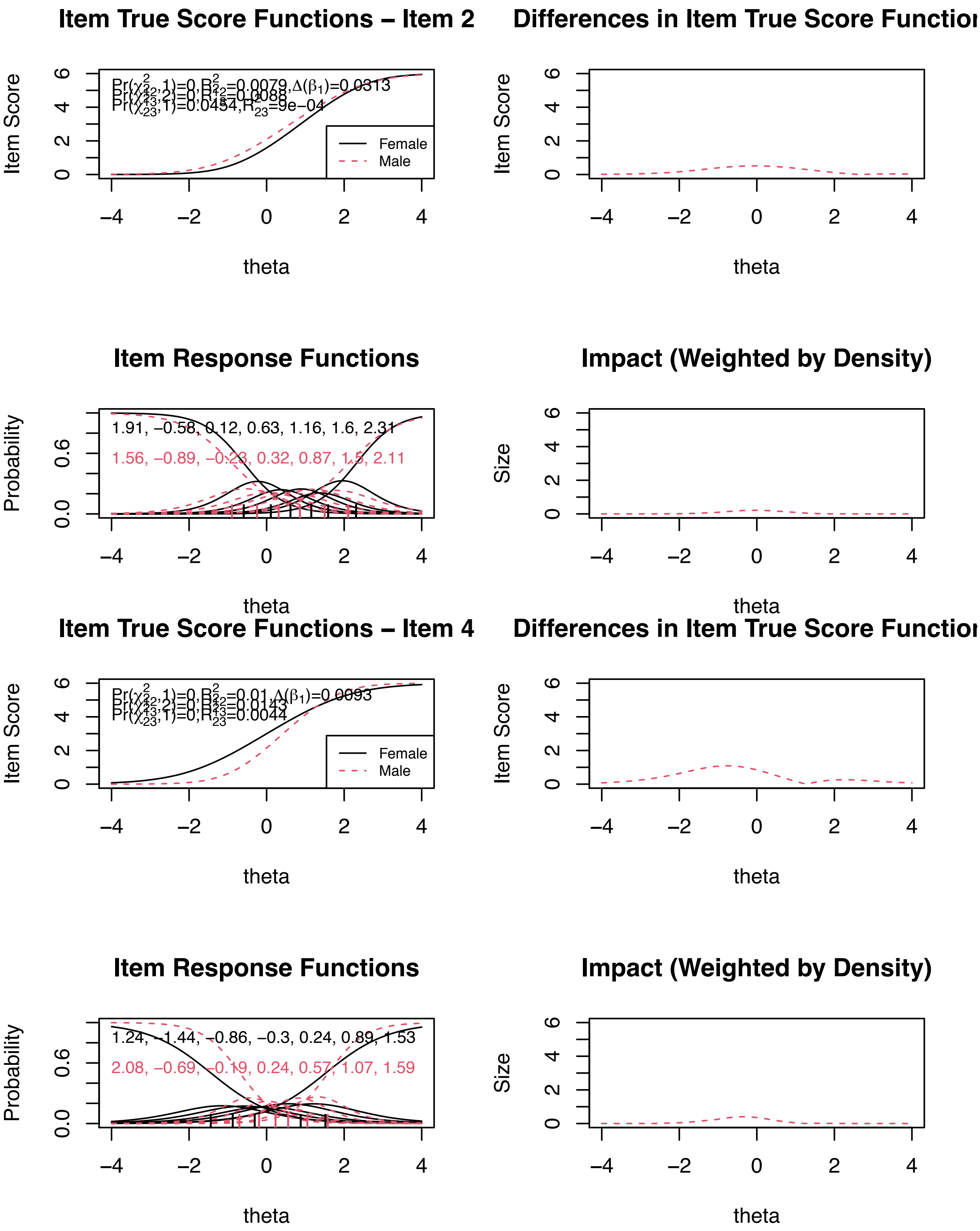

Item True Score Functions – Item 2
Differences in Item True Score Functio
Item Score
theta
Female
Male
Item Response Functions
Impact (Weighted by Density)
Probability
Size
1.91, −0.58, 0.12, 0.63, 1.16, 1.6, 2.31
1.56, −0.89, −0.23, 0.32, 0.87, 1.5, 2.11
Item True Score Functions – Item 4
Differences in Item True Score Functio
1.24, −1.44, −0.86, −0.3, 0.24, 0.89, 1.53
2.08, −0.69, −0.19, 0.24, 0.57, 1.07, 1.59

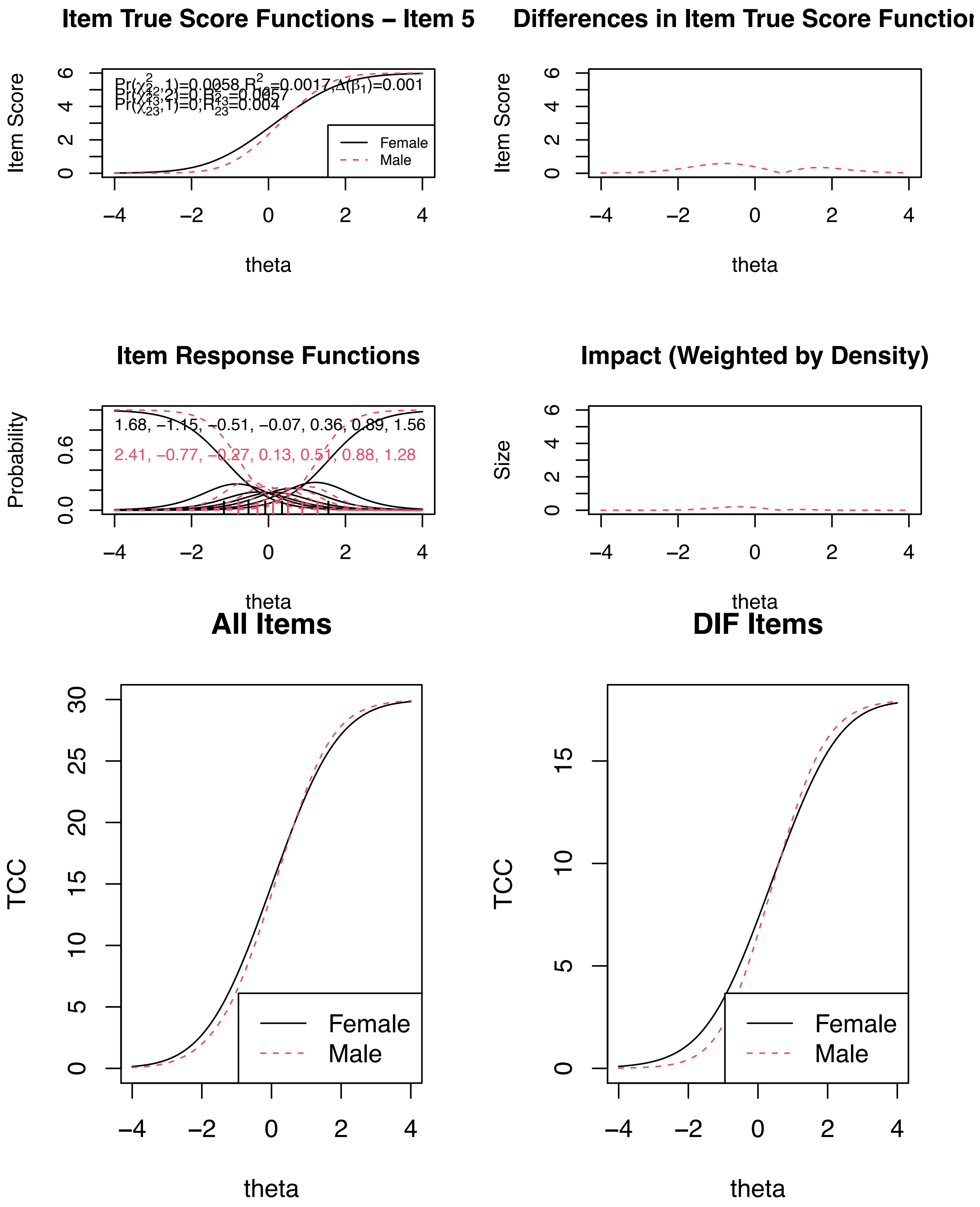
Item True Score Functions – Item 5
Item Score
theta
Female
Male
Differences in Item True Score Functio
Item Score
theta
Item Response Functions
Probability
1.68, −1.15, −0.51, −0.07, 0.36, 0.89, 1.56
2.41, −0.77, −0.27, 0.13, 0.51, 0.88, 1.28
theta
Impact (Weighted by Density)
Size
theta
All Items
TCC
Female
Male
theta
DIF Items
TCC
Female
Male
theta

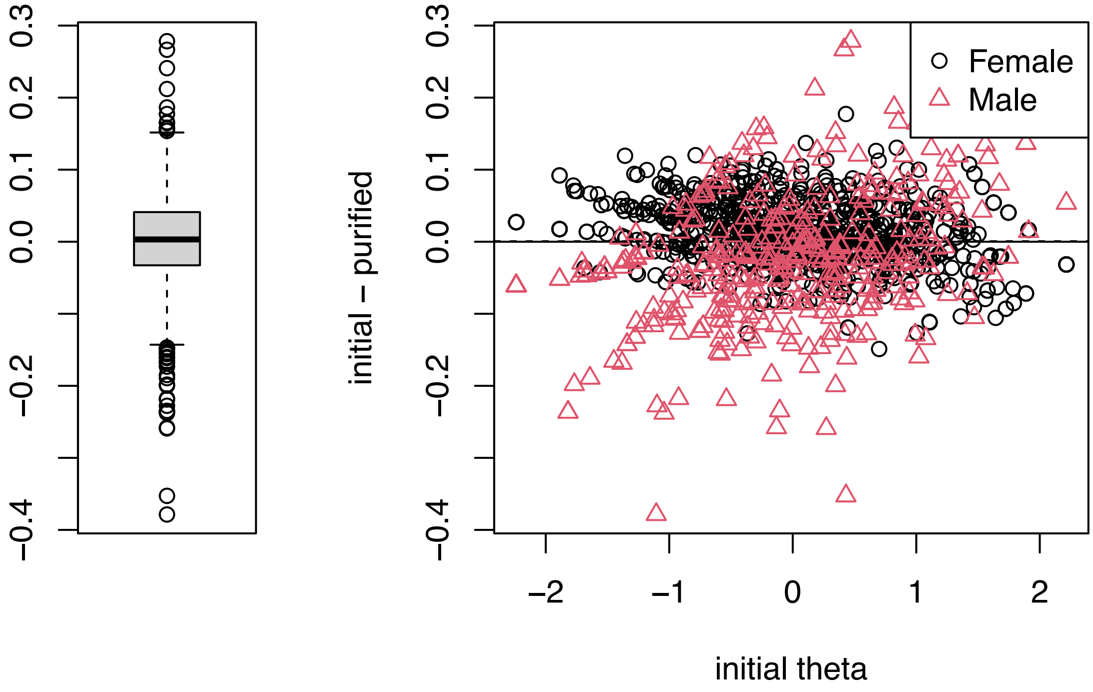

Age-based DIF: Somatic Anxiety

```
## Call:
## lordif::lordif(resp.data = as.data.frame(age.data), group = age)
##
##   Number of DIF groups: 2
##
##   Number of items flagged for DIF: 4 of 5
##
##   Items flagged: 1, 3, 4, 5
##
##   Number of iterations for purification: 2 of 10
##
##   Detection criterion: Chisqr
##
##   Threshold: alpha = 0.01
##
##   item ncat  chi12  chi13  chi23
## 1    1    7 0.0066 0.0001 0.0005
## 2    2    7 0.5523 0.7902 0.7315
## 3    3    7 0.0000 0.0000 0.0711
## 4    4    7 0.0001 0.0001 0.0296
## 5    5    7 0.0057 0.0181 0.5346
```

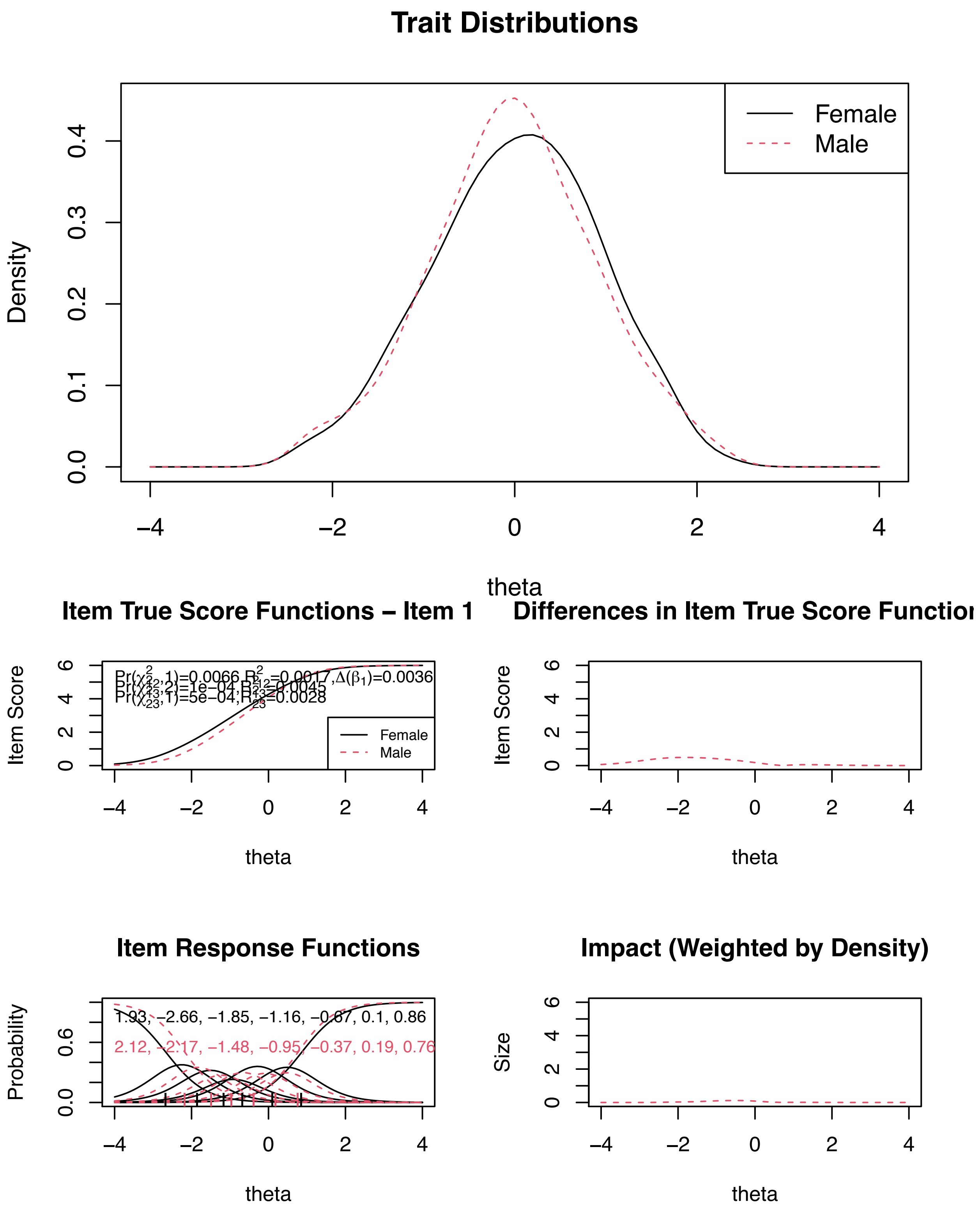

Trait Distributions
Density
theta
Female
Male
Item True Score Functions – Item 1
Differences in Item True Score Functio
Item Score
Item Response Functions
Probability
1.93, –2.66, –1.85, –1.16, –0.67, 0.1, 0.86
2.12, –2.17, –1.48, –0.95, –0.37, 0.19, 0.76
Impact (Weighted by Density)
Size

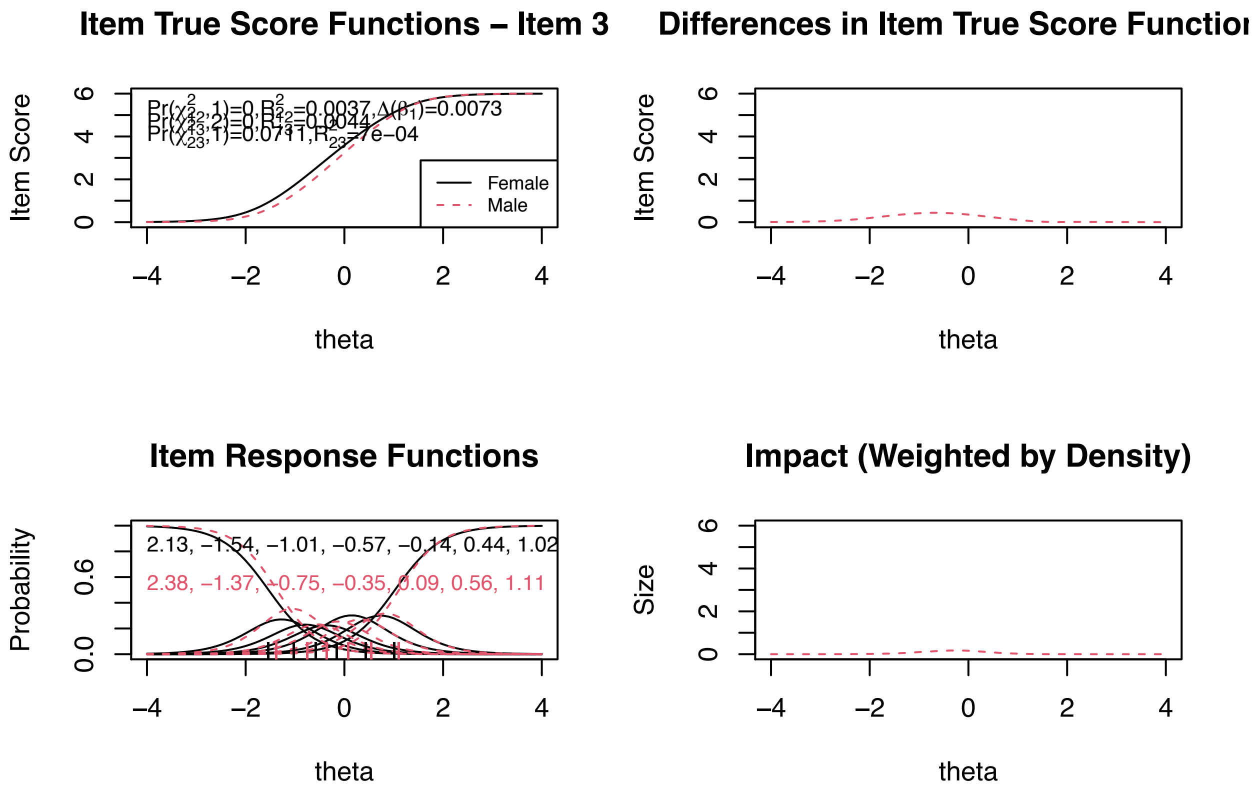
Item True Score Functions – Item 3
Differences in Item True Score Functio
Item Score
theta
Female
Male
Item Response Functions
Impact (Weighted by Density)
Probability
Size
2.13, −1.54, −1.01, −0.57, −0.14, 0.44, 1.02
2.38, −1.37, −0.75, −0.35, 0.09, 0.56, 1.11

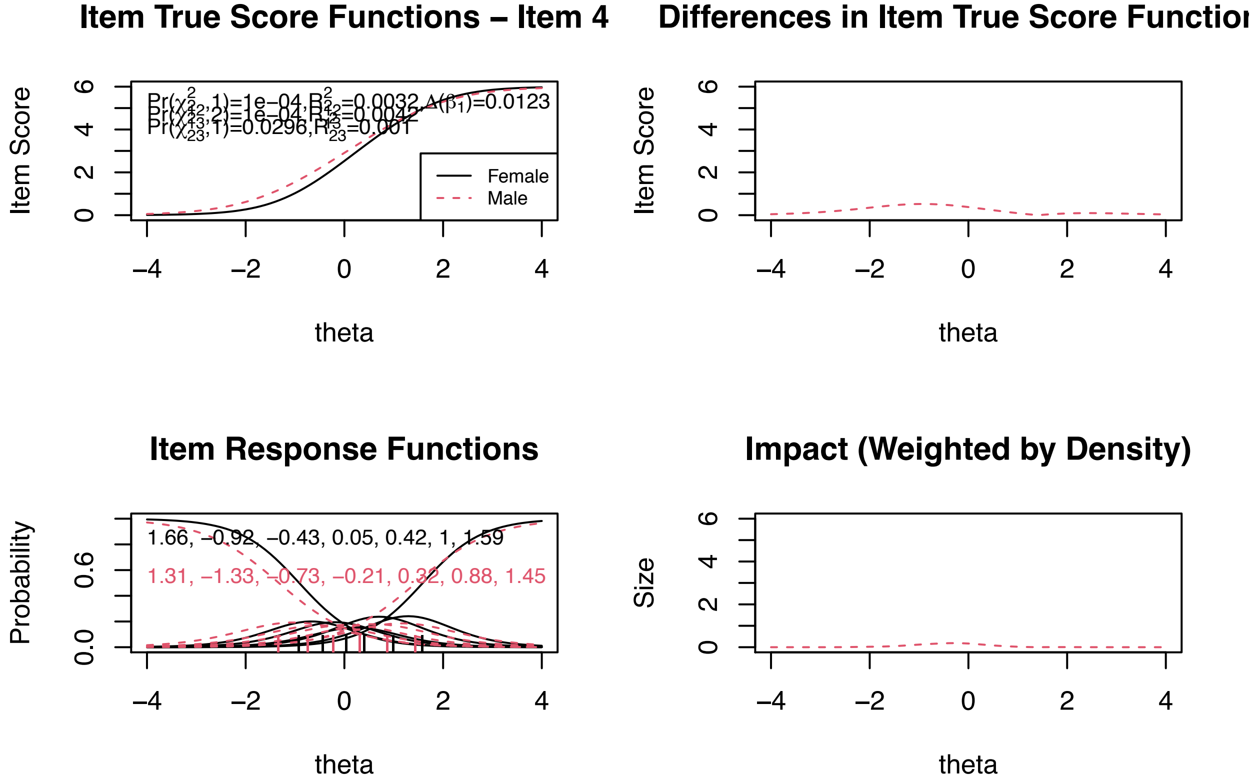
Item True Score Functions – Item 4
Differences in Item True Score Functio
Item Score
theta
Female
Male
Item Response Functions
Impact (Weighted by Density)
Probability
Size
1.66, −0.92, −0.43, 0.05, 0.42, 1, 1.59
1.31, −1.33, −0.73, −0.21, 0.32, 0.88, 1.45

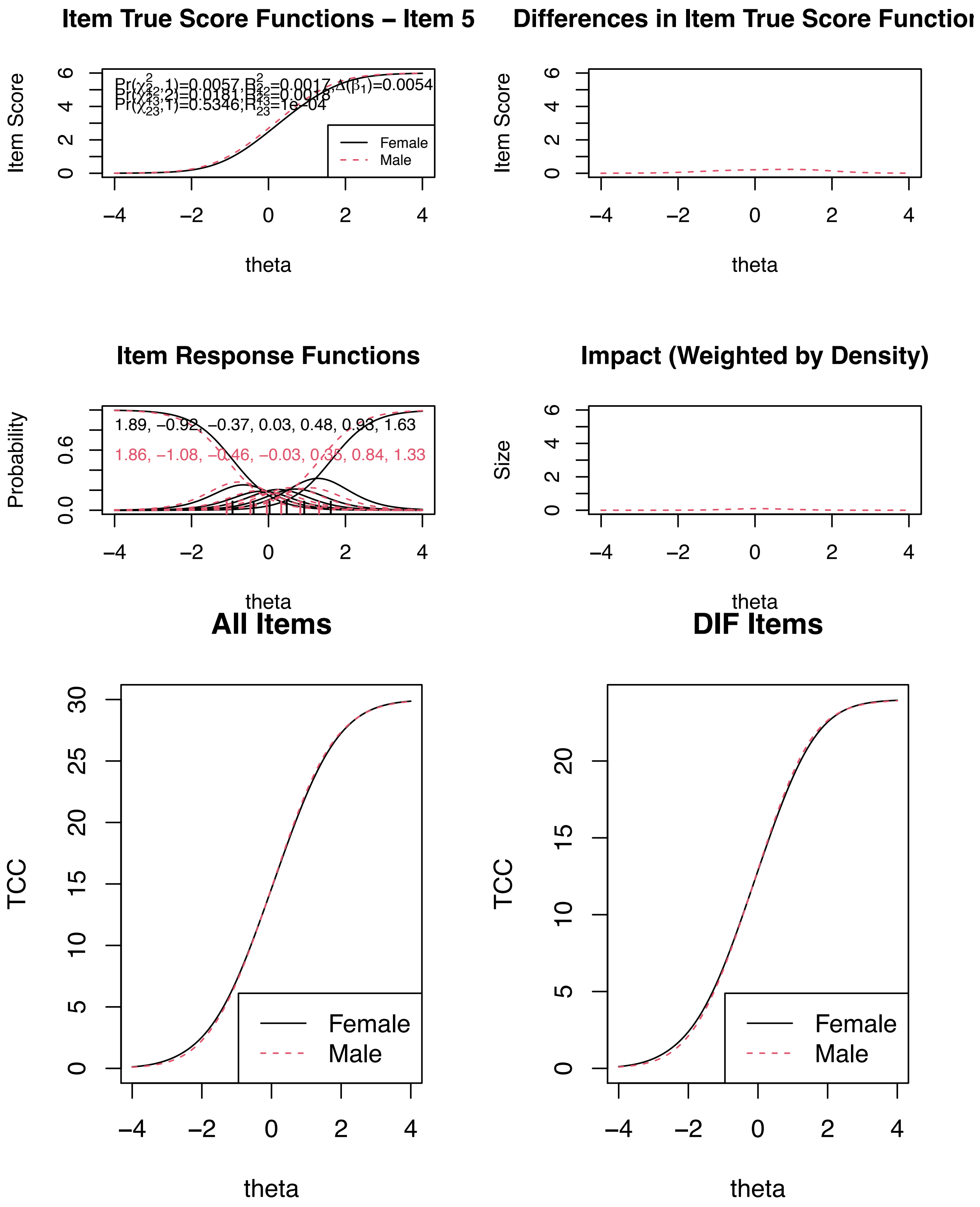
Item True Score Functions – Item 5
Item Score
theta
Female
Male
Differences in Item True Score Functio
Item Score
theta
Item Response Functions
Probability
theta
1.89, −0.92, −0.37, 0.03, 0.48, 0.93, 1.63
1.86, −1.08, −0.46, −0.03, 0.35, 0.84, 1.33
Impact (Weighted by Density)
Size
theta
All Items
TCC
theta
Female
Male
DIF Items
TCC
theta
Female
Male

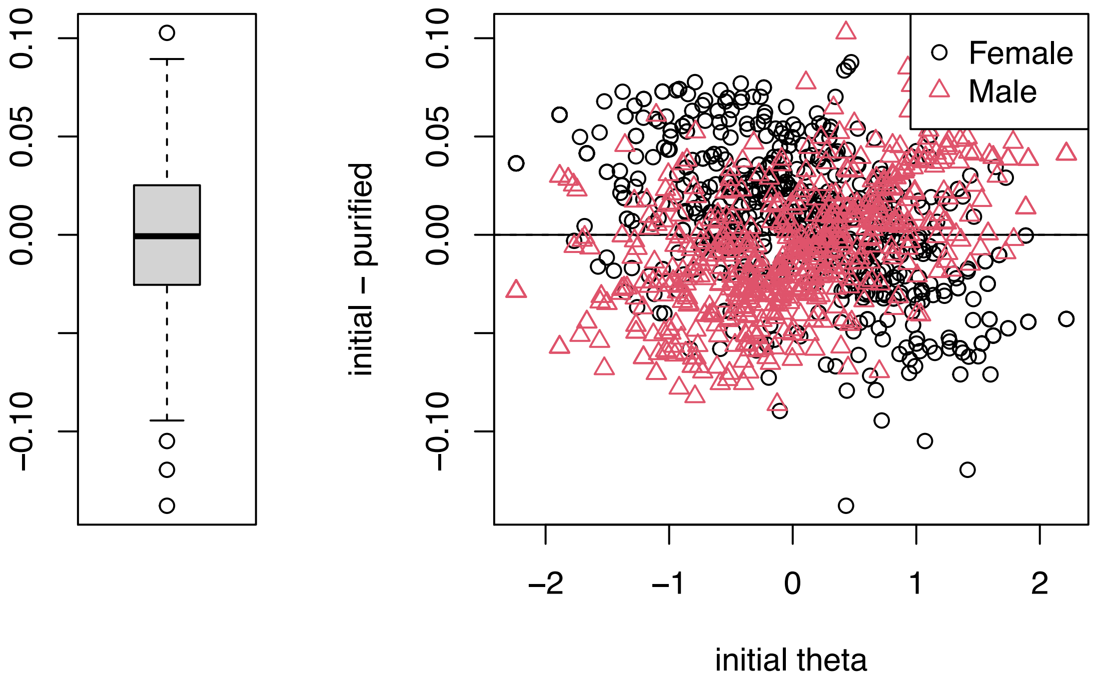

Back to top

Substance Use

Site 1

Reliability: Substance Use

```
## Cronbach's alpha is 0.845.
## Mean item-total correlation is 0.598.
## If each item were dropped:
##      raw_alpha std.alpha G6(smc) average_r S/N alpha se   var.r med.r
## Q59       0.81      0.81    0.74      0.58 4.2    0.013 0.00049  0.57
## Q4        0.79      0.81    0.74      0.59 4.3    0.013 0.00034  0.59
## Q107      0.80      0.82    0.75      0.60 4.6    0.013 0.00113  0.59
## Q35       0.81      0.83    0.76      0.62 4.8    0.012 0.00043  0.61
```

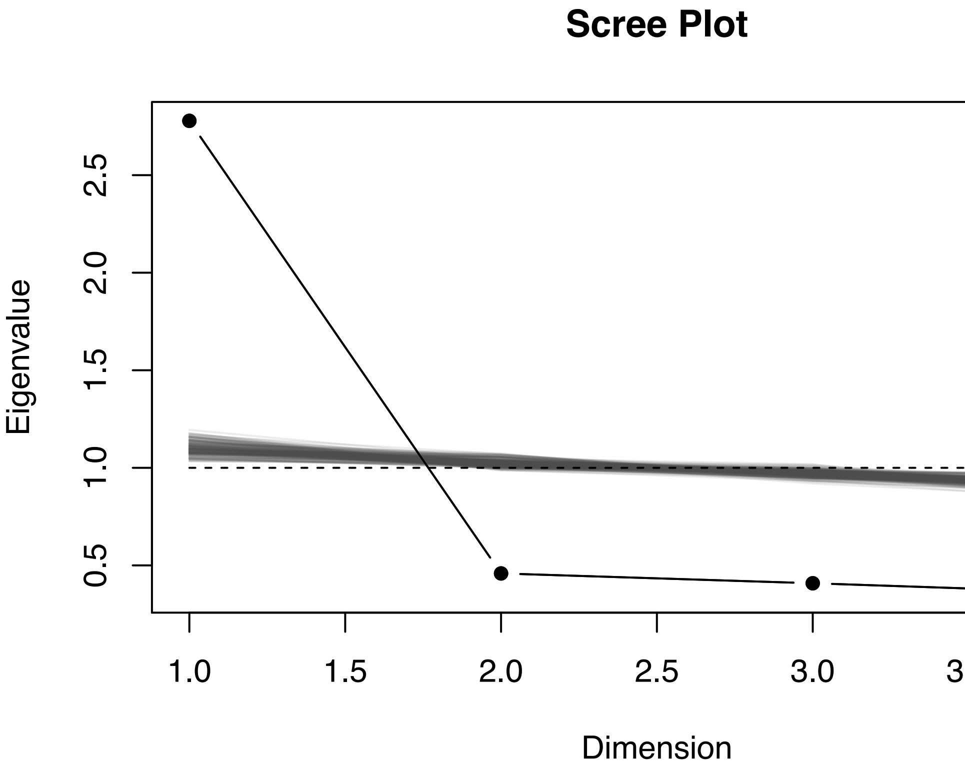

Unidimensionality: Substance Use

```
## [1] "Ratio of first to second eigenvalues: 6.057"

## [1] 2.7783561 0.4586974 0.4083178 0.3546287

## Factor Analysis using method =  minres
## Call: fa(r = grm_obj$X)
## Standardized loadings (pattern matrix) based upon correlation matrix
##       MR1   h2   u2 com
## Q59  0.78 0.61 0.39   1
## Q4   0.80 0.65 0.35   1
## Q107 0.77 0.59 0.41   1
## Q35  0.72 0.52 0.48   1
##
##                 MR1
## SS loadings    2.37
## Proportion Var 0.59
##
## Mean item complexity =  1
## Test of the hypothesis that 1 factor is sufficient.
##
## The degrees of freedom for the null model are  6  and the objective function was  1.69 with Chi Squa
## The degrees of freedom for the model are 2  and the objective function was  0
##
## The root mean square of the residuals (RMSR) is  0.01
## The df corrected root mean square of the residuals is  0.02
##
## The harmonic number of observations is  546 with the empirical chi square  0.62  with prob <  0.73
## The total number of observations was  617  with Likelihood Chi Square =  2.17  with prob <  0.34
##
## Tucker Lewis Index of factoring reliability =  1
## RMSEA index =  0.012  and the 90 % confidence intervals are  0 0.082
```

```
## BIC =  -10.68
## Fit based upon off diagonal values = 1
## Measures of factor score adequacy
##                                                    MR1
## Correlation of (regression) scores with factors   0.93
## Multiple R square of scores with factors          0.86
## Minimum correlation of possible factor scores     0.71
```

Graded-Response Model: Substance Use

```
##      Extrmt1 Extrmt2 Extrmt3 Extrmt4 Extrmt5 Extrmt6 Dscrmn
## Q59   -0.737  -1.144  -1.440  -1.781  -2.187  -2.403 -3.399
## Q4    -1.129  -1.802  -2.154  -2.321  -2.595  -2.741 -3.435
## Q107  -1.326  -1.790  -2.011  -2.285  -2.532  -2.711 -3.324
## Q35   -1.414  -1.848  -2.114  -2.354  -2.649  -3.064 -3.149
```

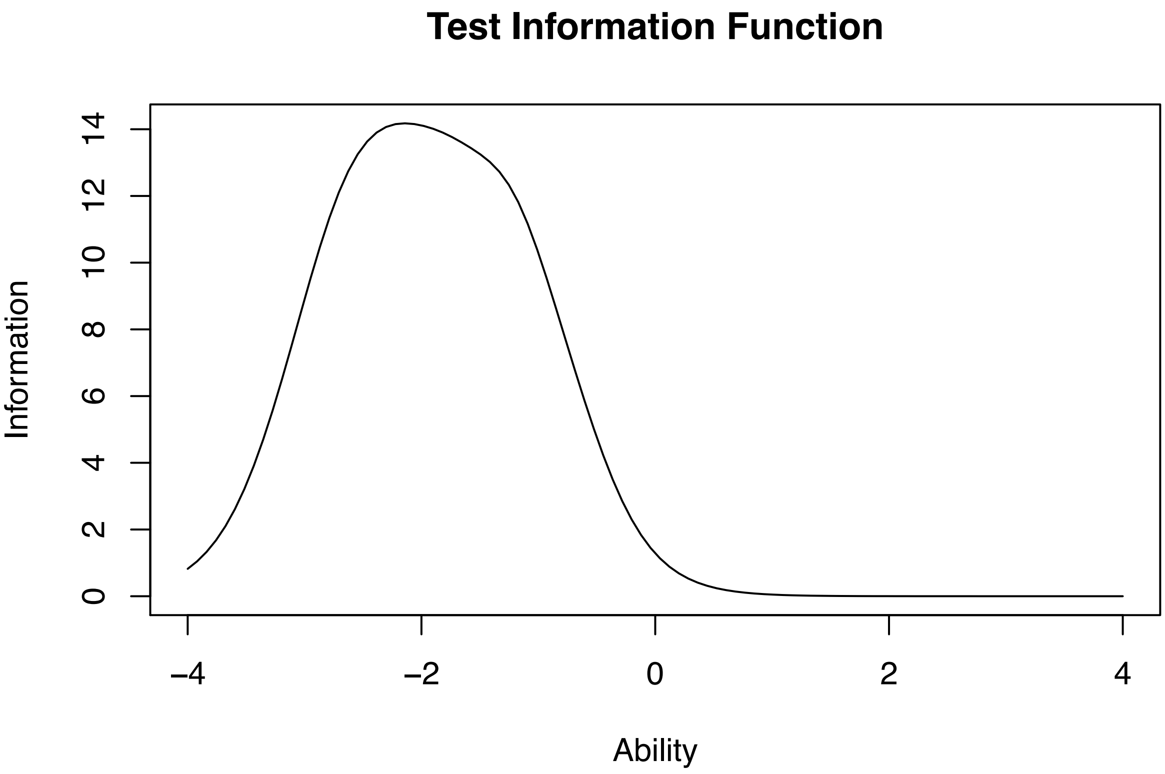

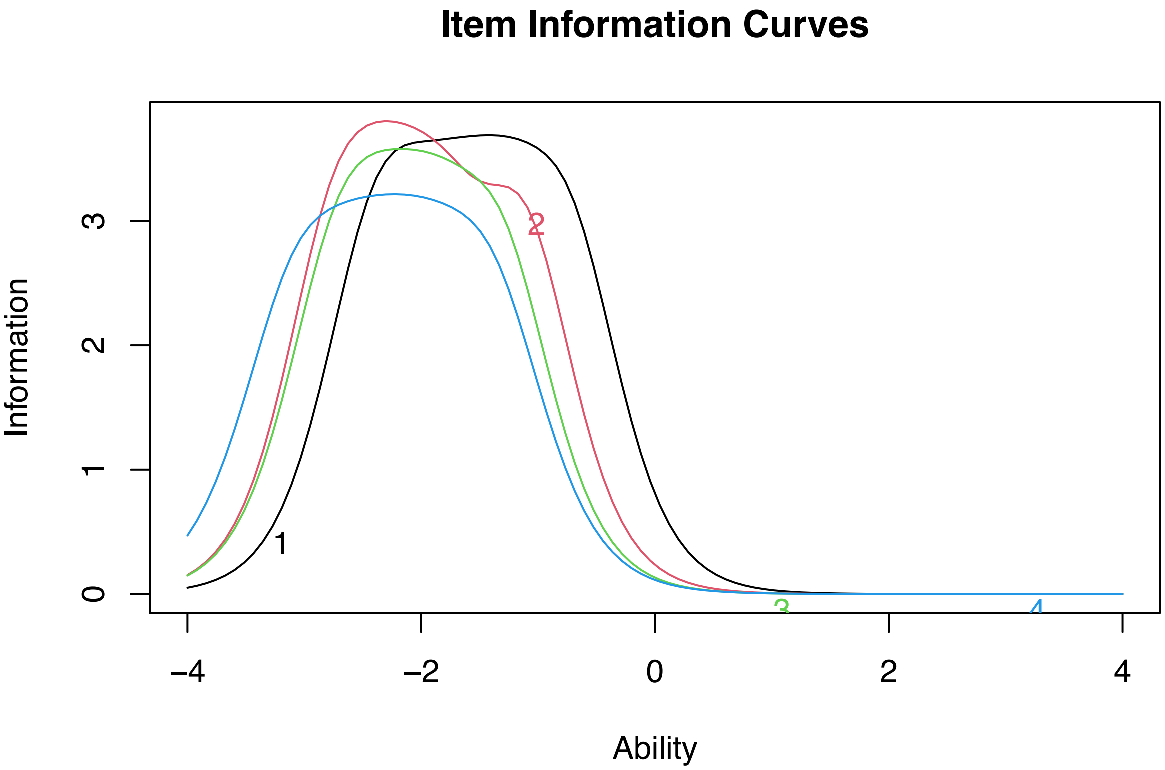

Back to top

Site 2

Reliability: Substance Use

```
## Cronbach's alpha is 0.917.
## Mean item-total correlation is 0.755.
## If each item were dropped:
##      raw_alpha std.alpha G6(smc) average_r  S/N alpha se   var.r med.r
## Q59       0.92      0.92    0.88      0.79 11.1   0.0059 0.00079  0.80
## Q4        0.88      0.89    0.84      0.72  7.9   0.0084 0.00192  0.74
## Q107      0.88      0.90    0.86      0.74  8.7   0.0082 0.00429  0.75
## Q35       0.90      0.91    0.87      0.77  9.8   0.0070 0.00100  0.75
```

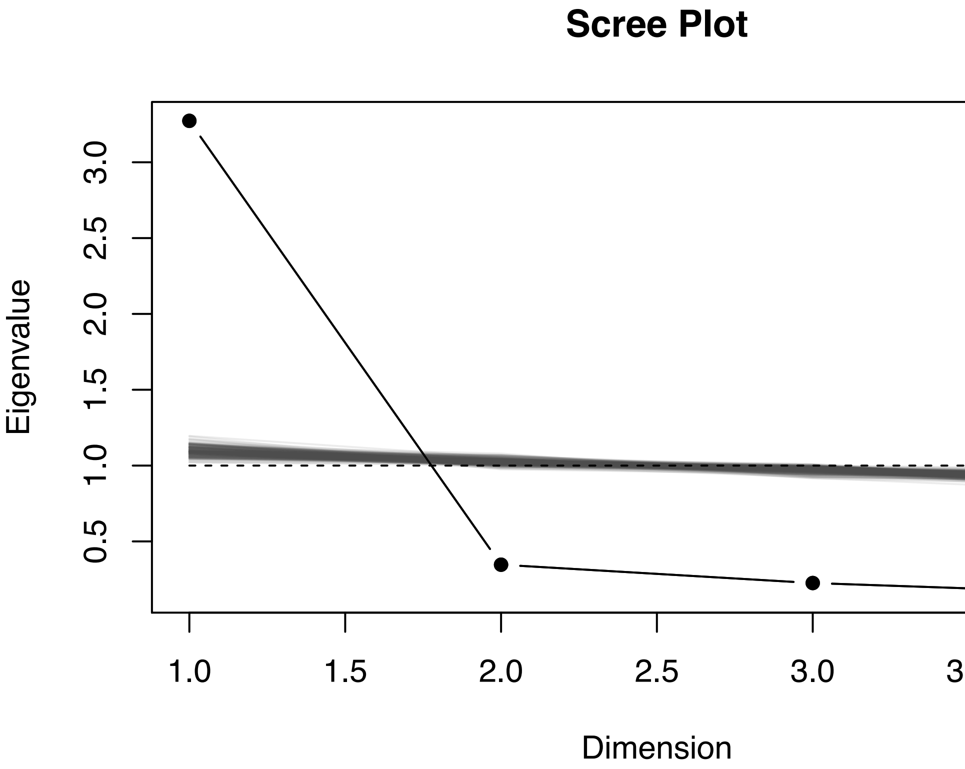

Unidimensionality: Substance Use

```
## [1] "Ratio of first to second eigenvalues: 9.448"

## [1] 3.2729631 0.3464255 0.2256145 0.1549969

## Factor Analysis using method =  minres
## Call: fa(r = grm_obj$X)
## Standardized loadings (pattern matrix) based upon correlation matrix
##       MR1   h2   u2 com
## Q59  0.81 0.65 0.35   1
## Q4   0.93 0.87 0.13   1
## Q107 0.89 0.79 0.21   1
## Q35  0.85 0.73 0.27   1
##
##                 MR1
## SS loadings    3.04
## Proportion Var 0.76
##
## Mean item complexity =  1
## Test of the hypothesis that 1 factor is sufficient.
##
## The degrees of freedom for the null model are  6  and the objective function was  3.23 with Chi Squa
## The degrees of freedom for the model are 2  and the objective function was  0.05
##
## The root mean square of the residuals (RMSR) is  0.02
## The df corrected root mean square of the residuals is  0.04
##
## The harmonic number of observations is  520 with the empirical chi square  2.82  with prob <  0.24
## The total number of observations was  596  with Likelihood Chi Square =  28.56  with prob <  6.3e-07
##
## Tucker Lewis Index of factoring reliability =  0.958
## RMSEA index =  0.149  and the 90 % confidence intervals are  0.104 0.2
```

```
## BIC =  15.78
## Fit based upon off diagonal values = 1
## Measures of factor score adequacy
##                                                   MR1
## Correlation of (regression) scores with factors   0.97
## Multiple R square of scores with factors          0.94
## Minimum correlation of possible factor scores     0.88
```

Graded-Response Model: Substance Use

```
##      Extrmt1 Extrmt2 Extrmt3 Extrmt4 Extrmt5 Extrmt6 Dscrmn
## Q59   -0.737  -1.057  -1.291  -1.581  -1.845  -2.047 -2.745
## Q4    -1.153  -1.454  -1.638  -1.969  -2.178  -2.579 -3.523
## Q107  -1.000  -1.370  -1.620  -1.870  -2.091  -2.313 -3.112
## Q35   -1.157  -1.545  -1.766  -2.062  -2.223  -2.387 -3.478
```

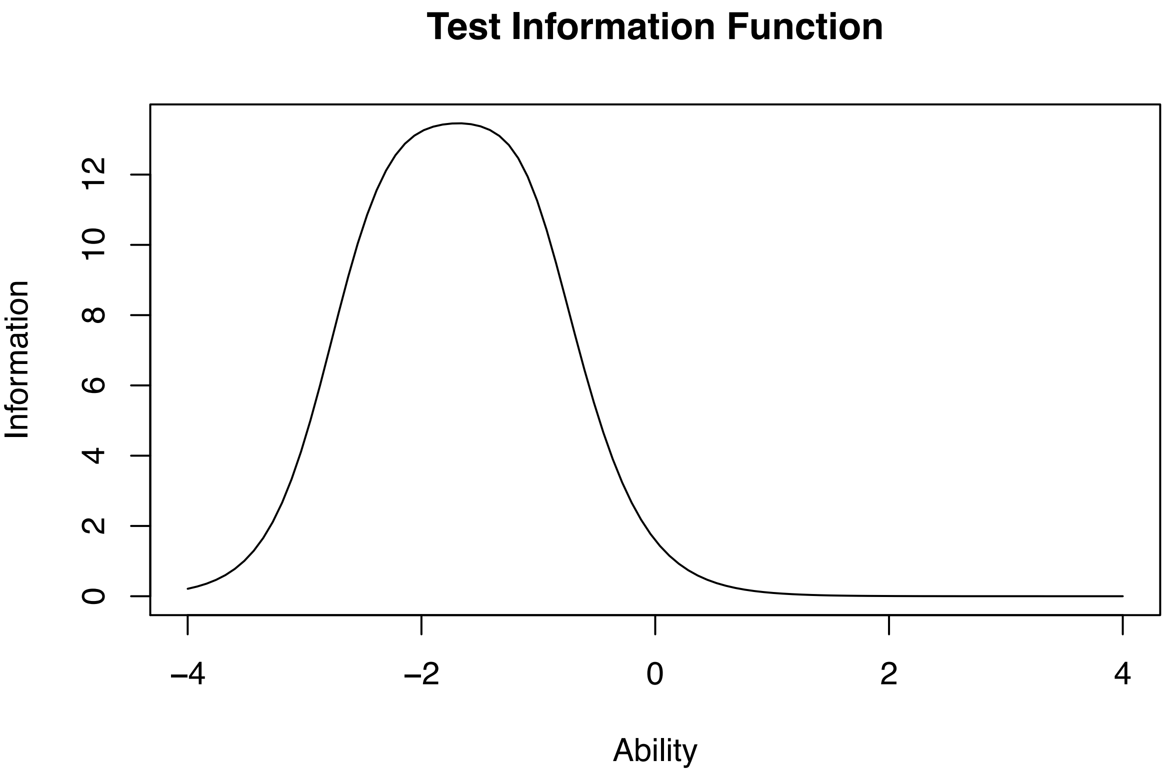

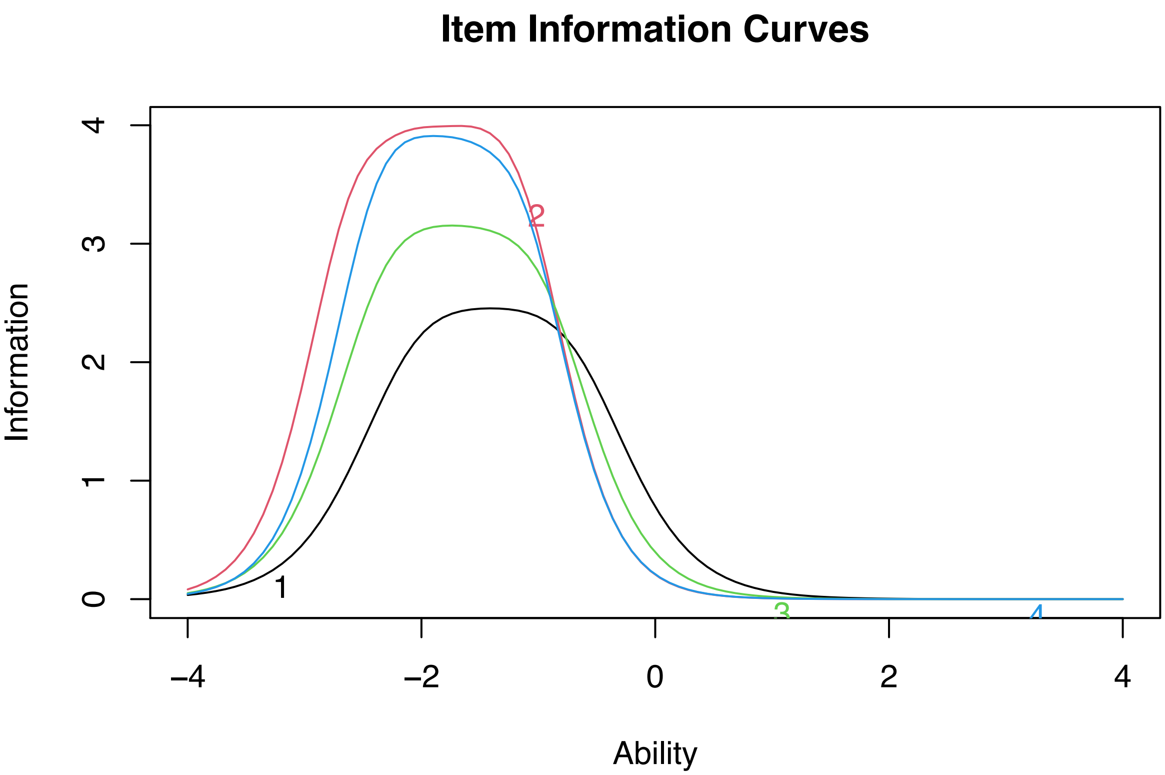

Back to top

Site DIF

```
## Call:
## lordif::lordif(resp.data = as.data.frame(merged_data), group = site)
##
##   Number of DIF groups: 2
##
##   Number of items flagged for DIF: 1 of 4
##
##   Items flagged: 3
##
##   Number of iterations for purification: 2 of 10
##
##   Detection criterion: Chisqr
##
##   Threshold: alpha = 0.01
##
##   item ncat  chi12  chi13  chi23
## 1    1    7 0.8874 0.7627 0.4701
## 2    2    6 0.4715 0.5554 0.4174
## 3    3    6 0.0004 0.0018 0.5952
## 4    4    6 0.4924 0.7698 0.8195
```

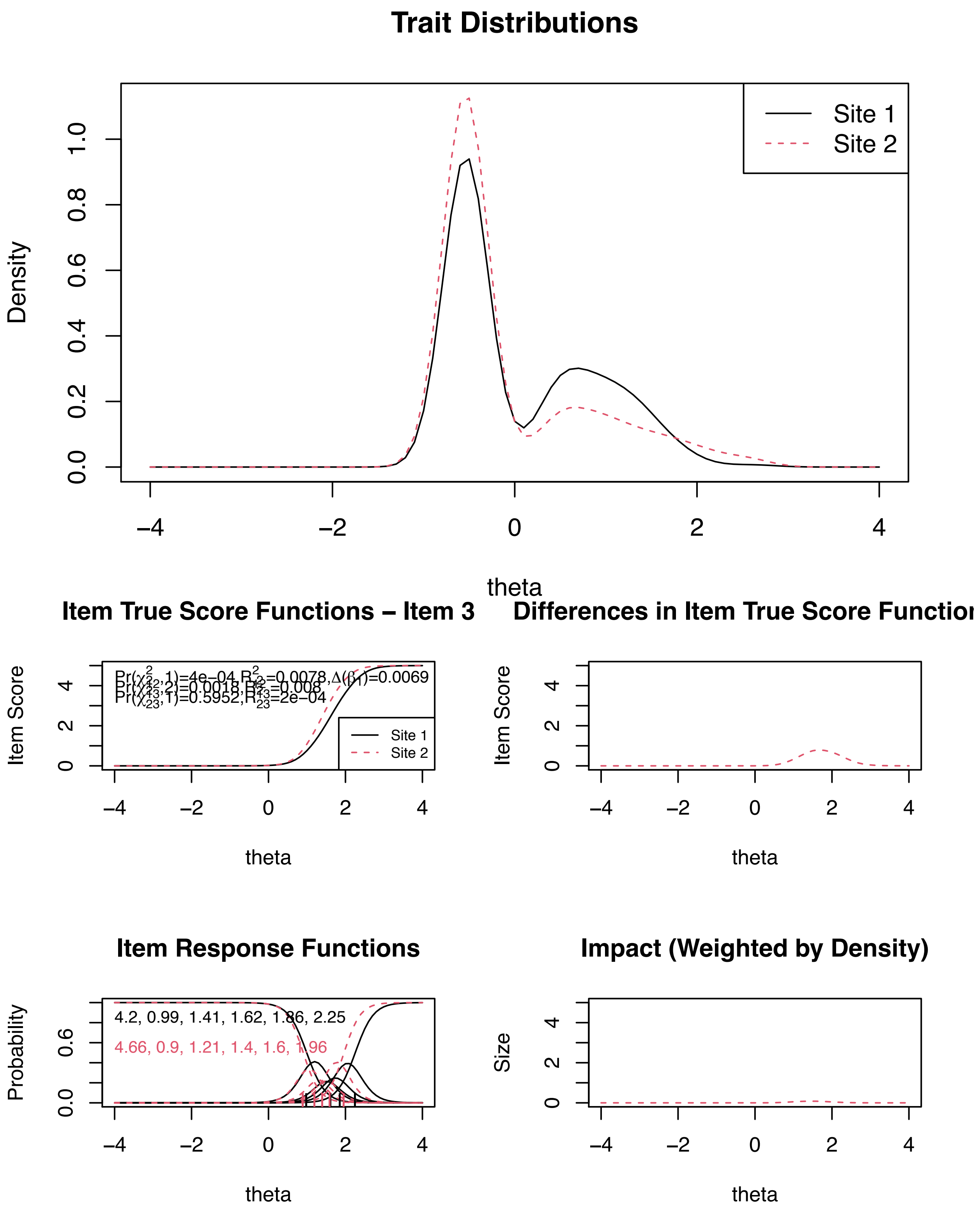
Trait Distributions
Density
theta
Site 1
Site 2
Item True Score Functions – Item 3
Item Score
Site 1
Site 2
Differences in Item True Score Functio
Item Score
theta
Item Response Functions
Probability
4.2, 0.99, 1.41, 1.62, 1.86, 2.25
4.66, 0.9, 1.21, 1.4, 1.6, 1.96
Impact (Weighted by Density)
Size

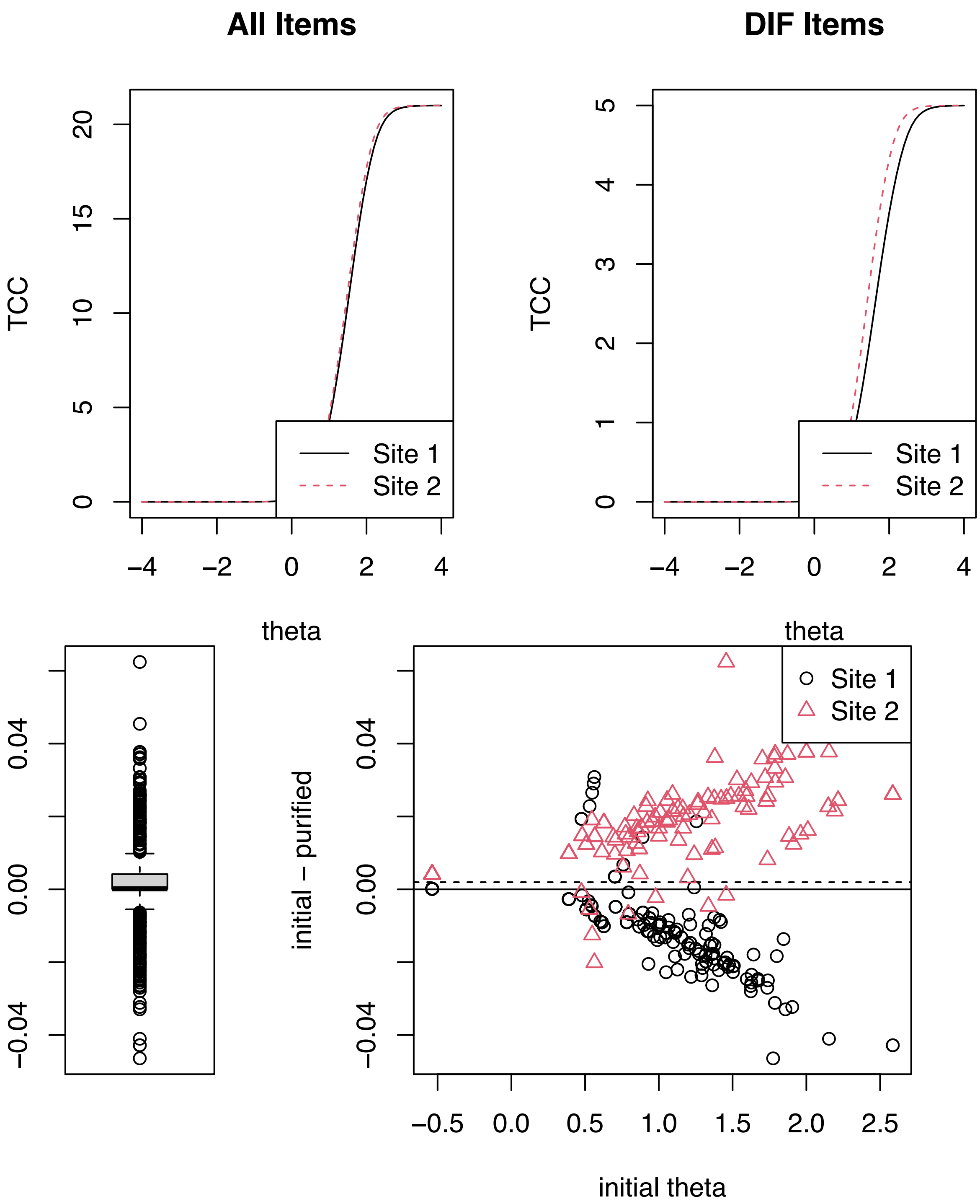

Back to top

Gender-based DIF: Substance Use

```
## Call:
## lordif::lordif(resp.data = as.data.frame(sex.data), group = sex)
##
##   Number of DIF groups: 2
```

```
##
##   Number of items flagged for DIF: 0 of 4
##
##   Items flagged:
##
##   Number of iterations for purification: 1 of 10
##
##   Detection criterion: Chisqr
##
##   Threshold: alpha = 0.01
```

Age-based DIF: Substance Use

```
## Call:
## lordif::lordif(resp.data = as.data.frame(age.data), group = age)
##
##   Number of DIF groups: 2
##
##   Number of items flagged for DIF: 1 of 4
##
##   Items flagged: 4
##
##   Number of iterations for purification: 2 of 10
##
##   Detection criterion: Chisqr
##
##   Threshold: alpha = 0.01
##
##   item ncat  chi12  chi13  chi23
## 1    1    7 0.4450 0.7122 0.7571
## 2    2    7 0.5039 0.2775 0.1457
## 3    3    6 0.5665 0.4831 0.2885
## 4    4    7 0.0003 0.0005 0.1325
```

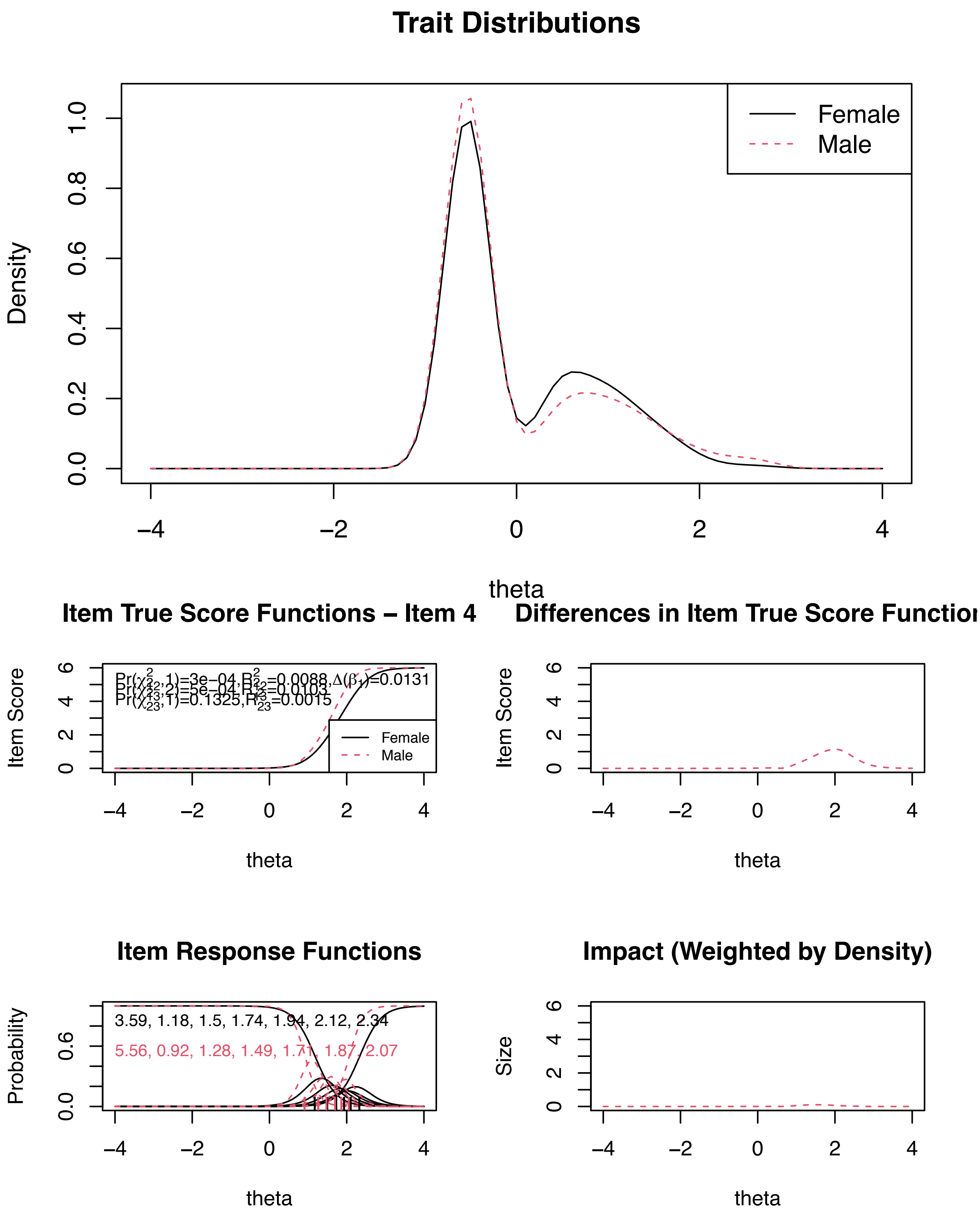
Trait Distributions
Density
theta
Female
Male
Item True Score Functions – Item 4
Item Score
Female
Male
Differences in Item True Score Functior
Item Score
theta
Item Response Functions
Probability
3.59, 1.18, 1.5, 1.74, 1.94, 2.12, 2.34
5.56, 0.92, 1.28, 1.49, 1.71, 1.87, 2.07
theta
Impact (Weighted by Density)
Size
theta

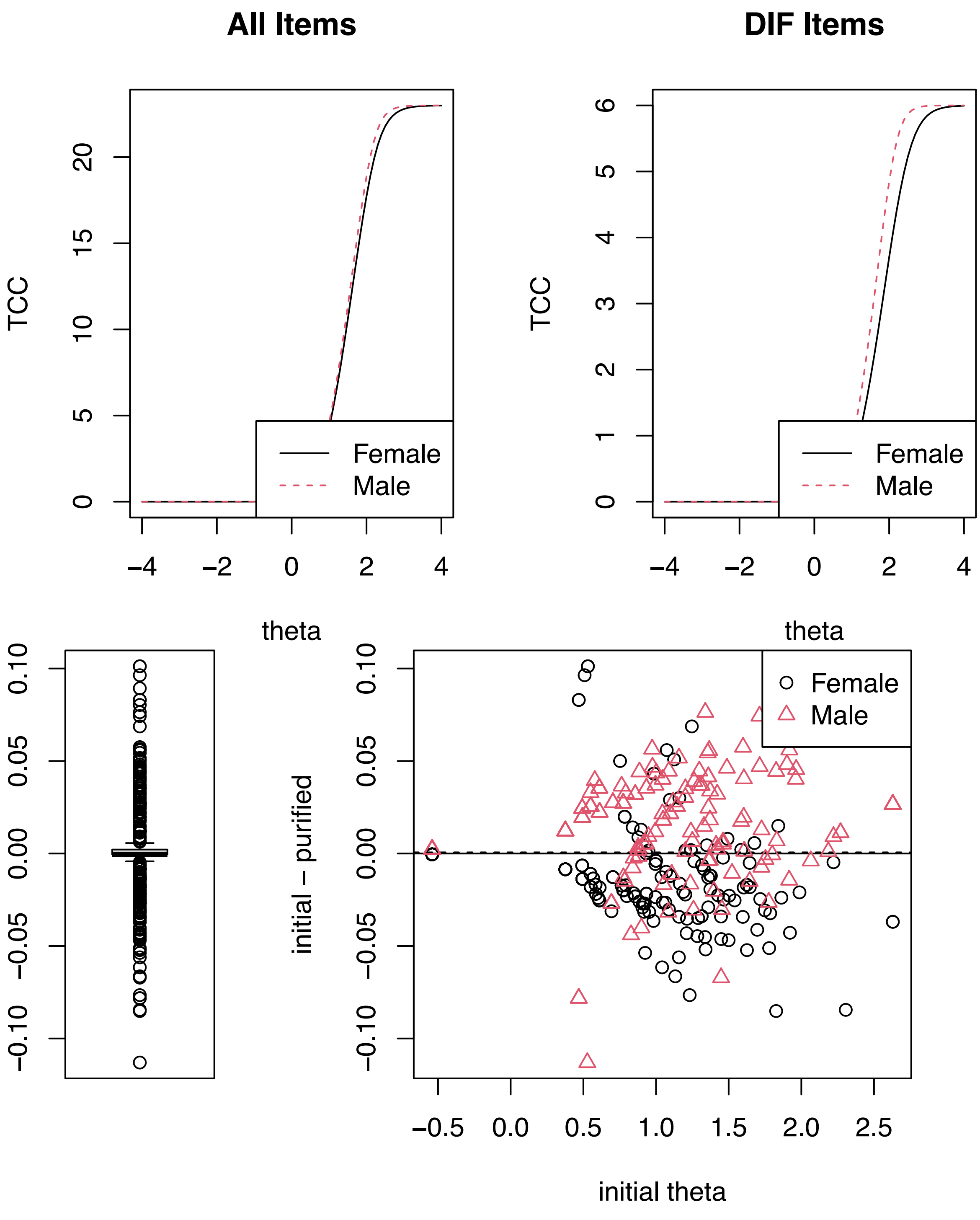

Back to top

Suicide

Site 1

Did not converge.

Site 2

Reliability: Suicide

```
## Cronbach's alpha is 0.823.
## Mean item-total correlation is 0.575.
## If each item were dropped:
##      raw_alpha std.alpha G6(smc) average_r S/N alpha se   var.r med.r
## Q19       0.76      0.80    0.72      0.57 3.9    0.015 4.3e-04  0.56
## Q105      0.76      0.80    0.73      0.57 4.0    0.015 2.5e-04  0.57
## Q77       0.77      0.81    0.74      0.59 4.3    0.014 2.7e-05  0.59
## Q106      0.80      0.80    0.73      0.57 4.1    0.014 3.5e-04  0.57
```

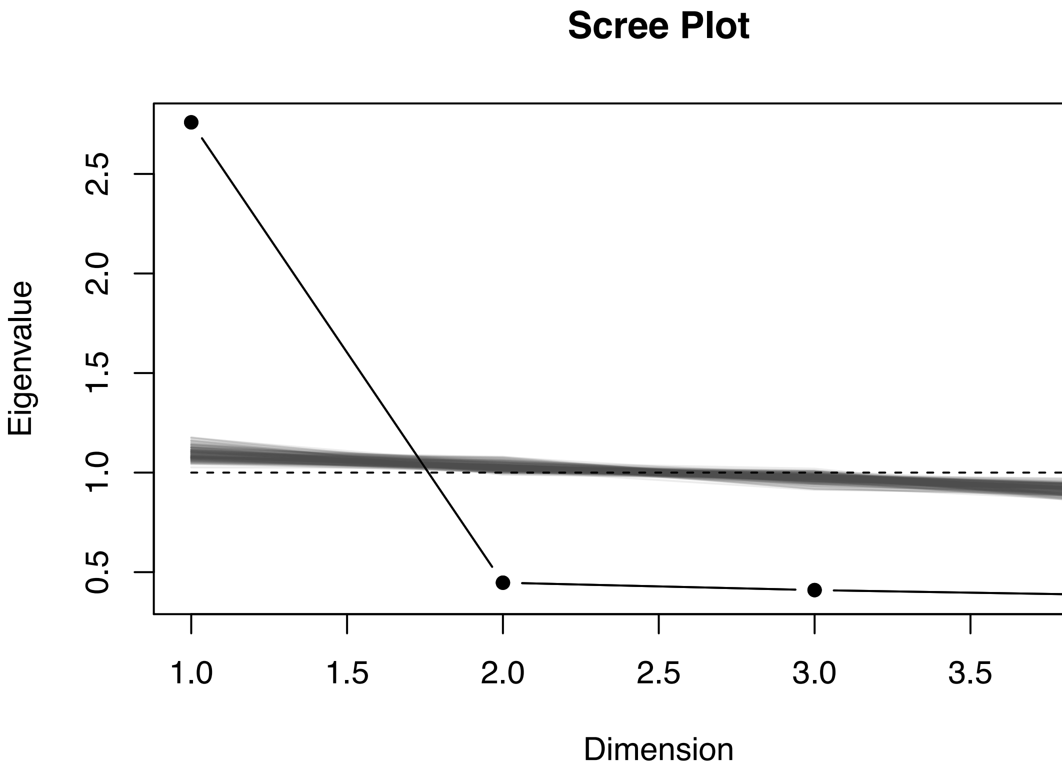

Unidimensionality: Suicide

```
## [1] "Ratio of first to second eigenvalues: 6.173"

## [1] 2.7591314 0.4469352 0.4098761 0.3840574

## Factor Analysis using method =  minres
## Call: fa(r = grm_obj$X)
## Standardized loadings (pattern matrix) based upon correlation matrix
##       MR1   h2   u2 com
## Q19  0.79 0.62 0.38   1
## Q105 0.77 0.60 0.40   1
## Q77  0.74 0.55 0.45   1
## Q106 0.76 0.58 0.42   1
##
##                 MR1
## SS loadings    2.35
## Proportion Var 0.59
##
```

```
## Mean item complexity =  1
## Test of the hypothesis that 1 factor is sufficient.
##
## The degrees of freedom for the null model are  6  and the objective function was  1.64 with Chi Squa
## The degrees of freedom for the model are 2  and the objective function was  0
##
## The root mean square of the residuals (RMSR) is  0.01
## The df corrected root mean square of the residuals is  0.01
##
## The harmonic number of observations is  567 with the empirical chi square  0.36  with prob <  0.84
## The total number of observations was  596  with Likelihood Chi Square =  1.11  with prob <  0.57
##
## Tucker Lewis Index of factoring reliability =  1.003
## RMSEA index =  0  and the 90 % confidence intervals are  0 0.068
## BIC =  -11.67
## Fit based upon off diagonal values = 1
## Measures of factor score adequacy
##                                                    MR1
## Correlation of (regression) scores with factors   0.92
## Multiple R square of scores with factors          0.85
## Minimum correlation of possible factor scores     0.70
```

Graded-Response Model: Suicide

```
##      Extrmt1 Extrmt2 Extrmt3 Extrmt4 Extrmt5 Extrmt6 Dscrmn
## Q19   -0.801  -1.335  -1.747  -2.089  -2.401  -2.620 -2.500
## Q105  -1.602  -1.753  -1.805  -2.049  -2.169  -2.266 -3.606
## Q77   -1.442  -1.859  -1.907  -2.128  -2.413  -2.682 -3.375
## Q106  -2.015  -2.317  -2.601  -2.983  -3.275  -3.581 -2.090
```

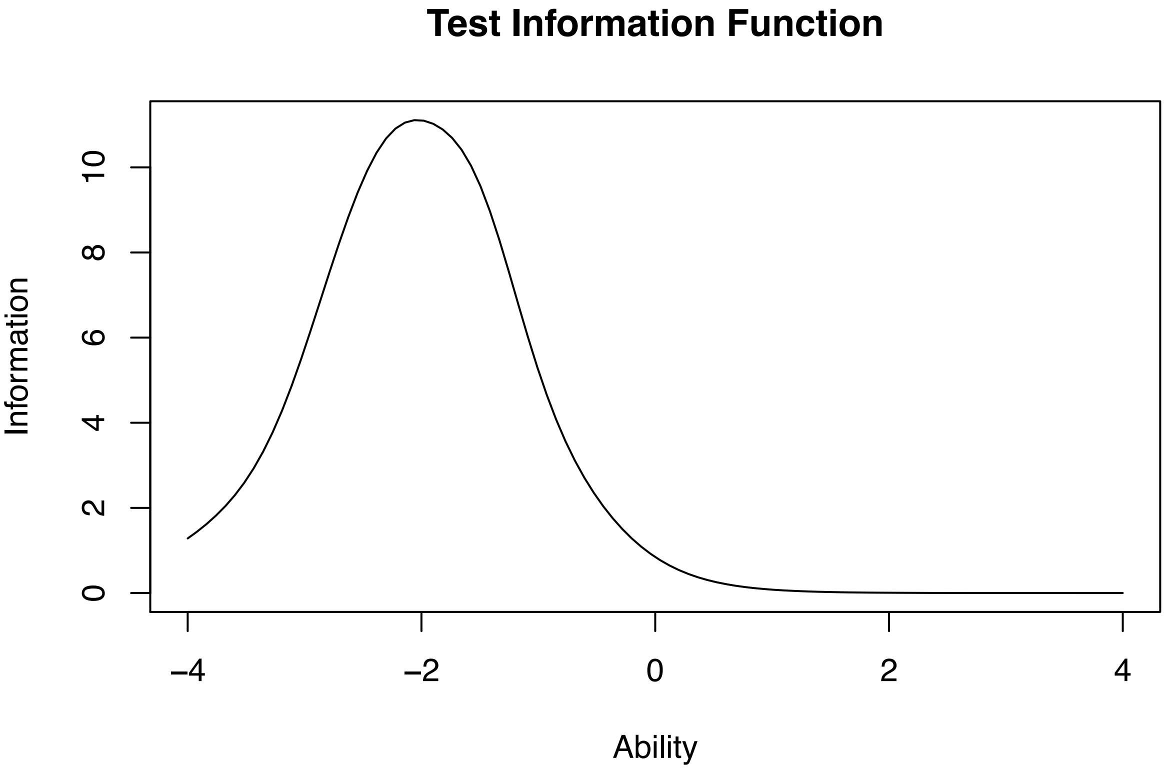

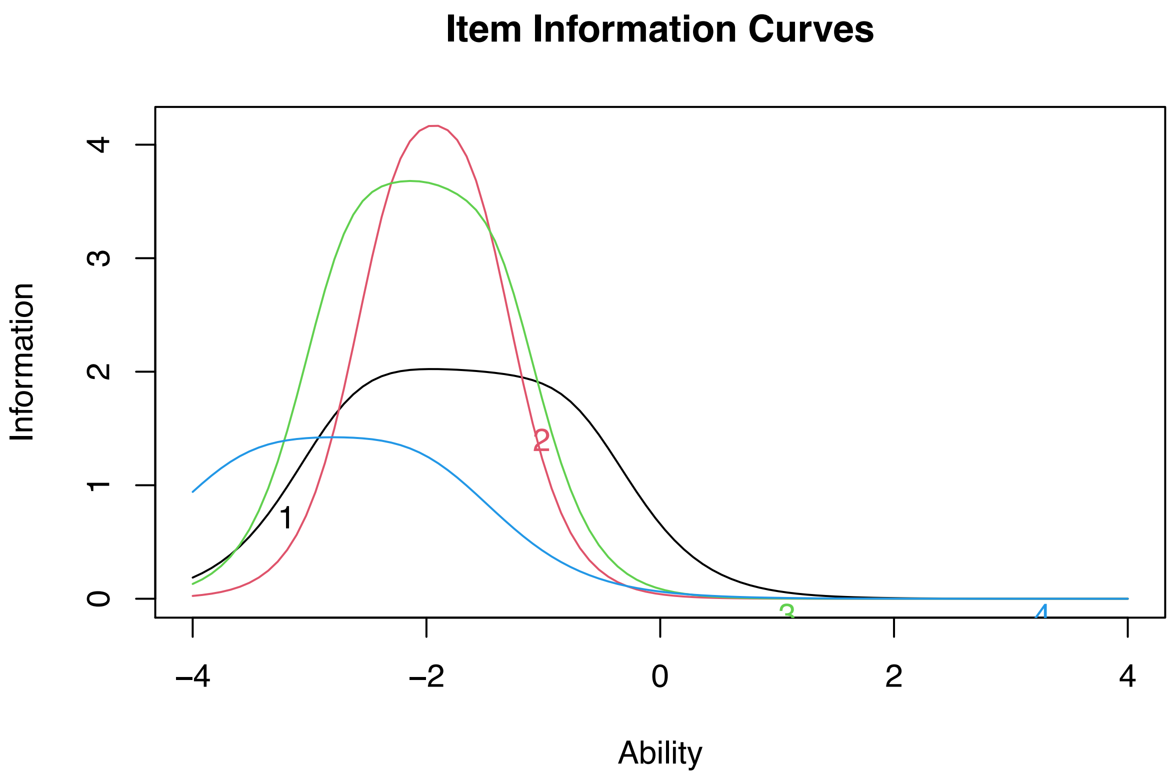

Back to top

Gender-based DIF: Suicide

```
## Call:
## lordif::lordif(resp.data = as.data.frame(sex.data), group = sex)
##
##   Number of DIF groups: 2
##
##   Number of items flagged for DIF: 1 of 4
##
##   Items flagged: 2
##
##   Number of iterations for purification: 2 of 10
##
##   Detection criterion: Chisqr
##
##   Threshold: alpha = 0.01
##
##   item ncat  chi12  chi13  chi23
## 1    1    7 0.5858 0.4369 0.2437
## 2    2    7 0.0052 0.0000 0.0001
## 3    3    7 0.8737 0.5515 0.2805
## 4    4    5 0.4501 0.1497 0.0724
```

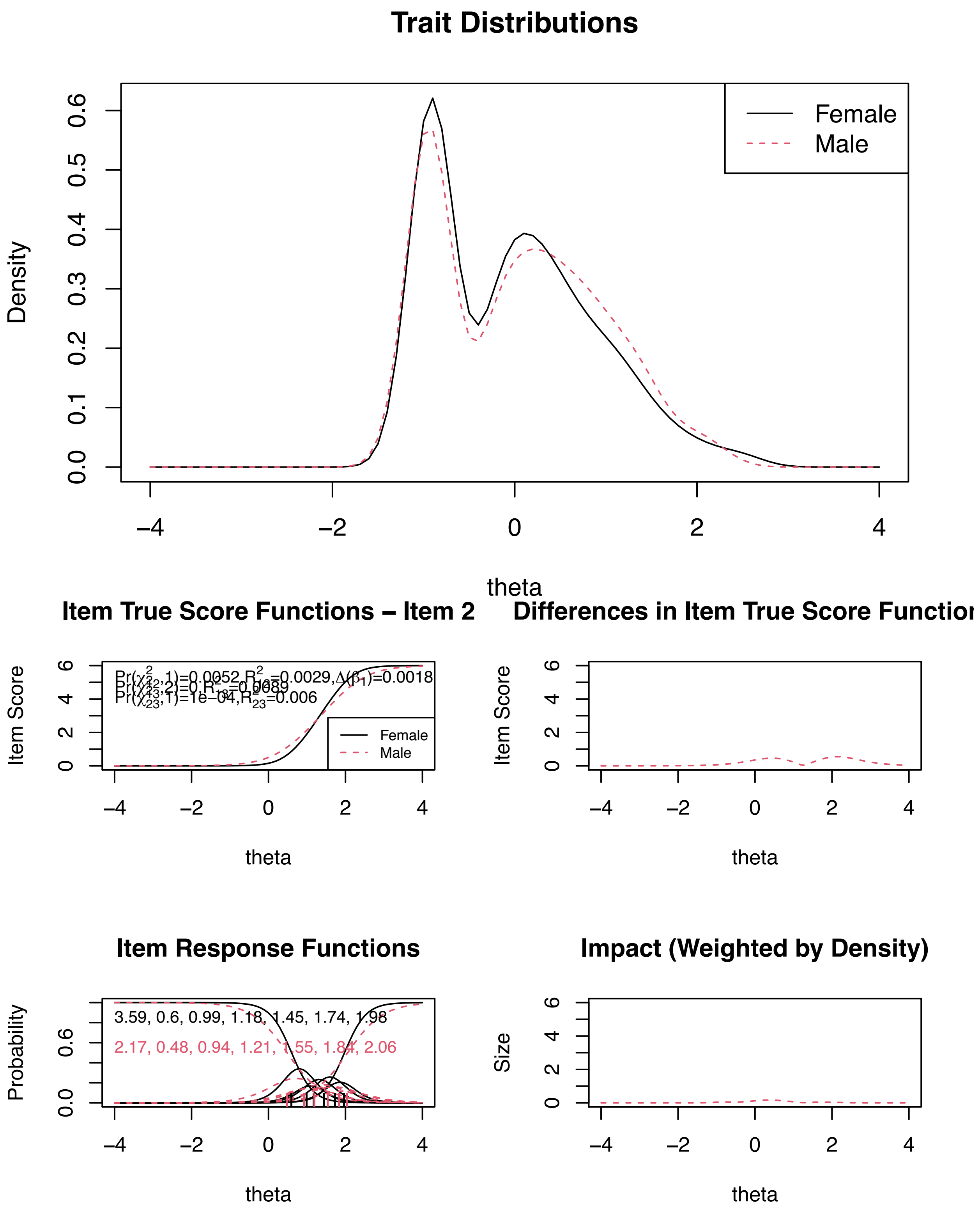
Trait Distributions
Density
Female
Male
theta
Item True Score Functions – Item 2
Differences in Item True Score Functio
Item Score
Female
Male
Item Response Functions
Probability
3.59, 0.6, 0.99, 1.18, 1.45, 1.74, 1.98
2.17, 0.48, 0.94, 1.21, 1.55, 1.84, 2.06
Impact (Weighted by Density)
Size

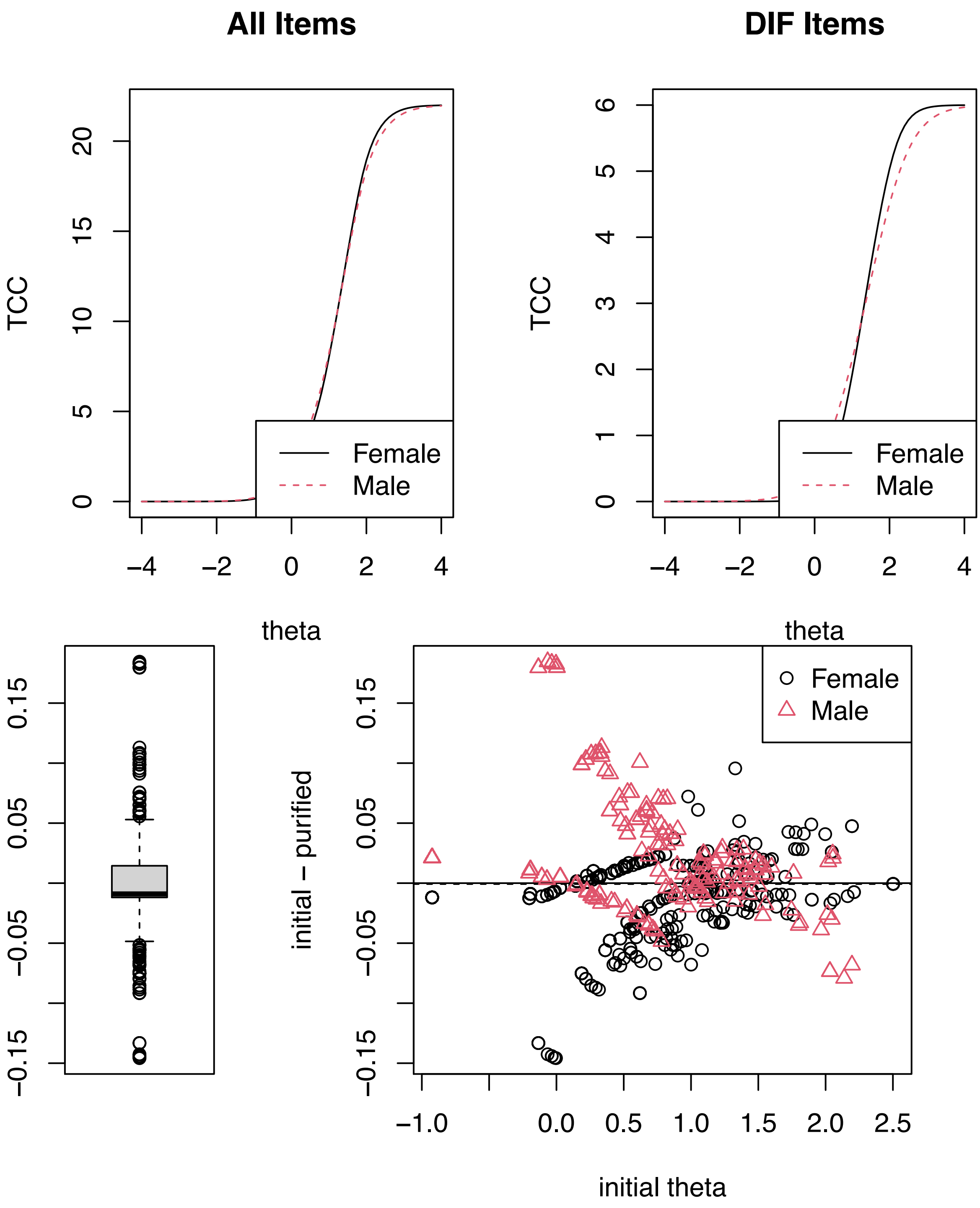

Age-based DIF: Suicide

```
## Call:
## lordif::lordif(resp.data = as.data.frame(age.data), group = age)
##
##   Number of DIF groups: 2
##
```

```
##   Number of items flagged for DIF: 1 of 4
##
##   Items flagged: 4
##
##   Number of iterations for purification: 2 of 10
##
##   Detection criterion: Chisqr
##
##   Threshold: alpha = 0.01
##
##   item ncat  chi12  chi13  chi23
## 1    1    7 0.7743 0.4980 0.2520
## 2    2    7 0.1809 0.2467 0.3153
## 3    3    7 0.7417 0.9157 0.7952
## 4    4    4 0.0001 0.0001 0.0712
```

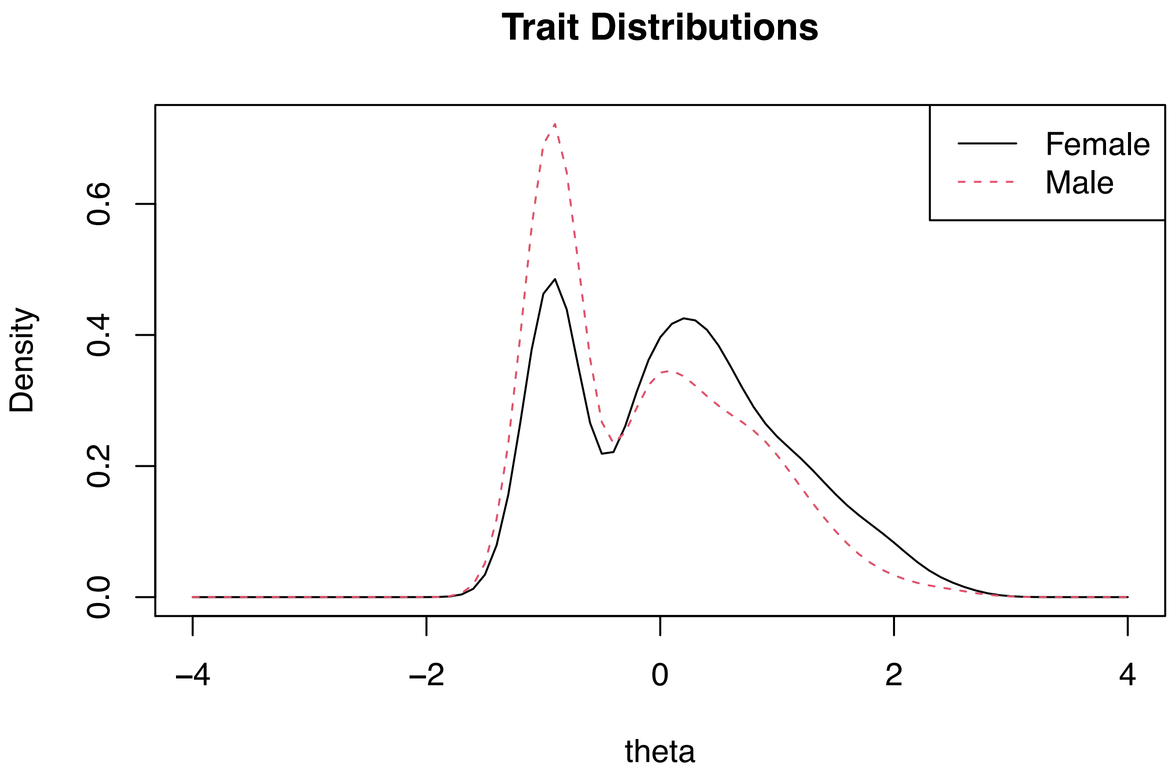

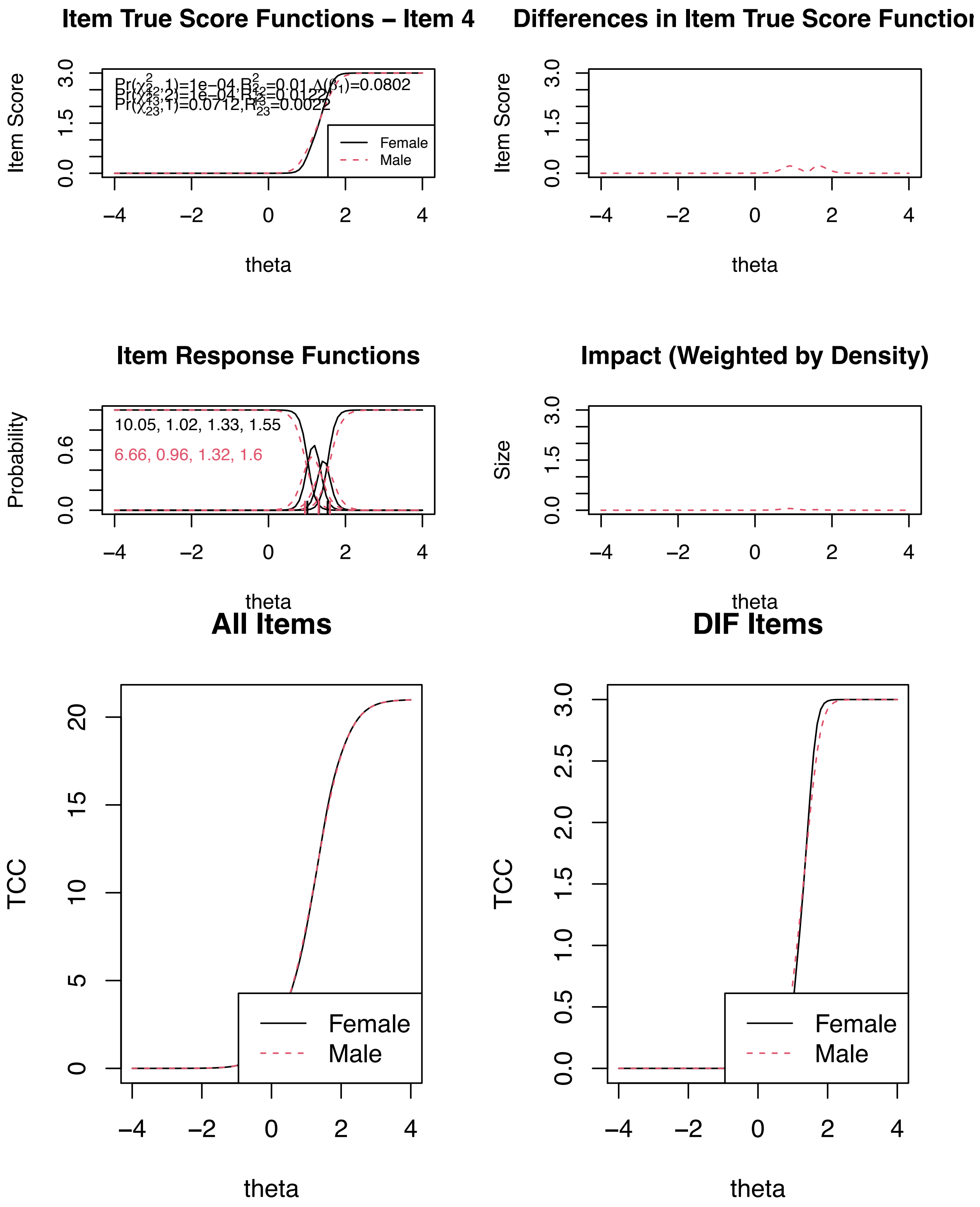
Item True Score Functions – Item 4
Differences in Item True Score Functio
Item Score
theta
Female
Male
Item Response Functions
Impact (Weighted by Density)
Probability
Size
10.05, 1.02, 1.33, 1.55
6.66, 0.96, 1.32, 1.6
All Items
DIF Items
TCC

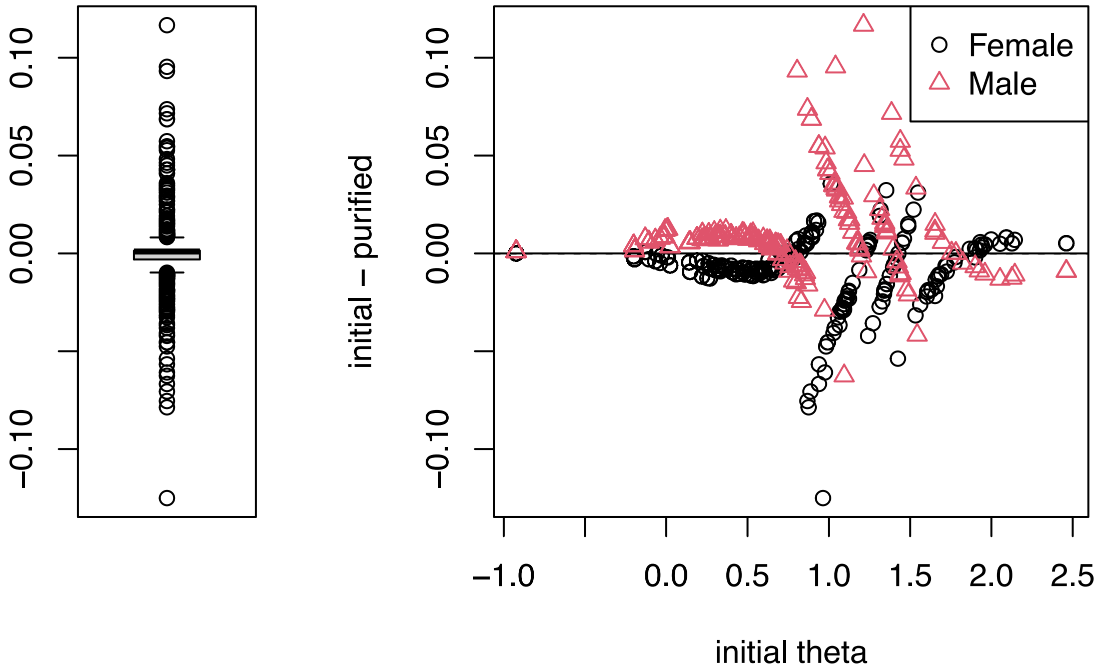

Back to top

Trauma Reaction

Site 1

Reliability: Trauma Reaction

```
## Cronbach's alpha is 0.744.
## Mean item-total correlation is 0.425.
## If each item were dropped:
##      raw_alpha std.alpha G6(smc) average_r S/N alpha se  var.r med.r
## Q111      0.59      0.59    0.50      0.33 1.5    0.029 0.0042  0.32
## Q110      0.71      0.72    0.68      0.46 2.6    0.021 0.0417  0.37
## Q113      0.77      0.77    0.72      0.53 3.4    0.016 0.0227  0.50
## Q112      0.65      0.65    0.57      0.38 1.8    0.025 0.0139  0.37
```

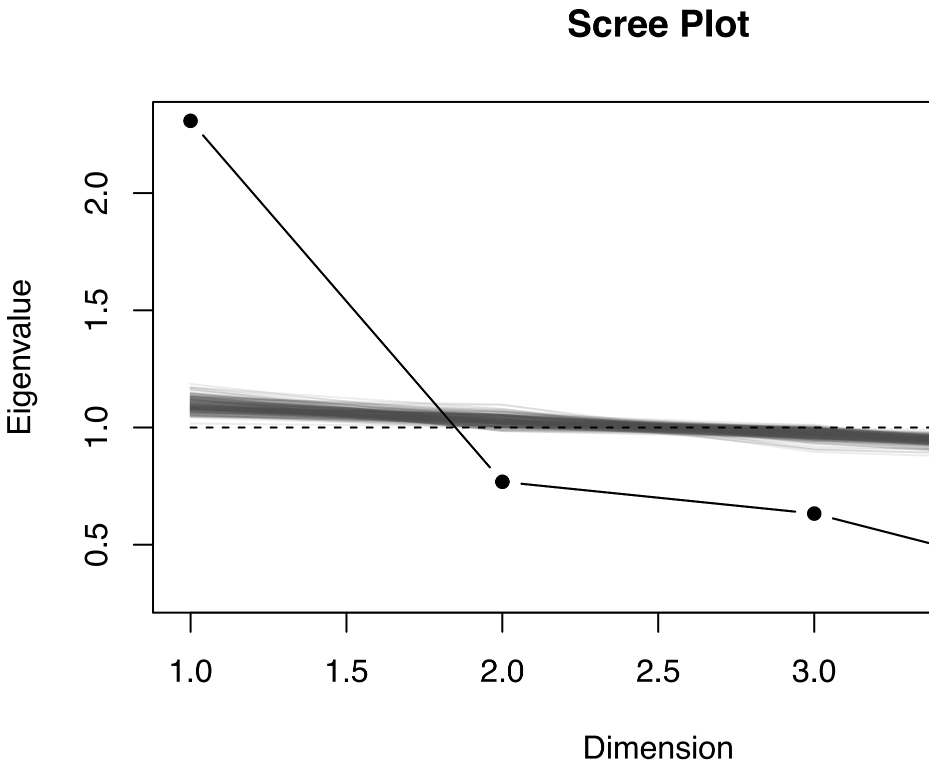

Unidimensionality: Trauma Reaction

```
## [1] "Ratio of first to second eigenvalues: 3.004"

## [1] 2.3081635 0.7682523 0.6327374 0.2908468

## Factor Analysis using method =  minres
## Call: fa(r = grm_obj$X)
## Standardized loadings (pattern matrix) based upon correlation matrix
##       MR1   h2   u2 com
## Q111 0.91 0.83 0.17   1
## Q110 0.55 0.31 0.69   1
## Q113 0.42 0.18 0.82   1
## Q112 0.75 0.56 0.44   1
##
##                 MR1
## SS loadings    1.88
## Proportion Var 0.47
##
## Mean item complexity =  1
## Test of the hypothesis that 1 factor is sufficient.
##
## The degrees of freedom for the null model are  6  and the objective function was  1.12 with Chi Squa
## The degrees of freedom for the model are 2  and the objective function was  0.01
##
## The root mean square of the residuals (RMSR) is  0.02
## The df corrected root mean square of the residuals is  0.03
##
## The harmonic number of observations is  547 with the empirical chi square  1.96  with prob <  0.38
## The total number of observations was  617  with Likelihood Chi Square =  3.16  with prob <  0.21
##
## Tucker Lewis Index of factoring reliability =  0.995
## RMSEA index =  0.031  and the 90 % confidence intervals are  0 0.091
```

```
## BIC =  -9.69
## Fit based upon off diagonal values = 1
## Measures of factor score adequacy
##                                                   MR1
## Correlation of (regression) scores with factors   0.93
## Multiple R square of scores with factors          0.87
## Minimum correlation of possible factor scores     0.75
```

Graded-Response Model: Trauma Reaction

```
##      Extrmt1 Extrmt2 Extrmt3 Extrmt4 Extrmt5 Extrmt6 Dscrmn
## Q111  -0.415   0.060   0.401   0.680   0.992   1.163  2.688
## Q110   0.284   0.541   0.712   0.968   1.209   1.462  2.038
## Q113  -1.105  -0.600  -0.256   0.169   1.007   1.512  0.982
## Q112   0.066   0.271   0.425   0.685   0.936   1.097  4.074
```

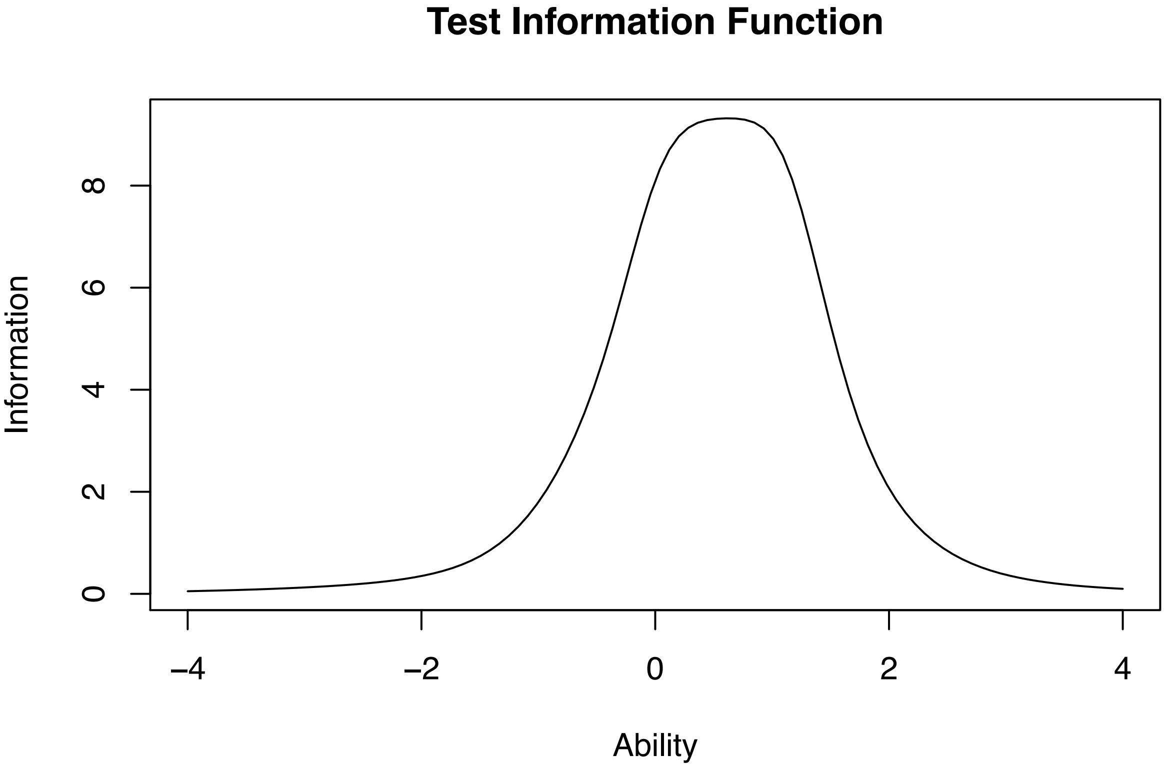

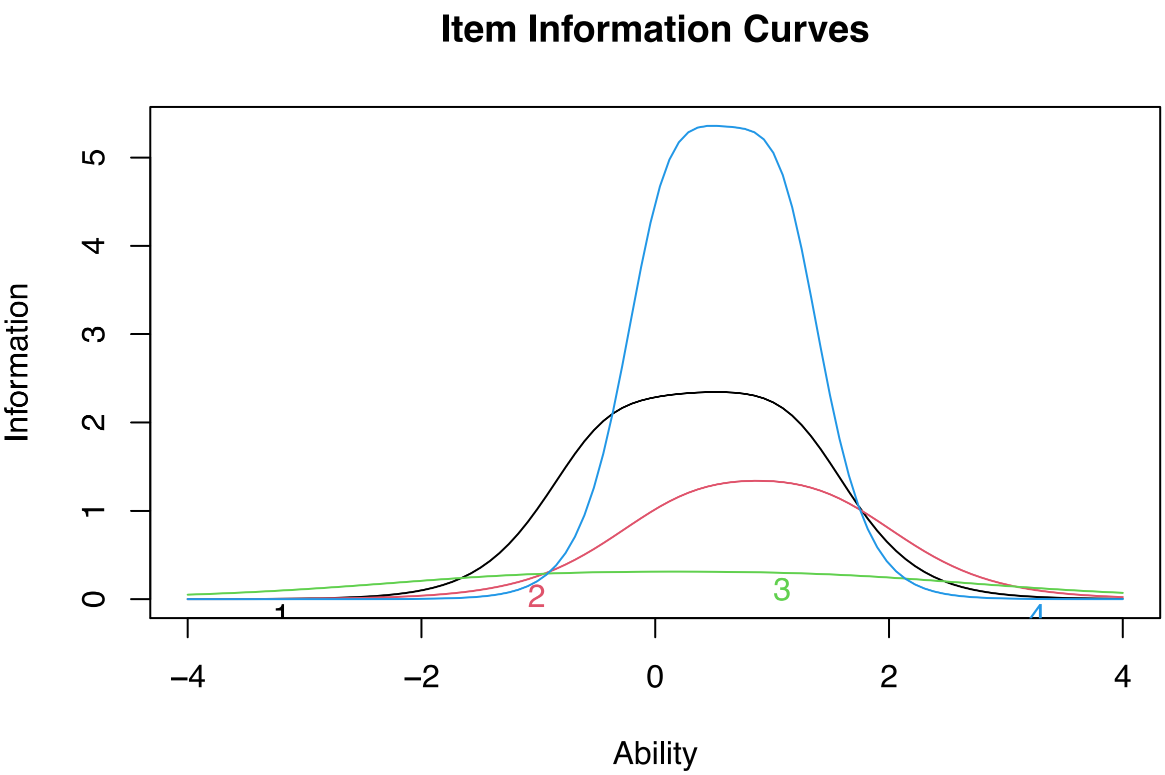

Back to top

Site 2

Reliability: Trauma Reaction

```
## Cronbach's alpha is 0.765.
## Mean item-total correlation is 0.454.
## If each item were dropped:
##      raw_alpha std.alpha G6(smc) average_r S/N alpha se  var.r med.r
## Q111      0.65      0.65    0.56      0.38 1.9    0.025 0.0025  0.37
## Q110      0.72      0.73    0.67      0.47 2.7    0.020 0.0228  0.40
## Q113      0.78      0.78    0.71      0.54 3.5    0.016 0.0104  0.53
## Q112      0.68      0.69    0.61      0.42 2.2    0.023 0.0096  0.40
```

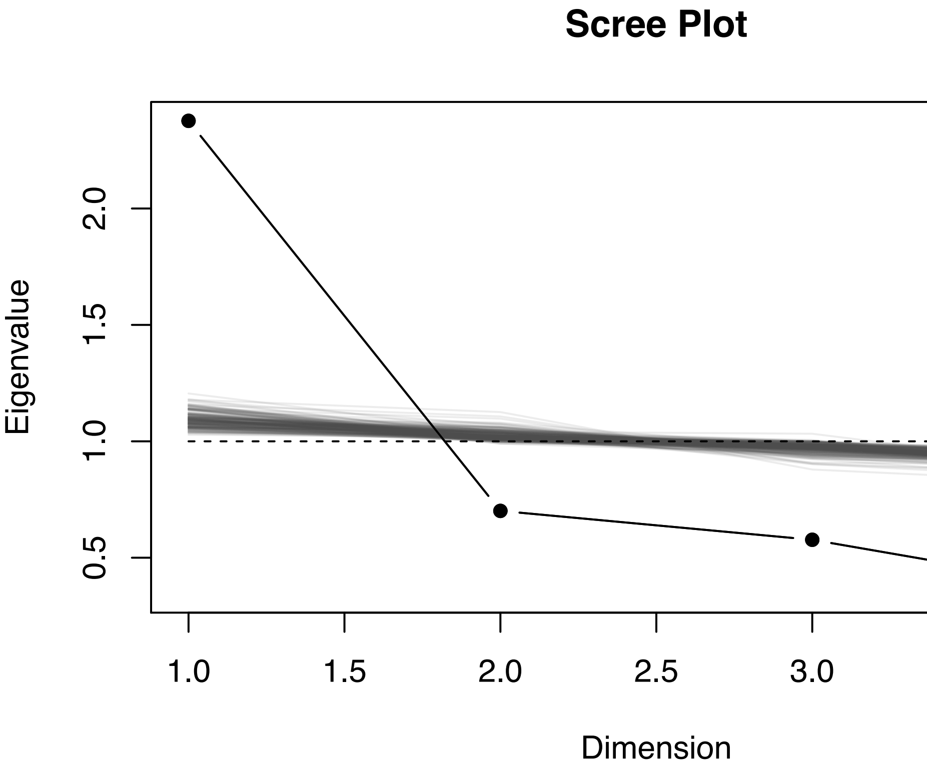

Unidimensionality: Trauma Reaction

```
## [1] "Ratio of first to second eigenvalues: 3.389"

## [1] 2.3764852 0.7011546 0.5770705 0.3452897

## Factor Analysis using method =  minres
## Call: fa(r = grm_obj$X)
## Standardized loadings (pattern matrix) based upon correlation matrix
##       MR1   h2   u2 com
## Q111 0.85 0.72 0.28   1
## Q110 0.63 0.39 0.61   1
## Q113 0.50 0.25 0.75   1
## Q112 0.73 0.54 0.46   1
##
##                 MR1
## SS loadings    1.90
## Proportion Var 0.48
##
## Mean item complexity =  1
## Test of the hypothesis that 1 factor is sufficient.
##
## The degrees of freedom for the null model are  6  and the objective function was  1.1 with Chi Square
## The degrees of freedom for the model are 2  and the objective function was  0.01
##
## The root mean square of the residuals (RMSR) is  0.02
## The df corrected root mean square of the residuals is  0.03
##
## The harmonic number of observations is  523 with the empirical chi square  2.36  with prob <  0.31
## The total number of observations was  596  with Likelihood Chi Square =  4.66  with prob <  0.097
##
## Tucker Lewis Index of factoring reliability =  0.988
## RMSEA index =  0.047  and the 90 % confidence intervals are  0 0.105
```

```
## BIC =  -8.12
## Fit based upon off diagonal values = 1
## Measures of factor score adequacy
##                                                    MR1
## Correlation of (regression) scores with factors   0.91
## Multiple R square of scores with factors          0.82
## Minimum correlation of possible factor scores     0.65
```

Graded-Response Model: Trauma Reaction

```
##      Extrmt1 Extrmt2 Extrmt3 Extrmt4 Extrmt5 Extrmt6 Dscrmn
## Q111  -0.398   0.096   0.479   0.822   1.209   1.571  3.163
## Q110  -0.053   0.464   0.895   1.200   1.629   2.074  1.660
## Q113  -1.071  -0.434   0.089   0.527   1.173   1.971  1.053
## Q112  -0.185   0.252   0.617   0.999   1.277   1.668  2.444
```

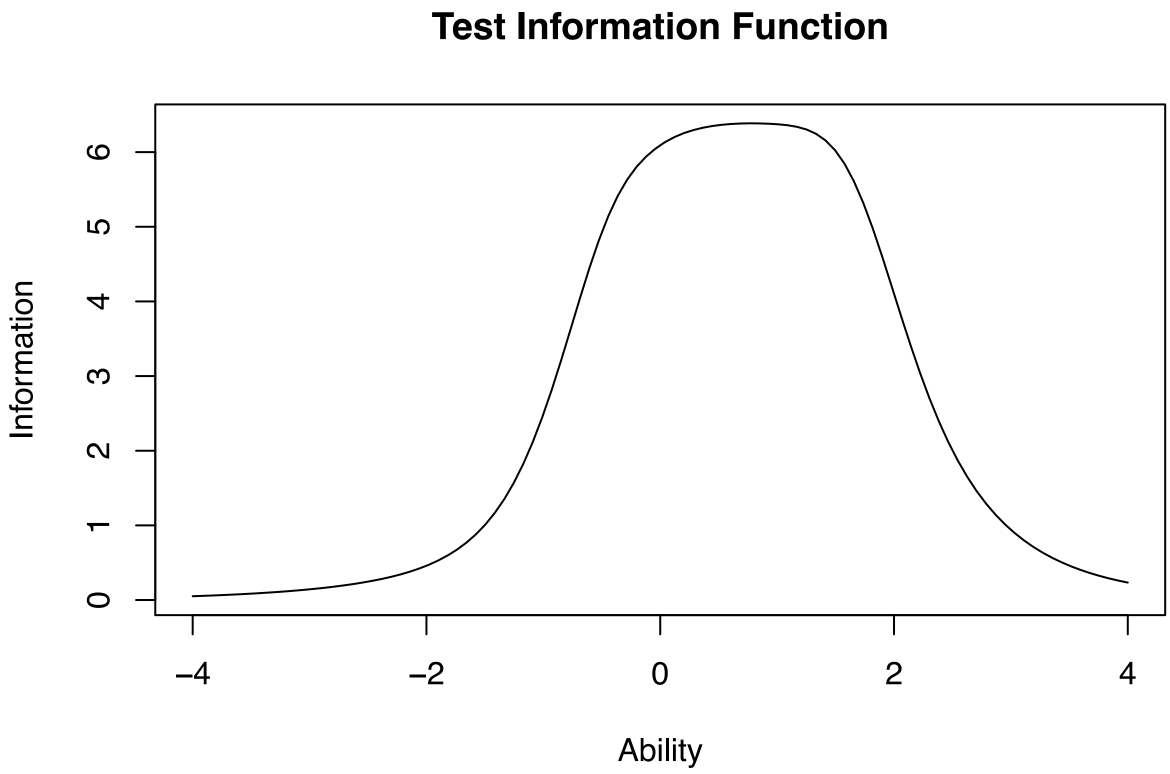

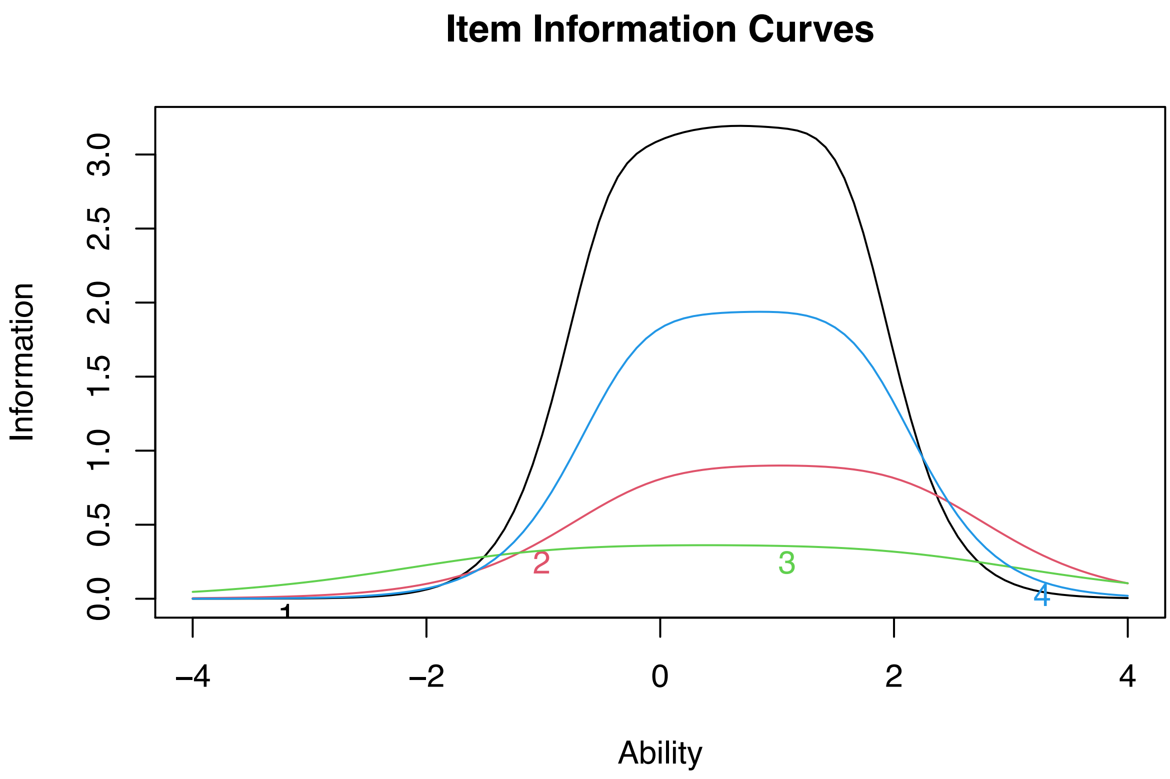

Back to top

Site DIF

```
## Call:
## lordif::lordif(resp.data = as.data.frame(merged_data), group = site)
##
##   Number of DIF groups: 2
##
##   Number of items flagged for DIF: 1 of 4
##
##   Items flagged: 4
##
##   Number of iterations for purification: 2 of 10
##
##   Detection criterion: Chisqr
##
##   Threshold: alpha = 0.01
##
##   item ncat  chi12  chi13  chi23
## 1    1    7 0.7018 0.9287 0.9720
## 2    2    7 0.2885 0.5013 0.6140
## 3    3    7 0.0312 0.0718 0.4292
## 4    4    7 0.0007 0.0022 0.3627
```

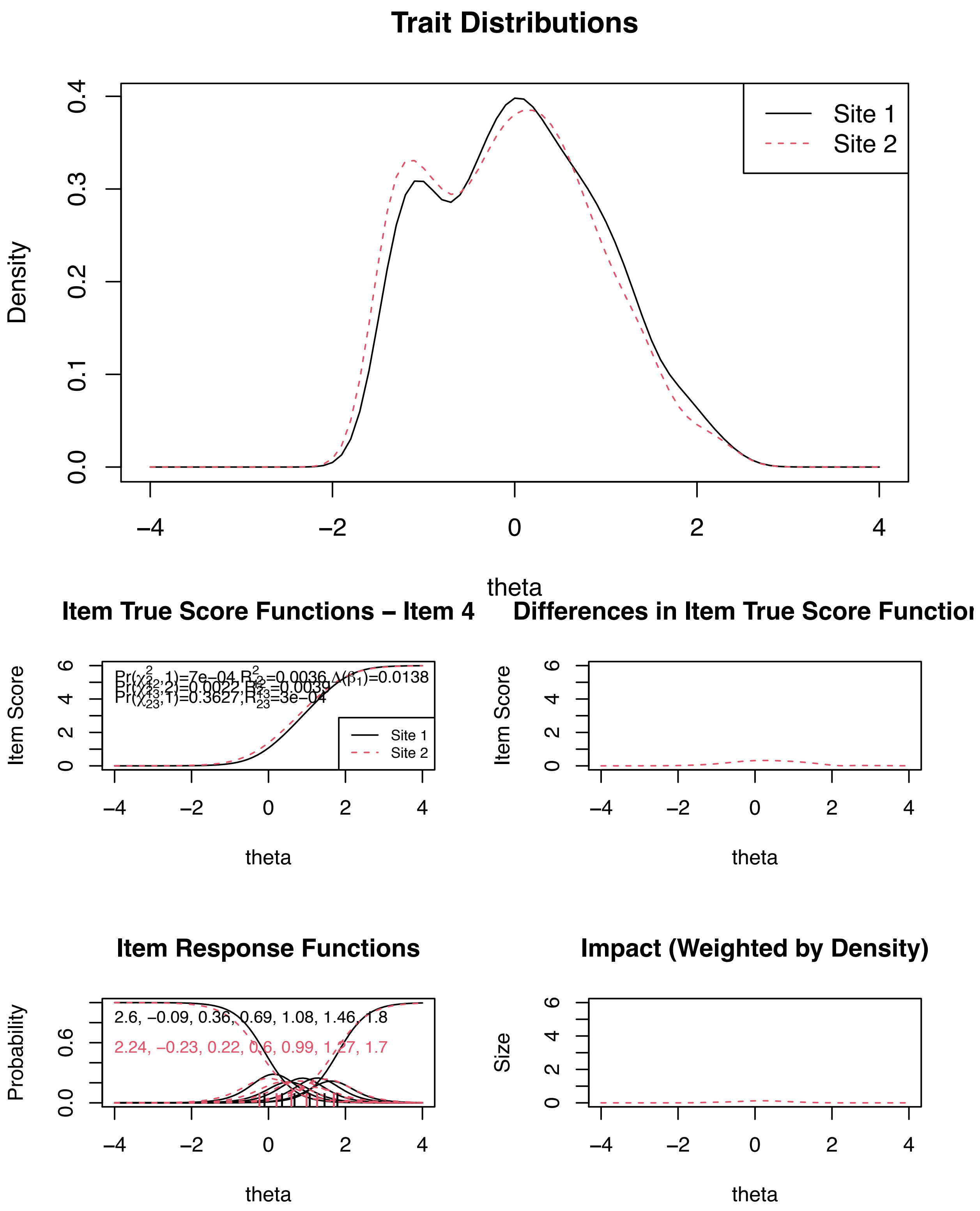
Trait Distributions
Density
theta
Site 1
Site 2
Item True Score Functions – Item 4
Item Score
Differences in Item True Score Functio
Item Response Functions
Probability
2.6, −0.09, 0.36, 0.69, 1.08, 1.46, 1.8
2.24, −0.23, 0.22, 0.6, 0.99, 1.27, 1.7
Impact (Weighted by Density)
Size

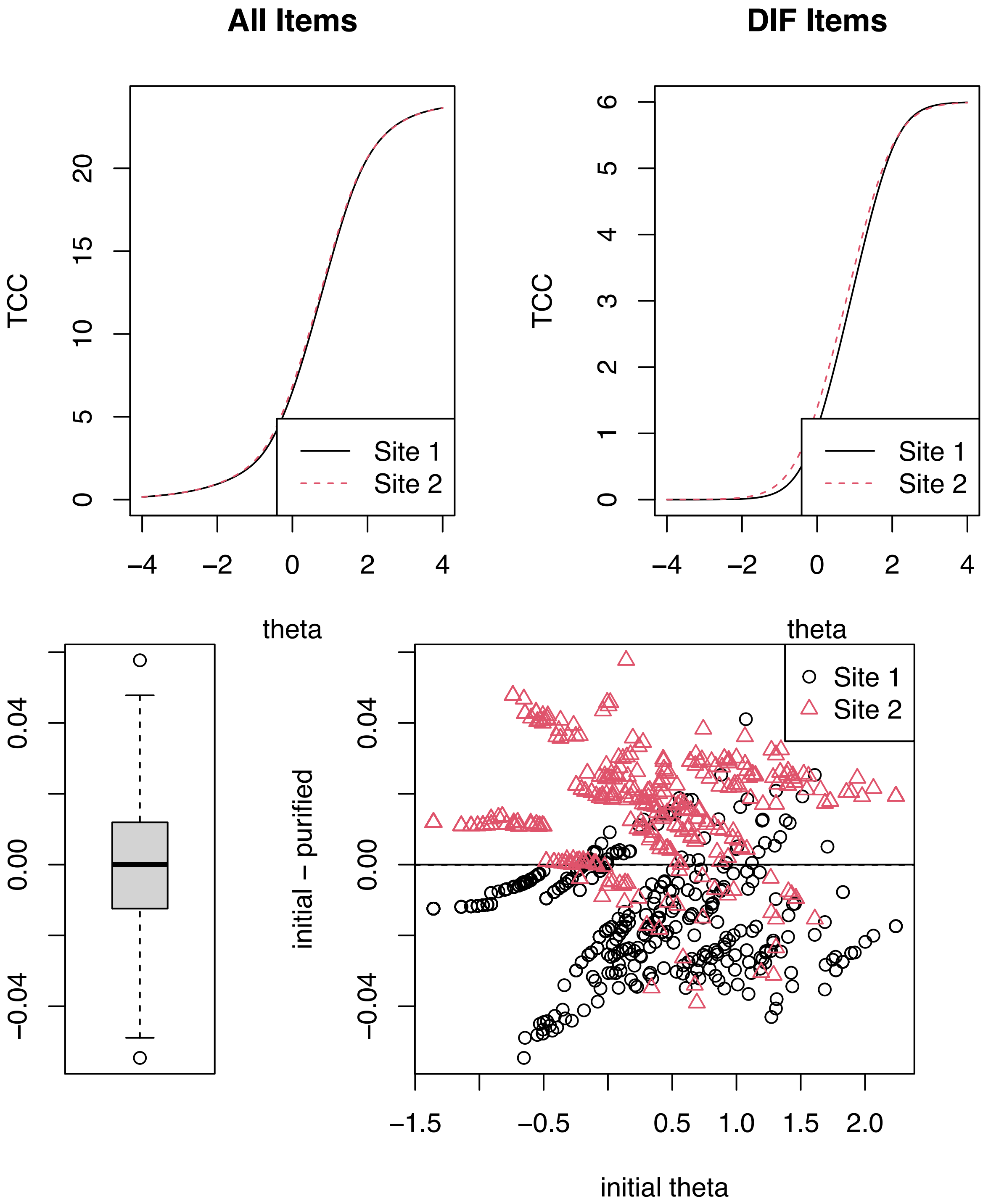

Back to top

Gender-based DIF: Trauma Reaction

```
## Call:
## lordif::lordif(resp.data = as.data.frame(sex.data), group = sex)
##
##   Number of DIF groups: 2
```

```
##
##   Number of items flagged for DIF: 1 of 4
##
##   Items flagged: 4
##
##   Number of iterations for purification: 2 of 10
##
##   Detection criterion: Chisqr
##
##   Threshold: alpha = 0.01
##
##   item ncat  chi12  chi13  chi23
## 1    1    7 0.3398 0.6171 0.8158
## 2    2    7 0.1989 0.2936 0.3708
## 3    3    7 0.1562 0.3659 0.9837
## 4    4    7 0.0005 0.0011 0.2069
```

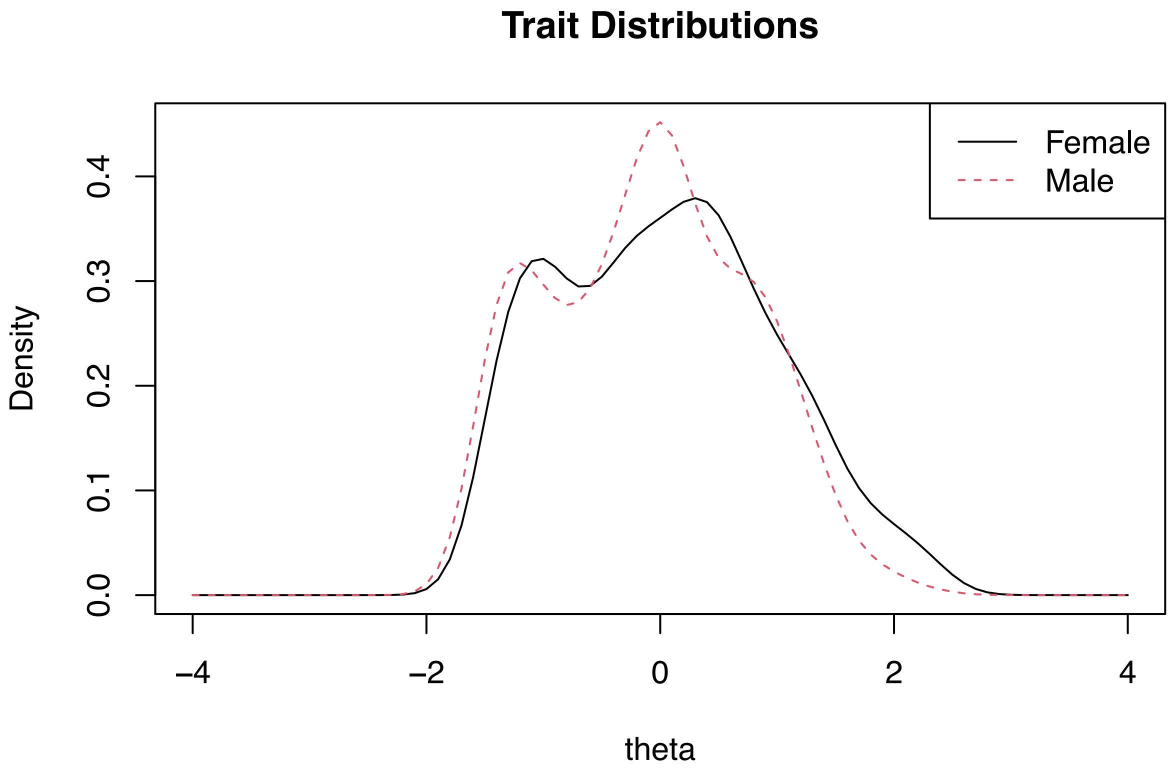

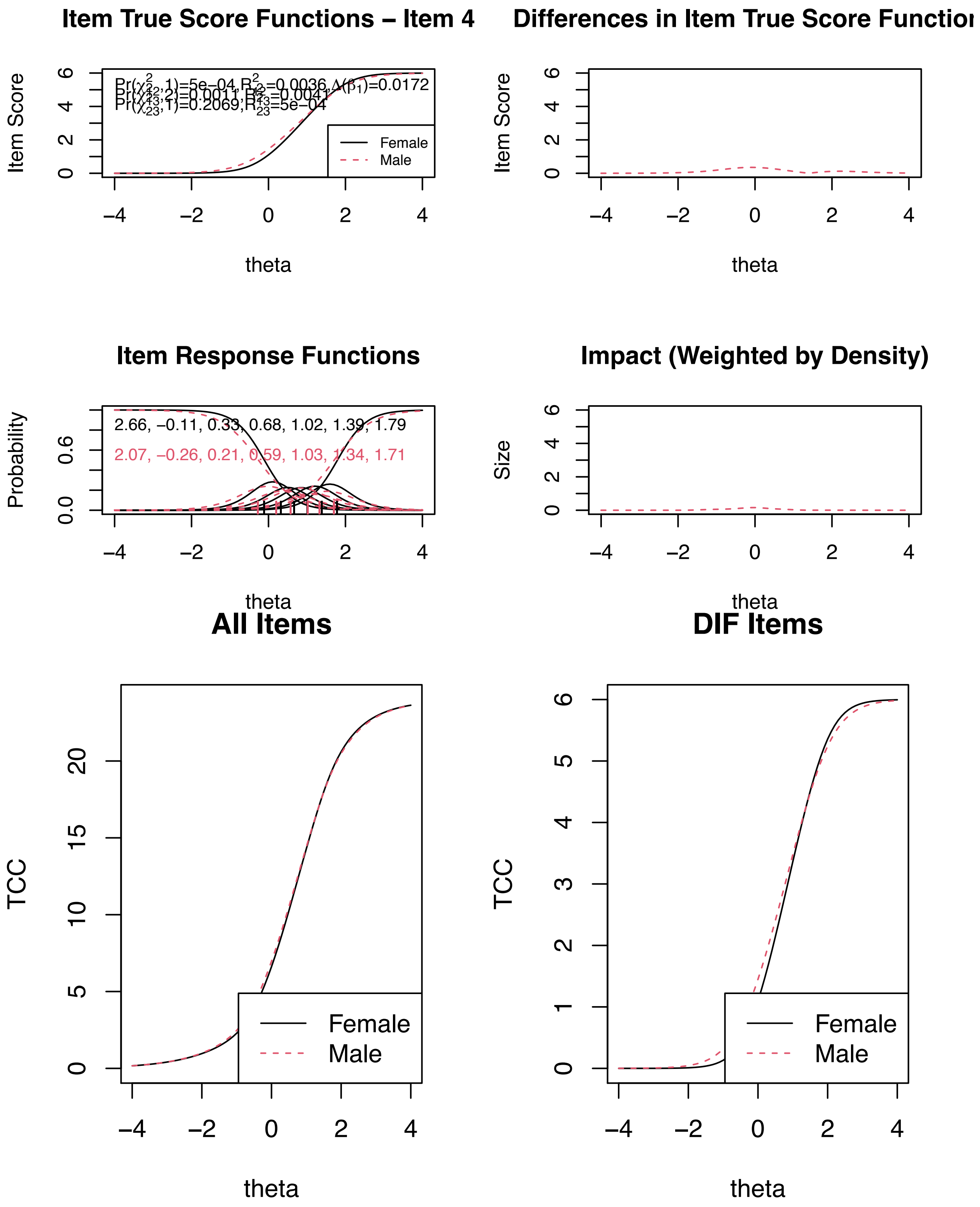

Item True Score Functions – Item 4
Differences in Item True Score Functio
Item Response Functions
Impact (Weighted by Density)
All Items
DIF Items
Item Score
Probability
Size
TCC
theta
Female
Male
2.66, −0.11, 0.33, 0.68, 1.02, 1.39, 1.79
2.07, −0.26, 0.21, 0.59, 1.03, 1.34, 1.71

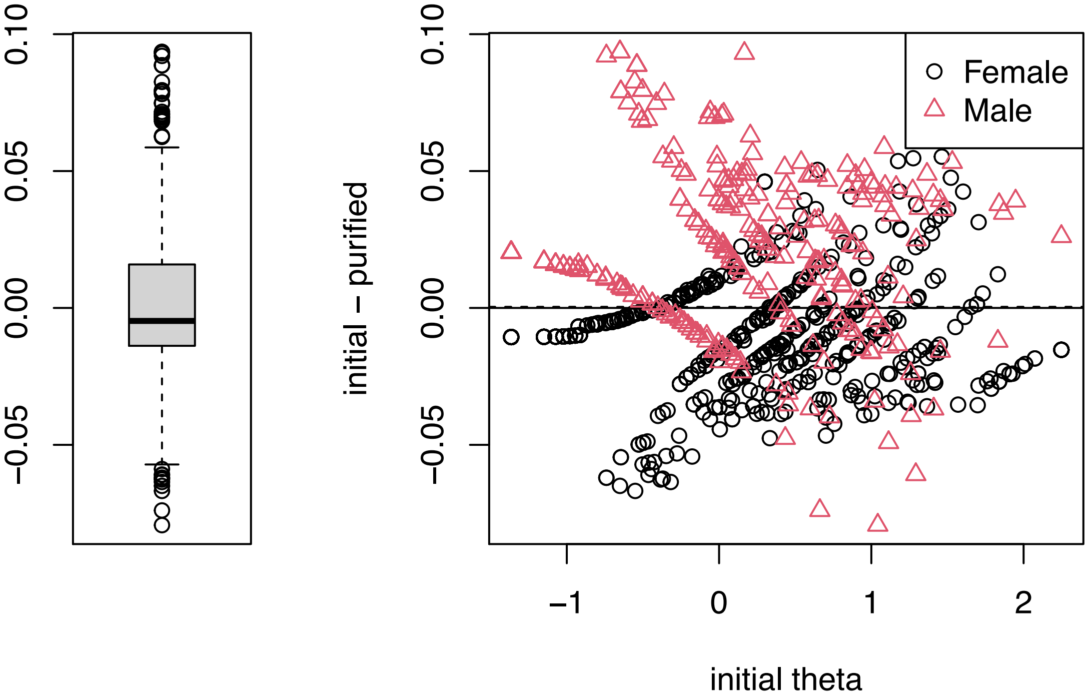

Age-based DIF: Trauma Reaction

```
## Call:
## lordif::lordif(resp.data = as.data.frame(age.data), group = age)
##
##   Number of DIF groups: 2
##
##   Number of items flagged for DIF: 0 of 4
##
##   Items flagged:
##
##   Number of iterations for purification: 1 of 10
##
##   Detection criterion: Chisqr
##
##   Threshold: alpha = 0.01
```

Back to top

Readiness for Recovery

Site 1

Reliability: Readiness for Recovery

```
## Cronbach's alpha is 0.407.
## Mean item-total correlation is 0.184.
## If each item were dropped:
##      raw_alpha std.alpha G6(smc) average_r  S/N alpha se var.r med.r
## Q133      0.26      0.26    0.15      0.15 0.35    0.059    NA  0.15
```

```
## Q134      0.23      0.24    0.13      0.13 0.31    0.060    NA  0.13
## Q135      0.42      0.42    0.27      0.27 0.73    0.046    NA  0.27
```

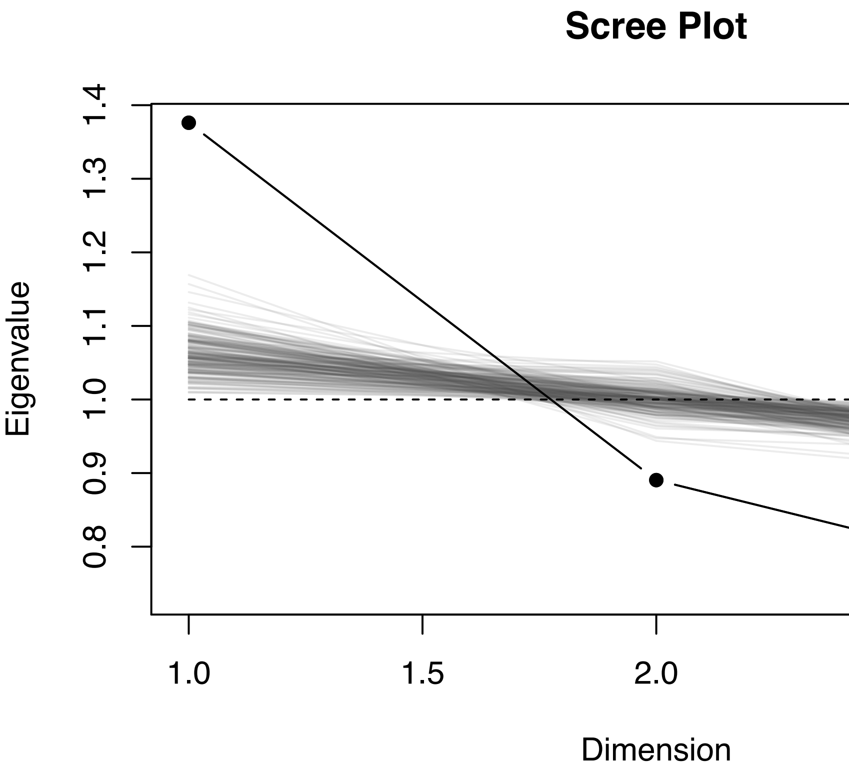

Unidimensionality: Readiness for Recovery

```
## [1] "Ratio of first to second eigenvalues: 1.545"

## [1] 1.3761048 0.8904652 0.7334300

## Factor Analysis using method =  minres
## Call: fa(r = grm_obj$X)
## Standardized loadings (pattern matrix) based upon correlation matrix
##       MR1    h2   u2 com
## Q133 0.49 0.240 0.76   1
## Q134 0.54 0.295 0.70   1
## Q135 0.28 0.078 0.92   1
##
##                 MR1
## SS loadings    0.61
## Proportion Var 0.20
##
## Mean item complexity =  1
## Test of the hypothesis that 1 factor is sufficient.
##
## The degrees of freedom for the null model are  3  and the objective function was  0.11 with Chi Squa
## The degrees of freedom for the model are 0  and the objective function was  0
##
## The root mean square of the residuals (RMSR) is  0
## The df corrected root mean square of the residuals is  NA
##
## The harmonic number of observations is  580 with the empirical chi square  0  with prob <  NA
## The total number of observations was  617  with Likelihood Chi Square =  0  with prob <  NA
```

```
##
## Tucker Lewis Index of factoring reliability =  -Inf
## Fit based upon off diagonal values = 1
## Measures of factor score adequacy
##                                                    MR1
## Correlation of (regression) scores with factors   0.67
## Multiple R square of scores with factors          0.45
## Minimum correlation of possible factor scores    -0.10
```

Graded-Response Model: Readiness for Recovery

```
##      Extrmt1 Extrmt2 Extrmt3 Extrmt4 Extrmt5 Extrmt6 Dscrmn
## Q133  -2.029  -1.128  -0.294   0.608   1.356   2.535  1.149
## Q134  -3.394  -2.757  -1.904  -1.095  -0.297   0.674  1.158
## Q135  -0.409   1.377   2.911   5.159   7.875  11.332  0.475
```

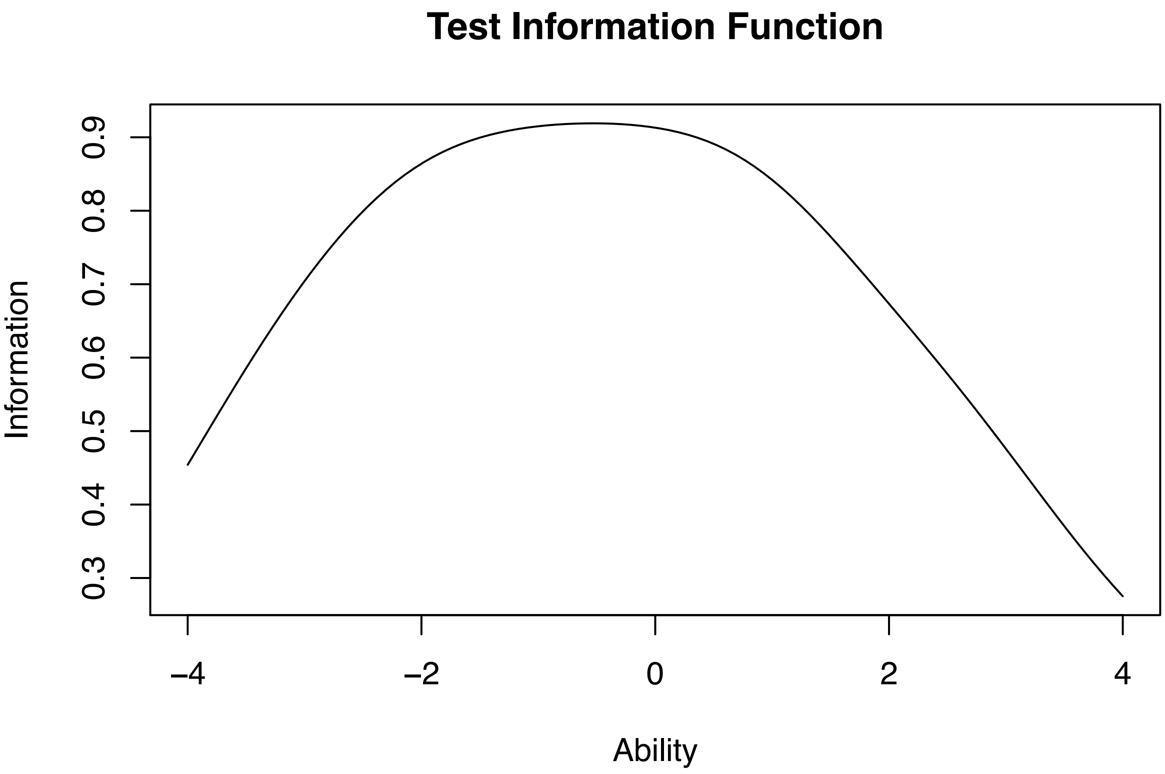

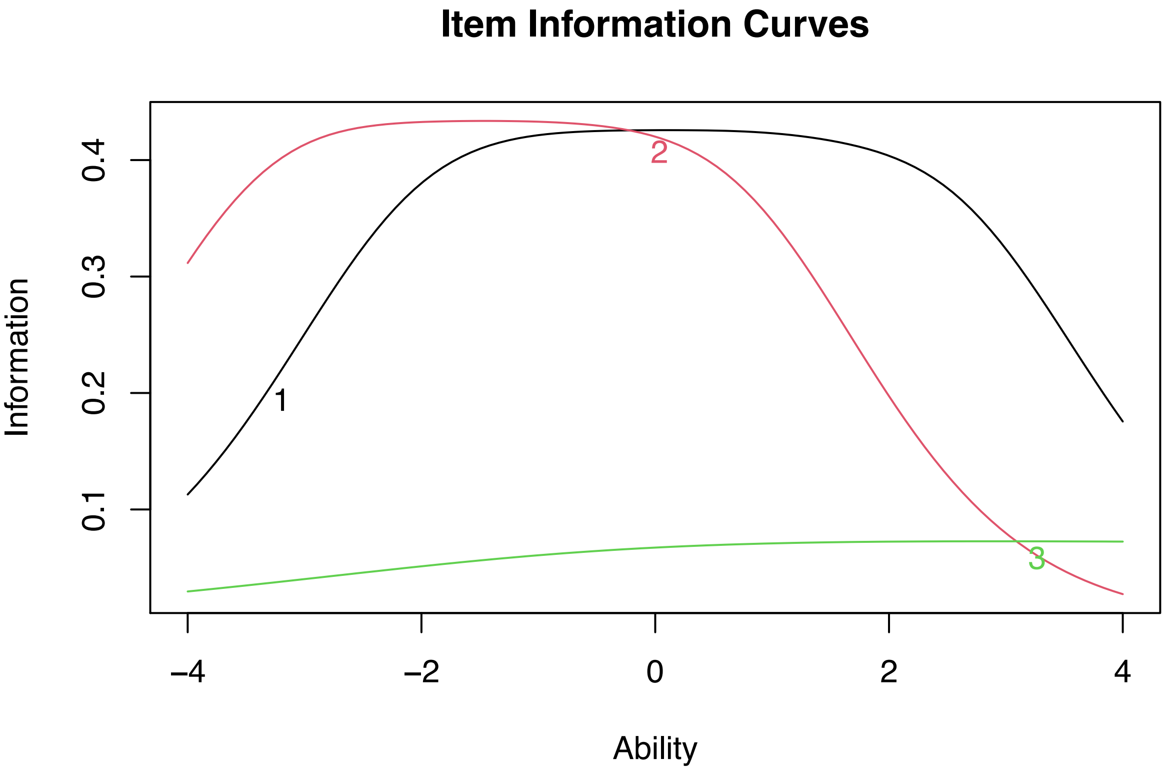

Back to top

Site 2

Reliability: Readiness for Recovery

```
## Cronbach's alpha is 0.441.
## Mean item-total correlation is 0.205.
## If each item were dropped:
##      raw_alpha std.alpha G6(smc) average_r  S/N alpha se var.r med.r
## Q133      0.20      0.20    0.11      0.11 0.25    0.065    NA  0.11
## Q134      0.31      0.31    0.19      0.19 0.45    0.056    NA  0.19
## Q135      0.48      0.48    0.32      0.32 0.94    0.042    NA  0.32
```

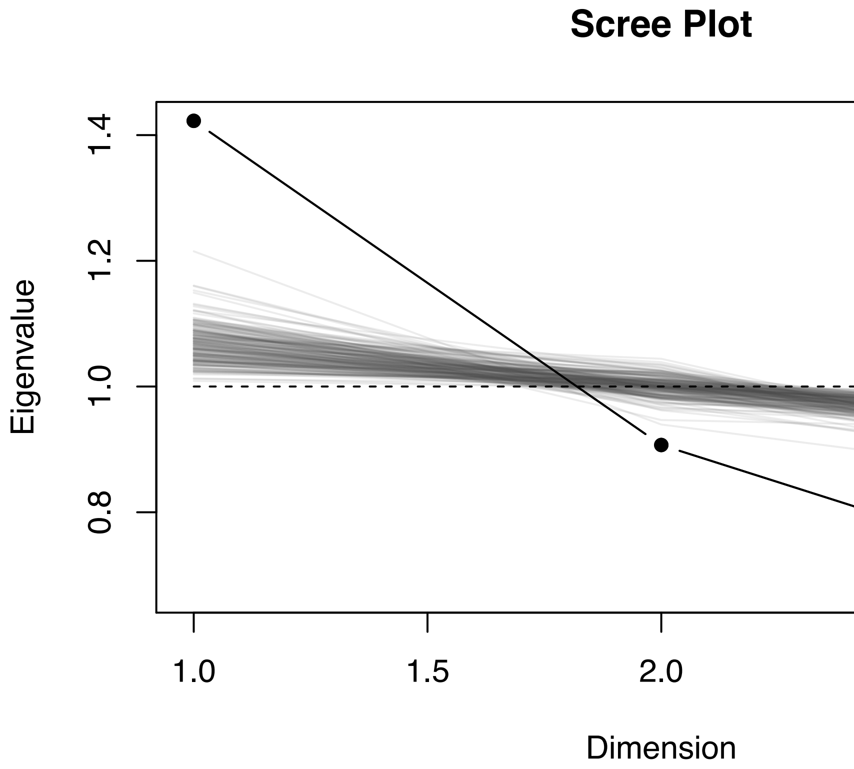

Unidimensionality: Readiness for Recovery

```
## [1] "Ratio of first to second eigenvalues: 1.569"

## [1] 1.4226754 0.9069711 0.6703535

## Factor Analysis using method =  minres
## Call: fa(r = grm_obj$X)
## Standardized loadings (pattern matrix) based upon correlation matrix
##       MR1    h2   u2 com
## Q133 0.73 0.537 0.46   1
## Q134 0.44 0.189 0.81   1
## Q135 0.25 0.064 0.94   1
##
##                 MR1
## SS loadings    0.79
## Proportion Var 0.26
##
## Mean item complexity =  1
## Test of the hypothesis that 1 factor is sufficient.
##
## The degrees of freedom for the null model are  3  and the objective function was  0.15 with Chi Squa
## The degrees of freedom for the model are 0  and the objective function was  0
##
## The root mean square of the residuals (RMSR) is  0
## The df corrected root mean square of the residuals is  NA
##
## The harmonic number of observations is  565 with the empirical chi square  0  with prob <  NA
## The total number of observations was  596  with Likelihood Chi Square =  0  with prob <  NA
##
## Tucker Lewis Index of factoring reliability =  -Inf
## Fit based upon off diagonal values = 1
## Measures of factor score adequacy
```

```
##                                                    MR1
## Correlation of (regression) scores with factors   0.77
## Multiple R square of scores with factors          0.59
## Minimum correlation of possible factor scores     0.19
```

Graded-Response Model: Readiness for Recovery

```
##      Extrmt1 Extrmt2 Extrmt3 Extrmt4 Extrmt5 Extrmt6 Dscrmn
## Q133  -1.403  -0.678  -0.008   0.751   1.598   2.670  1.594
## Q134  -3.089  -2.412  -1.619  -0.687   0.049   1.237  1.008
## Q135   0.079   1.181   2.381   4.147   5.219   7.678  0.573
```

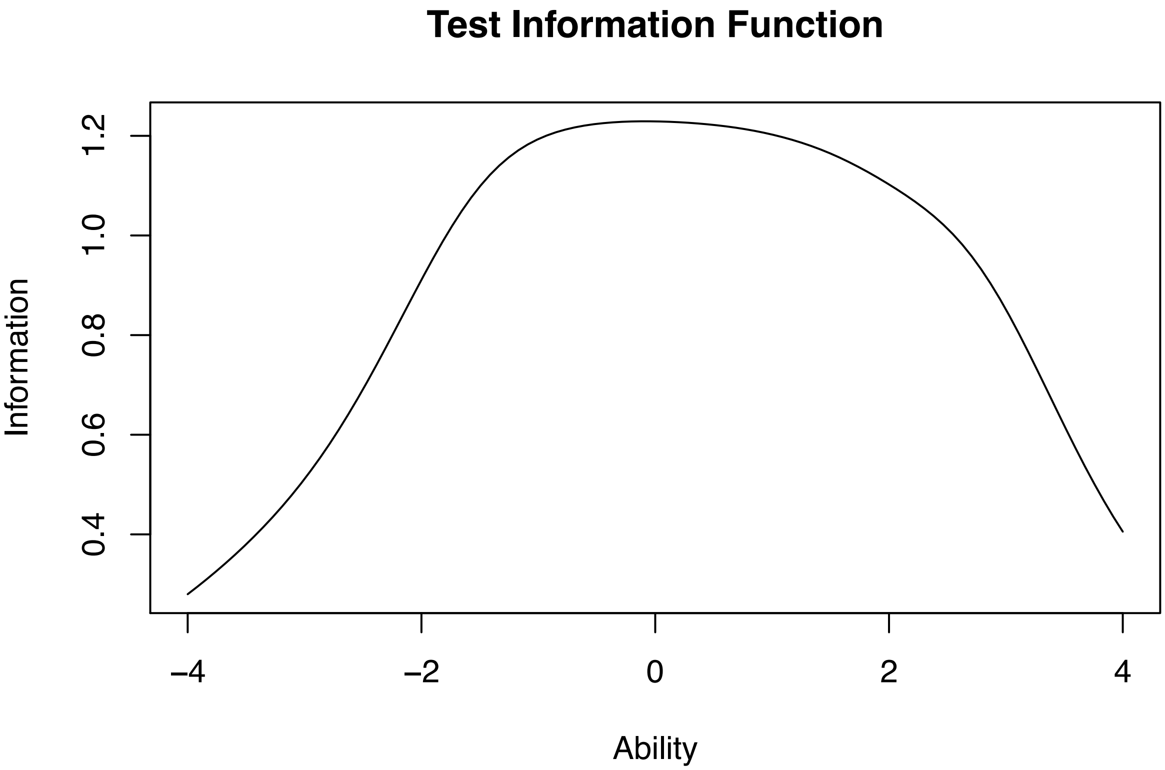

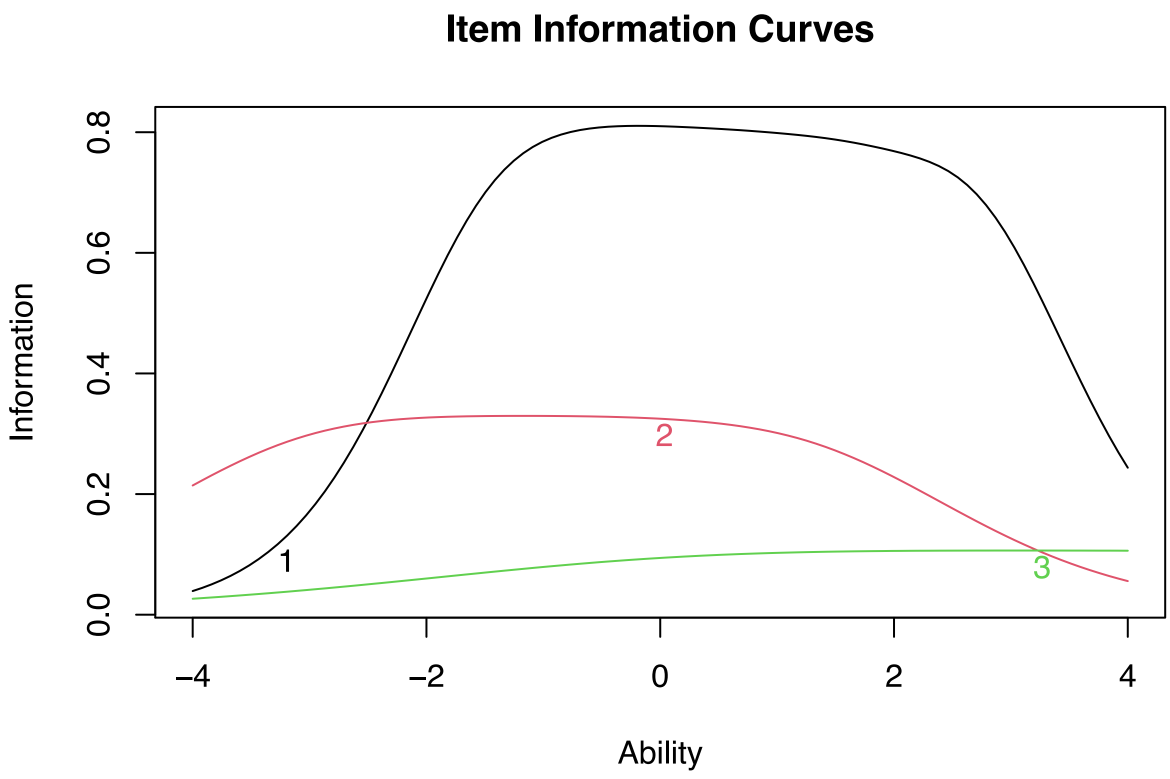

Back to top

Gender-based DIF: Readiness for Recovery

```
## No Gender-based DIF detected
```

Age-based DIF: Readiness for Recovery

```
## No age-based DIF detected
```

Back to top

Recovery Environment

Site 1

Reliability: Recovery Environment

```
## Cronbach's alpha is 0.643.
## Mean item-total correlation is 0.266.
## If each item were dropped:
##      raw_alpha std.alpha G6(smc) average_r S/N alpha se   var.r med.r
## Q80       0.60      0.60    0.53      0.27 1.5    0.026 0.00146  0.29
## Q136      0.58      0.58    0.51      0.26 1.4    0.027 0.00150  0.25
## Q138      0.60      0.60    0.53      0.27 1.5    0.026 0.00104  0.28
## Q139      0.60      0.60    0.53      0.27 1.5    0.026 0.00114  0.29
## Q137      0.58      0.58    0.51      0.26 1.4    0.028 0.00091  0.25
```

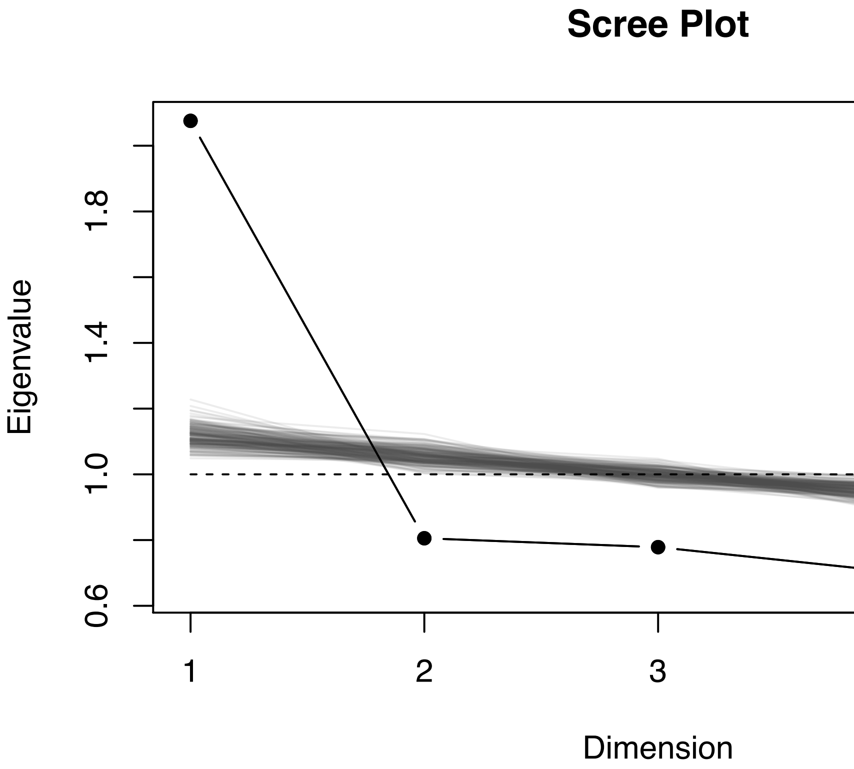

Unidimensionality: Recovery Environment

```
## [1] "Ratio of first to second eigenvalues: 2.577"

## [1] 2.0756735 0.8055102 0.7783887 0.7037467 0.6366809

## Factor Analysis using method =  minres
## Call: fa(r = grm_obj$X)
## Standardized loadings (pattern matrix) based upon correlation matrix
##       MR1   h2   u2 com
## Q80  0.50 0.25 0.75   1
## Q136 0.55 0.30 0.70   1
## Q138 0.50 0.25 0.75   1
## Q139 0.49 0.24 0.76   1
## Q137 0.56 0.31 0.69   1
##
##                 MR1
## SS loadings    1.35
## Proportion Var 0.27
##
## Mean item complexity =  1
## Test of the hypothesis that 1 factor is sufficient.
##
## The degrees of freedom for the null model are  10  and the objective function was  0.54 with Chi Squa
## The degrees of freedom for the model are 5  and the objective function was  0.01
##
## The root mean square of the residuals (RMSR) is  0.03
## The df corrected root mean square of the residuals is  0.04
##
## The harmonic number of observations is  563 with the empirical chi square  8.11  with prob <  0.15
## The total number of observations was  617  with Likelihood Chi Square =  8.49  with prob <  0.13
##
## Tucker Lewis Index of factoring reliability =  0.978
```

```
## RMSEA index =  0.034  and the 90 % confidence intervals are  0 0.071
## BIC =  -23.63
## Fit based upon off diagonal values = 0.99
## Measures of factor score adequacy
##                                                    MR1
## Correlation of (regression) scores with factors   0.81
## Multiple R square of scores with factors          0.65
## Minimum correlation of possible factor scores     0.30
```

Graded-Response Model: Recovery Environment

```
##      Extrmt1 Extrmt2 Extrmt3 Extrmt4 Extrmt5 Extrmt6 Dscrmn
## Q80   -0.614   0.120   0.776   1.468   2.287   3.476  1.159
## Q136  -1.205  -0.343   0.400   1.261   1.952   2.840  1.253
## Q138  -1.023  -0.359   0.218   0.902   1.580   2.352  1.134
## Q139  -1.797  -0.837   0.121   1.015   1.883   2.990  1.085
## Q137  -0.230   0.285   0.683   1.085   1.551   2.060  1.419
```

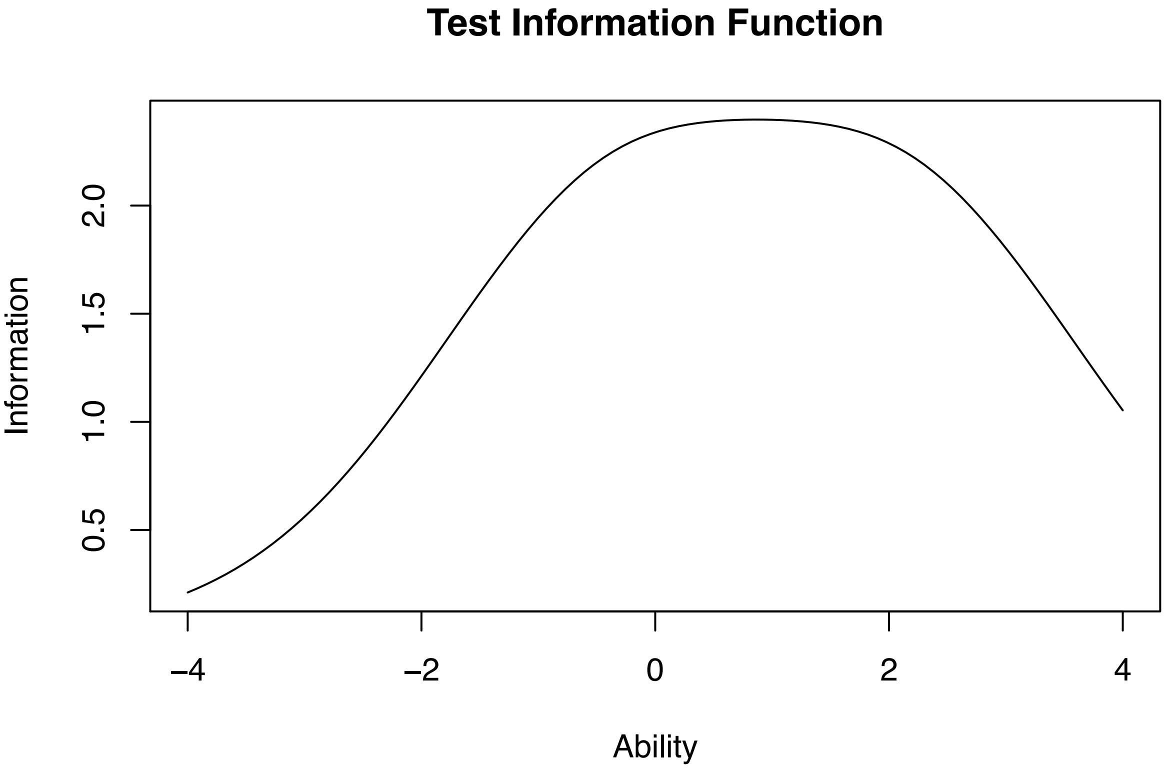

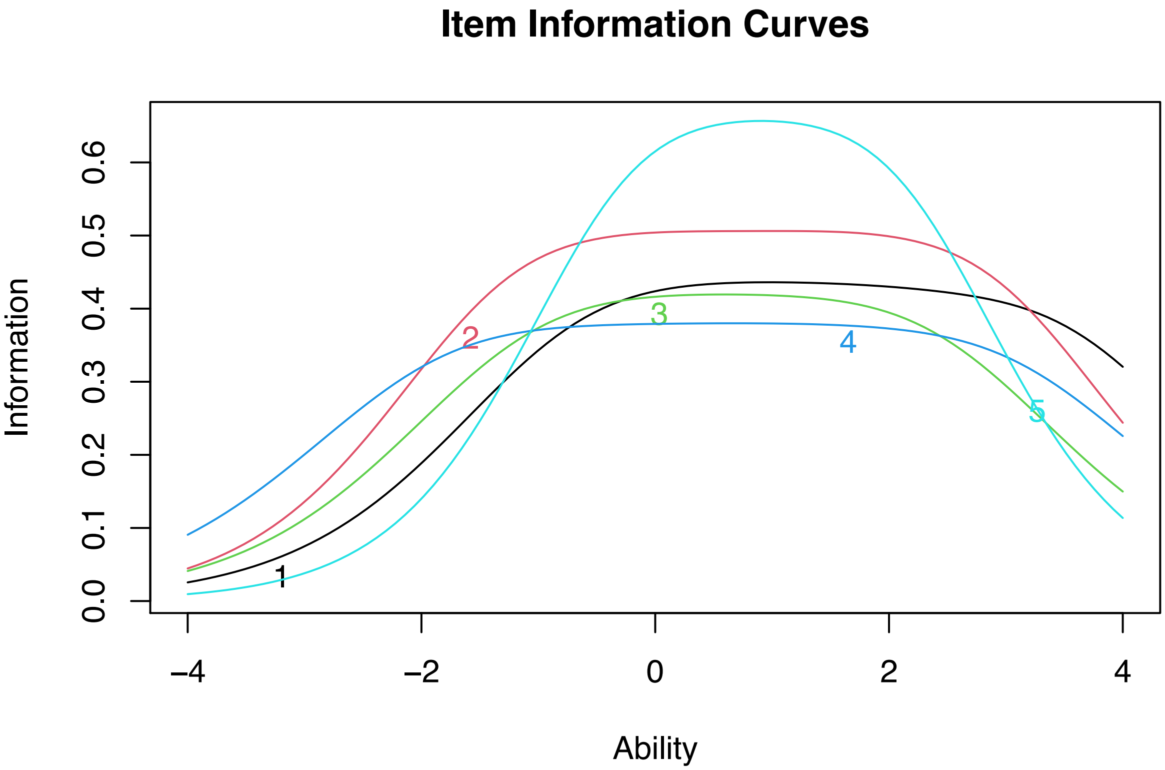

Back to top

Site 2

Reliability: Recovery Environment

```
## Cronbach's alpha is 0.633.
## Mean item-total correlation is 0.261.
## If each item were dropped:
##      raw_alpha std.alpha G6(smc) average_r S/N alpha se  var.r med.r
## Q80       0.58      0.58    0.52      0.26 1.4    0.028 0.0051  0.24
## Q136      0.56      0.56    0.50      0.24 1.3    0.029 0.0028  0.23
## Q138      0.58      0.59    0.53      0.26 1.4    0.028 0.0074  0.26
## Q139      0.56      0.57    0.50      0.25 1.3    0.029 0.0040  0.25
## Q137      0.61      0.62    0.55      0.29 1.6    0.026 0.0036  0.28
```

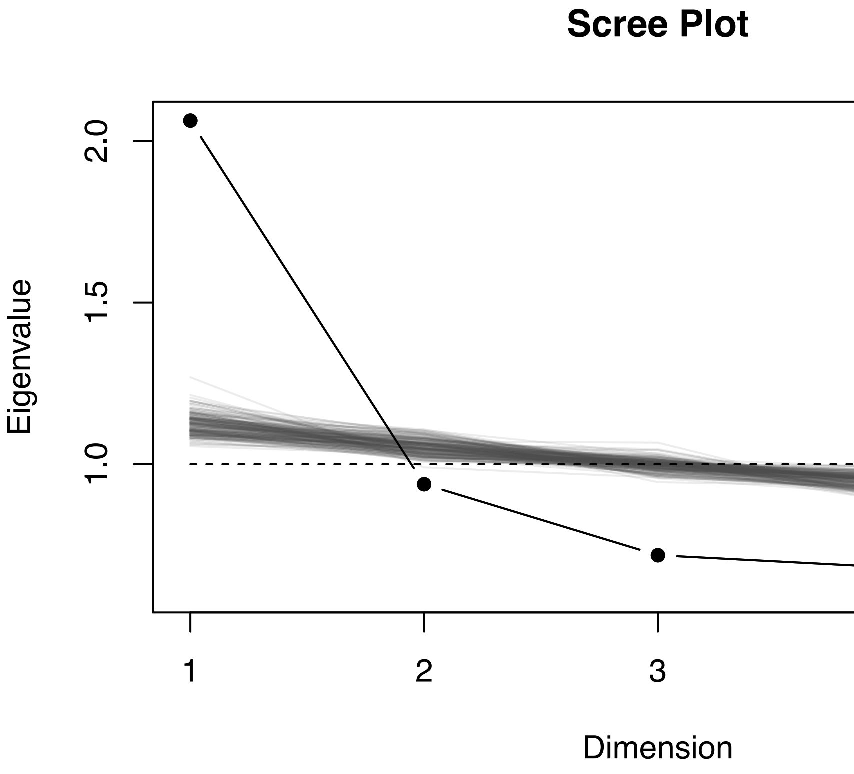

Unidimensionality: Recovery Environment

```
## [1] "Ratio of first to second eigenvalues: 2.199"

## [1] 2.0630248 0.9381012 0.7182923 0.6806631 0.5999186

## Factor Analysis using method =  minres
## Call: fa(r = grm_obj$X)
## Standardized loadings (pattern matrix) based upon correlation matrix
##       MR1   h2   u2 com
## Q80  0.52 0.28 0.72   1
## Q136 0.60 0.36 0.64   1
## Q138 0.47 0.22 0.78   1
## Q139 0.59 0.34 0.66   1
## Q137 0.39 0.15 0.85   1
##
##                 MR1
## SS loadings    1.35
## Proportion Var 0.27
##
## Mean item complexity =  1
## Test of the hypothesis that 1 factor is sufficient.
##
## The degrees of freedom for the null model are  10  and the objective function was  0.57 with Chi Squa
## The degrees of freedom for the model are 5  and the objective function was  0.04
##
## The root mean square of the residuals (RMSR) is  0.05
## The df corrected root mean square of the residuals is  0.07
##
## The harmonic number of observations is  553 with the empirical chi square  24.85  with prob <  0.000
## The total number of observations was  596  with Likelihood Chi Square =  21.92  with prob <  0.00054
##
## Tucker Lewis Index of factoring reliability =  0.896
```

```
## RMSEA index =  0.075  and the 90 % confidence intervals are  0.045 0.109
## BIC =  -10.03
## Fit based upon off diagonal values = 0.97
## Measures of factor score adequacy
##                                                   MR1
## Correlation of (regression) scores with factors   0.81
## Multiple R square of scores with factors          0.66
## Minimum correlation of possible factor scores     0.32
```

Graded-Response Model: Recovery Environment

```
##      Extrmt1 Extrmt2 Extrmt3 Extrmt4 Extrmt5 Extrmt6 Dscrmn
## Q80   -0.381   0.306   0.868   1.594   2.202   3.160  1.317
## Q136  -1.065  -0.232   0.315   1.010   1.645   2.396  1.538
## Q138  -0.739  -0.077   0.524   1.088   1.624   2.394  1.032
## Q139  -1.204  -0.420   0.321   1.114   1.757   3.051  1.466
## Q137   0.189   0.778   1.179   1.752   2.435   3.055  0.892
```

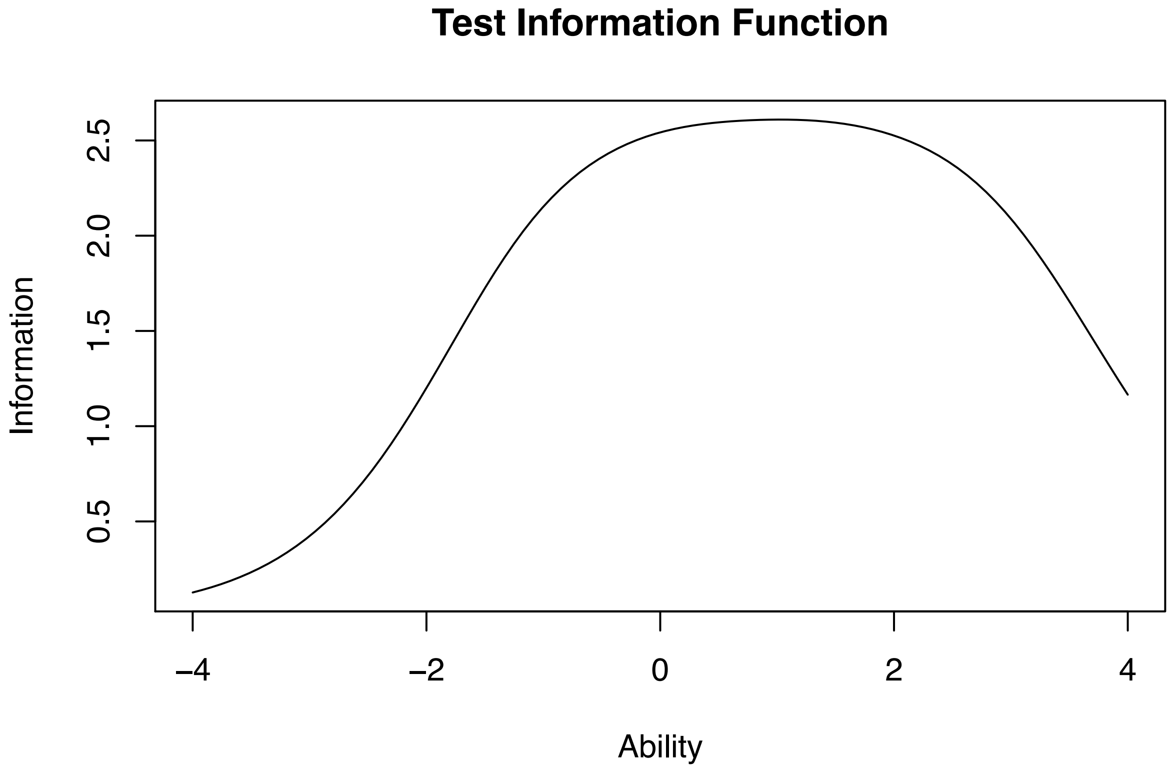

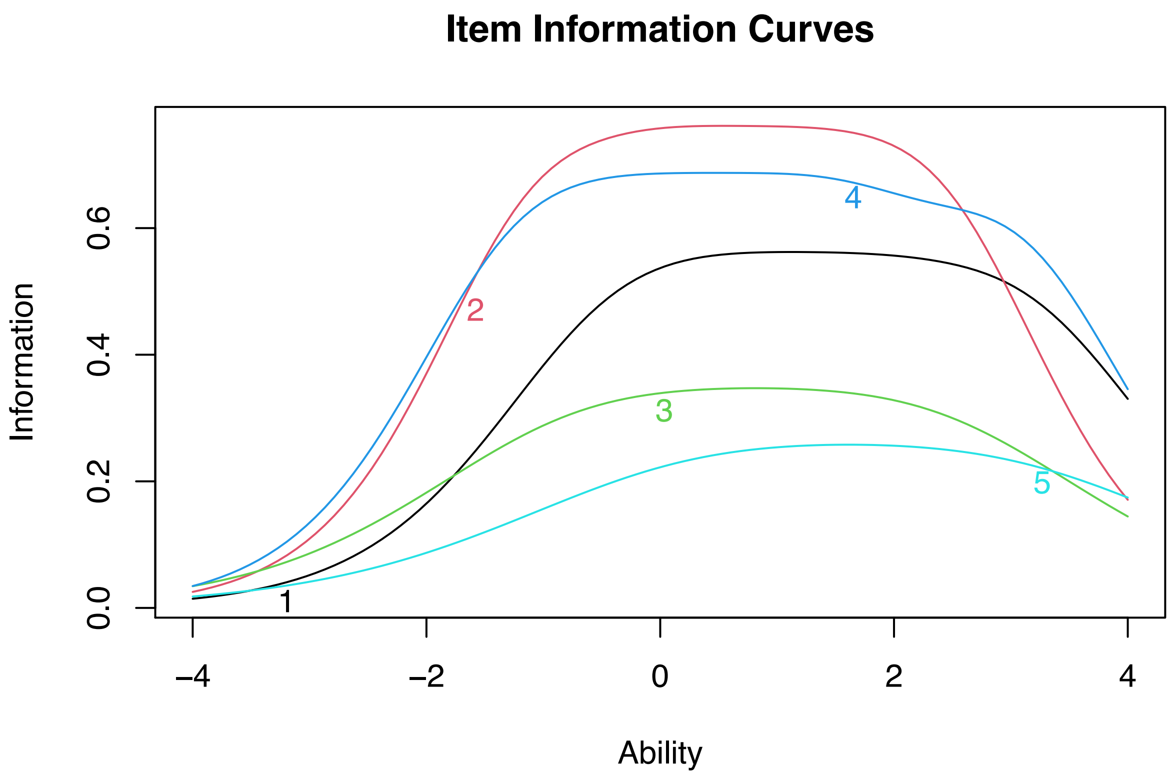

Back to top

Site DIF

```
## Call:
## lordif::lordif(resp.data = as.data.frame(merged_data), group = site)
##
##   Number of DIF groups: 2
##
##   Number of items flagged for DIF: 0 of 5
##
##   Items flagged:
##
##   Number of iterations for purification: 1 of 10
##
##   Detection criterion: Chisqr
##
##   Threshold: alpha = 0.01
```

Back to top

Gender-based DIF: Recovery Environment

```
## Call:
## lordif::lordif(resp.data = as.data.frame(sex.data), group = sex)
##
##   Number of DIF groups: 2
##
##   Number of items flagged for DIF: 2 of 5
```

```
##
##   Items flagged: 3, 4
##
##   Number of iterations for purification: 2 of 10
##
##   Detection criterion: Chisqr
##
##   Threshold: alpha = 0.01
##
##   item ncat  chi12  chi13  chi23
## 1    1    7 0.7349 0.7197 0.4611
## 2    2    7 0.7714 0.2528 0.1025
## 3    3    7 0.0000 0.0000 0.0021
## 4    4    7 0.0001 0.0006 0.7773
## 5    5    7 0.7025 0.9274 0.9449
```

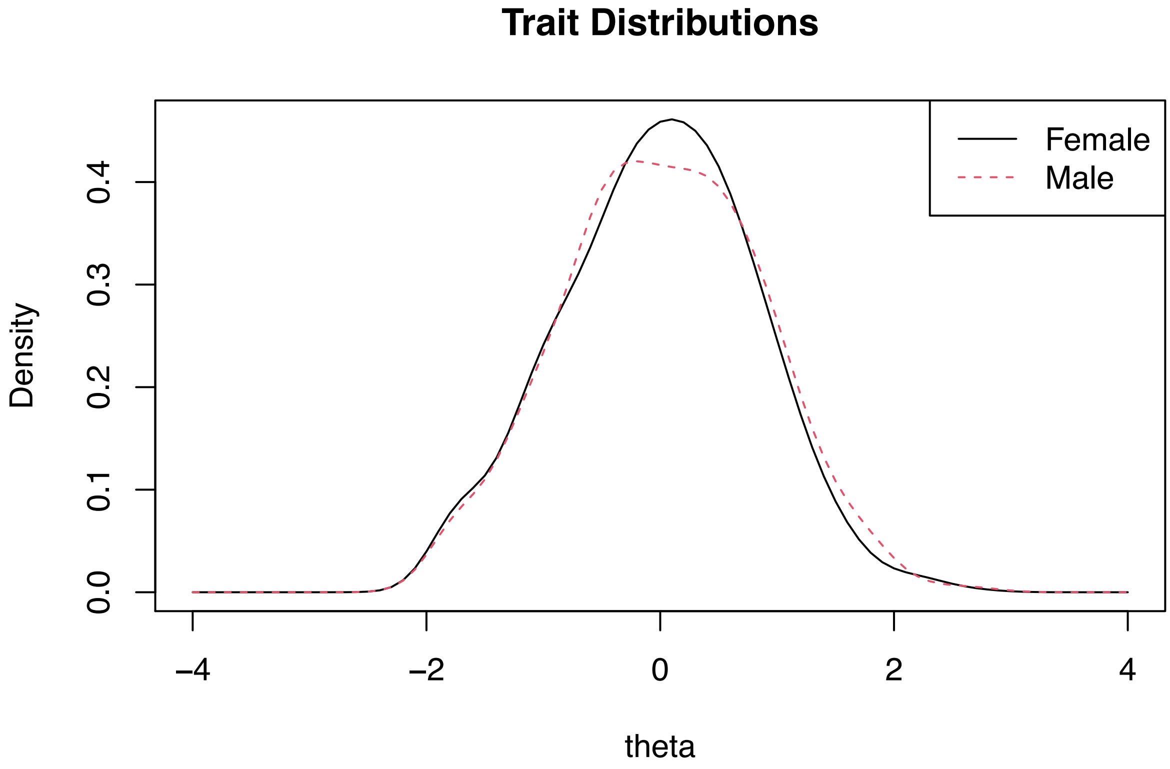

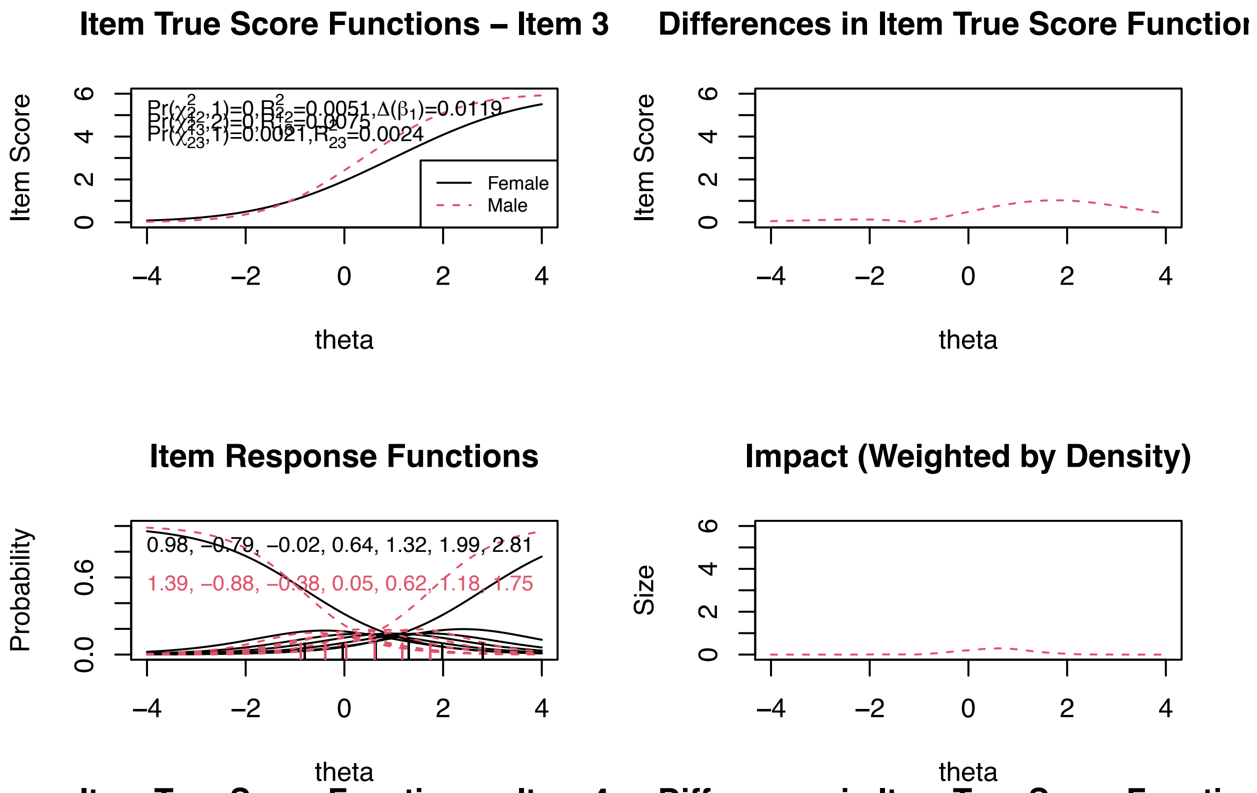
Item True Score Functions – Item 3
Differences in Item True Score Functio
Item Score
theta
Female
Male
Item Response Functions
Impact (Weighted by Density)
Probability
Size
0.98, –0.79, –0.02, 0.64, 1.32, 1.99, 2.81
1.39, –0.88, –0.38, 0.05, 0.62, 1.18, 1.75

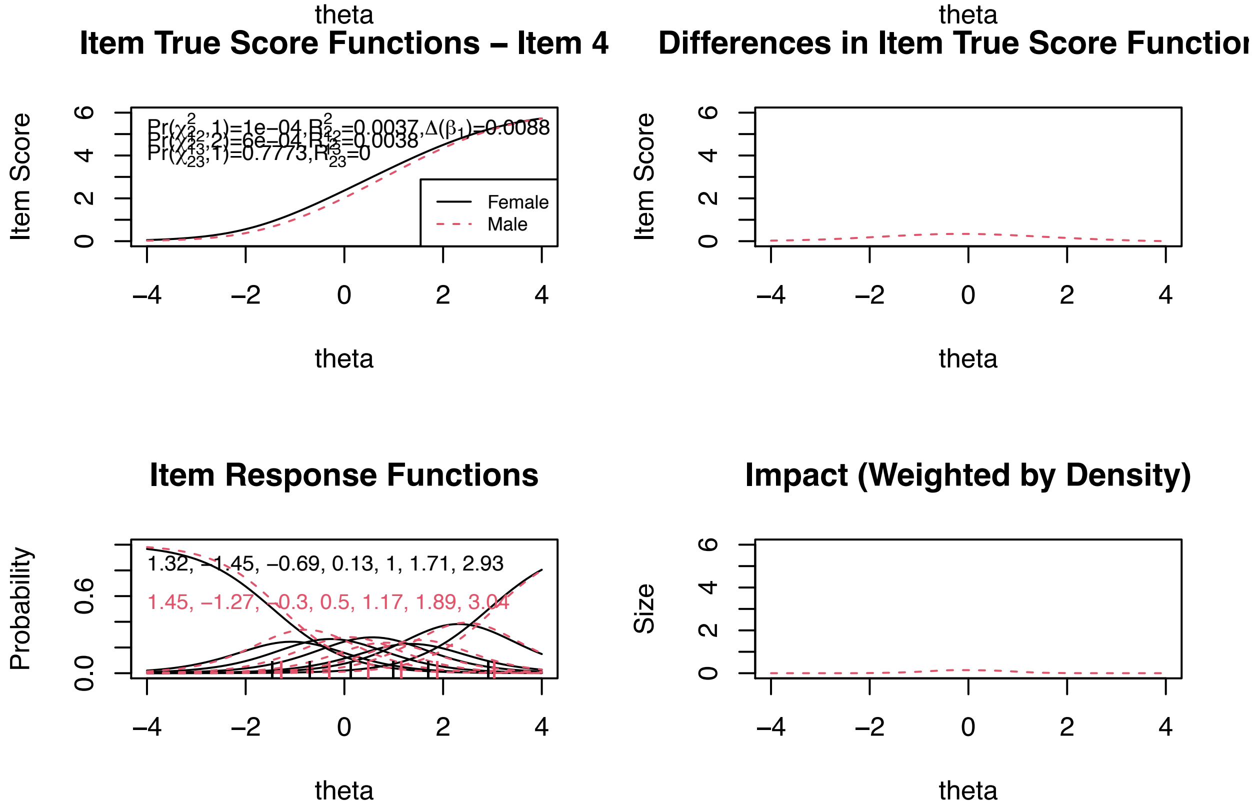
Item True Score Functions – Item 4
Differences in Item True Score Functio
Item Score
theta
Female
Male
Item Response Functions
Impact (Weighted by Density)
Probability
Size
1.32, –1.45, –0.69, 0.13, 1, 1.71, 2.93
1.45, –1.27, –0.3, 0.5, 1.17, 1.89, 3.04

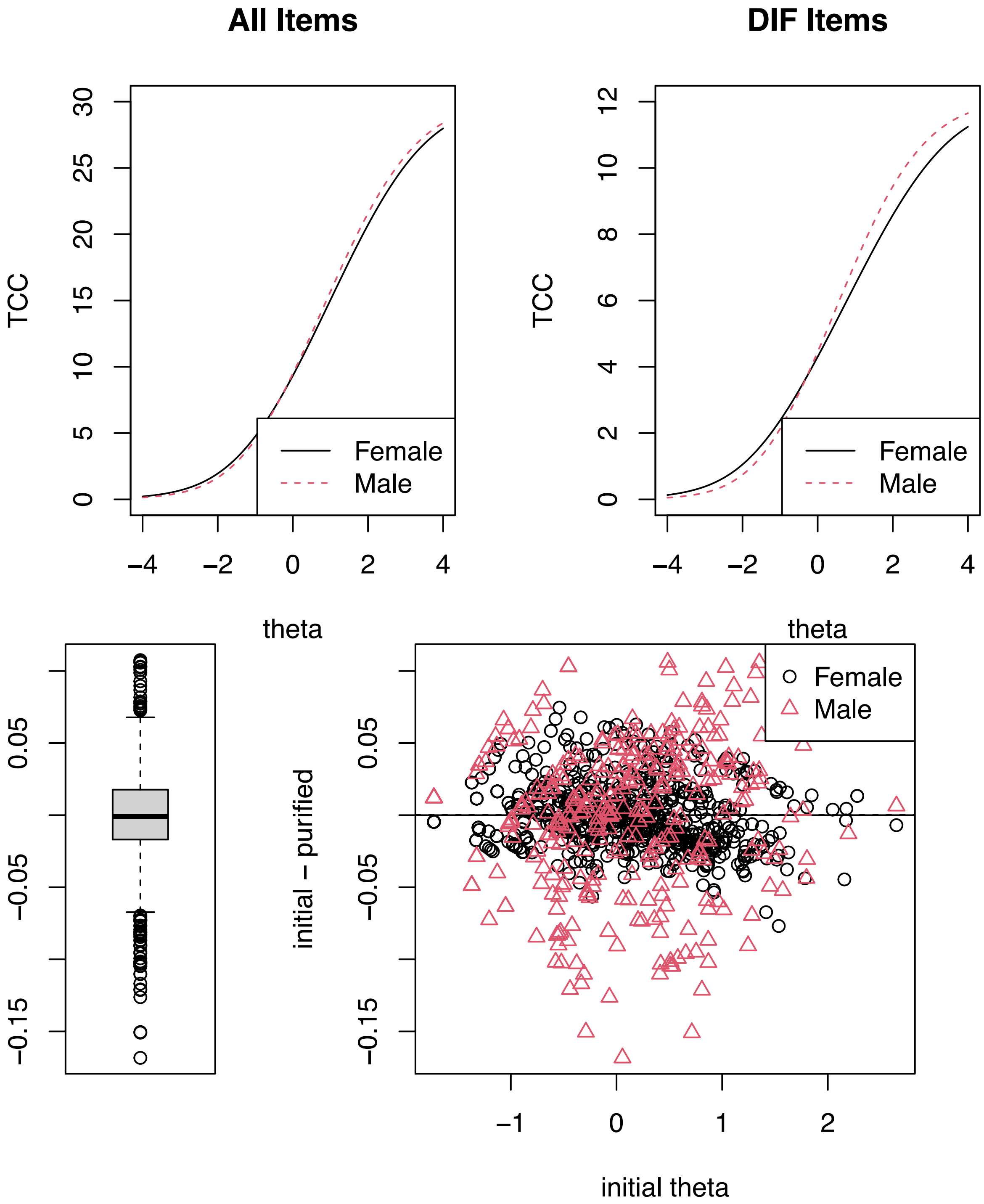

Age-based DIF: Recovery Environment

```
## Call:
## lordif::lordif(resp.data = as.data.frame(age.data), group = age)
##
##   Number of DIF groups: 2
##
```

```
##   Number of items flagged for DIF: 2 of 5
##
##   Items flagged: 1, 3
##
##   Number of iterations for purification: 2 of 10
##
##   Detection criterion: Chisqr
##
##   Threshold: alpha = 0.01
##
##   item ncat  chi12  chi13  chi23
## 1    1    7 0.0035 0.0103 0.4367
## 2    2    7 0.4265 0.7102 0.8199
## 3    3    7 0.0004 0.0000 0.0018
## 4    4    7 0.4924 0.7312 0.6940
## 5    5    7 0.9740 0.4214 0.1888
```

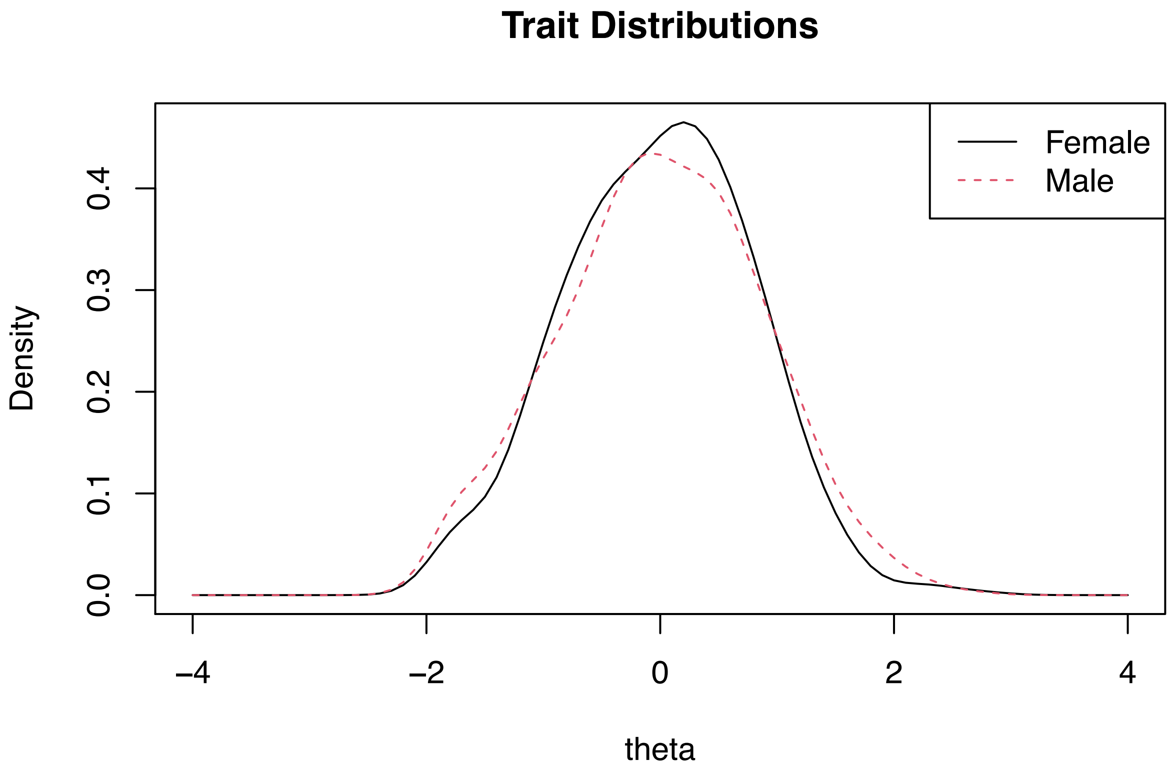

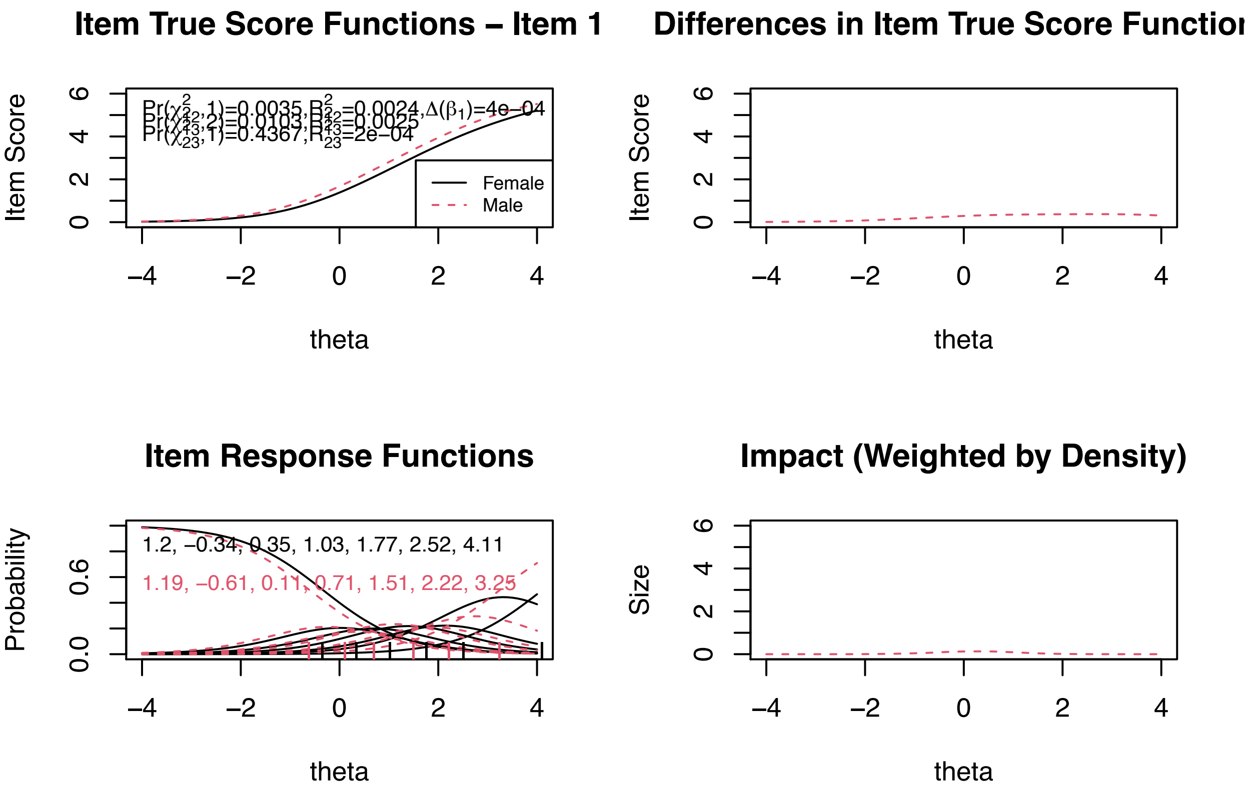
Item True Score Functions – Item 1
Differences in Item True Score Functio
Item Score
theta
Female
Male
Item Response Functions
Probability
1.2, −0.34, 0.35, 1.03, 1.77, 2.52, 4.11
1.19, −0.61, 0.11, 0.71, 1.51, 2.22, 3.25
Impact (Weighted by Density)
Size

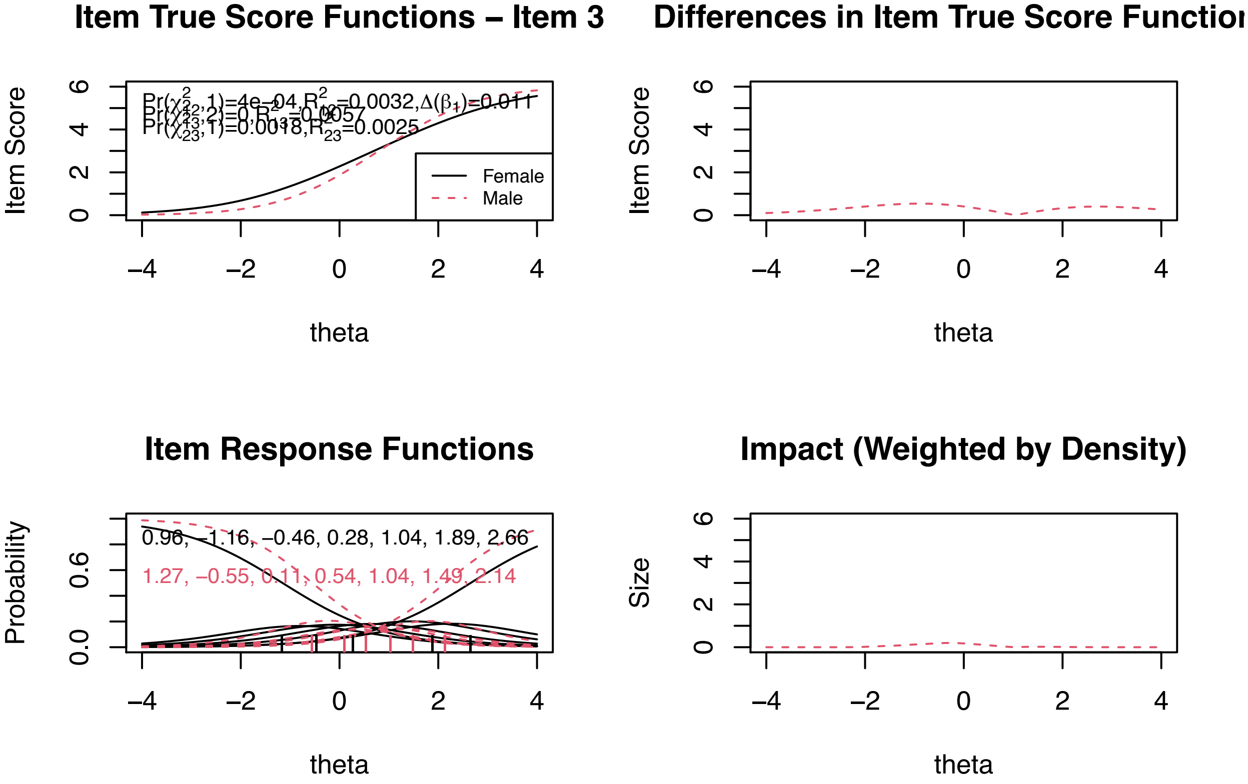
Item True Score Functions – Item 3
Differences in Item True Score Functio
Item Score
theta
Female
Male
Item Response Functions
Probability
0.96, −1.16, −0.46, 0.28, 1.04, 1.89, 2.66
1.27, −0.55, 0.11, 0.54, 1.04, 1.49, 2.14
Impact (Weighted by Density)
Size

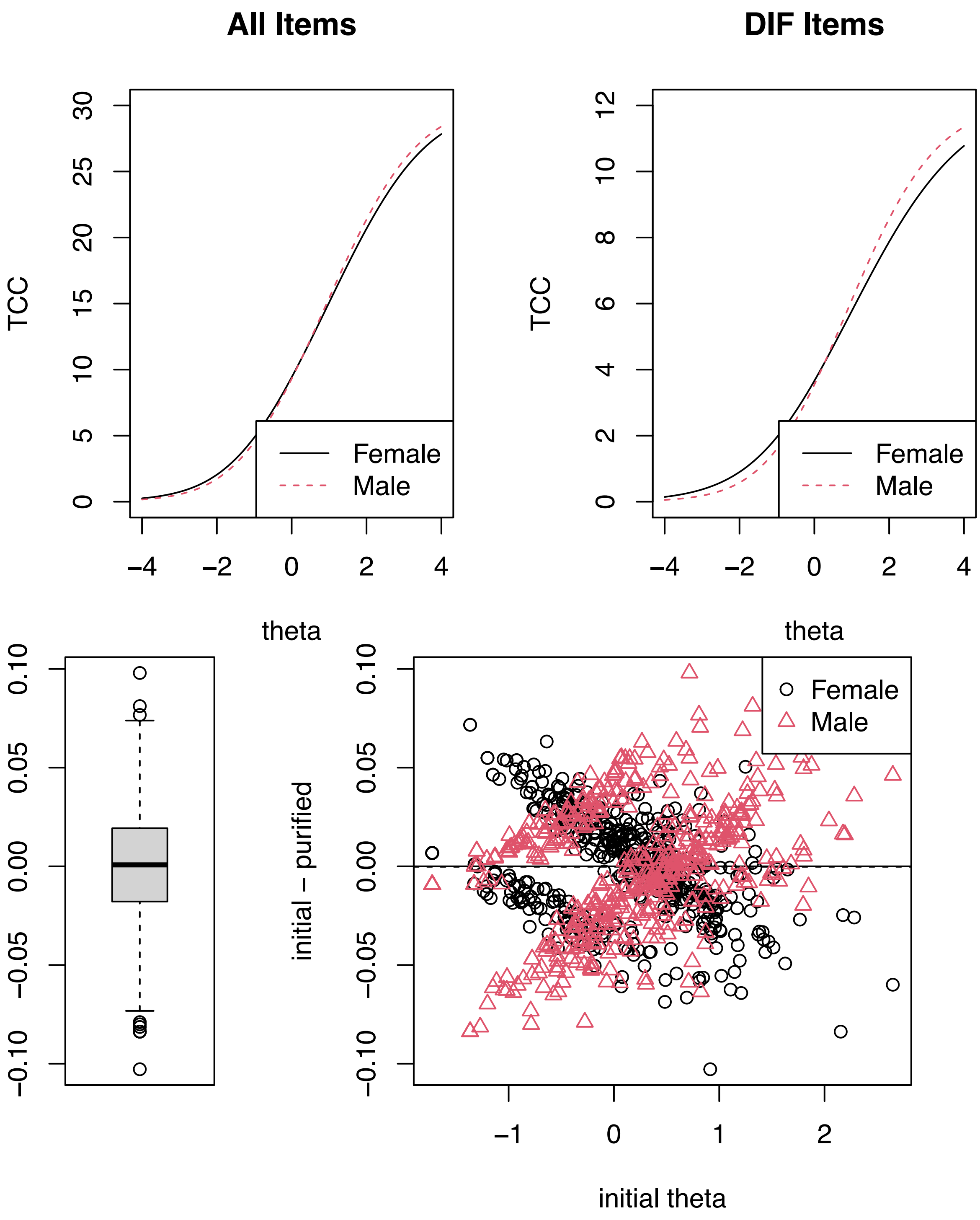

Back to top

Social Safety

Site 1

Reliability: Social Safety

```
## Cronbach's alpha is 0.749.
## Mean item-total correlation is 0.335.
## If each item were dropped:
##      raw_alpha std.alpha G6(smc) average_r S/N alpha se  var.r med.r
## Q43       0.69      0.69    0.66      0.31 2.2    0.020 0.0106  0.31
## Q131      0.71      0.71    0.68      0.33 2.4    0.019 0.0138  0.36
## Q40       0.69      0.69    0.66      0.31 2.2    0.020 0.0111  0.31
## Q132      0.75      0.75    0.72      0.38 3.1    0.016 0.0067  0.38
## Q62       0.70      0.71    0.68      0.33 2.4    0.019 0.0121  0.32
## Q50       0.73      0.74    0.71      0.36 2.8    0.017 0.0106  0.38
```

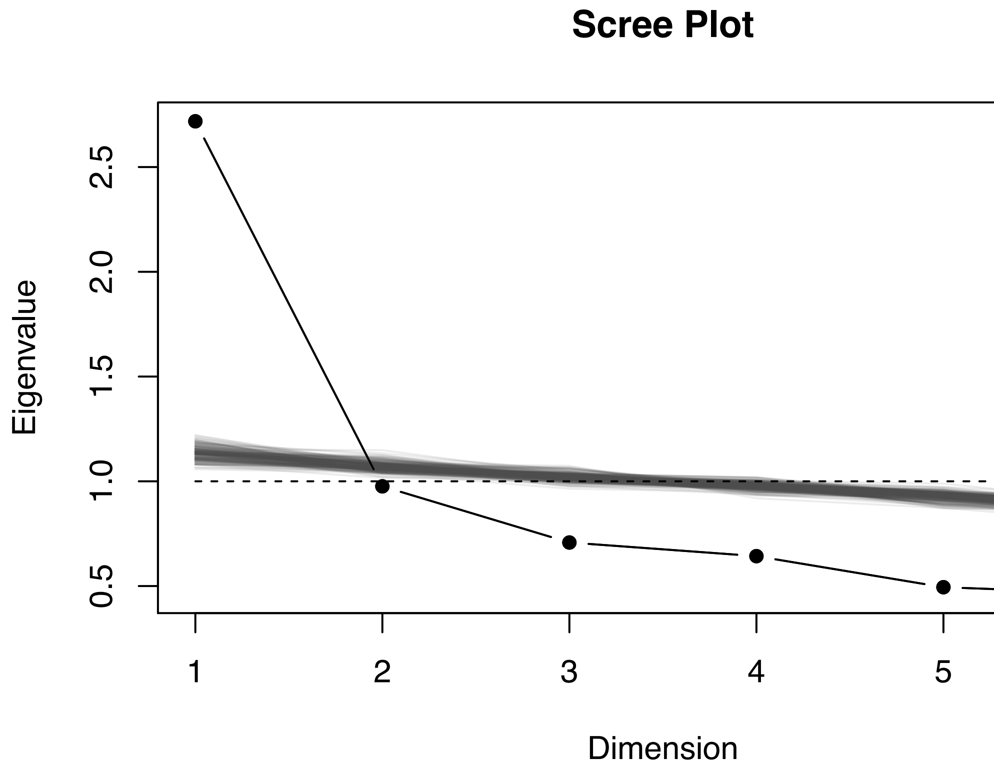

Unidimensionality: Social Safety

```
## [1] "Ratio of first to second eigenvalues: 2.786"
```

```
## [1] 2.7184604 0.9758992 0.7076665 0.6426698 0.4942761 0.4610280
```

```
## Factor Analysis using method =  minres
## Call: fa(r = grm_obj$X)
## Standardized loadings (pattern matrix) based upon correlation matrix
##       MR1   h2   u2 com
## Q43  0.71 0.50 0.50   1
## Q131 0.61 0.37 0.63   1
## Q40  0.68 0.46 0.54   1
## Q132 0.41 0.17 0.83   1
## Q62  0.62 0.38 0.62   1
## Q50  0.48 0.23 0.77   1
##
##                 MR1
## SS loadings    2.10
## Proportion Var 0.35
##
```

```
## Mean item complexity =  1
## Test of the hypothesis that 1 factor is sufficient.
##
## The degrees of freedom for the null model are  15  and the objective function was  1.29 with Chi Squa
## The degrees of freedom for the model are 9  and the objective function was  0.12
##
## The root mean square of the residuals (RMSR) is  0.06
## The df corrected root mean square of the residuals is  0.08
##
## The harmonic number of observations is  586 with the empirical chi square  63.24  with prob <  3.2e-
## The total number of observations was  617  with Likelihood Chi Square =  71.46  with prob <  7.9e-12
##
## Tucker Lewis Index of factoring reliability =  0.866
## RMSEA index =  0.106  and the 90 % confidence intervals are  0.084 0.13
## BIC =  13.64
## Fit based upon off diagonal values = 0.97
## Measures of factor score adequacy
##                                                    MR1
## Correlation of (regression) scores with factors   0.88
## Multiple R square of scores with factors          0.78
## Minimum correlation of possible factor scores     0.56
```

Graded-Response Model: Social Safety

```
##      Extrmt1 Extrmt2 Extrmt3 Extrmt4 Extrmt5 Extrmt6 Dscrmn
## Q43   -2.075  -1.407  -0.880  -0.295   0.201   0.926  1.997
## Q131  -1.805  -1.100  -0.522   0.303   0.922   1.968  1.483
## Q40   -2.454  -1.722  -1.060  -0.311   0.380   1.449  1.685
## Q132  -2.662  -1.523  -0.844   0.193   1.193   2.820  0.797
## Q62   -1.001  -0.360   0.175   0.684   1.239   1.964  1.603
## Q50   -2.966  -2.017  -1.434  -0.694   0.060   1.072  1.062
```

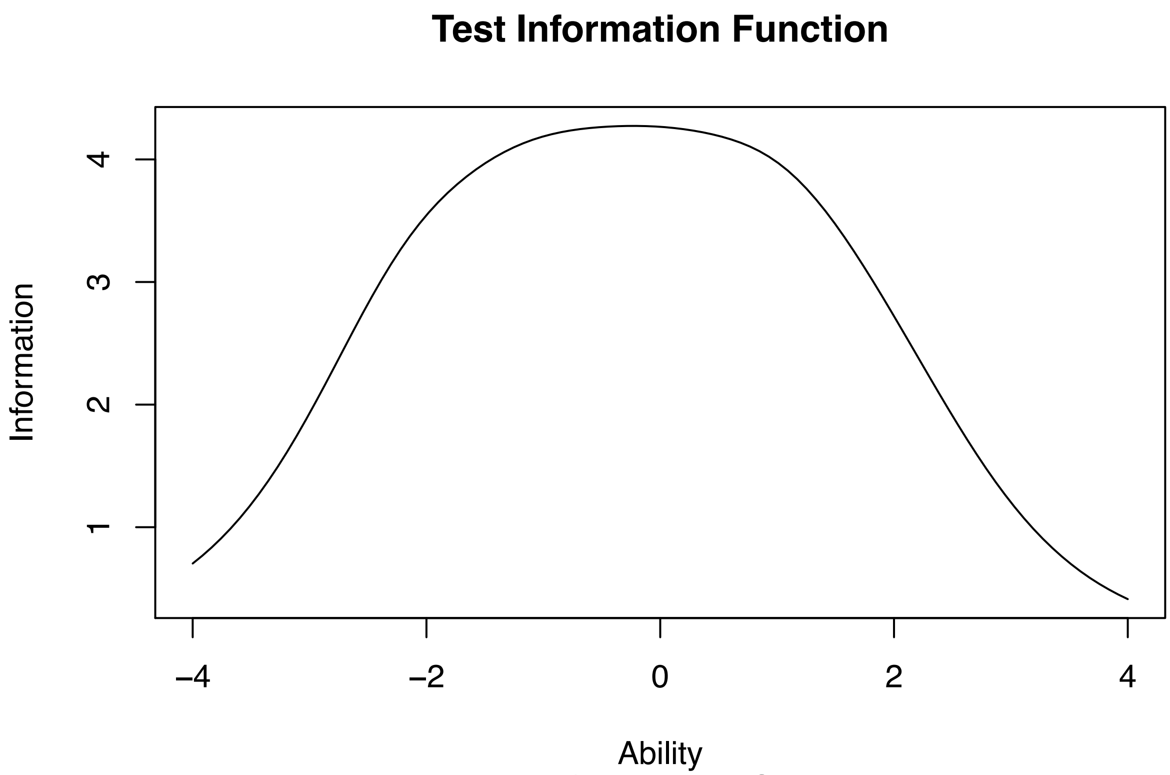

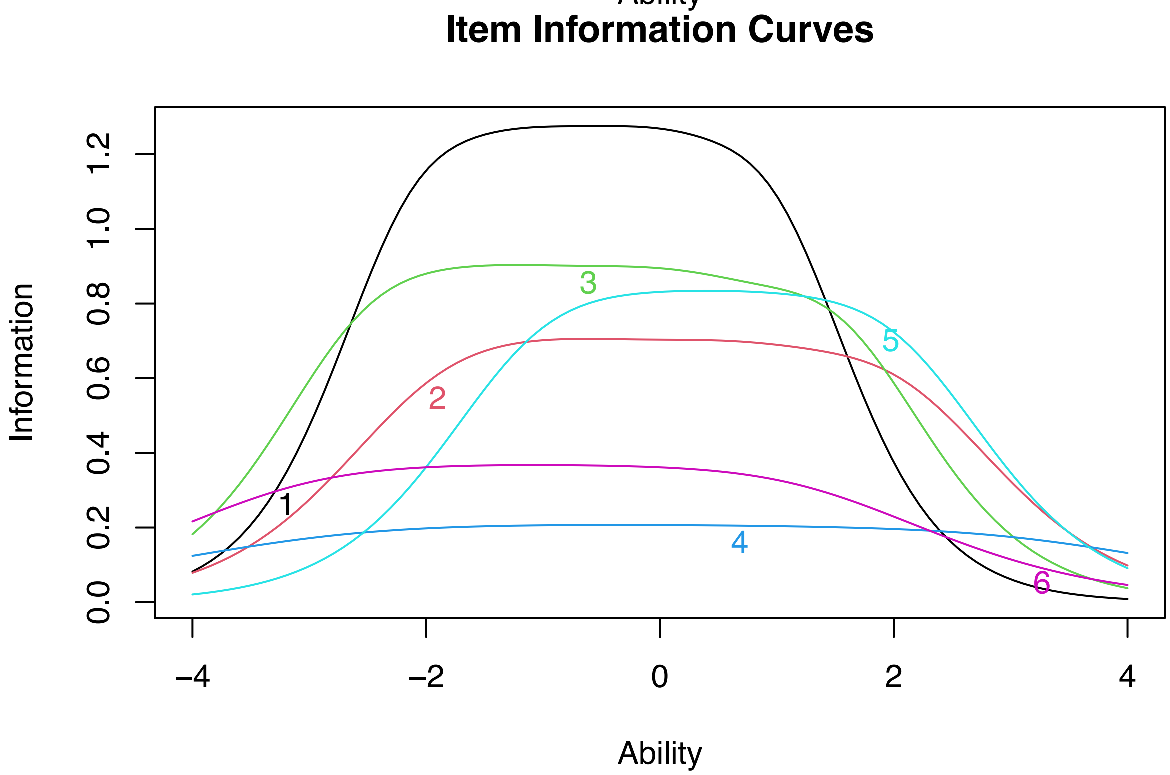

Back to top

Site 2

Reliability: Social Safety

```
## Cronbach's alpha is 0.742.
## Mean item-total correlation is 0.325.
## If each item were dropped:
##      raw_alpha std.alpha G6(smc) average_r S/N alpha se  var.r med.r
## Q43       0.69      0.69    0.66      0.31 2.3    0.020 0.0120  0.34
## Q131      0.69      0.69    0.67      0.31 2.3    0.020 0.0145  0.34
## Q40       0.69      0.69    0.66      0.31 2.2    0.020 0.0167  0.33
## Q132      0.76      0.76    0.72      0.39 3.2    0.016 0.0026  0.38
## Q62       0.69      0.69    0.66      0.31 2.2    0.020 0.0116  0.35
## Q50       0.70      0.70    0.67      0.32 2.4    0.019 0.0111  0.34
```

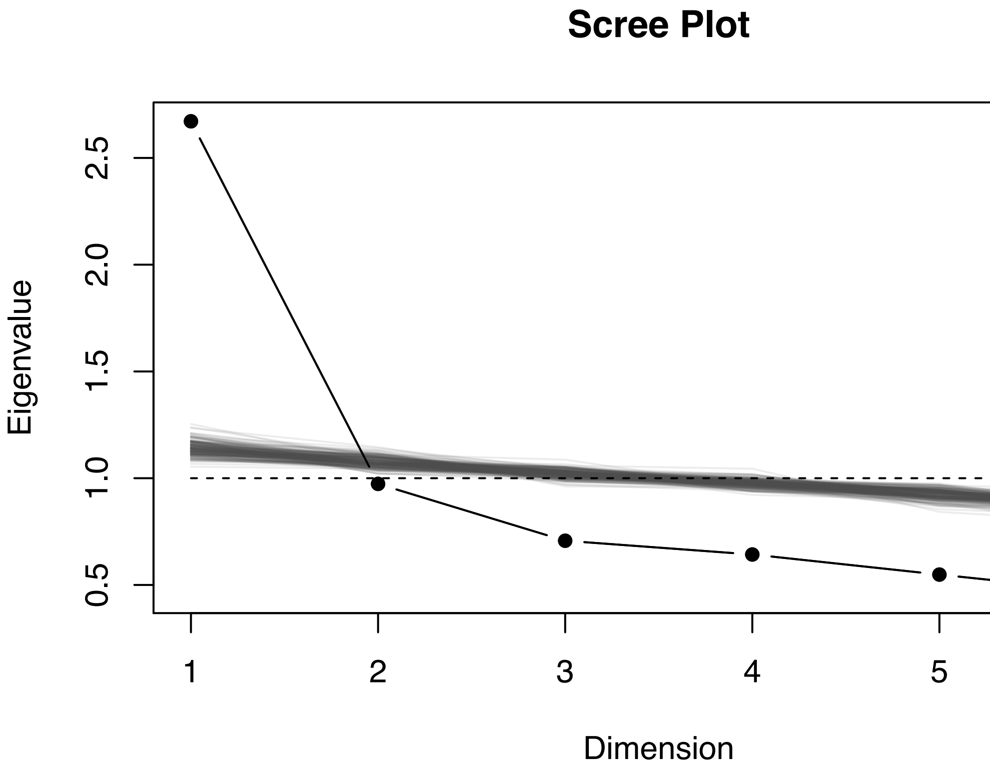

Unidimensionality: Social Safety

```
## [1] "Ratio of first to second eigenvalues: 2.745"
```

```
## [1] 2.6716093 0.9733936 0.7070720 0.6426684 0.5485788 0.4566778
```

```
## Factor Analysis using method =  minres
## Call: fa(r = grm_obj$X)
## Standardized loadings (pattern matrix) based upon correlation matrix
##       MR1   h2   u2 com
## Q43  0.63 0.40 0.60   1
## Q131 0.62 0.39 0.61   1
## Q40  0.63 0.39 0.61   1
## Q132 0.32 0.10 0.90   1
## Q62  0.64 0.40 0.60   1
## Q50  0.60 0.36 0.64   1
##
##                 MR1
## SS loadings    2.04
## Proportion Var 0.34
##
```

```
## Mean item complexity =  1
## Test of the hypothesis that 1 factor is sufficient.
##
## The degrees of freedom for the null model are  15  and the objective function was  1.22 with Chi Squa
## The degrees of freedom for the model are 9  and the objective function was  0.11
##
## The root mean square of the residuals (RMSR) is  0.06
## The df corrected root mean square of the residuals is  0.07
##
## The harmonic number of observations is  565 with the empirical chi square  54.3  with prob <  1.7e-08
## The total number of observations was  596  with Likelihood Chi Square =  64.1  with prob <  2.2e-10
##
## Tucker Lewis Index of factoring reliability =  0.87
## RMSEA index =  0.101  and the 90 % confidence intervals are  0.079 0.126
## BIC =  6.59
## Fit based upon off diagonal values = 0.97
## Measures of factor score adequacy
##                                                    MR1
## Correlation of (regression) scores with factors   0.88
## Multiple R square of scores with factors          0.77
## Minimum correlation of possible factor scores     0.53
```

Graded-Response Model: Social Safety

```
##      Extrmt1 Extrmt2 Extrmt3 Extrmt4 Extrmt5 Extrmt6 Dscrmn
## Q43   -1.810  -1.321  -0.755  -0.245   0.331   1.174  1.696
## Q131  -1.750  -1.083  -0.506   0.137   0.933   2.160  1.487
## Q40   -2.348  -1.725  -1.027  -0.229   0.552   1.596  1.531
## Q132  -2.887  -1.746  -0.742   0.723   2.193   3.834  0.635
## Q62   -0.877  -0.261   0.177   0.537   1.053   1.717  1.701
## Q50   -2.199  -1.418  -0.927  -0.264   0.319   1.155  1.508
```

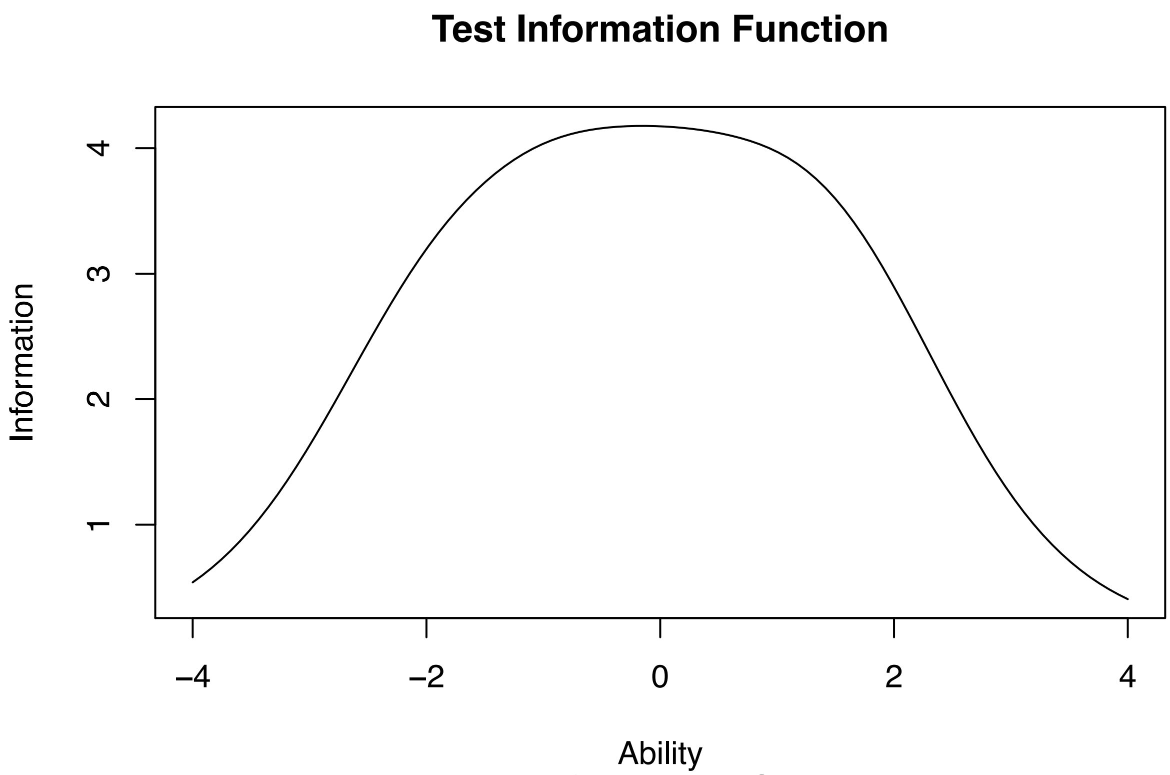

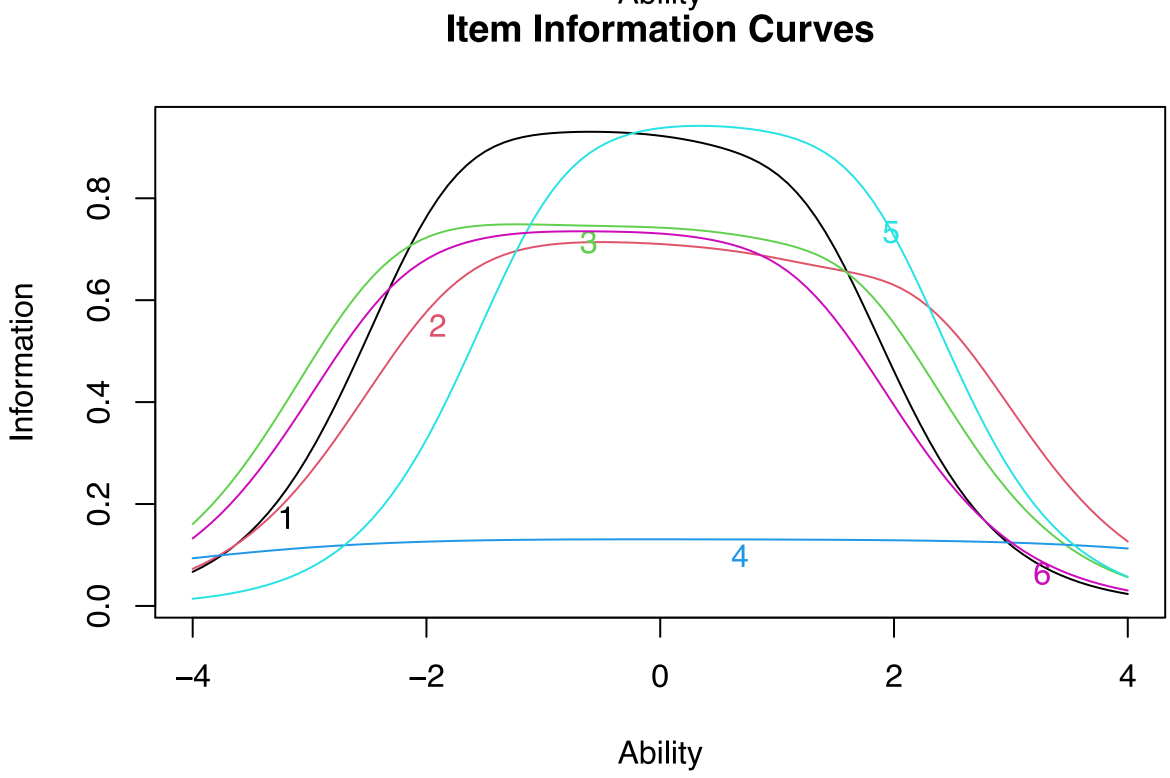

Back to top

Site DIF

```
## Call:
```

```
## lordif::lordif(resp.data = as.data.frame(merged_data), group = site)
##
##   Number of DIF groups: 2
##
##   Number of items flagged for DIF: 0 of 6
##
##   Items flagged:
##
##   Number of iterations for purification: 1 of 10
##
##   Detection criterion: Chisqr
##
##   Threshold: alpha = 0.01
```

Back to top

Gender-based DIF: Social Safety

```
## Call:
## lordif::lordif(resp.data = as.data.frame(sex.data), group = sex)
##
##   Number of DIF groups: 2
##
##   Number of items flagged for DIF: 4 of 6
##
##   Items flagged: 2, 4, 5, 6
##
##   Number of iterations for purification: 2 of 10
##
##   Detection criterion: Chisqr
##
##   Threshold: alpha = 0.01
##
##   item ncat  chi12  chi13  chi23
## 1    1    7 0.6586 0.8414 0.6985
## 2    2    7 0.0000 0.0000 0.9181
## 3    3    7 0.1196 0.2791 0.7193
## 4    4    7 0.0036 0.0138 0.7406
## 5    5    7 0.0006 0.0000 0.0034
## 6    6    7 0.0000 0.0000 0.9805
```

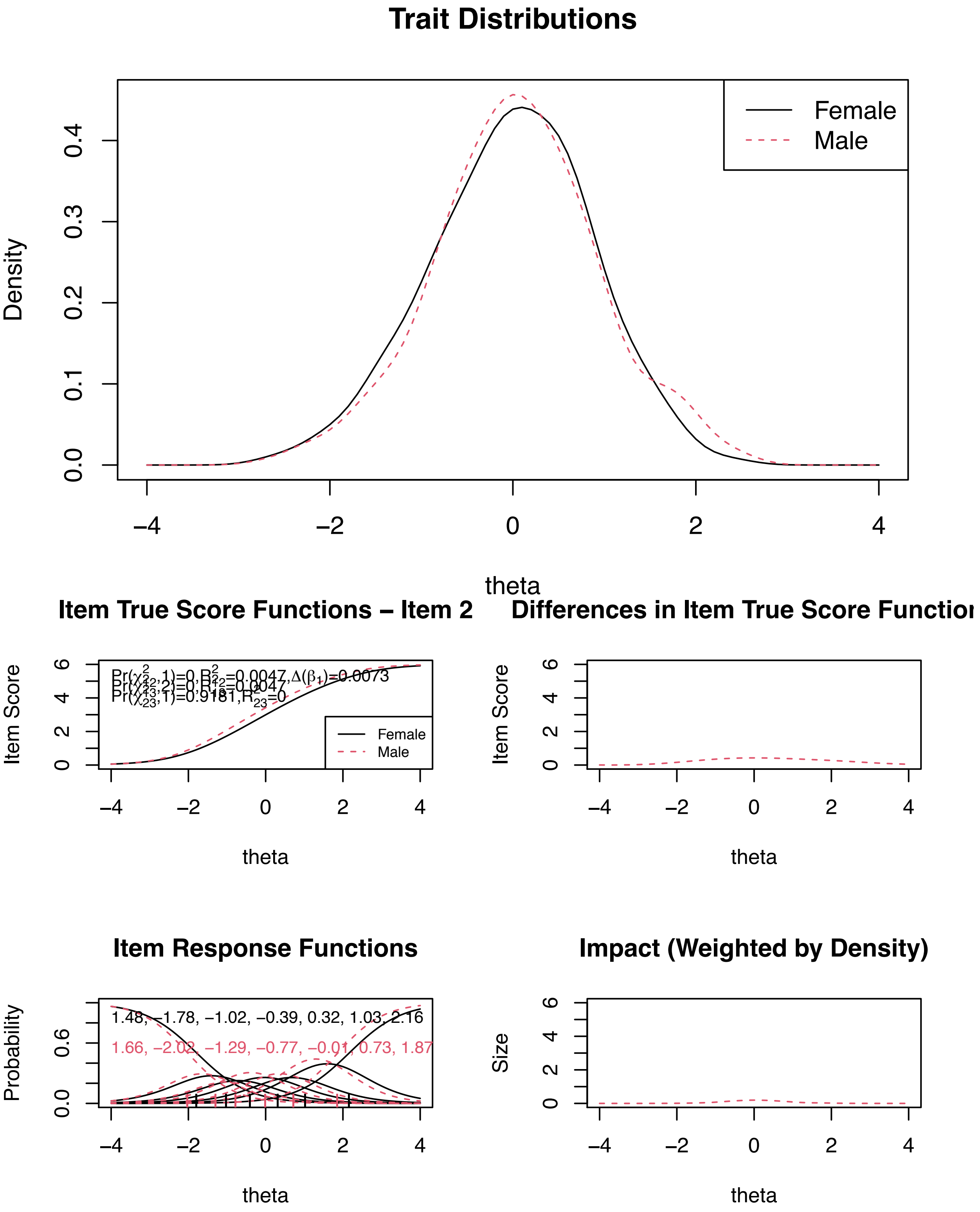
Trait Distributions
Density
theta
Female
Male
Item True Score Functions – Item 2
Item Score
Female
Male
theta
Differences in Item True Score Functio
Item Score
theta
Item Response Functions
Probability
1.48, −1.78, −1.02, −0.39, 0.32, 1.03, 2.16
1.66, −2.02, −1.29, −0.77, −0.01, 0.73, 1.87
theta
Impact (Weighted by Density)
Size
theta

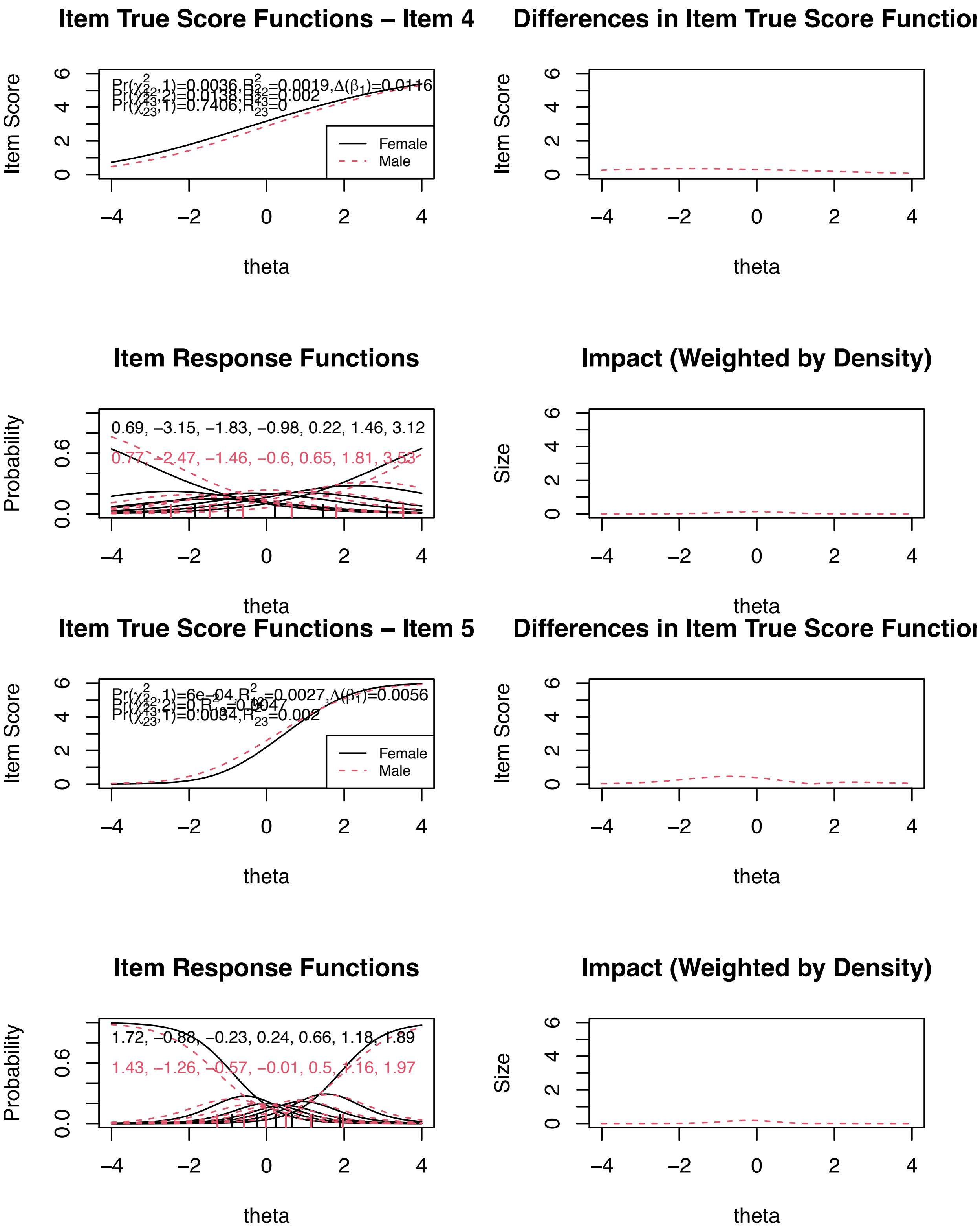
Item True Score Functions – Item 4
Differences in Item True Score Functio
Item Score
theta
Female
Male
Item Response Functions
Impact (Weighted by Density)
Probability
Size
0.69, –3.15, –1.83, –0.98, 0.22, 1.46, 3.12
0.77, –2.47, –1.46, –0.6, 0.65, 1.81, 3.53
Item True Score Functions – Item 5
1.72, –0.88, –0.23, 0.24, 0.66, 1.18, 1.89
1.43, –1.26, –0.57, –0.01, 0.5, 1.16, 1.97

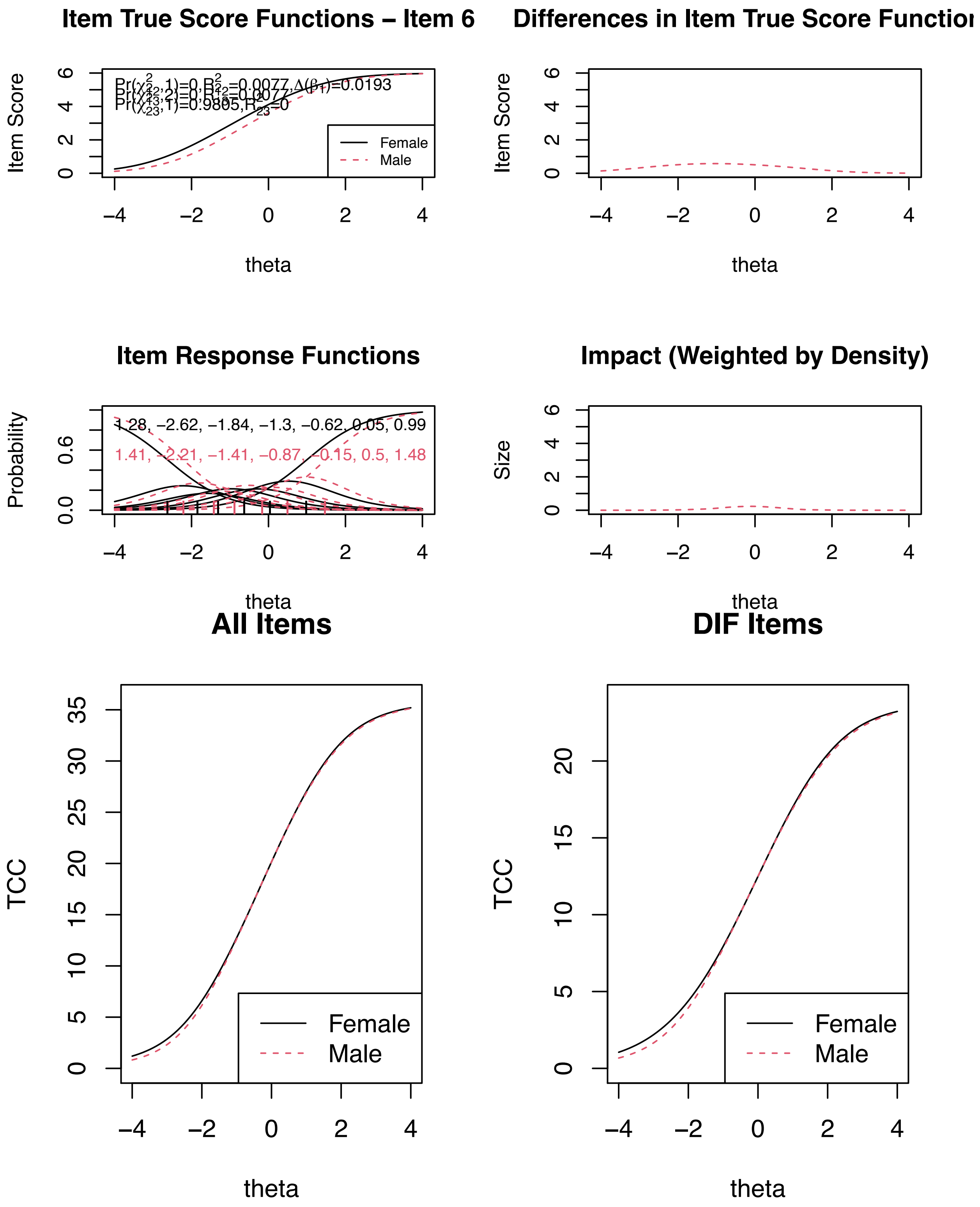

Item True Score Functions – Item 6
Differences in Item True Score Functio
Item Response Functions
Impact (Weighted by Density)
1.28, −2.62, −1.84, −1.3, −0.62, 0.05, 0.99
1.41, −2.21, −1.41, −0.87, −0.15, 0.5, 1.48
All Items
DIF Items
Female
Male
Item Score
Probability
Size
TCC
theta

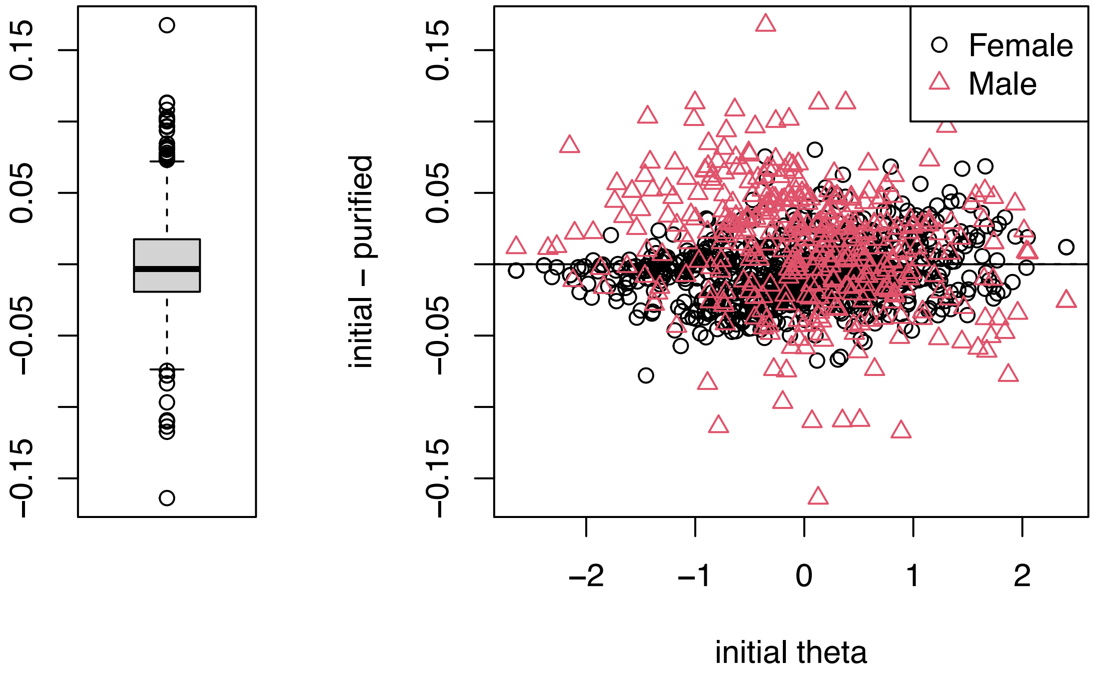

Age-based DIF: Social Safety

```
## Call:
## lordif::lordif(resp.data = as.data.frame(age.data), group = age)
##
##   Number of DIF groups: 2
##
##   Number of items flagged for DIF: 2 of 6
##
##   Items flagged: 3, 6
##
##   Number of iterations for purification: 2 of 10
##
##   Detection criterion: Chisqr
##
##   Threshold: alpha = 0.01
##
##   item ncat  chi12  chi13  chi23
## 1    1    7 0.8205 0.8712 0.6358
## 2    2    7 0.0112 0.0320 0.5006
## 3    3    7 0.0026 0.0097 0.6470
## 4    4    7 0.3093 0.3900 0.3567
## 5    5    7 0.1872 0.3098 0.4369
## 6    6    7 0.0004 0.0005 0.1158
```

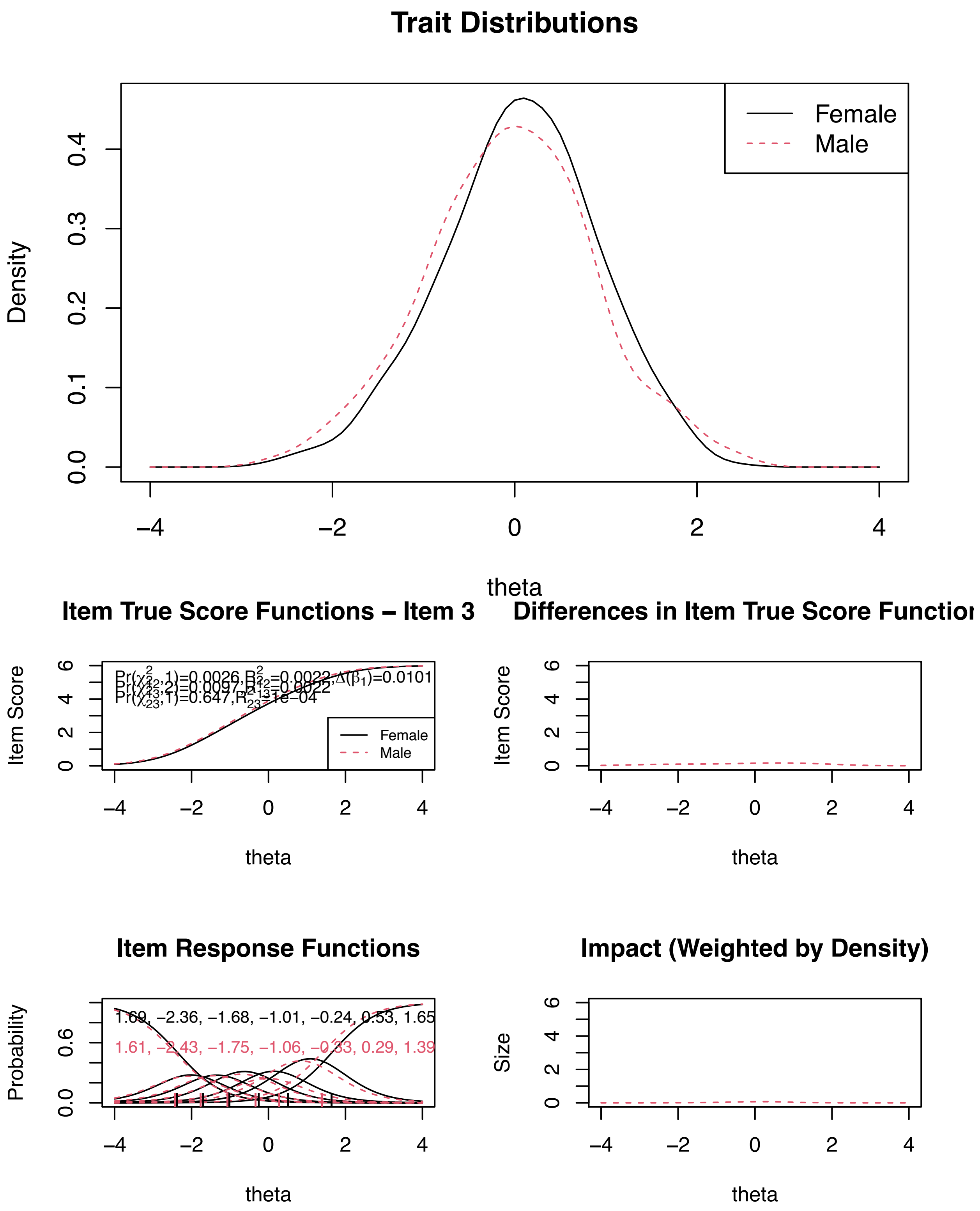
Trait Distributions
Density
theta
Female
Male
Item True Score Functions – Item 3
Item Score
Female
Male
theta
Differences in Item True Score Functio
Item Score
theta
Item Response Functions
Probability
1.69, −2.36, −1.68, −1.01, −0.24, 0.53, 1.65
1.61, −2.43, −1.75, −1.06, −0.33, 0.29, 1.39
theta
Impact (Weighted by Density)
Size
theta

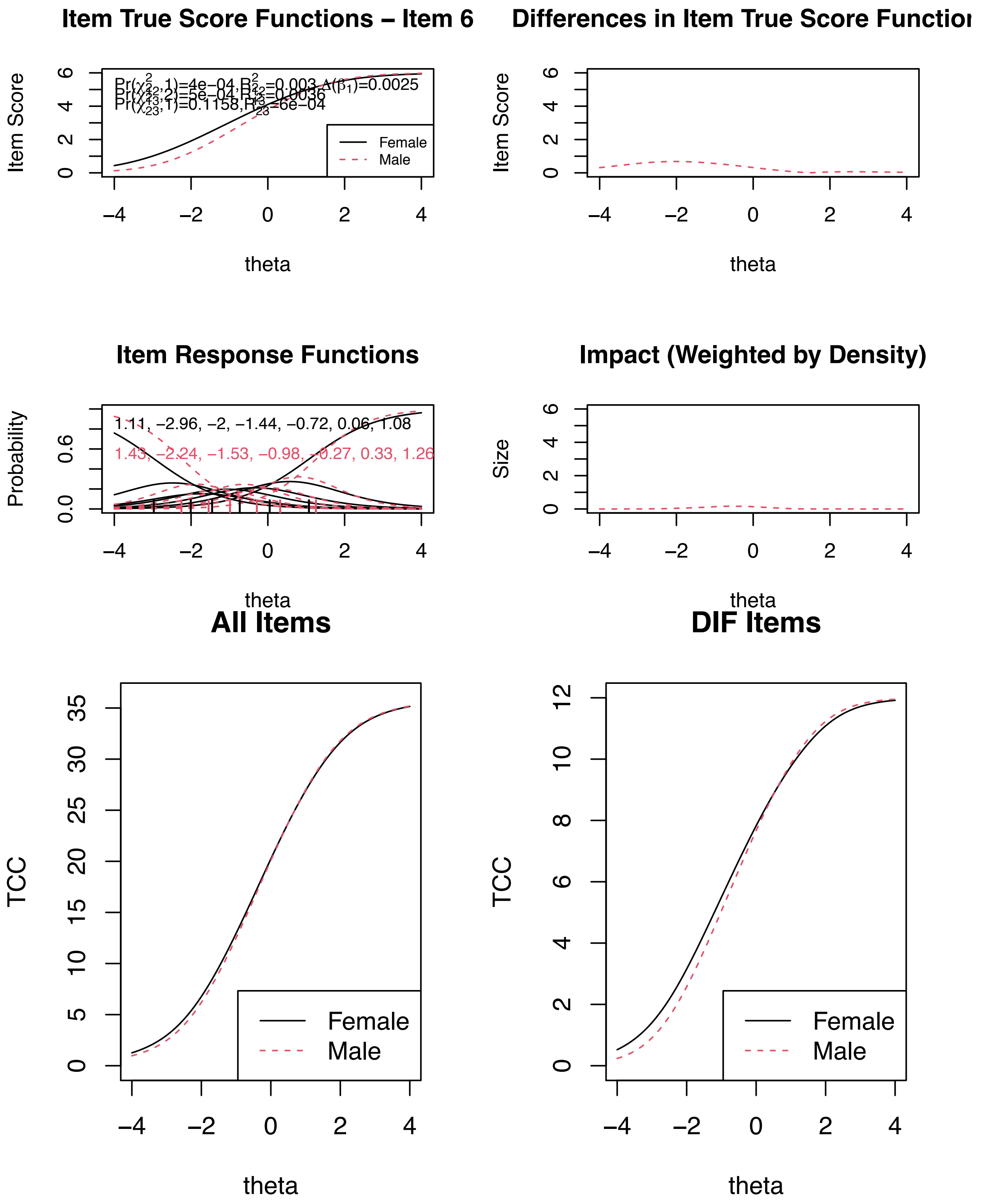
Item True Score Functions – Item 6
Pr(χ²12,1)=4e−04,R²12=0.003,Δ(β1)=0.0025
Pr(χ²13,2)=5e−04,R²13=0.0036
Pr(χ²23,1)=0.1158,R²23=6e−04
Female
Male
Item Score
theta
Differences in Item True Score Functio
Item Score
theta
Item Response Functions
1.11, −2.96, −2, −1.44, −0.72, 0.06, 1.08
1.43, −2.24, −1.53, −0.98, −0.27, 0.33, 1.26
Probability
theta
Impact (Weighted by Density)
Size
theta
All Items
TCC
Female
Male
theta
DIF Items
TCC
Female
Male
theta

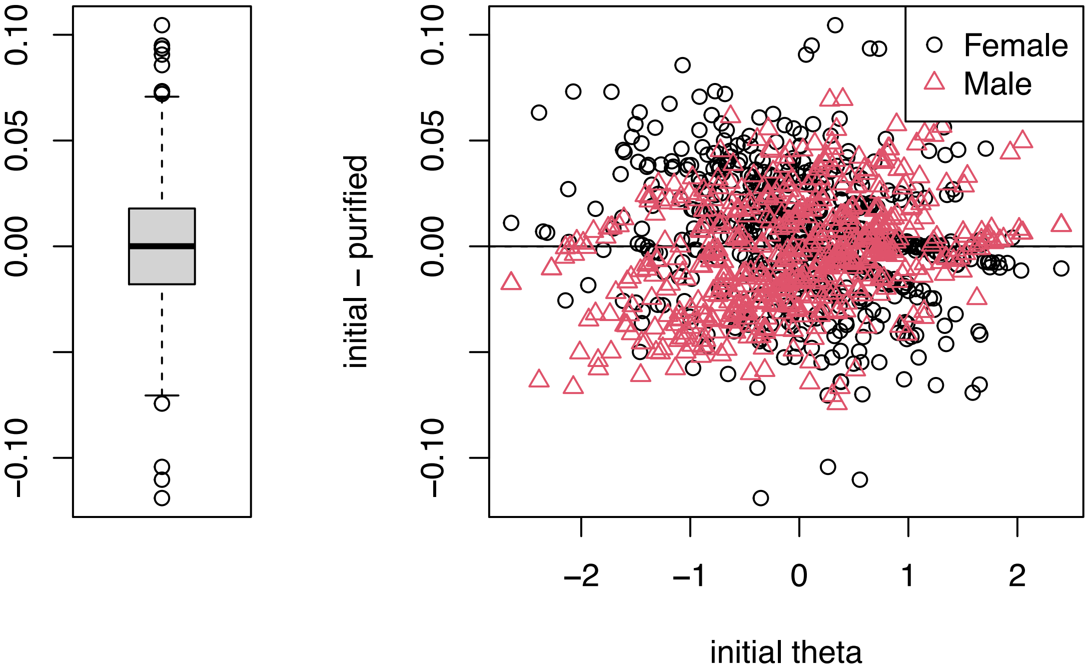

Back to top

Need for Control

Site 1

Reliability: Need for Control

```
## Cronbach's alpha is 0.624.
## Mean item-total correlation is 0.293.
## If each item were dropped:
##      raw_alpha std.alpha G6(smc) average_r S/N alpha se  var.r med.r
## Q26       0.50      0.51    0.42      0.26 1.0    0.035 0.0075  0.22
## Q20       0.54      0.54    0.45      0.28 1.2    0.032 0.0040  0.26
## Q68       0.58      0.57    0.50      0.31 1.3    0.029 0.0249  0.24
## Q130      0.59      0.59    0.51      0.32 1.4    0.029 0.0210  0.26
```

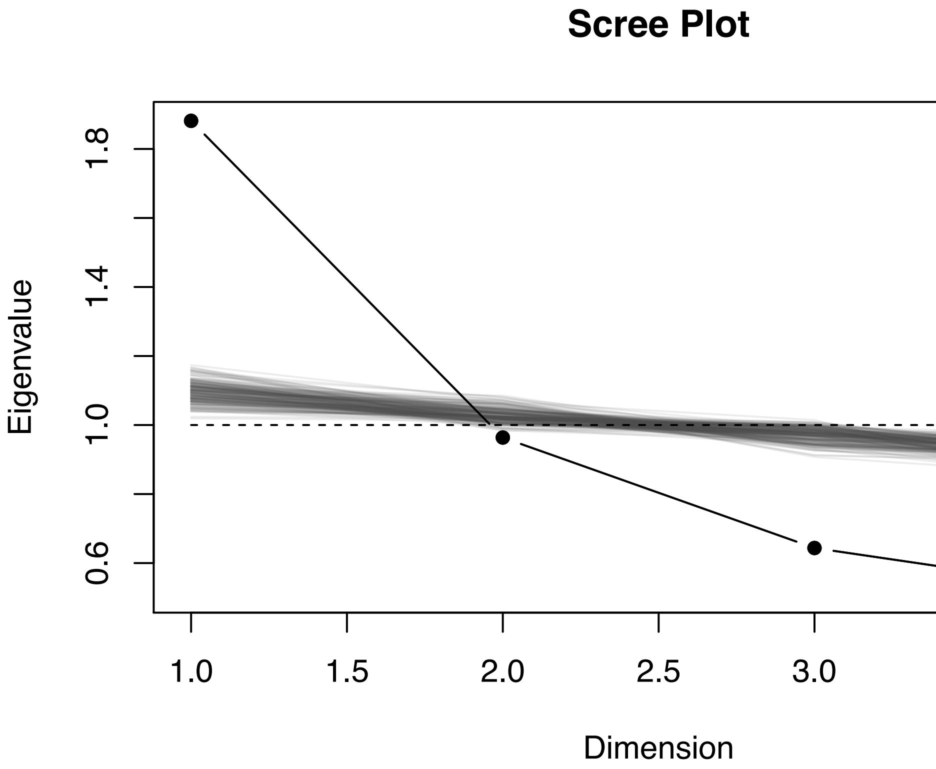

Unidimensionality: Need for Control

```
## [1] "Ratio of first to second eigenvalues: 1.952"

## [1] 1.8814133 0.9637693 0.6436392 0.5111782

## Factor Analysis using method =  minres
## Call: fa(r = grm_obj$X)
## Standardized loadings (pattern matrix) based upon correlation matrix
##       MR1   h2   u2 com
## Q26  0.68 0.46 0.54   1
## Q20  0.61 0.37 0.63   1
## Q68  0.46 0.21 0.79   1
## Q130 0.43 0.18 0.82   1
##
##                 MR1
## SS loadings    1.22
## Proportion Var 0.30
##
## Mean item complexity =  1
## Test of the hypothesis that 1 factor is sufficient.
##
## The degrees of freedom for the null model are  6  and the objective function was  0.52 with Chi Squa
## The degrees of freedom for the model are 2  and the objective function was  0.08
##
## The root mean square of the residuals (RMSR) is  0.09
## The df corrected root mean square of the residuals is  0.15
##
## The harmonic number of observations is  573 with the empirical chi square  49.77  with prob <  1.6e-
## The total number of observations was  617  with Likelihood Chi Square =  46.68  with prob <  7.3e-11
##
## Tucker Lewis Index of factoring reliability =  0.569
## RMSEA index =  0.19  and the 90 % confidence intervals are  0.145 0.24
```

```
## BIC =  33.83
## Fit based upon off diagonal values = 0.92
## Measures of factor score adequacy
##                                                    MR1
## Correlation of (regression) scores with factors   0.81
## Multiple R square of scores with factors          0.66
## Minimum correlation of possible factor scores     0.31
```

Graded-Response Model: Need for Control

```
##      Extrmt1 Extrmt2 Extrmt3 Extrmt4 Extrmt5 Extrmt6 Dscrmn
## Q26   -1.335  -0.663  -0.269   0.327   0.877   1.529  1.901
## Q20   -1.302  -0.606  -0.175   0.234   0.771   1.376  1.596
## Q68   -2.806  -1.824  -1.226  -0.396   0.399   1.313  0.933
## Q130  -1.633  -0.217   0.611   1.633   2.691   3.668  0.796
```

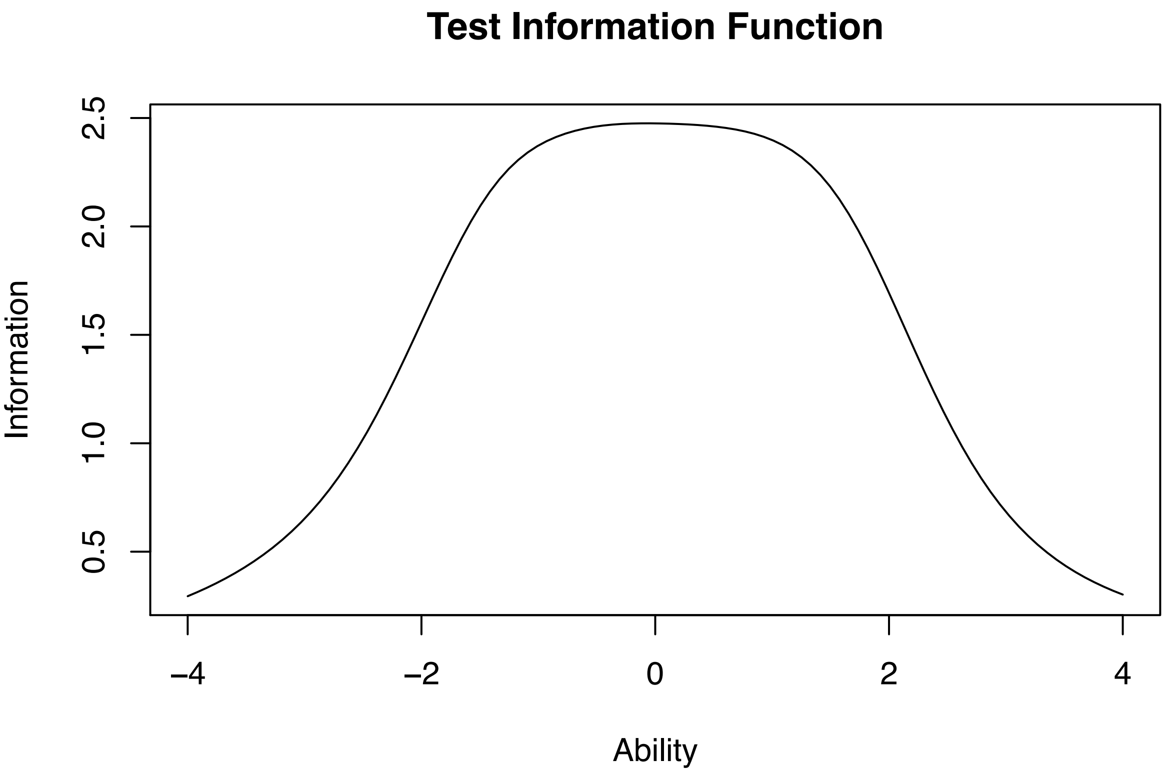

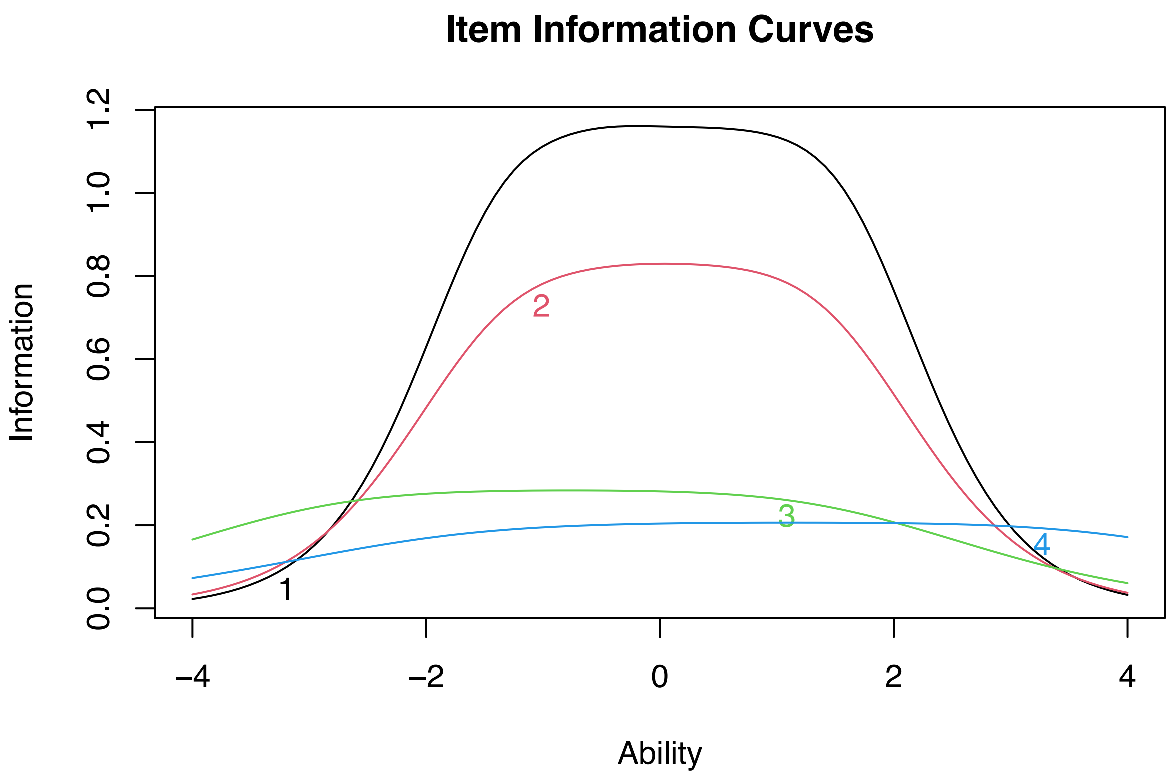

Back to top

Site 2

Reliability: Need for Control

```
## Cronbach's alpha is 0.674.
## Mean item-total correlation is 0.341.
## If each item were dropped:
##      raw_alpha std.alpha G6(smc) average_r S/N alpha se   var.r med.r
## Q26       0.59      0.59    0.50      0.33 1.5    0.029 0.00087  0.34
## Q20       0.57      0.57    0.47      0.31 1.3    0.031 0.00164  0.31
## Q68       0.65      0.65    0.56      0.38 1.9    0.024 0.00855  0.35
## Q130      0.62      0.62    0.53      0.35 1.6    0.027 0.01469  0.29
```

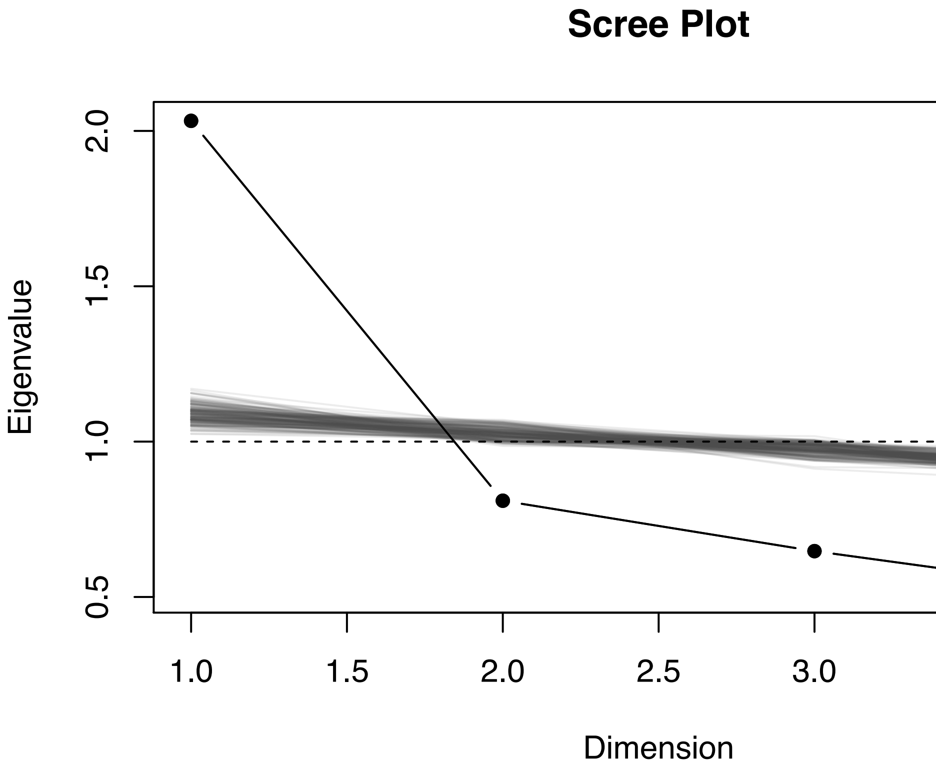

Unidimensionality: Need for Control

```
## [1] "Ratio of first to second eigenvalues: 2.51"

## [1] 2.0323792 0.8097225 0.6475203 0.5103781

## Factor Analysis using method =  minres
## Call: fa(r = grm_obj$X)
## Standardized loadings (pattern matrix) based upon correlation matrix
##       MR1   h2   u2 com
## Q26  0.63 0.40 0.60   1
## Q20  0.69 0.48 0.52   1
## Q68  0.47 0.22 0.78   1
## Q130 0.55 0.30 0.70   1
##
##                 MR1
## SS loadings    1.40
## Proportion Var 0.35
##
## Mean item complexity =  1
## Test of the hypothesis that 1 factor is sufficient.
##
## The degrees of freedom for the null model are  6  and the objective function was  0.61 with Chi Squa
## The degrees of freedom for the model are 2  and the objective function was  0.03
##
## The root mean square of the residuals (RMSR) is  0.05
## The df corrected root mean square of the residuals is  0.08
##
## The harmonic number of observations is  554 with the empirical chi square  14.81  with prob <  0.000
## The total number of observations was  596  with Likelihood Chi Square =  16.92  with prob <  0.00021
##
## Tucker Lewis Index of factoring reliability =  0.874
## RMSEA index =  0.112  and the 90 % confidence intervals are  0.067 0.164
```

```
## BIC =  4.14
## Fit based upon off diagonal values = 0.98
## Measures of factor score adequacy
##                                                    MR1
## Correlation of (regression) scores with factors   0.83
## Multiple R square of scores with factors          0.70
## Minimum correlation of possible factor scores     0.39
```

Graded-Response Model: Need for Control

```
##      Extrmt1 Extrmt2 Extrmt3 Extrmt4 Extrmt5 Extrmt6 Dscrmn
## Q26   -0.901  -0.194   0.277   0.758   1.262   1.803  1.732
## Q20   -0.983  -0.325   0.094   0.504   1.004   1.632  1.936
## Q68   -2.187  -1.267  -0.637  -0.026   0.742   1.421  1.060
## Q130  -0.798   0.190   0.716   1.367   2.045   2.695  1.240
```

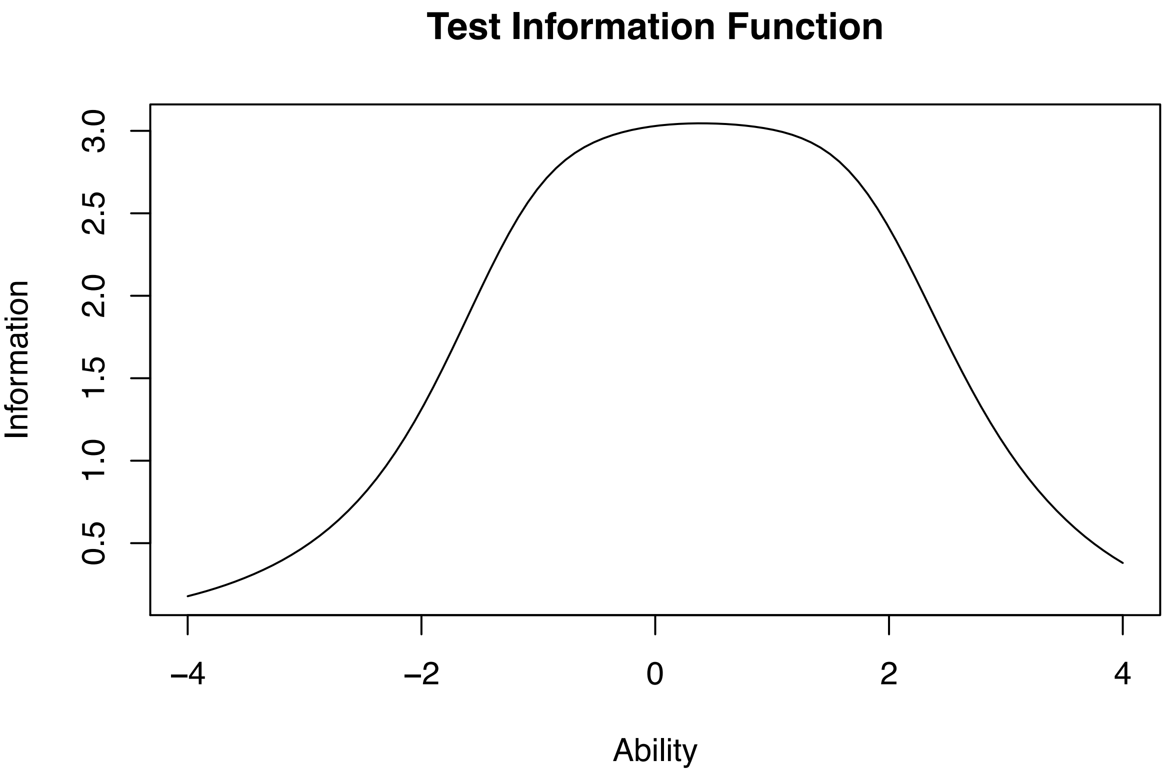

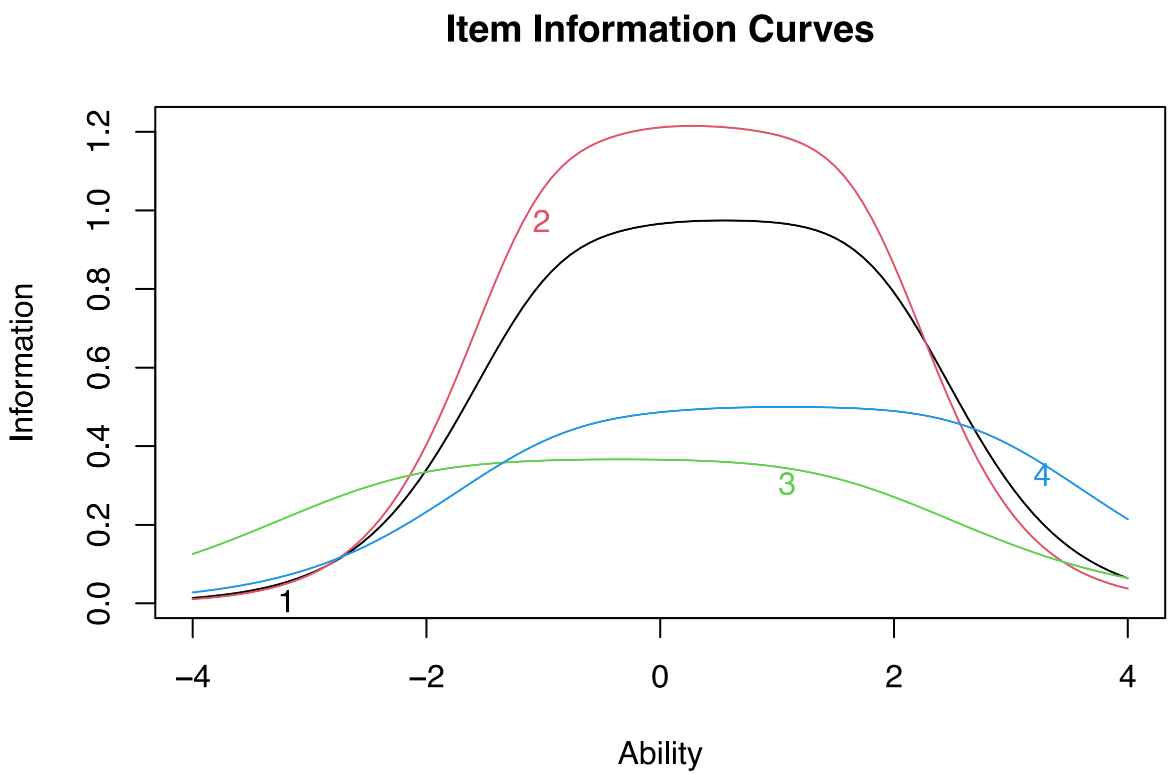

Back to top

Site DIF

```
## Call:
## lordif::lordif(resp.data = as.data.frame(merged_data), group = site)
##
##   Number of DIF groups: 2
##
##   Number of items flagged for DIF: 0 of 4
##
##   Items flagged:
##
##   Number of iterations for purification: 1 of 10
##
##   Detection criterion: Chisqr
##
##   Threshold: alpha = 0.01
```

Back to top

Gender-based DIF: Need for Control

```
## Call:
## lordif::lordif(resp.data = as.data.frame(sex.data), group = sex)
##
##   Number of DIF groups: 2
##
##   Number of items flagged for DIF: 2 of 4
```

```
##
##   Items flagged: 3, 4
##
##   Number of iterations for purification: 5 of 10
##
##   Detection criterion: Chisqr
##
##   Threshold: alpha = 0.01
##
##   item ncat  chi12  chi13  chi23
## 1    1    7 0.5272 0.5910 0.4194
## 2    2    7 0.3421 0.2306 0.1541
## 3    3    7 0.0000 0.0000 0.0700
## 4    4    7 0.0001 0.0006 0.9197
```

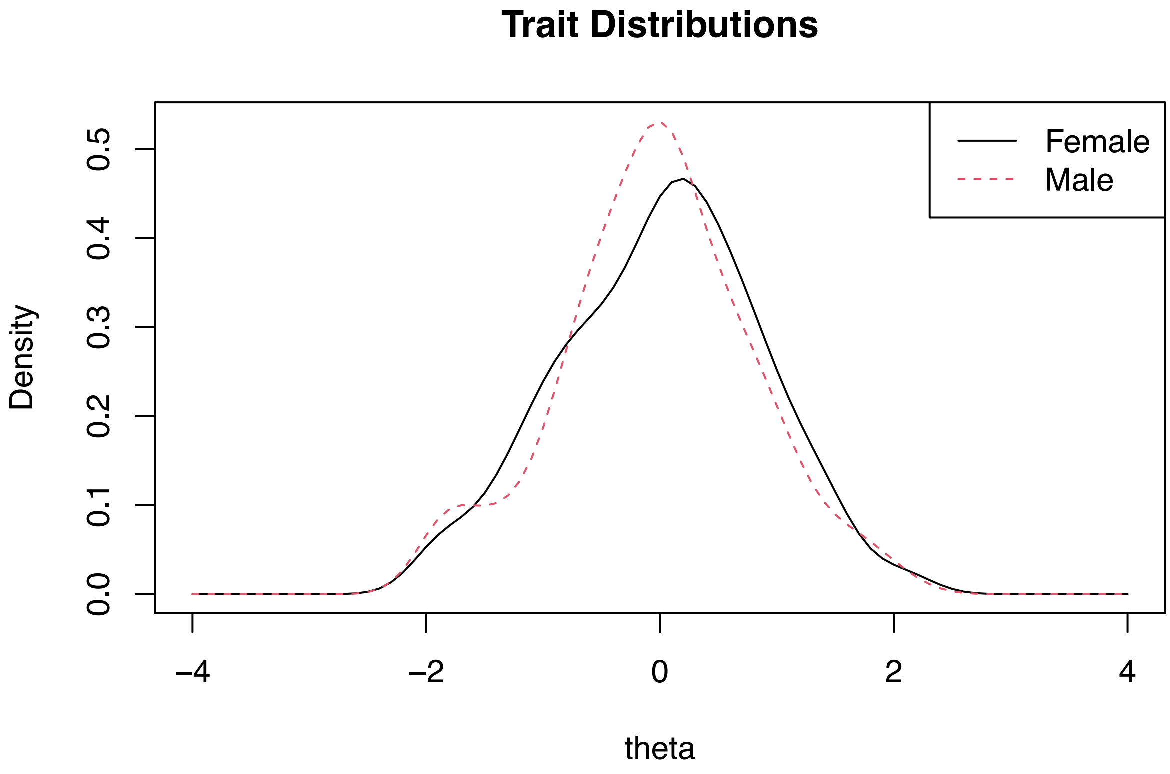

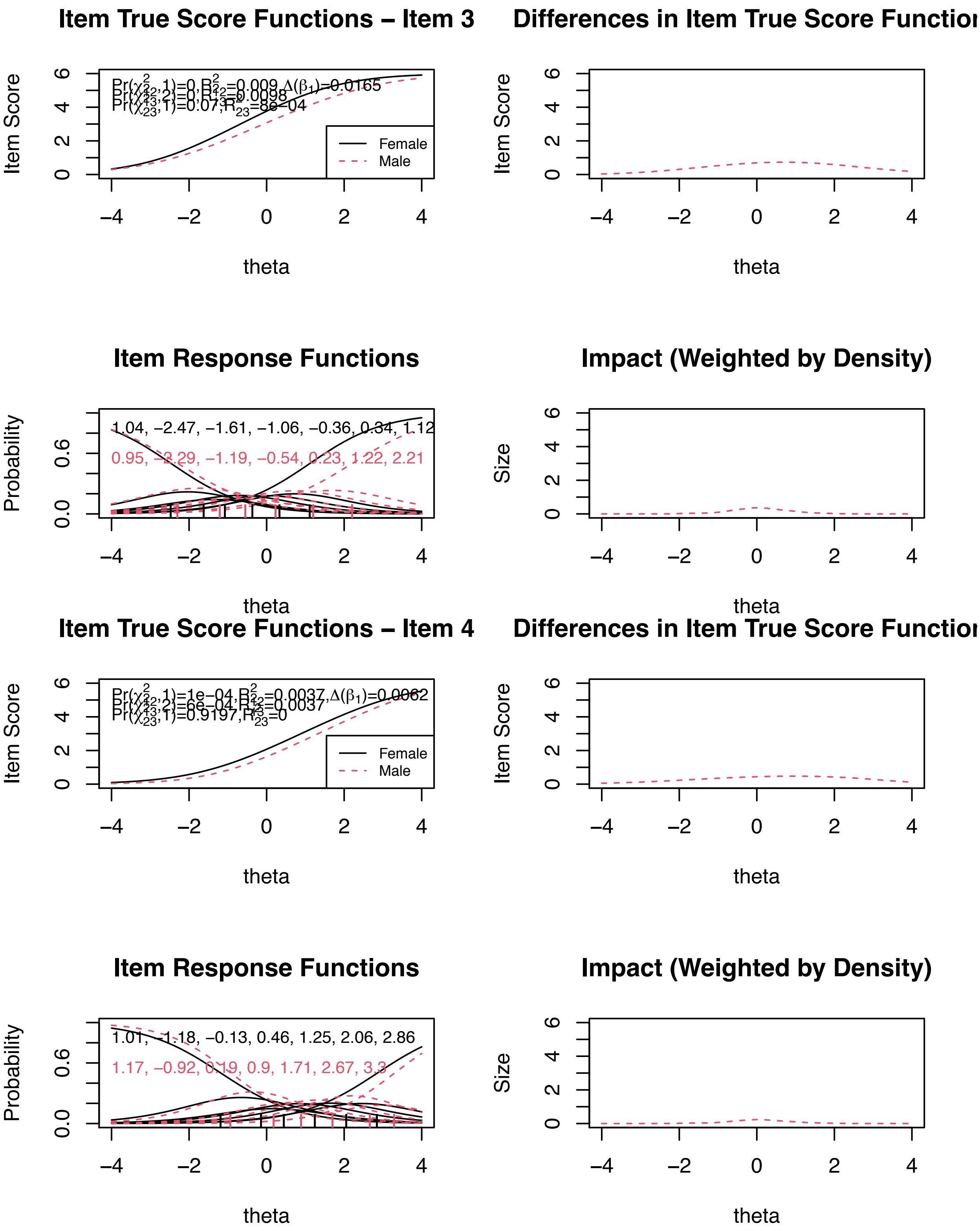
Item True Score Functions – Item 3
Differences in Item True Score Functio
Item Score
theta
Female
Male
Item Response Functions
Impact (Weighted by Density)
Probability
Size
1.04, −2.47, −1.61, −1.06, −0.36, 0.34, 1.12
0.95, −2.29, −1.19, −0.54, 0.23, 1.22, 2.21
Item True Score Functions – Item 4
1.01, −1.18, −0.13, 0.46, 1.25, 2.06, 2.86
1.17, −0.92, 0.19, 0.9, 1.71, 2.67, 3.3

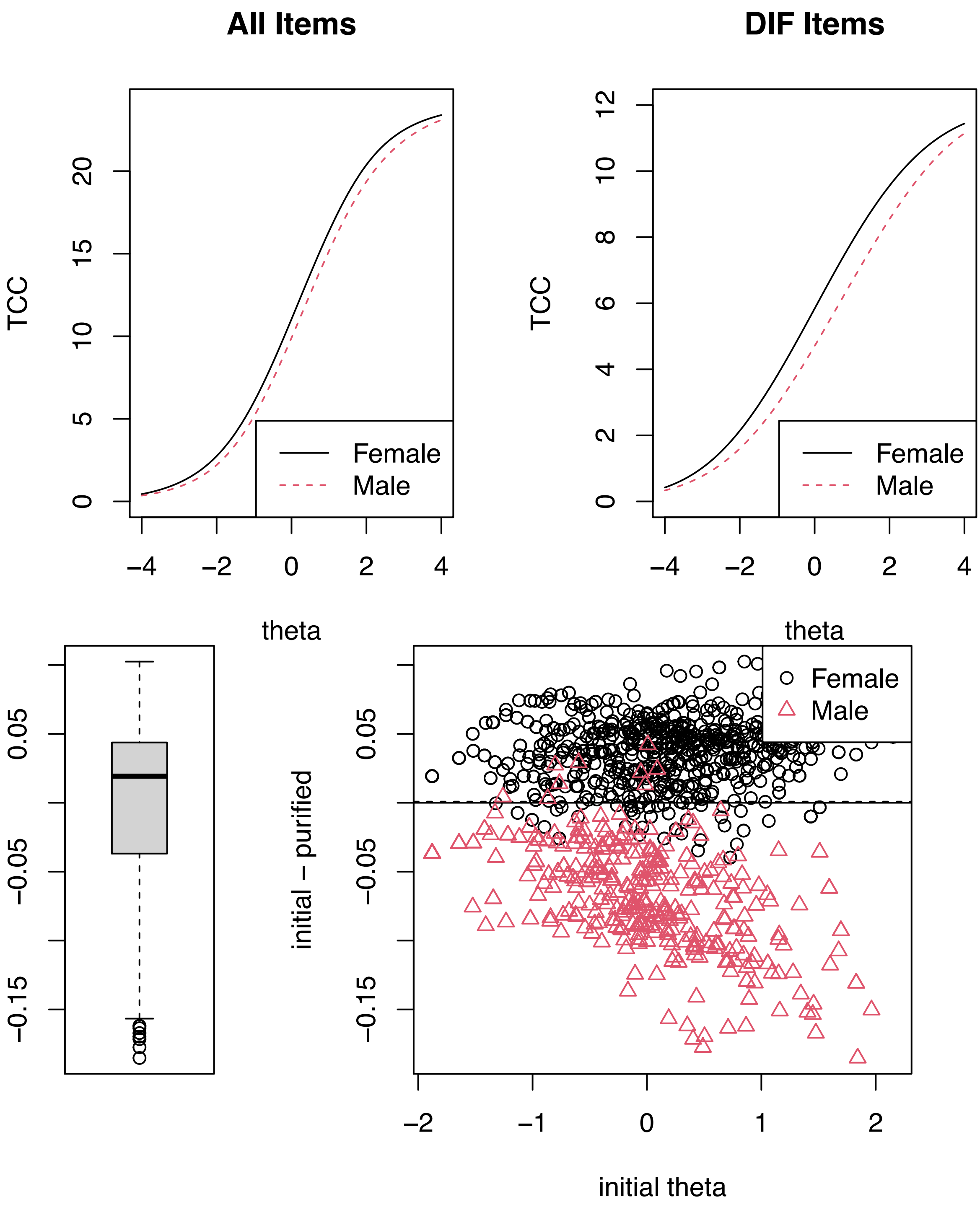

Age-based DIF: Need for Control

```
## Call:
## lordif::lordif(resp.data = as.data.frame(age.data), group = age)
##
##   Number of DIF groups: 2
##
```

```
##   Number of items flagged for DIF: 1 of 4
##
##   Items flagged: 2
##
##   Number of iterations for purification: 2 of 10
##
##   Detection criterion: Chisqr
##
##   Threshold: alpha = 0.01
##
##   item ncat  chi12  chi13  chi23
## 1    1    7 0.0846 0.1100 0.2301
## 2    2    7 0.0000 0.0000 0.0122
## 3    3    7 0.6912 0.8055 0.6001
## 4    4    7 0.7262 0.9398 0.9686
```

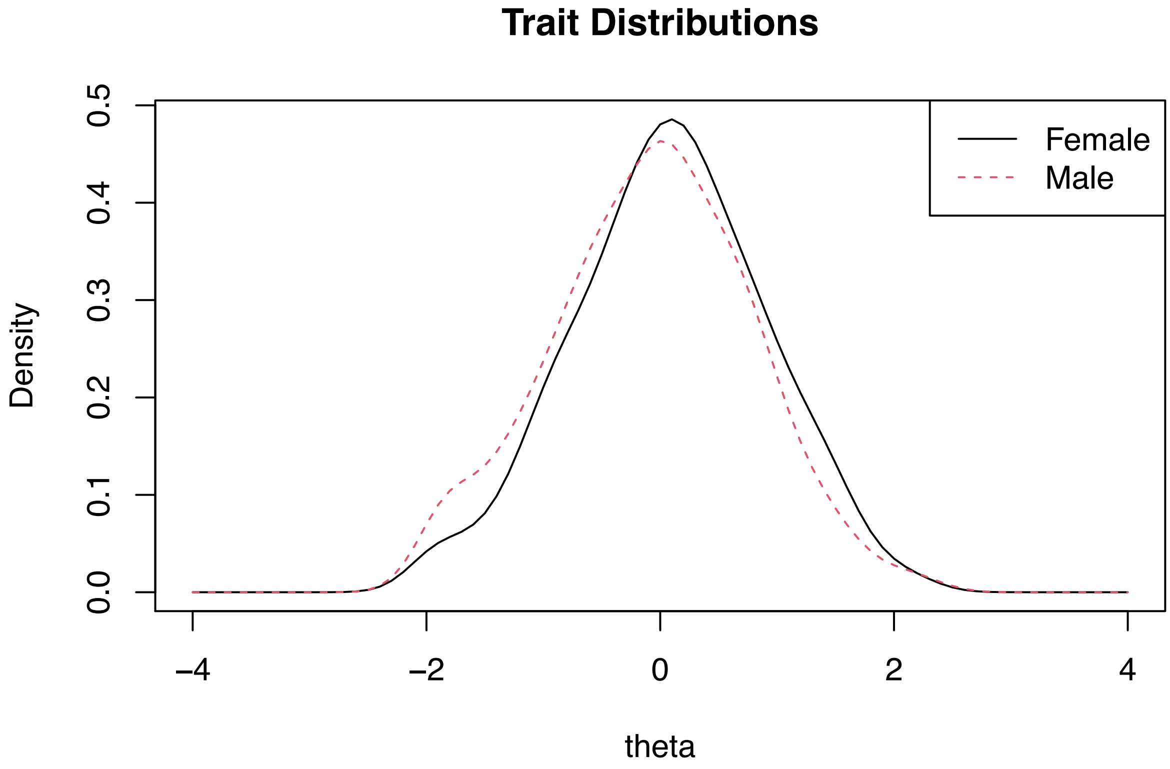

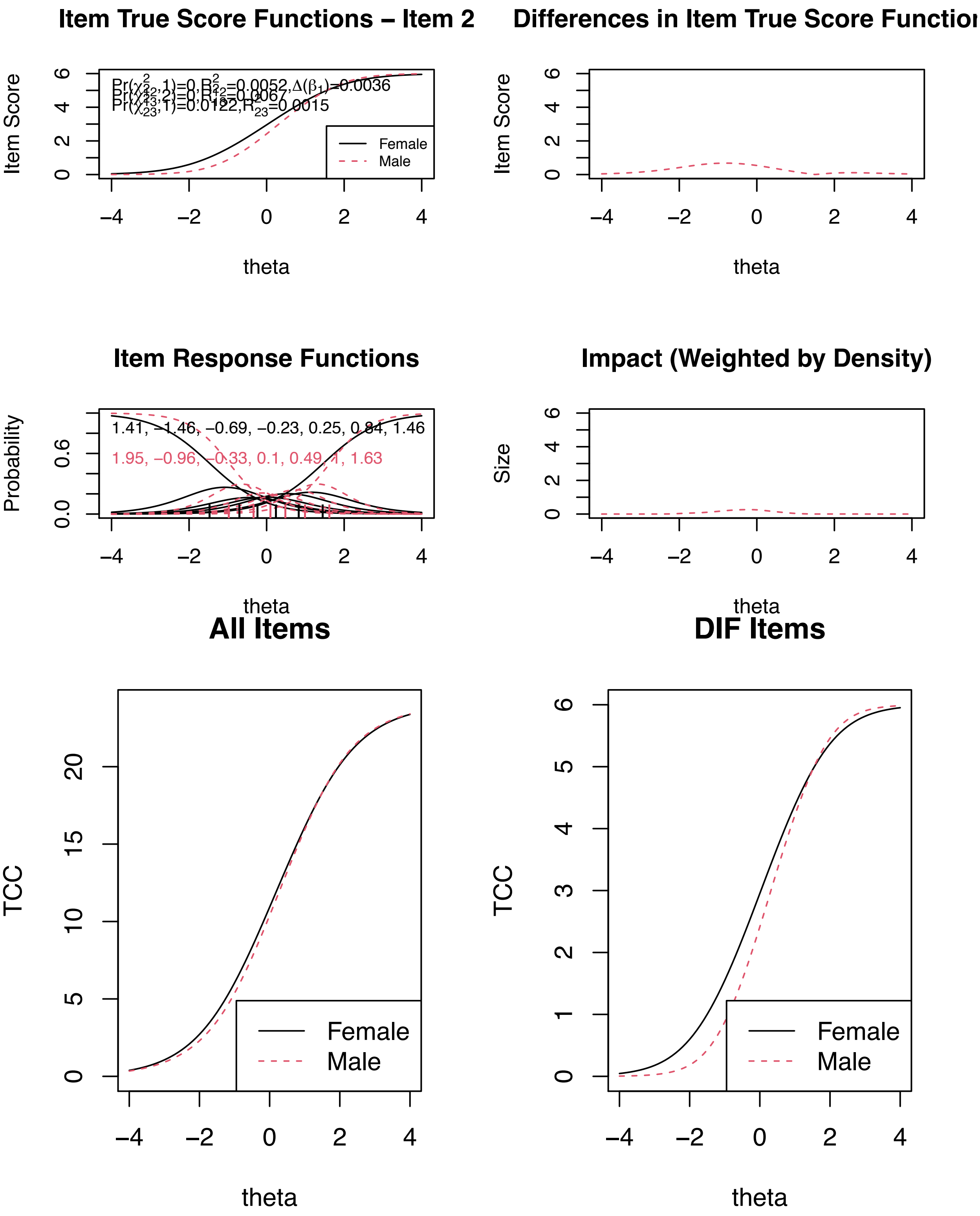

Item True Score Functions – Item 2
Differences in Item True Score Functio
Item Score
theta
Female
Male
Item Response Functions
Impact (Weighted by Density)
Probability
Size
1.41, −1.46, −0.69, −0.23, 0.25, 0.84, 1.46
1.95, −0.96, −0.33, 0.1, 0.49, 1, 1.63
All Items
DIF Items
TCC

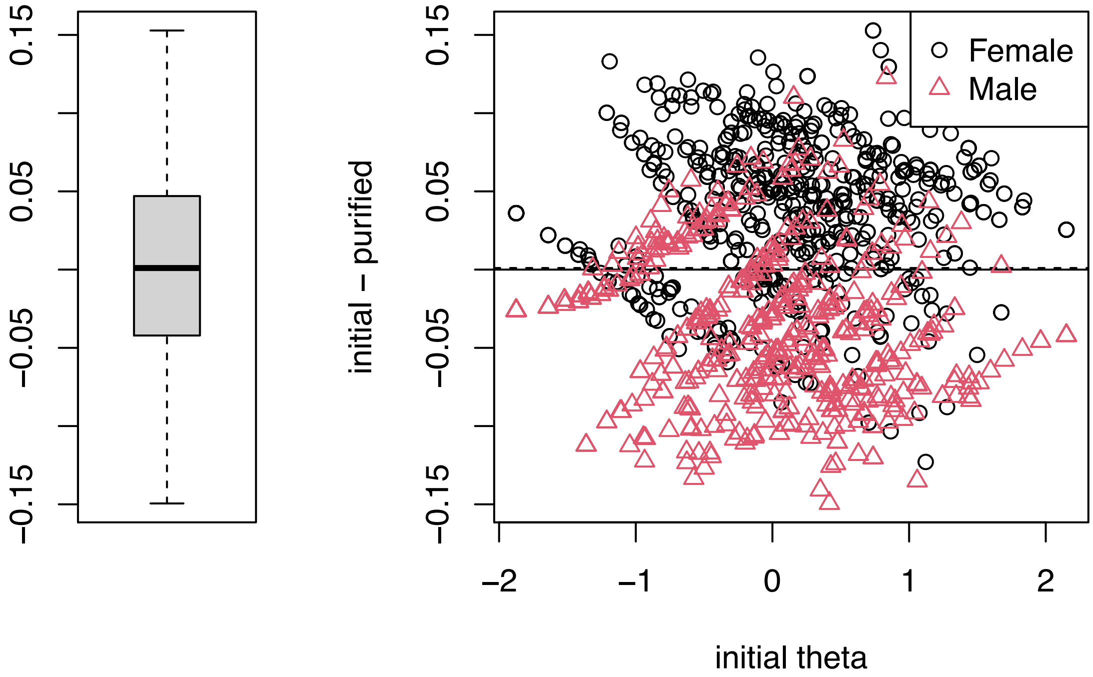

Back to top

Hopelessness

Site 1

Reliability: Hopelessness

```
## Cronbach's alpha is 0.807.
## Mean item-total correlation is 0.448.
## If each item were dropped:
##      raw_alpha std.alpha G6(smc) average_r S/N alpha se  var.r med.r
## Q15       0.84      0.84    0.80      0.56 5.2    0.011 0.0019  0.58
## Q61       0.74      0.73    0.71      0.40 2.7    0.017 0.0430  0.42
## Q115      0.74      0.73    0.71      0.40 2.7    0.017 0.0299  0.46
## Q24       0.75      0.75    0.73      0.42 2.9    0.016 0.0321  0.46
## Q88       0.77      0.76    0.74      0.45 3.2    0.015 0.0256  0.49
```

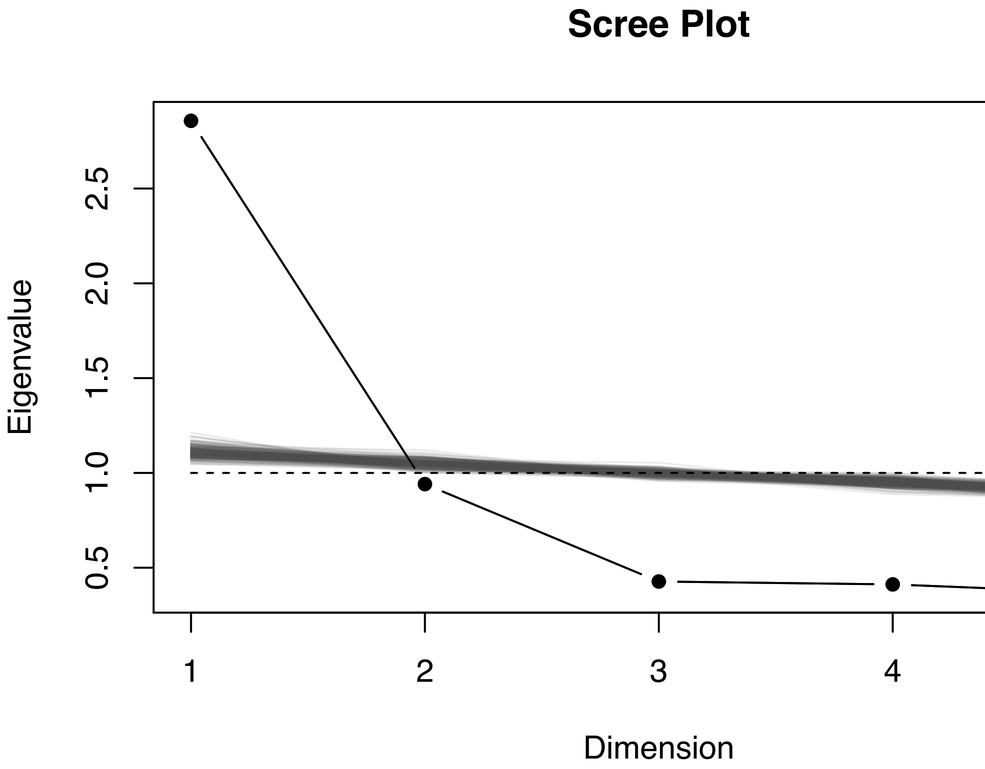

Unidimensionality: Hopelessness

```
## [1] "Ratio of first to second eigenvalues: 3.036"
```

```
## [1] 2.8569948 0.9409413 0.4273236 0.4119507 0.3627897
```

```
## Factor Analysis using method =  minres
## Call: fa(r = grm_obj$X)
## Standardized loadings (pattern matrix) based upon correlation matrix
##       MR1   h2   u2 com
## Q15  0.37 0.14 0.86   1
## Q61  0.76 0.57 0.43   1
## Q115 0.81 0.65 0.35   1
## Q24  0.75 0.56 0.44   1
## Q88  0.69 0.48 0.52   1
##
##                 MR1
## SS loadings    2.40
## Proportion Var 0.48
##
## Mean item complexity =  1
## Test of the hypothesis that 1 factor is sufficient.
##
## The degrees of freedom for the null model are  10  and the objective function was  1.76 with Chi Squa
## The degrees of freedom for the model are 5  and the objective function was  0.13
##
## The root mean square of the residuals (RMSR) is  0.07
## The df corrected root mean square of the residuals is  0.1
##
## The harmonic number of observations is  589 with the empirical chi square  55.26  with prob <  1.2e-
## The total number of observations was  617  with Likelihood Chi Square =  76.62  with prob <  4.3e-15
##
## Tucker Lewis Index of factoring reliability =  0.866
```

```
## RMSEA index =  0.152  and the 90 % confidence intervals are  0.123 0.184
## BIC =  44.49
## Fit based upon off diagonal values = 0.98
## Measures of factor score adequacy
##                                                     MR1
## Correlation of (regression) scores with factors   0.92
## Multiple R square of scores with factors          0.85
## Minimum correlation of possible factor scores     0.70
```

Graded-Response Model: Hopelessness

```
##      Extrmt1 Extrmt2 Extrmt3 Extrmt4 Extrmt5 Extrmt6 Dscrmn
## Q15   -1.537  -0.372   0.800   2.030   3.133   4.623  0.783
## Q61   -0.866  -0.287   0.213   0.776   1.257   1.829  2.336
## Q115  -1.480  -0.881  -0.387   0.139   0.689   1.276  2.604
## Q24   -1.544  -0.793  -0.394   0.130   0.641   1.333  2.080
## Q88   -2.100  -1.563  -1.117  -0.638  -0.110   0.542  1.935
```

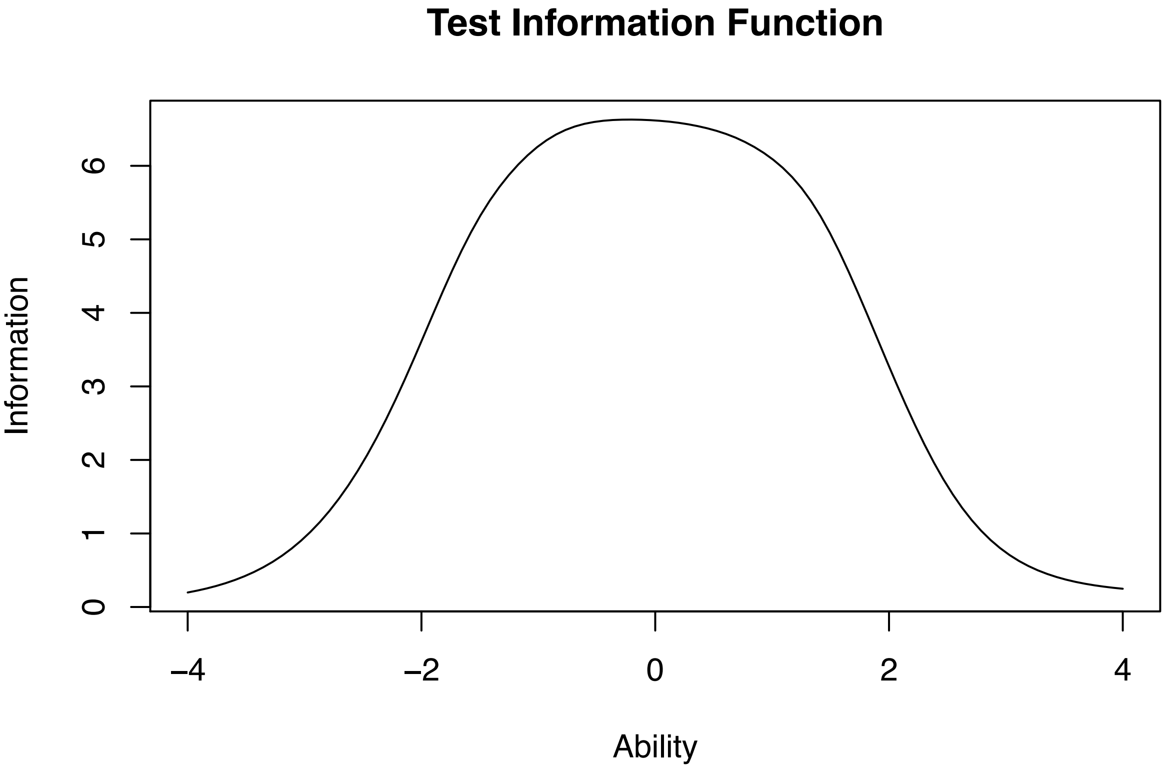

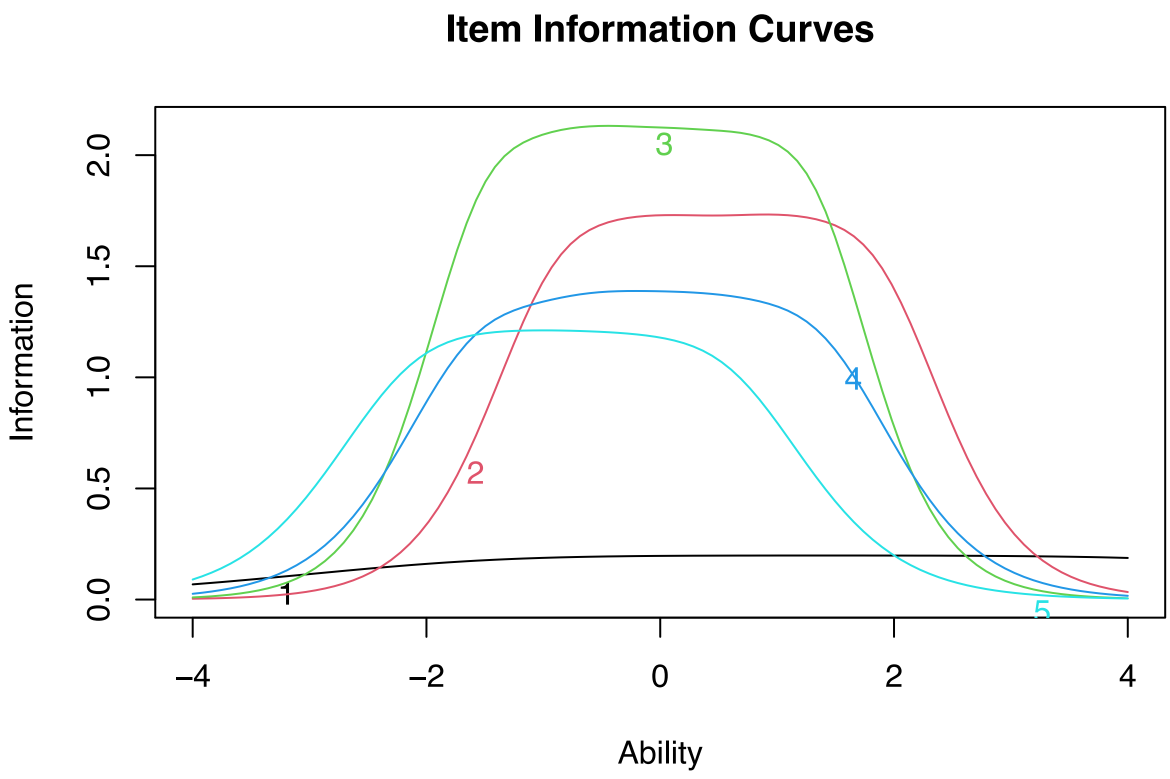

Back to top

Site 2

Reliability: Hopelessness

```
## Cronbach's alpha is 0.812.
## Mean item-total correlation is 0.46.
## If each item were dropped:
##      raw_alpha std.alpha G6(smc) average_r S/N alpha se  var.r med.r
## Q15       0.83      0.83    0.79      0.55 4.9    0.011 0.0035  0.56
## Q61       0.76      0.76    0.73      0.44 3.1    0.015 0.0308  0.44
## Q115      0.74      0.74    0.71      0.42 2.9    0.017 0.0178  0.46
## Q24       0.76      0.75    0.73      0.43 3.1    0.016 0.0208  0.46
## Q88       0.78      0.77    0.74      0.46 3.4    0.015 0.0155  0.49
```

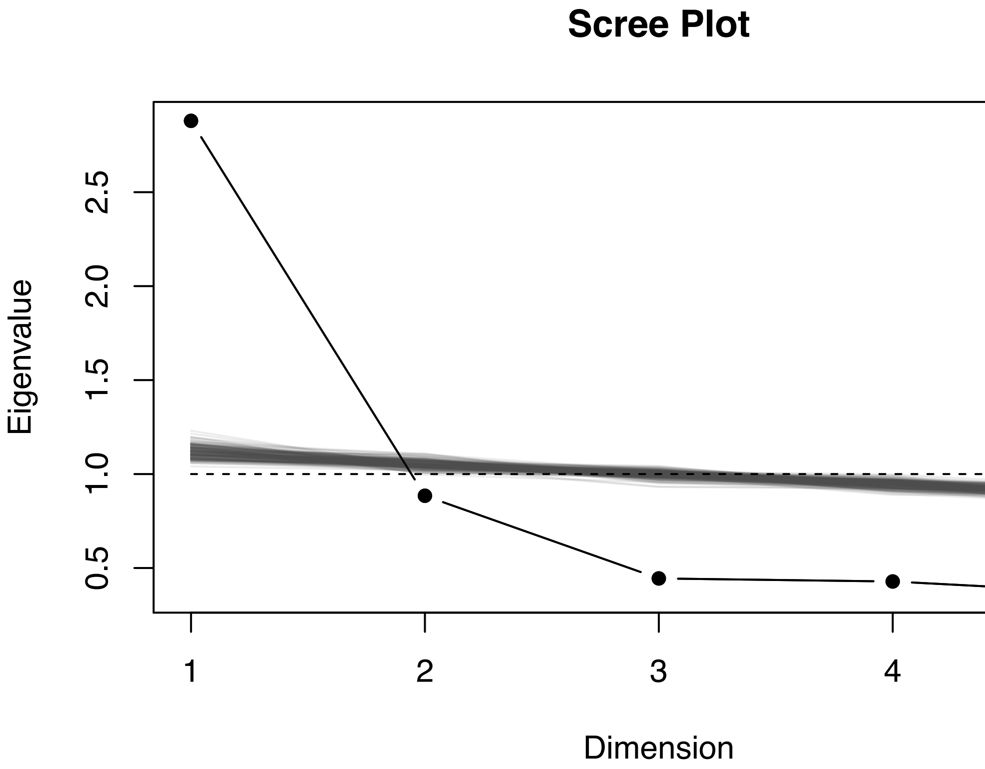

Unidimensionality: Hopelessness

```
## [1] "Ratio of first to second eigenvalues: 3.256"

## [1] 2.8795248 0.8845061 0.4445027 0.4283331 0.3631333

## Factor Analysis using method =  minres
## Call: fa(r = grm_obj$X)
## Standardized loadings (pattern matrix) based upon correlation matrix
##       MR1   h2   u2 com
## Q15  0.44 0.19 0.81   1
## Q61  0.71 0.51 0.49   1
## Q115 0.81 0.66 0.34   1
## Q24  0.76 0.57 0.43   1
## Q88  0.69 0.47 0.53   1
##
##                 MR1
## SS loadings    2.40
## Proportion Var 0.48
##
## Mean item complexity =  1
## Test of the hypothesis that 1 factor is sufficient.
##
## The degrees of freedom for the null model are  10  and the objective function was  1.74 with Chi Squa
## The degrees of freedom for the model are 5  and the objective function was  0.11
##
## The root mean square of the residuals (RMSR) is  0.06
## The df corrected root mean square of the residuals is  0.09
##
## The harmonic number of observations is  566 with the empirical chi square  47.63  with prob <  4.2e-
## The total number of observations was  596  with Likelihood Chi Square =  65.01  with prob <  1.1e-12
##
## Tucker Lewis Index of factoring reliability =  0.882
```

```
## RMSEA index =  0.142  and the 90 % confidence intervals are  0.112 0.174
## BIC =  33.06
## Fit based upon off diagonal values = 0.98
## Measures of factor score adequacy
##                                                   MR1
## Correlation of (regression) scores with factors   0.92
## Multiple R square of scores with factors          0.84
## Minimum correlation of possible factor scores     0.69
```

Graded-Response Model: Hopelessness

```
##      Extrmt1 Extrmt2 Extrmt3 Extrmt4 Extrmt5 Extrmt6 Dscrmn
## Q15   -0.801   0.062   0.933   2.042   2.959   4.350  0.926
## Q61   -0.387   0.251   0.693   1.207   1.732   2.267  2.014
## Q115  -1.168  -0.560  -0.037   0.488   0.876   1.374  2.850
## Q24   -1.012  -0.349   0.117   0.521   0.965   1.481  2.321
## Q88   -1.584  -1.019  -0.653  -0.115   0.288   0.814  2.121
```

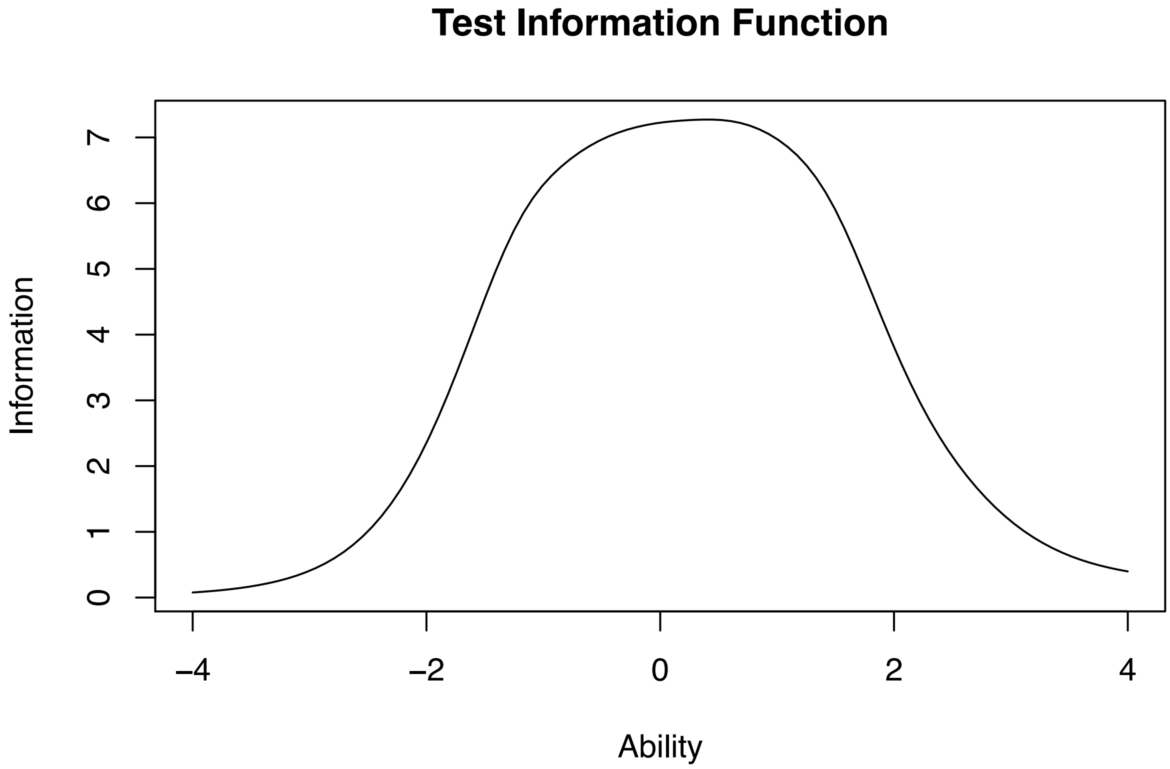

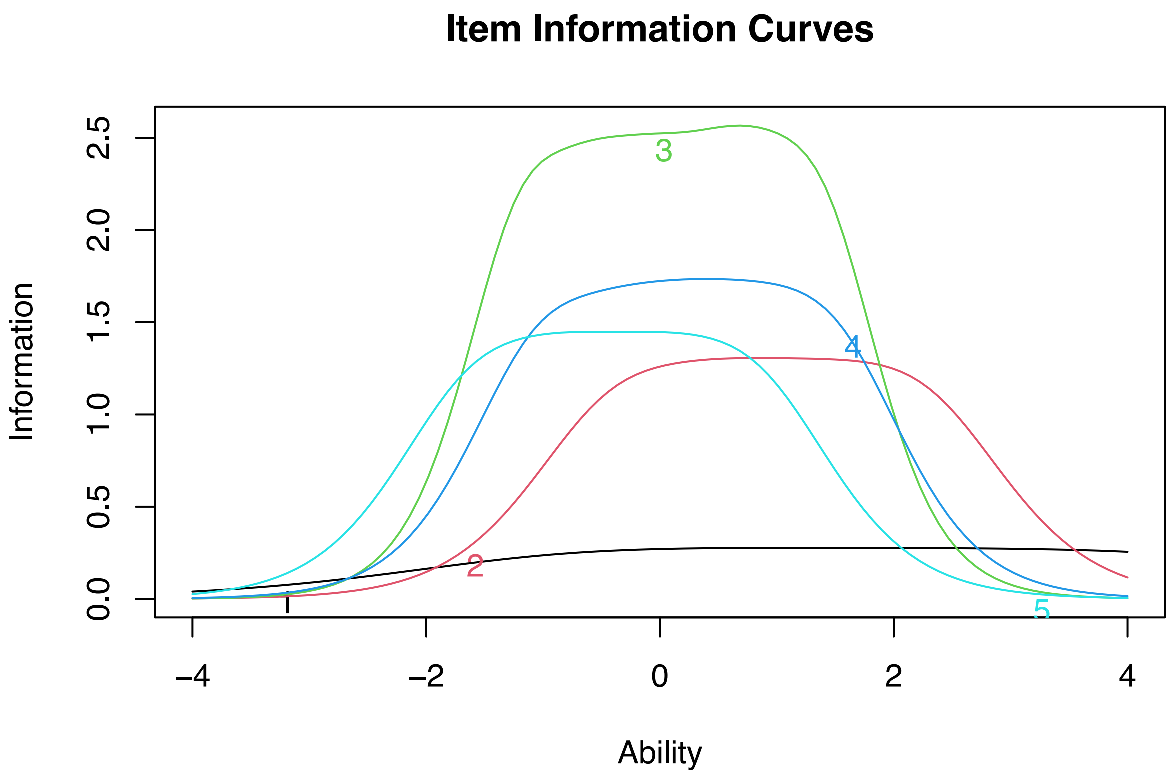

Back to top

Site DIF

```
## Call:
## lordif::lordif(resp.data = as.data.frame(merged_data), group = site)
##
##   Number of DIF groups: 2
##
##   Number of items flagged for DIF: 1 of 5
##
##   Items flagged: 3
##
##   Number of iterations for purification: 2 of 10
##
##   Detection criterion: Chisqr
##
##   Threshold: alpha = 0.01
##
##   item ncat  chi12  chi13  chi23
## 1    1    7 0.7690 0.2831 0.1184
## 2    2    7 0.0695 0.1213 0.3364
## 3    3    7 0.0020 0.0084 0.9961
## 4    4    7 0.6814 0.5746 0.3324
## 5    5    7 0.9635 0.4093 0.1816
```

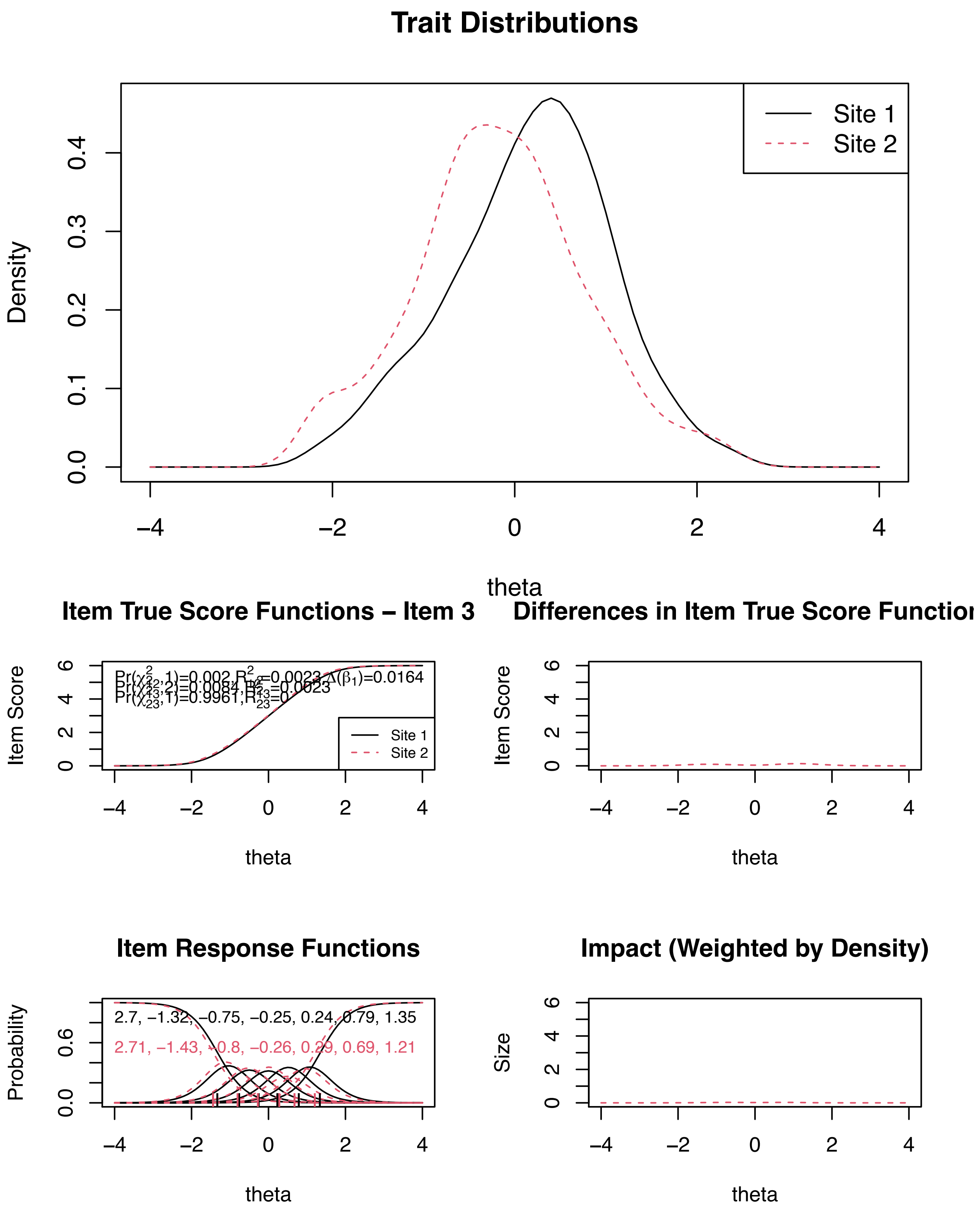
Trait Distributions
Density
Site 1
Site 2
theta
Item True Score Functions – Item 3
Item Score
Site 1
Site 2
theta
Differences in Item True Score Functio
Item Score
theta
Item Response Functions
Probability
2.7, −1.32, −0.75, −0.25, 0.24, 0.79, 1.35
2.71, −1.43, −0.8, −0.26, 0.29, 0.69, 1.21
theta
Impact (Weighted by Density)
Size
theta

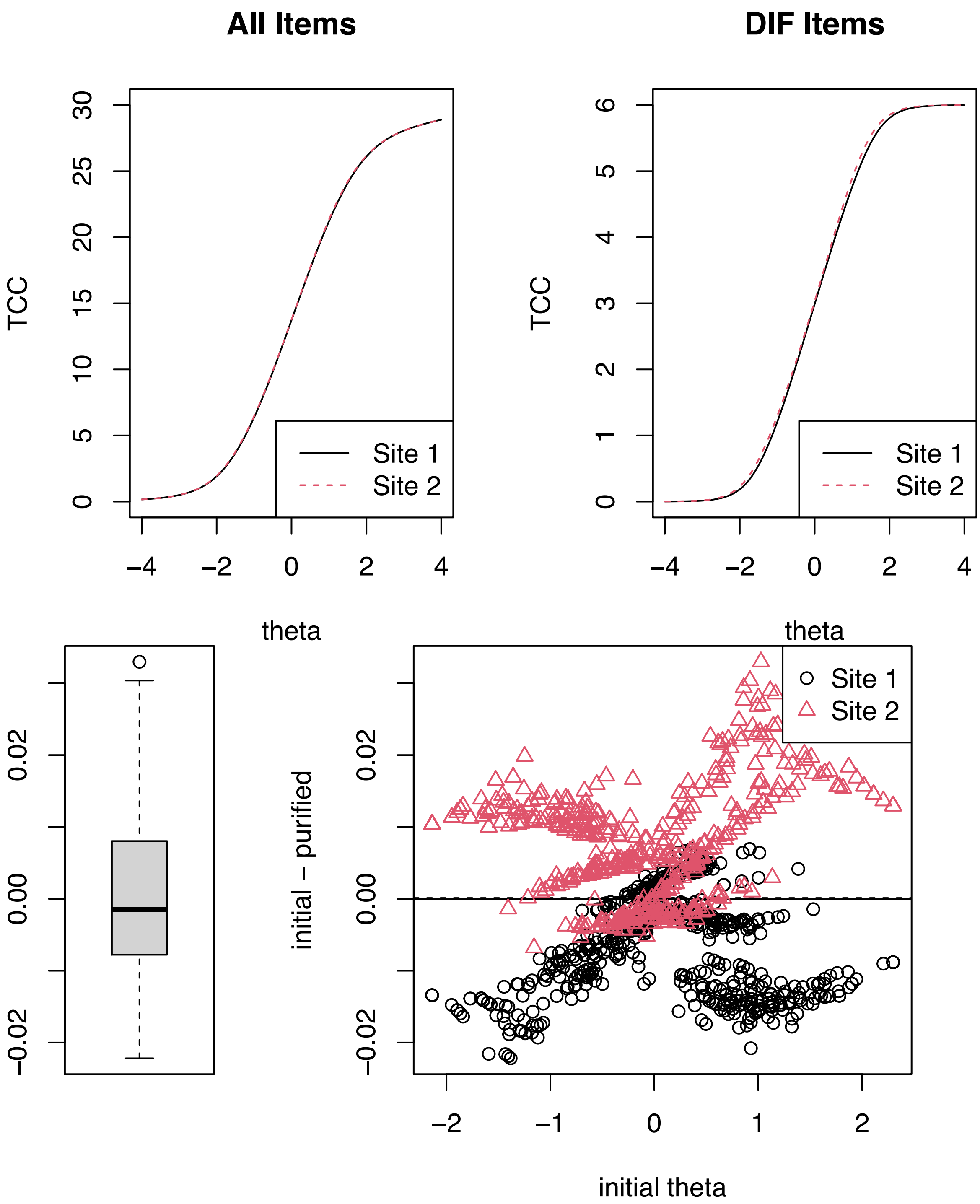

Back to top

Gender-based DIF: Hopelessness

```
## Call:
## lordif::lordif(resp.data = as.data.frame(sex.data), group = sex)
##
##   Number of DIF groups: 2
```

```
##
##   Number of items flagged for DIF: 1 of 5
##
##   Items flagged: 2
##
##   Number of iterations for purification: 2 of 10
##
##   Detection criterion: Chisqr
##
##   Threshold: alpha = 0.01
##
##   item ncat  chi12  chi13  chi23
## 1    1    7 0.1092 0.1791 0.3499
## 2    2    7 0.0000 0.0000 0.4704
## 3    3    7 0.4284 0.3726 0.2457
## 4    4    7 0.1036 0.2632 0.8874
## 5    5    7 0.6145 0.8328 0.7375
```

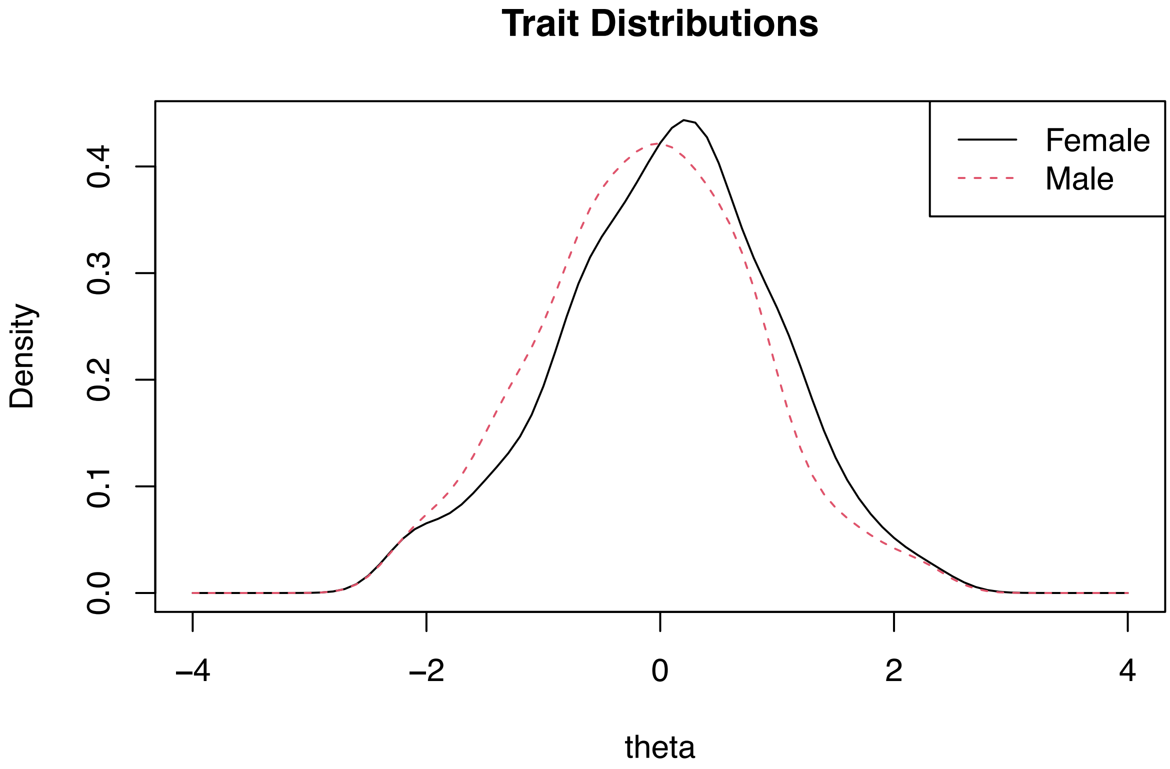

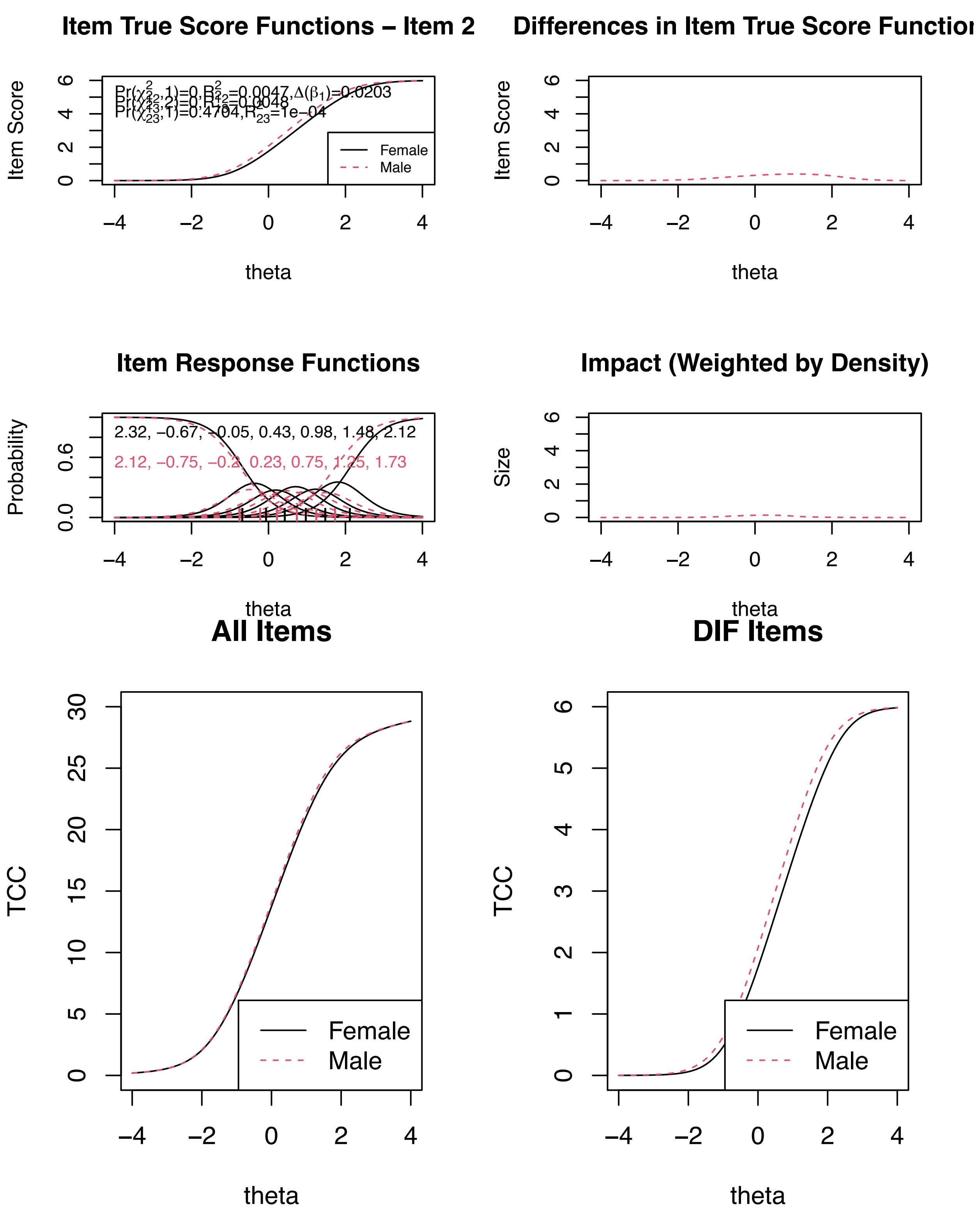
Item True Score Functions – Item 2
Item Score
theta
Pr(χ²12,1)=0,R²12=0.0047,Δ(β1)=0.0203
Pr(χ²13,2)=0,R²13=0.0048
Pr(χ²23,1)=0.4704,R²23=1e−04
Female
Male
Differences in Item True Score Functio
Item Score
theta
Item Response Functions
Probability
theta
2.32, −0.67, −0.05, 0.43, 0.98, 1.48, 2.12
2.12, −0.75, −0.2, 0.23, 0.75, 1.25, 1.73
Impact (Weighted by Density)
Size
theta
All Items
TCC
theta
Female
Male
DIF Items
TCC
theta
Female
Male

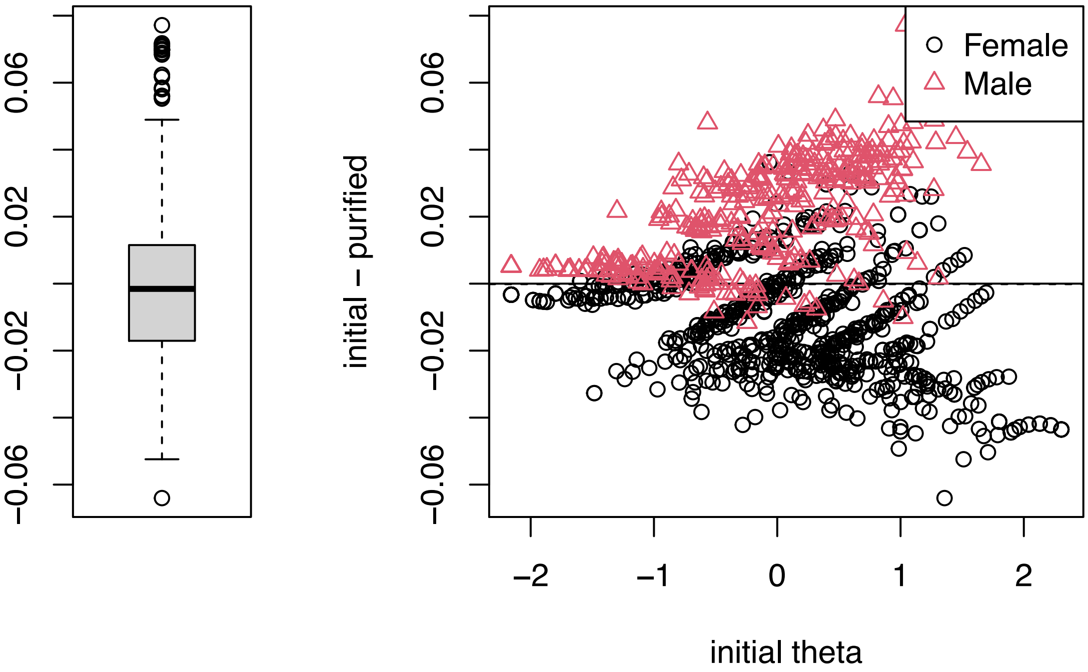

Age-based DIF: Hopelessness

```
## Call:
## lordif::lordif(resp.data = as.data.frame(age.data), group = age)
##
##   Number of DIF groups: 2
##
##   Number of items flagged for DIF: 0 of 5
##
##   Items flagged:
##
##   Number of iterations for purification: 1 of 10
##
##   Detection criterion: Chisqr
##
##   Threshold: alpha = 0.01
```

Back to top

Internal Avoidance

Site 1

Reliability: Internal Avoidance

```
## Cronbach's alpha is 0.733.
## Mean item-total correlation is 0.348.
## If each item were dropped:
##      raw_alpha std.alpha G6(smc) average_r S/N alpha se  var.r med.r
## Q10       0.78      0.78    0.73      0.47 3.5    0.015 0.0081  0.45
## Q34       0.62      0.62    0.57      0.29 1.6    0.024 0.0181  0.28
```

```
## Q78        0.70      0.69    0.67      0.36 2.2    0.020 0.0373  0.33
## Q122       0.66      0.65    0.62      0.32 1.9    0.022 0.0348  0.30
## Q123       0.65      0.64    0.61      0.31 1.8    0.023 0.0303  0.28
```

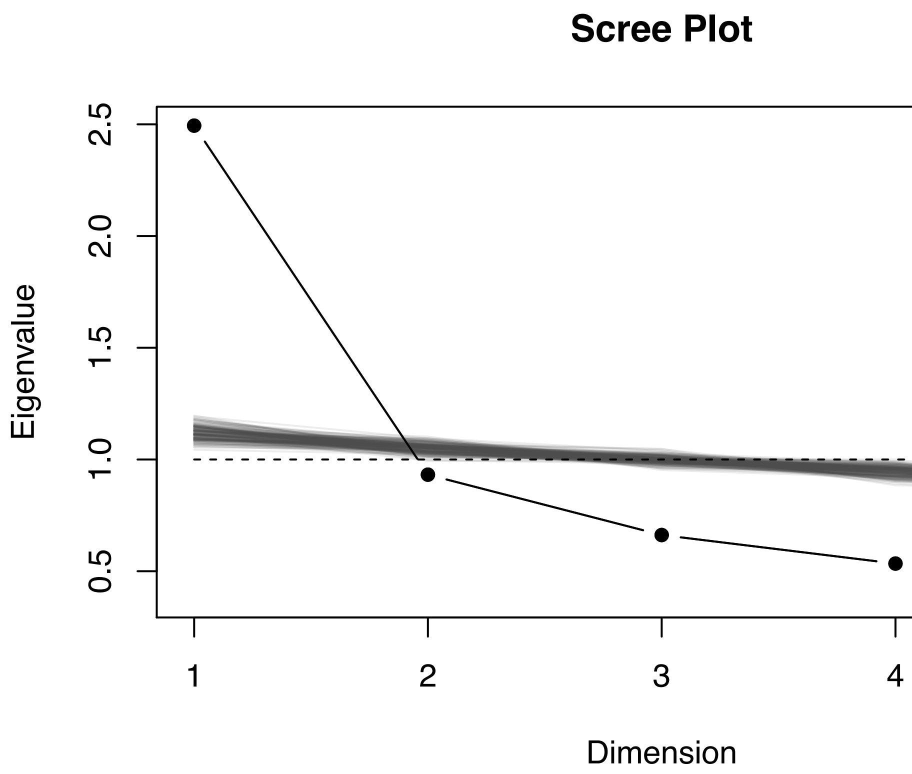

Unidimensionality: Internal Avoidance

```
## [1] "Ratio of first to second eigenvalues: 2.676"
```

```
## [1] 2.4939949 0.9320973 0.6619944 0.5341351 0.3777783
```

```
## Factor Analysis using method =  minres
## Call: fa(r = grm_obj$X)
## Standardized loadings (pattern matrix) based upon correlation matrix
##       MR1    h2   u2 com
## Q10  0.25 0.061 0.94   1
## Q34  0.82 0.678 0.32   1
## Q78  0.55 0.303 0.70   1
## Q122 0.67 0.446 0.55   1
## Q123 0.71 0.497 0.50   1
##
##                 MR1
## SS loadings    1.99
## Proportion Var 0.40
##
## Mean item complexity =  1
## Test of the hypothesis that 1 factor is sufficient.
##
## The degrees of freedom for the null model are  10  and the objective function was  1.17 with Chi Squa
## The degrees of freedom for the model are 5  and the objective function was  0.02
##
## The root mean square of the residuals (RMSR) is  0.03
## The df corrected root mean square of the residuals is  0.04
```

```
##
## The harmonic number of observations is  567 with the empirical chi square  7.15  with prob <  0.21
## The total number of observations was  617  with Likelihood Chi Square =  10.76  with prob <  0.056
##
## Tucker Lewis Index of factoring reliability =  0.984
## RMSEA index =  0.043  and the 90 % confidence intervals are  0 0.079
## BIC =  -21.36
## Fit based upon off diagonal values = 1
## Measures of factor score adequacy
##                                                    MR1
## Correlation of (regression) scores with factors   0.90
## Multiple R square of scores with factors          0.81
## Minimum correlation of possible factor scores     0.63
```

Graded-Response Model: Internal Avoidance

```
##      Extrmt1 Extrmt2 Extrmt3 Extrmt4 Extrmt5 Extrmt6 Dscrmn
## Q10   -5.076  -3.744  -1.704  -0.062   1.351   3.583  0.477
## Q34   -1.548  -0.913  -0.526  -0.140   0.369   0.881  2.888
## Q78   -2.058  -1.288  -0.752  -0.076   0.548   1.326  1.316
## Q122  -1.403  -0.883  -0.361   0.180   0.715   1.301  1.800
## Q123  -1.641  -0.975  -0.648  -0.143   0.427   1.030  1.866
```

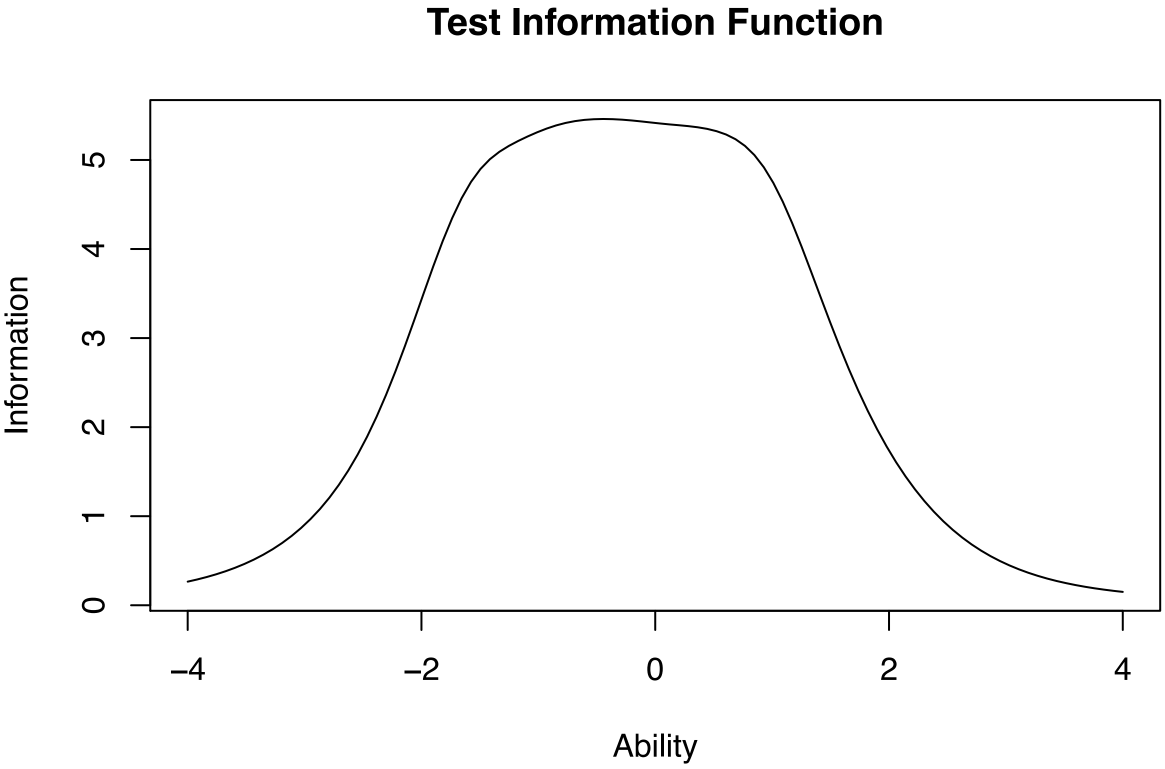

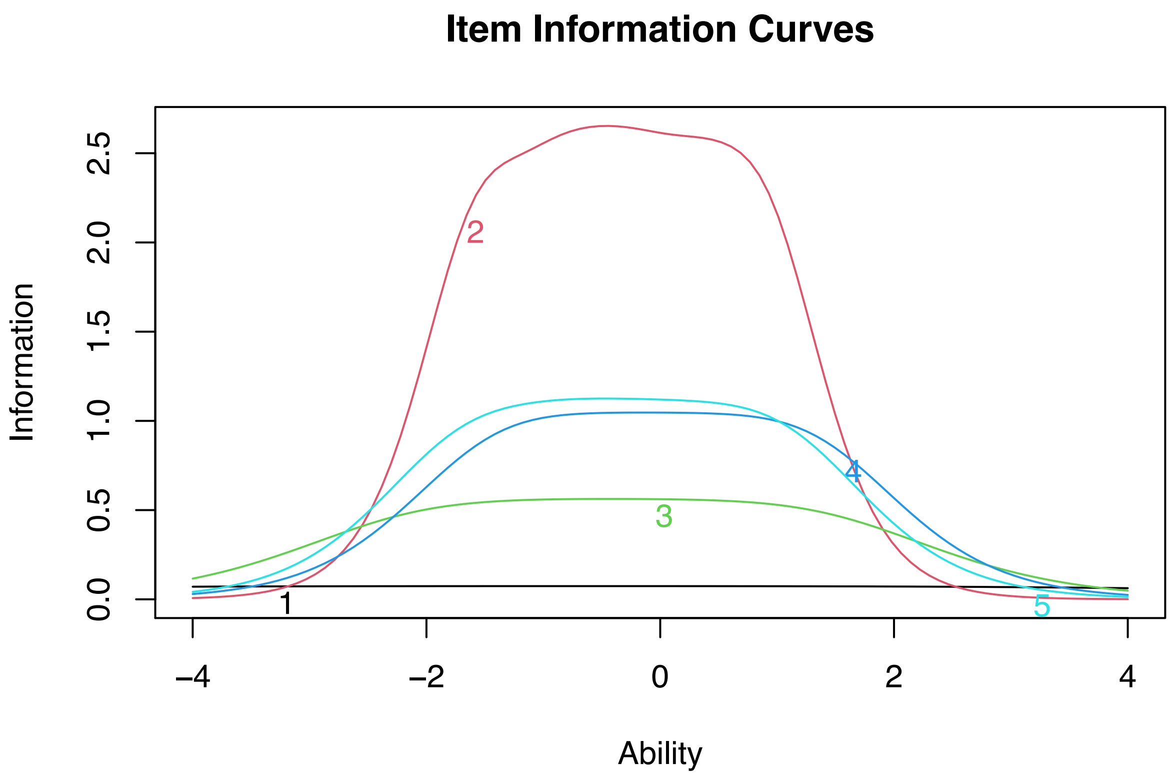

Back to top

Site 2

Reliability: Internal Avoidance

```
## Cronbach's alpha is 0.761.
## Mean item-total correlation is 0.381.
## If each item were dropped:
##      raw_alpha std.alpha G6(smc) average_r S/N alpha se var.r med.r
## Q10       0.79      0.79    0.76      0.49 3.9    0.014 0.013  0.50
## Q34       0.66      0.65    0.61      0.32 1.9    0.023 0.021  0.31
## Q78       0.75      0.74    0.71      0.42 2.9    0.017 0.036  0.41
## Q122      0.68      0.67    0.64      0.34 2.0    0.021 0.028  0.31
## Q123      0.68      0.68    0.65      0.35 2.1    0.021 0.029  0.31
```

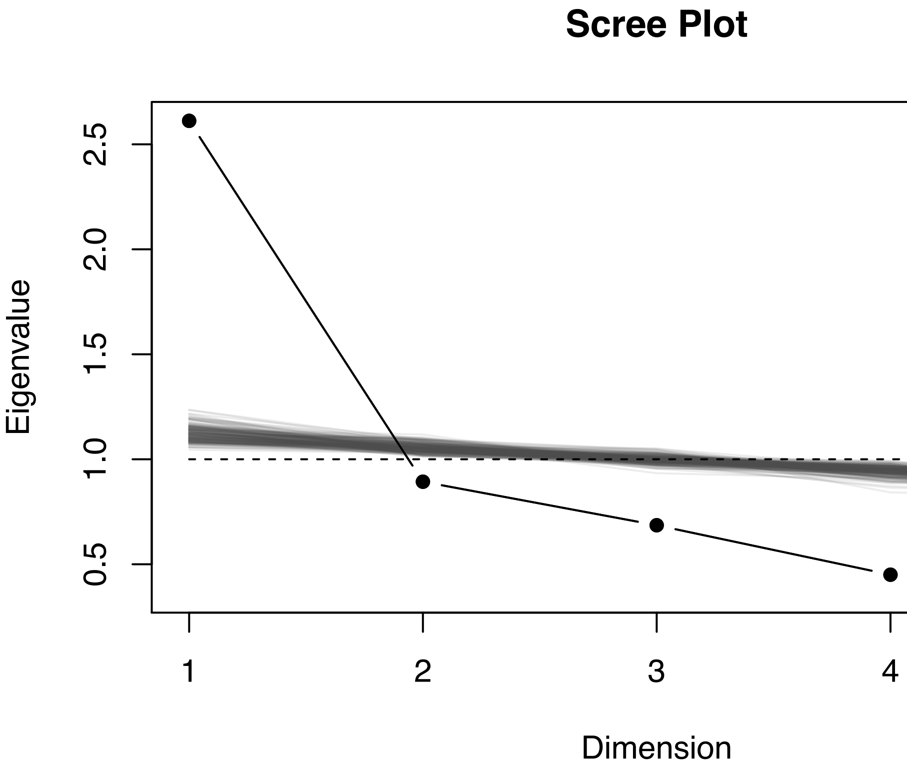

Unidimensionality: Internal Avoidance

```
## [1] "Ratio of first to second eigenvalues: 2.925"

## [1] 2.6116558 0.8929579 0.6857242 0.4501308 0.3595313

## Factor Analysis using method =  minres
## Call: fa(r = grm_obj$X)
## Standardized loadings (pattern matrix) based upon correlation matrix
##       MR1    h2   u2 com
## Q10  0.31 0.097 0.90   1
## Q34  0.83 0.694 0.31   1
## Q78  0.51 0.257 0.74   1
## Q122 0.75 0.561 0.44   1
## Q123 0.73 0.527 0.47   1
##
##                 MR1
## SS loadings    2.14
## Proportion Var 0.43
##
## Mean item complexity =  1
## Test of the hypothesis that 1 factor is sufficient.
##
## The degrees of freedom for the null model are  10  and the objective function was  1.35 with Chi Squa
## The degrees of freedom for the model are 5  and the objective function was  0.01
##
## The root mean square of the residuals (RMSR) is  0.02
## The df corrected root mean square of the residuals is  0.02
##
## The harmonic number of observations is  542 with the empirical chi square  3.12  with prob <  0.68
## The total number of observations was  596  with Likelihood Chi Square =  5.24  with prob <  0.39
##
## Tucker Lewis Index of factoring reliability =  0.999
```

```
## RMSEA index =  0.009  and the 90 % confidence intervals are  0 0.058
## BIC =  -26.71
## Fit based upon off diagonal values = 1
## Measures of factor score adequacy
##                                                    MR1
## Correlation of (regression) scores with factors   0.91
## Multiple R square of scores with factors          0.84
## Minimum correlation of possible factor scores     0.67
```

Graded-Response Model: Internal Avoidance

```
##      Extrmt1 Extrmt2 Extrmt3 Extrmt4 Extrmt5 Extrmt6 Dscrmn
## Q10    2.978   1.889   0.736  -0.634  -2.056  -3.669 -0.666
## Q34    1.203   0.682   0.252  -0.178  -0.645  -1.112 -3.012
## Q78    2.165   1.275   0.521  -0.180  -0.957  -1.771 -1.118
## Q122   1.198   0.618   0.144  -0.336  -0.792  -1.412 -2.279
## Q123   1.397   0.771   0.362  -0.093  -0.587  -1.066 -2.129
```

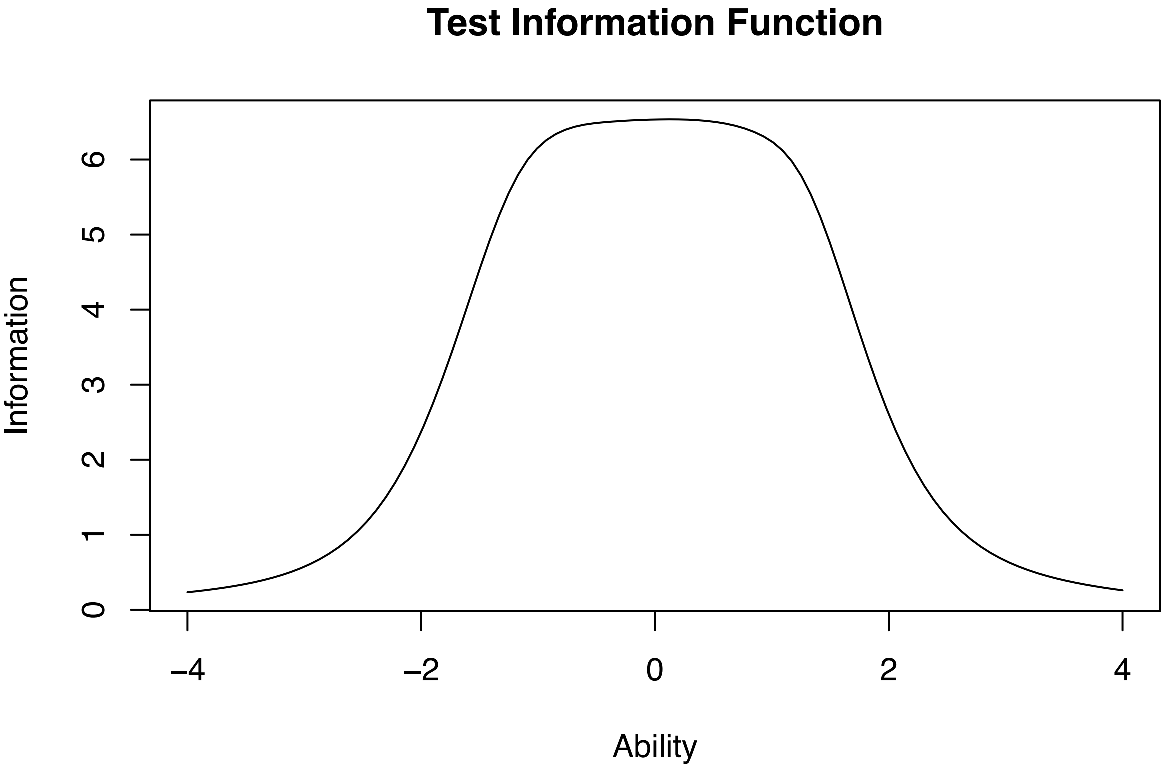

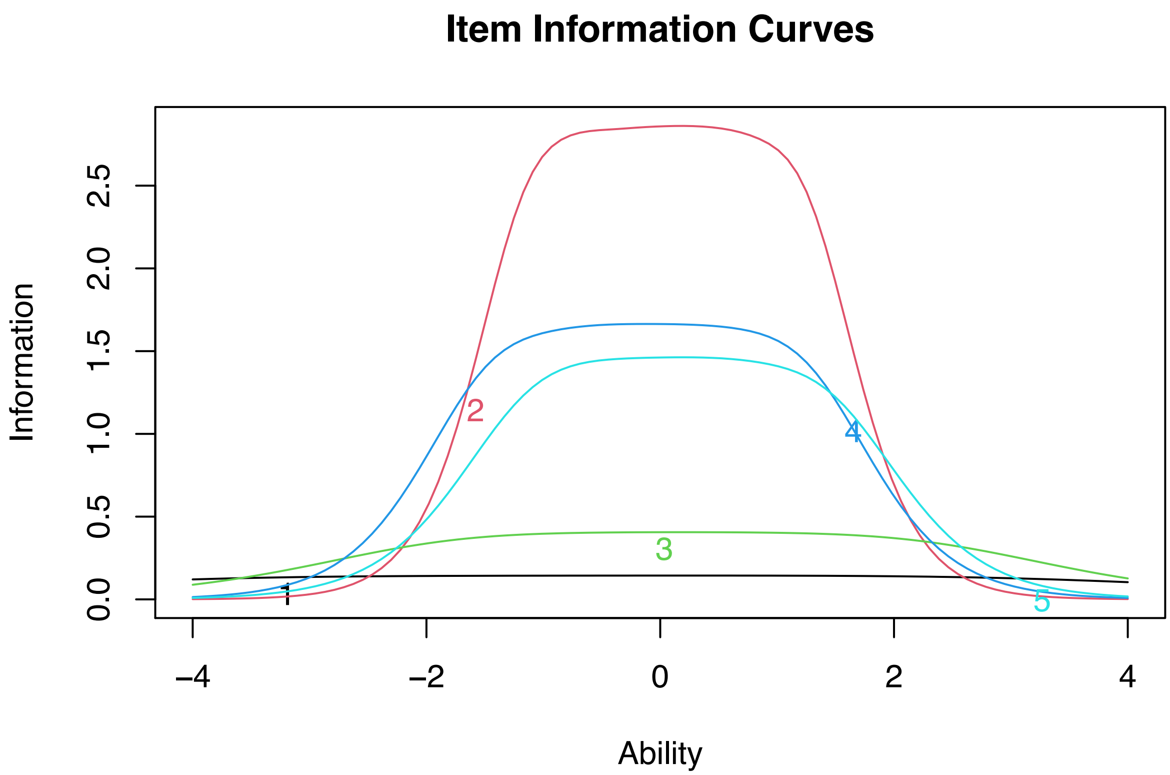

Back to top

Site DIF

```
## Call:
## lordif::lordif(resp.data = as.data.frame(merged_data), group = site)
##
##   Number of DIF groups: 2
##
##   Number of items flagged for DIF: 1 of 5
##
##   Items flagged: 1
##
##   Number of iterations for purification: 2 of 10
##
##   Detection criterion: Chisqr
##
##   Threshold: alpha = 0.01
##
##   item ncat  chi12  chi13  chi23
## 1    1    7 0.0080 0.0182 0.3231
## 2    2    7 0.1544 0.2378 0.3581
## 3    3    7 0.5892 0.2471 0.1135
## 4    4    7 0.2460 0.4601 0.6494
## 5    5    7 0.3006 0.5603 0.7682
```

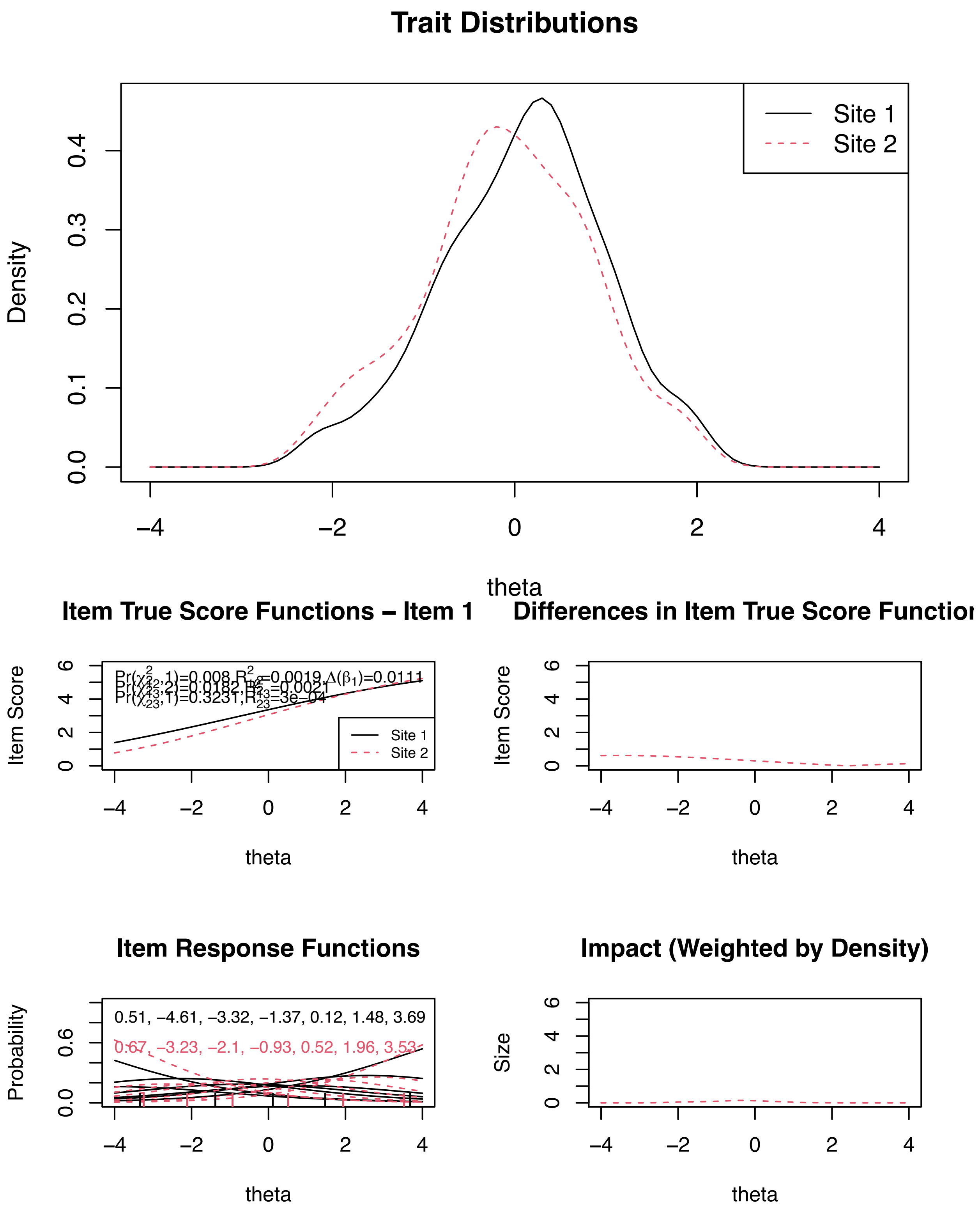
Trait Distributions
Density
theta
Site 1
Site 2
Item True Score Functions – Item 1
Differences in Item True Score Functio
Item Score
Item Response Functions
Impact (Weighted by Density)
Probability
Size
0.51, –4.61, –3.32, –1.37, 0.12, 1.48, 3.69
0.67, –3.23, –2.1, –0.93, 0.52, 1.96, 3.53

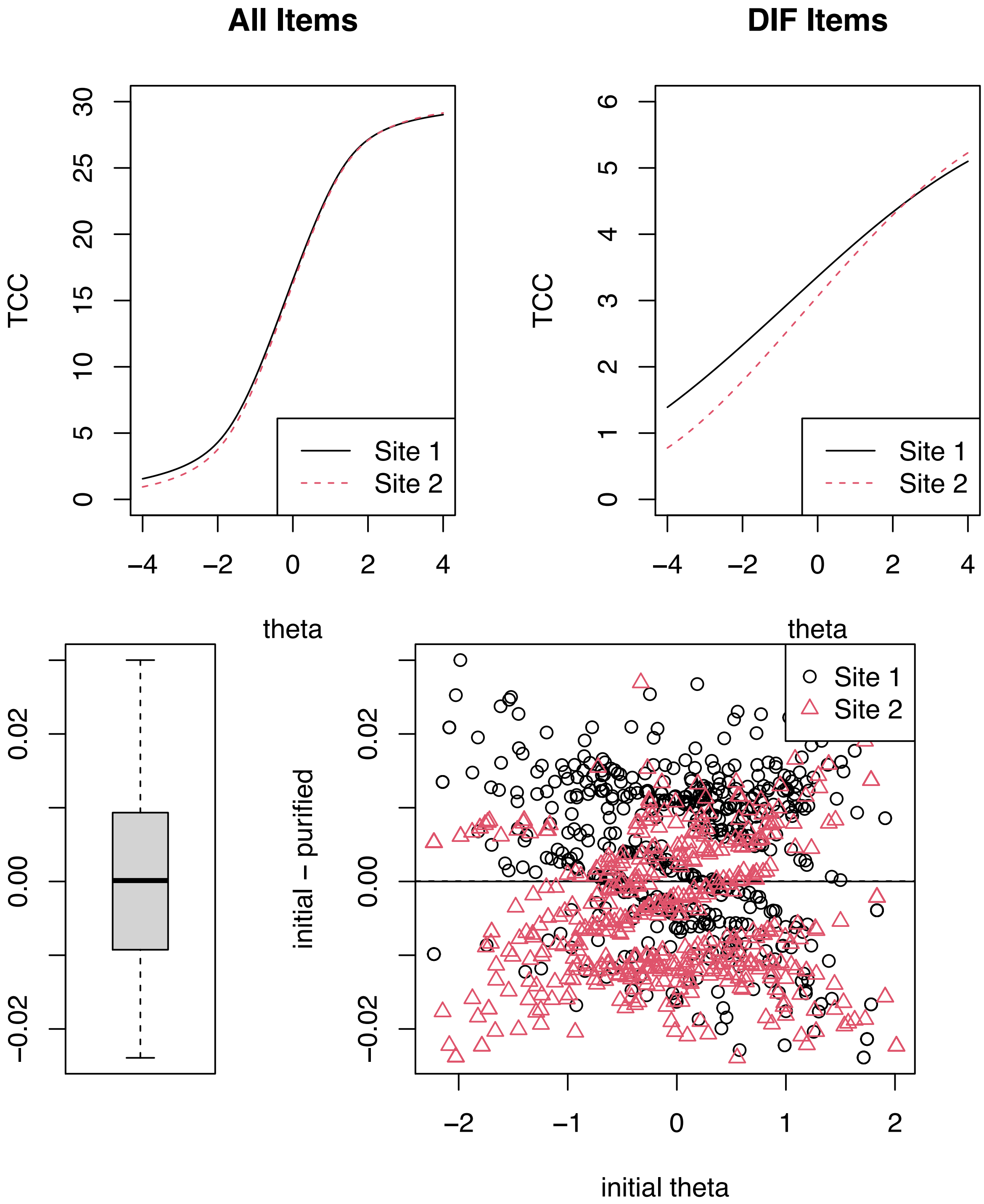

Back to top

Gender-based DIF: Internal Avoidance

```
## Call:
## lordif::lordif(resp.data = as.data.frame(sex.data), group = sex)
##
##   Number of DIF groups: 2
```

```
##
##   Number of items flagged for DIF: 2 of 5
##
##   Items flagged: 4, 5
##
##   Number of iterations for purification: 2 of 10
##
##   Detection criterion: Chisqr
##
##   Threshold: alpha = 0.01
##
##   item ncat  chi12  chi13  chi23
## 1    1    7 0.7329 0.8544 0.6562
## 2    2    7 0.9395 0.9899 0.9043
## 3    3    7 0.4268 0.6522 0.6364
## 4    4    7 0.0000 0.0000 0.9679
## 5    5    7 0.0003 0.0014 0.9609
```

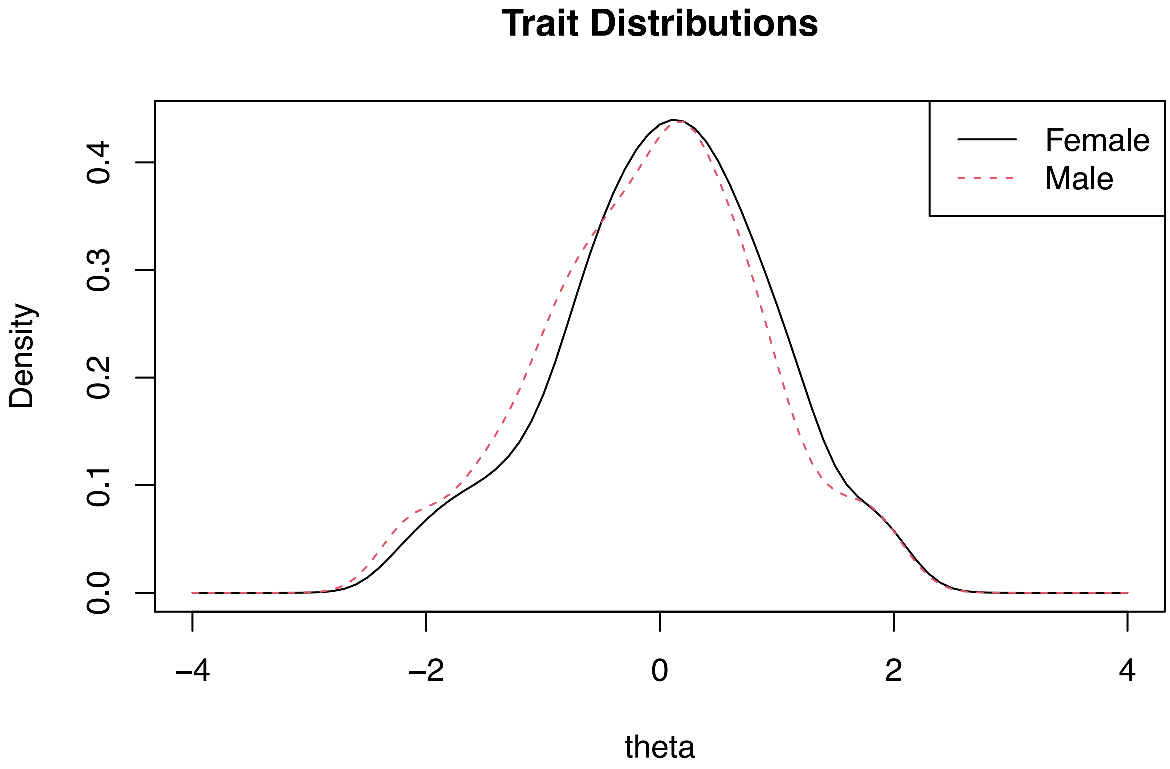

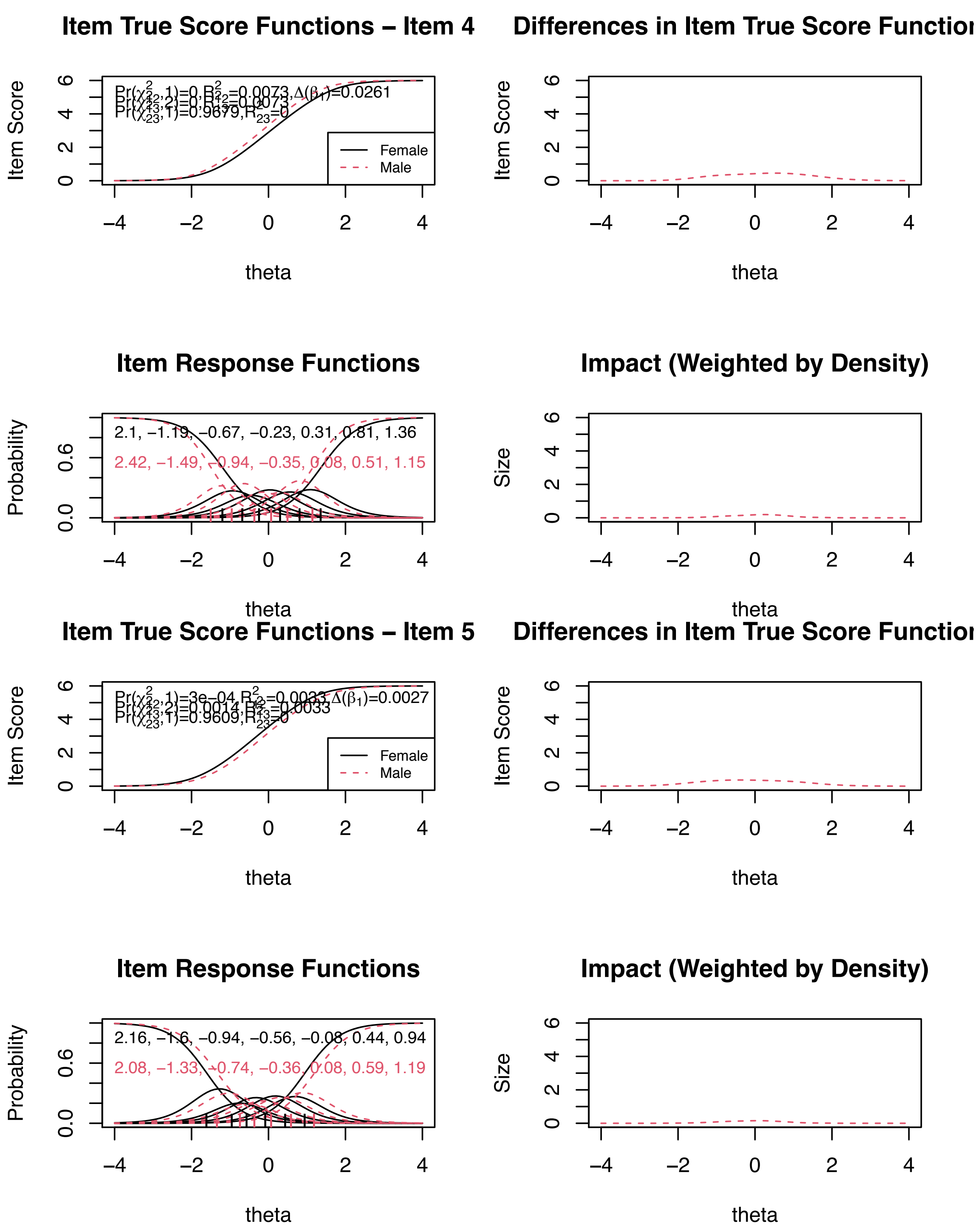
Item True Score Functions – Item 4
Differences in Item True Score Functio
Item Score
theta
Female
Male
2.1, −1.19, −0.67, −0.23, 0.31, 0.81, 1.36
2.42, −1.49, −0.94, −0.35, 0.08, 0.51, 1.15
Item Response Functions
Impact (Weighted by Density)
Probability
Size
Item True Score Functions – Item 5
2.16, −1.6, −0.94, −0.56, −0.08, 0.44, 0.94
2.08, −1.33, −0.74, −0.36, 0.08, 0.59, 1.19

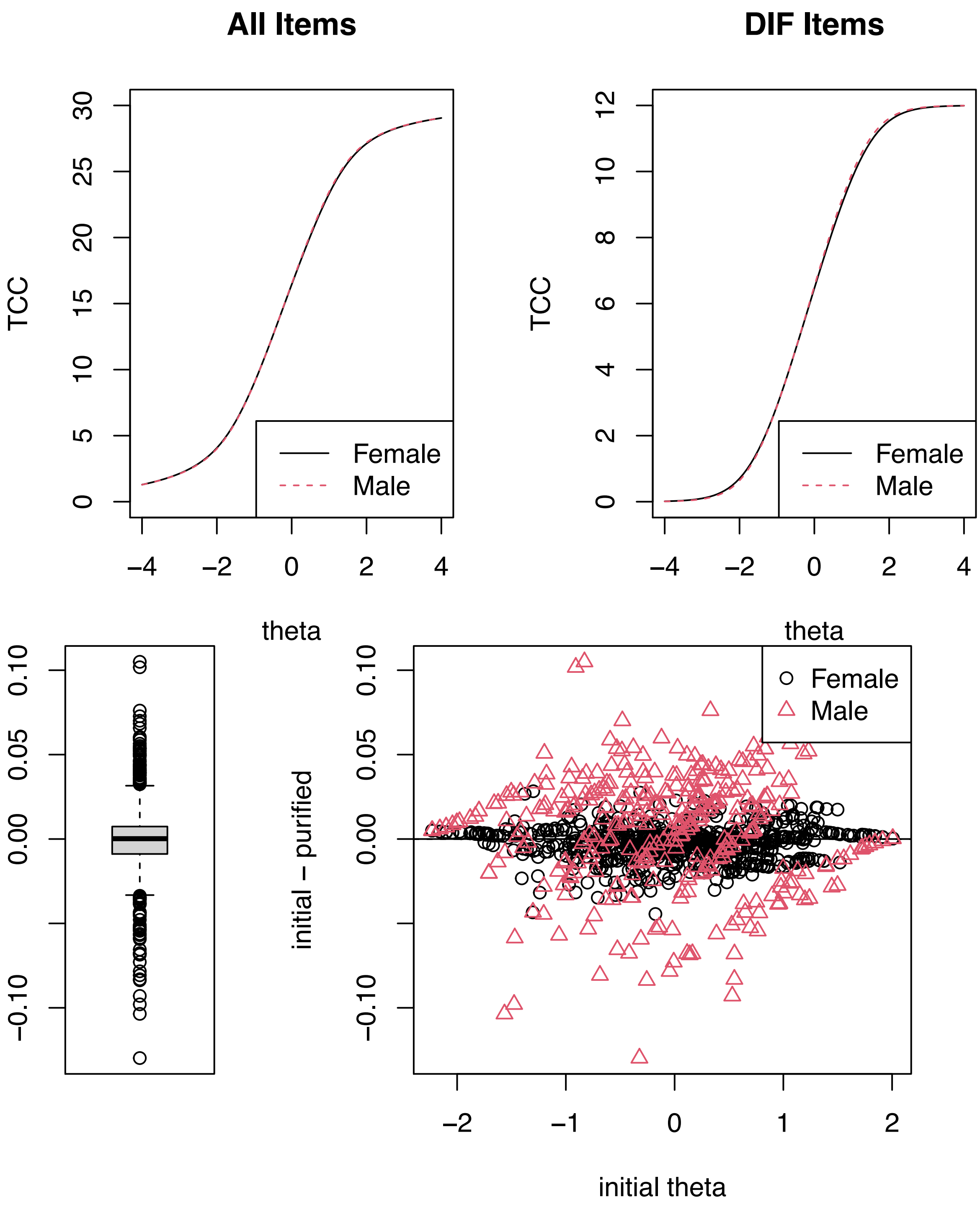

Age-based DIF: Internal Avoidance

```
## Call:
## lordif::lordif(resp.data = as.data.frame(age.data), group = age)
##
##   Number of DIF groups: 2
##
```

```
##   Number of items flagged for DIF: 1 of 5
##
##   Items flagged: 1
##
##   Number of iterations for purification: 2 of 10
##
##   Detection criterion: Chisqr
##
##   Threshold: alpha = 0.01
##
##   item ncat  chi12  chi13  chi23
## 1    1    7 0.0001 0.0003 0.4479
## 2    2    7 0.3786 0.6404 0.7333
## 3    3    7 0.3468 0.6054 0.7305
## 4    4    7 0.8914 0.8869 0.6379
## 5    5    7 0.0768 0.1975 0.7376
```

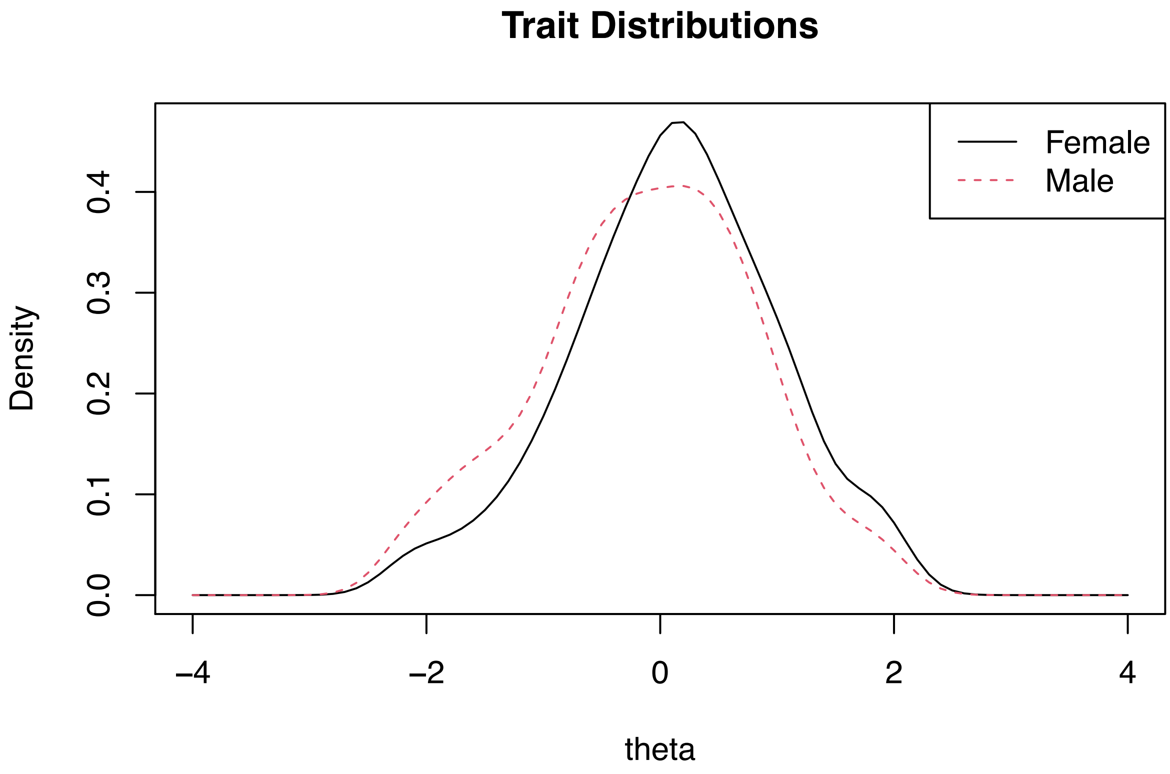

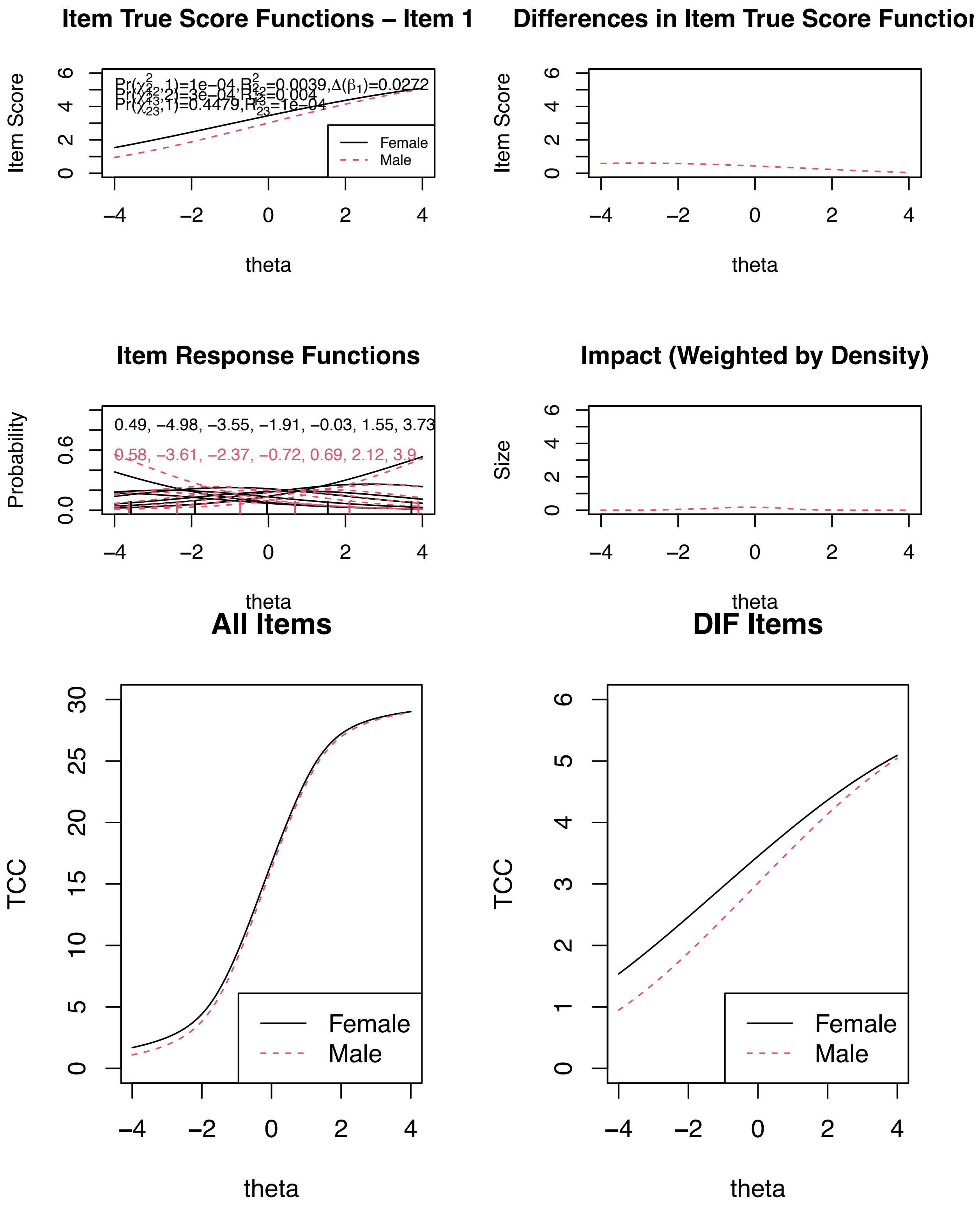
Item True Score Functions – Item 1
Differences in Item True Score Functio
Item Response Functions
Impact (Weighted by Density)
All Items
DIF Items
Female
Male
theta
Item Score
Probability
Size
TCC
0.49, −4.98, −3.55, −1.91, −0.03, 1.55, 3.73
0.58, −3.61, −2.37, −0.72, 0.69, 2.12, 3.9

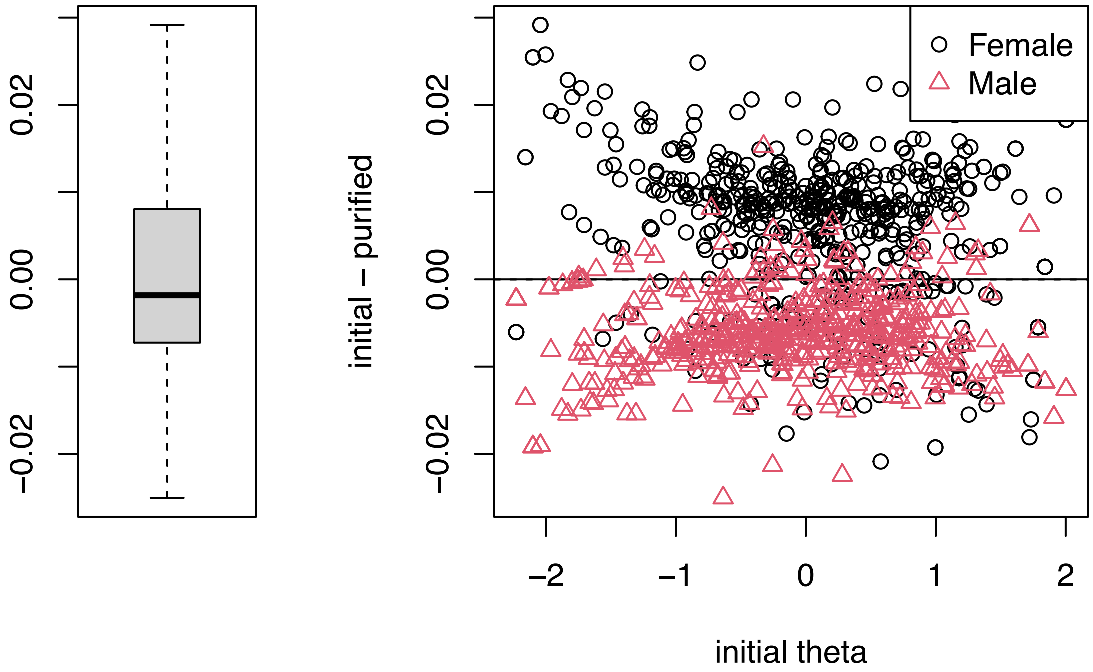

Back to top

Irritability

Site 1

Reliability: Irritability

```
## Cronbach's alpha is 0.45.
## Mean item-total correlation is 0.22.
## If each item were dropped:
##      raw_alpha std.alpha G6(smc) average_r  S/N alpha se var.r med.r
## Q37       0.38      0.38    0.23      0.23 0.61    0.050    NA  0.23
## Q114      0.46      0.46    0.30      0.30 0.87    0.043    NA  0.30
## Q65       0.22      0.22    0.12      0.12 0.28    0.063    NA  0.12
```

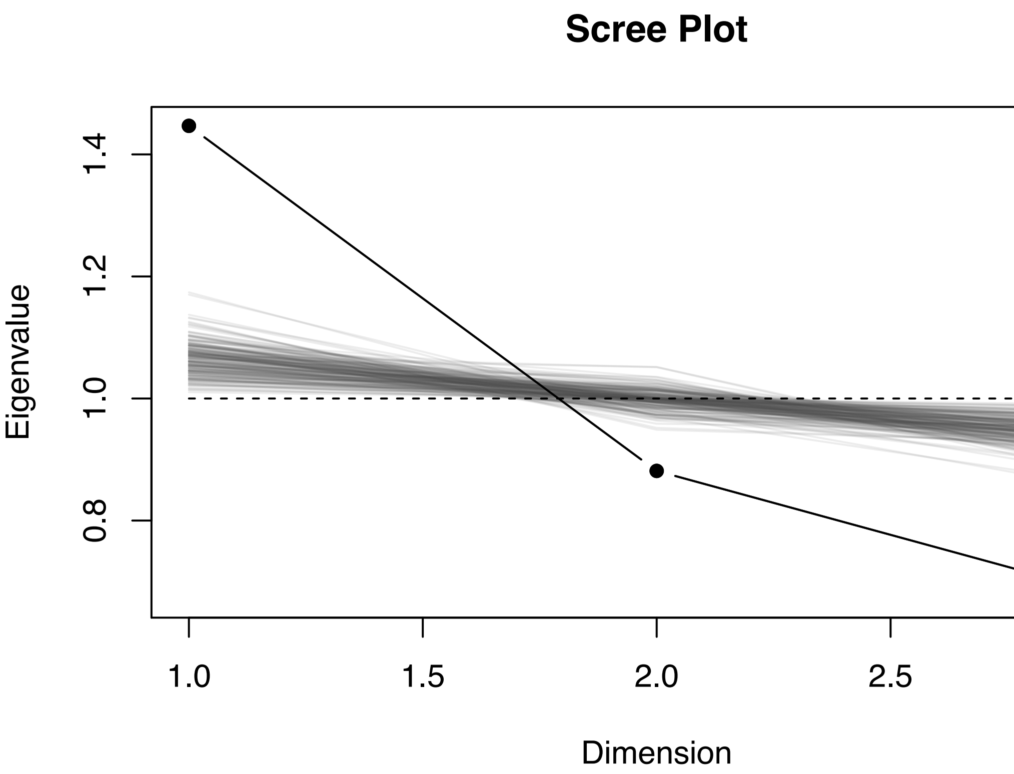

Unidimensionality: Irritability

```
## [1] "Ratio of first to second eigenvalues: 1.642"

## [1] 1.4467859 0.8813400 0.6718741

## Factor Analysis using method =  minres
## Call: fa(r = grm_obj$X)
## Standardized loadings (pattern matrix) based upon correlation matrix
##       MR1    h2   u2 com
## Q37  0.40 0.158 0.84   1
## Q114 0.31 0.096 0.90   1
## Q65  0.76 0.573 0.43   1
##
##                 MR1
## SS loadings    0.83
## Proportion Var 0.28
##
## Mean item complexity =  1
## Test of the hypothesis that 1 factor is sufficient.
##
## The degrees of freedom for the null model are  3  and the objective function was  0.15 with Chi Squa
## The degrees of freedom for the model are 0  and the objective function was  0
##
## The root mean square of the residuals (RMSR) is  0
## The df corrected root mean square of the residuals is  NA
##
## The harmonic number of observations is  580 with the empirical chi square  0  with prob <  NA
## The total number of observations was  617  with Likelihood Chi Square =  0  with prob <  NA
##
## Tucker Lewis Index of factoring reliability =  -Inf
## Fit based upon off diagonal values = 1
## Measures of factor score adequacy
```

```
##                                                    MR1
## Correlation of (regression) scores with factors   0.79
## Multiple R square of scores with factors          0.62
## Minimum correlation of possible factor scores     0.24
```

Graded-Response Model: Irritability

```
##      Extrmt1 Extrmt2 Extrmt3 Extrmt4 Extrmt5 Extrmt6 Dscrmn
## Q37   -3.013  -1.493  -0.510   0.211   1.123   2.158  0.823
## Q114  -0.386   0.857   1.726   2.639   3.518   4.816  0.707
## Q65   -0.345   0.409   0.849   1.366   1.937   2.456  2.439
```

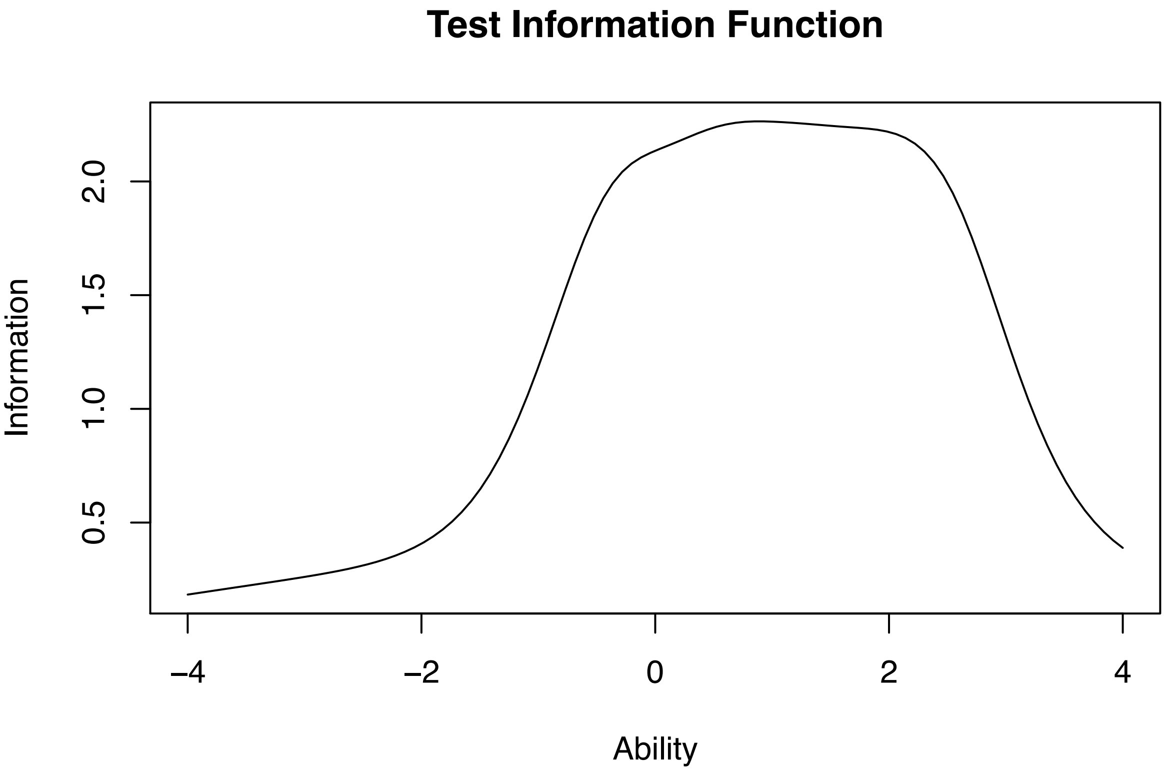

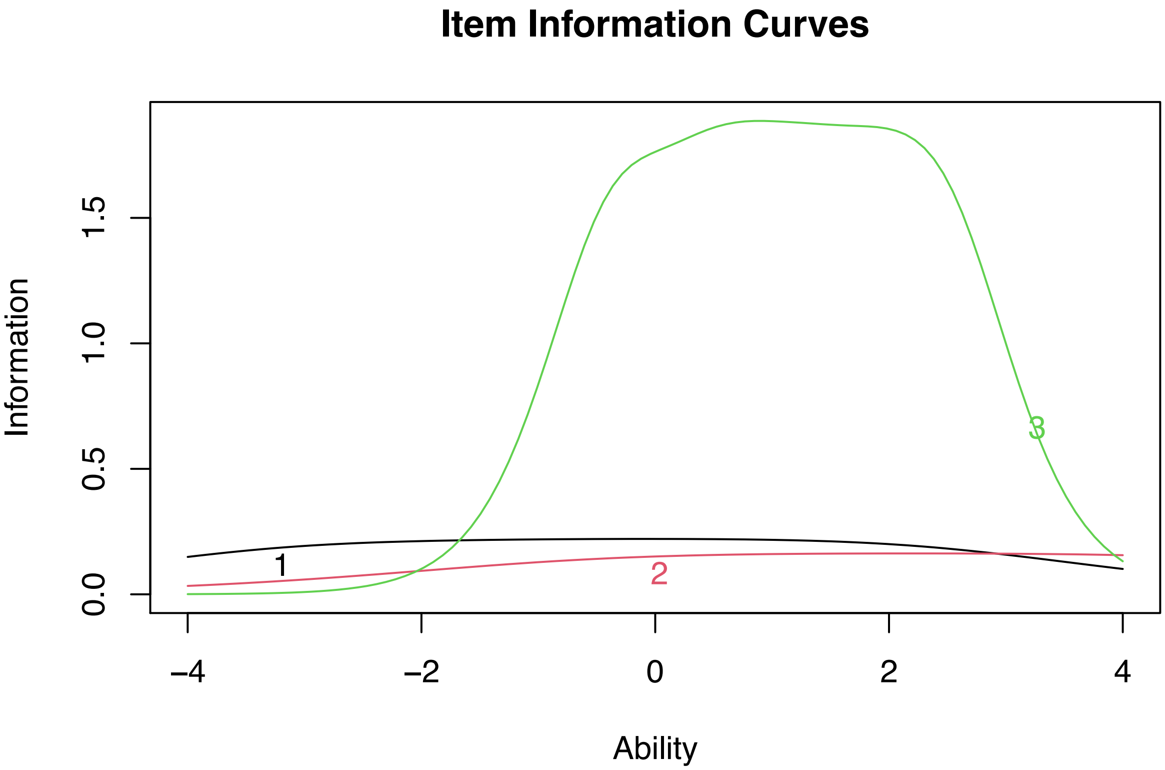

Back to top

Site 2

Reliability: Irritability

```
## Cronbach's alpha is 0.448.
## Mean item-total correlation is 0.227.
## If each item were dropped:
##      raw_alpha std.alpha G6(smc) average_r  S/N alpha se var.r med.r
## Q37       0.41      0.42   0.264     0.264 0.72    0.047    NA 0.264
## Q114      0.52      0.53   0.361     0.361 1.13    0.038    NA 0.361
## Q65       0.11      0.11   0.056     0.056 0.12    0.073    NA 0.056
```

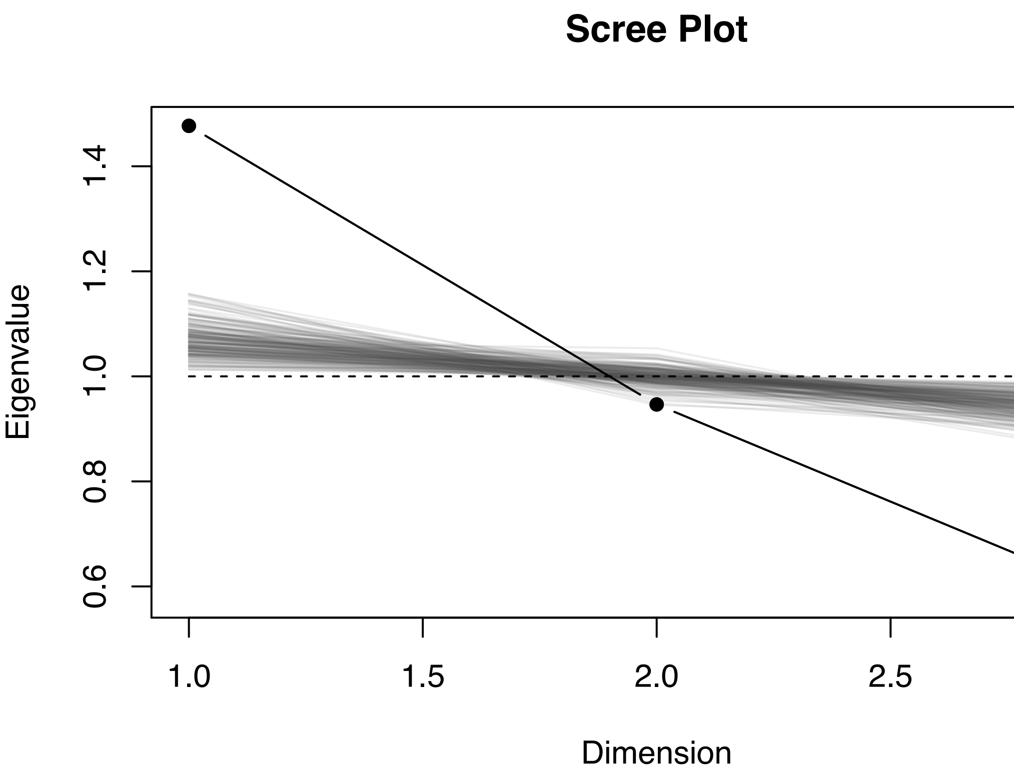

Unidimensionality: Irritability

```
## [1] "Ratio of first to second eigenvalues: 1.56"

## [1] 1.4769570 0.9464731 0.5765699

## Factor Analysis using method =  minres
## Call: fa(r = grm_obj$X)
## Standardized loadings (pattern matrix) based upon correlation matrix
##       MR1    h2      u2 com
## Q37  0.35 0.124  0.8763   1
## Q114 0.26 0.065  0.9346   1
## Q65  1.00 1.001 -0.0011   1
##
##                 MR1
## SS loadings    1.19
## Proportion Var 0.40
##
## Mean item complexity =  1
## Test of the hypothesis that 1 factor is sufficient.
##
## The degrees of freedom for the null model are  3  and the objective function was  0.22 with Chi Squa
## The degrees of freedom for the model are 0  and the objective function was  0
##
## The root mean square of the residuals (RMSR) is  0.02
## The df corrected root mean square of the residuals is  NA
##
## The harmonic number of observations is  561 with the empirical chi square  1.5  with prob <  NA
## The total number of observations was  596  with Likelihood Chi Square =  1.37  with prob <  NA
##
## Tucker Lewis Index of factoring reliability =  -Inf
## Fit based upon off diagonal values = 0.99
```

Graded-Response Model: Irritability

```
##      Extrmt1 Extrmt2 Extrmt3 Extrmt4 Extrmt5 Extrmt6 Dscrmn
## Q37   -2.095  -0.800  -0.173   0.657   1.547   2.430  0.938
## Q114  -0.526   0.705   1.549   2.589   3.628   4.320  0.700
## Q65   -0.217   0.560   0.992   1.426   1.853   2.076  3.688
```

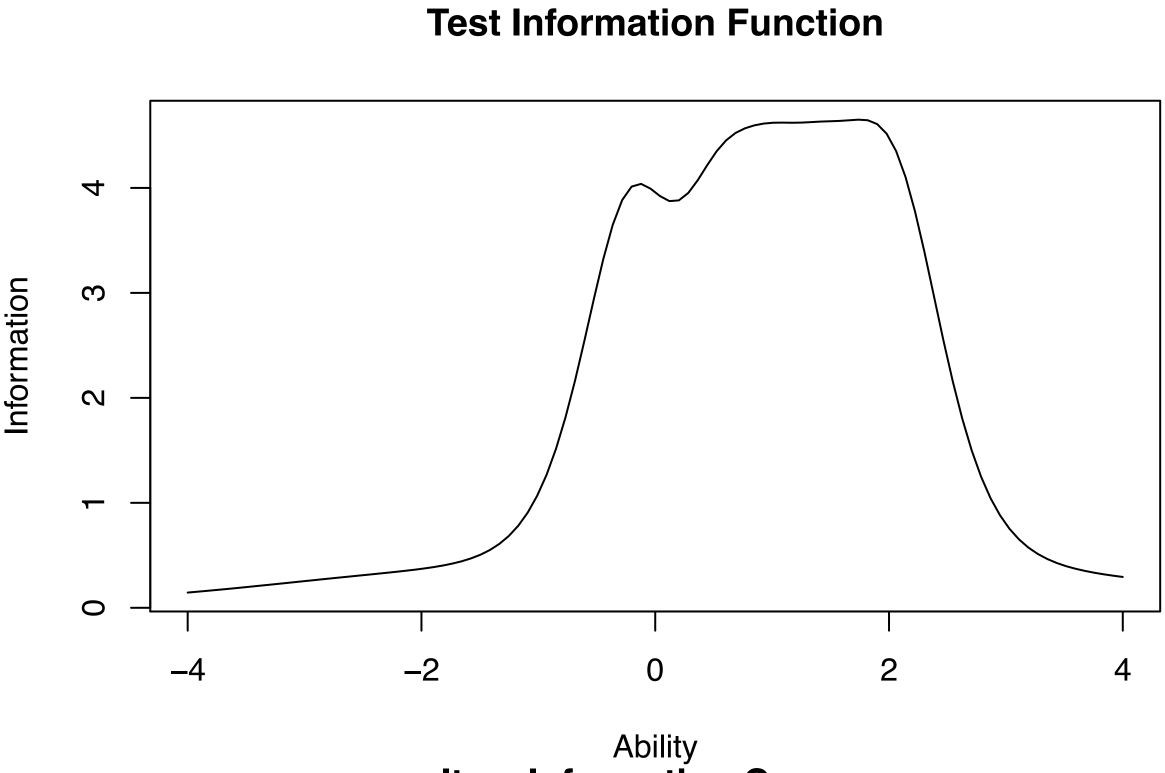

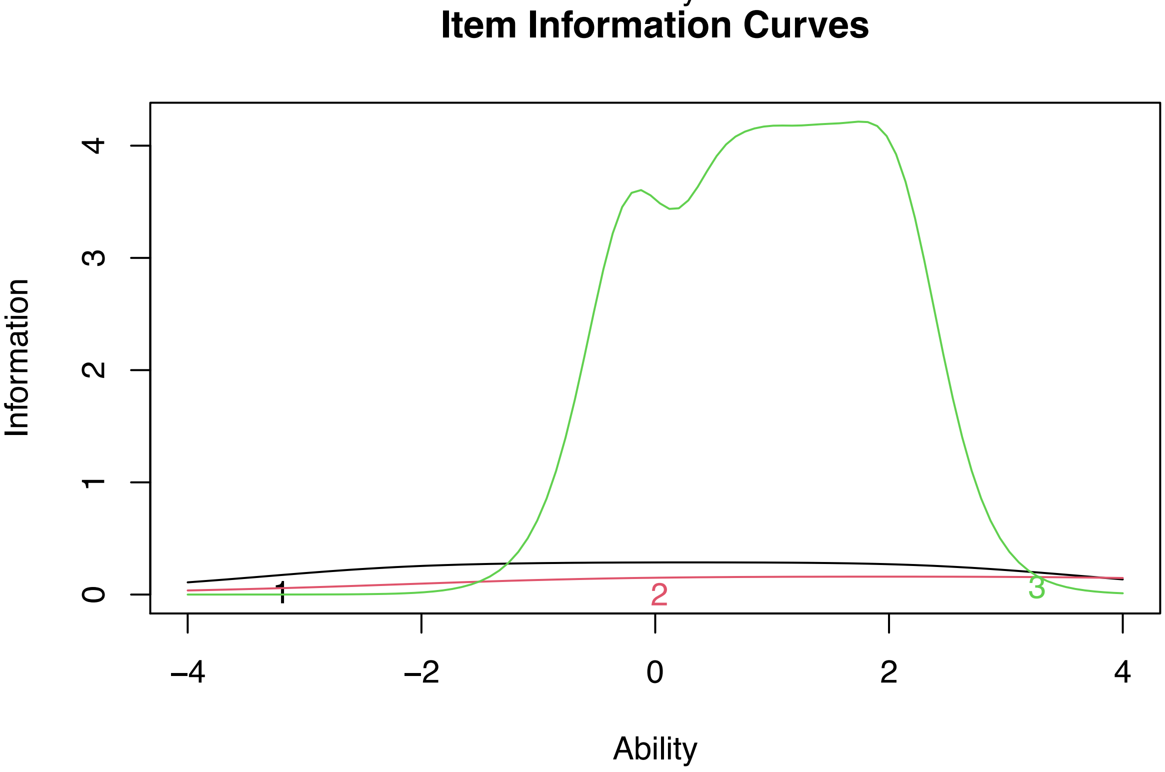

Back to top

Gender-based DIF: Irritability

```
## No Gender-based DIF detected
```

Age-based DIF: Irritability

```
## No age-based DIF detected
```

Back to top

Self-Criticism

Site 1

Reliability: Self-Criticism

```
## Cronbach's alpha is 0.887.
## Mean item-total correlation is 0.492.
## If each item were dropped:
##      raw_alpha std.alpha G6(smc) average_r S/N alpha se  var.r med.r
## Q67       0.88      0.88    0.88      0.52 7.5   0.0072 0.0082  0.50
## Q38       0.88      0.88    0.87      0.50 7.0   0.0074 0.0101  0.49
## Q126      0.86      0.86    0.86      0.47 6.3   0.0082 0.0087  0.49
## Q124      0.88      0.88    0.87      0.51 7.4   0.0071 0.0092  0.50
## Q129      0.88      0.88    0.87      0.50 7.1   0.0073 0.0115  0.50
## Q127      0.87      0.87    0.86      0.49 6.7   0.0078 0.0103  0.49
## Q128      0.86      0.86    0.85      0.47 6.1   0.0084 0.0074  0.47
## Q101      0.86      0.86    0.85      0.47 6.3   0.0083 0.0084  0.48
```

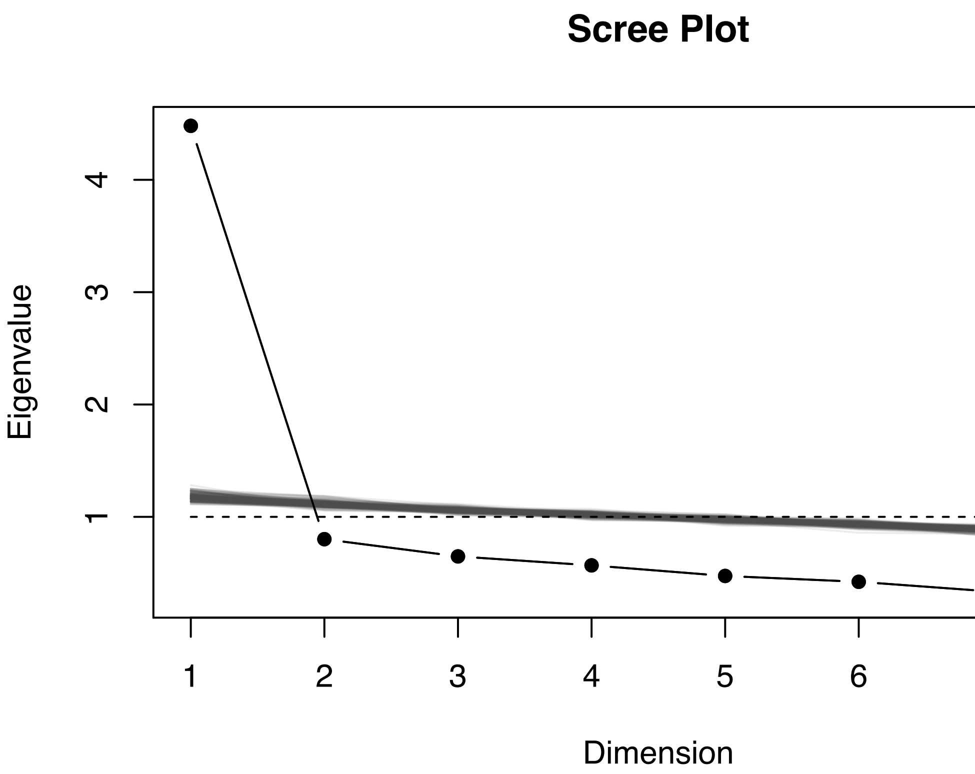

Unidimensionality: Self-Criticism

```
## [1] "Ratio of first to second eigenvalues: 5.592"

## [1] 4.4803988 0.8012562 0.6479159 0.5681547 0.4740296 0.4219289 0.3351470
## [8] 0.2711690

## Factor Analysis using method =  minres
## Call: fa(r = grm_obj$X)
## Standardized loadings (pattern matrix) based upon correlation matrix
##       MR1   h2   u2 com
## Q67  0.58 0.34 0.66   1
## Q38  0.66 0.44 0.56   1
## Q126 0.79 0.63 0.37   1
## Q124 0.59 0.34 0.66   1
## Q129 0.64 0.41 0.59   1
## Q127 0.72 0.52 0.48   1
## Q128 0.83 0.69 0.31   1
## Q101 0.80 0.64 0.36   1
##
##                 MR1
## SS loadings    4.01
## Proportion Var 0.50
##
## Mean item complexity =  1
## Test of the hypothesis that 1 factor is sufficient.
##
## The degrees of freedom for the null model are  28  and the objective function was  3.73 with Chi Squa
## The degrees of freedom for the model are 20  and the objective function was  0.19
##
## The root mean square of the residuals (RMSR) is  0.04
## The df corrected root mean square of the residuals is  0.05
##
```

```
## The harmonic number of observations is  580 with the empirical chi square  59.83  with prob <  7.6e-[illegible]
## The total number of observations was  617  with Likelihood Chi Square =  117.19  with prob <  9.4e-1[illegible]
##
## Tucker Lewis Index of factoring reliability =  0.94
## RMSEA index =  0.089  and the 90 % confidence intervals are  0.074 0.105
## BIC =  -11.31
## Fit based upon off diagonal values = 0.99
## Measures of factor score adequacy
##                                                    MR1
## Correlation of (regression) scores with factors   0.95
## Multiple R square of scores with factors          0.90
## Minimum correlation of possible factor scores     0.80
```

Graded-Response Model: Self-Criticism

```
##      Extrmt1 Extrmt2 Extrmt3 Extrmt4 Extrmt5 Extrmt6 Dscrmn
## Q67   -2.121  -1.359  -0.541   0.256   1.027   2.086  1.413
## Q38   -1.681  -0.879  -0.319   0.102   0.669   1.206  1.760
## Q126  -1.205  -0.715  -0.220   0.210   0.662   1.205  2.636
## Q124  -1.960  -0.659  -0.016   0.656   1.270   2.055  1.316
## Q129  -0.528   0.131   0.476   0.887   1.421   1.944  1.694
## Q127  -0.566  -0.170   0.190   0.597   1.061   1.513  2.090
## Q128  -1.056  -0.544  -0.209   0.188   0.666   1.185  2.947
## Q101  -0.635  -0.171   0.221   0.583   0.974   1.489  2.857
```

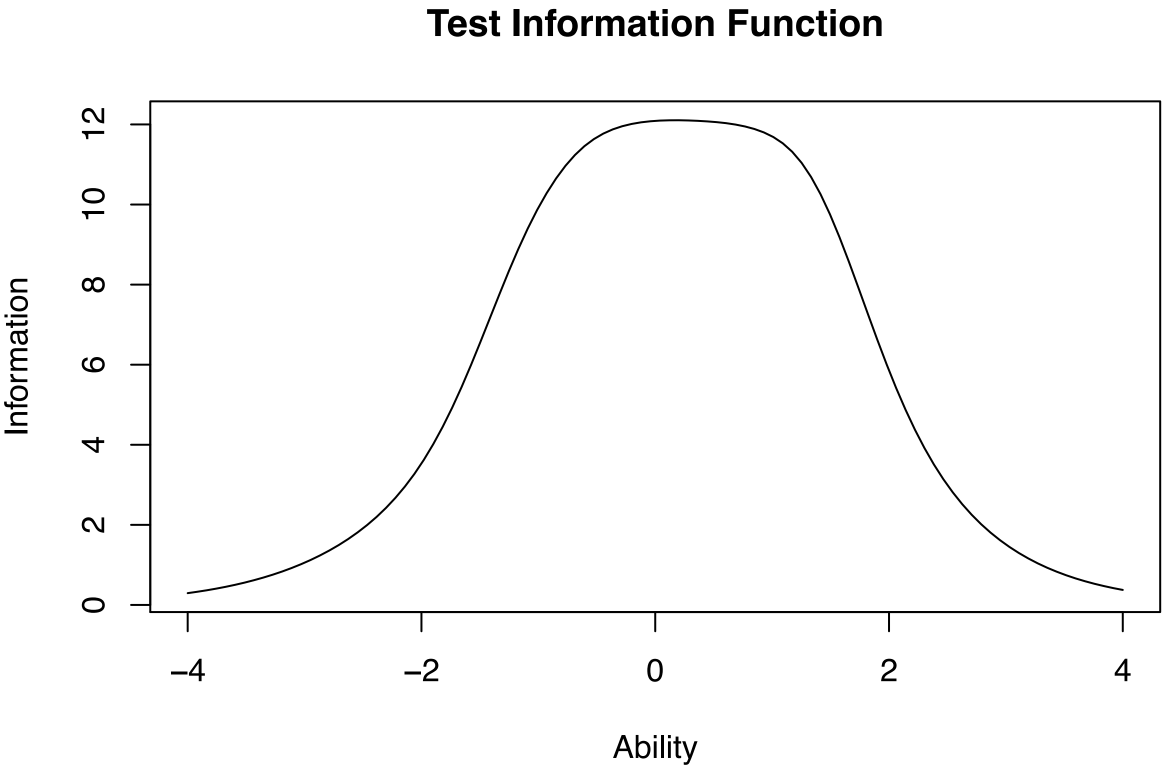

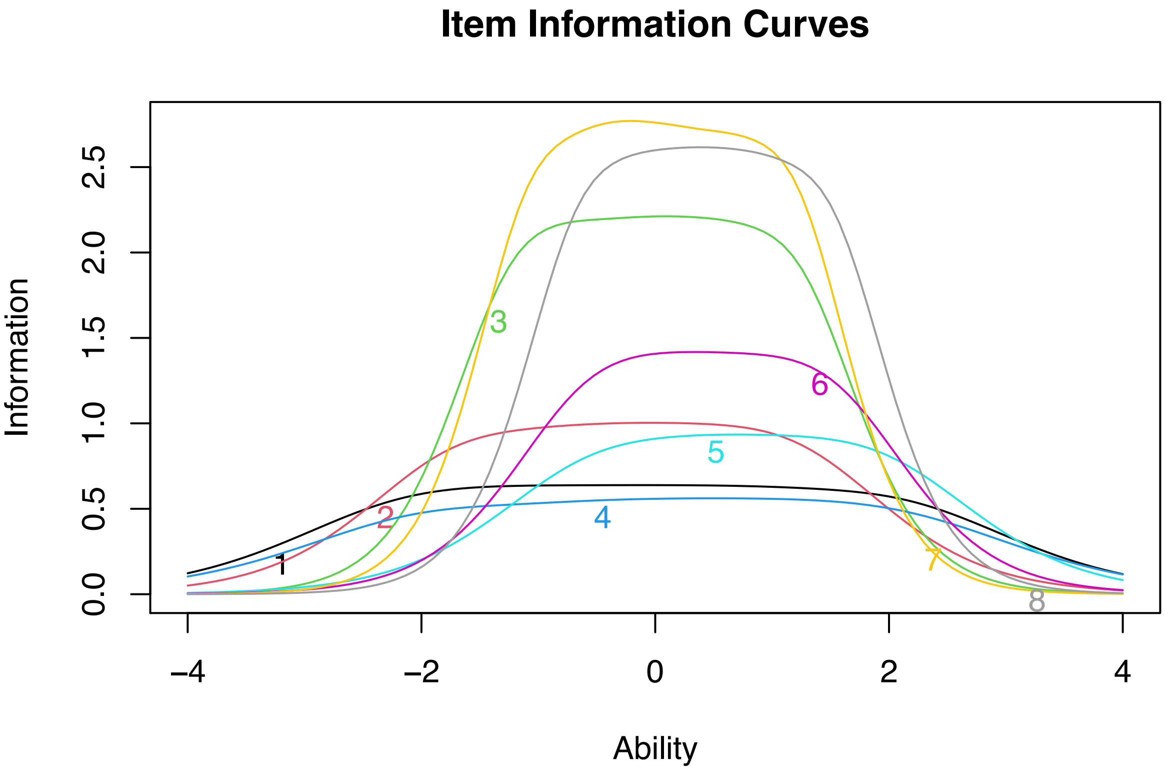

Back to top

Site 2

Reliability: Self-Criticism

```
## Cronbach's alpha is 0.901.
## Mean item-total correlation is 0.529.
## If each item were dropped:
##      raw_alpha std.alpha G6(smc) average_r S/N alpha se  var.r med.r
## Q67       0.90      0.90    0.90      0.56 8.8   0.0063 0.0074  0.57
## Q38       0.89      0.89    0.88      0.53 8.0   0.0067 0.0100  0.52
## Q126      0.88      0.88    0.87      0.51 7.2   0.0075 0.0072  0.50
## Q124      0.89      0.89    0.89      0.55 8.5   0.0065 0.0087  0.56
## Q129      0.89      0.89    0.88      0.53 7.9   0.0069 0.0105  0.52
## Q127      0.89      0.89    0.88      0.54 8.1   0.0067 0.0088  0.52
## Q128      0.88      0.88    0.87      0.51 7.2   0.0075 0.0072  0.50
## Q101      0.88      0.88    0.88      0.51 7.4   0.0073 0.0089  0.52
```

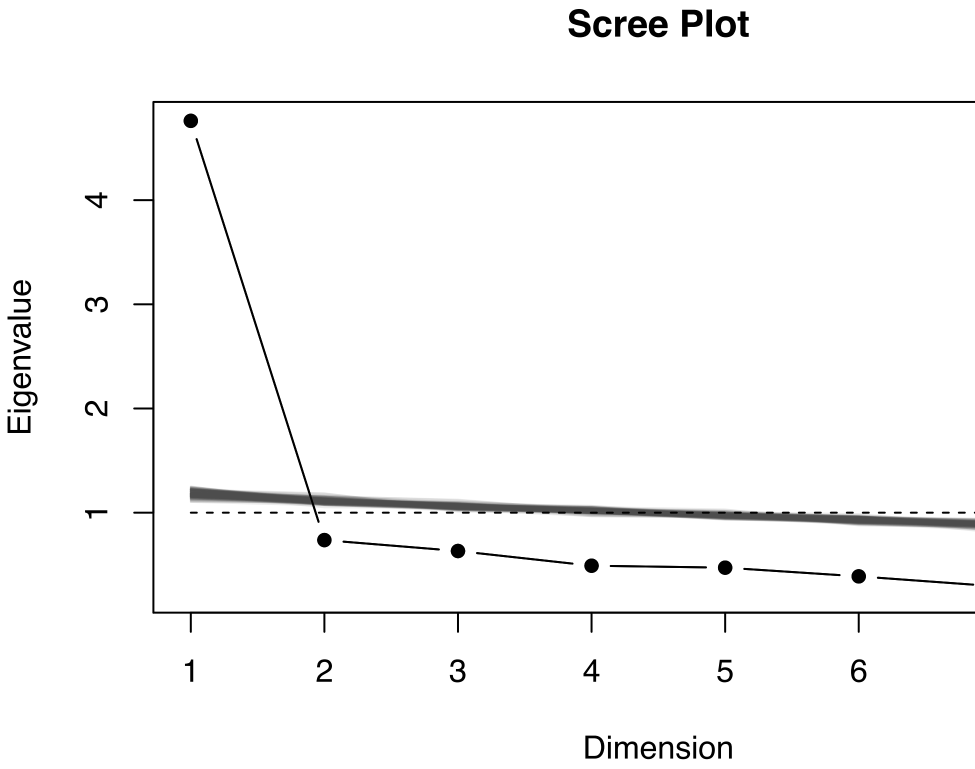

Unidimensionality: Self-Criticism

```
## [1] "Ratio of first to second eigenvalues: 6.461"

## [1] 4.7610683 0.7368737 0.6321382 0.4909212 0.4723034 0.3888566 0.2959108
## [8] 0.2219279

## Factor Analysis using method =  minres
## Call: fa(r = grm_obj$X)
## Standardized loadings (pattern matrix) based upon correlation matrix
##       MR1   h2   u2 com
## Q67  0.60 0.36 0.64   1
## Q38  0.70 0.49 0.51   1
## Q126 0.84 0.70 0.30   1
## Q124 0.64 0.41 0.59   1
## Q129 0.72 0.52 0.48   1
## Q127 0.70 0.49 0.51   1
## Q128 0.84 0.70 0.30   1
## Q101 0.81 0.65 0.35   1
##
##                 MR1
## SS loadings    4.33
## Proportion Var 0.54
##
## Mean item complexity =  1
## Test of the hypothesis that 1 factor is sufficient.
##
## The degrees of freedom for the null model are  28  and the objective function was  4.33 with Chi Squa
## The degrees of freedom for the model are 20  and the objective function was  0.26
##
## The root mean square of the residuals (RMSR) is  0.04
## The df corrected root mean square of the residuals is  0.05
##
```

```
## The harmonic number of observations is  561 with the empirical chi square  60.11  with prob <  6.8e-0
## The total number of observations was  596  with Likelihood Chi Square =  156.05  with prob <  4.3e-23
##
## Tucker Lewis Index of factoring reliability =  0.925
## RMSEA index =  0.107  and the 90 % confidence intervals are  0.092 0.123
## BIC =  28.24
## Fit based upon off diagonal values = 0.99
## Measures of factor score adequacy
##                                                    MR1
## Correlation of (regression) scores with factors   0.96
## Multiple R square of scores with factors          0.91
## Minimum correlation of possible factor scores     0.83
```

Graded-Response Model: Self-Criticism

```
##      Extrmt1 Extrmt2 Extrmt3 Extrmt4 Extrmt5 Extrmt6 Dscrmn
## Q67   -1.752  -0.985  -0.248   0.522   1.290   2.241  1.537
## Q38   -0.937  -0.215   0.190   0.623   1.069   1.627  1.943
## Q126  -0.660  -0.185   0.235   0.635   1.076   1.514  3.248
## Q124  -1.026  -0.066   0.531   1.125   1.659   2.276  1.696
## Q129   0.016   0.543   0.867   1.218   1.622   1.996  2.355
## Q127  -0.056   0.420   0.700   1.019   1.389   1.869  2.219
## Q128  -0.583  -0.074   0.298   0.714   1.058   1.499  3.337
## Q101  -0.156   0.232   0.602   1.001   1.432   1.870  2.994
```

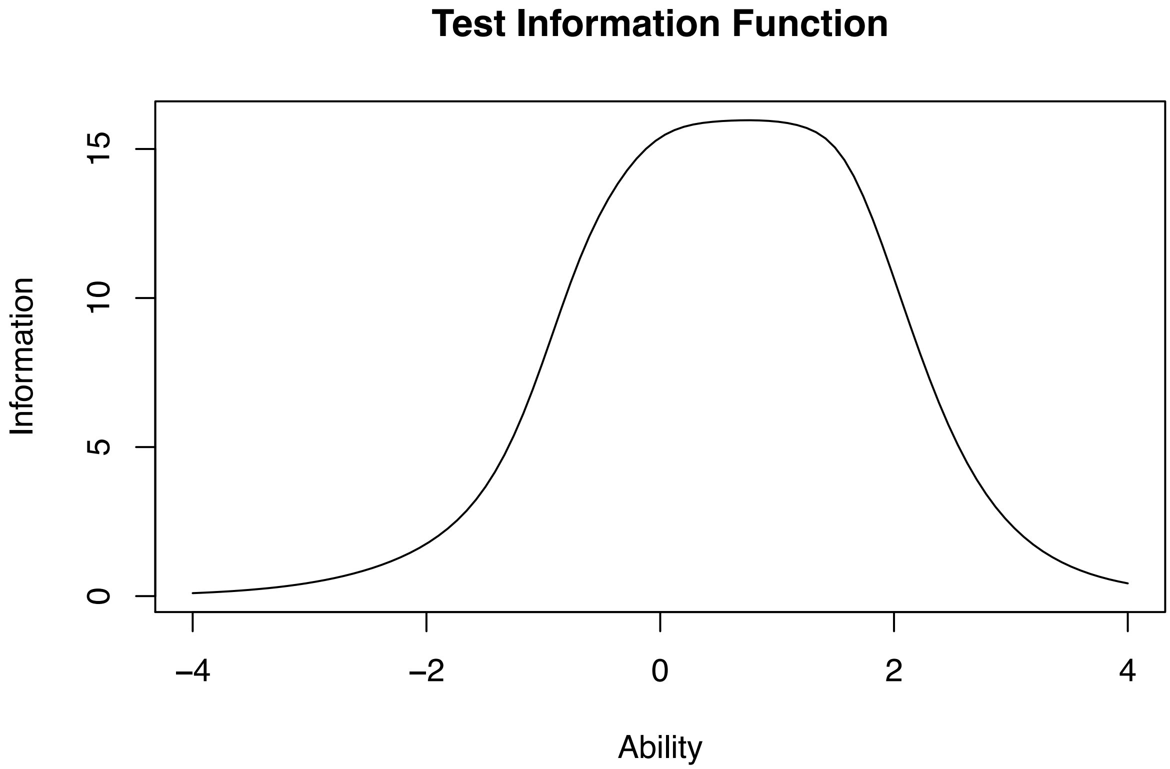

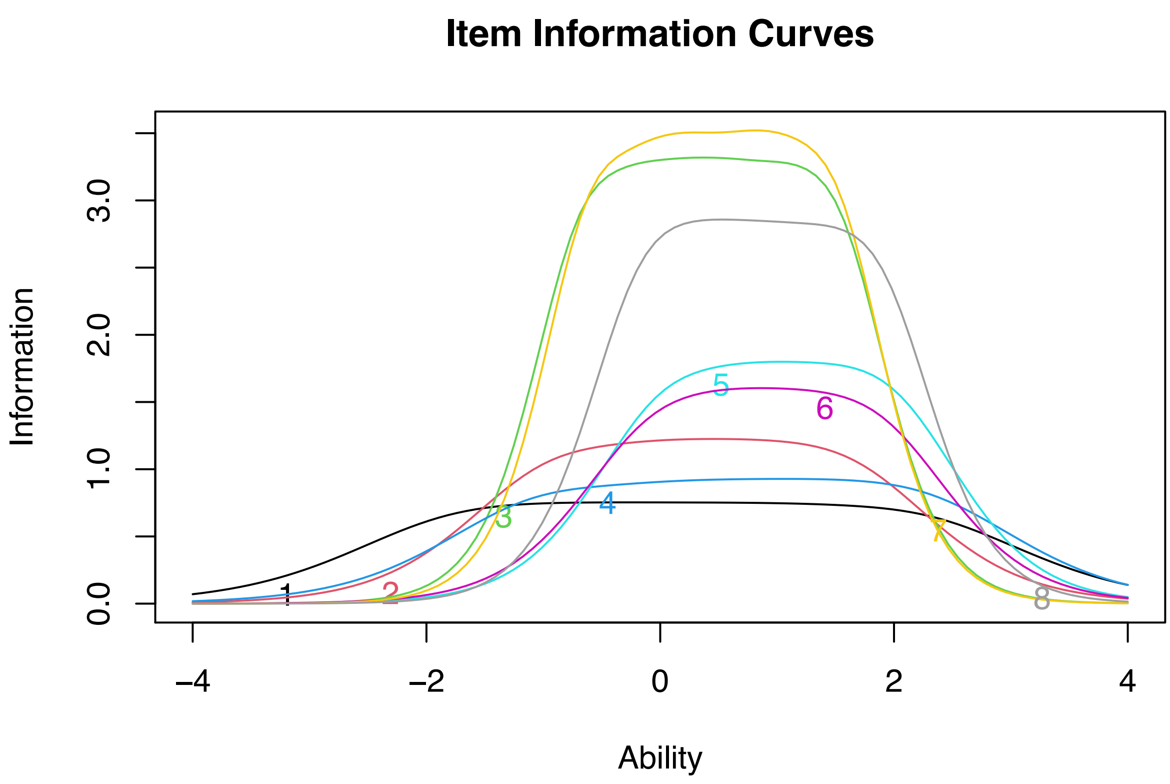

Back to top

Site DIF

```
## Call:
## lordif::lordif(resp.data = as.data.frame(merged_data), group = site)
##
##   Number of DIF groups: 2
##
##   Number of items flagged for DIF: 1 of 8
##
##   Items flagged: 1
##
##   Number of iterations for purification: 2 of 10
##
##   Detection criterion: Chisqr
##
##   Threshold: alpha = 0.01
##
##   item ncat  chi12  chi13  chi23
## 1    1    7 0.0004 0.0018 0.7988
## 2    2    7 0.2185 0.2951 0.3357
## 3    3    7 0.6728 0.6772 0.4381
## 4    4    7 0.1250 0.1393 0.2076
## 5    5    7 0.8057 0.6126 0.3376
## 6    6    7 0.7231 0.9270 0.8717
## 7    7    7 0.4443 0.6562 0.6118
## 8    8    7 0.4284 0.3017 0.1835
```

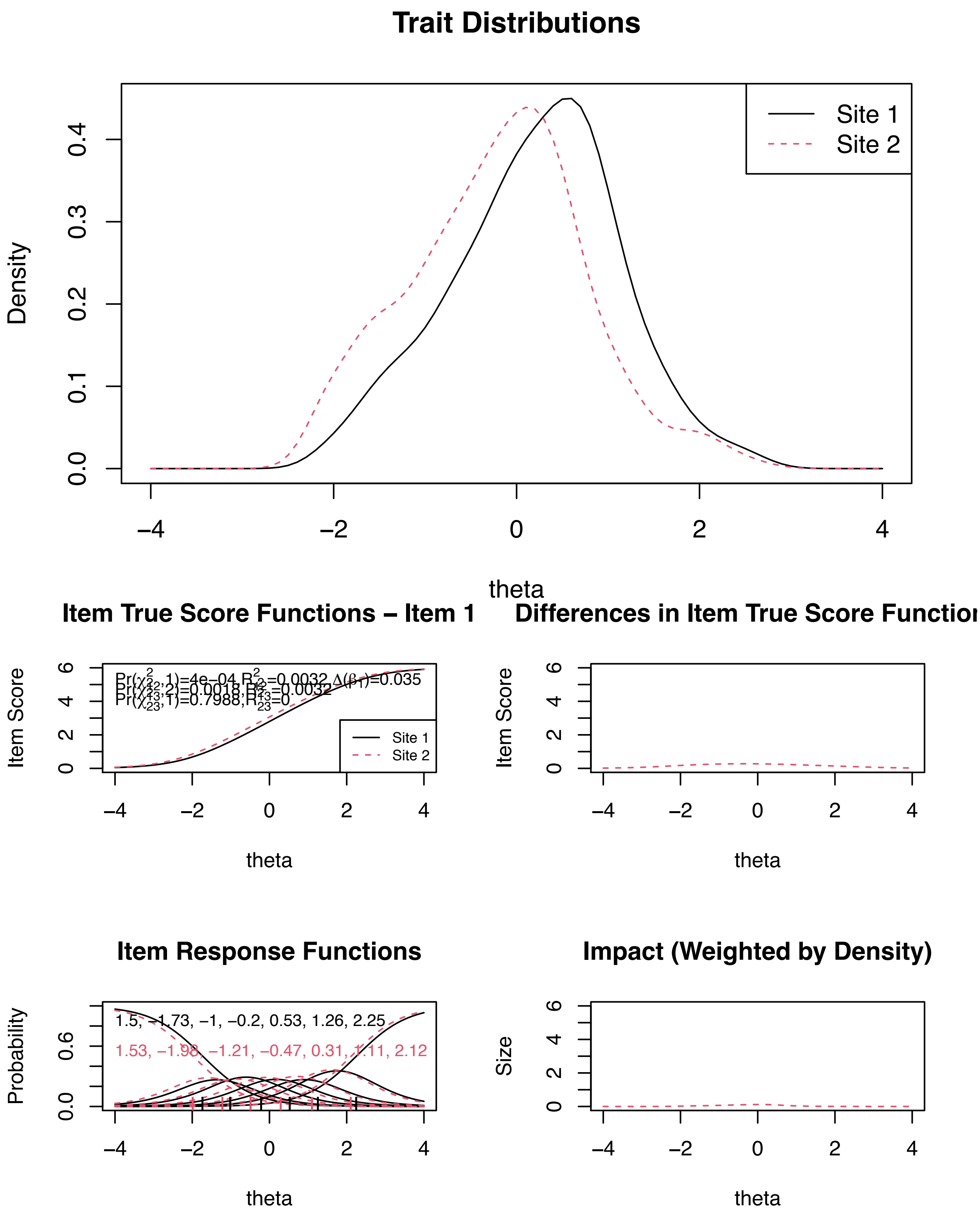
Trait Distributions
Site 1
Site 2
Density
theta
Item True Score Functions – Item 1
Site 1
Site 2
Item Score
theta
Differences in Item True Score Functio
Item Score
theta
Item Response Functions
1.5, −1.73, −1, −0.2, 0.53, 1.26, 2.25
1.53, −1.98, −1.21, −0.47, 0.31, 1.11, 2.12
Probability
theta
Impact (Weighted by Density)
Size
theta

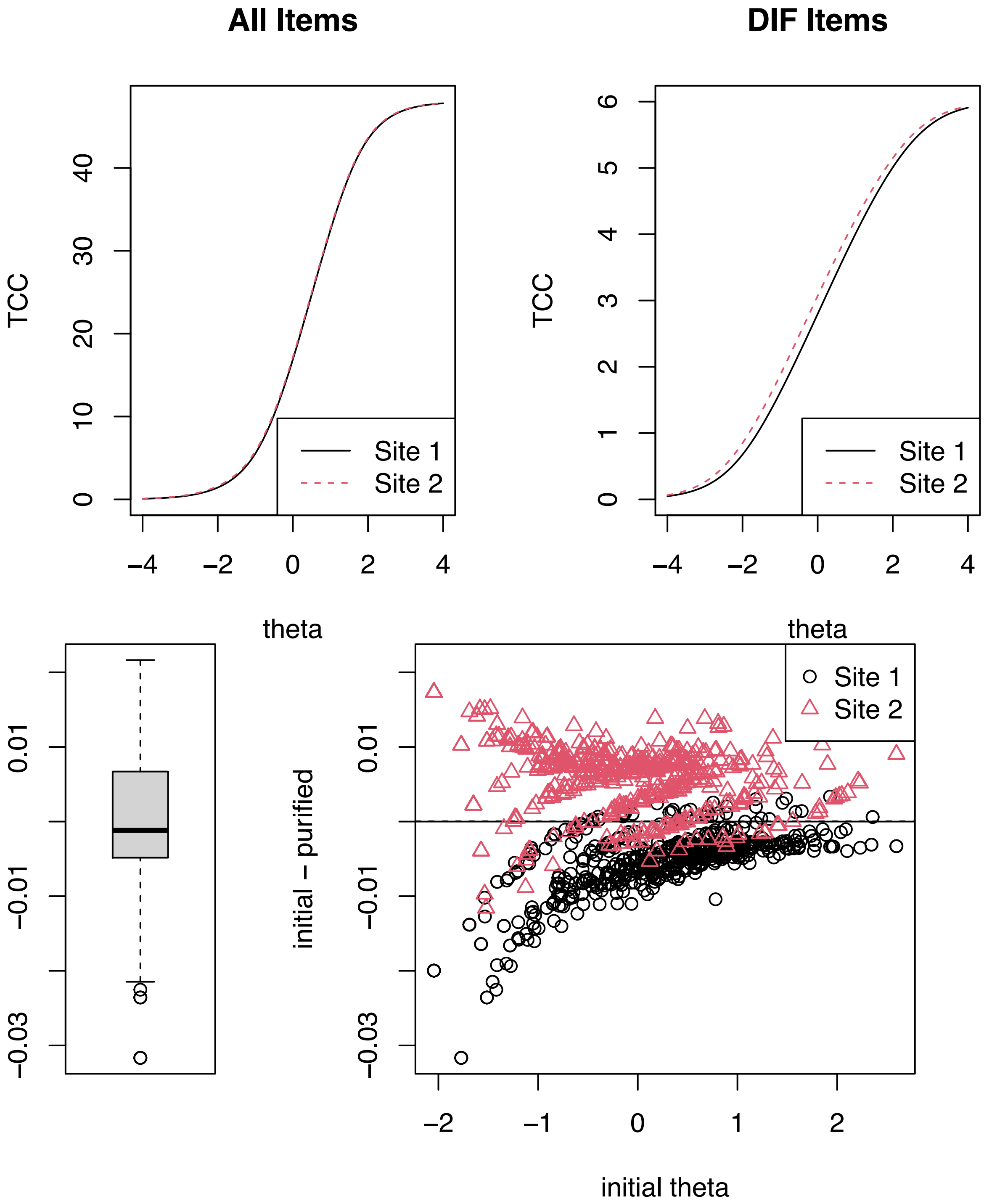

Back to top

Gender-based DIF: Self-Criticism

```
## Call:
## lordif::lordif(resp.data = as.data.frame(sex.data), group = sex)
##
##   Number of DIF groups: 2
```

```
##
##   Number of items flagged for DIF: 0 of 8
##
##   Items flagged:
##
##   Number of iterations for purification: 1 of 10
##
##   Detection criterion: Chisqr
##
##   Threshold: alpha = 0.01
```

Age-based DIF: Self-Criticism

```
## Call:
## lordif::lordif(resp.data = as.data.frame(age.data), group = age)
##
##   Number of DIF groups: 2
##
##   Number of items flagged for DIF: 3 of 8
##
##   Items flagged: 2, 4, 8
##
##   Number of iterations for purification: 3 of 10
##
##   Detection criterion: Chisqr
##
##   Threshold: alpha = 0.01
##
##   item ncat  chi12  chi13  chi23
## 1    1    7 0.9889 0.2715 0.1064
## 2    2    7 0.0021 0.0020 0.0867
## 3    3    7 0.2060 0.4493 0.9805
## 4    4    7 0.3001 0.0013 0.0005
## 5    5    7 0.0638 0.1449 0.5139
## 6    6    7 0.1421 0.2190 0.3474
## 7    7    7 0.0197 0.0625 0.7424
## 8    8    7 0.0009 0.0036 0.6409
```

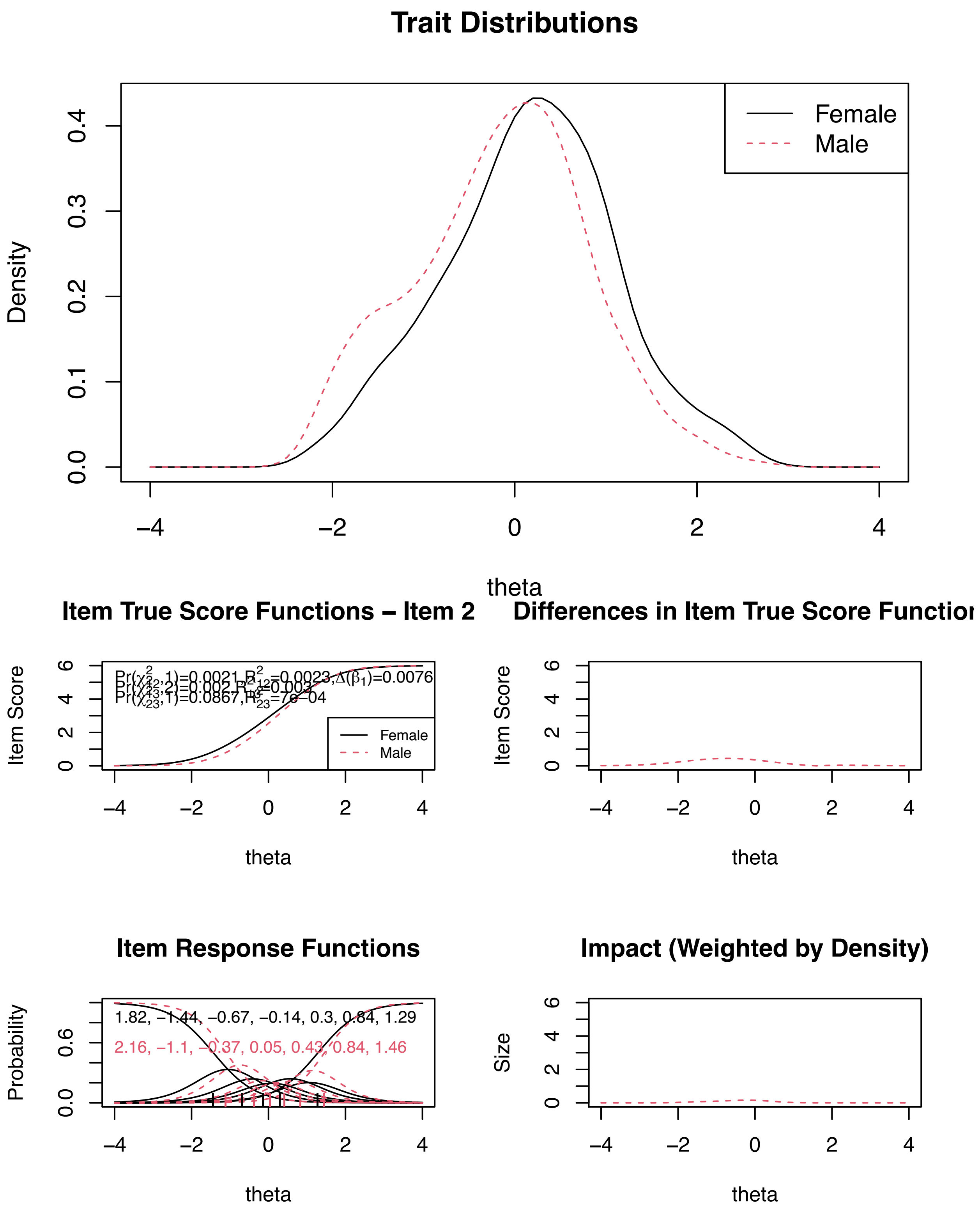
Trait Distributions
Density
theta
Female
Male
Item True Score Functions – Item 2
Pr(χ12², 1)=0.0021, R12²=0.0023, Δ(β1)=0.0076
Pr(χ13², 2)=0.002, R13²=0.003
Pr(χ23², 1)=0.0867, R23²=7e−04
Item Score
Female
Male
Differences in Item True Score Functio
Item Response Functions
Probability
1.82, −1.44, −0.67, −0.14, 0.3, 0.84, 1.29
2.16, −1.1, −0.37, 0.05, 0.43, 0.84, 1.46
Impact (Weighted by Density)
Size

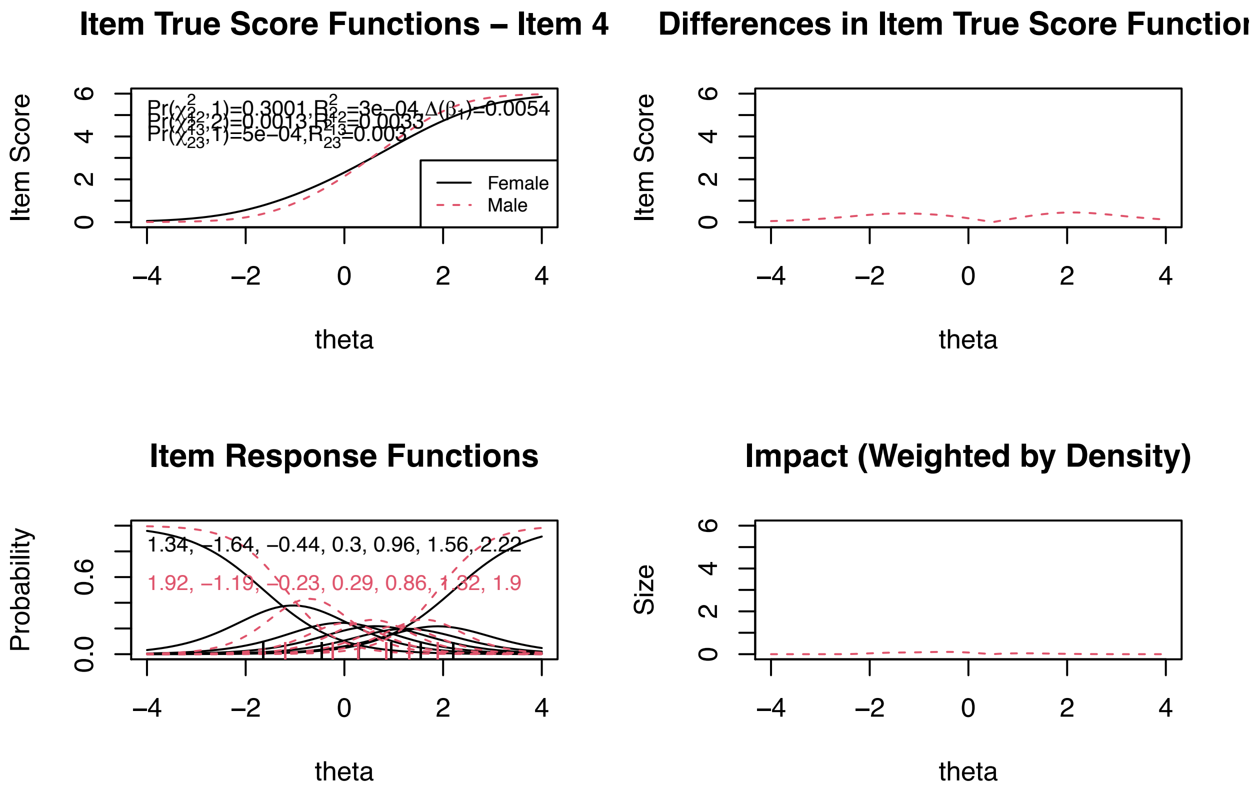
Item True Score Functions – Item 4
Differences in Item True Score Functio
Item Score
theta
Female
Male
Item Response Functions
Impact (Weighted by Density)
Probability
Size
1.34, –1.64, –0.44, 0.3, 0.96, 1.56, 2.22
1.92, –1.19, –0.23, 0.29, 0.86, 1.32, 1.9

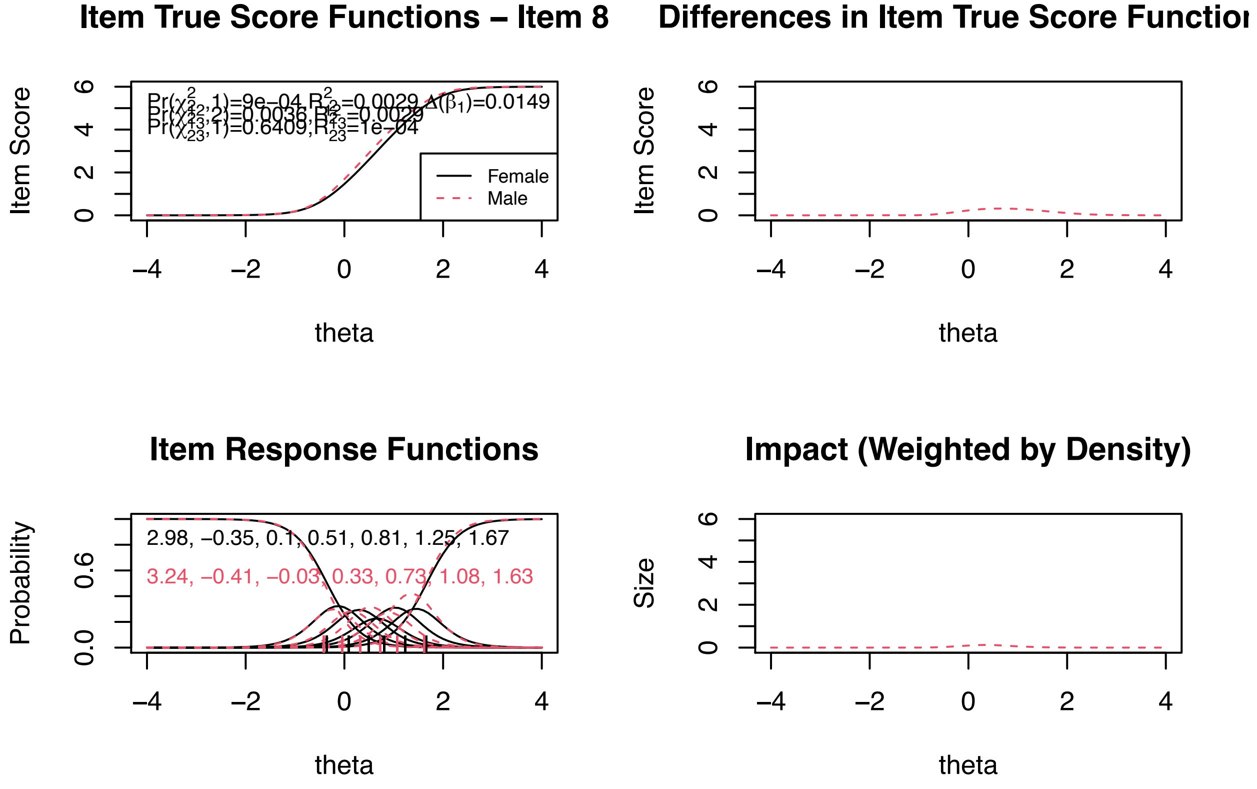
Item True Score Functions – Item 8
Differences in Item True Score Functio
Item Score
theta
Female
Male
Item Response Functions
Impact (Weighted by Density)
Probability
Size
2.98, –0.35, 0.1, 0.51, 0.81, 1.25, 1.67
3.24, –0.41, –0.03, 0.33, 0.73, 1.08, 1.63

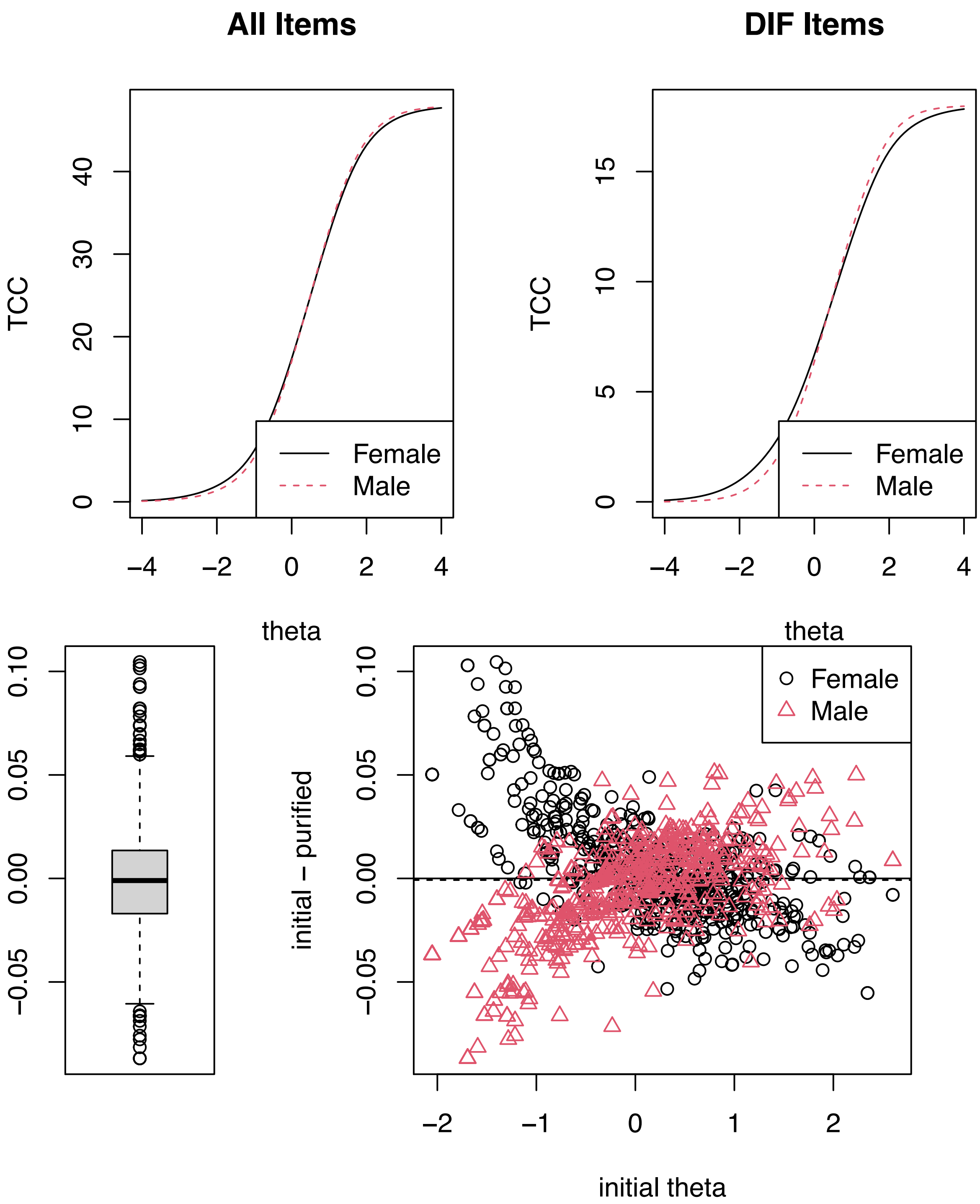

Back to top

Situational Avoidance

Site 1

Reliability: Situational Avoidance

```
## Cronbach's alpha is 0.645.
## Mean item-total correlation is 0.377.
## If each item were dropped:
##      raw_alpha std.alpha G6(smc) average_r  S/N alpha se var.r med.r
## Q119      0.50      0.50    0.34      0.34 1.01    0.040    NA  0.34
## Q17       0.68      0.68    0.52      0.52 2.16    0.026    NA  0.52
## Q118      0.43      0.43    0.28      0.28 0.77    0.045    NA  0.28
```

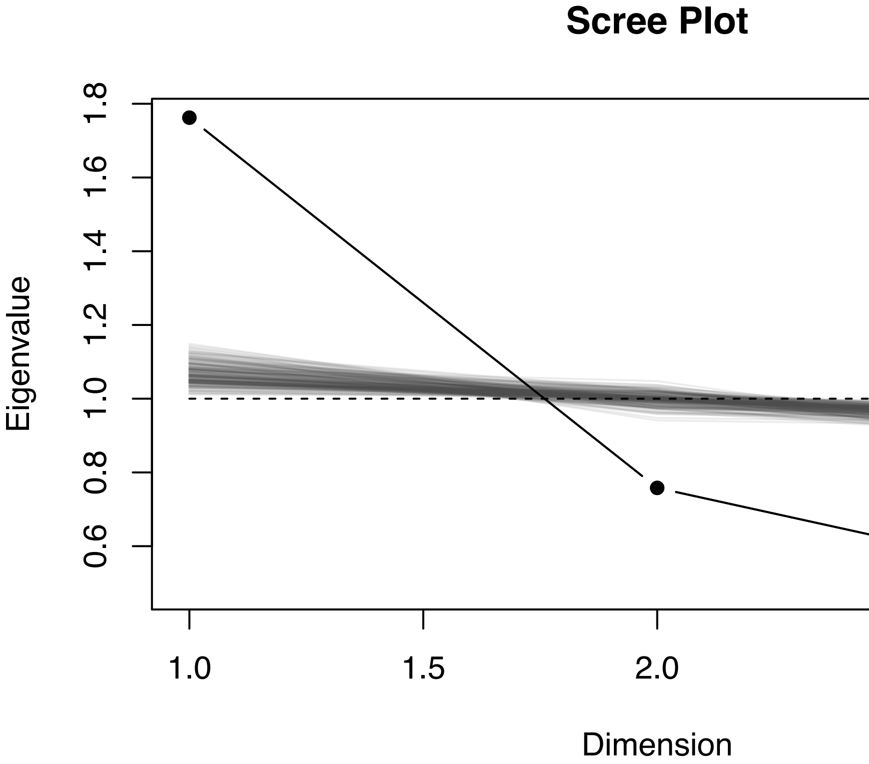

Unidimensionality: Situational Avoidance

```
## [1] "Ratio of first to second eigenvalues: 2.325"
```

```
## [1] 1.7623073 0.7580802 0.4796126
```

```
## Factor Analysis using method =  minres
## Call: fa(r = grm_obj$X)
## Standardized loadings (pattern matrix) based upon correlation matrix
##       MR1   h2   u2 com
## Q119 0.65 0.43 0.57   1
## Q17  0.42 0.18 0.82   1
## Q118 0.79 0.62 0.38   1
##
##                 MR1
## SS loadings    1.23
## Proportion Var 0.41
##
## Mean item complexity =  1
## Test of the hypothesis that 1 factor is sufficient.
##
## The degrees of freedom for the null model are  3  and the objective function was  0.45 with Chi Squa
## The degrees of freedom for the model are 0  and the objective function was  0
##
```

```
## The root mean square of the residuals (RMSR) is  0
## The df corrected root mean square of the residuals is  NA
##
## The harmonic number of observations is  585 with the empirical chi square  0  with prob <  NA
## The total number of observations was  617  with Likelihood Chi Square =  0  with prob <  NA
##
## Tucker Lewis Index of factoring reliability =  -Inf
## Fit based upon off diagonal values = 1
## Measures of factor score adequacy
##                                                    MR1
## Correlation of (regression) scores with factors   0.85
## Multiple R square of scores with factors          0.72
## Minimum correlation of possible factor scores     0.45
```

Graded-Response Model: Situational Avoidance

```
##      Extrmt1 Extrmt2 Extrmt3 Extrmt4 Extrmt5 Extrmt6 Dscrmn
## Q119   1.017   0.258  -0.119  -0.597  -1.036  -1.438 -1.615
## Q17    0.071  -0.774  -1.079  -1.540  -1.944  -2.785 -0.986
## Q118   0.807   0.224  -0.201  -0.566  -0.950  -1.379 -2.816
```

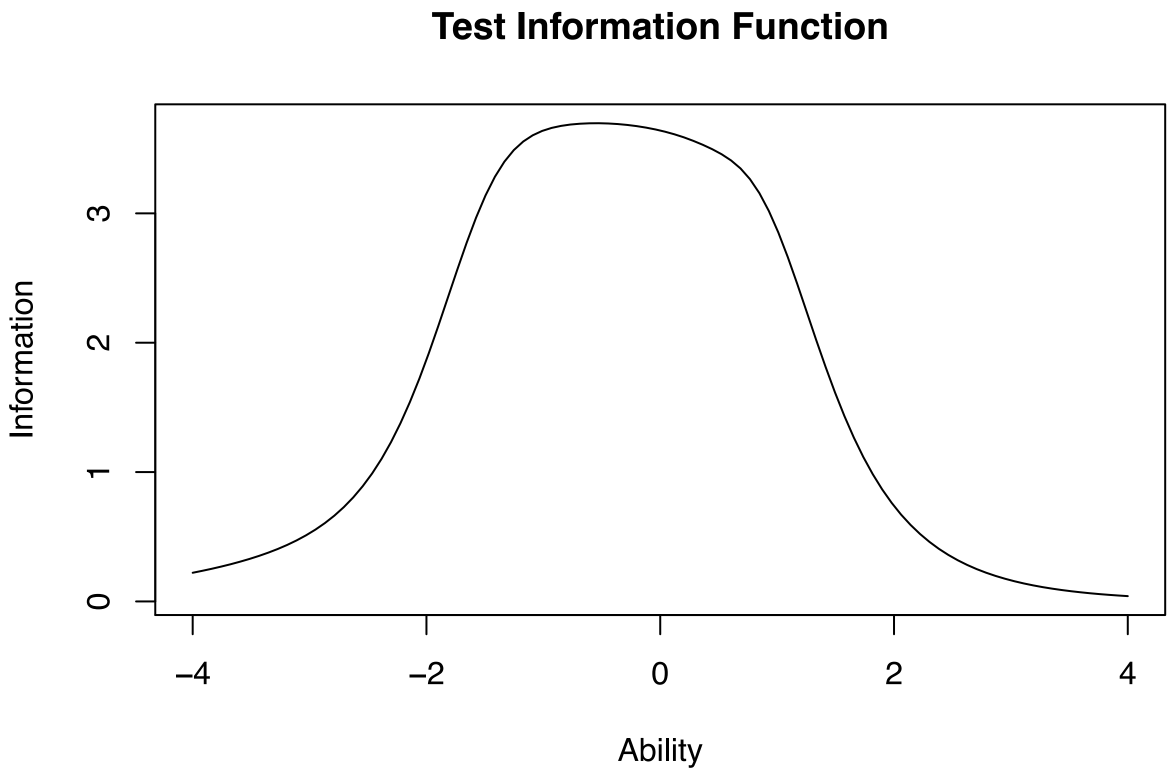

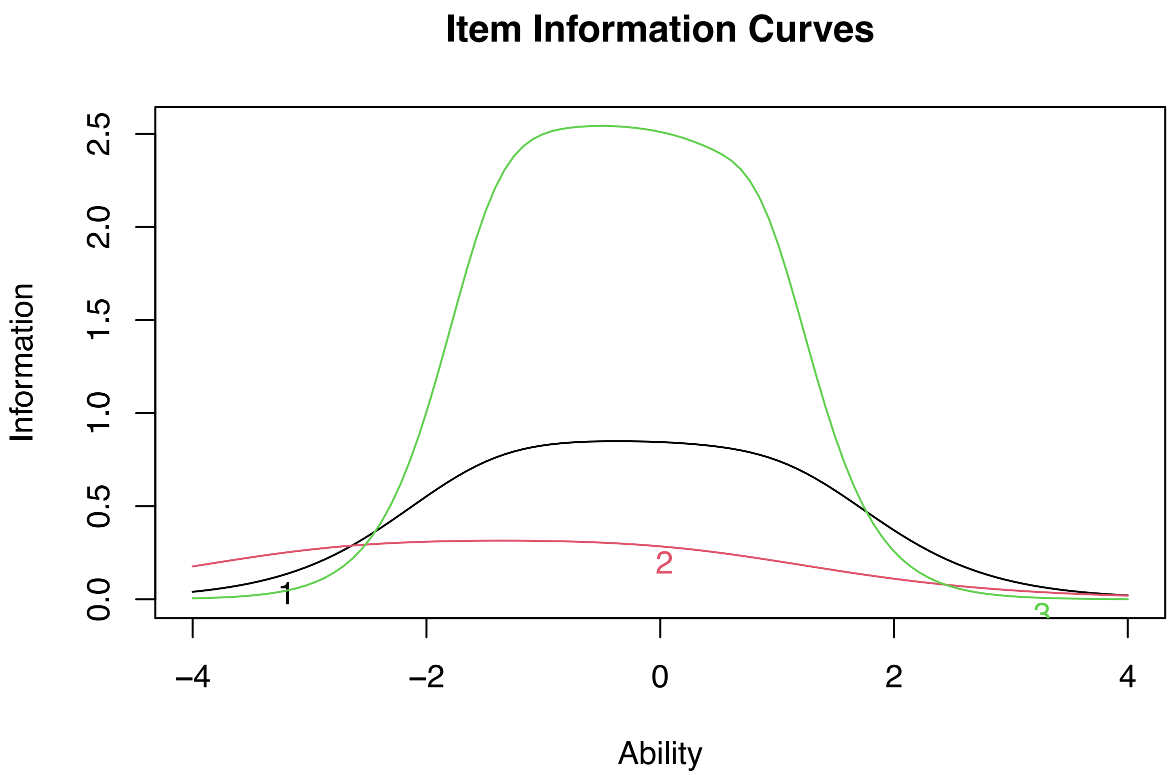

Back to top

Site 2

Reliability: Situational Avoidance

```
## Cronbach's alpha is 0.632.
## Mean item-total correlation is 0.365.
## If each item were dropped:
##      raw_alpha std.alpha G6(smc) average_r  S/N alpha se var.r med.r
## Q119      0.44      0.44    0.29      0.29 0.80    0.046    NA  0.29
## Q17       0.70      0.70    0.54      0.54 2.34    0.025    NA  0.54
## Q118      0.43      0.43    0.27      0.27 0.74    0.047    NA  0.27
```

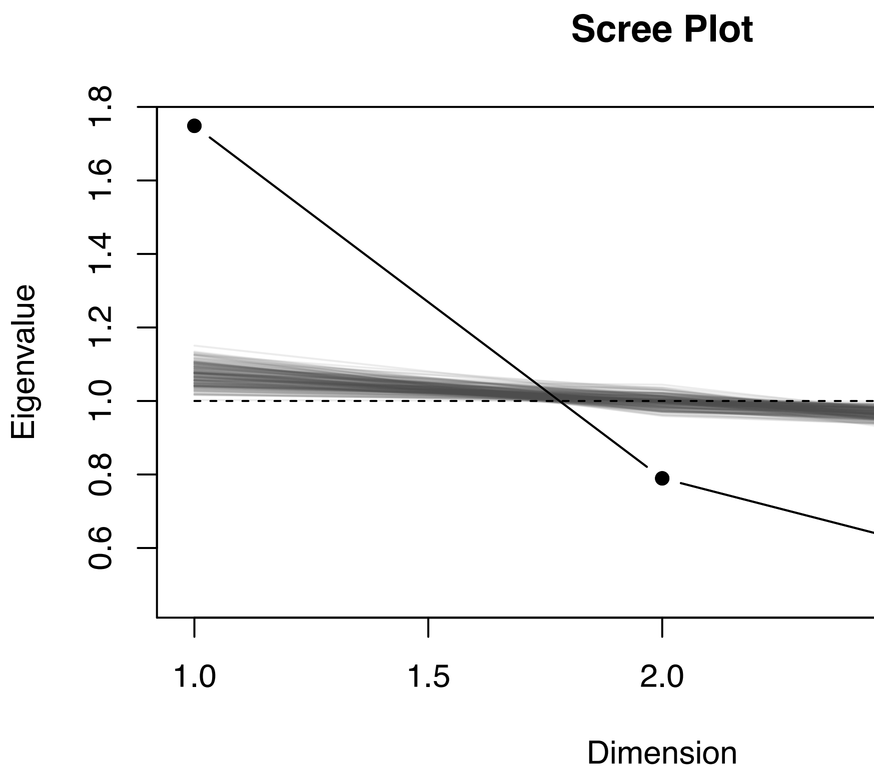

Unidimensionality: Situational Avoidance

```
## [1] "Ratio of first to second eigenvalues: 2.215"

## [1] 1.7485400 0.7895052 0.4619548

## Factor Analysis using method =  minres
## Call: fa(r = grm_obj$X)
## Standardized loadings (pattern matrix) based upon correlation matrix
##       MR1   h2   u2 com
## Q119 0.71 0.51 0.49   1
## Q17  0.38 0.15 0.85   1
## Q118 0.75 0.57 0.43   1
##
##                 MR1
## SS loadings    1.22
## Proportion Var 0.41
##
## Mean item complexity =  1
## Test of the hypothesis that 1 factor is sufficient.
##
## The degrees of freedom for the null model are  3  and the objective function was  0.45 with Chi Squa
## The degrees of freedom for the model are 0  and the objective function was  0
##
## The root mean square of the residuals (RMSR) is  0
## The df corrected root mean square of the residuals is  NA
##
## The harmonic number of observations is  564 with the empirical chi square  0  with prob <  NA
## The total number of observations was  596  with Likelihood Chi Square =  0  with prob <  NA
##
## Tucker Lewis Index of factoring reliability =  -Inf
## Fit based upon off diagonal values = 1
## Measures of factor score adequacy
```

```
##                                                   MR1
## Correlation of (regression) scores with factors   0.85
## Multiple R square of scores with factors          0.72
## Minimum correlation of possible factor scores     0.43
```

Graded-Response Model: Situational Avoidance

```
##      Extrmt1 Extrmt2 Extrmt3 Extrmt4 Extrmt5 Extrmt6 Dscrmn
## Q119   0.472  -0.048  -0.382  -0.765  -1.171  -1.507 -2.178
## Q17   -0.124  -0.938  -1.436  -1.818  -2.334  -3.146 -0.796
## Q118   0.705   0.069  -0.326  -0.741  -1.174  -1.640 -2.276
```

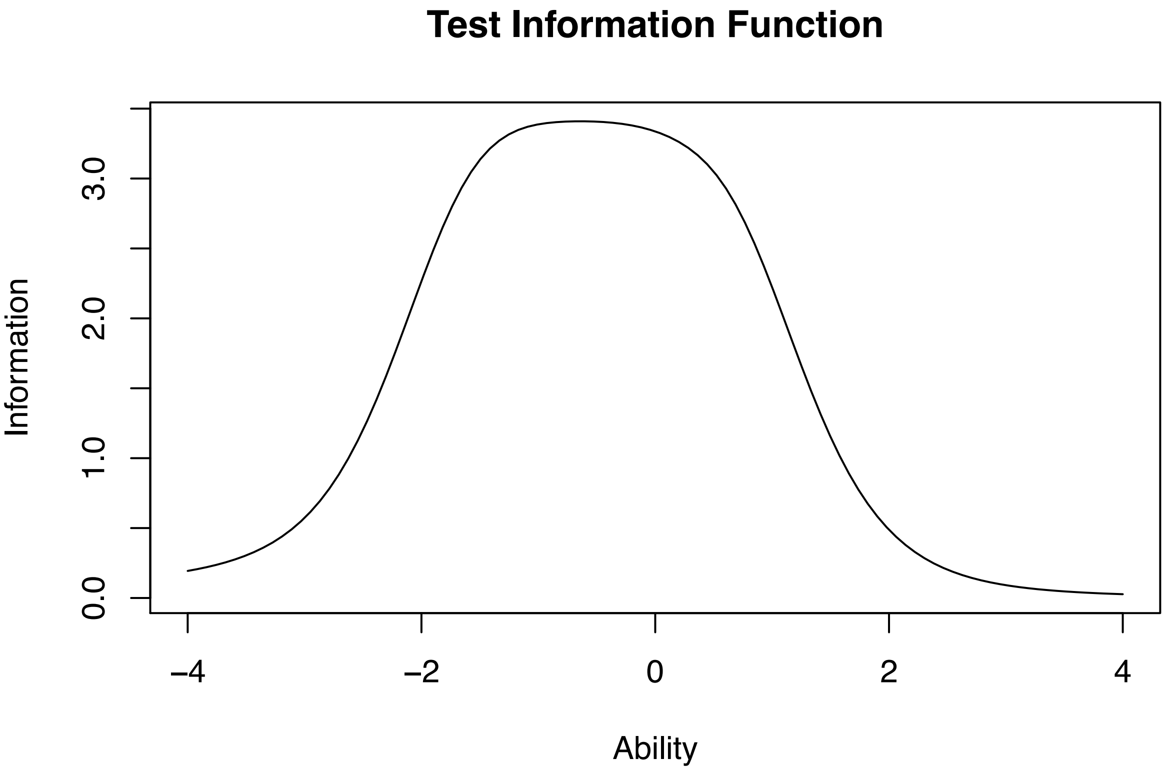

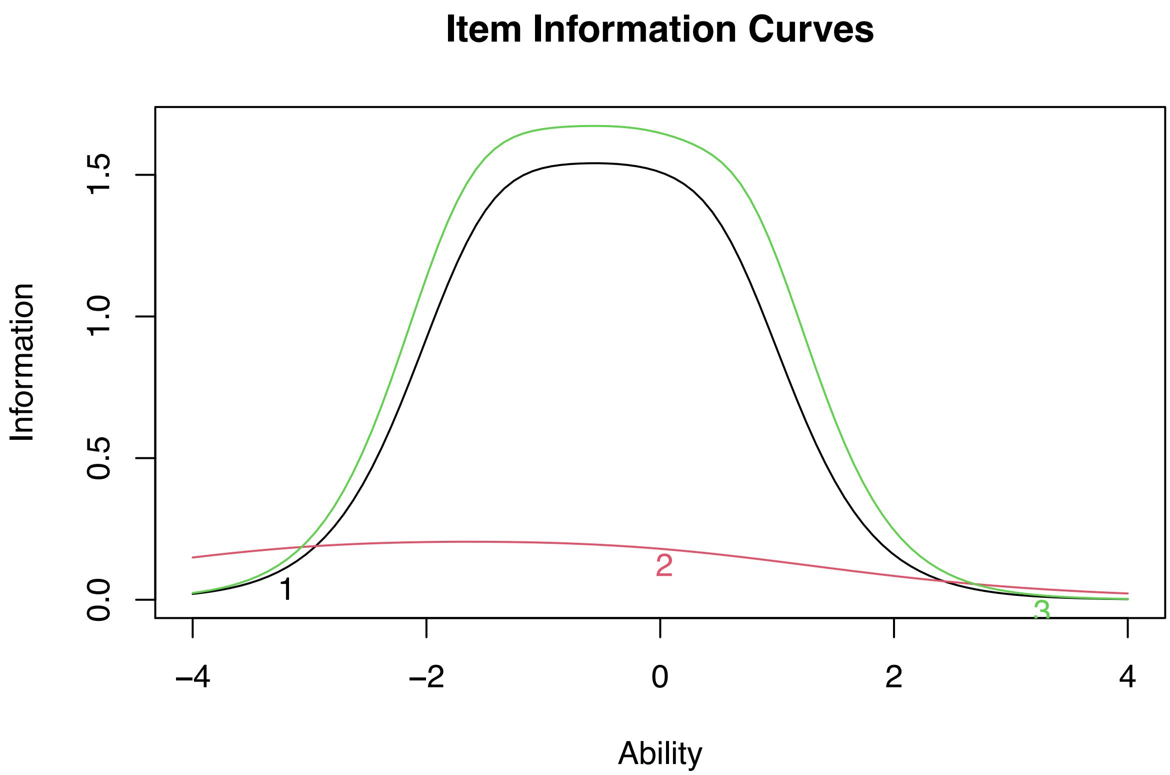

Back to top

Gender-based DIF: Situational Avoidance

```
## No Gender-based DIF detected
```

Age-based DIF: Situational Avoidance

```
## No age-based DIF detected
```

Back to top

Social Avoidance

Site 1

Reliability: Social Avoidance

```
## Cronbach's alpha is 0.747.
## Mean item-total correlation is 0.497.
## If each item were dropped:
##      raw_alpha std.alpha G6(smc) average_r S/N alpha se var.r med.r
## Q64       0.59      0.59    0.42      0.42 1.4    0.033    NA  0.42
## Q120      0.67      0.67    0.50      0.50 2.0    0.027    NA  0.50
## Q121      0.72      0.72    0.57      0.57 2.6    0.022    NA  0.57
```

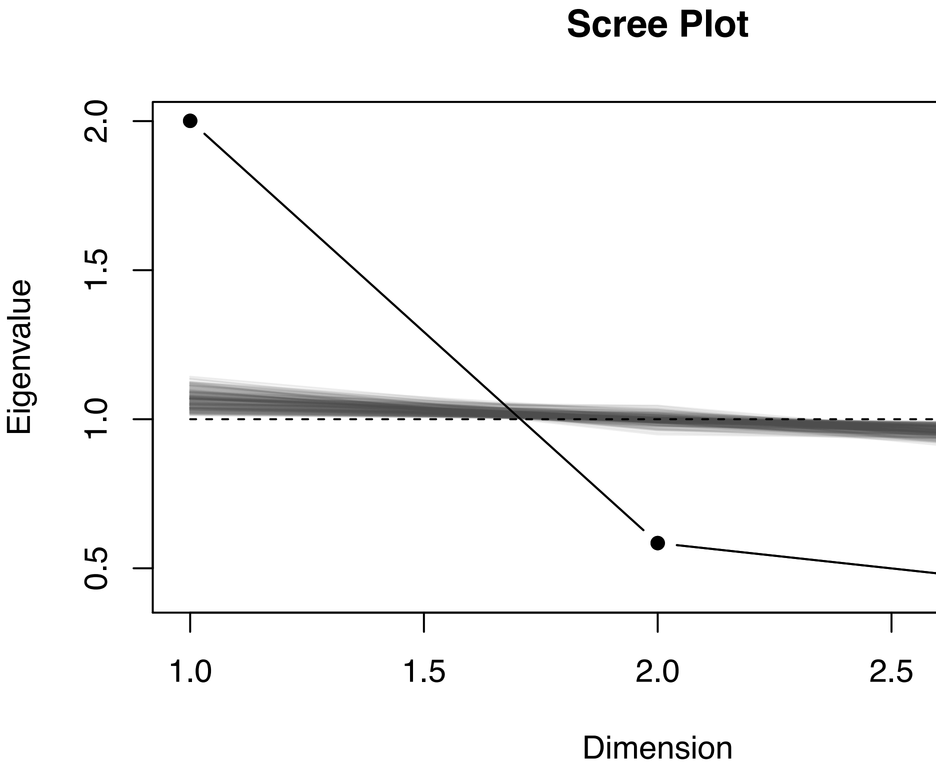

Unidimensionality: Social Avoidance

```
## [1] "Ratio of first to second eigenvalues: 3.423"
```

```
## [1] 2.0008696 0.5845459 0.4145844
```

```
## Factor Analysis using method =  minres
## Call: fa(r = grm_obj$X)
## Standardized loadings (pattern matrix) based upon correlation matrix
##       MR1   h2   u2 com
## Q64  0.83 0.68 0.32   1
## Q120 0.69 0.47 0.53   1
## Q121 0.62 0.38 0.62   1
##
##                 MR1
## SS loadings    1.53
## Proportion Var 0.51
##
## Mean item complexity =  1
## Test of the hypothesis that 1 factor is sufficient.
##
## The degrees of freedom for the null model are  3  and the objective function was  0.72 with Chi Squa
## The degrees of freedom for the model are 0  and the objective function was  0
##
## The root mean square of the residuals (RMSR) is  0
## The df corrected root mean square of the residuals is  NA
##
## The harmonic number of observations is  572 with the empirical chi square  0  with prob <  NA
## The total number of observations was  617  with Likelihood Chi Square =  0  with prob <  NA
##
## Tucker Lewis Index of factoring reliability =  -Inf
## Fit based upon off diagonal values = 1
## Measures of factor score adequacy
```

```
##                                                 MR1
## Correlation of (regression) scores with factors   0.89
## Multiple R square of scores with factors          0.79
## Minimum correlation of possible factor scores     0.57
```

Graded-Response Model: Social Avoidance

```
##      Extrmt1 Extrmt2 Extrmt3 Extrmt4 Extrmt5 Extrmt6 Dscrmn
## Q64   -1.159  -0.485  -0.075   0.326   0.783   1.313  3.428
## Q120  -1.456  -0.729  -0.161   0.308   0.810   1.467  1.845
## Q121  -1.274  -0.478   0.056   0.587   1.124   2.045  1.562
```

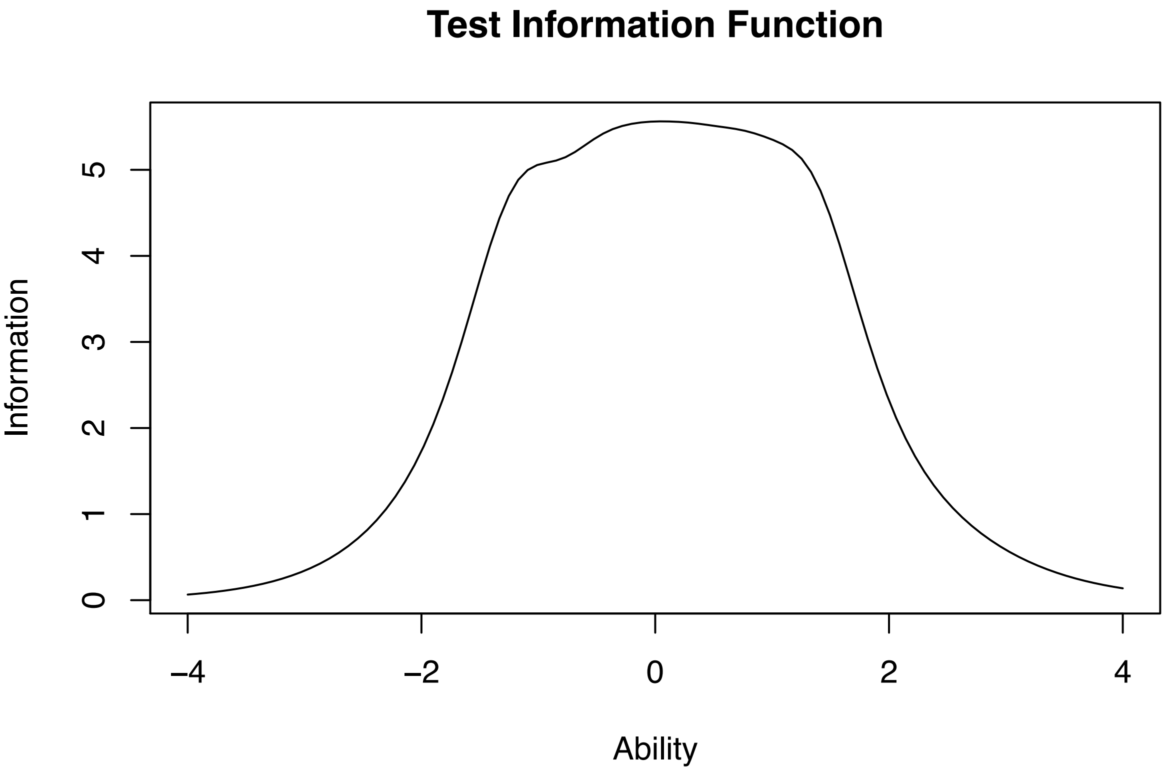

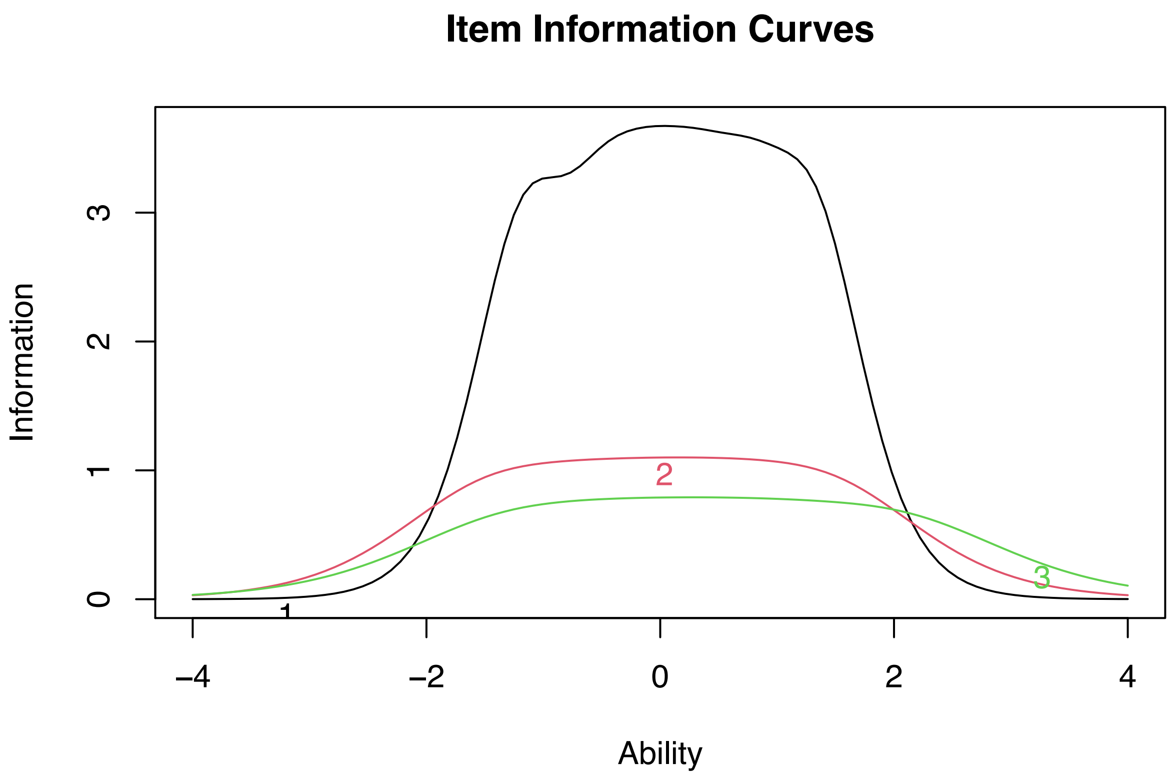

Back to top

Site 2

Reliability: Social Avoidance

```
## Cronbach's alpha is 0.789.
## Mean item-total correlation is 0.556.
## If each item were dropped:
##      raw_alpha std.alpha G6(smc) average_r S/N alpha se var.r med.r
## Q64       0.63      0.63    0.46      0.46 1.7    0.030    NA  0.46
## Q120      0.73      0.73    0.57      0.57 2.7    0.022    NA  0.57
## Q121      0.78      0.78    0.64      0.64 3.5    0.018    NA  0.64
```

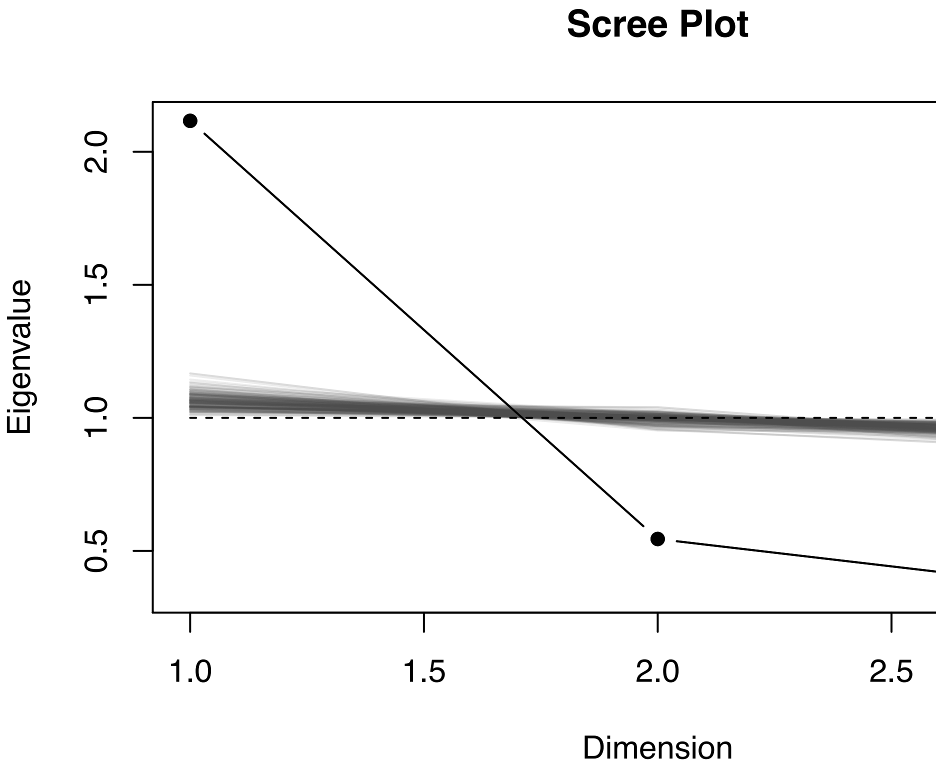

Unidimensionality: Social Avoidance

```
## [1] "Ratio of first to second eigenvalues: 3.885"

## [1] 2.1162198 0.5447536 0.3390266

## Factor Analysis using method =  minres
## Call: fa(r = grm_obj$X)
## Standardized loadings (pattern matrix) based upon correlation matrix
##       MR1   h2   u2 com
## Q64  0.89 0.78 0.22   1
## Q120 0.72 0.52 0.48   1
## Q121 0.64 0.41 0.59   1
##
##                 MR1
## SS loadings    1.72
## Proportion Var 0.57
##
## Mean item complexity =  1
## Test of the hypothesis that 1 factor is sufficient.
##
## The degrees of freedom for the null model are  3  and the objective function was  0.94 with Chi Squa
## The degrees of freedom for the model are 0  and the objective function was  0
##
## The root mean square of the residuals (RMSR) is  0
## The df corrected root mean square of the residuals is  NA
##
## The harmonic number of observations is  574 with the empirical chi square  0  with prob <  NA
## The total number of observations was  596  with Likelihood Chi Square =  0  with prob <  NA
##
## Tucker Lewis Index of factoring reliability =  -Inf
## Fit based upon off diagonal values = 1
## Measures of factor score adequacy
```

```
##                                                    MR1
## Correlation of (regression) scores with factors   0.92
## Multiple R square of scores with factors          0.84
## Minimum correlation of possible factor scores     0.69
```

Graded-Response Model: Social Avoidance

```
##      Extrmt1 Extrmt2 Extrmt3 Extrmt4 Extrmt5 Extrmt6 Dscrmn
## Q64   -0.930  -0.284   0.199   0.574   1.013   1.524  3.766
## Q120  -1.072  -0.457   0.037   0.484   0.926   1.438  2.104
## Q121  -1.110  -0.308   0.156   0.821   1.362   2.044  1.567
```

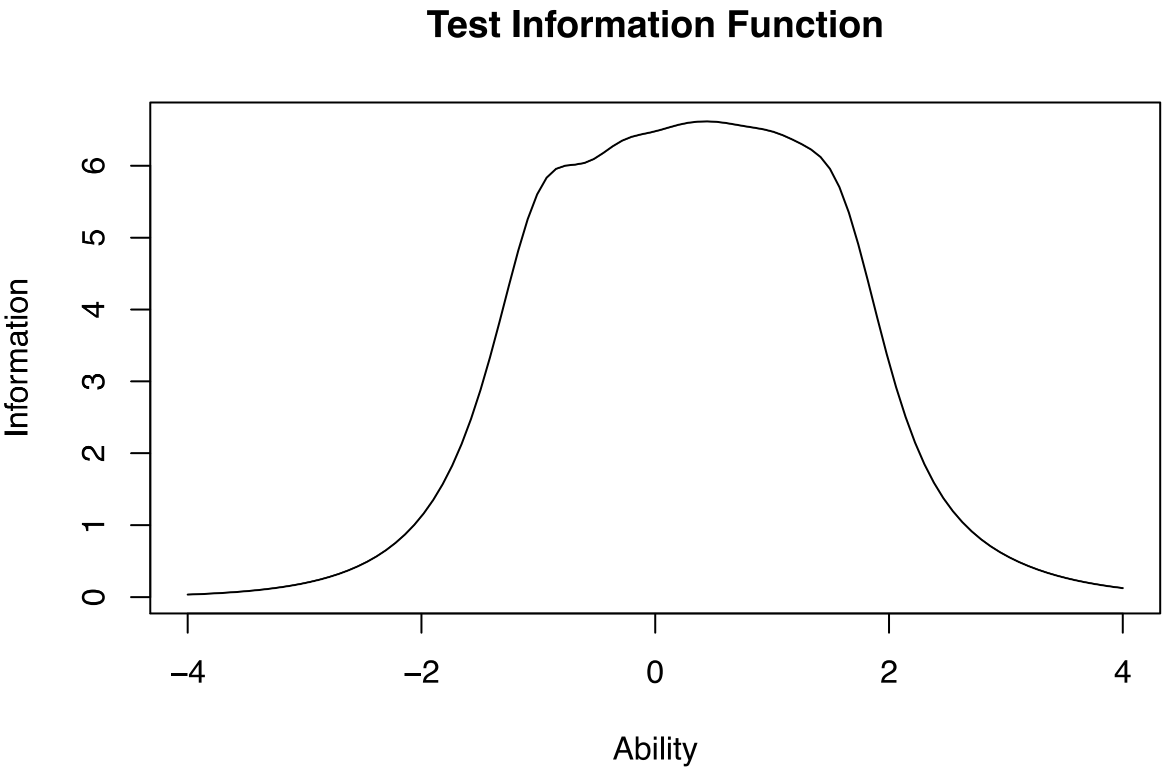

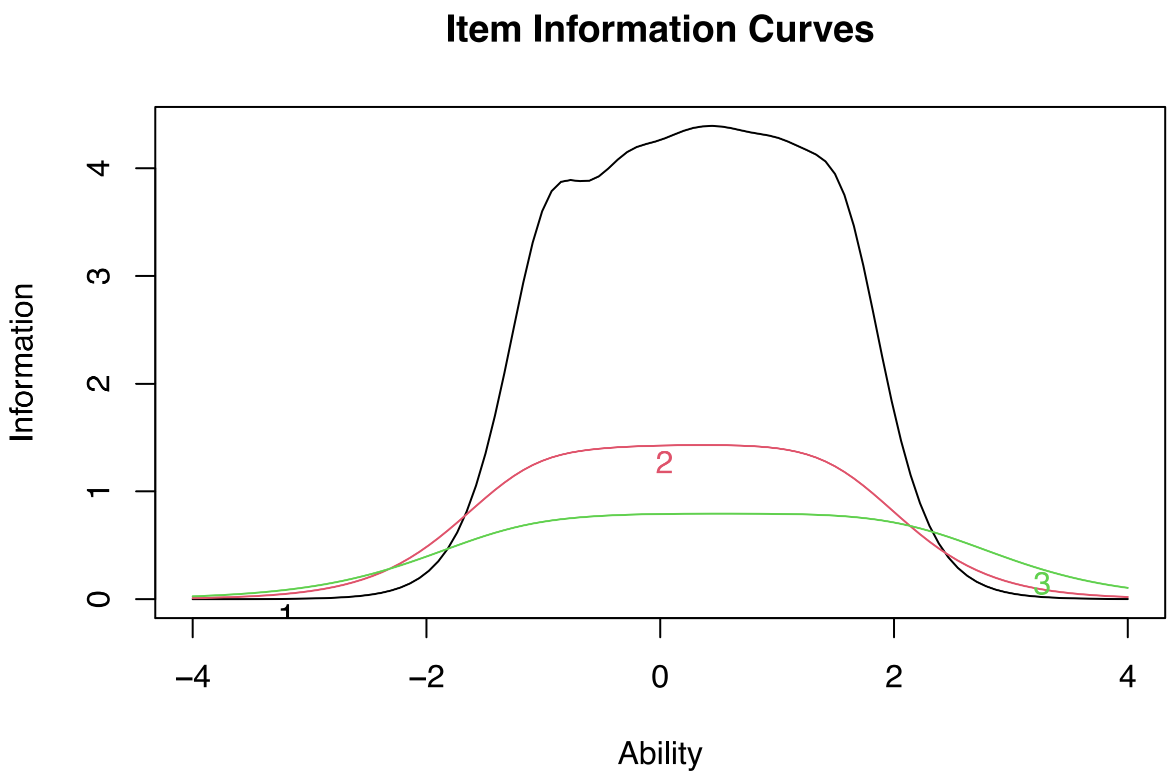

Back to top

Gender-based DIF: Social Avoidance

```
## No Gender-based DIF detected
```

Age-based DIF: Social Avoidance

```
## No age-based DIF detected
```

Back to top

Worry

Site 1

Reliability: Worry

```
## Cronbach's alpha is 0.792.
## Mean item-total correlation is 0.56.
## If each item were dropped:
##      raw_alpha std.alpha G6(smc) average_r S/N alpha se var.r med.r
## Q39       0.73      0.73    0.58      0.58 2.7    0.022    NA  0.58
## Q116      0.75      0.75    0.60      0.60 3.0    0.020    NA  0.60
## Q117      0.67      0.67    0.50      0.50 2.0    0.027    NA  0.50
```

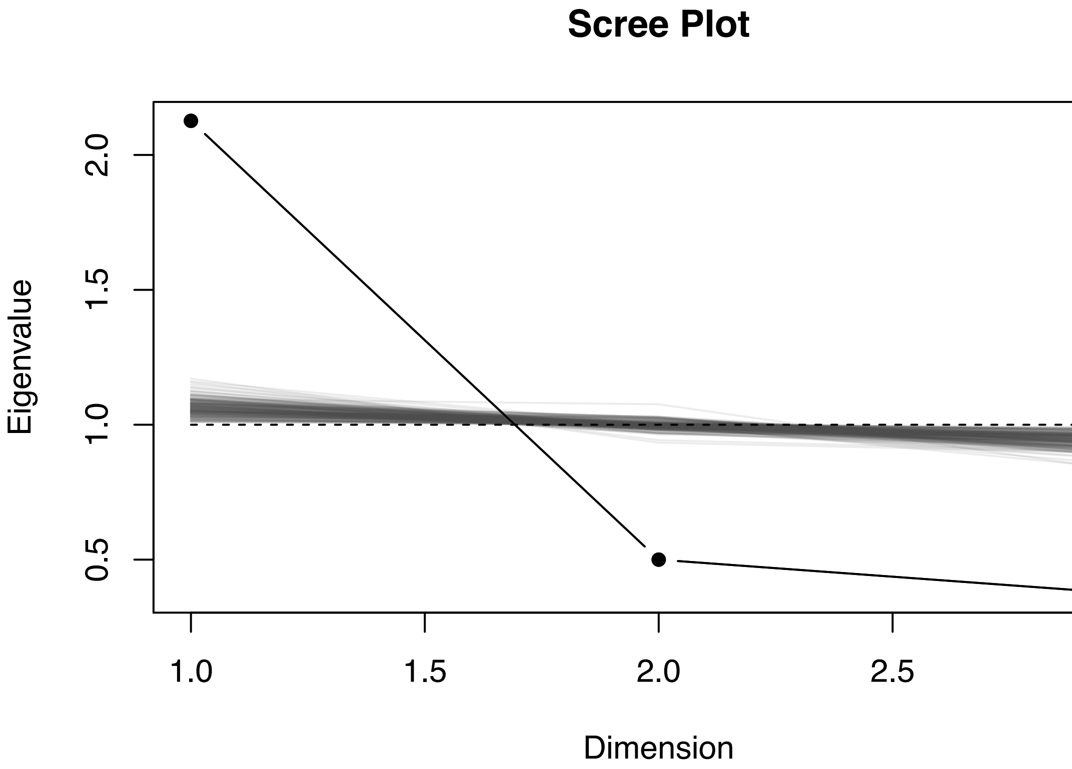

Unidimensionality: Worry

```
## [1] "Ratio of first to second eigenvalues: 4.253"

## [1] 2.1263888 0.4999869 0.3736244

## Factor Analysis using method =  minres
## Call: fa(r = grm_obj$X)
## Standardized loadings (pattern matrix) based upon correlation matrix
##       MR1   h2   u2 com
## Q39  0.72 0.52 0.48   1
## Q116 0.69 0.48 0.52   1
## Q117 0.84 0.70 0.30   1
##
##                  MR1
## SS loadings    1.71
## Proportion Var 0.57
##
## Mean item complexity =  1
## Test of the hypothesis that 1 factor is sufficient.
##
## The degrees of freedom for the null model are  3  and the objective function was  0.92 with Chi Squa
## The degrees of freedom for the model are 0  and the objective function was  0
##
## The root mean square of the residuals (RMSR) is  0
## The df corrected root mean square of the residuals is  NA
##
## The harmonic number of observations is  591 with the empirical chi square  0  with prob <  NA
## The total number of observations was  617  with Likelihood Chi Square =  0  with prob <  NA
##
## Tucker Lewis Index of factoring reliability =  -Inf
## Fit based upon off diagonal values = 1
## Measures of factor score adequacy
```

```
##                                                     MR1
## Correlation of (regression) scores with factors   0.90
## Multiple R square of scores with factors          0.81
## Minimum correlation of possible factor scores     0.63
```

Graded-Response Model: Worry

```
##      Extrmt1 Extrmt2 Extrmt3 Extrmt4 Extrmt5 Extrmt6 Dscrmn
## Q39   -2.109  -1.523  -1.007  -0.593   0.071   0.661  1.958
## Q116  -1.872  -1.550  -1.176  -0.691  -0.202   0.280  1.992
## Q117  -1.844  -1.286  -1.005  -0.603  -0.104   0.356  3.439
```

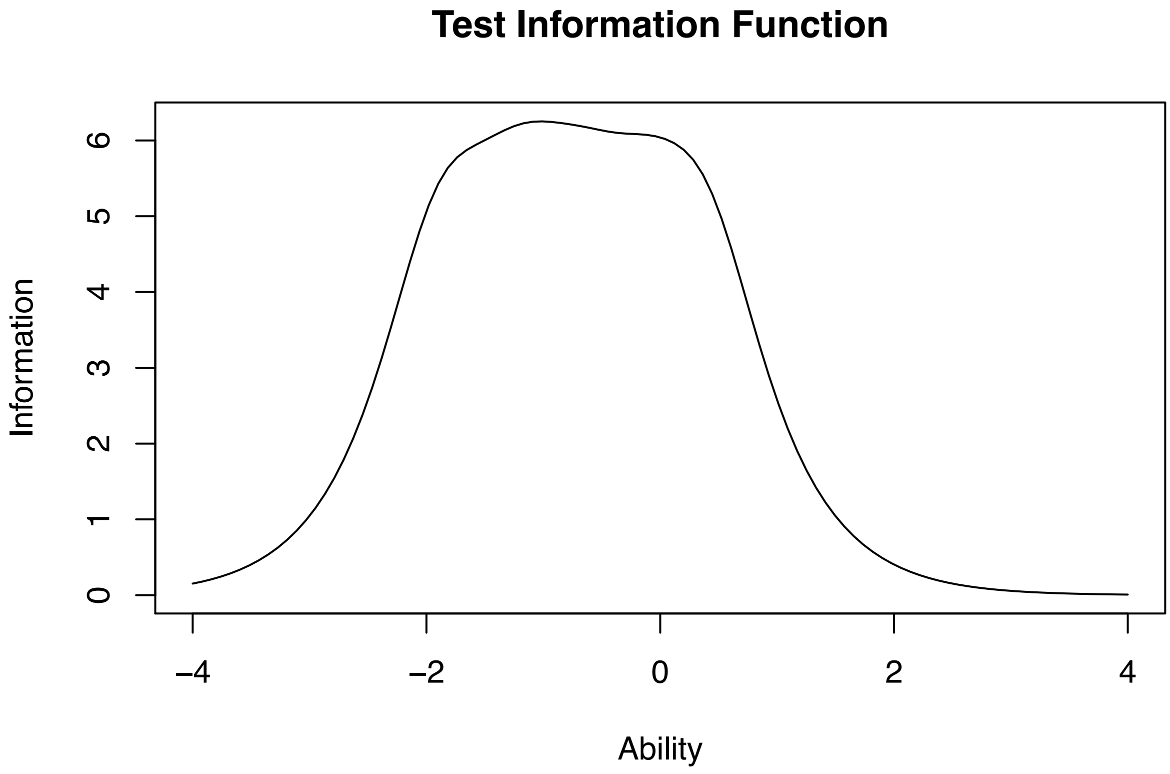

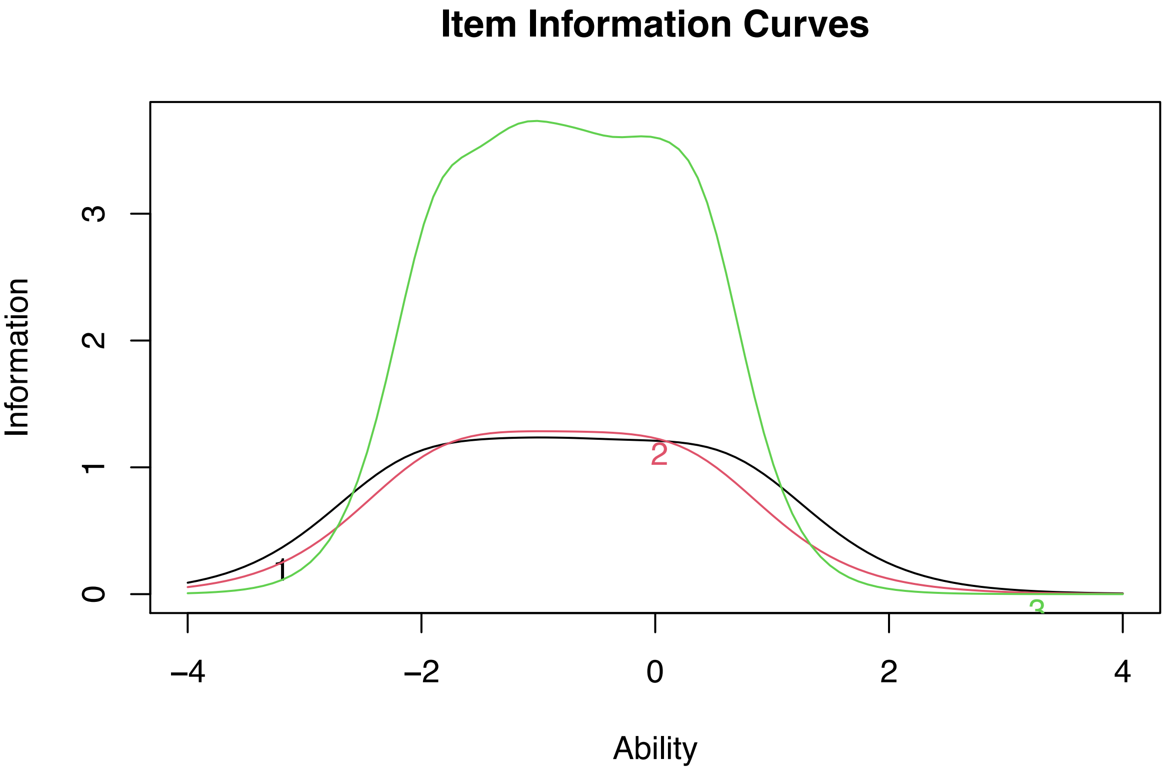

Back to top

Site 2

Reliability: Worry

```
## Cronbach's alpha is 0.807.
## Mean item-total correlation is 0.583.
## If each item were dropped:
##      raw_alpha std.alpha G6(smc) average_r S/N alpha se var.r med.r
## Q39       0.74      0.74    0.59      0.59 2.9    0.021    NA  0.59
## Q116      0.77      0.77    0.63      0.63 3.4    0.018    NA  0.63
## Q117      0.69      0.69    0.53      0.53 2.2    0.025    NA  0.53
```

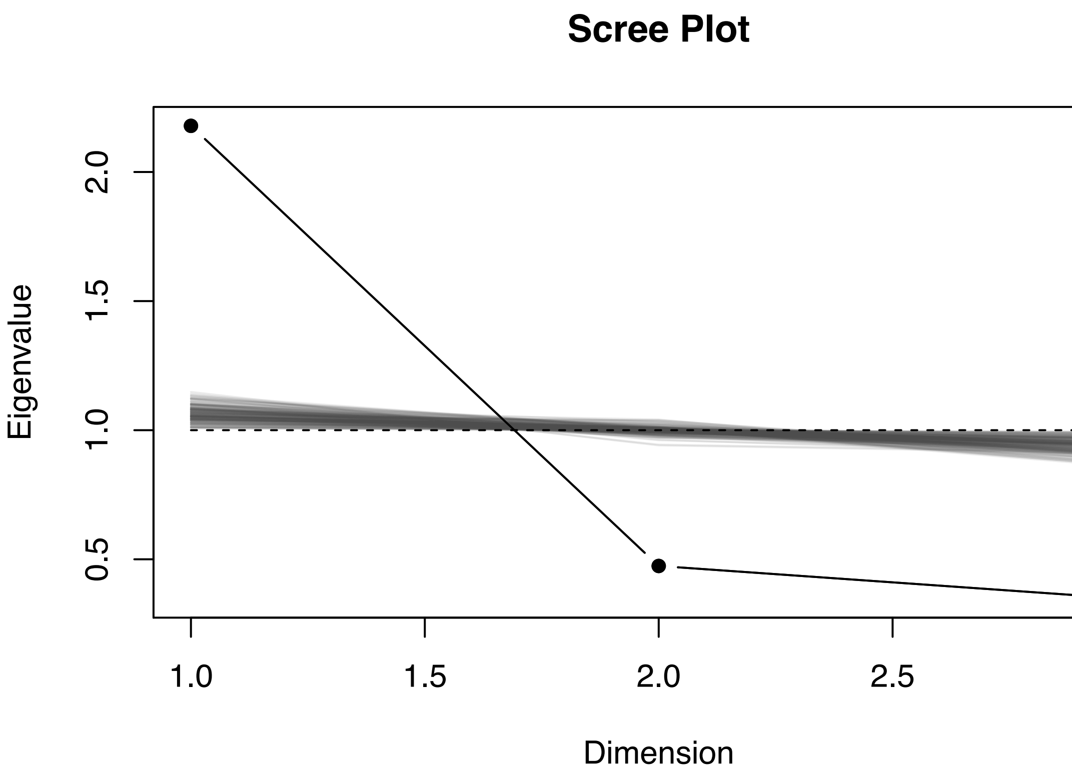

Unidimensionality: Worry

```
## [1] "Ratio of first to second eigenvalues: 4.596"
```

```
## [1] 2.178763 0.474076 0.347161
```

```
## Factor Analysis using method =  minres
## Call: fa(r = grm_obj$X)
## Standardized loadings (pattern matrix) based upon correlation matrix
##       MR1   h2   u2 com
## Q39  0.76 0.57 0.43   1
## Q116 0.70 0.49 0.51   1
## Q117 0.85 0.72 0.28   1
##
##                 MR1
## SS loadings    1.78
## Proportion Var 0.59
##
## Mean item complexity =  1
## Test of the hypothesis that 1 factor is sufficient.
##
## The degrees of freedom for the null model are  3  and the objective function was  1.03 with Chi Squa
## The degrees of freedom for the model are 0  and the objective function was  0
##
## The root mean square of the residuals (RMSR) is  0
## The df corrected root mean square of the residuals is  NA
##
## The harmonic number of observations is  572 with the empirical chi square  0  with prob <  NA
## The total number of observations was  596  with Likelihood Chi Square =  0  with prob <  NA
##
## Tucker Lewis Index of factoring reliability =  NaN
## Fit based upon off diagonal values = 1
## Measures of factor score adequacy
```

```
##                                                MR1
## Correlation of (regression) scores with factors   0.91
## Multiple R square of scores with factors          0.83
## Minimum correlation of possible factor scores     0.66
```

Graded-Response Model: Worry

```
##      Extrmt1 Extrmt2 Extrmt3 Extrmt4 Extrmt5 Extrmt6 Dscrmn
## Q39   -1.844  -1.087  -0.577  -0.154   0.365   0.849  2.352
## Q116  -1.844  -1.403  -0.961  -0.565  -0.112   0.497  2.106
## Q117  -1.682  -1.157  -0.780  -0.394   0.167   0.718  3.147
```

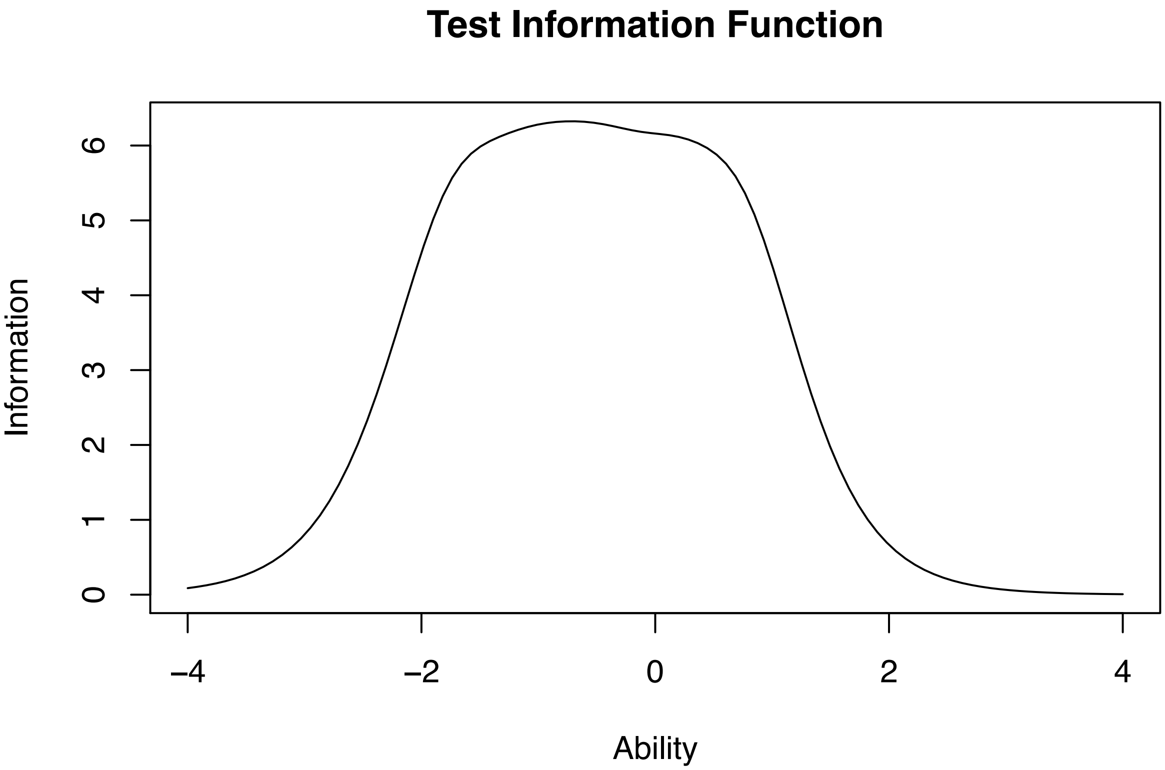

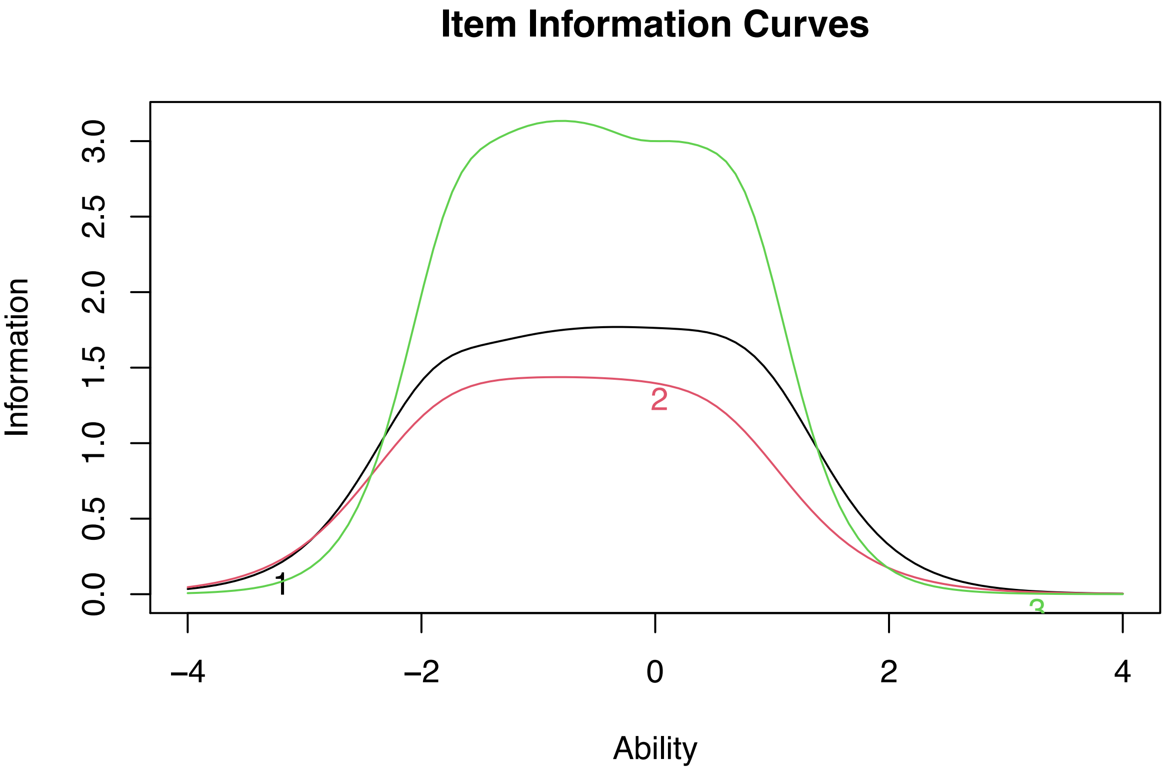

Back to top

Gender-based DIF: Worry

```
## No Gender-based DIF detected
```

Age-based DIF: Worry

```
## No age-based DIF detected
```

Back to top

Cognitive Problems

Site 1

Reliability: Cognitive Problems

```
## Cronbach's alpha is 0.893.
## Mean item-total correlation is 0.581.
## If each item were dropped:
##      raw_alpha std.alpha G6(smc) average_r S/N alpha se  var.r med.r
## Q142      0.87      0.87    0.87      0.58 7.0   0.0081 0.0133  0.58
## Q143      0.87      0.87    0.86      0.58 6.9   0.0081 0.0074  0.58
## Q144      0.86      0.86    0.86      0.55 6.1   0.0091 0.0120  0.51
## Q145      0.87      0.87    0.86      0.58 6.9   0.0082 0.0079  0.58
## Q146      0.89      0.89    0.89      0.61 7.8   0.0074 0.0118  0.63
## Q147      0.88      0.88    0.87      0.58 7.0   0.0080 0.0141  0.53
```

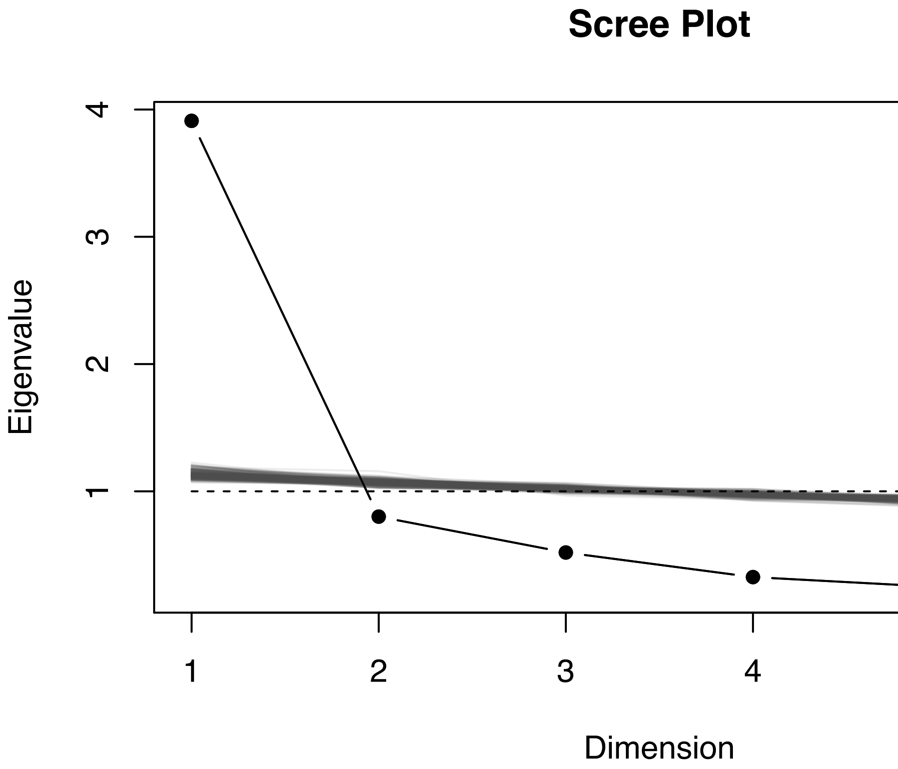

Unidimensionality: Cognitive Problems

```
## [1] "Ratio of first to second eigenvalues: 4.882"

## [1] 3.9111337 0.8010990 0.5190344 0.3259243 0.2476788 0.1951297

## Factor Analysis using method =  minres
## Call: fa(r = grm_obj$X)
## Standardized loadings (pattern matrix) based upon correlation matrix
##       MR1   h2   u2 com
## Q142 0.76 0.58 0.42   1
## Q143 0.77 0.60 0.40   1
## Q144 0.86 0.75 0.25   1
## Q145 0.77 0.60 0.40   1
## Q146 0.66 0.44 0.56   1
## Q147 0.74 0.55 0.45   1
##
##                 MR1
## SS loadings    3.51
## Proportion Var 0.58
##
## Mean item complexity =  1
## Test of the hypothesis that 1 factor is sufficient.
##
## The degrees of freedom for the null model are  15  and the objective function was  3.66 with Chi Squa
## The degrees of freedom for the model are 9  and the objective function was  0.56
##
## The root mean square of the residuals (RMSR) is  0.08
## The df corrected root mean square of the residuals is  0.11
##
## The harmonic number of observations is  590 with the empirical chi square  125.13  with prob <  1.2e-
## The total number of observations was  617  with Likelihood Chi Square =  341.82  with prob <  3.4e-68
##
```

```
## Tucker Lewis Index of factoring reliability =  0.751
## RMSEA index =  0.245  and the 90 % confidence intervals are  0.223 0.268
## BIC =  283.99
## Fit based upon off diagonal values = 0.98
## Measures of factor score adequacy
##                                                    MR1
## Correlation of (regression) scores with factors   0.95
## Multiple R square of scores with factors          0.90
## Minimum correlation of possible factor scores     0.81
```

Graded-Response Model: Cognitive Problems

```
##      Extrmt1 Extrmt2 Extrmt3 Extrmt4 Extrmt5 Extrmt6 Dscrmn
## Q142  -1.448  -0.902  -0.382   0.054   0.566   1.003  2.293
## Q143  -1.257  -0.678  -0.309   0.096   0.608   1.173  2.414
## Q144  -0.941  -0.460  -0.023   0.410   0.823   1.310  3.496
## Q145  -0.808  -0.267   0.077   0.494   1.009   1.514  2.397
## Q146  -1.341  -0.702  -0.190   0.479   1.069   1.977  1.563
## Q147  -1.378  -0.737  -0.295   0.149   0.761   1.354  2.060
```

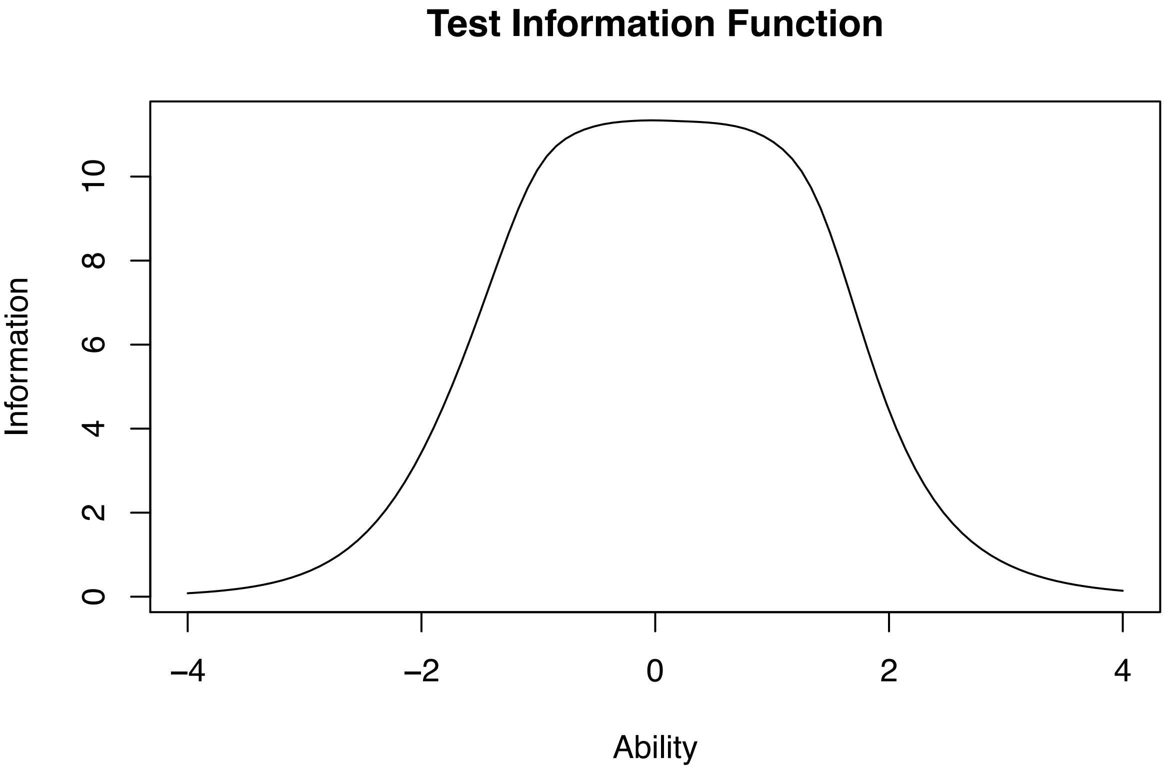

Item Information Curves

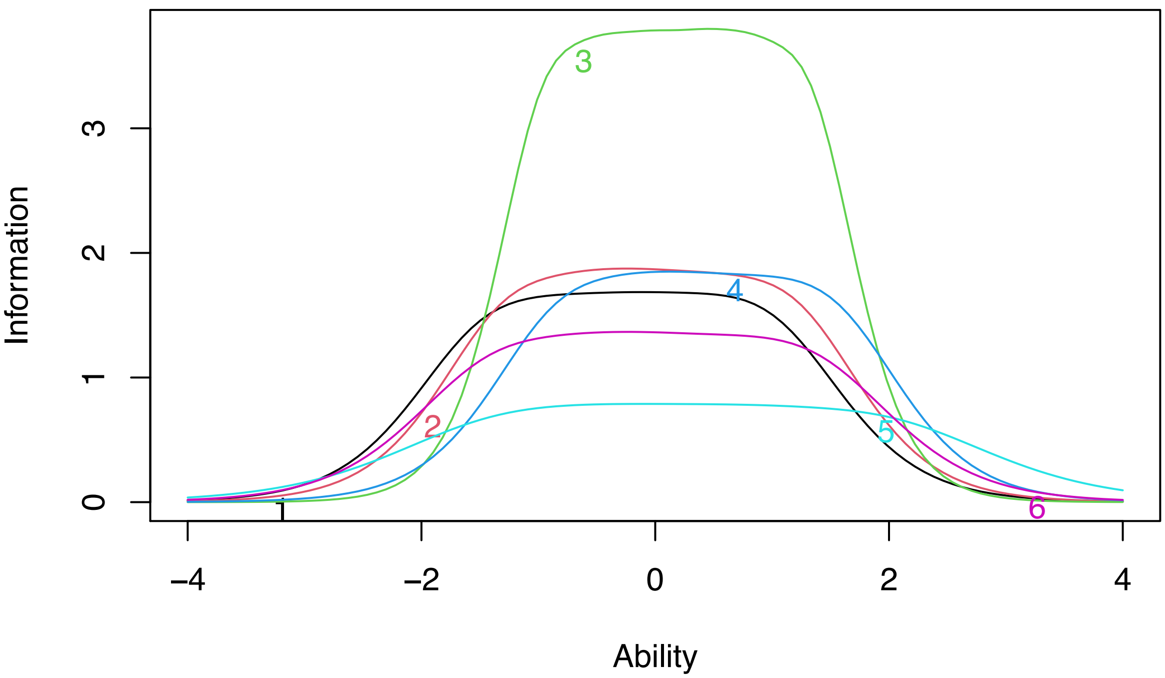

Back to top

Site 2

Reliability: Cognitive Problems

```
## Cronbach's alpha is 0.9.
## Mean item-total correlation is 0.6.
## If each item were dropped:
##      raw_alpha std.alpha G6(smc) average_r S/N alpha se  var.r med.r
## Q142      0.88      0.88    0.88      0.60 7.6   0.0076 0.0073  0.60
## Q143      0.88      0.88    0.87      0.60 7.4   0.0077 0.0061  0.60
## Q144      0.87      0.87    0.87      0.57 6.8   0.0085 0.0078  0.53
## Q145      0.88      0.88    0.87      0.60 7.6   0.0075 0.0060  0.60
## Q146      0.89      0.89    0.89      0.62 8.3   0.0071 0.0087  0.65
## Q147      0.88      0.88    0.88      0.60 7.4   0.0077 0.0096  0.55
```

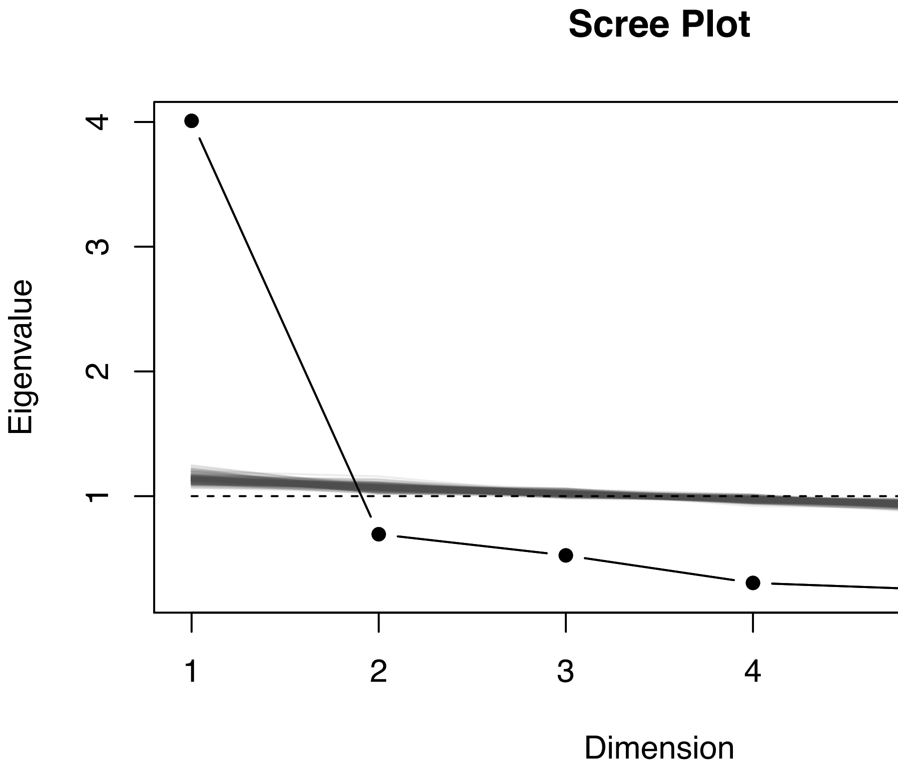

Unidimensionality: Cognitive Problems

```
## [1] "Ratio of first to second eigenvalues: 5.771"

## [1] 4.0091280 0.6946725 0.5250306 0.3042152 0.2494068 0.2175470

## Factor Analysis using method =  minres
## Call: fa(r = grm_obj$X)
## Standardized loadings (pattern matrix) based upon correlation matrix
##       MR1   h2   u2 com
## Q142 0.77 0.59 0.41   1
## Q143 0.79 0.62 0.38   1
## Q144 0.86 0.74 0.26   1
## Q145 0.76 0.59 0.41   1
## Q146 0.70 0.49 0.51   1
## Q147 0.78 0.61 0.39   1
##
##                 MR1
## SS loadings    3.62
## Proportion Var 0.60
##
## Mean item complexity =  1
## Test of the hypothesis that 1 factor is sufficient.
##
## The degrees of freedom for the null model are  15  and the objective function was  3.72 with Chi Squa
## The degrees of freedom for the model are 9  and the objective function was  0.44
##
## The root mean square of the residuals (RMSR) is  0.07
## The df corrected root mean square of the residuals is  0.09
##
## The harmonic number of observations is  574 with the empirical chi square  85.01  with prob <  1.6e-
## The total number of observations was  596  with Likelihood Chi Square =  258.79  with prob <  1.4e-5
##
```

```
## Tucker Lewis Index of factoring reliability =  0.81
## RMSEA index =  0.216  and the 90 % confidence intervals are  0.194 0.239
## BIC =  201.27
## Fit based upon off diagonal values = 0.99
## Measures of factor score adequacy
##                                                    MR1
## Correlation of (regression) scores with factors   0.95
## Multiple R square of scores with factors          0.91
## Minimum correlation of possible factor scores     0.81
```

Graded-Response Model: Cognitive Problems

```
##      Extrmt1 Extrmt2 Extrmt3 Extrmt4 Extrmt5 Extrmt6 Dscrmn
## Q142  -1.085  -0.441  -0.075   0.395   0.849   1.295  2.682
## Q143  -1.275  -0.628  -0.164   0.284   0.716   1.205  2.315
## Q144  -0.650  -0.131   0.251   0.564   0.949   1.307  3.817
## Q145  -0.900  -0.198   0.223   0.745   1.188   1.685  2.234
## Q146  -0.838  -0.120   0.241   0.881   1.482   2.113  1.903
## Q147  -0.858  -0.307   0.105   0.645   1.108   1.643  2.578
```

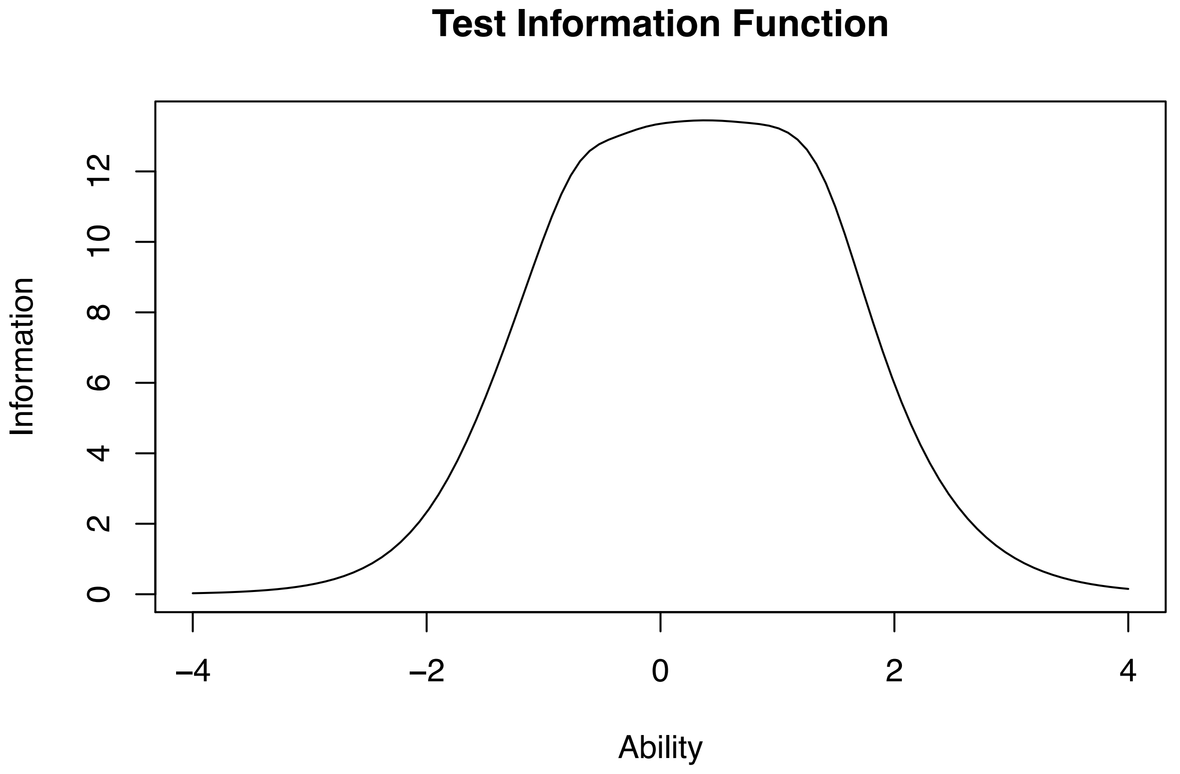

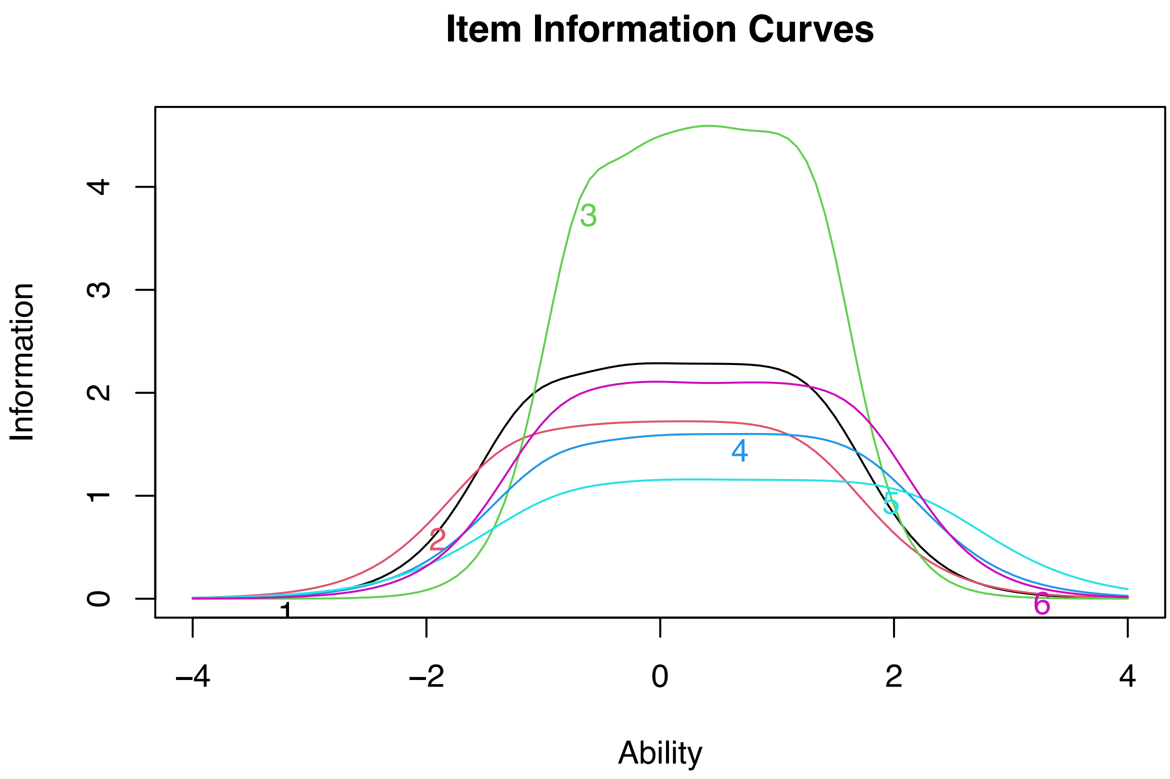

Back to top

Site DIF

```
## Call:
## lordif::lordif(resp.data = as.data.frame(merged_data), group = site)
##
##   Number of DIF groups: 2
##
##   Number of items flagged for DIF: 4 of 6
##
##   Items flagged: 2, 4, 5, 6
##
##   Number of iterations for purification: 2 of 10
##
##   Detection criterion: Chisqr
##
##   Threshold: alpha = 0.01
##
##   item ncat  chi12  chi13  chi23
## 1    1    7 0.0160 0.0478 0.5980
## 2    2    7 0.0052 0.0091 0.2078
## 3    3    7 0.6611 0.2717 0.1202
## 4    4    7 0.0115 0.0002 0.0011
## 5    5    7 0.0006 0.0028 0.9669
## 6    6    7 0.0000 0.0002 0.9054
```

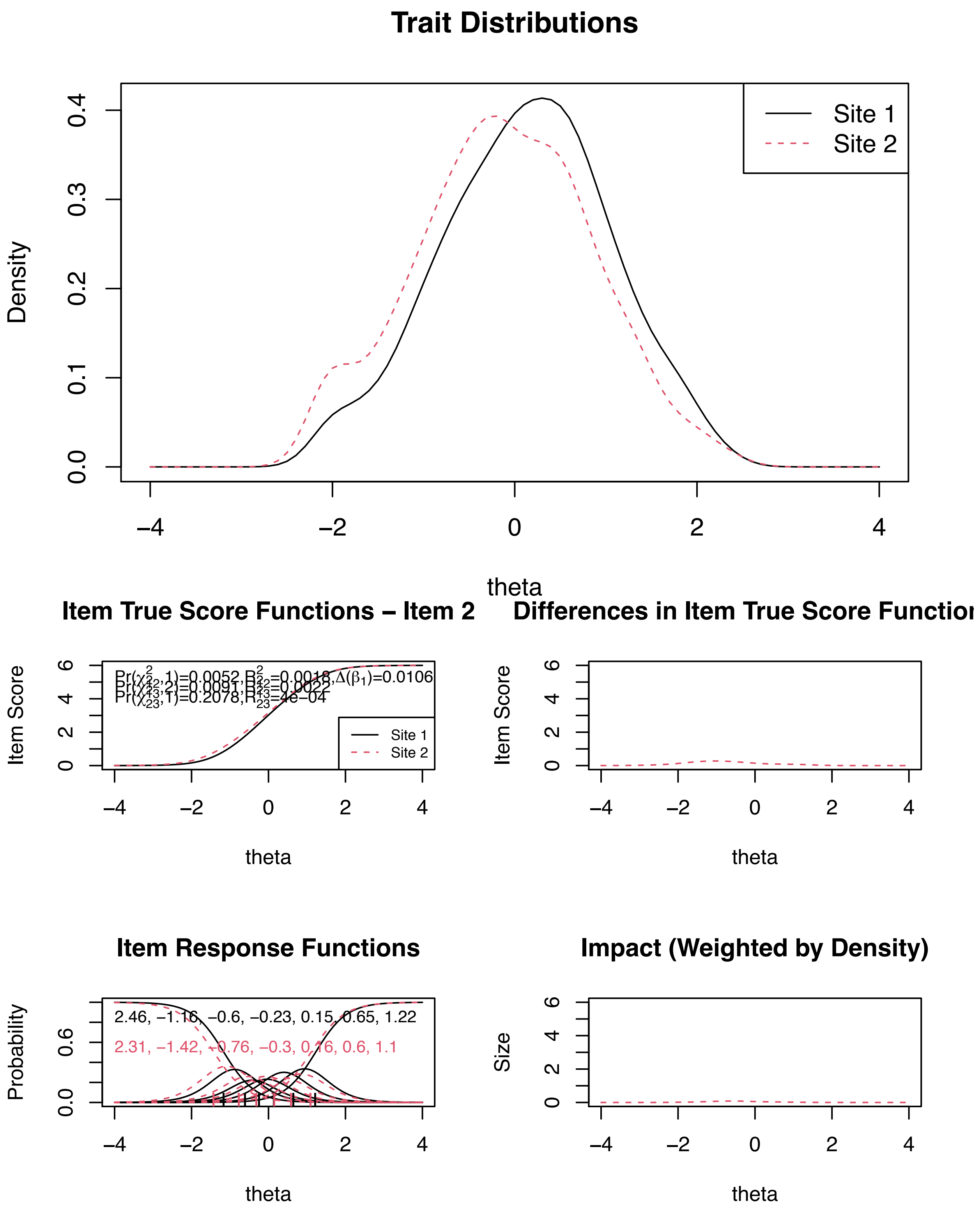
Trait Distributions
Density
theta
Site 1
Site 2
Item True Score Functions – Item 2
Item Score
Site 1
Site 2
Differences in Item True Score Functio
Item Response Functions
Probability
2.46, −1.16, −0.6, −0.23, 0.15, 0.65, 1.22
2.31, −1.42, −0.76, −0.3, 0.16, 0.6, 1.1
Impact (Weighted by Density)
Size

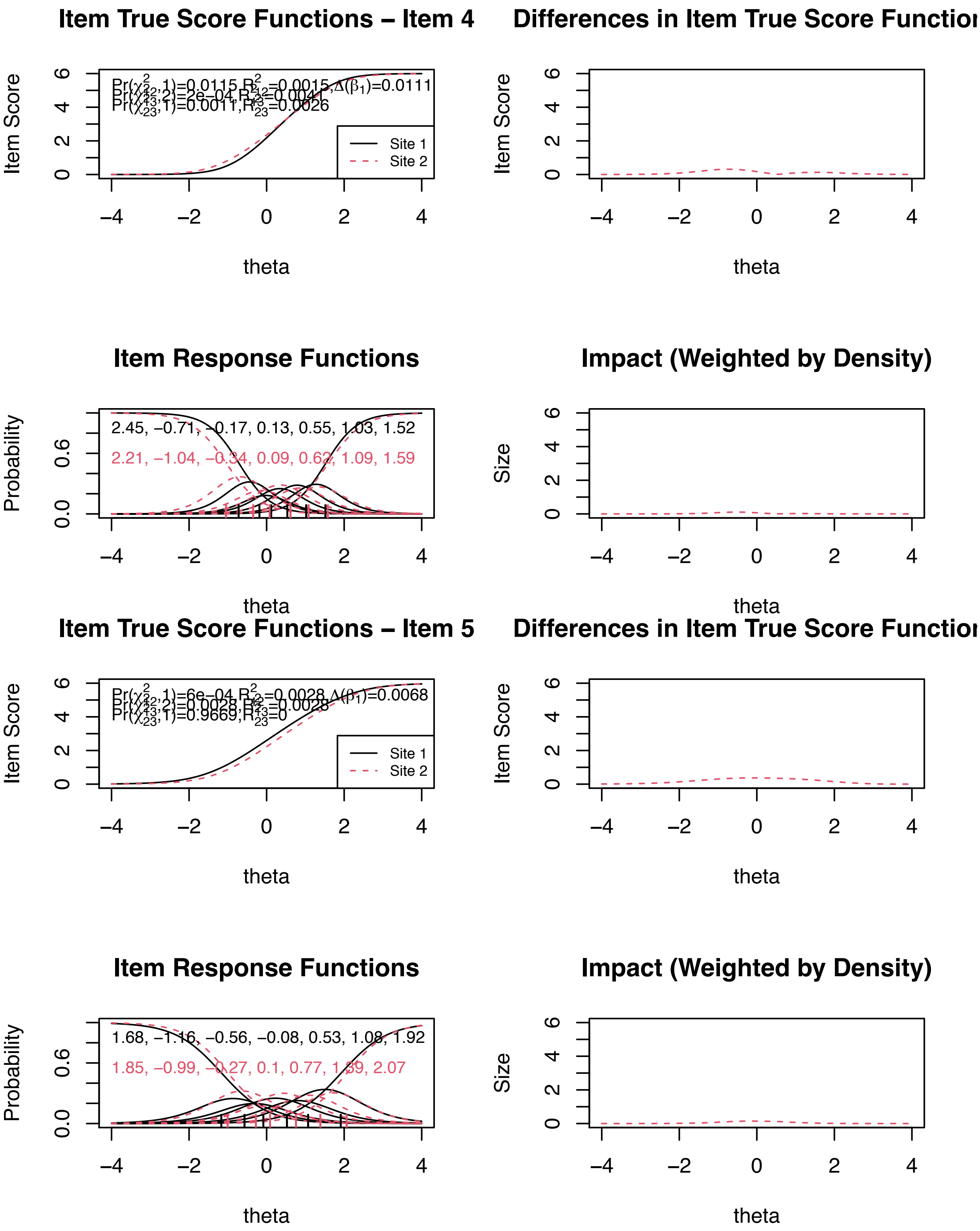
Item True Score Functions – Item 4
Differences in Item True Score Functio
Item Score
theta
Site 1
Site 2
Item Response Functions
Impact (Weighted by Density)
Probability
Size
2.45, –0.71, –0.17, 0.13, 0.55, 1.03, 1.52
2.21, –1.04, –0.34, 0.09, 0.62, 1.09, 1.59
Item True Score Functions – Item 5
1.68, –1.16, –0.56, –0.08, 0.53, 1.08, 1.92
1.85, –0.99, –0.27, 0.1, 0.77, 1.39, 2.07

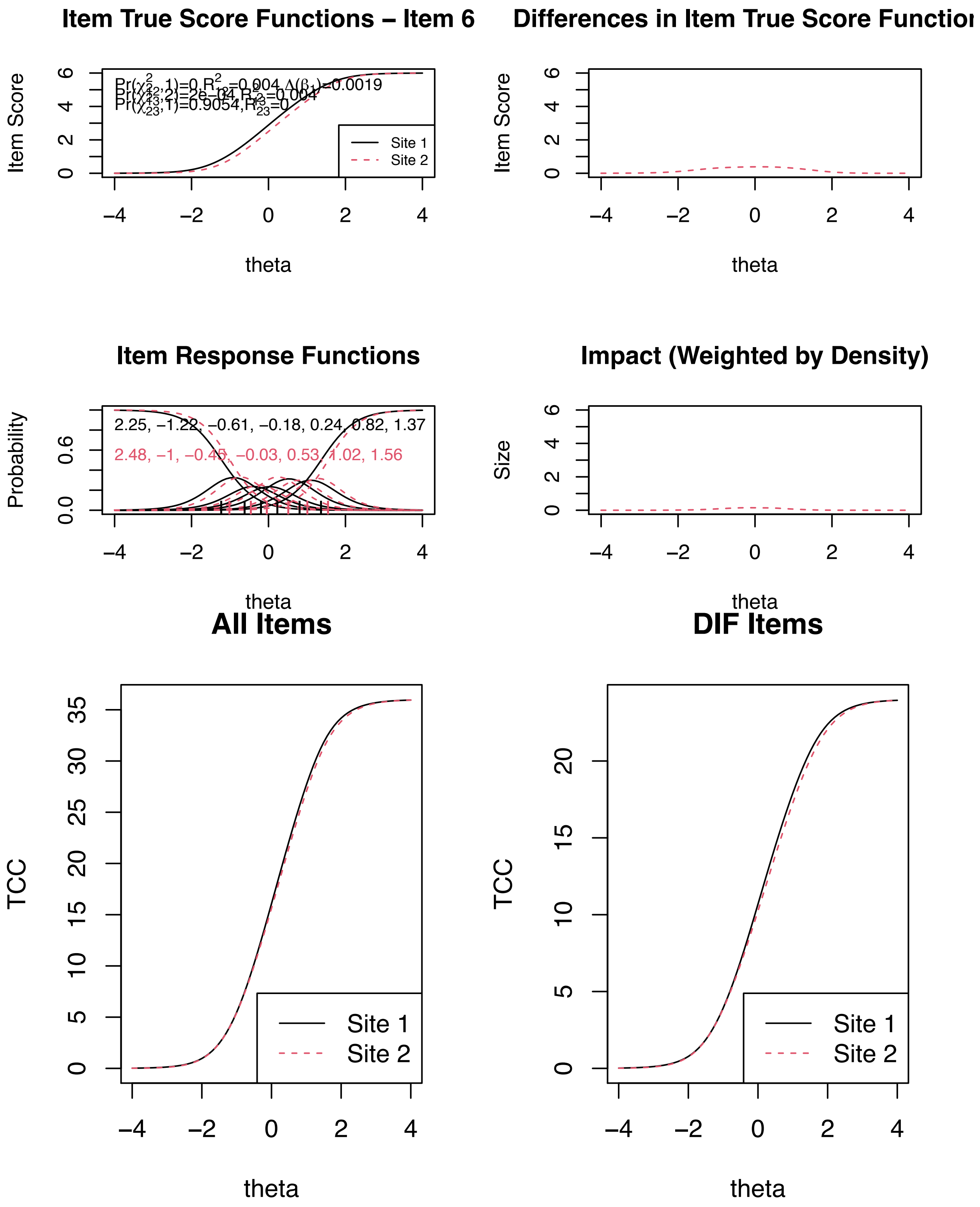
Item True Score Functions – Item 6
Differences in Item True Score Functio
Item Score
theta
Site 1
Site 2
Item Response Functions
Impact (Weighted by Density)
Probability
Size
2.25, −1.22, −0.61, −0.18, 0.24, 0.82, 1.37
2.48, −1, −0.45, −0.03, 0.53, 1.02, 1.56
All Items
DIF Items
TCC

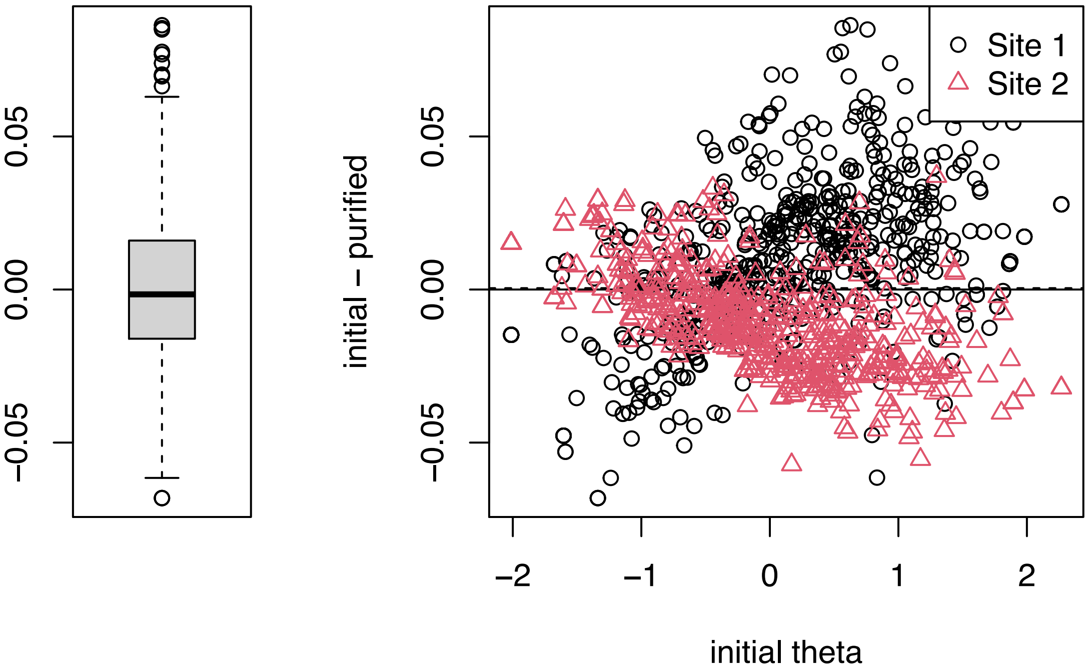

Back to top

Gender-based DIF: Cognitive Problems

```
## Call:
## lordif::lordif(resp.data = as.data.frame(sex.data), group = sex)
##
##   Number of DIF groups: 2
##
##   Number of items flagged for DIF: 2 of 6
##
##   Items flagged: 5, 6
##
##   Number of iterations for purification: 2 of 10
##
##   Detection criterion: Chisqr
##
##   Threshold: alpha = 0.01
##
##   item ncat  chi12  chi13  chi23
## 1    1    7 0.3749 0.1503 0.0831
## 2    2    7 0.2049 0.2124 0.2220
## 3    3    7 0.2336 0.3152 0.3455
## 4    4    7 0.2022 0.2066 0.2165
## 5    5    7 0.0097 0.0336 0.7521
## 6    6    7 0.0054 0.0160 0.4660
```

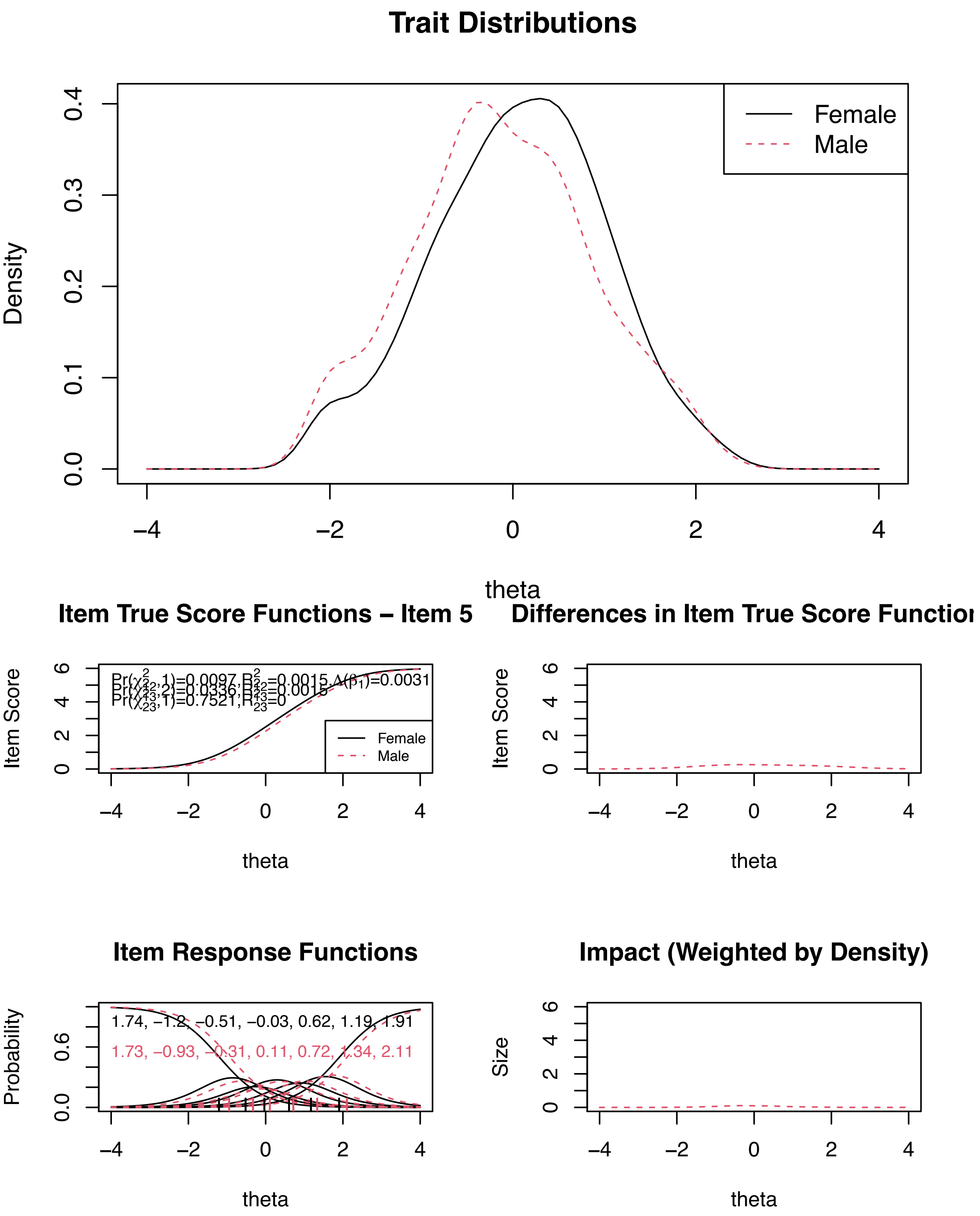
Trait Distributions
Density
theta
Female
Male
Item True Score Functions – Item 5
Differences in Item True Score Functio
Item Score
Female
Male
Item Response Functions
Probability
1.74, −1.2, −0.51, −0.03, 0.62, 1.19, 1.91
1.73, −0.93, −0.31, 0.11, 0.72, 1.34, 2.11
Impact (Weighted by Density)
Size

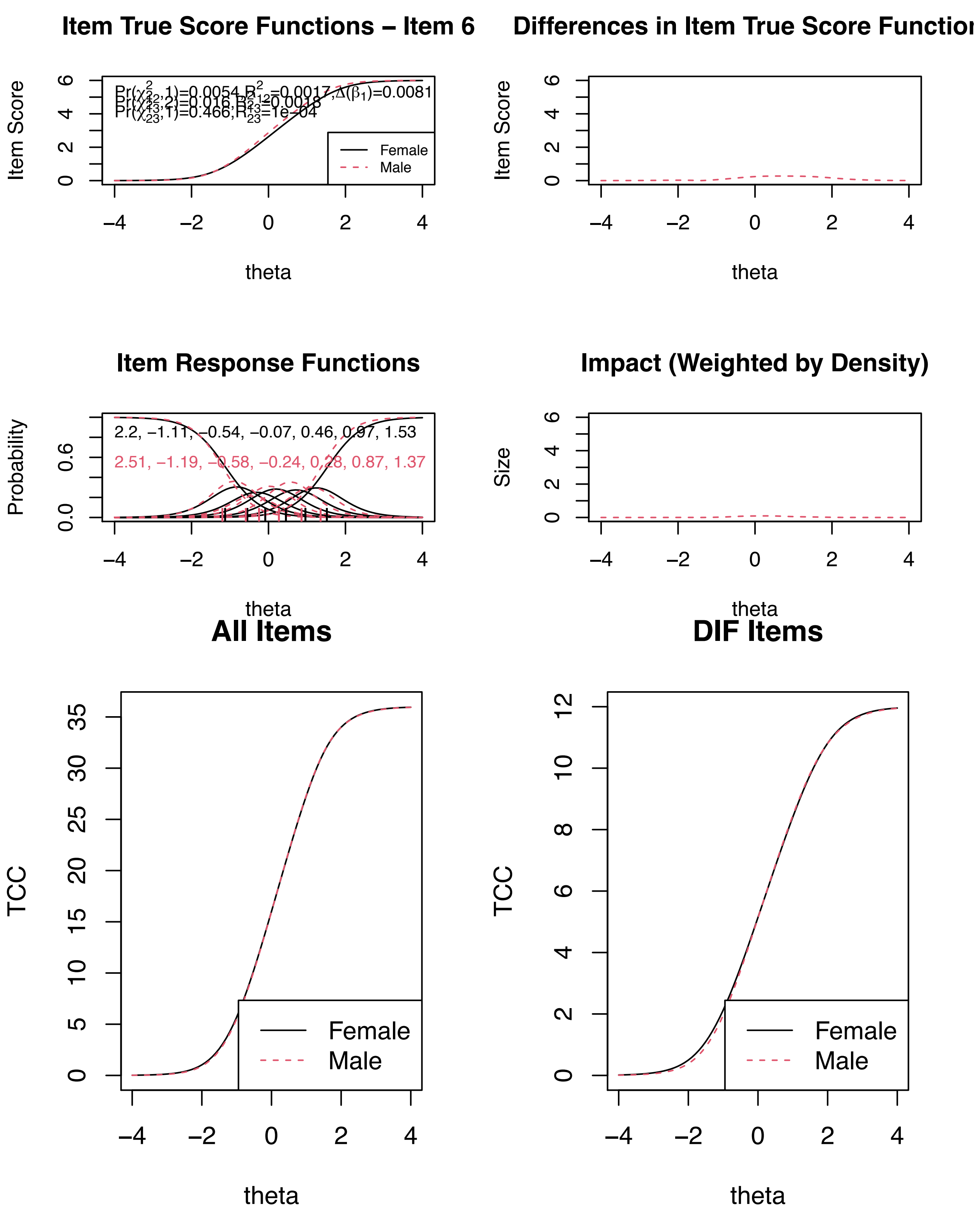
Item True Score Functions – Item 6
Item Score
theta
Female
Male
Differences in Item True Score Functio
Item Score
theta
Item Response Functions
Probability
theta
2.2, −1.11, −0.54, −0.07, 0.46, 0.97, 1.53
2.51, −1.19, −0.58, −0.24, 0.28, 0.87, 1.37
Impact (Weighted by Density)
Size
theta
All Items
TCC
theta
Female
Male
DIF Items
TCC
theta
Female
Male

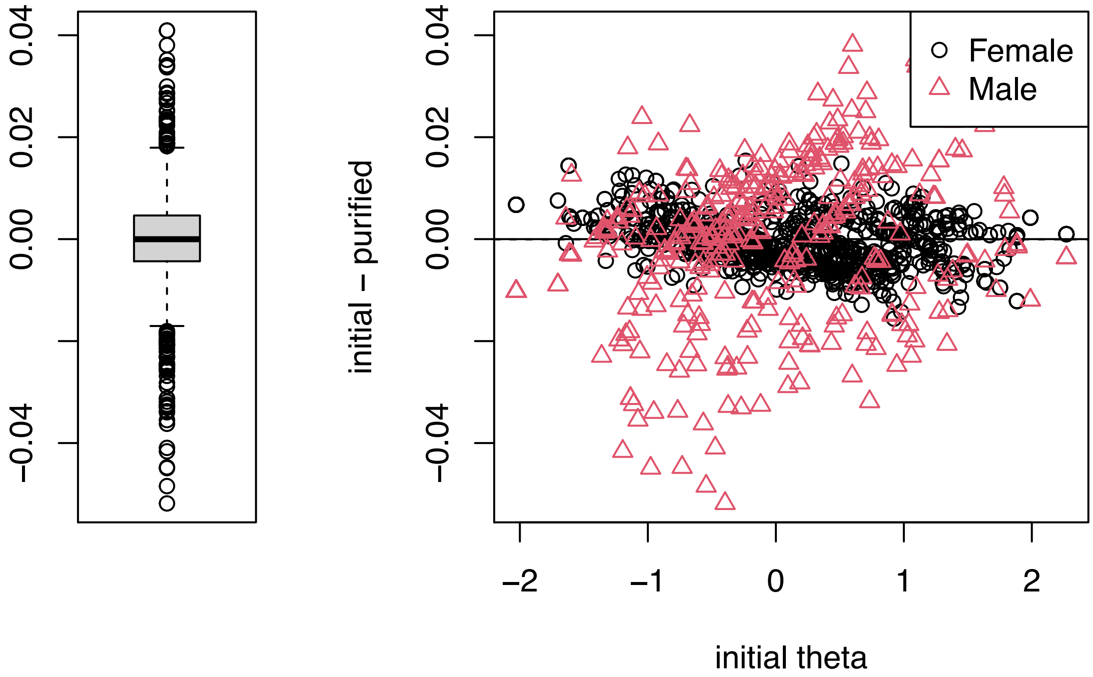

Age-based DIF: Cognitive Problems

```
## Call:
## lordif::lordif(resp.data = as.data.frame(age.data), group = age)
##
##   Number of DIF groups: 2
##
##   Number of items flagged for DIF: 4 of 6
##
##   Items flagged: 1, 2, 4, 6
##
##   Number of iterations for purification: 2 of 10
##
##   Detection criterion: Chisqr
##
##   Threshold: alpha = 0.01
##
##   item ncat  chi12  chi13  chi23
## 1    1    7 0.0000 0.0000 0.7268
## 2    2    7 0.0008 0.0015 0.1834
## 3    3    7 0.2718 0.3282 0.3123
## 4    4    7 0.0011 0.0046 0.7475
## 5    5    7 0.6536 0.1278 0.0479
## 6    6    7 0.0000 0.0000 0.1619
```

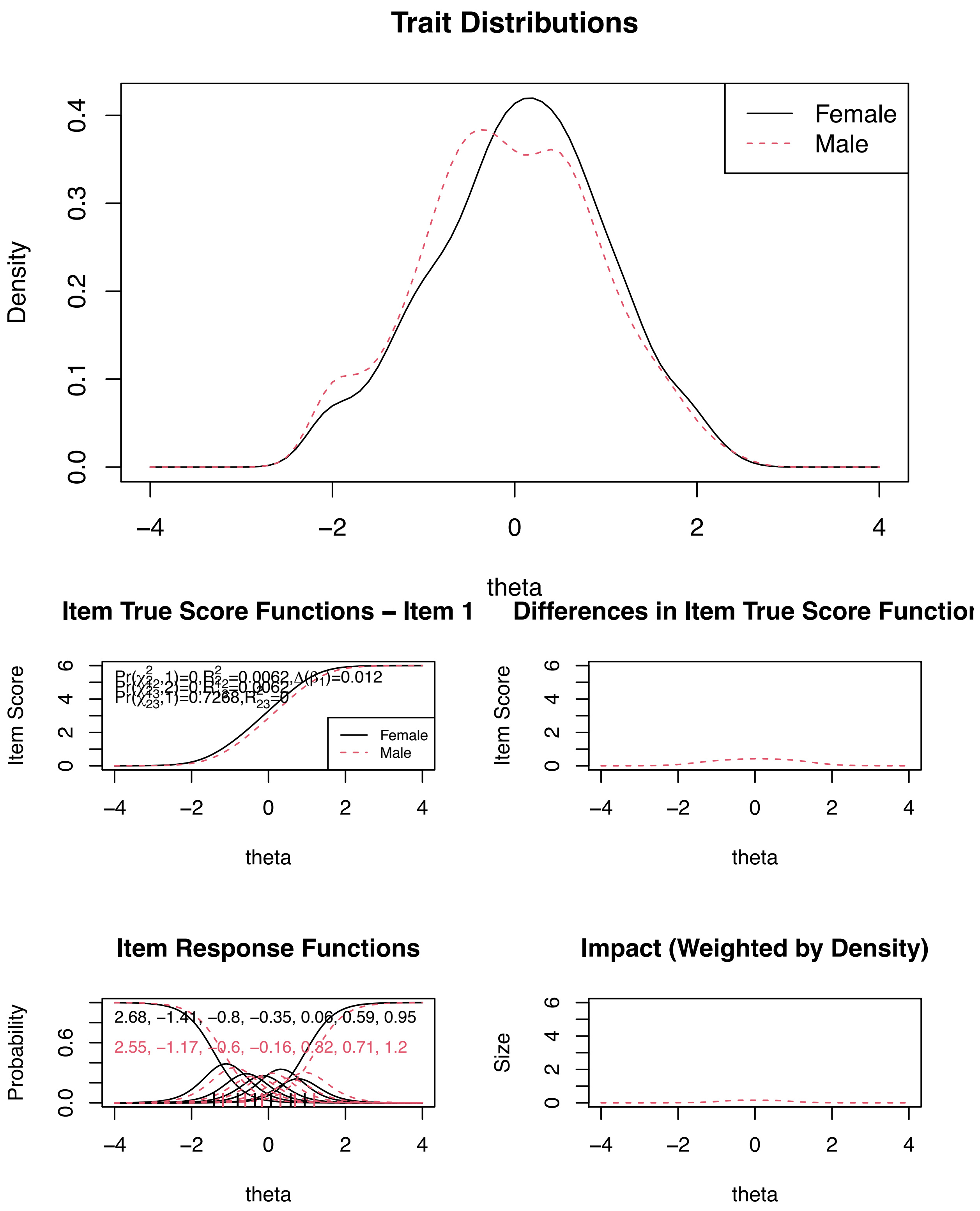
Trait Distributions
Density
theta
Female
Male
Item True Score Functions – Item 1
Differences in Item True Score Functio
Item Score
Pr(χ^2_{12},1)=0,R^2_{12}=0.0062,$\Delta(\beta_1)$=0.012
Pr(χ^2_{13},2)=0,R^2_{13}=0.0062
Pr(χ^2_{23},1)=0.7268,R^2_{23}=0
Item Response Functions
Impact (Weighted by Density)
Probability
Size
2.68, −1.41, −0.8, −0.35, 0.06, 0.59, 0.95
2.55, −1.17, −0.6, −0.16, 0.32, 0.71, 1.2

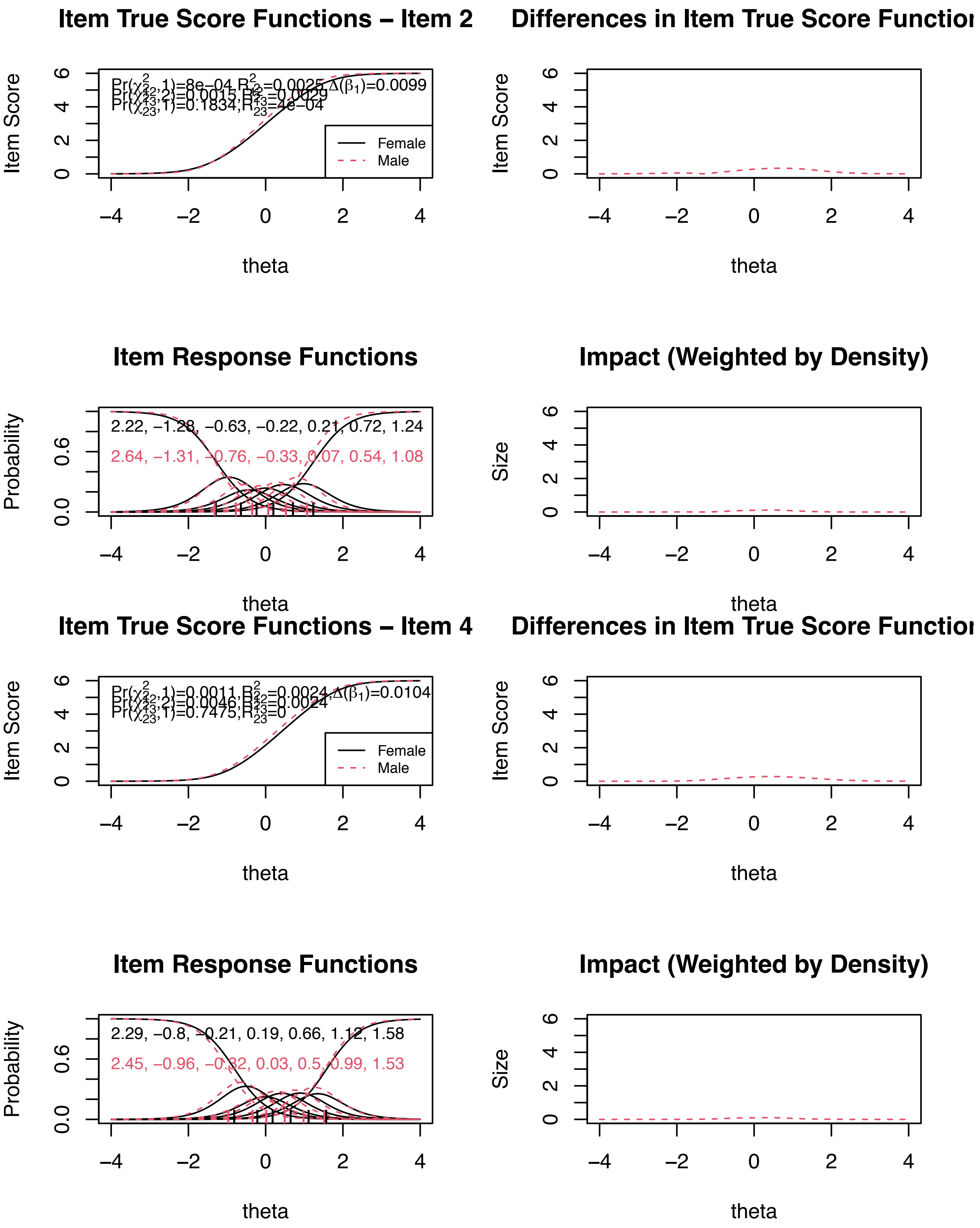

Item True Score Functions – Item 2
Differences in Item True Score Functio
Item Response Functions
2.22, −1.28, −0.63, −0.22, 0.21, 0.72, 1.24
2.64, −1.31, −0.76, −0.33, 0.07, 0.54, 1.08
Impact (Weighted by Density)
Item True Score Functions – Item 4
Differences in Item True Score Functio
Item Response Functions
2.29, −0.8, −0.21, 0.19, 0.66, 1.12, 1.58
2.45, −0.96, −0.32, 0.03, 0.5, 0.99, 1.53
Impact (Weighted by Density)
Female
Male
Item Score
Probability
Size
theta

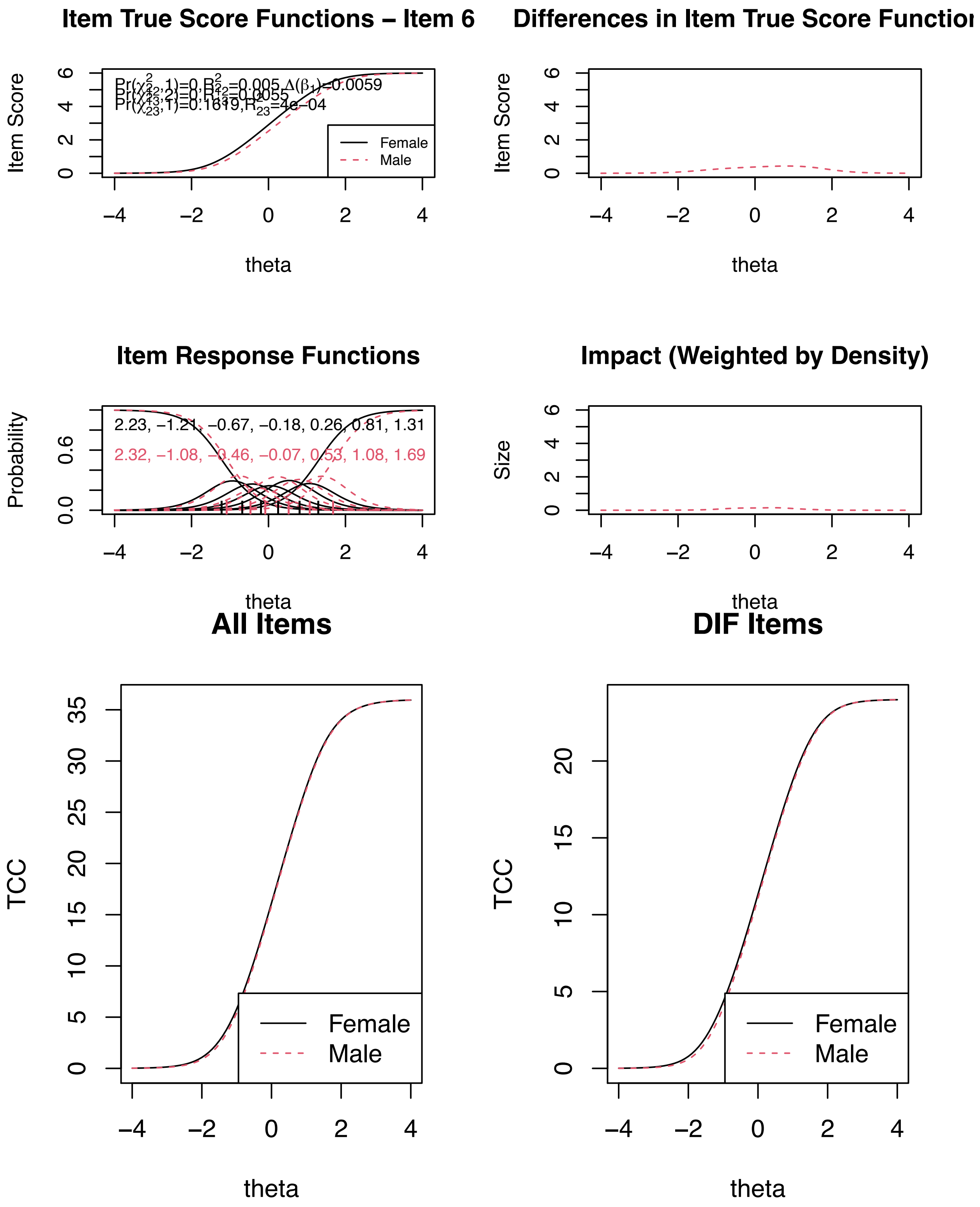
Item True Score Functions – Item 6
Item Score
theta
Female
Male
Differences in Item True Score Functio
Item Score
theta
Item Response Functions
Probability
theta
2.23, −1.21, −0.67, −0.18, 0.26, 0.81, 1.31
2.32, −1.08, −0.46, −0.07, 0.53, 1.08, 1.69
Impact (Weighted by Density)
Size
theta
All Items
TCC
theta
Female
Male
DIF Items
TCC
theta
Female
Male

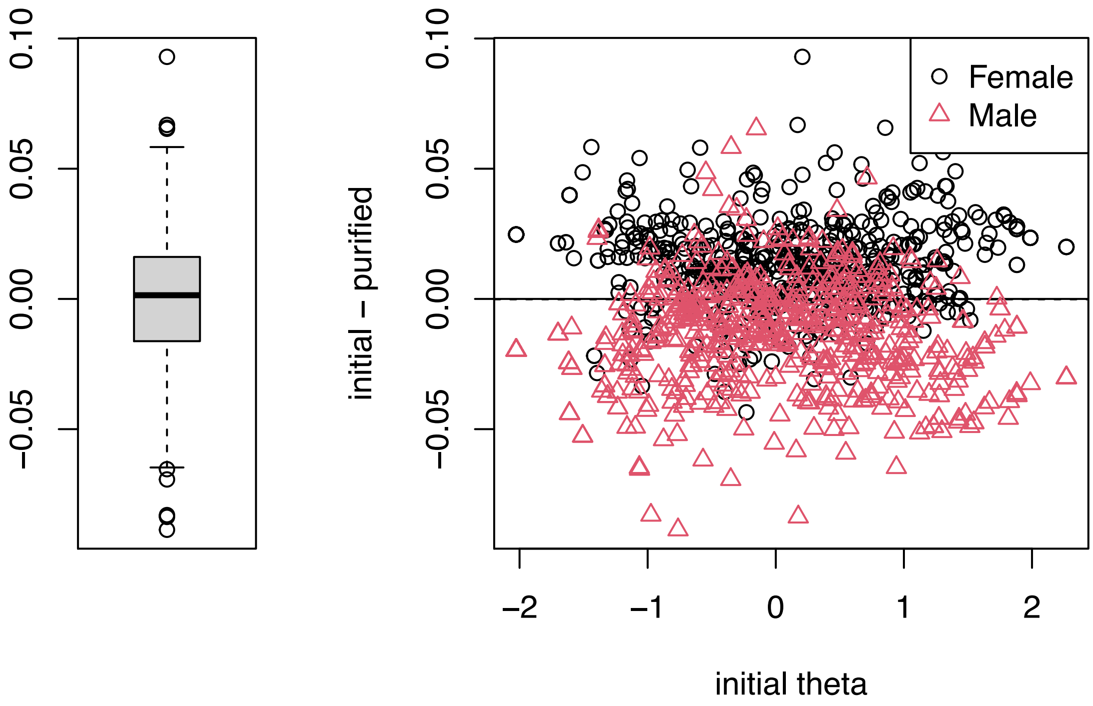

Back to top

General Functioning

Site 1

Reliability: General Functioning

```
## Cronbach's alpha is 0.562.
## Mean item-total correlation is 0.31.
## If each item were dropped:
##      raw_alpha std.alpha G6(smc) average_r  S/N alpha se var.r med.r
## Q27       0.44      0.45    0.29      0.29 0.82    0.044    NA  0.29
## Q140      0.37      0.38    0.24      0.24 0.61    0.049    NA  0.24
## Q141      0.58      0.58    0.41      0.41 1.36    0.034    NA  0.41
```

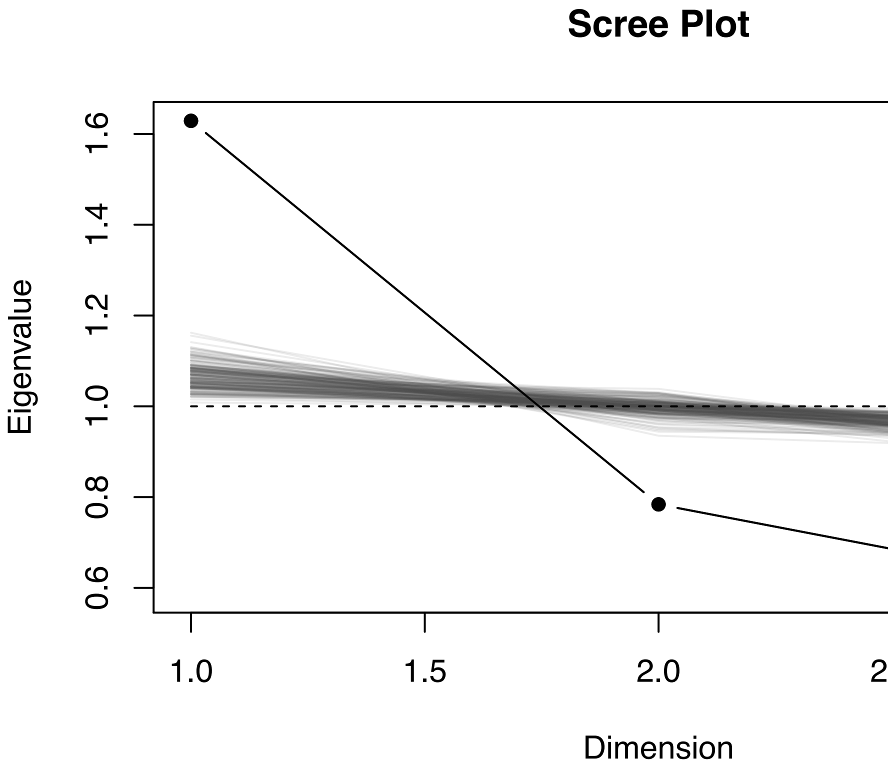

Unidimensionality: General Functioning

```
## [1] "Ratio of first to second eigenvalues: 2.078"

## [1] 1.6288837 0.7840136 0.5871027

## Factor Analysis using method =  minres
## Call: fa(r = grm_obj$X)
## Standardized loadings (pattern matrix) based upon correlation matrix
##       MR1   h2   u2 com
## Q27  0.57 0.33 0.67   1
## Q140 0.71 0.51 0.49   1
## Q141 0.41 0.17 0.83   1
##
##                 MR1
## SS loadings    1.00
## Proportion Var 0.33
##
## Mean item complexity =  1
## Test of the hypothesis that 1 factor is sufficient.
##
## The degrees of freedom for the null model are  3  and the objective function was  0.29 with Chi Squa
## The degrees of freedom for the model are 0  and the objective function was  0
##
## The root mean square of the residuals (RMSR) is  0
## The df corrected root mean square of the residuals is  NA
##
## The harmonic number of observations is  575 with the empirical chi square  0  with prob <  NA
## The total number of observations was  617  with Likelihood Chi Square =  0  with prob <  NA
##
## Tucker Lewis Index of factoring reliability =  -Inf
## Fit based upon off diagonal values = 1
## Measures of factor score adequacy
```

```
##                                                  MR1
## Correlation of (regression) scores with factors   0.79
## Multiple R square of scores with factors          0.63
## Minimum correlation of possible factor scores     0.26
```

Graded-Response Model: General Functioning

```
##      Extrmt1 Extrmt2 Extrmt3 Extrmt4 Extrmt5 Extrmt6 Dscrmn
## Q27   -2.877  -2.249  -1.441  -0.541   0.313   1.495  1.396
## Q140  -2.067  -1.322  -0.638   0.014   0.774   1.793  1.928
## Q141  -3.260  -2.348  -1.573  -0.998  -0.140   0.909  0.772
```

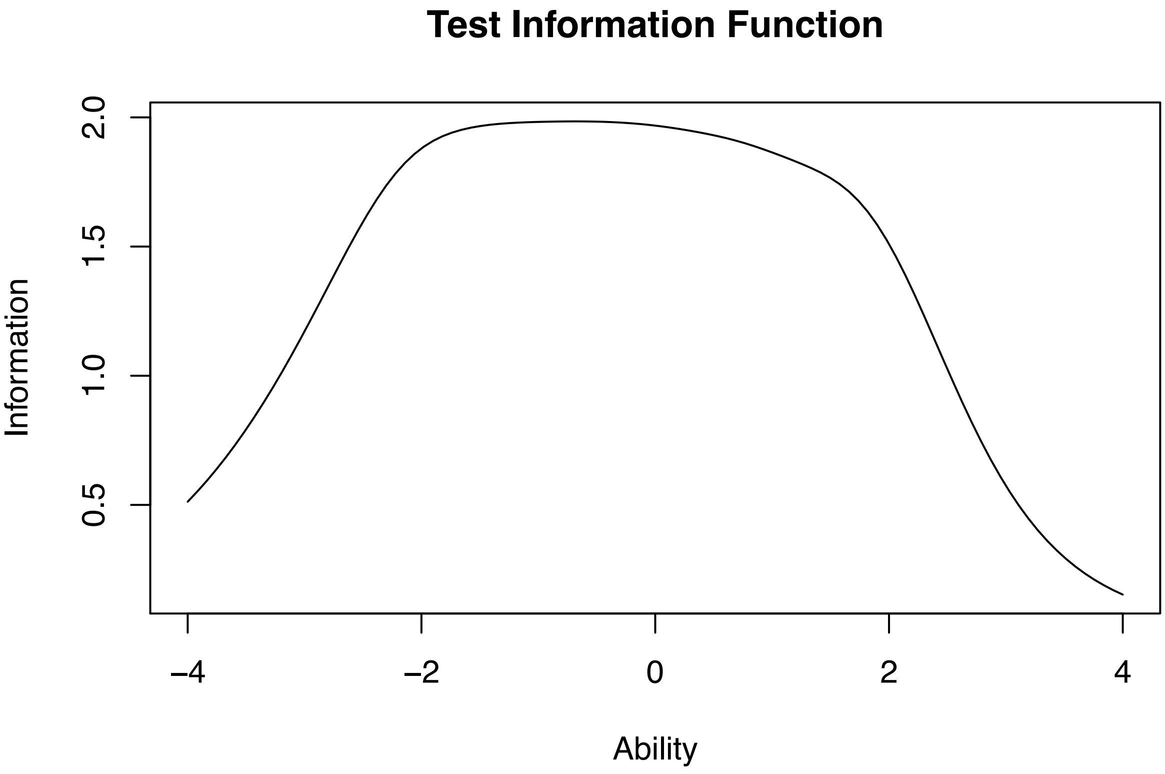

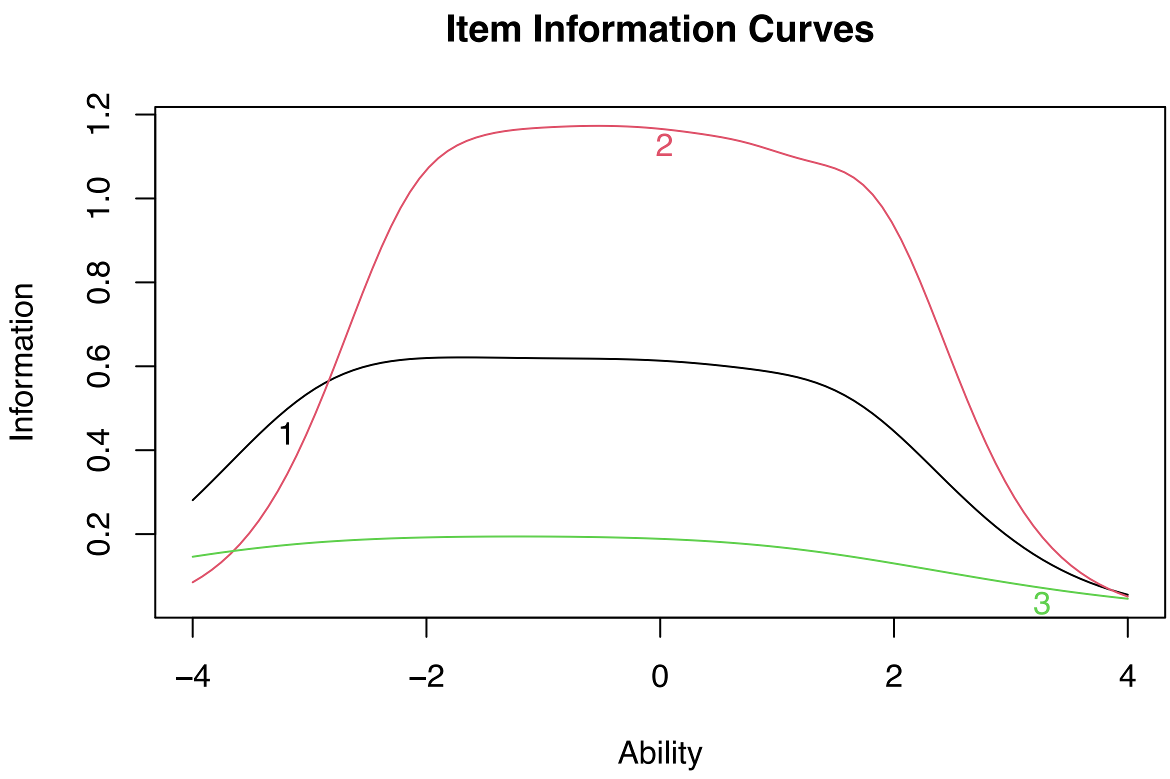

Back to top

Site 2

Reliability: General Functioning

```
## Cronbach's alpha is 0.596.
## Mean item-total correlation is 0.338.
## If each item were dropped:
##      raw_alpha std.alpha G6(smc) average_r  S/N alpha se var.r med.r
## Q27       0.41      0.41    0.26      0.26 0.71    0.047    NA  0.26
## Q140      0.48      0.48    0.32      0.32 0.94    0.042    NA  0.32
## Q141      0.60      0.60    0.43      0.43 1.52    0.032    NA  0.43
```

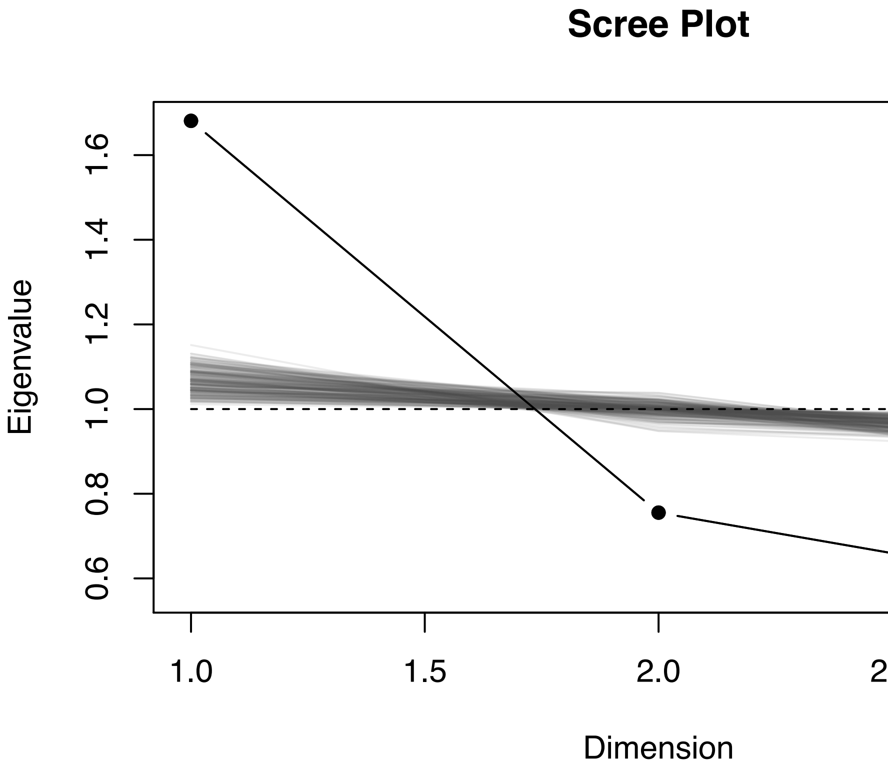

Unidimensionality: General Functioning

```
## [1] "Ratio of first to second eigenvalues: 2.225"
```

```
## [1] 1.6809103 0.7553387 0.5637510
```

```
## Factor Analysis using method =  minres
## Call: fa(r = grm_obj$X)
## Standardized loadings (pattern matrix) based upon correlation matrix
##       MR1   h2   u2 com
## Q27  0.72 0.52 0.48   1
## Q140 0.59 0.35 0.65   1
## Q141 0.44 0.20 0.80   1
##
##                 MR1
## SS loadings    1.07
## Proportion Var 0.36
##
## Mean item complexity =  1
## Test of the hypothesis that 1 factor is sufficient.
##
## The degrees of freedom for the null model are  3  and the objective function was  0.33 with Chi Squa
## The degrees of freedom for the model are 0  and the objective function was  0
##
## The root mean square of the residuals (RMSR) is  0
## The df corrected root mean square of the residuals is  NA
##
## The harmonic number of observations is  548 with the empirical chi square  0  with prob <  NA
## The total number of observations was  596  with Likelihood Chi Square =  0  with prob <  NA
##
## Tucker Lewis Index of factoring reliability =  -Inf
## Fit based upon off diagonal values = 1
## Measures of factor score adequacy
```

```
##                                                    MR1
## Correlation of (regression) scores with factors   0.81
## Multiple R square of scores with factors          0.65
## Minimum correlation of possible factor scores     0.31
```

Graded-Response Model: General Functioning

```
##      Extrmt1 Extrmt2 Extrmt3 Extrmt4 Extrmt5 Extrmt6 Dscrmn
## Q27   -1.684  -1.243  -0.599   0.101   0.728   1.640  1.933
## Q140  -1.953  -1.189  -0.387   0.317   1.161   2.296  1.503
## Q141  -2.124  -1.291  -0.639   0.087   0.912   1.693  0.953
```

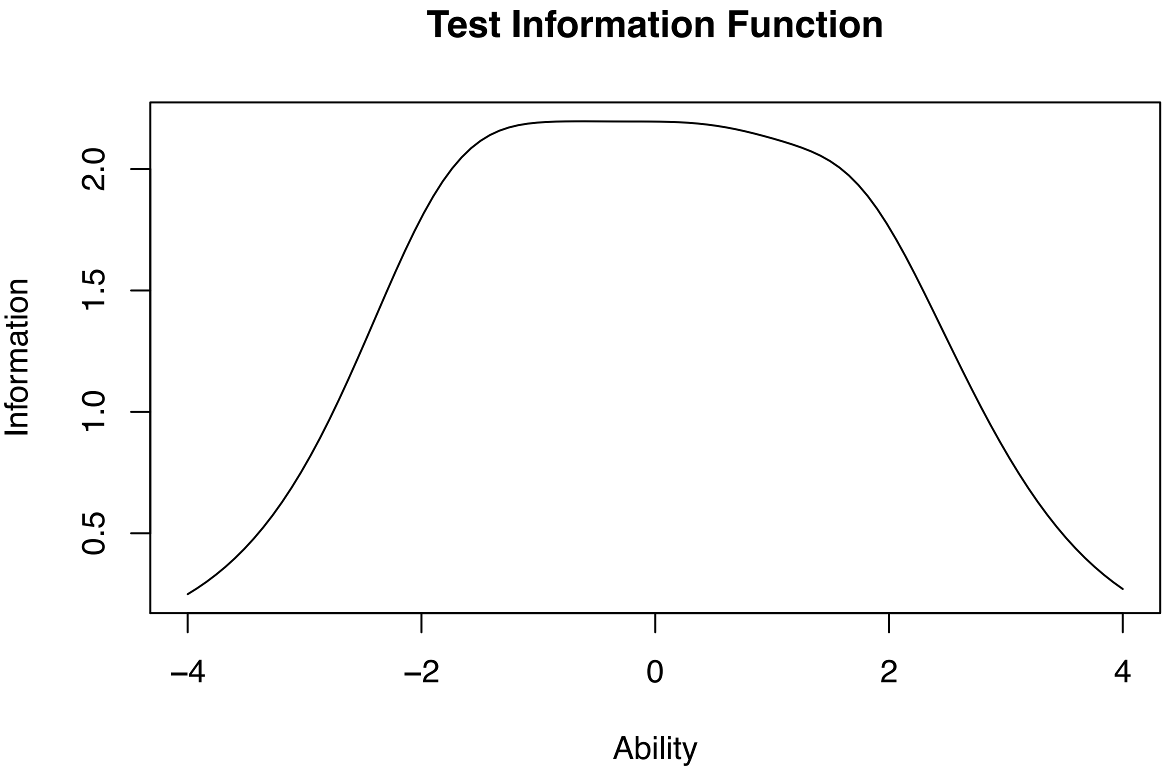

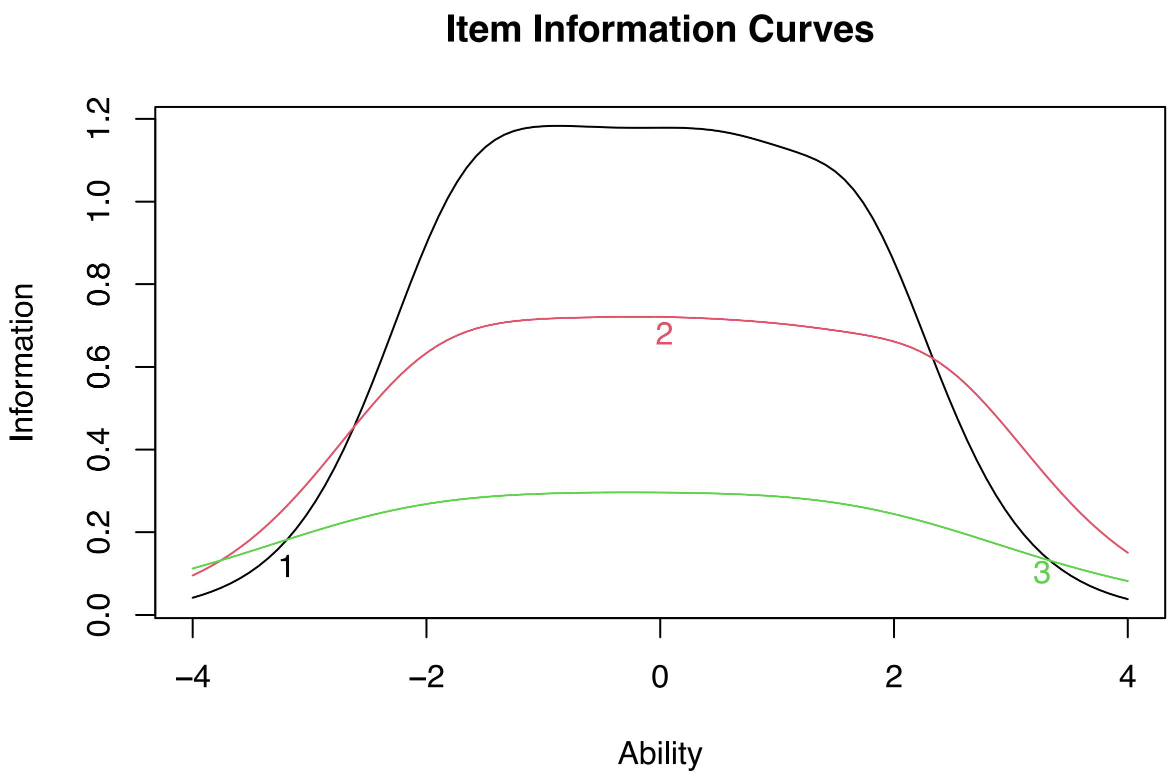

Back to top

Gender-based DIF: General Functioning

```
## No Gender-based DIF detected
```

Age-based DIF: General Functioning

```
## No age-based DIF detected
```

Back to top

Substance Recovery

Site 1

Reliability: Substance Recovery

```
## Cronbach's alpha is 0.503.
## Mean item-total correlation is 0.22.
## If each item were dropped:
##      raw_alpha std.alpha G6(smc) average_r  S/N alpha se   var.r med.r
## Q109      0.34      0.38    0.32      0.17 0.61    0.046 0.02251  0.14
## Q154      0.35      0.37    0.30      0.16 0.58    0.044 0.01800  0.15
## Q108      0.43      0.45    0.37      0.22 0.83    0.039 0.01512  0.15
## Q155      0.58      0.60    0.50      0.33 1.48    0.028 0.00078  0.33
```

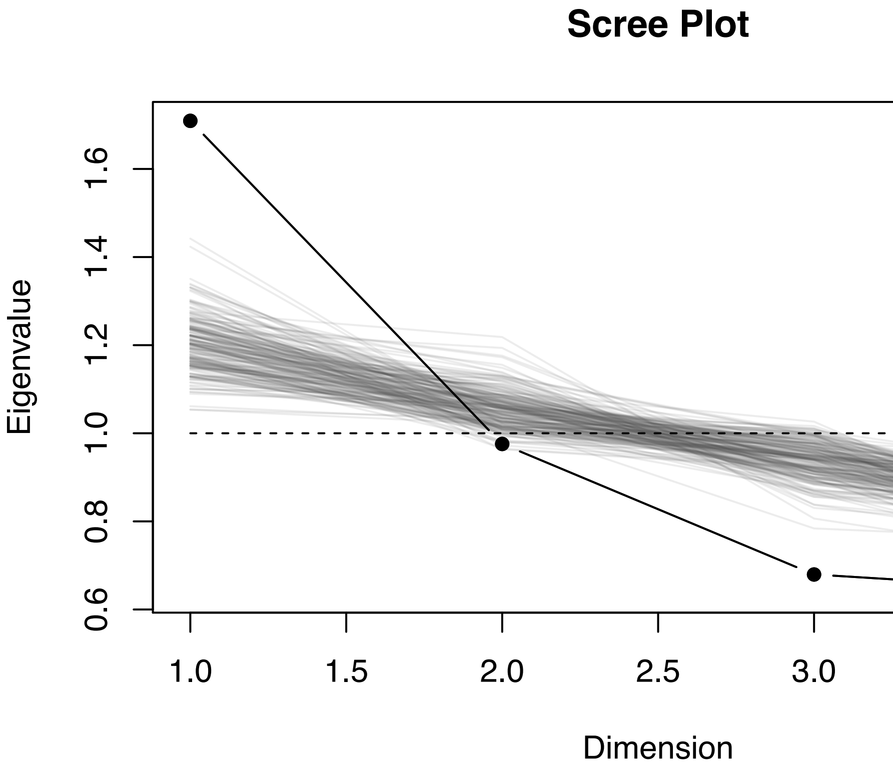

Unidimensionality: Substance Recovery

```
## [1] "Ratio of first to second eigenvalues: 1.752"

## [1] 1.7091740 0.9757107 0.6794088 0.6357065

## Factor Analysis using method =  minres
## Call: fa(r = grm_obj$X)
## Standardized loadings (pattern matrix) based upon correlation matrix
##       MR1    h2   u2 com
## Q109 0.61 0.368 0.63   1
## Q154 0.62 0.388 0.61   1
## Q108 0.49 0.240 0.76   1
## Q155 0.20 0.039 0.96   1
##
##                 MR1
## SS loadings    1.04
## Proportion Var 0.26
##
## Mean item complexity =  1
## Test of the hypothesis that 1 factor is sufficient.
##
## The degrees of freedom for the null model are  6  and the objective function was  0.33 with Chi Squa
## The degrees of freedom for the model are 2  and the objective function was  0.01
##
## The root mean square of the residuals (RMSR) is  0.03
## The df corrected root mean square of the residuals is  0.05
##
## The harmonic number of observations is  129 with the empirical chi square  1.35  with prob <  0.51
## The total number of observations was  617  with Likelihood Chi Square =  5.81  with prob <  0.055
##
## Tucker Lewis Index of factoring reliability =  0.941
## RMSEA index =  0.056  and the 90 % confidence intervals are  0 0.111
```

```
## BIC =  -7.04
## Fit based upon off diagonal values = 0.99
## Measures of factor score adequacy
##                                                   MR1
## Correlation of (regression) scores with factors   0.78
## Multiple R square of scores with factors          0.61
## Minimum correlation of possible factor scores     0.22
```

Graded-Response Model: Substance Recovery

```
##      Extrmt1 Extrmt2 Extrmt3 Extrmt4 Extrmt5 Extrmt6 Dscrmn
## Q109  -0.560   0.515   1.032   1.451   1.827   2.743  1.149
## Q154   0.653   1.265   1.471   1.683   1.732   1.962  3.627
## Q108  -0.298   0.952   1.774   2.772   3.164   3.793  1.187
## Q155  -1.123   0.882   1.668   2.670   3.335   4.046  0.565
```

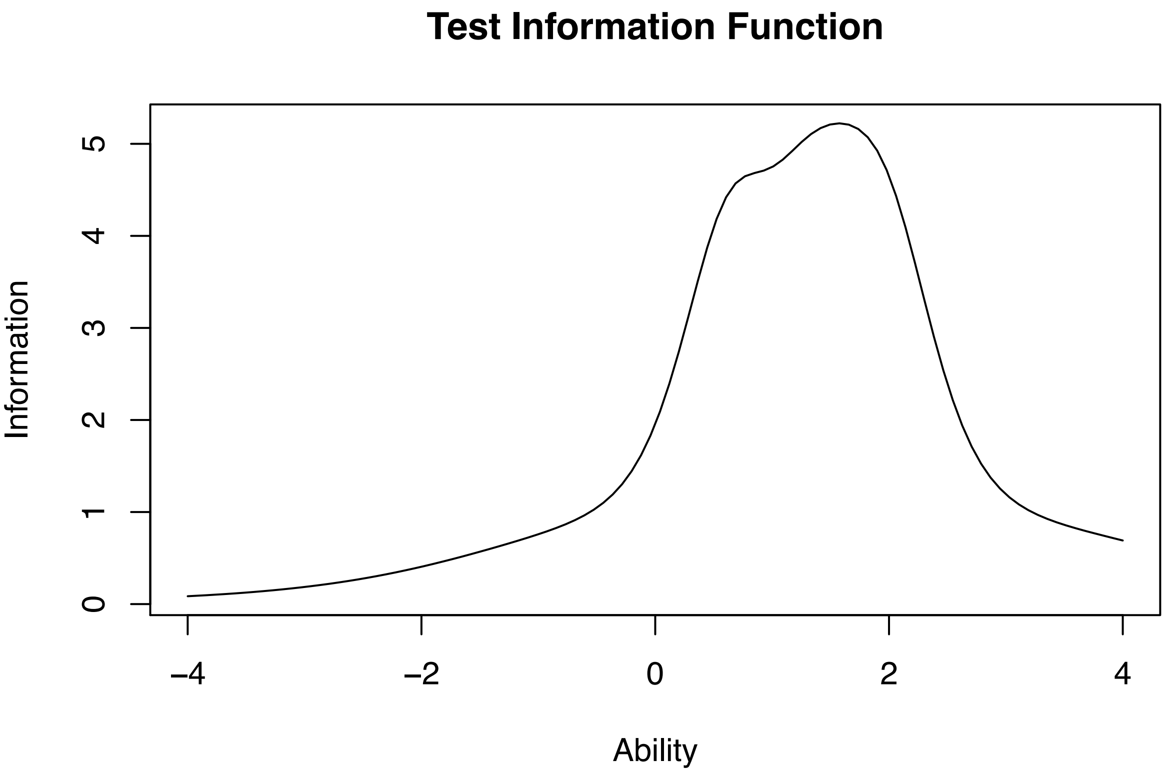

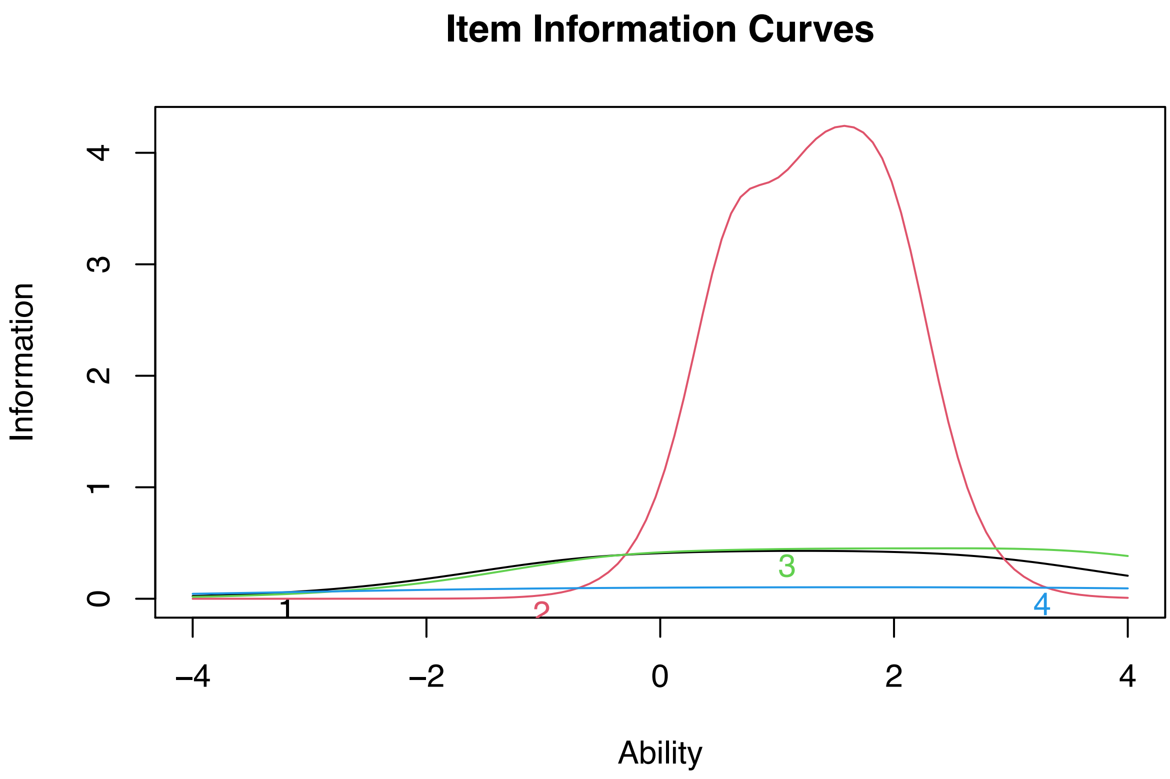

Back to top

Site 2

Did not converge.

Gender-based DIF: Substance Recovery

```
## Call:
## lordif::lordif(resp.data = as.data.frame(sex.data), group = sex)
##
##   Number of DIF groups: 2
##
##   Number of items flagged for DIF: 0 of 4
##
##   Items flagged:
##
##   Number of iterations for purification: 1 of 10
##
##   Detection criterion: Chisqr
##
##   Threshold: alpha = 0.01
```

Age-based DIF: Substance Recovery

```
## Call:
## lordif::lordif(resp.data = as.data.frame(age.data), group = age)
```

```
##
##   Number of DIF groups: 2
##
##   Number of items flagged for DIF: 0 of 4
##
##   Items flagged:
##
##   Number of iterations for purification: 1 of 10
##
##   Detection criterion: Chisqr
##
##   Threshold: alpha = 0.01
```

Back to top

Session Information

Functions

Here is the code for the relevant custom functions created at the start of this analysis script. They are only reproduced here for presentation, not re-run here.

```
# Test information function
tif <- function(x){
  # x is a grm object
  plot(x, type = "IIC", items = 0, zrange = c(-4, 4), plot = TRUE)
}

# Item information curve
iic <- function(x){
  # x is a grm object
  plot(x, type = "IIC", zrange = c(-4, 4), plot = TRUE)
}

# IRT summary convenience function
irt_summary <- function(x){
  # x is a grm object from package `ltm`
  print(coef(x))
  tif(x)
  iic(x)
}

# Internal consistency convenience function
alpha_summary <- function(x){
  # x is an object from ltm::grm()
  if(class(x) != "grm"){
    warning("Not an object of type 'grm'.")
  }
  temp_alpha <- psych::alpha(x$X, check.keys = TRUE)
  alph <- temp_alpha$total$raw_alpha
  r_item_tot <- temp_alpha$total$average_r
  cat("Cronbach's alpha is ", round(alph, 3), ". \nMean item-total correlation is ",
      round(r_item_tot, 3), ".\nIf each item were dropped: \n", sep = "")
```

```
  print(temp_alpha$alpha.drop, digits = 2)
  invisible(temp_alpha)
  # The function will always print the raw alpha and mean item-total correlation when run.
  # If called as a saved object, however, it functions as the output of a call to psych::alpha.
}

# ROC curve convenience function
roc_summary <- function(x){
  # x is an roc object from {pROC}
  plot(x)
  auc(x)
}

# Unidimensionality: Scree plot with simulated random data sets

# This is the complete function scree.plot() from {psy} version 1.1 by Bruno Falissard.
# Bruno Falissard (2012). psy: Various procedures used in
  # psychometry. R package version 1.1.
  # https://CRAN.R-project.org/package=psy
# Borrowing it here so as not to require the package, with altered default values
# that include simulations and pairwise comparisons.
scree.plot2 <- function (namefile, title = "Scree Plot", type = "R", use = "P", simu = 200)
{
    mat <- namefile
    if (use == "complete.obs")
        mat <- na.omit(namefile)
    if (type == "R")
        eigenval <- eigen(cor(mat, use = "pairwise.complete.obs"),
            symmetric = TRUE)$values
    if (type == "V")
        eigenval <- eigen(cov(mat, use = "pairwise.complete.obs"),
            symmetric = TRUE)$values
    if (type == "E")
        eigenval <- namefile
    if (type == "M")
        eigenval <- eigen(namefile, symmetric = TRUE)$values
    nev <- length(eigenval)
    ### Added this code, using the eigenvalues later:
    eigenout <- eigenval
    ###
    plot(eigenval, type = "b", pch = 16, bty = "o", main = title,
        xlab = "Dimension", ylab = "Eigenvalue")
    # lines(c(1, nev), c(1, 1), lty = 2)  # AAM moving this later
    if (is.numeric(simu) && (type == "R")) {
        n <- dim(mat)[1]
        p <- dim(mat)[2]
        matsimu <- matrix(nrow = n, ncol = p)
        int <- rep(1, n * p)
        attr(int, "dim") <- c(n, p)
        mat <- pmax(as.matrix(mat), int)
        for (i in 1:simu) {
            matnorm <- rnorm(n * p)
            attr(matnorm, "dim") <- c(n, p)
```

```
            matsimu <- (mat/mat) * matnorm
            eigenval <- eigen(cor(matsimu, use = "pairwise.complete.obs"))$values
            points(eigenval, type = "l", col = gray(.3, .1))  # AAM changed col value.
        }
    }
    lines(c(1, nev), c(1, 1), lty = 2)  # AAM moved this here for visibility
    points(eigenout, type = "b", pch = 16, bty = "o")  # AAM added this as well.
    ### added this code:
    print(paste0("Ratio of first to second eigenvalues: ",
                 round((eigenout[1]/eigenout[2]), 3)))
    eigenout
    ###
}

# lordif() printing function
# All this does is removes the Quartz viewer device from the existing lordif::plot.lordif() function.
# This is necessary to create the report that you are reading, the code changes
# do not alter the analysis from the existing lordif package function.
plot2.lordif <- function (x, labels = c("Reference", "Focal"), width = 7, height = 7,
                          ...)
{
  ndif <- sum(x$flag)
  if (ndif == 0)
    stop(paste(deparse(substitute(x))), " contains no items flagged for DIF")
  if (ndif == x$ni)
    stop("all items in ", paste(deparse(substitute(x))),
         " have been flagged for DIF")
  if (x$ng != length(labels))
    labels <- paste("Group", 1:x$ng)
  sumpp <- function(pp) {
    ws <- rowSums(pp * (col(pp) - 1))
    return(ws)
  }
  maxcat <- ncol(x$ipar.sparse)
  # Made into a comment by AAM
  # sysname <- Sys.info()[["sysname"]]
  # if (sysname == "Windows") {
  #   dev.new(width = width, height = height, record = TRUE)
  # }
  # else if (sysname == "Linux") {
  #   dev.new(width = width, height = height)
  #   par(ask = TRUE)
  # }
  # else {
  #   dev.new(width = width, height = height)
  # }
  par(mfrow = c(1, 1))
  theta <- seq(x$options$minTheta, x$options$maxTheta, x$options$inc)
  difitems <- (1:x$ni)[x$flag]
  difselections <- x$selection[x$flag]
  itemnames <- row.names(x$ipar.sparse)
  gpar <- array(NA, c(ndif, maxcat, x$ng))
  cpar <- as.matrix(x$ipar.sparse[1:(x$ni - ndif), ])
```

```
pp <- array(NA, c(length(theta), ndif, maxcat, x$ng))
gtheta <- split(x$calib.sparse$theta, x$group)
gdensity <- matrix(0, length(theta), x$ng)
for (i in 1:x$ng) {
  gdensity[, i] <- density(unlist(gtheta[names(table(x$group))[i]]),
                           n = length(theta),
                           from = x$options$minTheta,
                           to = x$options$maxTheta,
                           bw = 0.25)$y
}
plot(theta, gdensity[, 1], type = "l", xlab = "theta", ylab = "Density",
     ylim = c(0, max(gdensity)), lty = 1, col = 1, main = "Trait Distributions",
     ...)
for (g in 2:x$ng) {
  lines(theta, gdensity[, g], lty = g, col = g)
}
legend("topright", labels, lty = 1:x$ng, col = 1:x$ng, bg = "white")
par(mfrow = c(2, 2))
for (i in 1:length(difitems)) {
  ncat <- x$ncat[difitems[i]]
  plot(theta, seq(0, ncat - 1, along.with = theta), type = "n",
       xlab = "theta", ylab = "Item Score",
       main = paste0("Item True Score Functions - Item ",
                     difselections[i]), ...)
  for (g in 1:x$ng) {
    gpar[i, , g] <- unlist(x$ipar.sparse[which(itemnames ==
                                                  paste0("I",
                                                         difselections[i],
                                                         ".", g)), ])
    if (x$options$model == "GPCM")
      pp[, i, 1:ncat, g] <- lordif::probgpcm(theta, gpar[i,
                                                  1, g], gpar[i, 2:ncat, g])
    else pp[, i, 1:ncat, g] <- lordif::probgrm(theta, gpar[i,
                                                  1, g], gpar[i, 2:ncat, g])
    lines(theta, sumpp(pp[, i, 1:ncat, g]), lty = g,
          col = g)
  }
  legend("bottomright", labels, lty = 1:x$ng, col = 1:x$ng,
         cex = 0.7, bg = "white")
  chi12 <- paste(x$stats[difitems[i], "df12"], ")=", x$stats[difitems[i],
                                                             "chi12"], sep = "")
  pseudo12 <- x$stats[difitems[i], paste("pseudo12.", x$options$pseudo.R2,
                                         sep = "")]
  beta12 <- round(x$stats[difitems[i], "beta12"], 4)
  chi13 <- paste(x$stats[difitems[i], "df13"], ")=", x$stats[difitems[i],
                                                             "chi13"], sep = "")
  pseudo13 <- x$stats[difitems[i], paste("pseudo13.", x$options$pseudo.R2,
                                         sep = "")]
  chi23 <- paste(x$stats[difitems[i], "df23"], ")=", x$stats[difitems[i],
                                                             "chi23"], sep = "")
  pseudo23 <- x$stats[difitems[i], paste("pseudo23.", x$options$pseudo.R2,
                                         sep = "")]
  text(min(theta), ncat - 1, substitute(paste("Pr(", chi[12]^2,
```

```
                                              ",", chi12, ",", R[12]^2, "=",
                                              pseudo12, ",", Delta,
                                              "(", beta[1], ")=", beta12,
                                              sep = "")), adj = c(0,
                                                                  1), cex = 0.8)
    text(min(theta), (ncat - 1) * 0.9,
         substitute(paste("Pr(",
                          chi[13]^2, ",", chi13, ",", R[13]^2, "=", pseudo13,
                                                        sep = "")), adj = c(0, 1), cex = 0.8)
    text(min(theta), (ncat - 1) * 0.8,
         substitute(paste("Pr(",
                          chi[23]^2, ",", chi23, ",", R[23]^2, "=", pseudo23,
                          sep = "")), adj = c(0, 1), cex = 0.8)
    plot(theta, seq(0, ncat - 1, along.with = theta), type = "n",
         xlab = "theta", ylab = "Item Score", main = "Differences in Item True Score Functions",
         ...)
    for (g in 2:x$ng) {
      lines(theta, abs(sumpp(pp[, i, 1:ncat, 1]) - sumpp(pp[,
                                                            i, 1:ncat, g])), lty = g, col = g)
    }
    plot(theta, seq(0, 1, along.with = theta), type = "n",
         xlab = "theta", ylab = "Probability", main = "Item Response Functions",
         ...)
    for (g in 1:x$ng) {
      for (k in 1:ncat) {
        lines(theta, pp[, i, k, g], lty = g, cex = 0.1,
              col = g)
      }
    }
    for (g in 1:x$ng) {
      text(x$options$minTheta, 0.8 - (g - 1) * par()$cxy[2],
           paste(round(gpar[i, , g][!is.na(gpar[i, , g])],
                       2), collapse = ", "), col = g, adj = c(0, 0),
           cex = 0.8)
      for (k in 2:ncat) {
        if (!is.na(gpar[i, k, g]))
          text(gpar[i, k, g], 0, "|", col = g)
      }
    }
    plot(theta, seq(0, ncat - 1, along.with = theta), type = "n",
         xlab = "theta", ylab = "Size", main = "Impact (Weighted by Density)",
         ...)
    for (g in 2:x$ng) {
      lines(theta, gdensity[, g] * abs(sumpp(pp[, i, 1:ncat,
                                              1]) - sumpp(pp[, i, 1:ncat, g])),
            lty = g, col = g)
    }
  }
  par(mfrow = c(1, 2))
  plot(theta, seq(0, sum(!is.na(x$ipar)) - x$ni, along = theta),
       xlab = "theta", ylab = "TCC", type = "n", main = "All Items",
       ...)
  for (g in 1:x$ng) {
```

```
    apar <- rbind(cpar, gpar[, , g])
    lines(theta, lordif::tcc(apar[, 1], apar[, -1, drop = F], theta,
                     model = x$options$model), lty = g, col = g)
  }
  legend("bottomright", labels, lty = 1:x$ng, col = 1:x$ng,
         bg = "white")
  plot(theta, seq(0, sum(!is.na(gpar[, , 1])) - ndif, along = theta),
       xlab = "theta", ylab = "TCC", type = "n", main = "DIF Items",
       ...)
  for (g in 1:x$ng) {
    lines(theta, lordif::tcc(gpar[, 1, g], matrix(gpar[, -1, g],
                                            nrow = ndif), theta, model = x$options$model),
          lty = g,
          col = g)
  }
  legend("bottomright", labels, lty = 1:x$ng, col = 1:x$ng,
         bg = "white")
  layout(matrix(c(1, 2), ncol = 2), widths = c(1, 2))
  boxplot(x$calib$theta - x$calib.sparse$theta, col = "light grey")
  difference <- x$calib$theta - x$calib.sparse$theta
  plot(x$calib$theta, difference, type = "n", xlab = "initial theta",
       ylab = "initial - purified", ...)
  abline(h = 0)
  abline(h = mean(x$calib$theta - x$calib.sparse$theta), lty = 2)
  for (i in 1:x$ng) {
    points(x$calib$theta[x$group == as.numeric(names(table(x$group))[i])],
           difference[x$group == as.numeric(names(table(x$group))[i])],
           col = i, pch = i)
  }
  legend("topright", labels, pch = 1:x$ng, col = 1:x$ng, bg = "white")

  # Need to reset the layout so that future plots don't also have the same layout.
  layout(matrix(1))  # Added by AAM, 5/13/19
}

# making function to reverse score the integers
rev_score <- function(x){
  x * -1 + 8
}

# this interactively reverse codes items prior to scale analysis, only used for data
# combination reasons.
# More specifically, {mirt} and {ltm} have slightly different rules for automatically
# reverse-scoring items with negative correlations, which results in variable appearances for
# IRT parameters. Also, the different data collection and storage required across the samples
# in the study had different procedures for reverse scoring.
# The goal for this function is to standardize that reverse scoring across samples and analysis
# packages, so that it will reverse score any items that *should* be reverse scored based on
# empirical results.
# takes a tibble, returns one.
reverse_onfly <- function(data){
  x <- cor(data)
  while(any(x < -.1)){  # are there any negative cells in the correlation matrix?
```

```r
    # Assumes full data, no NA.
    # Do this over and over again until it's not true:
    indices <- which(cor(data) < -.1, arr.ind = TRUE)
    # Note: allows for some small negative correlations, which would be very bad item combinations
    name_quo <- rownames(indices)[1]
    print(paste("name_quo ", name_quo))
    name_use <- as.name(name_quo)
    print(paste("name_use", name_use))
    data <- mutate(data, !!name_use := rev_score(!!name_use))
    x <- cor(data)
  }
  data # returns the data frame.
}
```

Session Info

```
## R version 4.0.2 (2020-06-22)
## Platform: x86_64-apple-darwin17.0 (64-bit)
## Running under: macOS Mojave 10.14.6
##
## Matrix products: default
## BLAS:   /Library/Frameworks/R.framework/Versions/4.0/Resources/lib/libRblas.dylib
## LAPACK: /Library/Frameworks/R.framework/Versions/4.0/Resources/lib/libRlapack.dylib
##
## locale:
## [1] en_US.UTF-8/en_US.UTF-8/en_US.UTF-8/C/en_US.UTF-8/en_US.UTF-8
##
## attached base packages:
## [1] stats4    stats     graphics  grDevices utils     datasets  methods
## [8] base
##
## other attached packages:
##  [1] forcats_0.5.0   stringr_1.4.0   purrr_0.3.4     readr_1.4.0
##  [5] tidyr_1.1.2     tibble_3.0.4    tidyverse_1.3.0 NORSEpkg_0.1.0
##  [9] dplyr_1.0.2     lordif_0.3-3    rms_6.0-1       SparseM_1.78
## [13] Hmisc_4.4-1     ggplot2_3.3.2   Formula_1.2-4   survival_3.2-7
## [17] mirt_1.33.2     lattice_0.20-41 pROC_1.16.2     psych_2.0.9
## [21] ltm_1.1-1       polycor_0.7-10  msm_1.6.8       MASS_7.3-53
## [25] rmarkdown_2.4   knitr_1.30
##
## loaded via a namespace (and not attached):
##  [1] TH.data_1.0-10        colorspace_1.4-1      ellipsis_0.3.1
##  [4] htmlTable_2.1.0       base64enc_0.1-3       fs_1.5.0
##  [7] rstudioapi_0.11       Deriv_4.1.1           MatrixModels_0.4-1
## [10] fansi_0.4.1           mvtnorm_1.1-1         lubridate_1.7.9
## [13] xml2_1.3.2            codetools_0.2-16      splines_4.0.2
## [16] mnormt_2.0.2          jsonlite_1.7.1        broom_0.7.1
## [19] cluster_2.1.0         dbplyr_1.4.4          png_0.1-7
## [22] compiler_4.0.2        httr_1.4.2            backports_1.1.10
## [25] assertthat_0.2.1      Matrix_1.2-18         cli_2.1.0
## [28] htmltools_0.5.0       quantreg_5.73         tools_4.0.2
## [31] gtable_0.3.0          glue_1.4.2            Rcpp_1.0.5
## [34] cellranger_1.1.0      vctrs_0.3.4           nlme_3.1-149
```

```
## [37] conquer_1.0.2         xfun_0.18             rvest_0.3.6
## [40] lifecycle_0.2.0       dcurver_0.9.2         polspline_1.1.19
## [43] zoo_1.8-8             scales_1.1.1          hms_0.5.3
## [46] parallel_4.0.2        sandwich_3.0-0        expm_0.999-5
## [49] RColorBrewer_1.1-2    yaml_2.2.1            gridExtra_2.3
## [52] rpart_4.1-15          latticeExtra_0.6-29   stringi_1.5.3
## [55] checkmate_2.0.0       permute_0.9-5         rlang_0.4.8
## [58] pkgconfig_2.0.3       matrixStats_0.57.0    evaluate_0.14
## [61] htmlwidgets_1.5.2     tidyselect_1.1.0      plyr_1.8.6
## [64] magrittr_1.5          R6_2.4.1              generics_0.0.2
## [67] multcomp_1.4-14       DBI_1.1.0             pillar_1.4.6
## [70] haven_2.3.1           foreign_0.8-80        withr_2.3.0
## [73] mgcv_1.8-33           nnet_7.3-14           modelr_0.1.8
## [76] crayon_1.3.4          tmvnsim_1.0-2         jpeg_0.1-8.1
## [79] grid_4.0.2            readxl_1.3.1          data.table_1.13.0
## [82] blob_1.2.1            vegan_2.5-6           reprex_0.3.0
## [85] digest_0.6.25         GPArotation_2014.11-1 munsell_0.5.0
```
